# Systematic analysis of expression profiles of HMGB family members for prognostic application in non-small cell lung cancer

**DOI:** 10.3389/fmolb.2022.844618

**Published:** 2022-07-18

**Authors:** Xuefeng Zheng, Xu Wang, Yunduan He, Hong Ge

**Affiliations:** Department of Radiation Oncology, The Affiliated Cancer Hospital of Zhengzhou University & Henan Cancer Hospital, Zhengzhou, China

**Keywords:** non-small cell lung cancer, HMGB family, bioinformatics analysis, prognostic value, biomaker

## Abstract

**Background:** Lung cancer is a significant challenge to human health. Members of the high mobility group (HMG) superfamily (HMGB proteins) are implicated in a wide variety of physiological and pathophysiological processes, but the expression and prognostic value of HMGB family members in non-small cell lung cancer (NSCLC) have not been elucidated.

**Methods:** In this study, ONCOMINE, UALCAN, GEPIA, Kaplan–Meier Plotter, starBase, OncomiR databases, and GeneMANIA were utilized to evaluate the prognostic significance of HMGB family members in NSCLC.

**Results:** HMGB2/3 expression levels were higher in NSCLC patients. HMGB1 expression was higher in lung squamous cell carcinoma (LUSC) and was lower in lung adenocarcinoma (LUAD) tissue than in normal lung tissue. HMGB2 expression was related to cancer stage. Increased HMGB1 mRNA expression levels were associated with improved lung cancer prognosis, including overall survival (OS), first-progression survival (FP), and post-progression survival (PPS). There was no significant association between HMGB2 levels and prognostic indicators. HMGB3 expression was associated with poorer OS. GeneMANIA and GO/KEGG pathway analysis showed that HMGB family members mainly associated with chromosome condensation, regulation of chromatin organization, and nucleosome binding in NSCLC. HMGBs expression were closely correlated with infiltrating levels of specific types of immune cells in NSCLC, especially Th2 cells, Th17 cells, and mast cells. hsa-miR-25-3p, hsa-miR-374a-3p, and hsa-miR-93-5p were significantly positively correlated with HMGB1, HMGB2, and HMGB3, respectively. However, hsa-miR-30a-5p was predicted to significantly negatively regulate HMGB3 expression.

**Conclusion:** Our study revealed that HMGB1 is positively related to the improved prognosis in NSCLC, and demonstrate that HMGB3 might be a risk factor for poorer survival of NSCLC patients.

## Introduction

Lung cancer is one of the most common malignant tumors and poses a severe threat to human health. Recent data from the International Agency for Research on Cancer (IARC) revealed that lung cancer accounted for the highest number of cancer-related deaths worldwide, and was responsible for nearly 1.8 million deaths in 2020, more than any other type of cancers (Baltayiannis et al., 2013; Bray et al., 2018). Small cell lung cancer (SCLC) and non-small cell lung cancer (NSCLC) are the two main histological types of lung cancer. NSCLC is the most common type of lung cancer, accounting for 85% of all lung cancer cases. NSCLC is further subtyped into adenocarcinomas (ADC; which comprise ∼40–50% of NSCLC), squamous cell carcinomas (SCC; which comprise ∼20–40% of NSCLC), and large cell carcinomas (LGC; which comprise ∼20% of NSCLC) (Wang Y et al., 2019; Relli et al., 2019). Despite the significant efforts devoted to the study of NSCLC, the exact underlying mechanisms that contribute to the development, progression, and recurrence of NSCLC remain to be clearly elucidated. Therefore, it is urgent to identify new highly specific and sensitive biomarkers and new molecular targets to improve care and outcomes for patients with NSCLC.

HMGB proteins are members of the high mobility group (HMG) superfamily. This family includes four members: HMGB1, HMGB2, HMGB3, and HMGB4 (Taniguchi et al., 2018; Niu et al., 2020; Wen et al., 2021). HMGB1/2/3 proteins are composed of two DNA-binding HMGB-boxes and an acidic tail. HMGB4 lacks the acidic tail and its nucleotide sequence is less well-conserved than the sequences of the other HMGB family members (Taniguchi et al., 2018; Niu et al., 2020; Wen et al., 2021). Since research into HMGB4 is limited, we mainly focused on the role of HMGB1/2/3 in NSCLC. HMGBs have been implicated in a wide variety of physiological and pathophysiological processes, involving infection, injury, and inflammation by regulating DNA replication, transcription, recombination, and repair (Niu et al., 2020). HMGBs also are known to play important role in tumorigenesis and cancer progression, including NSCLC (Taniguchi et al., 2018; Qiu et al., 2020; Wen et al., 2021).

Abnormal expression of some HMGB family members has been reported in NSCLC, and several HMGB genes have prognostic value in NSCLC. Qiu et al. (2020) demonstrated that HMGB1 is highly expressed in NSCLC tissue compared with normal tissue, and showed that HMGB1 facilitates NSCLC progression via activating the Wnt/β-catenin pathway. In addition, Wang et al. (2014) reported that HMGB1 was significantly and positively associated with tumor grade and stage in NSCLC, was positively associated with MMP-9, and may serve as a prognostic and predictive marker for NSCLC. Li S et al. (2018) showed a potential mechanism underlying the oncogenic role of HMGB2 in NSCLC development through the miR-181a-5p/HMGB2 axis. Similarly, Lou et al. (2021) found that HMGB2 protein expression was higher in tumor tissue compared with adjacent tissue, and correlated with lymph node (LYN) metastasis and advanced TNM stage in NSCLC, indicating a poor prognosis. HMGB3 is highly expressed in a variety of cancers, including breast cancer, gastric adenocarcinoma, lung cancer, bladder cancer, esophageal cancer, glioma, and prostate cancer (Niu et al., 2020; Wen et al., 2021). Song et al. (2019) and Xie et al. (2020) demonstrated that HMGB3 could accelerate the proliferation and colony formation of NSCLC cells, and reduced apoptosis of NSCLC cells regulated by circEPSTI1/miR-145, suggesting that HMGB3 may be a novel independent prognostic marker of worse outcome for NSCLC patients (Song et al., 2013).

These reports suggest that HMGB1/2/3 all act as oncogenes in NSCLC, but some other studies have contradicted these findings. Kang et al. (2013) and Luan et al. (2017) indicated that HMGB1 acts as tumor suppressor in pancreatic cancer and endometrial carcinoma. Furthermore, higher mRNA expression of HMGB1 led to a positive prognosis in lung cancer patients (Tang et al., 2010; Wu et al., 2018). In addition, Niu et al. (2020) reported that overexpression of HMGB2 associated with longer OS in cancers including invasive breast carcinoma (BRCA), cervical squamous cell carcinoma/endocervical adenocarcinoma (CESC), LUSC, rectal adenocarcinoma (READ), stomach adenocarcinoma (STAD), and thyroid carcinoma (THCA). In summary, the role of distinct HMGB family members remains unknown, especially regarding the development and progression of NSCLC. In the present study, bioinformatics analyses were performed to analyze the expression, prognosis, and mutations of different HMGB family members and to determine the relationship between HMGB family members and cancer stage in lung cancer patients. Additionally, we evaluated microRNA-HMGB interaction maps and explored the correlation of HMGB levels with tumor-infiltrating immune cells in NSCLC.

## Materials and methods

### Ethics statement

The study was approved by the Institutional Review Board of Zhengzhou University. All data were retrieved from the online databases, and written informed consent had already been obtained from patients prior to the deposition information or data in those databases.

### ONCOMINE database

ONCOMINE is a cancer microarray database and web-based data-mining platform, which provides tools for genome-wide expression analysis (Rhodes et al., 2004). Using ONCOMINE, transcription levels of HMGB family members were compared between cancer tissues and corresponding adjacent normal tissues. A *p*-value of *p* < 0.05, a fold change of 2, and a gene rank in the top 10% were set as the significance thresholds.

### UALCAN

UALCAN is an interactive web-portal used to perform to in-depth analyses of TCGA gene expression data. In our study, UALCAN was used to illustrate the distinct expression levels of HMBG family members in tumor and normal tissues. Differences were evaluated with Student’s *t*-tests, and a *p*-value of *p* < 0.05 was set as the significant level.

### GEPIA

GEPIA is a web-based tool used deliver fast and customizable functionalities based on TCGA and GTEx data, and is freely available to all users. All the datasets on the server are computed by a standard pipeline and are compatible with each other. GEPIA covers a series of functions, including differential expression analysis, patient survival analysis, and correlation analysis (Tang et al., 2017). In this study, “Expression DIY-Stage Plot” was utilized to evaluate the relationship between HMGB family members and tumor stage. Differences were evaluated with Student’s *t*-test, and a *p*-value of *p* < 0.05 was set as the significant level.

### Kaplan–Meier Plotter

The Kaplan-Meier method is the most common way to estimate survival times and probabilities. The Kaplan-Meier Plotter database can evaluate the effects of 54,000 genes on survival in 21 cancer types including breast, ovarian, lung, and gastric cancer (Nagy et al., 2018). Sources for the databases include GEO, EGA, and TCGA. In this study, KM Plotter was used to analyze the prognostic value of mRNA expression of HMGB family members in lung cancer in which cancer patients were split into high- and low- expression group, based on median expression values. Differences between groups were considered to be significantly different with a log-rank test *p*-value of *p* < 0.05.

### Gene MANIA

Gene MANIA is a flexible, user-friendly web interface for searching genes that are related to a set of input genes, using a large set of functional association data. Association data include protein and genetic interactions, pathways, co-expression, co-localization, and protein domain similarity. Using Gene MANIA, we input “HMGB1; HMGB2; HMGB3” and analyzed by using the automatically selected weighting method in *Homo sapiens*.

### Starbase

Starbase is a pan-cancer database used to identify interaction networks of lncRNAs, microRNAs, RNA-binding proteins, and mRNAs from large-scale CLIP-Seq data and tumor samples. In the “Target Gene” section, we obtained HMGB-related microRNAs by sequentially inputting “HMGB1, HMGB2, and HMGB3” genes into starBase. We then verified the correlation of each microRNA with HMGB genes in LUAD or LUSC under the “Pan-Cancer” section.

### OncomiR

OncomiR is an online resource for exploring microRNA dysregulation in cancer. We retrieved a list of microRNAs closely associated with patient survival in LUAD or LUSC, with a log-rank *p*-value of *p* < 0.05 (univariate Cox analysis).

## Results

### Expression profiles of HMGB family members in NSCLC patients

**FIGURE 1:**
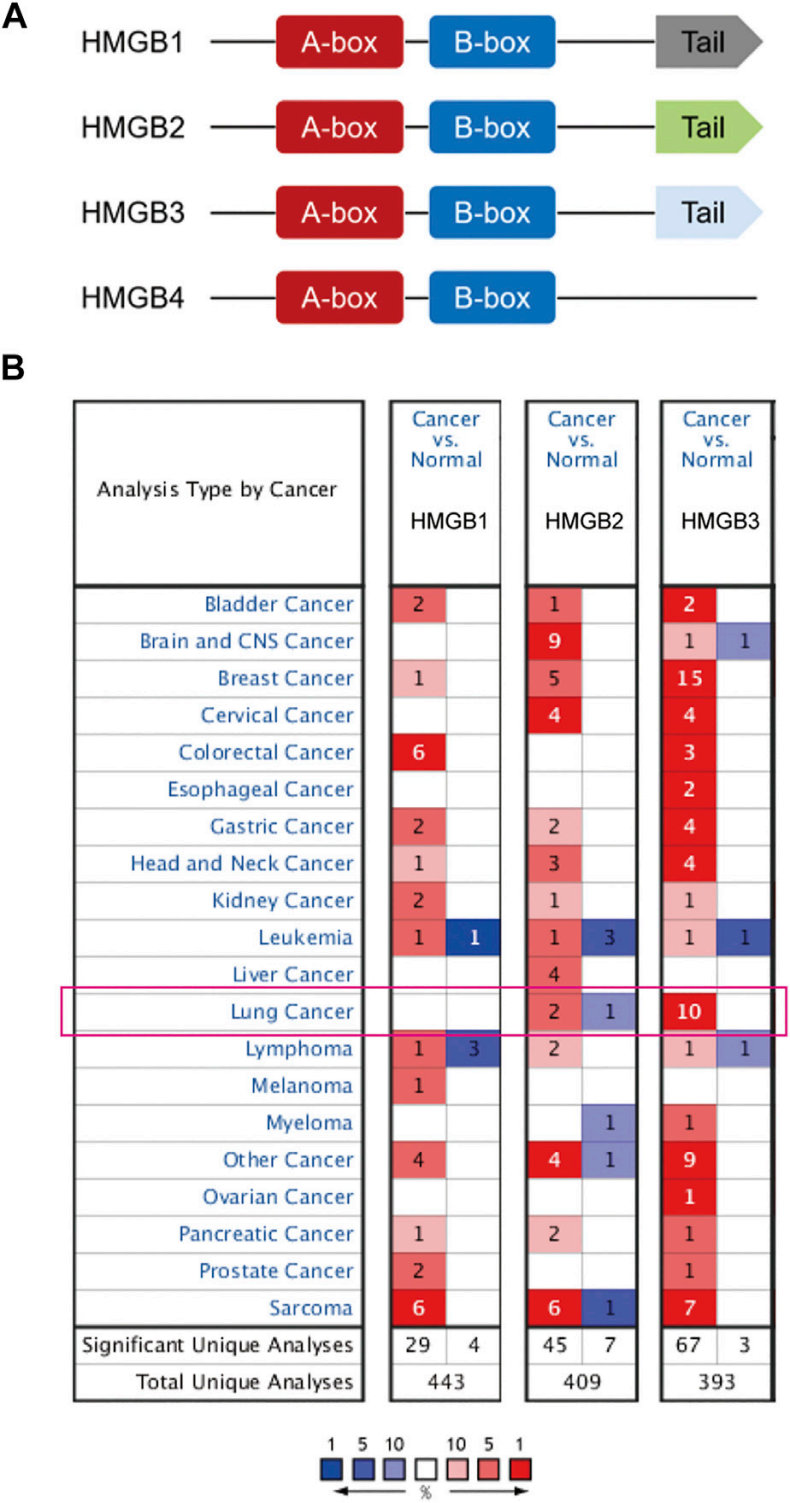
**(A)** Schematic diagram of HMGB protein family members. HMGB1/2/3 have box-A/B and an acidic C-terminal tail, while HMGB4 has no acidic C-terminal tail. **(B)** The number of ONCOMINE analyses in which HMGB family members are significantly differentially expressed between cancer and normal tissue. Student’s t-test was used to compared the transcriptional differences. Cutoffs for *p*-value and fold change were as following: *p*-value: 0.05, fold change: 2, gene rank: 10%, data type: mRNA. Red: up-regulation; Blue: down-regulation.

**FIGURE 2:**
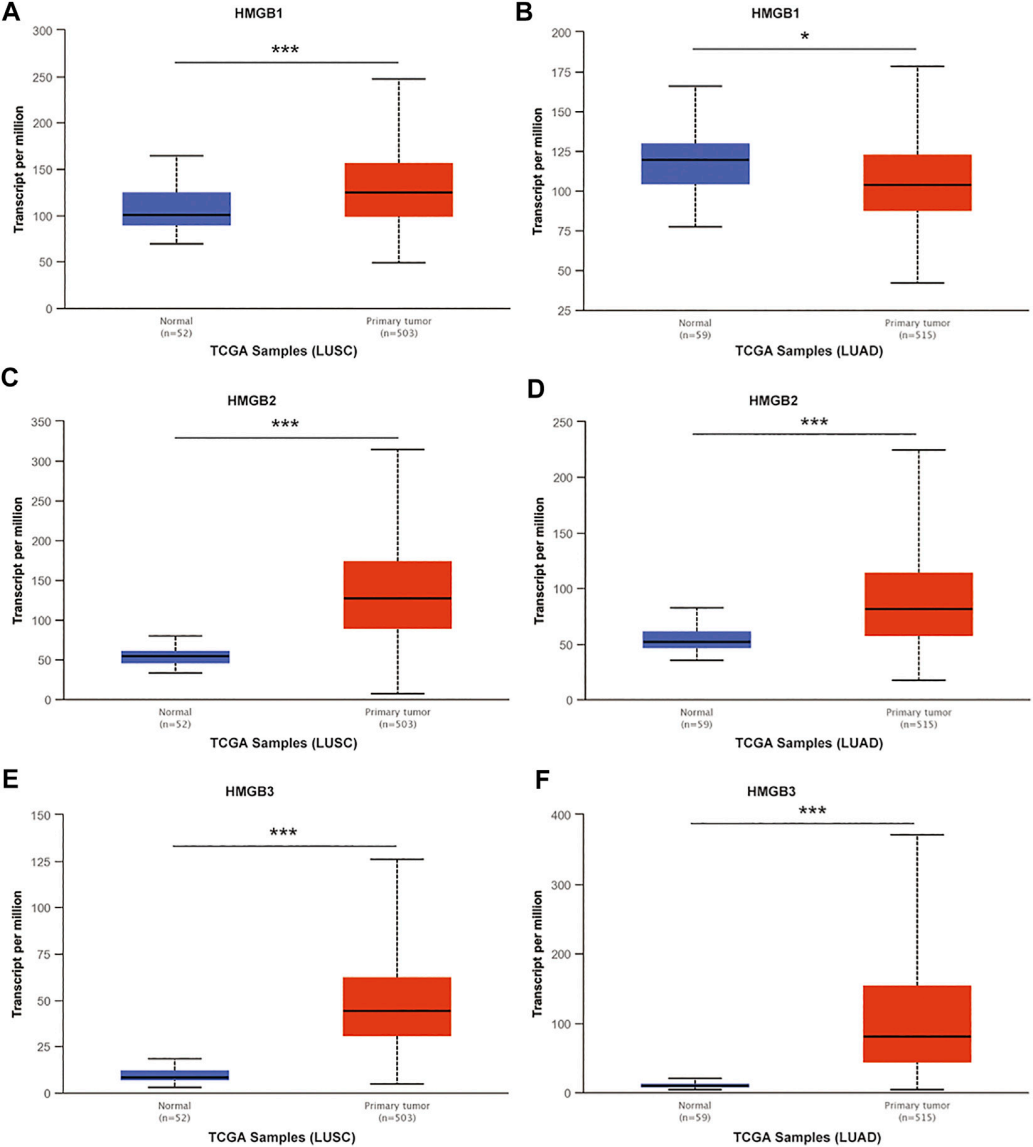
The mRNA expression of HMGB family members in NSCLC tissues and normal lung tissues (UALCAN). mRNA expressions of HMGB1/2/3 were elevated in LUSC tissue compared to normal samples **(A,C,E)**. HMGB2/3 was higher in LUAD tissue than in normal lung tissues **(D,F)**, while the expression level of HMGB1 was lower in the LUAD tissue than in normal lung tissue **(B)**. ***p < 0.001, *p < 0.05.

To understand the relationship between HMGB family members and NSCLC, we first compared the definite genomic sites (Table 1) and protein structures of all members (Figure 1A). Details of the differences in HMGB gene sequences are shown in Supplementary Material S1. HMGB1/2/3 are highly similar in structure, containing two stable binding peptide motifs (A-box and B-box) and an acidic tail, while HMGB4 has no acidic tail. Subsequently, we focused on HMGB1/2/3 in patients with NSCLC. ONCOMINE and UALCAN were utilized to compare expression levels of HMGB family members in cancer vs. normal tissue samples. We identified dysregulated levels of HMGB gene transcripts in 20 different types of cancers, in comparison with normal samples, utilizing the ONCOMINE database (Figure 1B). The differences in HMGB gene expression in cancer and normal tissues indicated a potential role for HMGBs in tumorigenesis and cancer progression. In lung cancer samples, the mRNA expression levels of HMGB2 were slightly elevated, and HMGB3 was significantly up-regulated in cancer tissue vs. normal tissues in multiple datasets (Figure 1B). For example, in the Hou Lung dataset (Hou et al., 2010), a 5.974-fold increase in HMGB3 mRNA expression was identified in lung adenocarcinoma (LUAD) tissues vs. normal lung tissue (*p* = 3.38E-15; Table 2). The mRNA expression of HMGB2 in lung squamous cell carcinoma (LUSC) was higher by a fold change of 2.698 (*p* = 0.003) compared to normal lung tissue (Table 2; Bhattacharjee, et al., 2001). Furthermore, we compared the mRNA expression of HMGB family members between 515 LUAD and 59 normal tissues, and 503 LUSC and 52 normal tissues, using UALCAN. HMGB2/3 levels were significantly elevated in both LUAD and LUSC tumor tissues when compared to corresponding normal tissues (Figure 2); data from the Human Protein Atlas followed a similar pattern (Supplementary Figure S1). Of note, although HMGB1 was higher in LUSC tissue than in normal lung tissue, it was lower in LUAD tissue than in lung tissue; this was not the case with HMGB2/3. Based on these analyses, we concluded that HMGB family member genes were over-expressed in patients with NSCLC, with the exception of HMGB1 in LUAD.

**TABLE 1:** The chromosomal locations of HMGB1, 2, 3, and 4.

HMGB family	HMGB1	HMGB2	HMGB3	HMGB4
Chromosomal location	13q12.3	4q34.1	Xq28	1p35.1

**TABLE 2:** ONCOMINE analyses indicating significant differences in mRNA expression of HMGB family members between lung cancer and normal lung tissue.

	Type of lung cancer vs. normal	Fold change	*p*-value	t-test	References
HMGB2	Squamous Cell Lung Carcinoma vs. Normal	2.698	0.003	2.895	Bhattacharjee et al. (2001)
HMGB3	Lung Adenocarcinoma vs. Normal	4.651	2.08e-25	15.357	Landi et al. (2008)
	Lung Adenocarcinoma vs. Normal	3.721	5.19e-10	8.603	Su et al. (2007)
	Lung Adenocarcinoma vs. Normal	4.543	4.59e-21	16.016	Okayama et al. (2012)
	Lung Adenocarcinoma vs. Normal	5.974	3.38e-15	10.682	Hou et al. (2010)
	Squamous Cell Lung Carcinoma vs. Normal	4.877	6.71e-17	13.624	Hou et al. (2010)
	Large Cell Lung Carcinoma vs. Normal	6.378	1.91e-7	7.445	Hou et al. (2010)
	Lung Adenocarcinoma vs. Normal	4.203	5.18e-4	3.819	Bhattacharjee et al. (2001)
	Squamous Cell Lung Carcinoma vs. Normal	3.672	3.31e-4	7.858	Wachi et al. (2005)
	Lung Adenocarcinoma vs. Normal	4.367	5.58e-16	10.385	Selamat et al. (2012)

### Correlation between mRNA expression of HMGB family members and NSCLC tumor stage

**FIGURE 3:**
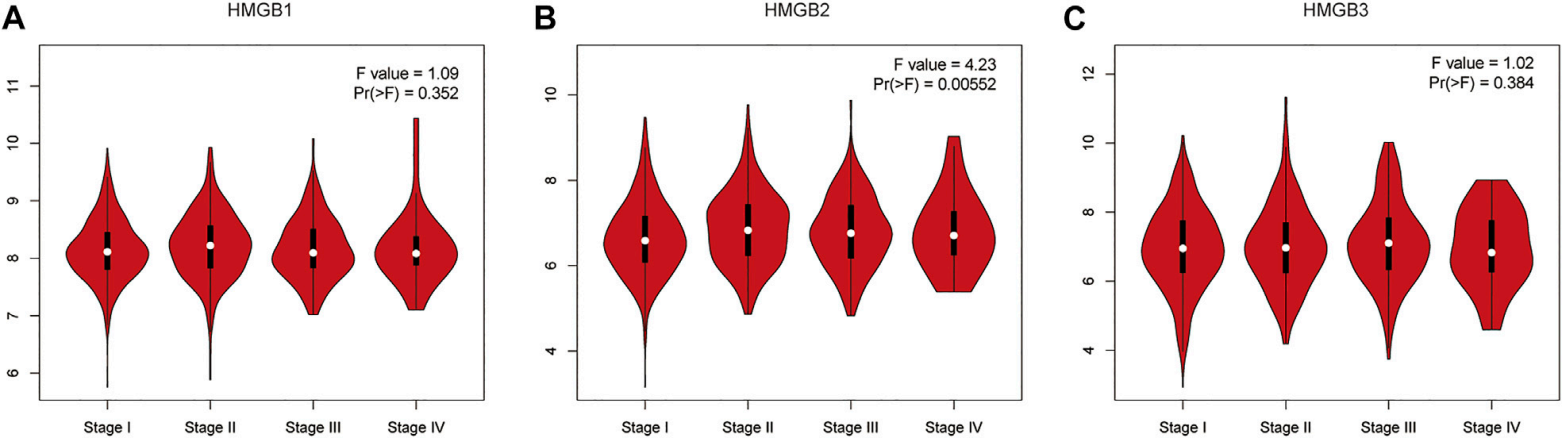
The relationship between mRNA expression of HMGB family members and tumor stage in patients with NSCLC (GEPIA). The mRNA expression of HMGB2 was significantly related to tumor stage **(B)**, while the mRNA expression of HMGB1/3 was not associated with tumor stage **(A,C)**.

GEPIA was used analyze the relationships between mRNA expression of different HMGB family members and NSCLC tumor stage. The mRNA expression of HMGB2 was significantly related to cancer stages (Figure 3), while the mRNA expression of HMGB1 and HMGB3 were not significantly associated with NSCLC tumor stage. The molecular reason for these differences in association with tumor stage of HMGB2 vs. HMGB1/3 is not yet understood.

### Prognostic features of HMGB family members in lung cancer outcomes

**FIGURE 4:**
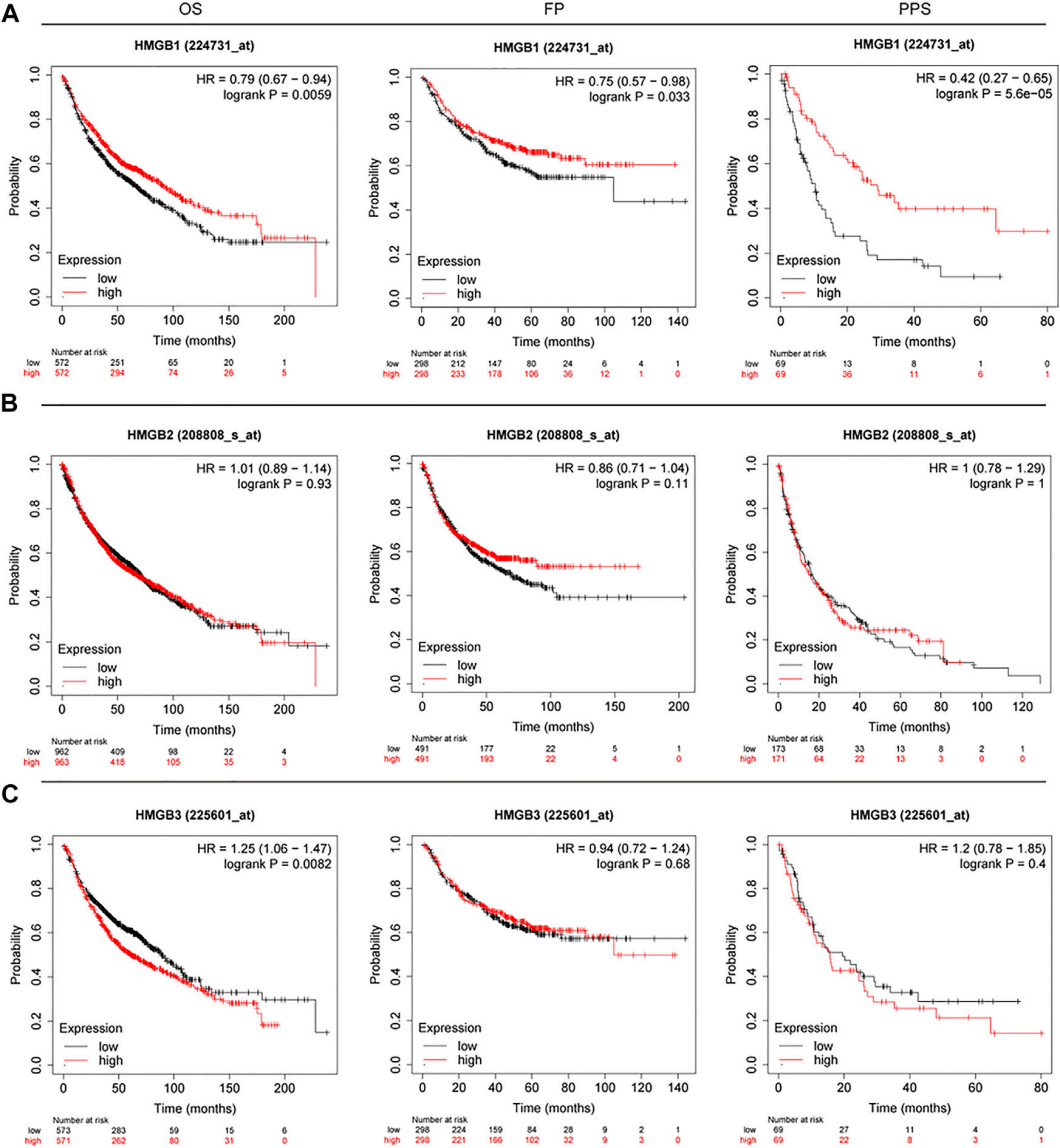
OS, FP, and PPS survival curves of patients with lung cancer subset by expression of HMGB family members (Kaplan–Meier Plotter). The threshold of significance was a *p*-value of < 0.05. The association between prognostic value and **(A)** HMGB1, **(B)** HMGB2, **(C)** HMGB3. Red: high expression; Black: low expression. Left: OS; middle: FP; right: PPS.

To understand the prognostic features of HMGB family in patients with lung cancer, the Kaplan–Meier Plotter database (2015 version) was used to evaluate overall survival (OS), first-progression survival (FP), and post-progression survival (PPS). In each cohort, patients were divided into low and high-risk group based on the median cutoff value (see Figure 4; Table 3). Higher expression of HMGB1 mRNA were associated with improved OS (*p* = 0.0059), FP (*p* = 0.0331), and PPS (*p* = 5.6E-5). In contrast, higher HMGB3 mRNA expression was significantly associated with poorer OS (*p* = 0.0082). There was no significant correlation found between HMGB3 expression and FP or PPS in lung cancer. Similarly, there was no significant association observed between HMGB2 expression and OS, FP, or PPS in lung cancer.

**TABLE 3:** The prognostic values of HMGB family members in lung cancer patients (Kaplan–Meier plotter).

HMGB member	OS				FP				PPS			
	Cases	HR	95%Cl	*p*-value	Cases	HR	95%Cl	*p*-value	Cases	HR	95%Cl	*p*-value
HMGB1(224731_at)	1927	0.79	0.67–0.94	0.0059	982	0.75	0.57–0.98	0.0331	344	0.42	0.27–0.65	5.6e-5
HMGB2(208808_s_at)	1927	1.01	0.89–1.14	0.9314	982	0.86	0.71–1.04	0.1144	344	1	0.78–1.29	0.9965
HMGB3(225601_at)	1927	1.25	1.06–1.47	0.0082	982	0.94	0.72–1.24	0.6783	344	1.2	0.78–1.85	0.3992

### Protein and genetic interactions, pathways, and co-expression of HMGB family genes

**FIGURE 5:**
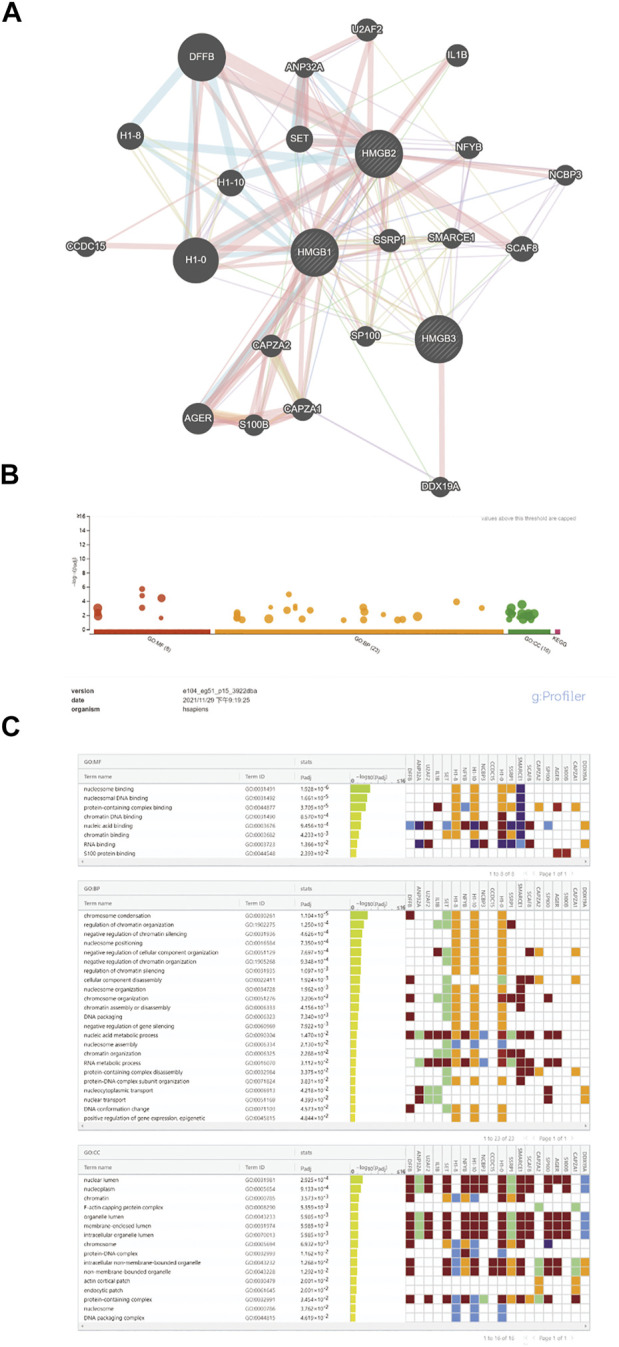
Functional involvement of HMGB family members. GeneMANIA was used to predict the function of HMGB family and GO and KEGG analysis were performed to find related genes and pathways. **(A)** Schematic diagram of protein and genetic interactions, pathways, and co-expression of HMGB family members. **(B,C)** GO and KEGG analysis details.

After analyzing the prognostic value of HMGB family members in NSCLC patients, we used Gene MANIA to analyze protein and genetic interactions, pathways, and co-expression HMGB family members. HMGB family members were found to closely interact with histones, including H1-0, H1-8 and H1-10 (Figure 5A, Supplementary Material S2). In addition, we found that DFFB was the most frequent interacting partner with HMGB family members in this interactive network. DFFB is known to play a vital role in apoptosis (Takeshita et al., 2012; Han et al., 2020). Next, GO and KEGG analysis were performed in g:Profiler to analyze the potential role of HMGB family members and 20 related genes (Figures 5B,C). Chromosome condensation (GO: 0030261) and regulation of chromatin organization (GO: 001902275) were the top two GO Biological Pathways. The most enriched GO Cellular Components of the 16 genes were the nuclear lumen (GO: 003198) and nucleoplasm (GO: 0005654). The three most significantly enriched GO Molecular Functions were nucleosome binding (GO: 0031491), nucleosomal DNA binding (GO: 0031492), and protein-containing complex binding (GO: 0044877). In the KEGG analysis, no significant enrichment was found in relation to the function of HMGB family members in NSCLC. These results suggest that HMGB family members may participate in chromosome condensation, regulation of chromatin organization, and nucleosome binding in NSCLC.

### HMGB expression correlates with cytotoxic immune cell infiltration in NSCLC

**FIGURE 6:**
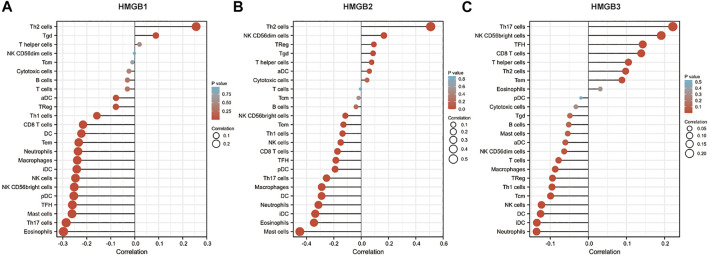
Correlation of HMGB family members gene expression with levels of immune infiltration in NSCLC. Tcm, central memory T cells; Tem, effector memory T cells; TFH, T follicular helper cells; Tgd, gamma delta T cells; Treg, T regulatory cells.

Immune infiltration in the tumor microenvironment is closely related to clinical outcomes in cancers, including NSCLC (Fridman et al., 2011; Chen et al., 2019; Guo et al., 2022). We used the ssGSEA algorithm on the XIANTAO platform (www.xiantao.love) to assess the correlation between expression of HMGB genes and immune infiltration profiles of 24 types of infiltrating immune cells in NSCLC samples. Spearman correlation analysis identified a significant positive correlation between HMGB1 expression levels and levels of infiltrating Th2 cells (R = 0.256, *p* < 0.001), and a significant negative correlation between HMGB1 levels and infiltrating eosinophils (R = −0.298, *p* < 0.001), Th17 cells (R = −0.286, *p* < 0.001), and mast cells (R = −0.262, *p* < 0.001) (Figure 6A; Table 4). Similarly, HMGB2 expression was positively correlation with infiltrating Th2 cells (R = 0.509, *p* < 0.001) and was negatively correlated with levels of infiltrating mast cells (R = −0.449, *p* < 0.001), eosinophils (R = −0.346, *p* < 0.001), and iDC (R = −0.336, *p* < 0.001) (Figure 6B; Table 4). In addition, HMGB3 expression was positively associated with infiltrating Th17 cells (R = 0.222, *p* < 0.001) and had a weak negative correlation with infiltrating neutrophils (R = −0.136, *p* < 0.001) and iDC (R = −0.135, *p* < 0.001; Figure 6C, Table 4). These findings suggest that HMGB genes play a role in mediating infiltration of specific types of immune cells in NSCLC, especially Th2 cells, Th17 cells, and mast cells.

**TABLE 4:** The Spearman correlation R value and *p*-value of HMGB family members with infiltrating immune cells in NSCLC samples.

Immune cell	Spearman correlation R			*p-* value		
	HMGB1	HMGB2	HMGB3	HMGB1	HMGB2	HMGB3
aDC	−0.078	0.060	−0.061	0.011	0.054	0.051
B cells	−0.031	−0.038	−0.052	0.321	0.219	0.097
CD8 T cells	−0.216	−0.174	0.139	<0.001	<0.001	<0.001
Cytotoxic cells	−0.024	−.043	−0.033	0.436	0.168	0.291
DC	−0.223	−0.290	−0.126	<0.001	<0.001	<0.001
Eosinophils	−0.298	−0.346	0.031	<0.001	<0.001	0.317
iDC	−0.242	−0.336	−0.135	<0.001	<0.001	<0.001
Macrophages	−0.240	−0.289	−0.087	<0.001	<0.001	0.005
Mast cells	−0.262	−0.449	−0.054	<0.001	<0.001	0.081
Neutrophils	−0.237	−0.313	−0.136	<0.001	<0.001	<0.001
NK CD56bright cells	−0.252	−0.116	0.191	<0.001	<0.001	<0.001
NK CD56dim cells	−0.002	0.166	−0.064	0.959	<0.001	0.041
NK cells	−0.247	−0.150	−0.123	<0.001	<0.001	<0.001
pDC	−0.254	−0.191	−0.020	<0.001	<0.001	0.528
T cells	−0.031	−0.005	−0.078	0.318	0.870	0.012
T helper cells	0.020	0.076	0.105	0.530	0.014	<0.001
Tcm	−0.010	−0.019	−0.099	0.747	0.531	0.001
Tem	−0.234	−0.130	0.088	<0.001	<0.001	0.005
TFH	−0.260	−0.185	0.143	<0.001	<0.001	<0.001
Tgd	0.088	0.087	−0.049	0.005	0.005	0.118
Th1 cells	−0.158	−0.136	−0.095	<0.001	<0.001	0.002
Th17 cells	−0.286	−0.254	0.222	<0.001	<0.001	<0.001
Th2 cells	0.256	0.509	0.098	<0.001	<0.001	0.002
TReg	−0.079	0.093	−0.094	0.011	0.003	0.002

### microRNA–HMGB interactions in the prognosis of NSCLC

**FIGURE 7:**
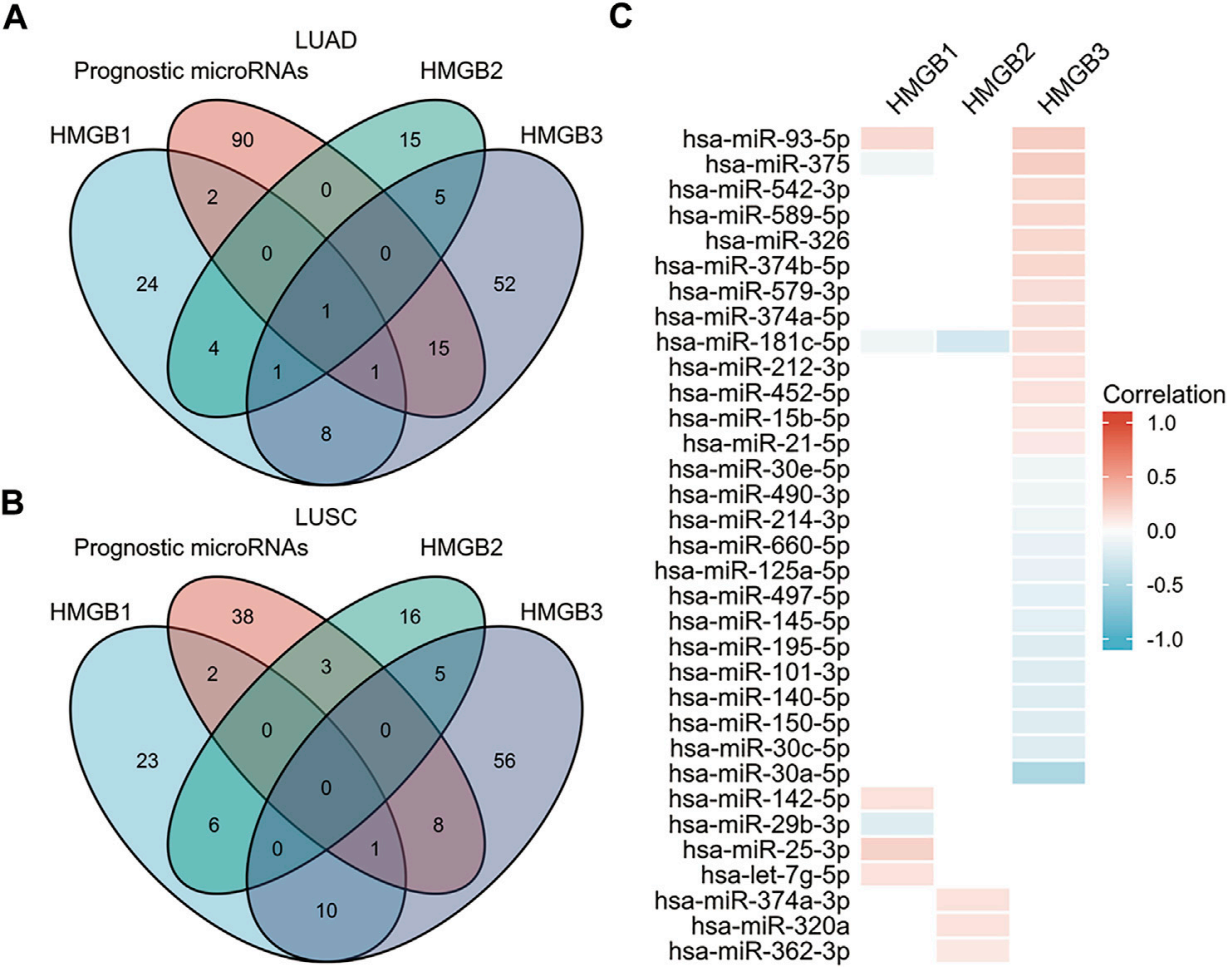
Identification of microRNAs of prognostic value in NSCLC and their predicted interactions with HMGB family member genes. **(A)** Venn diagram depicting the intersection of HMGB-targeting microRNAs in LUAD from the starBase and OncomiR databases. **(B)** Venn diagram depicting the intersection of HMGB-targeting microRNAs in LUSC from the starBase and OncomiR databases. **(C)** Correlation of HMGB family members expression and expression of microRNAs that have prognostic value in NSCLC.

MicroRNAs play important roles in cancer initiation and development (Lages et al., 2012; Takasaki, 2015). First, we explored microRNA–HMGBs interaction maps in NSCLC samples from Argonaute CLIP-Seq and degradome-Seq data by using the starBase database (Supplementary Material S3). Then, we searched significant microRNAs in survival in LUAD and LUSC on the database of OncomiR (Supplementary Material S4). Finally, by taking the intersection of the above results (Figure 7), 7, 4, and 26 microRNAs, respectively, were found to potentially target HMGB1, HMGB2, and HMGB3 in NSCLC. We evaluated the correlation of HMGBs expression and microRNAs in NSCLC (Figure 7C, Supplementary Figure S2). We found that hsa-miR-25-3p, hsa-miR-374a-3p, and hsa-miR-93-5p were significantly positively correlated with HMGB1, HMGB2, and HMGB3, respectively. Furthermore, hsa-miR-30a-5p, which has been linked to favorable OS (Wei et al., 2016; Zhang et al., 2017), was predicted to negatively regulate HMGB3 expression, which is consistent with our aforementioned results.

## Discussion and conclusion

HMGB proteins are chromatin-associated architectural proteins and are fundamental for efficient genome organization and execution of DNA replication, DNA repair, and transcription (Niu et al., 2020; Lou et al., 2021; Wen et al., 2021). The HMGB family consists of four members, including HMGB1-4. HMGB family members are widely involved in the initiation and development of a variety of cancers, including colorectal cancer, hepatocellular carcinoma, and gastric cancer (Fang et al., 2020; Niu et al., 2020; Wen et al., 2021). In this study, we provide a comprehensive analysis of differential expression, immune infiltration and evaluation of the prognostic significance of HMGB1/2/3 in NSCLC.

HMGB1 is a non-histone nuclear protein and is present in almost all eukaryotic cells. HMGB1 works as a nuclear protein and can also be released or secreted into the extracellular interstices to perform a range of biological functions. HMGB1 plays a significant role in many diseases, including autoimmune diseases, infectious diseases, inflammatory diseases, and cancer (Kang et al., 2013; Tripathi et al., 2019). In cancer, HMGB1 plays dual roles as both a pro-cancer and an anti-cancer factor by regulating multiple cellular processes, including proliferation, angiogenesis, and invasion (Wu et al., 2018; Ren et al., 2021). HMGB1 has been shown to enhance tumor growth, angiogenesis, and metastasis of a variety of cancers, including breast cancer, prostate cancer, colorectal cancer, and lung cancer (Zhang J et al., 2014; Zhang L et al., 2014; Meng et al., 2014; Süren et al., 2014; Liu et al., 2015; Sun et al., 2015; Ren et al., 2021). In contrast, Kang et al. (2013) and Luan et al. (2017) reported that HMGB1 acts as a tumor suppressor in pancreatic cancer and endometrial carcinoma (Tang et al., 2010; Zuo et al., 2014). In terms of lung cancer, HMGB1 levels are higher in cancer tissue and in patient serum than in non-cancer tissue, and are correlated with TNM stages and tumor size in NSCLC (Liu et al., 2017; Wang M et al., 2019; Wang et al., 2020). In contrast, some studies have shown that HMGB1 expression is lower in NSCLC tissue compared with matched normal lung tissue, especially in patient with stage III-IV disease (Shen et al., 2009; Wu et al., 2018). In our present study, we found over-expression of HMGB1 only in LUSC vs. normal lung, but under-expressed in LUAD tissue vs. normal lung. These data demonstrate that the upregulation or downregulation of HMGB1 in NSCLC still needs to be further explored, and may be related to pathological tumor type. Previous studies have shown that HMGB1 can promote tumor growth and metastasis through the TAK1-NF-*κ*B or RAGE/JNK/NF-*κ*B pathways (van Beijnum et al., 2013; He et al., 2016; Nguyen et al., 2016; Nguyen et al., 2017; Wu et al., 2018). However, in this study we found that higher mRNA expression of HMGB1 associated with improved OS, FP, and PPS in lung cancer; this is similar to the findings of Dumitriu et al. (2007) and Suzuki et al. (2012). In terms of mechanism, we explored immune infiltration levels correlated with expression of HMGB genes in NSCLC. We identified a strong positive relationship between HMGB1 expression and levels of infiltrating Th2 cells, and a significantly negative correlation between HMGB1 expression and levels of infiltrating eosinophils, Th17 cells, and mast cells. Moreover, we found that HMGB1 was significantly positively correlated with expression of hsa-miR-25-3p, hsa-miR-142-5p, hsa-miR-93-5p, and hsa-let-7g-5p, and was negatively correlated with hsa-miR-375, hsa-miR-181c-5p and hsa-miR-29b-3p. These microRNAs all act as tumor suppressors in NSCLC, according to the OncomiR database. These results suggest that immune infiltration and microRNAs might serve as mechanisms by which HMGB1 expression influences the NSCLC prognosis. These discoveries raise the possibility that HMGB1 may play a significant role in the tumorigenesis of NSCLC, and may be able to be developed as a novel therapeutic target.

High-mobility group box 2 (HMGB2) is generally considered to be an oncogene (Niu et al., 2020; Lou et al., 2021). HMGB2 can promote the proliferation of different cancer cells, including prostate cancer, breast cancer, HCC, and cervical cancer, through the AKT and Wnt pathways (Niu et al., 2020; Wen et al., 2021). In addition, HMGB2 can also promote tumor invasion and migration by regulating noncoding RNAs and CENPU (Li H et al., 2018; Han et al., 2019; Li et al., 2019; Niu et al., 2020). In the context of NSCLC, Lou et al. (2021) reported that HMGB2 protein expression was higher in tumor tissue compared with adjacent tissue, which is consistent with our results in LUAD and LUSC. Lou et al. (2021) also revealed that high tumor mRNA expression of HMGB2 was associated with shorter disease-free survival (DFS) and shorter OS. However, Niu et al. (2020) showed that overexpression of HMGB2 was prognostic of longer OS in cancers including BRCA, CESC, LUSC, READ, STAD, and THCA. Unexpectedly, in our study, HMGB2 expression levels did not correlate with OS, FP, or PPS of lung cancer patients. We found that HMGB2 expression showed a strong positive correlation with levels of infiltrating Th2 cells and negative correlations with the levels of infiltrating mast cells, eosinophils, and iDC. Th2 cells are considered to have tumor-promoting activities, and Th17 cells promote tumor growth by inducing angiogenesis (via IL-17) and by exerting immunosuppressive functions. Mast cells and eosinophils play both anti- and pro-tumorigenic activities, depending on the cellular milieu of the tumor. In addition, we found that HMGB2 expression was significantly positively correlated with expression of hsa-miR-374a-3p, hsa-miR-320a and hsa-miR-362-3p, but negatively correlated with hsa-miR-181c-5p. And these microRNAs are all involved in the prognosis of NSCLC patients. Together with these reports, the role of HMGB2 in lung cancer patients requires the further exploration.

HMGB3 is mainly expressed in embryonic and bone marrow hematopoietic stem cells, and not expressed or is expressed at low levels in normal adult tissues (Wen et al., 2021). HMGB3 acts as an oncogene in the development of human cancers, including liver cancer, breast cancer, gastric cancer, leukemia, and colon cancer (Ren et al., 2020; Wen et al., 2021). HMGB3 can regulate the cell cycle to induce tumor development, promote the proliferation and invasion of cancer cells by regulating the Wnt/*β*-catenin, MAPK, Akt, HIF-1*α*, and other signaling pathways, and enhances the activity of tumor stem cell genes to promote the proliferation of cancer cells (Gu et al., 2019a; Gu et al., 2019b; Ren et al., 2020; Wen et al., 2021). However, there are few studies on HMGB3 in lung cancer. HMGB3 was reported to be overexpressed in NSCLC tumor tissue compared to adjacent or normal tissues (Wang J et al., 2019; Li et al., 2020; Ren et al., 2020). Similarly, in our investigation, we found higher mRNA expression of HMGB3 in NSCLC tissues, but mRNA expression of HMGB3 was not correlated with tumor stage of NSCLC. High mRNA expression of HMGB3 was positively related with tumor growth, proliferation, and invasion of NSCLC, suggesting that it may associate with poorer prognosis (Gu et al., 2019a; Gu et al., 2019b; Wang J et al., 2019; Li et al., 2020; Ren et al., 2020). We found that higher levels of HMGB3 were associated with worse OS in NSCLC patients. This might be related to the infiltration of Th17 cells and lower expression of has-miR-30a-5p, as suggested by our results. Based on these data, we suggest that HMGB3 may act as an oncogene in NSCLC and may be prognostic of poor outcomes for patients with NSCLC.

In summary, this study elucidated the prognostic significance, gene interactions, immune infiltration, and co-expression relationships related to the mRNA expression of HMGB family members in NSCLC. Our research revealed that HMGB1 may act as a tumor suppressor gene in NSCLC, which is in contrast with the results of previous studies. We found no significant association between HMGB2 expression and prognostic characteristics of lung cancer. We found that higher expression of HMGB3 associated with poorer outcomes, suggesting that HMGB3 may act as an oncogene in NSCLC. Furthermore, HMGB genes may affect patient survival by altering the immune microenvironment or by interacting with microRNAs. Together, our findings suggest that HMGB family members may act as novel potential therapeutic targets for NSCLC, but further research is needed to better understand the roles of HMGB family members and their potential clinical utility in NSCLC.

## Data Availability

The datasets presented in this study can be found in online repositories. The names of the repository/repositories and accession number(s) can be found in the article/Supplementary Material.
