# Supplementary material for: Systematic analysis of expression profiles of HMGB family members for prognostic application in non-small cell lung cancer
Source: Front Mol Biosci. 2022 Jul 18;9:844618. doi: 10.3389/fmolb.2022.844618 (PMC9340210; doi:10.3389/fmolb.2022.844618)
Supplement: Supplementary file 2 [file DataSheet4.PDF]

LUAD

miRNA	Log Rank P-value	Log Rank FDR	Z-score	Upregulated in:
hsa-miR-1304-5p	1.82E-04	1.78E-01	4.241	Deceased
hsa-miR-29b-2-5p	2.64E-04	9.92E-02	3.646	Living
hsa-miR-148a-3p	2.67E-04	3.22E-01	3.693	Living
hsa-miR-29c-3p	4.56E-04	7.28E-02	3.523	Living
hsa-miR-31-3p	4.71E-04	5.20E-02	3.629	Deceased
hsa-miR-212-3p	5.37E-04	6.87E-01	3.454	Deceased
hsa-miR-101-3p	5.87E-04	1.66E-02	3.451	Living
hsa-miR-548b-3p	6.34E-04	5.48E-01	3.308	Living
hsa-miR-29c-5p	6.45E-04	1.36E-01	3.396	Living
hsa-miR-3187-3p	7.19E-04	2.93E-01	4.226	Deceased
hsa-miR-181c-5p	9.43E-04	1.45E-02	3.336	Living
hsa-miR-1468-5p	1.16E-03	3.08E-02	3.189	Living
hsa-miR-3926	1.41E-03	3.14E-01	2.544	Living
hsa-miR-30e-5p	1.45E-03	3.02E-01	3.172	Living
hsa-miR-548v	1.49E-03	5.19E-01	3.003	Living
hsa-miR-31-5p	1.58E-03	5.29E-03	3.24	Deceased
hsa-miR-132-3p	1.67E-03	8.91E-01	3.118	Deceased
hsa-miR-299-5p	1.95E-03	4.34E-02	3.276	Deceased
hsa-miR-1910-5p	2.06E-03	9.60E-01	3.755	Deceased
hsa-miR-582-5p	2.21E-03	1.99E-01	3.116	Deceased
hsa-miR-374b-3p	2.55E-03	7.70E-01	2.893	Living
hsa-miR-3126-3p	2.58E-03	7.46E-01	4.426	Deceased
hsa-miR-99a-5p	2.91E-03	1.29E-01	3.025	Living
hsa-miR-10b-3p	3.47E-03	5.35E-02	2.972	Deceased
hsa-miR-3653	4.13E-03	4.03E-01	2.841	Living
hsa-miR-2116-3p	4.57E-03	5.92E-01	3.137	Deceased
hsa-miR-195-3p	4.97E-03	7.16E-02	2.809	Living
hsa-miR-582-3p	5.44E-03	1.16E-01	2.827	Deceased
hsa-miR-30b-5p	5.54E-03	2.67E-01	2.766	Living
hsa-miR-299-3p	5.59E-03	3.02E-03	3.216	Deceased
hsa-miR-30d-3p	6.89E-03	2.47E-01	2.718	Living
hsa-miR-1305	7.43E-03	5.35E-02	3.309	Deceased
hsa-miR-150-5p	7.52E-03	9.56E-02	2.693	Living
hsa-miR-148a-5p	8.31E-03	4.25E-01	2.689	Living
hsa-miR-375	9.19E-03	1.20E-01	2.632	Living
hsa-miR-133b	9.99E-03	6.57E-02	2.381	Living
hsa-miR-497-5p	1.02E-02	2.27E-02	2.569	Living
hsa-miR-584-5p	1.04E-02	5.71E-01	2.648	Deceased
hsa-miR-99a-3p	1.07E-02	2.15E-01	2.454	Living
hsa-miR-200a-3p	1.10E-02	9.75E-02	2.61	Living
hsa-miR-181c-3p	1.14E-02	2.57E-01	2.536	Living
hsa-miR-490-3p	1.23E-02	2.04E-01	1.849	Living
hsa-miR-30c-5p	1.28E-02	4.86E-01	2.492	Living
hsa-miR-199b-5p	1.30E-02	4.80E-02	2.506	Living
hsa-miR-328-3p	1.31E-02	2.04E-01	2.478	Living
hsa-miR-466	1.32E-02	3.90E-01	0	Living
hsa-miR-550a-5p	1.35E-02	3.06E-02	2.48	Deceased
hsa-let-7g-3p	1.38E-02	2.79E-01	2.49	Living
hsa-miR-1915-3p	1.39E-02	9.89E-01	3.799	Deceased
hsa-miR-142-3p	1.43E-02	3.44E-01	2.456	Living
hsa-miR-142-5p	1.52E-02	4.03E-01	2.427	Living
hsa-miR-3607-3p	1.52E-02	8.33E-01	2.412	Living
hsa-miR-335-5p	1.56E-02	7.60E-01	2.423	Living
hsa-miR-3138	1.69E-02	8.63E-01	3.699	Deceased
hsa-miR-29b-3p	1.73E-02	2.93E-01	2.41	Living
hsa-miR-200a-5p	1.78E-02	3.10E-01	2.433	Living

hsa-miR-429	1.82E-02	4.23E-01	2.41	Deceased
hsa-miR-3189-3p	1.85E-02	2.91E-01	2.121	Living
hsa-miR-550a-3p	1.89E-02	6.87E-01	2.383	Deceased
hsa-miR-3682-3p	1.95E-02	1.68E-01	2.46	Deceased
hsa-miR-24-1-5p	2.03E-02	4.86E-01	2.304	Living
hsa-miR-1915-5p	2.05E-02	9.75E-01	3.547	Deceased
hsa-miR-3145-3p	2.06E-02	1.11E-01	3.548	Deceased
hsa-let-7c-5p	2.08E-02	6.53E-01	2.33	Living
hsa-miR-30d-5p	2.15E-02	2.86E-01	2.289	Living
hsa-miR-3125	2.25E-02	1.31E-01	3.48	Deceased
hsa-miR-3154	2.25E-02	1.31E-01	3.48	Deceased
hsa-miR-1306-3p	2.26E-02	2.27E-01	2.379	Deceased
hsa-miR-29a-5p	2.27E-02	4.12E-01	2.289	Living
hsa-miR-125a-5p	2.35E-02	4.86E-01	2.267	Living
hsa-miR-30a-5p	2.35E-02	6.74E-01	2.267	Living
hsa-miR-1197	2.41E-02	2.67E-01	3.413	Deceased
hsa-miR-1243	2.45E-02	1.59E-01	3.415	Deceased
hsa-miR-1976	2.46E-02	7.68E-02	2.267	Living
hsa-miR-545-3p	2.51E-02	5.41E-01	0	Living
hsa-miR-665	2.61E-02	2.12E-01	2.59	Deceased
hsa-miR-145-3p	2.68E-02	7.25E-01	2.252	Living
hsa-miR-873-5p	2.76E-02	7.88E-01	2.431	Deceased
hsa-miR-3920	3.03E-02	2.71E-01	2.429	Deceased
hsa-miR-196b-5p	3.06E-02	5.39E-02	2.193	Deceased
hsa-miR-501-3p	3.15E-02	7.69E-01	2.154	Living
hsa-miR-30e-3p	3.17E-02	9.25E-01	2.149	Living
hsa-miR-664a-5p	3.21E-02	6.95E-01	2.14	Living
hsa-miR-491-5p	3.22E-02	3.73E-01	2.102	Living
hsa-miR-551b-3p	3.23E-02	8.41E-01	2.086	Living
hsa-miR-21-5p	3.27E-02	8.24E-01	2.095	Deceased
hsa-miR-548f-3p	3.40E-02	1.11E-01	2.474	Deceased
hsa-miR-3943	3.42E-02	3.49E-01	3.045	Deceased
hsa-miR-500a-3p	3.46E-02	5.24E-01	2.107	Living
hsa-miR-195-5p	3.48E-02	7.78E-01	2.131	Living
hsa-let-7f-2-3p	3.51E-02	5.92E-01	1.98	Living
hsa-miR-3678-5p	3.62E-02	4.86E-01	1.594	Living
hsa-miR-326	3.63E-02	1.00E-01	2.098	Living
hsa-miR-188-3p	3.75E-02	2.45E-01	1.99	Living
hsa-miR-1254	3.76E-02	4.79E-01	2.186	Deceased
hsa-miR-1293	3.78E-02	6.21E-01	2.322	Deceased
hsa-miR-146b-3p	4.02E-02	1.01E-01	2.064	Living
hsa-miR-4286	4.10E-02	3.06E-01	1.14	Living
hsa-miR-660-5p	4.15E-02	9.56E-02	2.027	Living
hsa-miR-145-5p	4.18E-02	2.79E-01	2.035	Living
hsa-miR-450a-5p	4.18E-02	2.45E-01	2.022	Deceased
hsa-miR-942-5p	4.18E-02	5.90E-01	2.037	Deceased
hsa-miR-25-5p	4.40E-02	3.60E-01	2.307	Deceased
hsa-miR-140-5p	4.46E-02	6.92E-01	2.01	Living
hsa-miR-3065-3p	4.53E-02	1.46E-01	2	Living
hsa-miR-668-3p	4.54E-02	3.09E-01	2.377	Deceased
hsa-miR-16-1-3p	4.54E-02	5.51E-01	1.997	Living
hsa-miR-411-3p	4.73E-02	3.50E-02	2.112	Deceased
hsa-miR-26b-3p	4.73E-02	6.21E-01	2	Living

LUSC

miRNA	Log Rank P-value	Log Rank FDR	Z-score	Upregulated in:
hsa-miR-374a-3p	1.57E-04	0.992	3.805	Living
hsa-miR-187-3p	1.55E-03	0.665	3.153	Living
hsa-miR-3155a	2.76E-03	0.823	4.587	Deceased
hsa-miR-15b-5p	2.77E-03	0.823	2.988	Living
hsa-miR-542-3p	2.84E-03	0.749	2.915	Living
hsa-miR-615-5p	3.48E-03	0.823	4.46	Deceased
hsa-miR-25-3p	5.45E-03	0.69	2.775	Living
hsa-miR-214-3p	6.35E-03	0.74	2.733	Living
hsa-miR-500a-3p	7.60E-03	0.757	2.65	Living
hsa-miR-423-5p	7.66E-03	0.982	2.65	Living
hsa-miR-671-5p	1.05E-02	0.757	2.588	Living
hsa-miR-3174	1.11E-02	0.982	2.002	Living
hsa-miR-362-3p	1.12E-02	0.69	2.5	Living
hsa-miR-579-3p	1.26E-02	0.631	1.918	Living
hsa-miR-564	1.30E-02	0.68	3.885	Deceased
hsa-miR-16-2-3p	1.37E-02	0.823	2.476	Living
hsa-miR-3130-3p	1.38E-02	0.74	1.985	Living
hsa-miR-139-5p	1.55E-02	0.992	2.452	Deceased
hsa-miR-320a	1.63E-02	0.803	2.379	Living
hsa-miR-182-5p	1.71E-02	0.834	2.394	Living
hsa-miR-374a-5p	1.83E-02	0.914	2.375	Living
hsa-miR-28-3p	2.05E-02	0.982	2.298	Living
hsa-miR-708-5p	2.19E-02	0.982	2.311	Living
hsa-miR-589-5p	2.25E-02	0.957	2.238	Living
hsa-miR-3646	2.36E-02	0.938	3.438	Deceased
hsa-miR-500b-5p	2.48E-02	0.69	2.238	Living
hsa-miR-326	2.52E-02	0.757	2.256	Deceased
hsa-miR-500a-5p	2.66E-02	0.69	2.211	Living
hsa-miR-3912-3p	2.68E-02	0.757	2.427	Deceased
hsa-miR-450a-5p	2.71E-02	0.982	2.202	Living
hsa-miR-374b-5p	2.74E-02	0.868	2.195	Living
hsa-miR-1295a	2.75E-02	0.631	2.107	Living
hsa-miR-501-3p	2.89E-02	0.898	2.182	Living
hsa-miR-452-5p	2.94E-02	0.49	2.193	Living
hsa-miR-944	2.96E-02	0.631	2.247	Living
hsa-miR-502-3p	3.01E-02	0.803	2.179	Living
hsa-miR-3611	3.04E-02	0.97	0	Living
hsa-miR-337-5p	3.18E-02	0.823	1.543	Living
hsa-miR-93-5p	3.24E-02	0.69	2.142	Living
hsa-miR-1285-3p	3.25E-02	0.982	1.473	Living
hsa-miR-607	3.30E-02	0.69	1.17	Living
hsa-let-7g-5p	3.75E-02	0.957	2.095	Living
hsa-miR-369-5p	3.85E-02	0.906	2.03	Living
hsa-miR-3663-3p	3.86E-02	0.731	1.449	Living
hsa-miR-98-5p	3.92E-02	0.992	2.054	Deceased
hsa-miR-362-5p	3.93E-02	0.852	2.054	Living
hsa-let-7i-5p	3.98E-02	0.992	2.049	Living
hsa-miR-3605-3p	4.06E-02	0.69	2.074	Deceased
hsa-miR-193a-3p	4.08E-02	0.69	2.042	Deceased
hsa-miR-144-5p	4.21E-02	0.49	2.037	Living
hsa-miR-99b-3p	4.39E-02	0.757	2.007	Deceased
hsa-miR-338-5p	4.84E-02	0.982	1.982	Deceased
