# Supplementary material for: Systematic analysis of expression profiles of HMGB family members for prognostic application in non-small cell lung cancer
Source: Front Mol Biosci. 2022 Jul 18;9:844618. doi: 10.3389/fmolb.2022.844618 (PMC9340210; doi:10.3389/fmolb.2022.844618)
Supplement: Supplementary file 3 [file DataSheet3.PDF]

HMGB1

microRNA	LUAD		microRNA	LUSC	
	Coefficient-R	p-value		Coefficient-R	p-value
hsa-miR-106b-5p	0.214	1.01E-06	hsa-miR-106a-5p	0.373	3.92E-17
hsa-miR-17-5p	0.213	1.16E-06	hsa-miR-17-5p	0.352	2.69E-15
hsa-miR-92a-3p	0.177	5.89E-05	hsa-miR-106b-5p	0.302	1.69E-11
hsa-let-7d-5p	0.176	6.45E-05	hsa-miR-20a-5p	0.268	2.87E-09
hsa-miR-518d-3p	0.154	4.60E-04	hsa-miR-383-5p	0.257	1.37E-08
hsa-miR-106a-5p	0.152	5.36E-04	hsa-miR-25-3p	0.232	3.04E-07
hsa-miR-142-5p	0.15	6.58E-04	hsa-miR-577	0.228	5.26E-07
hsa-miR-520d-5p	0.15	6.39E-04	hsa-miR-20b-5p	0.225	7.33E-07
hsa-miR-143-3p	0.141	1.42E-03	hsa-miR-92a-3p	0.224	7.86E-07
hsa-miR-144-3p	0.14	1.48E-03	hsa-miR-363-3p	0.215	2.25E-06
hsa-miR-20a-5p	0.137	1.83E-03	hsa-miR-93-5p	0.19	2.93E-05
hsa-miR-320b	0.132	2.80E-03	hsa-let-7d-5p	0.187	4.10E-05
hsa-miR-1323	0.13	3.16E-03	hsa-miR-141-3p	0.179	8.67E-05
hsa-miR-98-5p	0.121	6.25E-03	hsa-miR-1301-3p	0.172	1.65E-04
hsa-miR-3918	0.121	6.32E-03	hsa-miR-524-5p	0.16	4.49E-04
hsa-miR-25-3p	0.12	6.43E-03	hsa-miR-320b	0.16	4.53E-04
hsa-miR-524-5p	0.119	7.15E-03	hsa-miR-124-3p	0.148	1.23E-03
hsa-miR-641	0.116	8.55E-03	hsa-let-7g-5p	0.145	1.50E-03
hsa-miR-10a-5p	0.115	9.22E-03	hsa-miR-32-5p	0.141	2.13E-03
hsa-miR-10b-5p	0.115	9.31E-03	hsa-miR-122-5p	0.137	2.75E-03
hsa-miR-190a-5p	0.114	9.97E-03	hsa-miR-181c-5p	0.131	4.29E-03
hsa-miR-627-5p	0.112	1.13E-02	hsa-miR-183-5p	0.126	6.01E-03
hsa-let-7g-5p	0.111	1.21E-02	hsa-miR-181d-5p	0.122	7.91E-03
hsa-miR-183-5p	0.104	1.82E-02	hsa-miR-129-5p	0.118	1.02E-02
hsa-miR-363-3p	0.095	3.22E-02	hsa-miR-320d	0.112	1.47E-02
hsa-miR-520h	0.091	3.88E-02	hsa-miR-200a-3p	0.111	1.58E-02
hsa-miR-522-3p	0.089	4.42E-02	hsa-miR-105-5p	0.11	1.62E-02
hsa-let-7b-5p	0.087	4.97E-02	hsa-miR-10b-5p	0.105	2.23E-02
hsa-miR-518f-3p	0.086	5.05E-02	hsa-miR-200b-3p	0.105	2.20E-02
hsa-miR-520g-3p	0.085	5.41E-02	hsa-miR-627-5p	0.096	3.61E-02
hsa-let-7a-5p	0.084	5.83E-02	hsa-miR-518d-3p	0.093	4.21E-02
hsa-let-7f-5p	0.084	5.82E-02	hsa-miR-1185-5p	0.093	4.18E-02
hsa-miR-498	0.084	5.73E-02	hsa-miR-641	0.091	4.86E-02
hsa-let-7i-5p	0.082	6.47E-02	hsa-miR-200c-3p	0.088	5.66E-02
hsa-miR-1197	0.082	6.46E-02	hsa-miR-204-5p	0.087	5.90E-02
hsa-miR-2115-3p	0.082	6.31E-02	hsa-miR-505-3p	0.087	5.74E-02
hsa-miR-4429	0.081	6.80E-02	hsa-miR-429	0.083	7.13E-02
hsa-miR-519d-3p	0.08	6.92E-02	hsa-miR-670-3p	0.083	7.09E-02
hsa-miR-216b-5p	0.078	7.90E-02	hsa-miR-4766-5p	0.082	7.25E-02
hsa-miR-541-5p	0.077	8.34E-02	hsa-miR-599	0.076	9.87E-02
hsa-miR-518a-3p	0.074	9.58E-02	hsa-miR-5000-3p	0.076	9.75E-02
hsa-miR-5000-3p	0.074	9.32E-02	hsa-miR-3174	0.073	1.12E-01
hsa-miR-518c-3p	0.073	9.96E-02	hsa-miR-450b-5p	0.072	1.17E-01
hsa-miR-3140-3p	0.073	1.01E-01	hsa-miR-142-3p	0.07	1.28E-01
hsa-miR-3679-5p	0.072	1.05E-01	hsa-miR-190a-5p	0.064	1.64E-01
hsa-miR-142-3p	0.069	1.21E-01	hsa-miR-186-5p	0.063	1.68E-01
hsa-miR-4661-5p	0.068	1.25E-01	hsa-miR-126-3p	0.062	1.81E-01
hsa-miR-383-5p	0.066	1.37E-01	hsa-miR-140-5p	0.061	1.87E-01
hsa-miR-4766-5p	0.066	1.36E-01	hsa-miR-483-3p	0.061	1.81E-01
hsa-miR-199a-3p	0.062	1.61E-01	hsa-miR-4661-5p	0.061	1.85E-01
hsa-miR-199b-3p	0.062	1.60E-01	hsa-miR-142-5p	0.06	1.93E-01
hsa-miR-889-3p	0.062	1.60E-01	hsa-miR-518f-3p	0.06	1.90E-01
hsa-miR-320d	0.062	1.64E-01	hsa-let-7i-5p	0.053	2.51E-01
hsa-miR-421	0.061	1.69E-01	hsa-miR-107	0.052	2.57E-01
hsa-miR-518b	0.06	1.73E-01	hsa-miR-488-3p	0.052	2.56E-01

hsa-miR-548o-3p	0.059	1.82E-01	hsa-miR-5581-3p	0.049	2.89E-01
hsa-miR-410-3p	0.056	2.07E-01	hsa-miR-380-3p	0.048	2.94E-01
hsa-miR-1301-3p	0.056	2.09E-01	hsa-miR-520d-5p	0.046	3.17E-01
hsa-miR-140-5p	0.055	2.15E-01	hsa-miR-136-5p	0.044	3.34E-01
hsa-miR-1185-5p	0.053	2.35E-01	hsa-miR-1298-5p	0.044	3.40E-01
hsa-miR-411-5p	0.051	2.51E-01	hsa-miR-98-5p	0.042	3.65E-01
hsa-miR-101-3p	0.05	2.64E-01	hsa-miR-513b-5p	0.039	3.98E-01
hsa-miR-3150a-3p	0.05	2.63E-01	hsa-miR-1179	0.039	3.99E-01
hsa-miR-31-5p	0.048	2.77E-01	hsa-miR-3150a-3p	0.039	3.98E-01
hsa-miR-20b-5p	0.048	2.80E-01	hsa-miR-411-5p	0.037	4.27E-01
hsa-miR-216a-5p	0.047	2.85E-01	hsa-miR-4756-5p	0.037	4.22E-01
hsa-miR-944	0.047	2.88E-01	hsa-miR-320a	0.036	4.35E-01
hsa-miR-577	0.043	3.34E-01	hsa-miR-520h	0.036	4.39E-01
hsa-miR-4756-5p	0.043	3.36E-01	hsa-miR-101-3p	0.034	4.59E-01
hsa-miR-493-5p	0.041	3.60E-01	hsa-miR-188-5p	0.034	4.65E-01
hsa-miR-488-3p	0.039	3.84E-01	hsa-miR-126-5p	0.033	4.78E-01
hsa-miR-105-5p	0.037	4.08E-01	hsa-miR-433-3p	0.031	5.00E-01
hsa-miR-483-3p	0.037	3.98E-01	hsa-miR-421	0.031	5.05E-01
hsa-miR-499b-5p	0.036	4.19E-01	hsa-miR-514b-5p	0.03	5.14E-01
hsa-miR-93-5p	0.034	4.39E-01	hsa-miR-548o-3p	0.028	5.43E-01
hsa-miR-429	0.034	4.45E-01	hsa-miR-144-3p	0.027	5.56E-01
hsa-miR-34c-5p	0.033	4.53E-01	hsa-miR-498	0.027	5.53E-01
hsa-miR-490-3p	0.029	5.07E-01	hsa-miR-518c-3p	0.026	5.70E-01
hsa-let-7c-5p	0.028	5.34E-01	hsa-miR-576-5p	0.025	5.82E-01
hsa-let-7e-5p	0.028	5.23E-01	hsa-miR-892c-5p	0.024	6.05E-01
hsa-miR-361-5p	0.028	5.21E-01	hsa-miR-520g-3p	0.023	6.14E-01
hsa-miR-32-5p	0.027	5.43E-01	hsa-miR-154-5p	0.019	6.73E-01
hsa-miR-190b	0.024	5.88E-01	hsa-miR-518a-3p	0.019	6.76E-01
hsa-miR-370-3p	0.023	5.98E-01	hsa-miR-522-3p	0.018	6.92E-01
hsa-miR-381-3p	0.023	5.96E-01	hsa-miR-299-3p	0.017	7.17E-01
hsa-miR-337-3p	0.022	6.25E-01	hsa-miR-449a	0.016	7.29E-01
hsa-miR-505-3p	0.021	6.39E-01	hsa-miR-519d-3p	0.015	7.38E-01
hsa-miR-665	0.021	6.41E-01	hsa-miR-4436a	0.013	7.84E-01
hsa-miR-494-3p	0.02	6.44E-01	hsa-miR-193a-3p	0.012	8.02E-01
hsa-miR-205-5p	0.019	6.69E-01	hsa-miR-541-5p	0.011	8.08E-01
hsa-miR-154-5p	0.019	6.68E-01	hsa-miR-490-3p	0.01	8.26E-01
hsa-miR-188-5p	0.019	6.75E-01	hsa-miR-510-5p	0.007	8.86E-01
hsa-miR-193a-3p	0.018	6.91E-01	hsa-miR-410-3p	0.006	8.88E-01
hsa-miR-107	0.016	7.24E-01	hsa-miR-889-3p	0.004	9.24E-01
hsa-miR-576-5p	0.016	7.17E-01	hsa-miR-1197	0.004	9.29E-01
hsa-miR-218-5p	0.014	7.55E-01	hsa-miR-103a-3p	0.003	9.50E-01
hsa-miR-186-5p	0.013	7.73E-01	hsa-miR-218-5p	0.003	9.44E-01
hsa-miR-449b-5p	0.013	7.70E-01	hsa-miR-514a-3p	0.003	9.42E-01
hsa-miR-450b-5p	0.013	7.74E-01	hsa-miR-616-3p	0.003	9.42E-01
hsa-miR-409-3p	0.012	7.82E-01	hsa-miR-92b-3p	0.001	9.90E-01
hsa-miR-126-3p	0.011	8.12E-01	hsa-miR-384	0	1.00E+00
hsa-miR-380-3p	0.011	8.11E-01	hsa-miR-1323	0	9.98E-01
hsa-miR-3174	0.011	7.97E-01	hsa-miR-5688	0	1.00E+00
hsa-miR-5590-3p	0.01	8.18E-01	hsa-miR-320c	-0.001	9.87E-01
hsa-miR-449a	0.006	8.87E-01	hsa-miR-3918	-0.001	9.83E-01
hsa-miR-211-5p	0.003	9.50E-01	hsa-miR-361-5p	-0.002	9.71E-01
hsa-miR-516a-5p	0.003	9.38E-01	hsa-miR-211-5p	-0.004	9.32E-01
hsa-miR-5581-3p	0.003	9.44E-01	hsa-miR-449b-5p	-0.005	9.08E-01
hsa-miR-5688	0.002	9.58E-01	hsa-miR-518b	-0.007	8.75E-01
hsa-miR-320c	0.001	9.87E-01	hsa-miR-216b-5p	-0.007	8.72E-01
hsa-miR-367-3p	0	1.00E+00	hsa-miR-944	-0.008	8.57E-01
hsa-miR-384	0	1.00E+00	hsa-miR-513c-5p	-0.009	8.52E-01
hsa-miR-382-3p	0	9.93E-01	hsa-miR-409-3p	-0.01	8.25E-01

hsa-miR-136-5p	-0.001	9.86E-01	hsa-miR-3140-3p	-0.01	8.22E-01
hsa-miR-379-3p	-0.002	9.66E-01	hsa-miR-579-3p	-0.011	8.07E-01
hsa-miR-4640-3p	-0.002	9.68E-01	hsa-miR-642a-3p	-0.011	8.14E-01
hsa-miR-506-5p	-0.002	9.73E-01	hsa-miR-582-5p	-0.014	7.62E-01
hsa-miR-3617-5p	-0.003	9.44E-01	hsa-miR-506-5p	-0.014	7.58E-01
hsa-miR-599	-0.004	9.24E-01	hsa-miR-493-5p	-0.015	7.45E-01
hsa-miR-1179	-0.004	9.32E-01	hsa-miR-3679-5p	-0.016	7.20E-01
hsa-miR-4739	-0.004	9.35E-01	hsa-miR-367-3p	-0.017	7.08E-01
hsa-miR-200c-3p	-0.006	8.84E-01	hsa-miR-495-3p	-0.017	7.11E-01
hsa-miR-642b-3p	-0.006	8.85E-01	hsa-miR-4429	-0.018	7.01E-01
hsa-miR-513c-5p	-0.008	8.51E-01	hsa-miR-642b-5p	-0.019	6.83E-01
hsa-miR-616-3p	-0.009	8.39E-01	hsa-miR-205-5p	-0.024	5.97E-01
hsa-miR-200b-3p	-0.01	8.27E-01	hsa-let-7f-5p	-0.029	5.28E-01
hsa-miR-129-5p	-0.011	8.11E-01	hsa-let-7c-5p	-0.031	4.96E-01
hsa-miR-299-3p	-0.011	8.02E-01	hsa-miR-375	-0.031	5.04E-01
hsa-miR-514b-5p	-0.012	7.88E-01	hsa-miR-199a-3p	-0.033	4.69E-01
hsa-miR-513b-5p	-0.013	7.64E-01	hsa-miR-199b-3p	-0.033	4.70E-01
hsa-miR-642a-3p	-0.013	7.75E-01	hsa-miR-34c-5p	-0.035	4.41E-01
hsa-miR-1298-5p	-0.014	7.60E-01	hsa-miR-190b	-0.036	4.29E-01
hsa-miR-126-5p	-0.015	7.29E-01	hsa-miR-664b-3p	-0.036	4.30E-01
hsa-miR-103a-3p	-0.017	7.09E-01	hsa-let-7b-5p	-0.038	4.11E-01
hsa-miR-642b-5p	-0.017	6.97E-01	hsa-miR-665	-0.038	4.10E-01
hsa-miR-496	-0.025	5.73E-01	hsa-miR-181b-5p	-0.04	3.79E-01
hsa-miR-193b-3p	-0.029	5.15E-01	hsa-miR-31-5p	-0.041	3.74E-01
hsa-miR-122-5p	-0.03	4.96E-01	hsa-miR-224-3p	-0.042	3.56E-01
hsa-miR-411-3p	-0.031	4.81E-01	hsa-miR-496	-0.043	3.48E-01
hsa-miR-892c-5p	-0.033	4.58E-01	hsa-let-7a-5p	-0.047	3.05E-01
hsa-miR-320a	-0.035	4.33E-01	hsa-miR-140-3p	-0.048	3.00E-01
hsa-miR-4436a	-0.035	4.26E-01	hsa-miR-10a-5p	-0.05	2.77E-01
hsa-miR-141-3p	-0.037	4.09E-01	hsa-miR-382-3p	-0.051	2.71E-01
hsa-miR-495-3p	-0.038	3.91E-01	hsa-miR-5590-3p	-0.052	2.60E-01
hsa-miR-181d-5p	-0.04	3.63E-01	hsa-miR-381-3p	-0.053	2.48E-01
hsa-miR-510-5p	-0.044	3.23E-01	hsa-miR-29c-3p	-0.054	2.42E-01
hsa-miR-514a-3p	-0.044	3.24E-01	hsa-miR-885-5p	-0.055	2.35E-01
hsa-miR-582-5p	-0.044	3.24E-01	hsa-miR-494-3p	-0.058	2.09E-01
hsa-miR-485-3p	-0.045	3.11E-01	hsa-miR-370-3p	-0.059	2.02E-01
hsa-miR-124-3p	-0.048	2.77E-01	hsa-miR-499b-5p	-0.062	1.80E-01
hsa-miR-200a-3p	-0.05	2.55E-01	hsa-miR-379-3p	-0.067	1.45E-01
hsa-miR-433-3p	-0.062	1.63E-01	hsa-miR-411-3p	-0.068	1.38E-01
hsa-miR-204-5p	-0.063	1.55E-01	hsa-miR-143-3p	-0.071	1.23E-01
hsa-miR-181b-5p	-0.07	1.14E-01	hsa-miR-216a-5p	-0.072	1.16E-01
hsa-miR-29c-3p	-0.072	1.02E-01	hsa-miR-2115-3p	-0.073	1.10E-01
hsa-miR-579-3p	-0.072	1.05E-01	hsa-miR-193b-3p	-0.078	9.00E-02
hsa-miR-885-5p	-0.077	8.11E-02	hsa-miR-4739	-0.078	8.89E-02
hsa-miR-140-3p	-0.085	5.57E-02	hsa-miR-337-3p	-0.082	7.30E-02
hsa-miR-375	-0.088	4.54E-02	hsa-miR-29b-3p	-0.086	6.00E-02
hsa-miR-92b-3p	-0.091	3.97E-02	hsa-miR-642b-3p	-0.088	5.54E-02
hsa-miR-181c-5p	-0.097	2.78E-02	hsa-miR-361-3p	-0.09	5.06E-02
hsa-miR-670-3p	-0.105	1.70E-02	hsa-miR-485-3p	-0.098	3.33E-02
hsa-miR-22-3p	-0.137	1.82E-03	hsa-miR-516a-5p	-0.1	3.00E-02
hsa-miR-181a-5p	-0.138	1.71E-03	hsa-miR-181a-5p	-0.101	2.81E-02
hsa-miR-224-3p	-0.179	4.65E-05	hsa-let-7e-5p	-0.114	1.32E-02
hsa-miR-29a-3p	-0.186	2.33E-05	hsa-miR-34a-5p	-0.121	8.55E-03
hsa-miR-29b-3p	-0.186	2.20E-05	hsa-miR-4640-3p	-0.124	6.63E-03
hsa-miR-664b-3p	-0.193	1.12E-05	hsa-miR-3617-5p	-0.144	1.62E-03
hsa-miR-361-3p	-0.209	1.85E-06	hsa-miR-29a-3p	-0.18	7.96E-05
hsa-miR-34a-5p	-0.257	3.70E-09	hsa-miR-22-3p	-0.29	1.26E-10

HMGB2

microRNA	LUAD		microRNA	LUSC	
	Coefficient-R	p-value		Coefficient-R	p-value
hsa-miR-421	0.317	2.15E-13	hsa-miR-9-5p	0.255	1.67E-08
hsa-miR-590-5p	0.293	1.41E-11	hsa-miR-96-5p	0.253	2.35E-08
hsa-miR-9-5p	0.262	1.86E-09	hsa-miR-181d-5p	0.251	2.95E-08
hsa-miR-425-5p	0.234	8.33E-08	hsa-miR-345-5p	0.245	6.13E-08
hsa-miR-345-5p	0.227	2.01E-07	hsa-miR-181b-5p	0.243	8.39E-08
hsa-miR-374a-3p	0.147	8.53E-04	hsa-miR-181c-5p	0.235	2.12E-07
hsa-miR-96-5p	0.13	3.30E-03	hsa-miR-361-5p	0.229	4.48E-07
hsa-miR-33b-5p	0.127	3.91E-03	hsa-miR-425-5p	0.227	5.76E-07
hsa-miR-361-5p	0.12	6.73E-03	hsa-miR-651-5p	0.183	5.83E-05
hsa-miR-320b	0.112	1.10E-02	hsa-miR-874-3p	0.183	6.14E-05
hsa-miR-33a-5p	0.11	1.26E-02	hsa-miR-556-3p	0.158	5.35E-04
hsa-miR-520d-5p	0.108	1.42E-02	hsa-miR-421	0.152	9.04E-04
hsa-miR-524-5p	0.099	2.54E-02	hsa-miR-374a-3p	0.149	1.14E-03
hsa-miR-2115-3p	0.096	3.02E-02	hsa-miR-590-5p	0.148	1.22E-03
hsa-miR-381-3p	0.094	3.35E-02	hsa-miR-181a-5p	0.143	1.82E-03
hsa-miR-651-5p	0.091	3.99E-02	hsa-miR-320a	0.141	2.04E-03
hsa-miR-556-3p	0.086	5.19E-02	hsa-miR-33b-5p	0.132	3.94E-03
hsa-miR-1271-5p	0.084	5.76E-02	hsa-miR-320b	0.122	8.03E-03
hsa-miR-320c	0.081	6.64E-02	hsa-miR-362-3p	0.112	1.44E-02
hsa-miR-889-3p	0.077	8.14E-02	hsa-miR-33a-5p	0.111	1.53E-02
hsa-miR-222-3p	0.058	1.89E-01	hsa-miR-218-5p	0.103	2.52E-02
hsa-miR-130a-5p	0.056	2.05E-01	hsa-miR-320c	0.095	3.82E-02
hsa-miR-543	0.052	2.40E-01	hsa-miR-320d	0.093	4.31E-02
hsa-miR-329-3p	0.046	3.00E-01	hsa-miR-5009-3p	0.071	1.22E-01
hsa-miR-221-3p	0.04	3.67E-01	hsa-miR-130a-5p	0.07	1.27E-01
hsa-miR-21-5p	0.025	5.77E-01	hsa-miR-885-5p	0.066	1.49E-01
hsa-miR-582-5p	0.024	5.91E-01	hsa-miR-1271-5p	0.058	2.08E-01
hsa-miR-362-3p	0.023	5.98E-01	hsa-miR-582-5p	0.053	2.50E-01
hsa-miR-320d	0.022	6.20E-01	hsa-miR-520d-5p	0.049	2.86E-01
hsa-miR-369-3p	0.015	7.32E-01	hsa-miR-524-5p	0.045	3.27E-01
hsa-miR-181b-5p	-0.003	9.43E-01	hsa-miR-490-3p	0.039	3.91E-01
hsa-miR-874-3p	-0.003	9.40E-01	hsa-miR-221-3p	0.016	7.24E-01
hsa-miR-320a	-0.031	4.78E-01	hsa-miR-2115-3p	0.012	7.99E-01
hsa-miR-490-3p	-0.059	1.81E-01	hsa-miR-23c	0.009	8.45E-01
hsa-miR-5009-3p	-0.072	1.03E-01	hsa-miR-222-3p	0.007	8.88E-01
hsa-miR-885-5p	-0.09	4.28E-02	hsa-miR-543	-0.025	5.86E-01
hsa-miR-181d-5p	-0.125	4.71E-03	hsa-miR-139-5p	-0.051	2.64E-01
hsa-miR-23c	-0.134	2.46E-03	hsa-miR-329-3p	-0.073	1.14E-01
hsa-miR-23a-3p	-0.136	2.09E-03	hsa-miR-23b-3p	-0.1	2.88E-02
hsa-miR-181a-5p	-0.168	1.38E-04	hsa-miR-381-3p	-0.1	3.00E-02
hsa-miR-218-5p	-0.194	1.00E-05	hsa-miR-889-3p	-0.109	1.74E-02
hsa-miR-22-3p	-0.205	2.86E-06	hsa-miR-369-3p	-0.136	3.02E-03
hsa-miR-139-5p	-0.242	2.90E-08	hsa-miR-21-5p	-0.159	5.14E-04
hsa-miR-181c-5p	-0.26	2.39E-09	hsa-miR-23a-3p	-0.193	2.28E-05
hsa-miR-23b-3p	-0.266	9.98E-10	hsa-miR-22-3p	-0.301	2.00E-11

HMGB3

microRNA	LUAD		microRNA	LUSC	
	Coefficient-R	p-value		Coefficient-R	p-value
hsa-miR-130b-3p	0.276	2.05E-10	hsa-miR-183-5p	0.362	3.58E-16
hsa-miR-629-5p	0.262	1.68E-09	hsa-miR-96-5p	0.32	9.72E-13
hsa-miR-375	0.251	8.00E-09	hsa-miR-130b-3p	0.309	5.87E-12
hsa-miR-301a-3p	0.223	3.25E-07	hsa-miR-106b-5p	0.266	3.81E-09
hsa-miR-96-5p	0.219	5.66E-07	hsa-miR-877-5p	0.257	1.26E-08
hsa-miR-183-5p	0.215	9.34E-07	hsa-miR-93-5p	0.255	1.66E-08

hsa-miR-106b-5p	0.206	2.49E-06	hsa-miR-301a-3p	0.252	2.73E-08
hsa-miR-326	0.199	5.58E-06	hsa-miR-582-5p	0.248	4.28E-08
hsa-miR-877-5p	0.197	6.90E-06	hsa-miR-128-3p	0.247	4.97E-08
hsa-miR-148b-3p	0.196	7.65E-06	hsa-miR-942-5p	0.247	5.02E-08
hsa-miR-105-5p	0.191	1.29E-05	hsa-miR-17-5p	0.236	1.99E-07
hsa-miR-503-5p	0.19	1.58E-05	hsa-miR-505-3p	0.231	3.43E-07
hsa-miR-708-5p	0.189	1.69E-05	hsa-miR-769-5p	0.213	2.93E-06
hsa-miR-181d-5p	0.182	3.54E-05	hsa-miR-542-3p	0.211	3.36E-06
hsa-miR-17-5p	0.181	3.66E-05	hsa-miR-20b-5p	0.209	4.20E-06
hsa-miR-128-3p	0.171	9.94E-05	hsa-miR-421	0.207	5.34E-06
hsa-miR-181c-5p	0.169	1.26E-04	hsa-miR-200a-3p	0.204	7.42E-06
hsa-miR-454-3p	0.166	1.63E-04	hsa-miR-589-5p	0.202	9.14E-06
hsa-miR-421	0.165	1.79E-04	hsa-miR-629-5p	0.2	1.11E-05
hsa-miR-615-3p	0.155	4.19E-04	hsa-miR-590-5p	0.197	1.54E-05
hsa-miR-141-3p	0.15	6.67E-04	hsa-miR-374b-5p	0.197	1.47E-05
hsa-miR-20b-5p	0.15	6.49E-04	hsa-miR-141-3p	0.196	1.77E-05
hsa-miR-212-3p	0.149	7.30E-04	hsa-miR-429	0.193	2.33E-05
hsa-miR-106a-5p	0.145	1.04E-03	hsa-miR-625-3p	0.191	2.74E-05
hsa-miR-4664-3p	0.142	1.26E-03	hsa-miR-20a-5p	0.187	4.29E-05
hsa-miR-579-3p	0.138	1.69E-03	hsa-miR-7-5p	0.186	4.62E-05
hsa-miR-331-3p	0.135	2.19E-03	hsa-miR-200c-3p	0.183	5.79E-05
hsa-miR-3139	0.134	2.45E-03	hsa-miR-576-5p	0.177	1.03E-04
hsa-miR-424-5p	0.133	2.48E-03	hsa-miR-579-3p	0.175	1.22E-04
hsa-miR-93-5p	0.13	3.20E-03	hsa-miR-107	0.172	1.66E-04
hsa-miR-552-3p	0.128	3.77E-03	hsa-miR-374a-5p	0.17	1.99E-04
hsa-miR-20a-5p	0.126	4.39E-03	hsa-miR-454-3p	0.166	2.80E-04
hsa-miR-107	0.124	4.79E-03	hsa-miR-200b-3p	0.163	3.70E-04
hsa-miR-556-3p	0.124	4.84E-03	hsa-miR-5000-3p	0.163	3.50E-04
hsa-miR-7-5p	0.12	6.60E-03	hsa-miR-4664-3p	0.161	4.35E-04
hsa-miR-590-5p	0.118	7.75E-03	hsa-miR-103a-3p	0.16	4.74E-04
hsa-miR-21-5p	0.114	1.00E-02	hsa-miR-375	0.157	5.91E-04
hsa-miR-124-3p	0.111	1.18E-02	hsa-miR-452-5p	0.148	1.20E-03
hsa-miR-616-3p	0.108	1.49E-02	hsa-miR-556-3p	0.145	1.56E-03
hsa-miR-5688	0.1	2.31E-02	hsa-miR-616-3p	0.142	1.97E-03
hsa-miR-4784	0.097	2.82E-02	hsa-miR-30b-5p	0.139	2.45E-03
hsa-miR-3187-3p	0.086	5.12E-02	hsa-miR-5195-3p	0.134	3.38E-03
hsa-miR-505-3p	0.081	6.64E-02	hsa-miR-4784	0.133	3.66E-03
hsa-miR-542-3p	0.08	7.07E-02	hsa-miR-216a-3p	0.133	3.63E-03
hsa-miR-302d-3p	0.076	8.67E-02	hsa-miR-532-3p	0.132	3.90E-03
hsa-miR-200c-3p	0.071	1.08E-01	hsa-miR-186-5p	0.13	4.62E-03
hsa-miR-576-5p	0.071	1.08E-01	hsa-miR-331-3p	0.123	7.52E-03
hsa-miR-545-3p	0.069	1.17E-01	hsa-miR-15b-5p	0.12	8.80E-03
hsa-miR-371a-5p	0.068	1.24E-01	hsa-miR-491-5p	0.12	9.12E-03
hsa-miR-4676-3p	0.066	1.36E-01	hsa-miR-664b-3p	0.12	8.73E-03
hsa-miR-624-3p	0.065	1.45E-01	hsa-miR-185-5p	0.118	9.93E-03
hsa-miR-5000-3p	0.063	1.56E-01	hsa-miR-3187-3p	0.116	1.15E-02
hsa-miR-4677-3p	0.061	1.68E-01	hsa-miR-660-5p	0.111	1.60E-02
hsa-miR-5195-3p	0.058	1.88E-01	hsa-miR-194-5p	0.11	1.63E-02
hsa-miR-582-5p	0.053	2.36E-01	hsa-miR-106a-5p	0.109	1.76E-02
hsa-miR-650	0.05	2.54E-01	hsa-miR-532-5p	0.108	1.82E-02
hsa-miR-1252-5p	0.045	3.13E-01	hsa-miR-296-3p	0.102	2.62E-02
hsa-miR-589-5p	0.043	3.26E-01	hsa-miR-105-5p	0.096	3.62E-02
hsa-miR-1179	0.042	3.40E-01	hsa-miR-490-3p	0.096	3.66E-02
hsa-miR-380-3p	0.04	3.65E-01	hsa-miR-624-3p	0.093	4.17E-02
hsa-miR-378g	0.04	3.67E-01	hsa-miR-181b-5p	0.09	5.05E-02
hsa-miR-376a-3p	0.036	4.13E-01	hsa-miR-362-3p	0.09	5.01E-02
hsa-miR-132-3p	0.035	4.25E-01	hsa-miR-330-5p	0.088	5.40E-02
hsa-miR-512-3p	0.034	4.40E-01	hsa-miR-130a-3p	0.087	5.79E-02

hsa-miR-122-5p	0.031	4.85E-01	hsa-miR-3139	0.087	5.88E-02
hsa-miR-769-5p	0.031	4.87E-01	hsa-miR-424-5p	0.085	6.57E-02
hsa-miR-519c-3p	0.029	5.09E-01	hsa-miR-642b-3p	0.081	7.74E-02
hsa-miR-329-3p	0.028	5.33E-01	hsa-miR-30d-5p	0.08	8.10E-02
hsa-miR-381-3p	0.024	5.89E-01	hsa-miR-503-5p	0.077	9.52E-02
hsa-miR-409-3p	0.024	5.82E-01	hsa-miR-4676-3p	0.077	9.56E-02
hsa-miR-889-3p	0.024	5.85E-01	hsa-miR-181d-5p	0.075	1.01E-01
hsa-miR-27b-3p	0.021	6.36E-01	hsa-miR-627-5p	0.072	1.20E-01
hsa-miR-129-2-3p	0.02	6.44E-01	hsa-miR-876-5p	0.07	1.30E-01
hsa-miR-376b-3p	0.019	6.76E-01	hsa-miR-15a-5p	0.068	1.41E-01
hsa-miR-371a-3p	0.014	7.49E-01	hsa-miR-148b-3p	0.068	1.37E-01
hsa-miR-526b-5p	0.014	7.50E-01	hsa-miR-4677-3p	0.068	1.42E-01
hsa-miR-365a-3p	0.013	7.77E-01	hsa-miR-224-3p	0.066	1.48E-01
hsa-miR-410-3p	0.013	7.65E-01	hsa-miR-641	0.065	1.55E-01
hsa-miR-942-5p	0.013	7.72E-01	hsa-miR-3163	0.064	1.66E-01
hsa-miR-365b-3p	0.013	7.78E-01	hsa-miR-362-5p	0.061	1.87E-01
hsa-miR-134-5p	0.012	7.89E-01	hsa-miR-4756-5p	0.059	1.97E-01
hsa-miR-3681-3p	0.012	7.85E-01	hsa-miR-371a-5p	0.051	2.63E-01
hsa-miR-24-3p	0.01	8.23E-01	hsa-miR-3150b-3p	0.051	2.64E-01
hsa-miR-369-3p	0.009	8.32E-01	hsa-miR-132-3p	0.05	2.77E-01
hsa-miR-373-3p	0.007	8.83E-01	hsa-miR-892c-3p	0.047	3.02E-01
hsa-miR-655-3p	0.006	8.89E-01	hsa-miR-1298-5p	0.046	3.13E-01
hsa-miR-520a-5p	0.005	9.07E-01	hsa-miR-326	0.045	3.24E-01
hsa-miR-516b-5p	0.004	9.27E-01	hsa-miR-3167	0.044	3.44E-01
hsa-miR-330-5p	0.004	9.25E-01	hsa-miR-28-3p	0.039	4.02E-01
hsa-miR-372-3p	0.002	9.71E-01	hsa-miR-668-3p	0.037	4.26E-01
hsa-miR-518f-5p	0.002	9.58E-01	hsa-miR-212-3p	0.034	4.61E-01
hsa-miR-524-5p	-0.001	9.85E-01	hsa-miR-483-3p	0.034	4.63E-01
hsa-miR-641	-0.001	9.78E-01	hsa-miR-545-3p	0.031	4.97E-01
hsa-miR-668-3p	-0.001	9.90E-01	hsa-miR-135a-5p	0.03	5.08E-01
hsa-miR-642b-3p	-0.001	9.86E-01	hsa-miR-588	0.029	5.29E-01
hsa-miR-892c-3p	-0.001	9.90E-01	hsa-miR-1294	0.028	5.47E-01
hsa-miR-4739	-0.002	9.61E-01	hsa-miR-376b-3p	0.026	5.65E-01
hsa-miR-498	-0.003	9.46E-01	hsa-miR-1297	0.026	5.74E-01
hsa-miR-628-5p	-0.003	9.50E-01	hsa-miR-519a-3p	0.025	5.93E-01
hsa-miR-588	-0.004	9.36E-01	hsa-miR-302d-3p	0.024	5.97E-01
hsa-miR-876-5p	-0.004	9.27E-01	hsa-miR-552-3p	0.021	6.50E-01
hsa-miR-520e	-0.006	8.95E-01	hsa-miR-302b-3p	0.02	6.69E-01
hsa-miR-519d-3p	-0.006	8.96E-01	hsa-miR-338-3p	0.019	6.80E-01
hsa-miR-377-3p	-0.007	8.75E-01	hsa-miR-515-5p	0.017	7.15E-01
hsa-miR-4761-3p	-0.008	8.49E-01	hsa-miR-4295	0.017	7.07E-01
hsa-miR-429	-0.01	8.26E-01	hsa-miR-26b-5p	0.013	7.72E-01
hsa-miR-665	-0.012	7.84E-01	hsa-miR-222-3p	0.013	7.72E-01
hsa-miR-519a-3p	-0.014	7.59E-01	hsa-miR-495-3p	0.013	7.73E-01
hsa-miR-520d-5p	-0.015	7.34E-01	hsa-miR-4761-3p	0.013	7.81E-01
hsa-miR-627-5p	-0.015	7.31E-01	hsa-miR-380-3p	0.012	7.98E-01
hsa-miR-1197	-0.016	7.17E-01	hsa-miR-16-5p	0.01	8.28E-01
hsa-miR-320b	-0.017	7.04E-01	hsa-miR-28-5p	0.01	8.31E-01
hsa-miR-520a-3p	-0.018	6.88E-01	hsa-miR-124-3p	0.01	8.31E-01
hsa-miR-525-5p	-0.018	6.85E-01	hsa-miR-320a	0.01	8.35E-01
hsa-miR-200a-3p	-0.019	6.61E-01	hsa-miR-1252-5p	0.009	8.49E-01
hsa-miR-302b-3p	-0.021	6.30E-01	hsa-miR-513a-5p	0.008	8.61E-01
hsa-miR-296-3p	-0.021	6.38E-01	hsa-miR-510-5p	0.008	8.63E-01
hsa-miR-520f-3p	-0.022	6.16E-01	hsa-miR-371a-3p	0.007	8.74E-01
hsa-miR-510-5p	-0.022	6.26E-01	hsa-miR-520a-5p	0.007	8.87E-01
hsa-miR-320d	-0.023	6.02E-01	hsa-miR-519a-5p	0.007	8.84E-01
hsa-miR-3150b-3p	-0.023	6.07E-01	hsa-miR-520d-5p	0.006	8.96E-01
hsa-miR-4701-5p	-0.023	6.09E-01	hsa-miR-30c-5p	0.004	9.23E-01

hsa-miR-515-5p	-0.025	5.68E-01	hsa-miR-320b	0.004	9.31E-01
hsa-miR-526b-3p	-0.025	5.74E-01	hsa-miR-516b-5p	0.003	9.54E-01
hsa-miR-541-3p	-0.025	5.77E-01	hsa-miR-320c	0.003	9.46E-01
hsa-miR-654-5p	-0.026	5.58E-01	hsa-miR-122-5p	0.001	9.87E-01
hsa-miR-520b	-0.027	5.48E-01	hsa-miR-302c-3p	0.001	9.80E-01
hsa-miR-520c-3p	-0.027	5.44E-01	hsa-miR-129-2-3p	0.001	9.77E-01
hsa-miR-214-3p	-0.029	5.11E-01	hsa-miR-650	-0.001	9.85E-01
hsa-miR-491-5p	-0.029	5.14E-01	hsa-miR-221-3p	-0.002	9.71E-01
hsa-miR-16-5p	-0.03	5.00E-01	hsa-miR-520e	-0.004	9.23E-01
hsa-miR-130a-3p	-0.03	4.91E-01	hsa-miR-526b-5p	-0.005	9.17E-01
hsa-miR-205-5p	-0.033	4.59E-01	hsa-miR-541-3p	-0.008	8.61E-01
hsa-miR-15b-5p	-0.033	4.53E-01	hsa-miR-4701-5p	-0.01	8.34E-01
hsa-miR-506-5p	-0.033	4.62E-01	hsa-miR-365b-3p	-0.01	8.28E-01
hsa-miR-519a-5p	-0.034	4.41E-01	hsa-miR-365a-3p	-0.011	8.19E-01
hsa-miR-103a-3p	-0.037	4.10E-01	hsa-miR-494-3p	-0.012	7.90E-01
hsa-miR-302a-3p	-0.037	4.01E-01	hsa-miR-665	-0.012	7.99E-01
hsa-miR-520d-3p	-0.037	4.03E-01	hsa-miR-514b-5p	-0.013	7.84E-01
hsa-miR-506-3p	-0.037	4.03E-01	hsa-miR-383-5p	-0.015	7.48E-01
hsa-miR-1298-5p	-0.038	3.90E-01	hsa-miR-1179	-0.015	7.51E-01
hsa-miR-494-3p	-0.04	3.72E-01	hsa-miR-522-3p	-0.018	6.98E-01
hsa-miR-3163	-0.042	3.39E-01	hsa-miR-628-5p	-0.02	6.65E-01
hsa-miR-27a-3p	-0.043	3.31E-01	hsa-miR-543	-0.02	6.61E-01
hsa-miR-543	-0.043	3.29E-01	hsa-miR-513b-5p	-0.021	6.50E-01
hsa-miR-522-3p	-0.045	3.10E-01	hsa-miR-181a-5p	-0.022	6.31E-01
hsa-miR-520g-3p	-0.049	2.65E-01	hsa-miR-302a-3p	-0.023	6.18E-01
hsa-miR-1294	-0.056	2.07E-01	hsa-miR-320d	-0.023	6.15E-01
hsa-miR-3167	-0.056	2.03E-01	hsa-miR-329-3p	-0.026	5.74E-01
hsa-miR-152-3p	-0.058	1.92E-01	hsa-miR-708-5p	-0.026	5.73E-01
hsa-miR-483-3p	-0.058	1.93E-01	hsa-miR-369-3p	-0.027	5.64E-01
hsa-miR-181b-5p	-0.059	1.85E-01	hsa-miR-520a-3p	-0.027	5.52E-01
hsa-miR-433-3p	-0.059	1.82E-01	hsa-miR-181c-5p	-0.029	5.29E-01
hsa-miR-519b-3p	-0.06	1.75E-01	hsa-miR-615-3p	-0.03	5.10E-01
hsa-miR-320c	-0.062	1.61E-01	hsa-miR-513c-5p	-0.03	5.16E-01
hsa-miR-4756-5p	-0.062	1.59E-01	hsa-miR-27b-3p	-0.031	4.97E-01
hsa-miR-30d-5p	-0.063	1.52E-01	hsa-miR-655-3p	-0.033	4.78E-01
hsa-miR-135a-5p	-0.063	1.52E-01	hsa-miR-498	-0.034	4.62E-01
hsa-miR-186-5p	-0.064	1.47E-01	hsa-miR-520f-3p	-0.034	4.62E-01
hsa-miR-520h	-0.064	1.50E-01	hsa-miR-518f-5p	-0.034	4.61E-01
hsa-miR-200b-3p	-0.065	1.44E-01	hsa-miR-526b-3p	-0.035	4.41E-01
hsa-miR-216a-3p	-0.068	1.26E-01	hsa-miR-654-5p	-0.035	4.46E-01
hsa-miR-26b-5p	-0.069	1.19E-01	hsa-miR-30e-5p	-0.036	4.35E-01
hsa-miR-495-3p	-0.072	1.05E-01	hsa-miR-410-3p	-0.036	4.34E-01
hsa-miR-362-3p	-0.072	1.05E-01	hsa-miR-378g	-0.041	3.69E-01
hsa-miR-664b-3p	-0.072	1.04E-01	hsa-miR-373-3p	-0.042	3.62E-01
hsa-miR-185-5p	-0.073	9.99E-02	hsa-miR-519d-3p	-0.043	3.45E-01
hsa-miR-625-3p	-0.073	9.90E-02	hsa-miR-506-3p	-0.045	3.22E-01
hsa-miR-15a-5p	-0.074	9.28E-02	hsa-miR-1197	-0.045	3.23E-01
hsa-miR-148a-3p	-0.074	9.34E-02	hsa-miR-524-5p	-0.049	2.91E-01
hsa-miR-30e-5p	-0.089	4.42E-02	hsa-miR-4739	-0.05	2.80E-01
hsa-miR-490-3p	-0.093	3.56E-02	hsa-miR-519c-3p	-0.052	2.62E-01
hsa-miR-513b-5p	-0.095	3.18E-02	hsa-miR-525-5p	-0.055	2.34E-01
hsa-miR-642a-3p	-0.095	3.21E-02	hsa-miR-520g-3p	-0.055	2.30E-01
hsa-miR-514b-5p	-0.099	2.53E-02	hsa-miR-342-3p	-0.057	2.13E-01
hsa-miR-28-5p	-0.106	1.69E-02	hsa-miR-125a-5p	-0.059	1.97E-01
hsa-miR-532-3p	-0.107	1.52E-02	hsa-miR-24-3p	-0.06	1.89E-01
hsa-miR-374b-5p	-0.109	1.36E-02	hsa-miR-140-3p	-0.061	1.86E-01
hsa-miR-374a-5p	-0.11	1.29E-02	hsa-miR-377-3p	-0.063	1.69E-01
hsa-miR-143-3p	-0.114	9.74E-03	hsa-miR-101-3p	-0.064	1.64E-01

hsa-miR-513a-5p	-0.118	7.31E-03	hsa-miR-372-3p	-0.065	1.60E-01
hsa-miR-383-5p	-0.122	5.72E-03	hsa-miR-140-5p	-0.068	1.41E-01
hsa-miR-342-3p	-0.122	5.90E-03	hsa-miR-520h	-0.07	1.29E-01
hsa-miR-338-3p	-0.124	4.85E-03	hsa-miR-381-3p	-0.074	1.06E-01
hsa-miR-660-5p	-0.13	3.15E-03	hsa-miR-519b-3p	-0.075	1.02E-01
hsa-miR-362-5p	-0.132	2.72E-03	hsa-miR-889-3p	-0.075	1.04E-01
hsa-miR-125a-5p	-0.139	1.57E-03	hsa-miR-497-5p	-0.076	9.80E-02
hsa-miR-26a-5p	-0.147	8.34E-04	hsa-miR-520c-3p	-0.076	9.63E-02
hsa-miR-452-5p	-0.147	8.55E-04	hsa-miR-520b	-0.078	9.00E-02
hsa-miR-497-5p	-0.15	6.46E-04	hsa-miR-139-5p	-0.083	6.98E-02
hsa-miR-513c-5p	-0.151	6.10E-04	hsa-miR-520d-3p	-0.083	7.02E-02
hsa-miR-181a-5p	-0.152	5.64E-04	hsa-miR-433-3p	-0.088	5.42E-02
hsa-miR-532-5p	-0.159	3.04E-04	hsa-miR-512-3p	-0.088	5.62E-02
hsa-miR-224-3p	-0.164	2.01E-04	hsa-miR-409-3p	-0.089	5.30E-02
hsa-miR-139-5p	-0.169	1.21E-04	hsa-miR-146b-5p	-0.091	4.79E-02
hsa-miR-125b-5p	-0.174	7.76E-05	hsa-miR-642a-3p	-0.1	2.96E-02
hsa-miR-320a	-0.175	6.99E-05	hsa-miR-506-5p	-0.102	2.55E-02
hsa-miR-145-5p	-0.177	5.73E-05	hsa-miR-214-3p	-0.104	2.33E-02
hsa-miR-146b-5p	-0.193	1.06E-05	hsa-miR-3681-3p	-0.108	1.83E-02
hsa-miR-195-5p	-0.194	1.01E-05	hsa-miR-134-5p	-0.109	1.71E-02
hsa-miR-101-3p	-0.199	5.74E-06	hsa-miR-26a-5p	-0.11	1.63E-02
hsa-miR-140-5p	-0.2	5.26E-06	hsa-miR-195-5p	-0.115	1.23E-02
hsa-miR-150-5p	-0.2	4.88E-06	hsa-miR-150-5p	-0.125	6.30E-03
hsa-miR-28-3p	-0.208	2.16E-06	hsa-miR-145-5p	-0.129	5.00E-03
hsa-miR-30c-5p	-0.214	1.05E-06	hsa-miR-21-5p	-0.13	4.53E-03
hsa-miR-194-5p	-0.216	8.28E-07	hsa-miR-205-5p	-0.13	4.42E-03
hsa-miR-135b-5p	-0.234	8.32E-08	hsa-miR-152-3p	-0.161	4.31E-04
hsa-miR-140-3p	-0.27	4.95E-10	hsa-miR-143-3p	-0.163	3.70E-04
hsa-miR-146a-5p	-0.352	2.06E-16	hsa-miR-148a-3p	-0.177	1.03E-04
hsa-miR-222-3p	-0.359	5.28E-17	hsa-miR-27a-3p	-0.187	3.93E-05
hsa-miR-221-3p	-0.408	6.52E-22	hsa-miR-125b-5p	-0.188	3.61E-05
hsa-miR-30a-5p	-0.482	4.27E-31	hsa-miR-30a-5p	-0.21	3.91E-06
			hsa-miR-135b-5p	-0.227	5.85E-07
			hsa-miR-146a-5p	-0.352	2.06E-16
