# Supplementary material for: Systematic analysis of expression profiles of HMGB family members for prognostic application in non-small cell lung cancer
Source: Front Mol Biosci. 2022 Jul 18;9:844618. doi: 10.3389/fmolb.2022.844618 (PMC9340210; doi:10.3389/fmolb.2022.844618)
Supplement: Supplementary file 4 [file Table2.docx]

Supplementary Material

## Supplementary Figures

**Figure S1**


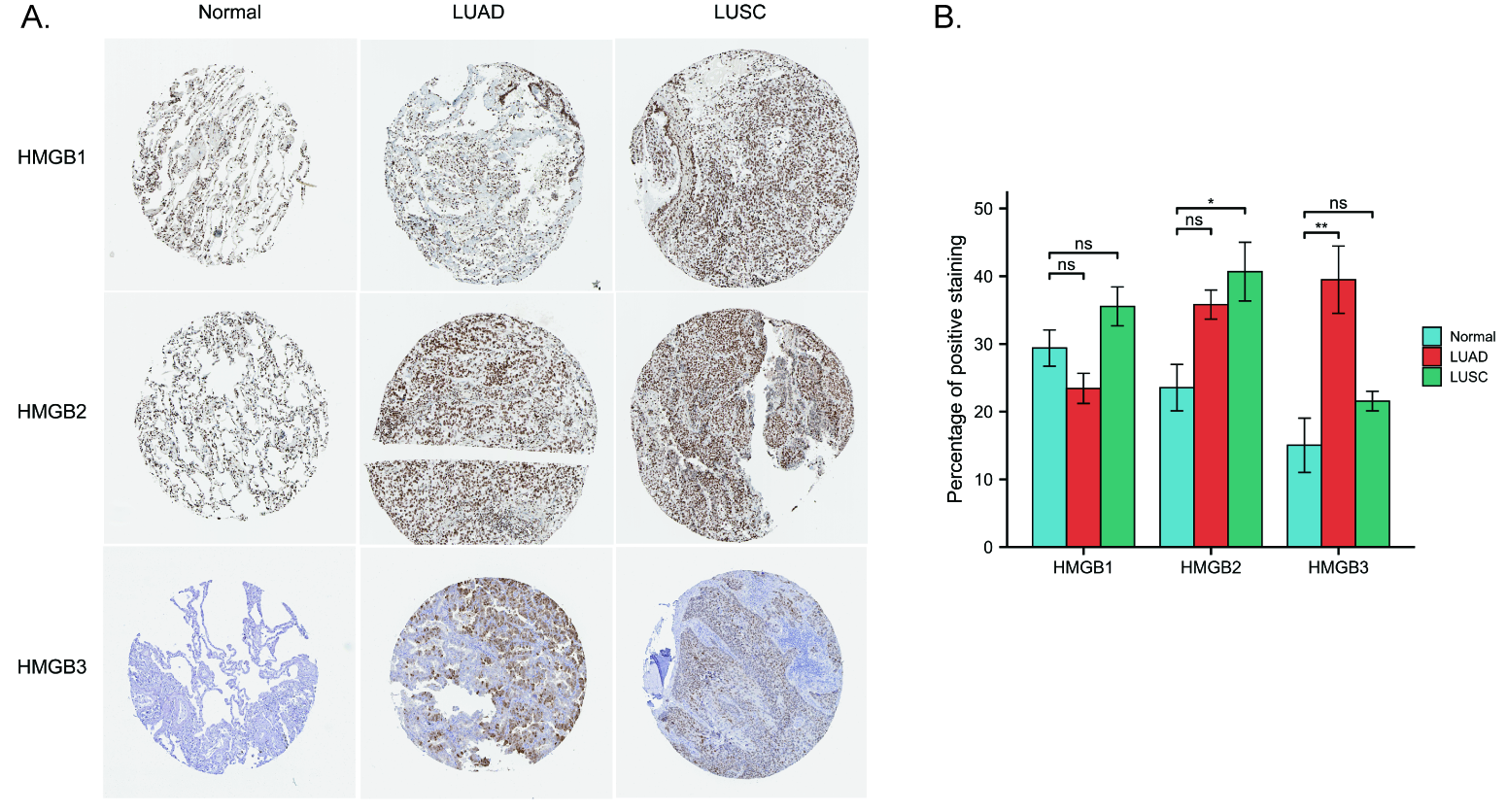


**Figure S1.** Immunohistochemical staining for HMGB1, HMGB2, and HMGB3 in normal lung tissues and NSCLC tumors. (A) All the immunostaining pictures were downloaded from HPA database. (B) Quantification of protein expression by using ImageJ. HPA, the Human Protein Atlas. ***p* < 0.01, **p* < 0.05; ns, not statistically significant.

**Figure S2**

**
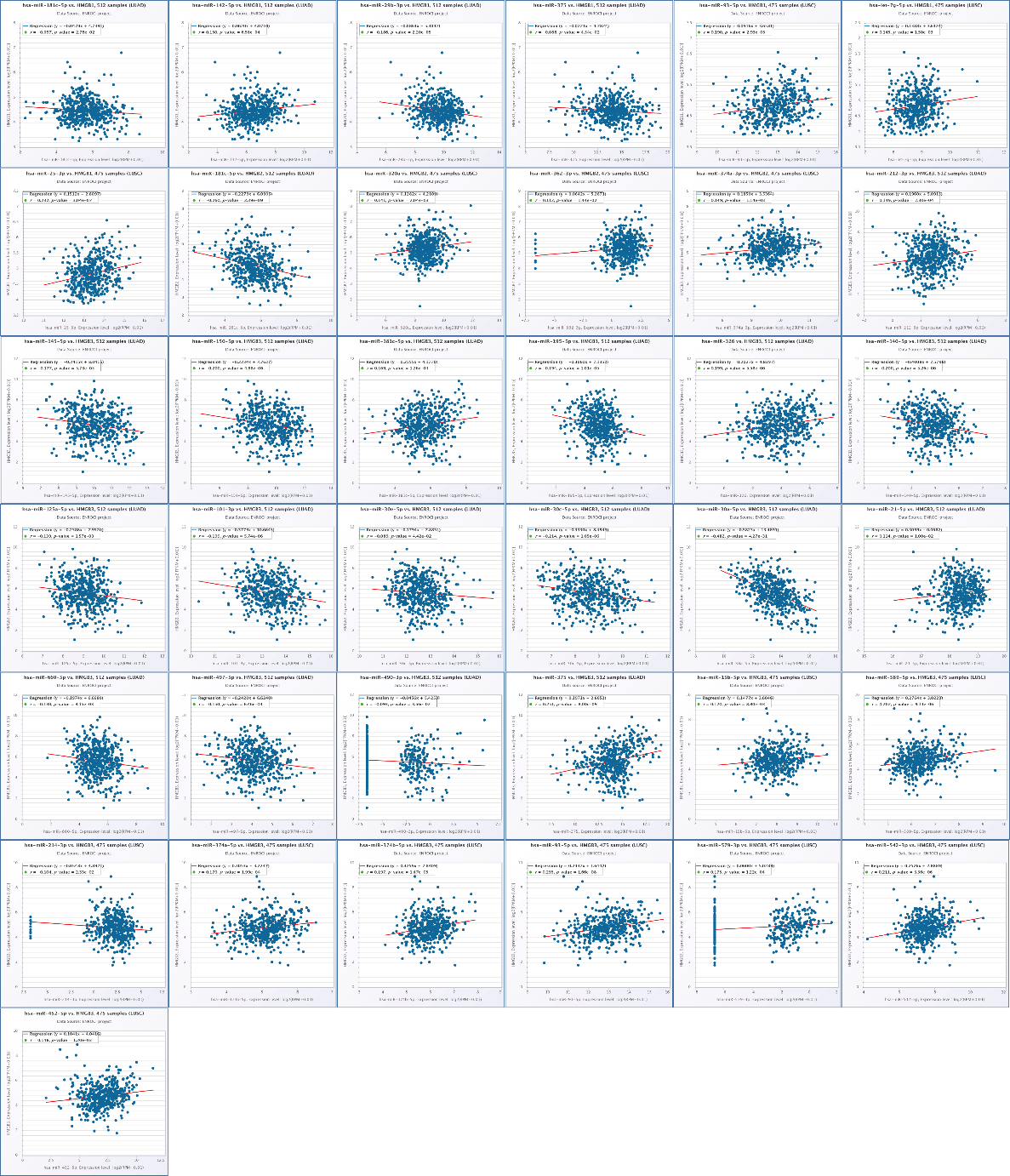
**

**Figure S2.** Correlation of HMGBs expression and prognostic microRNAs in LUAD or LUSC. Data analysis from starBase2.0 database.
