# Supplementary material for: Systematic analysis of expression profiles of HMGB family members for prognostic application in non-small cell lung cancer
Source: Front Mol Biosci. 2022 Jul 18;9:844618. doi: 10.3389/fmolb.2022.844618 (PMC9340210; doi:10.3389/fmolb.2022.844618)
Supplement: Supplementary file 5 [file DataSheet1.PDF]

```
CLUSTAL format alignment by MAFFT FFT-NS-i (v7.487)


NC_000013.11:c3 TTTCTGCGGAGGGATTACGCTGACGAAAGAGACCTGCTTGCGCGTCGCTGTTCCGTGGTC
NC_000001.11:33 ------------------------------------------------------------
NC_000023.11:15 ------------------------------------------------------------
NC_000004.12:c1 ------------------------------------------------------------


NC_000013.11:c3 CGCGCGAGCGTGGTCGGGAGCCGCTGGTTCCTGGGGTGACCCGCGGAGGTGGGAGAGGGA
NC_000001.11:33 ------------------------------------------------------------
NC_000023.11:15 ------------------------------------------------------------
NC_000004.12:c1 ------------------------------------------------------------


NC_000013.11:c3 AGGGCTTCCGAAGCCGGCGGGGGTGCCATGGACCCTCTCCGCCGGCGCGGCCTTCACAGC
NC_000001.11:33 ------------------------------------------------------------
NC_000023.11:15 ------------------------------------------------------------
NC_000004.12:c1 ------------------------------------------------------------


NC_000013.11:c3 TGGGCCGCGCCGGGCATCCGTAGTCCGCTCTCCCAAAGCCTCGGTGGAGCTGAAGCTGCC
NC_000001.11:33 ------------------------------------------------------------
NC_000023.11:15 --------------AGTTTGTAGTTCAGCTTC----------------------------
NC_000004.12:c1 ------------------------------------------------------------


NC_000013.11:c3 ACAGAGTGCATGTTCACAAAGGGTCATCACACACGGAGCTGCCCCTCCCTGTCTCCCTAG
NC_000001.11:33 ------------------------------------------------------------
NC_000023.11:15 ------------------------------------------------------------
NC_000004.12:c1 ------------------------------------------------------------


NC_000013.11:c3 AGCCCATCTTCGAGGCCAGGGGCTTTTCTACCAGGATTCTGGGGTGTTTCTCCTCCTTTC
NC_000001.11:33 ------------------------------------------------------------
NC_000023.11:15 ------------AGGCCAAG----------------------------------------
NC_000004.12:c1 ------------------------------------------------------------


NC_000013.11:c3 CTCCCTCCCAGATCTTCTCACGGTAAGGGGAGCAGCGAAAGCGCAGGGACTTTGCATTCC
NC_000001.11:33 ------------------------------------------------------------
NC_000023.11:15 ------------------------------------------------------------
NC_000004.12:c1 ------------------------------------------------------------


NC_000013.11:c3 ACGACCCGTTCTGACTAGTCAACAGCCGATCTGTCCCTGCTGCTCTAATTCCAGCTGCCC
NC_000001.11:33 ------------------------------------------------------------
NC_000023.11:15 ------------------------------------------------------------
NC_000004.12:c1 ------------------------------------------------------------


NC_000013.11:c3 TGCCTTGTTTTAACTTCAGAGAAAGGGGGAGTTCTCATTTGATAAGTTTAAGCCTTTGCT
NC_000001.11:33 ------------------------------------------------------------
NC_000023.11:15 -----TGCTTCAGCTCCATATAAGAGGTAGCTT---------------------------
NC_000004.12:c1 ------------------------------------------------------------


NC_000013.11:c3 TTCGTAGGAAGGTCATGTGGCTTAAGGGACATCGTGACCGCGTATGCTATTTCTGCCTGT
NC_000001.11:33 ------------------------------------------------------------
NC_000023.11:15 ------------------------------------------------------------
NC_000004.12:c1 ------------------------------------------------------------


NC_000013.11:c3 GCATTCTAAATCTTGGGGGCAGCATATTCCAGAAGTCCTTTTGGTCGATTGGTTCTGTGT
```

```
NC_000001.11:33 ------------------------------------------------------------
NC_000023.11:15 ----------TCTTGAGAGCAG--------------------------------------
NC_000004.12:c1 ------------------------------------------------------------

NC_000013.11:c3 CCTAGGATAACAATTTGTAGTTTCTGACCATTTCTTTACAGAAAAACCACAATTGGTATT
NC_000001.11:33 ------------------------------------------------------------
NC_000023.11:15 ------------------------------------------------------------
NC_000004.12:c1 ------------------------------------------------------------

NC_000013.11:c3 TTGGACTGCGGGGTTTTTTAGTGGTCTCAAACTAAATGATTATATTCTGGAATAATGCTG
NC_000001.11:33 ------------------------------------------------------------
NC_000023.11:15 ------------------------------------------------------------
NC_000004.12:c1 ------------------------------------------------------------

NC_000013.11:c3 ACAATTTGGATAGGGTGGTGTGGAGGAAACAAGTCTCGTGTAGAAGAAATTATTTAGTAA
NC_000001.11:33 ------------------------------------------------------------
NC_000023.11:15 ------------------------------------------------------------
NC_000004.12:c1 ------------------------------------------------------------

NC_000013.11:c3 AAAGGATTTTAGTTTTTGGTACTTCTGAATGCAAATGGCCAAGGAATCCAGCAGTTTGTT
NC_000001.11:33 ------------------------------------------------------------
NC_000023.11:15 ------------------------------------------------------------
NC_000004.12:c1 ------------------------------------------------------------

NC_000013.11:c3 GGGGTTTCTCCAGACAAAAATAGGCTGGTTAGTTTGTTTACCTTTTTCTGTTGCTTTACT
NC_000001.11:33 ------------------------------------------------------------
NC_000023.11:15 ------------------------------------------------------------
NC_000004.12:c1 ------------------------------------------------------------

NC_000013.11:c3 AGATTGTTAATGTATTTGTATATTTAAGATCTAGCTAGTTAGGTTTTTCTTTTTTATGAA
NC_000001.11:33 ------------------------------------------------------------
NC_000023.11:15 ------------------------------------------------------------
NC_000004.12:c1 ------------------------------------------------------------

NC_000013.11:c3 TTGTGATTAAAATTGGAGAAAGTAGTACTTCCTCCTGAAATAGACAAAACTCAGAGGGTT
NC_000001.11:33 ------------------------------------------------------------
NC_000023.11:15 ------------------------------------------------------------
NC_000004.12:c1 ------------------------------------------------------------

NC_000013.11:c3 GTTTTCTTGTTCACCACTGCTACTTCCATTTTTAGAATGGAATACACTATTTGTAAAAAA
NC_000001.11:33 ------------------------------------------------------------
NC_000023.11:15 ---------------------------------AGATCGAAATATTTCCTATGCAAAGGA
NC_000004.12:c1 ------------------------------------------------------------

NC_000013.11:c3 CAAACTTGCTGAGTATTAACTCATAAATTGAACAAAACAATTGGTTCAGTTAAAATGTAG
NC_000001.11:33 ------------------------------------------------------------
NC_000023.11:15 TGG---------------------------------------------------------
NC_000004.12:c1 ------------------------------------------------------------

NC_000013.11:c3 CTAAAATTGTAAGACTGCAAAACAACATCACCAAACTGAATTGTATTTTTTAAAACGTTG
NC_000001.11:33 ----------------------------------------------------AAGTGGTG
NC_000023.11:15 ----------------------------------------------------AAATACTT
NC_000004.12:c1 ----------------------------------------------------AAA-----
                                                                    **.
```

```
NC_000013.11:c3 TCTGCAAAGTTGAATTTTAGTCTTTCAGTGTAAATTTGGTATTTTTTCAGAAGAAAAATT
NC_000001.11:33 TCTGCCAGAT----------TTCCCCGCTGTAAAGTTACTATTTGTCC------------
NC_000023.11:15 CCTG----------------CGCGTAAGCGCAATCCTTTCATCATTCGAAGTATGTGGGG
NC_000004.12:c1 ------------------------------------------------------------

NC_000013.11:c3 AGTAACAGAAACAAAATGGAGGCGGGGCTTGAGTTGTAAAG-AGAAAGGAAGGGATTTTA
NC_000001.11:33 ------------------------------------------------------------
NC_000023.11:15 AGAAATAGATGGTCAATGG--GCAGGGTTGGGGCAGTGGGGCAGGGAAGCATGGATCTT-
NC_000004.12:c1 ------------------------------------------------------------

NC_000013.11:c3 GTGTGTTCTGTGTTTTCGCTCTAGAGCTTTTTCCTCTTGTAAAAGGTAGTACAAGACTTA
NC_000001.11:33 ------------------------------------------------------------
NC_000023.11:15 -----------------------------------------GAGGGTGGCA---------
NC_000004.12:c1 ------------------------------------------------------------

NC_000013.11:c3 TAAAGGGAAGAACTTTGGTACAGGAGATCAGCAGAGATTGACTAGAAAACCCCTTTCGTT
NC_000001.11:33 ------------------------------------------------------------
NC_000023.11:15 -------------------ACTGGGGGCCAGTTTGGGTTGG-------------------
NC_000004.12:c1 ------------------------------------------------------------

NC_000013.11:c3 TATATTATAGAGTAACGGTCCCAAGTTGAGAATACTGTATACATTTCTGGTTATTCCTAT
NC_000001.11:33 ------------------------------------------------------------
NC_000023.11:15 ------------------------------------------------------------
NC_000004.12:c1 ------------------------------------------------------------

NC_000013.11:c3 ATATTTGAAATGCTTTTTTCTACATATCCTGGTCCCAGAGAGAGTGCAGTACCTTCCAGT
NC_000001.11:33 -----------------CTTTCCATGTTCTATTC--------------------------
NC_000023.11:15 ------GAGATGCTCTTATTTCTCTGGTTTGCCC--------------------------
NC_000004.12:c1 ------------------------------------------------------------

NC_000013.11:c3 GGGATTAAAGCTAATTGCGTCCATCGCTTGTCAGCAGTGTGGGACAGAAATTTTATTGAC
NC_000001.11:33 ------------------------------------------------------------
NC_000023.11:15 ------------------------------------------------------------
NC_000004.12:c1 ------------------------------------------------------------

NC_000013.11:c3 CTATTTCCAGTTATAAACTCACATGATTTAAAGGGAGGAAGCTTTTATTAAATTAATAAC
NC_000001.11:33 ------------------------------------------------------------
NC_000023.11:15 ------------------------------------------------------------
NC_000004.12:c1 ------------------------------------------------------------

NC_000013.11:c3 TAACGAACAGCTGCAGTAGTTCAGTGCTTCTGTATGATTAGGATTTTCTCTTTTTTTCTC
NC_000001.11:33 ------------------------------------------------------------
NC_000023.11:15 ------------------------------------------------------------
NC_000004.12:c1 ------------------------------------------------------------

NC_000013.11:c3 TCATTTTAGCCACTCACGATGTCAGCTCGTGCACCTTCTACTAAGTTAGATTCCTTAACC
NC_000001.11:33 ------------------------------------------------------------
NC_000023.11:15 ------------------------------------------------------------
NC_000004.12:c1 ------------------------------------------------------------

NC_000013.11:c3 ACAATACAAGTCTTTGGCCCAAACCTGGGATATAAAAGCTACTTGCTATGATTTCTTTTT
NC_000001.11:33 ------------------------------------------------------------
NC_000023.11:15 ------------------------------------------------------------
```

```
NC_000004.12:c1 ------------------------------------------------------------

NC_000013.11:c3 TAGTCTCATAAAAAAAAAATGAGAGTTGAAGTGACTGACTGCTTTCTAGAAGCACTCTAT
NC_000001.11:33 ------------------------------------------------------------
NC_000023.11:15 -------------------------TTGAAGCTGCTG-----CCTCTGGGAATGCCCTGG
NC_000004.12:c1 ------------------------------------------------------------

NC_000013.11:c3 GAGTACAGTCTGTAATCCCACATCTCAATCTAACATTCAGAAAGCGGTGAAAACCAAAAA
NC_000001.11:33 ------------------------------------------------------------
NC_000023.11:15 GTGTG-------------------------------------------------------
NC_000004.12:c1 ------------------------------------------------------------

NC_000013.11:c3 CTTCTTAAGCTTGGCAACAAAATTCATTTGGTAGCAAAATATGACCTGAACTATTGAGAA
NC_000001.11:33 ------------------------------------------------------------
NC_000023.11:15 ------------------------------------------------------------
NC_000004.12:c1 ------------------------------------------------------------

NC_000013.11:c3 GCTGTATATAGTCTTTATCCCATGTGTTTTGCTGAAACTATATTAATATGTTTGATTATG
NC_000001.11:33 ------------------------------------------------------------
NC_000023.11:15 ------------------CCCATACGT--------GAGTACGTGTATACATGTGCTGCTT
NC_000004.12:c1 ------------------------------------------------------------

NC_000013.11:c3 GGGTGCTGCCCTTGGCCCCTGGTATGTGTTATATAATGGTTTATATACCCTGTAACACTT
NC_000001.11:33 ------------------------------------------------------------
NC_000023.11:15 GTGAGTTGGCATGGGCTTGTG---------------------------------------
NC_000004.12:c1 ------------------------------------------------------------

NC_000013.11:c3 AAAAATTGTATGAAATTCTTATTTCTAAAACACATCCAACCCTTGGGTTTTAGTTAAGGA
NC_000001.11:33 ------------------------------------------------------------
NC_000023.11:15 ------------------------------------TGACCCTTGTCATCCATCCAAGGG
NC_000004.12:c1 ------------------------------------------------------------

NC_000013.11:c3 ATTGTAAACCTGTATTCATATTTTTGGCAACACGCTTGTGCTATGCATGTGTATATTTAA
NC_000001.11:33 ------------------------------------------------------------
NC_000023.11:15 CCCACCACCCTG-----GTTCCCCCACCAAGGTGCCTGC---------------------
NC_000004.12:c1 ------------------------------------------------------------

NC_000013.11:c3 AAAAGTAACTTAAGACATTTTAGTTATTTCAGTAAATTCAGATGTGTTAGGATAGAAAAA
NC_000001.11:33 ------------------------------------------------------------
NC_000023.11:15 ------------------------------------------------------------
NC_000004.12:c1 ------------------------------------------------------------

NC_000013.11:c3 AATACGTGTATCATATTGTGTGTCTGTCATATCATGCATCTATCATATTGTATACATGCA
NC_000001.11:33 ------------------------------------------------------------
NC_000023.11:15 --CAACTGTGCCGTGTCACTTGCCACT---------------------------------
NC_000004.12:c1 ------------------------------------------------------------

NC_000013.11:c3 ATGATAGAGGTATGCACAGCATATTTTTAAGTGTAGCCGTATAGTGATCCAAACAAGAAA
NC_000001.11:33 -----------------------------------------------------------A
NC_000023.11:15 -----------------------------------------------------------G
NC_000004.12:c1 ------------------------------------------------------------

NC_000013.11:c3 TTAGTAGTTTCAGGGCTTAAGGTTAATTTATACATTAGACTCTAAAATTTAGATAAAACA
```

```
NC_000001.11:33 TTAGTAGT----------------------------------------------------
NC_000023.11:15 TGGGCAGTTGCTGGGCAGTAGCTGGACCCCAACTTGAATCTCTGG---------------
NC_000004.12:c1 ------------------------------------------------------------


NC_000013.11:c3 TGACAACTCGGCCGGGCGTGGTGGCTCACACCTGTAATCCCAGCACTTTGGGAGGCTGAG
NC_000001.11:33 ------------------------------------------------------------
NC_000023.11:15 ------------------------------------------------------------
NC_000004.12:c1 ------------------------------------------------------------


NC_000013.11:c3 GTGGGTGGATCACGAGGTCAGGAGTTCAAGACCAGCCTGGCCAAGATGGTGAAACCCCGT
NC_000001.11:33 ------------------------------------------------------------
NC_000023.11:15 -------------------------TCTAGAAAGGCCTGGCAAC----------------
NC_000004.12:c1 ------------------------------------------------------------


NC_000013.11:c3 CTCTACTAAAAAAAAAAAAAAAAAATACAAAAATTAACCGGGCGCGGTGGGAGGCGCCTG
NC_000001.11:33 ------------------------------------------------------------
NC_000023.11:15 ------------------------------------------------------------
NC_000004.12:c1 ------------------------------------------------------------


NC_000013.11:c3 TAAACCCAGCTACTCGGGAGGCTAAGGCAGGGAGAATTGCTTGAACCCCGGGGGTCAGAG
NC_000001.11:33 ---------------GGGTGGCTAAG----------------------------------
NC_000023.11:15 ------------CTTGAGAGGCTAGG----------------------------------
NC_000004.12:c1 ------------------------------------------------------------


NC_000013.11:c3 ATTGCAATGAGCTGAGATTACACCACTGCACTCCAGCCTGAGCGACAGAGCAAGACTCCA
NC_000001.11:33 ------------------------------------------------------------
NC_000023.11:15 ------------------------------------------------------------
NC_000004.12:c1 ------------------------------------------------------------


NC_000013.11:c3 TCTCAAAAAAACAAAAACAAAAACATGACAACTCGTAATGTAGCCCAAACTTACTTGGTT
NC_000001.11:33 ------------------------------------------------------------
NC_000023.11:15 ------------------------------------------------------------
NC_000004.12:c1 ------------------------------------------------------------


NC_000013.11:c3 CTGTTTTGCCCCCAGAAAATGAGAAGATGGTGTGATAATAGTCTAAAGTTGATACTGGAA
NC_000001.11:33 ------------------------------------------------------------
NC_000023.11:15 ------------------------------------------------------------
NC_000004.12:c1 ------------------------------------------------------------


NC_000013.11:c3 TATCTGACATCTAATCCCATCTCTTCTGCTGTGTGACCTTACACAAGTCACTGAATTTTT
NC_000001.11:33 ------------------------------------------------------------
NC_000023.11:15 ------------------------------------------------------------
NC_000004.12:c1 ------------------------------------------------------------


NC_000013.11:c3 AATAGTCTAAACTTGATATTGGAATATCTAGCATCTAATCCCATCTCTTCTGCTGTGTGA
NC_000001.11:33 ------------------------------------------------------------
NC_000023.11:15 ------------------------------------------------------------
NC_000004.12:c1 ------------------------------------------------------------


NC_000013.11:c3 CCTTATGCAAGTCACTGAATTTTTAAGGTCTCAGTTTACAGATCTATAAAATGAGAAGTT
NC_000001.11:33 ------------------------------------------------------------
NC_000023.11:15 ------------------------------------------------------------
NC_000004.12:c1 ------------------------------------------------------------
```

```
NC_000013.11:c3 TGGAATAGATGGATCTCTTAAGGAGAGTTCCTTATAGTTTCAACATTCCATGTGCAGGTA
NC_000001.11:33 ------------------------------------------------------------
NC_000023.11:15 ------------------------------------------------------------
NC_000004.12:c1 ------------------------------------------------------------

NC_000013.11:c3 TTTGCTCTTAGAAGTACCACCTGCTTTAAAAAGTTTTTCCTACTAAGGGTGTAATCCCAT
NC_000001.11:33 ------------------------------------------------------------
NC_000023.11:15 ------------------------------------------------------------
NC_000004.12:c1 ------------------------------------------------------------

NC_000013.11:c3 TTCATCTCAAACTGCTGTCTTGCTGATCTAAACTTGTCATTTTGTTCATAGGTAACACAT
NC_000001.11:33 ------------------------------------------------------------
NC_000023.11:15 ------------------------------------------------------------
NC_000004.12:c1 ------------------------------------------------------------

NC_000013.11:c3 TCATTCACGTGGTTCAAAATTCAAAAGGTACAAAAGGATACTCAGTCATTTTCTGCCTAG
NC_000001.11:33 ------------------------------------------------------------
NC_000023.11:15 ------------------------------------------------------------
NC_000004.12:c1 ------------------------------------------------------------

NC_000013.11:c3 GAAGCAACCAGTGCTACCAGTTTCTGGTATATTTTTCAGAAAAATTTTACACATGTAAGA
NC_000001.11:33 ------------------------------------------------------------
NC_000023.11:15 ------------------------------------------------------------
NC_000004.12:c1 ------------------------------------------------------------

NC_000013.11:c3 ACAAATAAATTACTATATATATTTTATTTTTCTTCCCTTTTTCTATGTAAATGGTACATA
NC_000001.11:33 ------------------------------------------------------------
NC_000023.11:15 ------------------------------------------------------------
NC_000004.12:c1 ------------------------------------------------------------

NC_000013.11:c3 CTATGCATAGTGATTATGTACCTTATTTGTTCACTTACCTTAAAAGTTGTTCTGTTATTA
NC_000001.11:33 ------------------------------------------------------------
NC_000023.11:15 ------------------------------------------------------------
NC_000004.12:c1 ------------------------------------------------------------

NC_000013.11:c3 TTTAAGGTATTTCCTTATTTTTATGGCTGCATAATCTTCCATTTTATAATGTACCATTAT
NC_000001.11:33 ------------------------------------------------------------
NC_000023.11:15 ------------------------------------------------------------
NC_000004.12:c1 ------------------------------------------------------------

NC_000013.11:c3 TTCATCTACCCTATCCTTTTGAACATTTAGGTGTTTTTTTTTTCCAGATCTTTATTAAAG
NC_000001.11:33 ------------------------------------------------------------
NC_000023.11:15 ------------------------------------------------------------
NC_000004.12:c1 ------------------------------------------------------------

NC_000013.11:c3 AGCATTGTGCAATGAAGACCTTTCTACGTACATCATTCTCCACATATGTGAATATATTTG
NC_000001.11:33 ------------------------------------------------------------
NC_000023.11:15 -----------------------------------------------------------G
NC_000004.12:c1 ------------------------------------------------------------

NC_000013.11:c3 TGCCCTAGCCAATGGTACGGCTGGGTCCTGTCTACTTTGACTTATGTGTTCTCCATGCCA
NC_000001.11:33 ------------------------------------------------------------
NC_000023.11:15 TGCTCTGGCAGAGGCGAGGTATTGGTC---------------------------------
```

```
NC_000004.12:c1 ------------------------------------------------------------

NC_000013.11:c3 GTATTGTATAGTGGAGTCAACACCCTGTTGACTCTAAAGTAAGCATATGAGATAAATCAC
NC_000001.11:33 ------------------------------------------------------------
NC_000023.11:15 ------------------------------------------------------------
NC_000004.12:c1 ------------------------------------------------------------

NC_000013.11:c3 ATCACCTGAATGGCTGTTTTTTCATGTGAGCCCTGAGAGCATGTATGTACATACATAATA
NC_000001.11:33 ------------------------------------------------------------
NC_000023.11:15 ------------------------------------------------------------
NC_000004.12:c1 ------------------------------------------------------------

NC_000013.11:c3 TGCATAAGTATGTATATATGAAGCAAGTAGATTATGTGTAGAGACTTTGTCCTAAACAAA
NC_000001.11:33 ------------------------------------------------------------
NC_000023.11:15 ----------------------------AGATCACTTGTGGAAACCCTGC----------
NC_000004.12:c1 ------------------------------------------------------------

NC_000013.11:c3 CAAACAAATAAATCTTTAAACACTAAAATCATGCCACTGAGCAAGATTTACAATGAGCAG
NC_000001.11:33 ------------------------------------------------------------
NC_000023.11:15 ------------------------------------------------------------
NC_000004.12:c1 ------------------------------------------------------------

NC_000013.11:c3 CATATGAAAACAGCAATGAGTGATGTAACAACAGGTTTGTTCTTTCCAGCTCATGCTCAT
NC_000001.11:33 --------------------------------------------TCCAGCCCACACT---
NC_000023.11:15 ---------------------------------------TCCCCTCTAGGCCTTGTG---
NC_000004.12:c1 ---------------------------------------------CCAGTTCACGCC---
                                                              *.** .* ...

NC_000013.11:c3 TCCAAGGCATGTACTCAATTAGTACTCTTAAGCTGATGTTTTTCTTTAAAATTTTTTGCC
NC_000001.11:33 ------------------------------------------------------------
NC_000023.11:15 ------------------------------------------------------------
NC_000004.12:c1 ------------------------------------------------------------

NC_000013.11:c3 AATAATATATGGTTGAATTTATGTAGCATGCAACTTCTAGATATTCCCACTTTACCAGTA
NC_000001.11:33 ------------------------------------------------------------
NC_000023.11:15 ------------------------------------------------------------
NC_000004.12:c1 ------------------------------------------------------------

NC_000013.11:c3 AGAAAACAATGTGTAAGGCTAGGCACAGTGGCTCACACCTCTAATCCCAGCACTTTGGGA
NC_000001.11:33 ------------------------------------------------------------
NC_000023.11:15 ------------------------------------------------------------
NC_000004.12:c1 ------------------------------------------------------------

NC_000013.11:c3 GGCTGAGGCAAGCAGATCACTTGAGGTCAGGAGTTCGAGGGTGTGGTGGCACATGCCTGT
NC_000001.11:33 ------------------------------------------------------------
NC_000023.11:15 ----GAGACAGGGGAA--------------------GAGGGAGTGG--------------
NC_000004.12:c1 ----GGAGC---------------------------------------------------

NC_000013.11:c3 AATCCCAGCTACTCAGGAGGATGAGGCATGAAAATGGCTTGATCCTGGGAGGTAGAGACT
NC_000001.11:33 ------------------------------------------------------------
NC_000023.11:15 -----------CTTGGGCAGGTCAGGC----------------CCTGGGA----------
NC_000004.12:c1 -------------------------------------------CCCGTGA----------

NC_000013.11:c3 GCAGGGACCTGATATTGTGCCTCTGCACTCCAGCCTGGACCACAGAGACCATACTGTCAG
```

```
NC_000001.11:33 ------------------------------------------------------------
NC_000023.11:15 ------------------------------------------------------------
NC_000004.12:c1 ------------------------------------------------------------

NC_000013.11:c3 AAAAACAAAACAGCAAAAACAAGGCCTAAAAGAAGTAACAATTTACTCAAATTTGCATTA
NC_000001.11:33 ------------------------------------------------------------
NC_000023.11:15 ------------------------------------------------------------
NC_000004.12:c1 ------------------------------------------------------------

NC_000013.11:c3 ACAAGGTGGCTGACCTGGTACTAAAACCTACATCCTCTCATTTCTAATCCAGGGCTTTTT
NC_000001.11:33 ------------------------------------------------------------
NC_000023.11:15 ------------------------------------------------------------
NC_000004.12:c1 ------------------------------------------------------------

NC_000013.11:c3 CCAGTATATTCTCGTAATTGGACATGTAGCTCTAGCAGATCTGAGAATGTCCAGTAATTA
NC_000001.11:33 ------------------------------------------------------------
NC_000023.11:15 ------------------------------------------------------------
NC_000004.12:c1 ------------------------------------------------------------

NC_000013.11:c3 GGAACTGCTTTAGGTACAGTCACTCTTCCTAGACTCTAGAGCAACCTAAAGTTCAGAAAG
NC_000001.11:33 ------------------------------------------------------------
NC_000023.11:15 ------------------------------------------------------------
NC_000004.12:c1 ------------------------------------------------------------

NC_000013.11:c3 CAGCAGAACGTTTTTCCAGTGACAAAATAACGTTCTATAAGTACTTGTTGAAGTAATCAC
NC_000001.11:33 ------------------------------------------------------------
NC_000023.11:15 --------------------------------------AACTGCTTGTAGAA--------
NC_000004.12:c1 ------------------------------------------------------------

NC_000013.11:c3 ACAACATGTTGATTAGCAATCAGGAAGCCTCCAAATCAAAAAATTAAGTGCAGATAAAGA
NC_000001.11:33 ------------------------------------------------------------
NC_000023.11:15 ------------------------------------------------------------
NC_000004.12:c1 ------------------------------------------------------------

NC_000013.11:c3 AACACTCTTTCCTAATTGGGACATAGTTGTCACTTCAGGGTGAATTGATCAGTCATAAGG
NC_000001.11:33 ------------------------------------------------------------
NC_000023.11:15 ------------------------------------------------------------
NC_000004.12:c1 ------------------------------------------------------------

NC_000013.11:c3 ACTAGAAGGAATTTAGGATTATTTGGACATAGGTAGTCTTACTTTCCCCAGAGTTCATGC
NC_000001.11:33 ------------------------------------------------------------
NC_000023.11:15 ------------------------------------------------------------
NC_000004.12:c1 ------------------------------------------------------------

NC_000013.11:c3 CATCATTTAATTTAGCCAGGAAATTTAAAATAATTGTCAACAGAAAAGGAGAAGTGGTAG
NC_000001.11:33 ------------------------------------------GGAGAGGGGGAGTG----
NC_000023.11:15 ------------------------------------------GGAGCAGGGAGGGG----
NC_000004.12:c1 ------------------------------------------------GGGAAGCG----
                                                                *.*..* *

NC_000013.11:c3 AAACAATAGCAAAAGAAAAATGGAAAGTTGGAGGATATGCTTTAGTAACTACATTCACAT
NC_000001.11:33 ------------------------------------------------------------
NC_000023.11:15 ------------------------------------------------------------
NC_000004.12:c1 ------------------------------------------------------------
```

```
NC_000013.11:c3 AGAAGGTTAGATATGCTTTAGTAACTGTATTCGCTTGAAGCTGGCATTGCAGCACATGAC
NC_000001.11:33 ------------------------------------------------------------
NC_000023.11:15 ------------------------------------------------------------
NC_000004.12:c1 ------------------------------------------------------------

NC_000013.11:c3 TGTCTGCTTTAAGGCCTTAGCTACCTGCTGACAATGCTAGTCCCCTTTTCTTTACATAAC
NC_000001.11:33 ------------------------------------------------------------
NC_000023.11:15 ------------------------------------------------------------
NC_000004.12:c1 ------------------------------------------------------------

NC_000013.11:c3 TGTCTTCTCATCCTTGGAGTCTTAGCTCGAGTGTCTTGTGTCAAGCTGTCCACCCTATTT
NC_000001.11:33 ------------------------------------------------------------
NC_000023.11:15 ------------------------------------------------------------
NC_000004.12:c1 ------------------------------------------------------------

NC_000013.11:c3 GAGGAGCCCTCTGTCCTCTACCCCTACTCTAATTCTTTCTCACCACCTGTTTGTTTTCCT
NC_000001.11:33 ------------------------------------------CCATCTGTTT--------
NC_000023.11:15 ------------------------------------------CTGTCTGTCTG-------
NC_000004.12:c1 ------------------------------------------------------------

NC_000013.11:c3 CATAGCATATGTATGTGTGTGTATATATGTGTATGTGTGCGTATGTATATATGTGTATGT
NC_000001.11:33 ------------------------------------------------------------
NC_000023.11:15 ------------------------------------------------------------
NC_000004.12:c1 ------------------------------------------------------------

NC_000013.11:c3 GTATATGTATATATACATGTATGTGTGTGTATATATGTATAACACACACACATACACATA
NC_000001.11:33 ------------------------------------------------------------
NC_000023.11:15 ------------------------------------------------------------
NC_000004.12:c1 ------------------------------------------------------------

NC_000013.11:c3 TATTTTTGGCCCCATAGTAGGTTGTGAGATCTGTAAGAGCAAGGATTACATCTGTTTGGT
NC_000001.11:33 ------------------------------------------------------------
NC_000023.11:15 ------------------------------------GAGAAAAG---------GCCTGGC
NC_000004.12:c1 ------------------------------------------------------------

NC_000013.11:c3 TCACCATCATCTATATAGTGCCTAGTACATAGTGCATATTTGTTGATGAGTTATCTTGGT
NC_000001.11:33 ------------------------------------------------------------
NC_000023.11:15 CTGCCATGAACTA-----------------------------------------------
NC_000004.12:c1 ------------------------------------------------------------

NC_000013.11:c3 GGCTCCCAATTTTCAATTTACATGGCTGACCAAACCTACTTTTAAAACAGAATGTATTAA
NC_000001.11:33 ------------------------------------------------------------
NC_000023.11:15 ------------------------------------------------------------
NC_000004.12:c1 ------------------------------------------------------------

NC_000013.11:c3 ATAGCCTATTTCATTCATGTTTGTTAGTTCAGACGTATGCATAATTGATACTCTTTTCTT
NC_000001.11:33 ------------------------------------------------------------
NC_000023.11:15 ------------------------------------------------------------
NC_000004.12:c1 ------------------------------------------------------------

NC_000013.11:c3 ATTGAGAAAGCTATTAACTTTTTCATCAATGGCTTTTTTTTTTTTTTACATTTACTGTTT
NC_000001.11:33 ------------------------------------------------------------
NC_000023.11:15 ------------------------------------------------------------
```

```
NC_000004.12:c1 ------------------------------------------------------------


NC_000013.11:c3 TTTTAATAAAGGATGTTTTAAAGGGTACAGATGAACAGCCAGATGAAGAGATGCACAGAG
NC_000001.11:33 ------------------------------------------------------------
NC_000023.11:15 ----------------TCTGAAGGGT---GAGGGATTACCCTGTGTGGAGATG-------
NC_000004.12:c1 ------------------------------------------------------------


NC_000013.11:c3 CCAAAAAGCTTGTATTTAATGTGCAAAGGACTGCCATAAATGCAGGGAGAATACAACAGT
NC_000001.11:33 ------------------------------------------------------------
NC_000023.11:15 ------------------------------------------------------------
NC_000004.12:c1 ------------------------------------------------------------


NC_000013.11:c3 GAATAATGCATAGTCCTTATTCCTAAAGAGAAGTAGAAACGTTTGTAAACAAATAGTTTT
NC_000001.11:33 ----------------TTATTCCTA-----------------------------------
NC_000023.11:15 ----------------TTGACCTTAA----------------------------------
NC_000004.12:c1 ------------------------------------------------------------


NC_000013.11:c3 CATACAAGGCGGGATGAAATAAGTGCTAAATAGAGTTGTACAGGCAATGTTTTGTAGAAG
NC_000001.11:33 ------------------------------------------------------------
NC_000023.11:15 ------------------------------------------------------------
NC_000004.12:c1 ------------------------------------------------------------


NC_000013.11:c3 CTATGGAGGAAAAAATGATTAATTTTAATTATAGGAAGACCCTCACAAAGGTGAATTTTT
NC_000001.11:33 ------------------------------------------------------------
NC_000023.11:15 ------------------------------------------------------------
NC_000004.12:c1 ------------------------------------------------------------


NC_000013.11:c3 ACTTCAGTCTTCAAAGAAAAATAGAGCACAAGTCCCTATAGATGAGGAATAGGGATGAAT
NC_000001.11:33 ------------------------------------------------------------
NC_000023.11:15 ------------------------------------------------------------
NC_000004.12:c1 ------------------------------------------------------------


NC_000013.11:c3 AAATGTTTAGCTTCACTTTTTAAAAAGTCCTTAAAATAGCTGTAATCCCAGCACTTTGGA
NC_000001.11:33 ------------------------------------------------------------
NC_000023.11:15 ------------------------------------------------------------
NC_000004.12:c1 ------------------------------------------------------------


NC_000013.11:c3 AGGCCGAGGTGGGCAGATCACGAGGTCAGGAGTTCGAGACCAGCCTGGCCAGCATGGTGA
NC_000001.11:33 ------------------------------------------------------------
NC_000023.11:15 ------------------------------------------------------------
NC_000004.12:c1 ------------------------------------------------------------


NC_000013.11:c3 AACCCCATCTCTACTAAAAAAACAAAAAATTAGCTGGGCACGTTGGCGCATGCCTGTAGT
NC_000001.11:33 ------------------------------------------------------------
NC_000023.11:15 ------------------------------------------------------------
NC_000004.12:c1 ------------------------------------------------------------


NC_000013.11:c3 CCCAGCTACTCAGGAGGCTGAGGCAGGAGCCTCAGATCCAAGATCGCACCACTGCACTCC
NC_000001.11:33 ------------------------------------------------------------
NC_000023.11:15 ------------------------------------------------------------
NC_000004.12:c1 ------------------------------------------------------------


NC_000013.11:c3 AGCCTGGGCAATAAAGCGAGACTGCGTCTCAAAAAAAAAGCAGTATTTACCACAAAGGAC
```

```
NC_000001.11:33 ------------------------------------------------------------
NC_000023.11:15 ------------------------------------------------------------
NC_000004.12:c1 ------------------------------------------------------------


NC_000013.11:c3 TCATAACACATTGTTACAAAGCAATCTTTTTAAAAAATGTTAGTTAAAAATAAAATGACT
NC_000001.11:33 ------------------------------------------------------------
NC_000023.11:15 ------------------------------------------------------------
NC_000004.12:c1 ------------------------------------------------------------


NC_000013.11:c3 CCAGCCATAAACATTATACTTCTGCAACTGAGGATGACCGTAAAATCACTTAACTGTTTT
NC_000001.11:33 ------------------------------------------------------------
NC_000023.11:15 ------------------------------------------------------------
NC_000004.12:c1 ------------------------------------------------------------


NC_000013.11:c3 GACTAGGTTGACAGCTAAATTGAGGGTCAGTTTAATGATTGAATTGTAACTGAGTGCTAA
NC_000001.11:33 ------------------------------------------------------------
NC_000023.11:15 ------------------------------------------------------------
NC_000004.12:c1 ------------------------------------------------------------


NC_000013.11:c3 TTGGAGCTGGTAATTGTTTAGTAAATGTATCAGTTAAGATACATTATTCATTAGATAAAA
NC_000001.11:33 ------------------------------------------------------------
NC_000023.11:15 ------------------------------------------------------------
NC_000004.12:c1 ------------------------------------------------------------


NC_000013.11:c3 ACTAATGAATAATGAAAACAGATTCATTTTTTAAAAAGATTTTTAAGCCACTGGTGGTCT
NC_000001.11:33 ------------------------------------------------------------
NC_000023.11:15 ------------------------------------------------------------
NC_000004.12:c1 ------------------------------------------------------------


NC_000013.11:c3 TTAATATATGGTTCTCACCACATAAGTTAAAATGGGGACTGTTTTTTGGTGGTCTAATCT
NC_000001.11:33 ------------------------------------------------------------
NC_000023.11:15 ------------------------------------------------------------
NC_000004.12:c1 ------------------------------------------------------------


NC_000013.11:c3 GATTTTGTAAGTTTATTTTTGCTTCCCTACCTCTGTTTTGCTAAGCAATATAGACCTTTT
NC_000001.11:33 ------------------------------------------------------------
NC_000023.11:15 ------------------------------------------------------------
NC_000004.12:c1 ------------------------------------------------------------


NC_000013.11:c3 CTTCCCACTTTTTTTTTTTTGTTTTAGTGTAGGCATTAGATTTACAGATCTCATTTAAAC
NC_000001.11:33 ------------------------------------------------------------
NC_000023.11:15 ------------------------------------------------------------
NC_000004.12:c1 ------------------------------------------------------------


NC_000013.11:c3 TTGGCCATATATTAATCTCAGTTTTGTTACTTCATACAAAATATTTAACCTCCCTGAGGC
NC_000001.11:33 ------------------------------------------------------------
NC_000023.11:15 ------------------------------------------------------------
NC_000004.12:c1 ------------------------------------------------------------


NC_000013.11:c3 TCAGTTTCACTGATAAAATGAAAACATCTACTAATCCCACTTCTGTTATTTAACTAATTT
NC_000001.11:33 ------------------------------------------------------------
NC_000023.11:15 ------------------------------------------------------------
NC_000004.12:c1 ------------------------------------------------------------
```

```
NC_000013.11:c3 TGGCCTCAGGCAAATTATTTAACCTCTCTGGGCTTCAGTTTTTCCATGTGTGAAATGAAA
NC_000001.11:33 ------------------------------------------------------------
NC_000023.11:15 ------------------------------------------------------------
NC_000004.12:c1 ------------------------------------------------------------

NC_000013.11:c3 TTCTCTTAAATGAGGTAAGACATAGAACAATGTCTGGCAGTTAATATGCCCTCACAGCAG
NC_000001.11:33 ------------------------------------------------------------
NC_000023.11:15 ------------------------------------------------------------
NC_000004.12:c1 ------------------------------------------------------------

NC_000013.11:c3 ATGTTGGTTTTCCCTCACCCTTCCACCTCATCTAAACCCTGCATGTCCTTTACAGATTGC
NC_000001.11:33 ------------------------------------------------------------
NC_000023.11:15 ------------------------------------------------------------
NC_000004.12:c1 ------------------------------------------------------------

NC_000013.11:c3 TTTAAATTCTGTTTTCTCTTTCCTTTTCCAACTCCTAGCACTCTCTTCTAGAGTTGTTGC
NC_000001.11:33 ------------------------------------------------------------
NC_000023.11:15 ------------------------------------------------------------
NC_000004.12:c1 ------------------------------------------------------------

NC_000013.11:c3 ACTTACCATATATACCATGGATTCTTAAATGGGTATTATTACAATTGACCCTTGAGCAAC
NC_000001.11:33 ------------------------------------------------------------
NC_000023.11:15 ------------------------------------------------------------
NC_000004.12:c1 ------------------------------------------------------------

NC_000013.11:c3 ACAGGGGTTAGGGACCACCCTCTCCTTCCCCATGCTTTTGACTGCCCAAAAAACGTAGCT
NC_000001.11:33 ------------------------------------------------------------
NC_000023.11:15 ------------------------------------------------------------
NC_000004.12:c1 ------------------------------------------------------------

NC_000013.11:c3 GCTAATAGCCCACTGATGACTGGAAGTTTACTGATAGCATATATTGTTGACTAACGTATT
NC_000001.11:33 ------------------------------------------------------------
NC_000023.11:15 ------GGCTCAGAGGTGGCTGGAGAT---------------------------------
NC_000004.12:c1 ------------------------------------------------------------

NC_000013.11:c3 TTGTATGTCATATGTGTTATATACTGTATTCTTACAATAAAGTAAGTTAGAGAAAAAAAG
NC_000001.11:33 ------------------------------------------------------------
NC_000023.11:15 ------------------------------------------------------------
NC_000004.12:c1 ------------------------------------------------------------

NC_000013.11:c3 TTACTAAGAAAATCATAAGGAAAAGTAAGTATATTTACTAATCATTAAGTGGAAATGGAT
NC_000001.11:33 ------------------------------------------------------------
NC_000023.11:15 ---------------------------------------------------GGAAGGAAC
NC_000004.12:c1 ------------------------------------------------------------

NC_000013.11:c3 CATCATAAAGATCTTCGTCCTTGTCACCTTCACATTGAGTAGACTGAAGAGGAGCTGAGT
NC_000001.11:33 ------------------------------------------------------------
NC_000023.11:15 TGCCATGAAG--------------------------------------------------
NC_000004.12:c1 ------------------------------------------------------------

NC_000013.11:c3 GGATAAAGAAAATGTTTATGTACATCGTGGAATACTATTCATCCATACAAAGGAACGAAA
NC_000001.11:33 ------------------------------------------------------------
NC_000023.11:15 ------------------------------------------------------------
```

```
NC_000004.12:c1 ------------------------------------------------------------

NC_000013.11:c3 TAATATCTTTTGCAGCAATTTGGATGGAGCTGGAGGCCAGTATTCTAAGTGAAGTAGCTC
NC_000001.11:33 ------------------------------------------------------------
NC_000023.11:15 ------------------------------------------------------------
NC_000004.12:c1 ------------------------------------------------------------

NC_000013.11:c3 AGGAATGGGAAACCAAAAATGGTATGTTCTCACTTAAAAGTGGGAGCTAAGCTATAAAAA
NC_000001.11:33 ------------------------------------------------------------
NC_000023.11:15 --------------GAAGATGGTAAGCTC-------------------------------
NC_000004.12:c1 ------------------------------------------------------------

NC_000013.11:c3 CACAGAGGCATAAGAATCATACAGTGGACTTTGGGGATTCGGGTTGGGGAAGAAGGTTGG
NC_000001.11:33 ------------------------------------------------------------
NC_000023.11:15 ------------------------------------------------------------
NC_000004.12:c1 ------------------------------------------------------------

NC_000013.11:c3 ATGGGGGTTAAAAGACTACATGTTGGATACAGTGTACACTGCTCAGGTGACAGTTGCACT
NC_000001.11:33 ------------------------------------------------------------
NC_000023.11:15 ------------------------------------------------------------
NC_000004.12:c1 ------------------------------------------------------------

NC_000013.11:c3 AAAATCTCAGAAATCACCACTAAAGAACTTATCCATGTAACCAAAAACCACCTGCACCCC
NC_000001.11:33 ------------------------------------------------------------
NC_000023.11:15 ------------------------------------------------------------
NC_000004.12:c1 ------------------------------------------------------------

NC_000013.11:c3 CCAAAACTATTGAAATAAACAGGACGGGTTGTTCTTGCTGTCTTAAGCCTCATGGCAGAG
NC_000001.11:33 ------------------------------------------------------------
NC_000023.11:15 ------------------------------------------------------------
NC_000004.12:c1 ------------------------------------------------------------

NC_000013.11:c3 GCTTAAGAGCAGGAGCATGTAGGGAGGGAGGGAGGAGAGGCAGGCACACTCGGTGTAACT
NC_000001.11:33 ------------------------------------------------------------
NC_000023.11:15 -CCTATGAGTGGAGGTATGCAAGCAGA----------AGGCAGGCA--------------
NC_000004.12:c1 ------------------------------------------------------------

NC_000013.11:c3 TTTATTGAAAAAAATCAAATCCATTTTATAAGTGGACCCAGGCAGTTCAAACCTGCATTG
NC_000001.11:33 ------------------------------------------------------------
NC_000023.11:15 ------------------------------------------------------------
NC_000004.12:c1 ------------------------------------------------------------

NC_000013.11:c3 TTCAGGGGTCAACTATATATTGGGAACTTGTTAAAACTGCAGCTGCTTGGGTTTCATGCA
NC_000001.11:33 ------------------------------------------------------------
NC_000023.11:15 ------------------------------------------------------------
NC_000004.12:c1 ------------------------------------------------------------

NC_000013.11:c3 TGATTCCATGAGTTTGGCATGGGGCCTGGGCATCTATATTTTTTTCTCCCTCAGATTTTC
NC_000001.11:33 ------------------------------------------------------------
NC_000023.11:15 ------------------------------------------------------------
NC_000004.12:c1 ------------------------------------------------------------

NC_000013.11:c3 TTACGCTTACCAATGATCACGGCCTTTCACCTATTTGTTTTTTGTTTGTTTGTTTTTTTG
```

```
NC_000001.11:33 ------------------------------------------------------------
NC_000023.11:15 ------------------------------------------------------------
NC_000004.12:c1 ------------------------------------------------------------

NC_000013.11:c3 AGACGGAGTCTGGCTCTGTCACCCAGGCTGGAGTGCAGTGGCGCAATGGCTCACTGCAAG
NC_000001.11:33 ------------------------------------------------------------
NC_000023.11:15 ------------------------------------------------------------
NC_000004.12:c1 ------------------------------------------------------------

NC_000013.11:c3 CTCCGCCTTCCGGGTTCACGCCATTCTCCTGCCTCAGCGTCCTGAGTAGCTGGGACTACA
NC_000001.11:33 ------------------------------------------------------------
NC_000023.11:15 ------------------------------------------------------------
NC_000004.12:c1 ------------------------------------------------------------

NC_000013.11:c3 GGCTCCCACCACTACGCCGGGCTAATTTTTTGTATTTTTAGTAGAGACGGGGTTTCACAC
NC_000001.11:33 ------------------------------------------------------------
NC_000023.11:15 -------ACCACTAAGCTGG----------------------------------------
NC_000004.12:c1 ------------------------------------------------------------

NC_000013.11:c3 CGTGTTAGCCAGGATGGTCTTGATCTCCTGACCTCGTGATCCACCCGCCTTGGCCTCCCC
NC_000001.11:33 ------------------------------------------------------------
NC_000023.11:15 ------------------------------------------------------------
NC_000004.12:c1 ------------------------------------------------------------

NC_000013.11:c3 AAGTGCTGGGATTACAGGCGTGAGCCGCCGCGCCCGGCCTCACCTATTAATCGTGTTTTT
NC_000001.11:33 ------------------------------------------------------------
NC_000023.11:15 ------------------------------------------------------------
NC_000004.12:c1 ------------------------------------------------------------

NC_000013.11:c3 TATAGTTTCTGTTTAAAACCAGGTGTTTTAGTTTAAACCTCAAATATACTATAAACTCCA
NC_000001.11:33 ------------------------------------------------------------
NC_000023.11:15 ------------------------------------------------------------
NC_000004.12:c1 ------------------------------------------------------------

NC_000013.11:c3 TGAGGGCACAAAATTAAACTTAGTTTAGAGGTTGATAAGTAATGCCCCACCTTTTTTATC
NC_000001.11:33 ------------------------------------------------------------
NC_000023.11:15 ------------------------------------------------------------
NC_000004.12:c1 ------------------------------------------------------------

NC_000013.11:c3 TGCCTGTGTTTGGATCCAGTTAAATCACATGAAGATTGAAATTAAGTTTACTGAAAAAAG
NC_000001.11:33 ------------------------------------------------------------
NC_000023.11:15 ------------------------------------------------------------
NC_000004.12:c1 ------------------------------------------------------------

NC_000013.11:c3 TTGTTTCAAAAGGTAGCTGAGATTTGGAGCAACAACGAGCTGCAAAGAACTAGGCAGATT
NC_000001.11:33 ------------------------------------------------------------
NC_000023.11:15 ------------------------------------------------------------
NC_000004.12:c1 ------------------------------------------------------------

NC_000013.11:c3 TAAAATTCGAGTTTTTCTATCAACAAGAATAGAGCTTGTCCCACTACATTTACTGTGTAA
NC_000001.11:33 ------------------------------------------------------------
NC_000023.11:15 ------------------------------------------------------------
NC_000004.12:c1 ------------------------------------------------------------
```

```
NC_000013.11:c3 GAGAGCAGGCCAAAACAAATGACCCTCTTCAGTCAGCCAAATCCCAAATGGAAAGAAGAG
NC_000001.11:33 ------------------------------------------------------------
NC_000023.11:15 -----------------------------------------TTCTAACAGGACAGAGGG-
NC_000004.12:c1 ------------------------------------------------------------

NC_000013.11:c3 AGGAAGTTGGCCGAGCCTCAGAATTTTTTCTCCCAAACTTGCTGTCAGATCAGTTAGCCC
NC_000001.11:33 ------------------------------------------------------------
NC_000023.11:15 ------------------------------------------------------------
NC_000004.12:c1 ------------------------------------------------------------

NC_000013.11:c3 CTCCTATCCTAGAAAGGAGTTAAGAGTAGAGGTCAGCCATGAATATTACGGTAAGGAAGT
NC_000001.11:33 ------------------------------------------------------------
NC_000023.11:15 ------------------------------------------------------------
NC_000004.12:c1 ------------------------------------------------------------

NC_000013.11:c3 TTTATGTGGAGTATGAAAGAGCTTTCCTCATGCCCTCAACAGTACTCAATAGGTATTAAT
NC_000001.11:33 ------------------------------------------------------------
NC_000023.11:15 ------------------------------------------------------------
NC_000004.12:c1 ------------------------------------------------------------

NC_000013.11:c3 GGATTTTTTTTTTAGTACTTTGCATCGGGTTGCTGTGATTTTTAGTACCTAAATAAGTTA
NC_000001.11:33 ------------------------------------------------------------
NC_000023.11:15 ------------------------------------------------------------
NC_000004.12:c1 ------------------------------------------------------------

NC_000013.11:c3 TGAAGTTACAACTTCAGTTAAATCAGTATGTATTATTGTATTAGTTATATTTTGTTATGT
NC_000001.11:33 ------------------------------------------------------------
NC_000023.11:15 ------------------------------------------------------------
NC_000004.12:c1 ------------------------------------------------------------

NC_000013.11:c3 AACAAATTATTCCCCAAACAGCTTAAAACAACAGACATTTGTCATTTCATACATTGTGTA
NC_000001.11:33 ------------------------------------------------------------
NC_000023.11:15 ------------------------------------------------------------
NC_000004.12:c1 ------------------------------------------------------------

NC_000013.11:c3 TGAAATGATACATGGTCAGGAATCTGGGAGTGGTTTAGCTGAGTGGCTCCAGTTCGGAGC
NC_000001.11:33 ------------------------------------------------------------
NC_000023.11:15 ------------------------------------------------------------
NC_000004.12:c1 ------------------------------------------------------------

NC_000013.11:c3 CTCTTATGTGGTTGCAGTCATCTAAAGGCTGCTTGAGTGTCTTCACAAAATGACACCTGG
NC_000001.11:33 ------------------------------------------------------------
NC_000023.11:15 ------------------------------------------------------------
NC_000004.12:c1 ------------------------------------------------------------

NC_000013.11:c3 CTTTTCCCCCAGAGCACGTGATCTGAGAGAGAGGCAGAAACCACAGTGTCTTTCACAACC
NC_000001.11:33 ------------------------------------------------------------
NC_000023.11:15 ------------------------------------------------------------
NC_000004.12:c1 ------------------------------------------------------------

NC_000013.11:c3 TAACCTCGGAAGTGACCTACTGTCACTGTGCTGTATTCTATGGGTTACACAGACCTACCT
NC_000001.11:33 ------------------------------------------------------------
NC_000023.11:15 ------------------------------------------------------------
```

```
NC_000004.12:c1 ------------------------------------------------------------

NC_000013.11:c3 ACACAAAGGTATGAATACCAAGAGGTGAAGGATTCATTGGAGGCCATCTTGGGGGCCGGC
NC_000001.11:33 ------------------------------------------------------------
NC_000023.11:15 ------------------------------------------------------------
NC_000004.12:c1 ------------------------------------------------------------

NC_000013.11:c3 TACCACAATTTTATAACATGTTAATTTCACTGTATACCTCACAGAACAAGTAATTCAGTA
NC_000001.11:33 ------------------------------------------------------------
NC_000023.11:15 ------------------------------------------------------------
NC_000004.12:c1 ------------------------------------------------------------

NC_000013.11:c3 ATGTCTAATATCCCAATAAAATCGTTTCATGGTAATTTTGTGAAATAATTGTATTTTTAT
NC_000001.11:33 ------------------------------------------------------------
NC_000023.11:15 ------------------------------------------------------------
NC_000004.12:c1 ------------------------------------------------------------

NC_000013.11:c3 ATATATTATAAAATTTGCTAGCTAGAGATTAGGCTGTGAGAATATGTCCAGAGGATGATT
NC_000001.11:33 ------------------------------------------------------------
NC_000023.11:15 ------------------------------------------------------------
NC_000004.12:c1 ------------------------------------------------------------

NC_000013.11:c3 CTCTACTACCAGCCCCTTCCTGTCTAGCTCTATTTCTGTATCAGTAATTTATATAGCATG
NC_000001.11:33 ------------------------------------------------------------
NC_000023.11:15 ------------------------------------------------------------
NC_000004.12:c1 ------------------------------------------------------------

NC_000013.11:c3 ATCAAGAACTACTCTGGTTACATGTTCTTCTAGATAGTCTGTGAAAGTTCAGGTGATTTA
NC_000001.11:33 ------------------------------------------------------------
NC_000023.11:15 ------------------------------------------------------------
NC_000004.12:c1 ------------------------------------------------------------

NC_000013.11:c3 TAGACAGACTTACCATAAGGTGATTGTATTAGTCTATTTTCACACTGCTGTAAAGAACTA
NC_000001.11:33 ------------------------------------------------------------
NC_000023.11:15 ------------------------------------------------------------
NC_000004.12:c1 ------------------------------------------------------------

NC_000013.11:c3 CCTGAGACTGGGTAATTTACAAAGAAAAGAGGTTTAATTGACTCACATTTCTGCATGGCT
NC_000001.11:33 ------------------------------------------------------------
NC_000023.11:15 ------------------------------------------------------ATGGAT
NC_000004.12:c1 ------------------------------------------------------------

NC_000013.11:c3 GGGGAGGCCTCAGGAAACTTAAAATCATGGCAGAAGGCAAAGGGGAAGCAAGGCACATCT
NC_000001.11:33 ------------------------------------------------------------
NC_000023.11:15 GAGGTGGCCTTAAGA---------------------------------------------
NC_000004.12:c1 ------------------------------------------------------------

NC_000013.11:c3 TACATGGTGGCAGAAGAGAGCGAGCAAGTAGGAGGAAGTCCCATGCGTTTAAACCATCGG
NC_000001.11:33 ------------------------------------------------------------
NC_000023.11:15 ------------------------------------------------------------
NC_000004.12:c1 ------------------------------------------------------------

NC_000013.11:c3 AGAACTCTTTCACAAGAACAGCAAGGGGGAAGGCCGCCCCCATGATCCAGTCCTTTCGCA
```

```
NC_000001.11:33 ------------------------------------------------------------
NC_000023.11:15 ------------------------------------------------------------
NC_000004.12:c1 ------------------------------------------------------------


NC_000013.11:c3 CCAGGCTTCTCCTTTGACACGTGGGGATTACAATTCAAGATGAGATTTGGGTGGAGACAC
NC_000001.11:33 ------------------------------------------------------------
NC_000023.11:15 ------------------------------------------------------------
NC_000004.12:c1 ------------------------------------------------------------


NC_000013.11:c3 AGAGCCAAACCATATGAGTGATAATAGCTACAATTTTGATTTTAGTATGAGTTAATAATT
NC_000001.11:33 ------------------------------------------------------------
NC_000023.11:15 ------------------------------------------------------------
NC_000004.12:c1 ------------------------------------------------------------


NC_000013.11:c3 ATAGTCTCCTGGGAGGTGCCTCATGCCCATCTACTGATTTATTCAAGTGAGGGTTTCTTT
NC_000001.11:33 ------------------------------------------------------------
NC_000023.11:15 ------------------------------------------------------------
NC_000004.12:c1 ------------------------------------------------------------


NC_000013.11:c3 TGCAGTGGTAATAATTAGGATAAGGTGGACTAGATGGCTAGTGAAGGTCATTGAAATAGT
NC_000001.11:33 ------------------------------------------------------------
NC_000023.11:15 ------------------------------------------------------------
NC_000004.12:c1 ------------------------------------------------------------


NC_000013.11:c3 CCATAGGCCCAAAGAGCCTAAAAACCATCACTGTAAGCAAGTGGCAGTGCTTTGATTAAA
NC_000001.11:33 ------------------------------------------------------------
NC_000023.11:15 ------------------------------------------------------------
NC_000004.12:c1 ------------------------------------------------------------


NC_000013.11:c3 ATTCTAATGCCAAAGCTTAAAAGAAGTTCAATAAGTGAAATAAAATGATTATAATTTATT
NC_000001.11:33 ------------------------------------------------------------
NC_000023.11:15 ------------------------------------------------------------
NC_000004.12:c1 ------------------------------------------------------------


NC_000013.11:c3 TCTTAAAATATGCTACGTAATCTTACAGTGTAAGTGAGGAAACTTGAAATGTTCATTGAA
NC_000001.11:33 ------------------------------------------------------------
NC_000023.11:15 ------------------------------------------------------------
NC_000004.12:c1 ------------------------------------------------------------


NC_000013.11:c3 TTCAGGAGTGCAGAATTCATTTGTCTTCTATAGCTATTCAACACAACAGAAAAGATAAAT
NC_000001.11:33 ------------------------------------------------------------
NC_000023.11:15 ------------------------------------------------------------
NC_000004.12:c1 ------------------------------------------------------------


NC_000013.11:c3 CACTTGAAGATTGTGGCAGAAGATGTTTTAACATAACCTGACAATGCCTAAACATTCTTT
NC_000001.11:33 ------------------------------------------------------------
NC_000023.11:15 ------------------------------------------------------------
NC_000004.12:c1 ------------------------------------------------------------


NC_000013.11:c3 GGAATTCTAACCATAAGCATTGTTAGGTCAGTTTGCATTTTAAGTTGCTTTAAGTACTTA
NC_000001.11:33 ------------------------------------------------------------
NC_000023.11:15 ------------------------------------------------------------
NC_000004.12:c1 ------------------------------------------------------------
```

```
NC_000013.11:c3 CAGATGAGTGACCAGTTGTAGAAGCCCAAATATTTGTATAATCAATTTTCTCATCCAGAT
NC_000001.11:33 ------------------------------------------------------------
NC_000023.11:15 ------------------------------------------------------------
NC_000004.12:c1 ------------------------------------------------------------

NC_000013.11:c3 GTAAGCTGTTTATTACATAGACATAAGCATGTAGAGCATATTATTATCAGACCCAGTGAA
NC_000001.11:33 ------------------------------------------------------------
NC_000023.11:15 ------------------------------------------------------------
NC_000004.12:c1 ------------------------------------------------------------

NC_000013.11:c3 CTCATTTGGATGTCATAATTTCCAGTCATGTAGGCATATTTGACAACAACTTTCACCCTC
NC_000001.11:33 ------------------------------------------------------------
NC_000023.11:15 ------------------------------------------------------------
NC_000004.12:c1 ------------------------------------------------------------

NC_000013.11:c3 AAGTGTTCGGAGGCACTATTTTGCTAATCAGGGAAGAAATTTTAGGAAATTCATTTTGCT
NC_000001.11:33 ------------------------------------------------------------
NC_000023.11:15 ------------------------------------------------------------
NC_000004.12:c1 ------------------------------------------------------------

NC_000013.11:c3 TTCCAAATGTAATATGATCAATAAAATGTTTGGTTTGGTTATTGATCAGCTTTAAGACAT
NC_000001.11:33 ------------------------------------------------------------
NC_000023.11:15 ------------------------------------------------------------
NC_000004.12:c1 ------------------------------------------------------------

NC_000013.11:c3 GCTCTCCTATGAATTCTTAGGGTAAATGAGAGATGGTAGTAGTTTTTTTCTGAATATAGG
NC_000001.11:33 ------------------------------------------------------------
NC_000023.11:15 ----------------------------------------------TCTCTGA-------
NC_000004.12:c1 ------------------------------------------------------------

NC_000013.11:c3 GTATATAAACCCTGAAAGTTGCTCAGTTGAGTCTTCAGAGCTAATAAGTGATGATAAATG
NC_000001.11:33 ------------------------------------------------------------
NC_000023.11:15 ------------------------------------------------------------
NC_000004.12:c1 ------------------------------------------------------------

NC_000013.11:c3 AAAAACAAATCTTGGAATAATTGATTTTCTGAAAATGAAAGGGCAGAGATATAAGGAAAT
NC_000001.11:33 ------------------------------------------------------------
NC_000023.11:15 ------------------------------------------------------------
NC_000004.12:c1 ------------------------------------------------------------

NC_000013.11:c3 TGGGAAATGTTAGACTATTGTGCCAAGAGTAATAAATGTATTCACTACCCTAAAAAACTA
NC_000001.11:33 ------------------------------------------------------------
NC_000023.11:15 ------------------------------------------------------------
NC_000004.12:c1 ------------------------------------------------------------

NC_000013.11:c3 TCCTTCTAGAATAGAAAAACAAGTTCAATGGCATGACATAATCAGTCAAATTTATTCTAT
NC_000001.11:33 ------------------------------------------------------------
NC_000023.11:15 ------------------------------------------------------------
NC_000004.12:c1 ------------------------------------------------------------

NC_000013.11:c3 AGGACAGGTCTTTTCAAACAGTCATCATTATTAATAGAGACCAAAGACACATGACAACCA
NC_000001.11:33 ------------------------------------------------------------
NC_000023.11:15 ------------------------------------------------------------
```

```
NC_000004.12:c1 ------------------------------------------------------------

NC_000013.11:c3 AATGCTGTGCATGGACTTTGACTTGATCCTGGTTTGCAAATCACATATTGGGGAAATTGT
NC_000001.11:33 ------------------------------------------------------------
NC_000023.11:15 ------------------------------------------------------------
NC_000004.12:c1 ------------------------------------------------------------

NC_000013.11:c3 AATATACCCAGCTATATAATATATTTGAGAATATATTTTTAATTTTCCTAGGTGTGTTCA
NC_000001.11:33 ------------------------------------------------------------
NC_000023.11:15 ------------------------------------------------------------
NC_000004.12:c1 ------------------------------------------------------------

NC_000013.11:c3 TGGTTACTATGGCTATGTAGGAGAATGGACTTATTTTAGGAGATACATCCGAGGTATATT
NC_000001.11:33 ------------------------------------------------------------
NC_000023.11:15 ------------------------------------------------------------
NC_000004.12:c1 ------------------------------------------------------------

NC_000013.11:c3 TAGAGGGGAAGTTATCCTCAAACAATACACAGTACATACAGGGAAAACATATGGCATTAA
NC_000001.11:33 ------------------------------------------------------------
NC_000023.11:15 ------------------------------------------------------------
NC_000004.12:c1 ------------------------------------------------------------

NC_000013.11:c3 ATGTTGAATCTGTGGTAAGAATAAGGGTGGTTGTCAAACAATTCTTTCAGCTTTTTTATA
NC_000001.11:33 ------------------------------------------------------------
NC_000023.11:15 ------------------------------------------------------------
NC_000004.12:c1 ------------------------------------------------------------

NC_000013.11:c3 TGTTTGCATATGTAAAATGAGAGAAAAAAAAAATCCCTCTCTGGAACCATGTTTCTTCACT
NC_000001.11:33 ------------------------------------------------------------
NC_000023.11:15 ------------------------------------------------------------
NC_000004.12:c1 ------------------------------------------------------------

NC_000013.11:c3 GAGCTTTGCAAGAAAAGGCCTCAAGAGAATGTATCATTGTTTATGGCTACCACCTCTACC
NC_000001.11:33 ------------------------------------------------------------
NC_000023.11:15 ------------------------------------------------------------
NC_000004.12:c1 ------------------------------------------------------------

NC_000013.11:c3 CTTCTTCTGCTGTTCTCTCTCTTGAATTCAGACCTGTGAGACTTTTATTCCTGTCTTCCC
NC_000001.11:33 ------------------------------------------------------------
NC_000023.11:15 ------------------------------------------------------------
NC_000004.12:c1 ------------------------------------------------------------

NC_000013.11:c3 ACCAAAACTCTCATTTCCACGTCGGCATTGGCCTCCATGTTGCCAGACCTGGTGGTCCTT
NC_000001.11:33 ------------------------------------------------------------
NC_000023.11:15 ------------------------------------------------------------
NC_000004.12:c1 ------------------------------------------------------------

NC_000013.11:c3 GCTTAGTCCTCATTTTGCTTGACCTCTAAGCAGCATTTGTTGCAGTCGATCAAATGAGTA
NC_000001.11:33 ------------------------------------------------------------
NC_000023.11:15 ------------------------------------------------------------
NC_000004.12:c1 ------------------------------------------------------------

NC_000013.11:c3 TAATGATTCTCTCAGACTCTCAGTTCTCCTACTTCTTGGCTTCTCCTTAGTATCTTTTGA
```

```
NC_000001.11:33 ------------------------------------------------------------
NC_000023.11:15 ------------------------------------------------------------
NC_000004.12:c1 ------------------------------------------------------------

NC_000013.11:c3 TGGTTCCTCCTCTTTTTTAAACATCGGAGTGCCTCTGGGCTCGGATCTCGTCTCAATTTG
NC_000001.11:33 -----------------------------------------------TCATTTCAATTTG
NC_000023.11:15 -----------------------------------------------ACTCCTGAGTCTG
NC_000004.12:c1 ---------------------------------------------TCTCCGTTGGGTCCG
                                                                *  .* ..*..*

NC_000013.11:c3 TGTTTAGTTCCTAAATCACCTTATCCTGTTGTATAGCTTTAAATACTATTCAGATGTGGA
NC_000001.11:33 ------------------------------------------------------------
NC_000023.11:15 ------------------------------------------------------------
NC_000004.12:c1 ------------------------------------------------------------

NC_000013.11:c3 GACTCCCAAGTTCATATTGCCACCCCAGCCTCTCCCCTGAACTTCAGACTCATTTATCCA
NC_000001.11:33 ------------------------------------------------------------
NC_000023.11:15 ------------------------------------------------------------
NC_000004.12:c1 ------------------------------------------------------------

NC_000013.11:c3 TCTGCCTACCTGATACATTCACTTGGTGCCAAATAGGCATTCCAAGTTTAATATATCCAG
NC_000001.11:33 ------------------------------------------------------------
NC_000023.11:15 ------------------------------------------------------------
NC_000004.12:c1 ------------------------------------------------------------

NC_000013.11:c3 AACTGAGCACGTTGGTCCCATCAGTCTCTTCTGCACAGCCCCAAACAAAGCAAGCACATG
NC_000001.11:33 ------------------------------------------------------------
NC_000023.11:15 ------------------------------------------------------------
NC_000004.12:c1 ------------------------------------------------------------

NC_000013.11:c3 CTATTTACCTGCTATTCCACATTCTTCCTCATCTCCGTATGAGGAACGTCGTTCTTCTCA
NC_000001.11:33 ------------------------------------------------------------
NC_000023.11:15 ------------------------------------------------------------
NC_000004.12:c1 ------------------------------------------------------------

NC_000013.11:c3 TTGCTCAGGCAGTAAACCCTGGTGTCTGATTCCTATTTTCCCCTCATACCTTACATTTAA
NC_000001.11:33 ------------------------------------------------------------
NC_000023.11:15 ------------------------------------------------------------
NC_000004.12:c1 ------------------------------------------------------------

NC_000013.11:c3 TCCCTCAGCGAATATGGTCACCAGTACTTTAAAAACATTCAGGCCGGGCGCGGTGGCTCA
NC_000001.11:33 ------------------------------------------------------------
NC_000023.11:15 ------------------------------------------------------------
NC_000004.12:c1 ------------------------------------------------------------

NC_000013.11:c3 CGCCTGCAATCCCAGCACTTTGGGAGGCCGAGGTGGGTGGATCACGGGATCAGGAGATGG
NC_000001.11:33 ------------------------------------------------------------
NC_000023.11:15 ------------------------------------------------------------
NC_000004.12:c1 ------------------------------------------------------------

NC_000013.11:c3 AGACCATCCTGGCTAACATGGTGAAACCCTGTCTGTACTAAAAATACAAAAAAATTAGCT
NC_000001.11:33 ------------------------------------------------------------
NC_000023.11:15 ------------------------------------------------------------
NC_000004.12:c1 ------------------------------------------------------------
```

```
NC_000013.11:c3 GGGTGTGGTGGCACGCGCCCGTAGTCCCAGCTACTCGGGAGGCTGAGGTGGGAGAATGGC
NC_000001.11:33 ------------------------------------------------------------
NC_000023.11:15 --------------------------------------------------------TAGT
NC_000004.12:c1 ------------------------------------------------------------

NC_000013.11:c3 GTGAACCCAGGAGGCAGAGCTTGCAGTGAGCCGAGATCACGCCACTGCACTCCAGCCCGG
NC_000001.11:33 ------------------------------------------------------------
NC_000023.11:15 GGGGACCTGGG-------------------------------------------------
NC_000004.12:c1 ------------------------------------------------------------

NC_000013.11:c3 GCGACAGAGCGAGACTCCGTCTCAAAAACAAACAAACAAAAAAACATTGGGAAGCTAAGC
NC_000001.11:33 ------------------------------------------------------------
NC_000023.11:15 ------------------------------------------------------------
NC_000004.12:c1 ------------------------------------------------------------

NC_000013.11:c3 ACTTCTCCAGCTCTTACCACTGCCATCTCAATCCAAGCTACATTCTAGGACAGATTATAA
NC_000001.11:33 ------------------------------------------------------------
NC_000023.11:15 ------------------------------------------------------------
NC_000004.12:c1 ------------------------------------------------------------

NC_000013.11:c3 CAGAGTTTCTCAGCCTTGGTACTATCAACGTTTTGGGCCAGCAATGTTGGGGGAAGGGCT
NC_000001.11:33 ------------------------------------------------------------
NC_000023.11:15 ------------------------------------------------------------
NC_000004.12:c1 ------------------------------------------------------------

NC_000013.11:c3 GTCTTGTGCATTGTAGGATGTTTAGTAGCATCCCTAGCTTCTACCTGCTAGATGCAAGTA
NC_000001.11:33 ------------------------------------------------------------
NC_000023.11:15 ---------------------TTAGTGACA------------------------------
NC_000004.12:c1 ------------------------------------------------------------

NC_000013.11:c3 GCACTCCGCCCCAACAGTTGTGACAGCCAGAAATGTCACCAGACATTGCCGAACTGTTCC
NC_000001.11:33 ------------------------------------------------------------
NC_000023.11:15 ------------------------------------------------------------
NC_000004.12:c1 ------------------------------------------------------------

NC_000013.11:c3 TTGGGGGGATATTGGATTATGGACCACTGGATTATAGCATTACCCTCCTATTGTTTCTGC
NC_000001.11:33 ------------------------------------------------------------
NC_000023.11:15 -------------------------------------CAATACCTGCCT-----------
NC_000004.12:c1 ------------------------------------------------------------

NC_000013.11:c3 TTCCTCTCACCGCCATGCAGCTTATTCTCCATCTAGCAACCAGTGTATCAGATCAAAACA
NC_000001.11:33 ------------------------------------------------------------
NC_000023.11:15 ------------------------------------------------------------
NC_000004.12:c1 ------------------------------------------------------------

NC_000013.11:c3 TGAATCAGATCATGTCTTGTCTTTTCTTCACAACCTTCCTGTGGCTTCCCATCTCATTAG
NC_000001.11:33 ------------------------------------------------------------
NC_000023.11:15 ------------------------------------------------------------
NC_000004.12:c1 ------------------------------------------------------------

NC_000013.11:c3 CGTCAAATCTGTAAGTGCCTACAGATCCCTTCCTGACCAATTTACATACCTGACCCACCC
NC_000001.11:33 ------------------------------------------------------------
NC_000023.11:15 ------------------------------------------------------------
```

```
NC_000004.12:c1 ------------------------------------------------------------

NC_000013.11:c3 ACCCCCCAACTTGGAGAGCAGCTTTTTCTCTTTGTGCAGATTGAAAAGGGCATCGTTTCC
NC_000001.11:33 ------------------------------------------------------------
NC_000023.11:15 ------------------------------------------------------------
NC_000004.12:c1 ------------------------------------------------------------

NC_000013.11:c3 ACTAGATGGATGCAGCCTCTTCCCCTGCTTTATTTTTCATCACAGCATTATCACCGCTTT
NC_000001.11:33 ----------------------------TTTAATCCTCATGGCTTTATTATCACCTC---
NC_000023.11:15 ----------------------------GCTCTTGTCAAAAGCAGCACTATCTCTGC---
NC_000004.12:c1 ------------------------------------------------------------

NC_000013.11:c3 ACATGTTCTGTTTGTTGAATGCATGGTAAGATGAGAAGTATAATTGTCAAAATGCATACA
NC_000001.11:33 ------------------------------------------------------------
NC_000023.11:15 ------------------------------------------------------------
NC_000004.12:c1 ------------------------------------------------------------

NC_000013.11:c3 GTCGTTCCTTGGTATCCATGGATTCCACATCTGTGGATTCAACCAACTGTGGATCCATAA
NC_000001.11:33 ------------------------------------------------------------
NC_000023.11:15 ------------------------------------------------------------
NC_000004.12:c1 ------------------------------------------------------------

NC_000013.11:c3 TATTAGACGAGAAAAAAAAGCATCTATTCTGAACACGCGTGGACTTTTTTCCTTGTCATT
NC_000001.11:33 ------------------------------------------------------------
NC_000023.11:15 ------------------------------------------------------------
NC_000004.12:c1 ------------------------------------------------------------

NC_000013.11:c3 ATTCCCTAAACAATACAGTATAACAACTATTTACGTAGTATTTTCATCGTATGAGGTATT
NC_000001.11:33 ------------------------------------------------------------
NC_000023.11:15 ------------------------------------------------------------
NC_000004.12:c1 ------------------------------------------------------------

NC_000013.11:c3 ATAAACTAGAGATGATTTAAAGGATACAGGAAGATGTGTAGGTTATATGCAAATACTATA
NC_000001.11:33 ------------------------------------------------------------
NC_000023.11:15 ------------------------------------------------------------
NC_000004.12:c1 ------------------------------------------------------------

NC_000013.11:c3 CCATTTTATGTAAAGGACTTTGTGGCTTAAACATGATTTATTATTTGTCATGATCCTGTA
NC_000001.11:33 ------------------------------------------------------------
NC_000023.11:15 ------------------------------------------------------------
NC_000004.12:c1 ------------------------------------------------------------

NC_000013.11:c3 GGTGACTGTAAAGTGCTTCTGCTGGTCTCACCTGGGCTGCTCATGCAGATTTTGATATCC
NC_000001.11:33 ------------------------------------------------------------
NC_000023.11:15 ------------------------------------------------------------
NC_000004.12:c1 ------------------------------------------------------------

NC_000013.11:c3 TTAGGGAGTCCTAGAACCAATTCCCCACAGATACTGAGGGATGACTTTTTAGTAAAAAGA
NC_000001.11:33 ------------------------------------------------------------
NC_000023.11:15 ------------------------------------------------------------
NC_000004.12:c1 ------------------------------------------------------------

NC_000013.11:c3 TAAGTTGCTAAAAGTTCACCTAACCCAATCTGAGAATGTCAAGAAAACCTTAGCATGTTA
```

NC_000001.11:33 --
NC_000023.11:15 --
NC_000004.12:c1 --

NC_000013.11:c3 ACATAACCTCAGAGATTCTACAAAGGTCTCCCAGAAGAAACGATGCCTAAATAAGACTTA
NC_000001.11:33 --
NC_000023.11:15 --
NC_000004.12:c1 --

NC_000013.11:c3 GTGTGGTCATATAGAAGGAGAGAAAGGGTATTAATAGTAAAGAGAAGGAGAAGGAGTGGA
NC_000001.11:33 --
NC_000023.11:15 --
NC_000004.12:c1 --

NC_000013.11:c3 GCCAATGCCCAGTTCAGTTTTGAACTGCCTCCTTGATACTTCCAAGAGGAGATTCTGCCG
NC_000001.11:33 --
NC_000023.11:15 --
NC_000004.12:c1 --

NC_000013.11:c3 TCAGGTAGTTAGAGAAACAAGTCTTAATCTCAGGAGAGACAGCTAGACTGTAAACACAGA
NC_000001.11:33 --
NC_000023.11:15 --
NC_000004.12:c1 --

NC_000013.11:c3 TTTGGGAATAATCACTAGATAAGCAGAAATGAGGGACTGTTTACATGTCAAAATTGGAAG
NC_000001.11:33 --
NC_000023.11:15 --
NC_000004.12:c1 --

NC_000013.11:c3 GAAAGCCTTGAAATTTAGTCTGATAAACTAAAGAAACTCCCTAAACAATTTGCCAGAGAG
NC_000001.11:33 --
NC_000023.11:15 --
NC_000004.12:c1 --

NC_000013.11:c3 AAAAATACAGAGGAAAATTTAAAACAAAATATAAGGCTGCACGGTGGCTTATACCTGTAA
NC_000001.11:33 --
NC_000023.11:15 --
NC_000004.12:c1 --

NC_000013.11:c3 TCCTAACACTTGGAGAGGCCAGGGCAGGCAGATTGCTTCAGCCCAGGAATTCAAGATCAG
NC_000001.11:33 --
NC_000023.11:15 --
NC_000004.12:c1 --

NC_000013.11:c3 CTTGGGGCAATAAGGCAAAACCTGATCTCTACCAAAAAAATACAAAATTAAGCTGGACGT
NC_000001.11:33 --
NC_000023.11:15 --
NC_000004.12:c1 --

NC_000013.11:c3 GGTGGCATGTGCCTGTGGTCCCAGCTCCTTGGGAGGCTGAGGTGGGAGGATCACCTGAAC
NC_000001.11:33 --
NC_000023.11:15 --
NC_000004.12:c1 --

```
NC_000013.11:c3 CTGGGGAAGTCGTGCTGCAGAGCTGTGATCACACCACTGCCCTCTGTCCTGGGTGACAGG
NC_000001.11:33 ------------------------------------------------------------
NC_000023.11:15 ------------------------------------------------------------
NC_000004.12:c1 ------------------------------------------------------------

NC_000013.11:c3 AAAAGGAAAAACAAGATGGGAGACTGCTAATGTGTTTCTCTCAGACAAGGAAAACAAAAC
NC_000001.11:33 ------------------------------------------------------------
NC_000023.11:15 ------------------------------------------------------------
NC_000004.12:c1 ------------------------------------------------------------

NC_000013.11:c3 GAGGTAAGGTCATTTGTTCTTGAGGAAGAACAATTATTTAGCTTAAACCAGACTAAAGCA
NC_000001.11:33 ------------------------------------------------------------
NC_000023.11:15 ------------------------------------------------------------
NC_000004.12:c1 ------------------------------------------------------------

NC_000013.11:c3 AGGGCAAGAGTTCTAAATCTGAAGAGTTCTAAAACATCAGTGATTATTGAAGTGTTGAGT
NC_000001.11:33 ------------------------------------------------------------
NC_000023.11:15 ------------------------------------------------------------
NC_000004.12:c1 ------------------------------------------------------------

NC_000013.11:c3 ACAACAGATAATAAAGCCAGCTGGCTATGCTACTCATATAAATTCTAAGGTTATTTTAAT
NC_000001.11:33 ------------------------------------------------------------
NC_000023.11:15 ------------------------------------------------------------
NC_000004.12:c1 ------------------------------------------------------------

NC_000013.11:c3 TTTTATGCATTATAAAAATCAGTTTTTCAAAGTTGGAAGTTTTTGGAGGAAAAAATGACT
NC_000001.11:33 ------------------------------------------------------------
NC_000023.11:15 ------------------------------------------------------------
NC_000004.12:c1 ------------------------------------------------------------

NC_000013.11:c3 TTTAAAGTATGAAGTTTTTCTCCCATTCTTACACTTCTAATTTAGTATTGTAAATGAACA
NC_000001.11:33 ------------------------------------------------------------
NC_000023.11:15 ------------------------------------------------------------
NC_000004.12:c1 ------------------------------------------------------------

NC_000013.11:c3 TGTTTTTAGTCGAACTGTAATAGTTGTCTAATTTTGTGTAACAAATGCTGCTCCAAATCT
NC_000001.11:33 ------------------------------------------------------------
NC_000023.11:15 ------------------------------------------------------------
NC_000004.12:c1 ------------------------------------------------------------

NC_000013.11:c3 TTGTGGTTTAAAAAACATCGTTTATTATTTGTCATGATCTGGTAGGTGACTGGAAGTTTC
NC_000001.11:33 ------------------------------------------------------------
NC_000023.11:15 ------------------------------------------------------------
NC_000004.12:c1 ------------------------------------------------------------

NC_000013.11:c3 CTCTGGTCTCACCTGGTTCTGTTCATGTGGCTGTAGTCAGTTAGCAGCTGGGCTGGGGTT
NC_000001.11:33 ------------------------------------------------------------
NC_000023.11:15 ------------------------------------------------------------
NC_000004.12:c1 ------------------------------------------------------------

NC_000013.11:c3 GAAGGGTCTAAGTGGGCCTCAGGGACATGTCTGAGACCATGGGGCTGGTGTCCCCGATGA
NC_000001.11:33 ------------------------------------------------------------
NC_000023.11:15 ------------------------------------------------------------
```

```
NC_000004.12:c1 ------------------------------------------------------------


NC_000013.11:c3 CATGTCTGAGGCTGTGGTGCTGGTGCTGGTGTCCCTGGGTGACATGTCTGAGGCCGTGGT
NC_000001.11:33 ------------------------------------------------------------
NC_000023.11:15 ------------------------------------------------------------
NC_000004.12:c1 ------------------------------------------------------------


NC_000013.11:c3 GCTGGTGTCCCCGGTGACATGTCTGAGGCTGTGGTGCTGGTGTCCCCGGTGACATGTCTG
NC_000001.11:33 ------------------------------------------------------------
NC_000023.11:15 ------------------------------------------------------------
NC_000004.12:c1 ------------------------------------------------------------


NC_000013.11:c3 AGGCTGTGGTGCTGGTATCCCCGGTGACATGTCTGAGGCTGTGGTACTGGTGTCACCTGG
NC_000001.11:33 ------------------------------------------------------------
NC_000023.11:15 ------------------------------------------------------------
NC_000004.12:c1 ------------------------------------------------------------


NC_000013.11:c3 CTTGTACGCTCGTACATGTGCACGTCCTGTTCACCCCCCTGCCCCGAGTATGCTCTCTTC
NC_000001.11:33 ------------------------------------------------------------
NC_000023.11:15 ------------------------------------------------------------
NC_000004.12:c1 ------------------------------------------------------------


NC_000013.11:c3 CTTCATAGTCTCCCCAGAGTTTTTCTTAACATGGCAACAGGAGTGTTCCAAGAGGGCAAA
NC_000001.11:33 ------------------------------------------------------------
NC_000023.11:15 ------------------------------------------------------------
NC_000004.12:c1 ------------------------------------------------------------


NC_000013.11:c3 AGCAAAAACTGCAGGTCTCTTAAGGACTAGTCTTAGAAGTCATACATCATCAGTTCTGCT
NC_000001.11:33 ------------------------------------------------------------
NC_000023.11:15 ------------------------------------------------------------
NC_000004.12:c1 ------------------------------------------------------------


NC_000013.11:c3 GCATTCTACCACACTATAATATATGTGGATTAATTCATTTCAACTTCACTACAATGCTAT
NC_000001.11:33 ------------------------------------------------------------
NC_000023.11:15 ------------------------------------------------------------
NC_000004.12:c1 ------------------------------------------------------------


NC_000013.11:c3 AAAAAGTAGGTAGTTACCTTACAACCCTCATTTTTTTTTTTTTTTTTTTTTTTTTTTTTT
NC_000001.11:33 ------------------------------------------------------------
NC_000023.11:15 ------------------------------CTTTTTTTTTTTTTTTT-------------
NC_000004.12:c1 ------------------------------------------------------------


NC_000013.11:c3 TTTTTTTTTTTTTGAGACGGAGTCTCGCTCTGTCGCCCAGGCTGGAGTGCAGTGGCGGGA
NC_000001.11:33 ------------------------------------------------------------
NC_000023.11:15 -----------------CGGAGTCTTGCTCTGTTGCCCAGGCTGGAGTGCAGTAGCATGA
NC_000004.12:c1 ------------------------------------------------------------


NC_000013.11:c3 TCTCGGCTCACTGCAAGCTCCGCCTCCCGGGTTCACGCCATTCTCCTGCCTCAGCCTCCC
NC_000001.11:33 ------------------------------------------------------------
NC_000023.11:15 TCTTGGCTCACTGCAACCTCCACCTCCCAGGTTAAAGCGATTCTCCTGCCTCAGCCTC--
NC_000004.12:c1 ------------------------------------------------------------


NC_000013.11:c3 AAGTAGCTGGGACTACAGGCGCCCGCCACTACGCCCGGCTAATTTTTTTGTATTTTTAGT
```

```
NC_000001.11:33 ------------------------------------------------------------
NC_000023.11:15 ------------CTACAGGCATGCATCACCACACCTGGCTAATTTTTTTGTATTTTTAGT
NC_000004.12:c1 ------------------------------------------------------------


NC_000013.11:c3 AGAGACGGGGTTTCACCGTTTTAGCCGGGATGGTCTCGATCTCTTGACCTCGTGATCCGC
NC_000001.11:33 ------------------------------------------------------------
NC_000023.11:15 AGAGACGGGGCTTCATCAGATTGGCCAGGCTGGTCTTGAACTCCTGATCTCGTGATCCGC
NC_000004.12:c1 ------------------------------------------------------------


NC_000013.11:c3 CCGCCTCGGCCTCCCAAAGTGCTGGGATTACAGGCGTGAGCCACCGCGCCCGGCCACAAC
NC_000001.11:33 ------------------------------------------------------------
NC_000023.11:15 CTGCCTCAGCCTCCCAAAGTACTGGAATTACAGGCATGAGCCACCGCGCTTGGCCTCTAT
NC_000004.12:c1 ------------------------------------------------------------


NC_000013.11:c3 CCTCATTTTTAAAGTGAGGACACTAAAACTCCGTACTAGTAGTTTGACCACAGGCCCCCA
NC_000001.11:33 ------------------------------------------------------------
NC_000023.11:15 CTTTGCCTTTTGA----------------TCTGGATTGGAACCTTGAT------------
NC_000004.12:c1 ------------------------------------------------------------


NC_000013.11:c3 ACAGGTAAGACACAGAACCAGGTAGGGTTTCTTTCATGCGTGTCCATGTGAAGAGACACC
NC_000001.11:33 ------------------------------------------------------------
NC_000023.11:15 ------------------------------------------------------------
NC_000004.12:c1 ------------------------------------------------------------


NC_000013.11:c3 AAACAGGCTTTGTGTGAGCAACATGGCTATTTATTTCACCTGGGTGCAGGCGGGCTGAGT
NC_000001.11:33 ------------------------------------------------------------
NC_000023.11:15 ------------------------------------------------------------
NC_000004.12:c1 ------------------------------------------------------------


NC_000013.11:c3 CAGAAAAGAGAGTCAGCAAAGGGTGGTGGATTATCATTACTTCTTATAGGTTTTGGGATA
NC_000001.11:33 -----------------------------------ACTACTTTTTAGAGATAT-------
NC_000023.11:15 ----GATGGGAGCCAACAG---------------CACCAGCTTCAGGGGCTCA--GGCTT
NC_000004.12:c1 -----------------------------------GCCGCTCTGCGGGACTCT-------
                                                      .... ...  . .. *

NC_000013.11:c3 GGTGGTGAAGTTAAGAGCAATGTTTTGCGGGCAGCGGTGGATCTCACAAATTACATTCTC
NC_000001.11:33 ------------------------------------------------------------
NC_000023.11:15 GGCTGGGAGGCTCAGAG-------------------------------------------
NC_000004.12:c1 ------------------------------------------------------------


NC_000013.11:c3 AAGGGTGGGGAGAATTACAAAGAACCTTCTTAAGGGTGGGGGAAATTATAAAGAACCTTC
NC_000001.11:33 ------------------------------------------------------------
NC_000023.11:15 -----------GAGTTGGTAAGAGGGGGCGTAGGGGTG----------------------
NC_000004.12:c1 ------------------------------------------------------------


NC_000013.11:c3 TTAAGGGTGGCGGAGATTACAAAGTACATTGATCAGTGAGGGTGGGGCAGAAACAAATCA
NC_000001.11:33 ------------------------------------------------------------
NC_000023.11:15 ------------------------------------------------------------
NC_000004.12:c1 ------------------------------------------------------------


NC_000013.11:c3 CAGTGGTAGAATGTCATTAGTTAAGGTTATTTTTACTTCTTTTGTGGATCTTCAGTTACT
NC_000001.11:33 ------------------------------------------------------------
NC_000023.11:15 ------------------------------------------------------------
NC_000004.12:c1 ------------------------------------------------------------
```

```
NC_000013.11:c3 TCAGGCCATCTGGATGTATACATGCAAGTCACAGGGGAAGTGATGGCTTGGCTTGGGATC
NC_000001.11:33 ------------------------------------------------------------
NC_000023.11:15 ------------------------------------------------------------
NC_000004.12:c1 ------------------------------------------------------------


NC_000013.11:c3 AGAGGCCTGACAGTTTCAATCTCTCCTTTGCTATCAACAAGCTCTGAGATGTTCATTTAT
NC_000001.11:33 ------------------------------------------------------------
NC_000023.11:15 ------------------------------------------------------------
NC_000004.12:c1 ------------------------------------------------------------


NC_000013.11:c3 CTGAGCCTGTTTCTTCTTCTGTAAAATGGGATTAATACAACTTGAGTGTCCCTTGCCTGA
NC_000001.11:33 ------------------------------------------------------------
NC_000023.11:15 ------------------------------------------------------------
NC_000004.12:c1 ------------------------------------------------------------


NC_000013.11:c3 AATGTTTGGGACCAGAAGTGTTTCAGATTTGGGTTTTTTCAGATTTTGAAATATTTGCAT
NC_000001.11:33 ------------------------------------------------------------
NC_000023.11:15 ------------------------------------------------------------
NC_000004.12:c1 ------------------------------------------------------------


NC_000013.11:c3 TATATTTAGCCAGTTGAGTATCCCTAATCACTTGAACCCAGGAGGCGGAGGTTGCAGTGA
NC_000001.11:33 -------------------------------------------------GGTTGTAATGA
NC_000023.11:15 ---------------------------------------AAGAAGCAGGTGCCCTGTGAA
NC_000004.12:c1 -----------------------------------------GAGGAAAAGCTCGCACCAG
                                                                   .. ..  ..

NC_000013.11:c3 GTCAGGATTGCGCCATTGCACTCCAGCCTGGTCAACAAGAGCAAAACTCCGTCTCAGGAA
NC_000001.11:33 GCTAGACTTGC-------------------------------------------------
NC_000023.11:15 GCCAGATCCTT-----TGCACACC------------------------------------
NC_000004.12:c1 GCAAGAATACC-------------------------------------------------
                *. **. .  .

NC_000013.11:c3 AAAAAAAGCTTCAGATTTTTGGATTAGGAATACTCAACCTATACTATTATACAGAATAAT
NC_000001.11:33 ------------------------------------------------------------
NC_000023.11:15 ------------------------------------------------------------
NC_000004.12:c1 ------------------------------------------------------------


NC_000013.11:c3 TTTTAGGATTGAATTGAGATGATTTCTAAAATATCTAGCAGAATACTTGATTCCTAGCAG
NC_000001.11:33 ------------------------------------------------------------
NC_000023.11:15 ------------------------------------------------------------
NC_000004.12:c1 ------------------------------------------------------------


NC_000013.11:c3 GTACTCAAAAATGTTAATTTCATCACGTTCTTCTTACTGTTCTCGGGTTGCTTTATTAGA
NC_000001.11:33 ------------------------------------------------------------
NC_000023.11:15 ------------------------------------------------------------
NC_000004.12:c1 ------------------------------------------------------------


NC_000013.11:c3 TGACGGAATGTTTTTATACCTGCTATTTGTCTTATTATCTTAAACAAAATTTGAATGACC
NC_000001.11:33 ------------------------------------------------------------
NC_000023.11:15 ------------------------------------------------------------
NC_000004.12:c1 ------------------------------------------------------------


NC_000013.11:c3 TACACCAGAATTATTATTAAAGCAGACAGTGATCTAACATTCCAGTAGCGGAGCAGTAAT
NC_000001.11:33 ----CCAAAGTTA-----------------------------------------------
NC_000023.11:15 ----TCAAACTCA-----------------------------------------------
```

```
NC_000004.12:c1 ------------------------------------------------------------

NC_000013.11:c3 AAAGAAAATTTCATACTGATGGAATTTATTTTAAGACTGTGATCTGAGCTGTTCTACCCT
NC_000001.11:33 ------------------------------------------------------------
NC_000023.11:15 ------------------------------------------------------------
NC_000004.12:c1 ------------------------------------------------------------

NC_000013.11:c3 AAGGTGTTAGTGGCTGATGATCACTTAATAGATTGCTCTGCCAGAGATTATTTACATTTT
NC_000001.11:33 ------------------------------------------------------------
NC_000023.11:15 ------------------------------------------------------------
NC_000004.12:c1 ------------------------------------------------------------

NC_000013.11:c3 CAGGGGAAATAAATTGCCAATGAAAGAATAGAAGAATTTTAGGTTTTGTGGCAAGCCAGT
NC_000001.11:33 ------------------------------------------------------------
NC_000023.11:15 ------------------------------------------------------------
NC_000004.12:c1 ------------------------------------------------------------

NC_000013.11:c3 GAGGAAACTGTGATTAGGAAAGGGATCTTTCAGTGGTACTCTTTGTGGTCTATCTACCAC
NC_000001.11:33 ------------------------------------------------------------
NC_000023.11:15 ------------------------------------------------------------
NC_000004.12:c1 ------------------------------------------------------------

NC_000013.11:c3 CATAGATGAGTCCTGGCAAGGTATCGAGTAACTTACCACAGTTTGTATATAATTTATAGA
NC_000001.11:33 ------------------------------------------------------------
NC_000023.11:15 ------------------------------------------------------------
NC_000004.12:c1 ------------------------------------------------------------

NC_000013.11:c3 TTTGGGTTTCTATAGGAAAAATTGCCTTTAATAAATTCAGATCATCATGCTATATTATTT
NC_000001.11:33 ------------------------------------------------------------
NC_000023.11:15 ------------------------------------------------------------
NC_000004.12:c1 ------------------------------------------------------------

NC_000013.11:c3 CATTTGGGTATGACATTTAGATGAGCACAGATGCAGTTACTATACTATAAATCTAACTGT
NC_000001.11:33 ------------------------------------------------------------
NC_000023.11:15 ------------------------------------------------------------
NC_000004.12:c1 ------------------------------------------------------------

NC_000013.11:c3 AGTGTTGACAAATTAAGGTCTAGGCCTGTTACGGGCTGAATTGTGCCCCACCCCCAATTC
NC_000001.11:33 ------------------------------------------------------------
NC_000023.11:15 ------------------------------------------------------------
NC_000004.12:c1 ------------------------------------------------------------

NC_000013.11:c3 ATAGGTTGAAGCCCTAATCCCCTGGAACTCGGAATGTGACTGTATTTGGACACAGGGCCG
NC_000001.11:33 ------------------------------------------------------------
NC_000023.11:15 ------------------------------------------------------------
NC_000004.12:c1 ------------------------------------------------------------

NC_000013.11:c3 TAAAAAGGTGATTAAGTTTAAATGAGGTCTTTATGGTGGACCCTAATCCAATATGACTAG
NC_000001.11:33 ------------------------------------------------------------
NC_000023.11:15 ------------------------------------------------------------
NC_000004.12:c1 ------------------------------------------------------------

NC_000013.11:c3 TATCTTTATAAGAAGAGGAAATTTGGATACGTGGGGAGACACCAGAAACAGACAGAGGGA
```

```
NC_000001.11:33 ------------------------------------------------------------
NC_000023.11:15 ------------------------------------------------------------
NC_000004.12:c1 ------------------------------------------------------------


NC_000013.11:c3 CAGCTATGTGAGGACATTTCAAGAAAGCAGCCATCTGCAAGTTAAGGAGAGAGGCCTCAG
NC_000001.11:33 ------------------------------------------------------------
NC_000023.11:15 ------------------------------------------------------------
NC_000004.12:c1 ------------------------------------------------------------


NC_000013.11:c3 AAACCAAACCAGCCAACAACTTTATCTTAGATTTCCAGCCTCCAGAACTGTGAGAAAATA
NC_000001.11:33 ------------------------------------------------------------
NC_000023.11:15 ------------------------------------------------------------
NC_000004.12:c1 ------------------------------------------------------------


NC_000013.11:c3 AATTTCTGTTGGTCCAGCTACCCAGTCTGTAGTGTTATCAGGGTTTTTTTGTTTTTGTTT
NC_000001.11:33 ------------------------------------------------------------
NC_000023.11:15 ------------------------------------------------------------
NC_000004.12:c1 ------------------------------------------------------------


NC_000013.11:c3 TTTTGTTTTTGAGACAGAGTTTTGCTCTTGTTGCCCAGGCTGGAGTACAATGGCCTGATC
NC_000001.11:33 ------------------------------------------------------------
NC_000023.11:15 ------------------------------------------------------------
NC_000004.12:c1 ------------------------------------------------------------


NC_000013.11:c3 TCAGCTCATCACAACCTCCGCCTCCCAGGTTCAAGCGATTCTCCTGCCTCAGTCTCCCAA
NC_000001.11:33 ------------------------------------------------------------
NC_000023.11:15 ------------------------------------------------------------
NC_000004.12:c1 ------------------------------------------------------------


NC_000013.11:c3 GTAGCTGGGATTACAGGCATGAGCCACCACACATGGCTAATTTTGTATTTTTAGTAGAGA
NC_000001.11:33 ------------------------------------------------------------
NC_000023.11:15 ------------------------------------------------------------
NC_000004.12:c1 ------------------------------------------------------------


NC_000013.11:c3 CGGGGTTTCTCCATGTTGGTCAGACTGGTCTCAAACTCCTGACCTCAGGTGATCCGCCCA
NC_000001.11:33 ------------------------------------------------------------
NC_000023.11:15 ------------------------------------------------------------
NC_000004.12:c1 ------------------------------------------------------------


NC_000013.11:c3 CCTTGGCCTCATAAAGTACTGGGATTACAGGCGTGAGCCGTTGTGCCCGGCCTGTAGTGT
NC_000001.11:33 ------------------------------------------------------------
NC_000023.11:15 ------------------------------------------------------------
NC_000004.12:c1 ------------------------------------------------------------


NC_000013.11:c3 TACGTTACGACAGCCCATGCAAACTAACACAGGGCCCAGTACCAACCCTTACACATGGGT
NC_000001.11:33 ------------------------------------------------------------
NC_000023.11:15 ------------------------------------------------------------
NC_000004.12:c1 ------------------------------------------------------------


NC_000013.11:c3 GTTTAATATTTGTTATTTTTTCAAAACTAAAACTGTTGGTTGCTACTTGGGAGGCTGAGG
NC_000001.11:33 ------------------------------------------------------------
NC_000023.11:15 ------------------------------------------------------------
NC_000004.12:c1 ------------------------------------------------------------
```

```
NC_000013.11:c3 TGGAGGATTGCTTGAGCCCAGGAGTTCAAGTCCAGCCTGGGCAACATAGTGAGACCCCAT
NC_000001.11:33 ------------------------------------------------------------
NC_000023.11:15 ------------------------------------------------------------
NC_000004.12:c1 ------------------------------------------------------------

NC_000013.11:c3 TCCTTTAAAAAAATTATATATACACACACATACACACACAATATATTATATATGTATATA
NC_000001.11:33 ------------------------------------------------------------
NC_000023.11:15 ------------------------------------------------------------
NC_000004.12:c1 ------------------------------------------------------------

NC_000013.11:c3 CACATATATATGCATATGCATATATAATATATACATATACGTGTGTACATGTATATATAT
NC_000001.11:33 ------------------------------------------------------------
NC_000023.11:15 ------------------------------------------------------------
NC_000004.12:c1 ------------------------------------------------------------

NC_000013.11:c3 AAAAGATCTACACACAAACATAAAATAAATAATAAATTTTTGTTGTAGAAAAATCAGGGA
NC_000001.11:33 ------------------------------------------------------------
NC_000023.11:15 ------------------------------------------------------------
NC_000004.12:c1 ------------------------------------------------------------

NC_000013.11:c3 TGGATTATAGATGCTGTTTCTCATGAAGTAATCAAATTAAGTTTCACAACCCAGCATTTG
NC_000001.11:33 ------------------------------------------------------------
NC_000023.11:15 ------------------------------------------------------------
NC_000004.12:c1 ------------------------------------------------------------

NC_000013.11:c3 AAATAGACCATTTTAAAAGAGTCGTCTTATCCTTTATTTTGCCACCAAGGCTGTAGCCCC
NC_000001.11:33 ------------------------------------------------------------
NC_000023.11:15 ------------------------------------------------------------
NC_000004.12:c1 ------------------------------------------------------------

NC_000013.11:c3 TTTCCTTCCATTTCTCTGTCCTCTTCACCCCACCCTACACTATCTTGTCCTGTTCCAACA
NC_000001.11:33 ------------------------------------------------------------
NC_000023.11:15 ------------------------------------------------------------
NC_000004.12:c1 ------------------------------------------------------------

NC_000013.11:c3 TACATGCATATAGGCCAGCTATAGCTCACTTCCTATATTTTCAGGTGTATTTTGTCTAGC
NC_000001.11:33 ------------------------------------------------------------
NC_000023.11:15 ------------------------------------------------------------
NC_000004.12:c1 ------------------------------------------------------------

NC_000013.11:c3 AAGCTTTAGTTATTTTTCCTAAGCAACAGGATTATCTTCCATGTTGTTTTTCTATTTCAC
NC_000001.11:33 ------------------------------------------------------------
NC_000023.11:15 ------------------------------------------------------------
NC_000004.12:c1 ------------------------------------------------------------

NC_000013.11:c3 TTTGTGAAAAATGAGGAGGCCAGTTGAAGAGGGAGAGAAGGCCTTAACATTTATTGAGCA
NC_000001.11:33 ------------------------------------------------------------
NC_000023.11:15 ------------------------------------------------------------
NC_000004.12:c1 ------------------------------------------------------------

NC_000013.11:c3 CTTATTTTATGCCAGGTACTGTTATTCACTTTACATAAAACATTTCCTTTAGTGAGAAAA
NC_000001.11:33 ------------------------------------------------------------
NC_000023.11:15 ------------------------------------------------------------
```

```
NC_000004.12:c1 ------------------------------------------------------------

NC_000013.11:c3 CAGCAGTGGCCACATTTAGAAAAATGAAAAGCAACAGTGACGATCTTGTAACATTGCTTT
NC_000001.11:33 ------------------------------------------------------------
NC_000023.11:15 ------------------------------------------------------------
NC_000004.12:c1 ------------------------------------------------------------

NC_000013.11:c3 AAAATGAGTCTCTGGCCCTTGCCCTACTTTCTGTATATGTGCTTAGCTCTGCCATTATCA
NC_000001.11:33 ------------------------------------------------------------
NC_000023.11:15 ------------------------------------------------------------
NC_000004.12:c1 ------------------------------------------------------------

NC_000013.11:c3 GATCTACTGTACCTGGTAGTTATGAAAAGTACATTAATTATTTAAATCTCTAGATGTAAG
NC_000001.11:33 ------------------------------------------------------------
NC_000023.11:15 ------------------------------------------------------------
NC_000004.12:c1 ------------------------------------------------------------

NC_000013.11:c3 TCATTTTGTGAGATGACAAACAGCATAACCTTGGGGTATGTGTCTATTAGAATGGGTTTA
NC_000001.11:33 ------------------------------------------------------------
NC_000023.11:15 ------------------------------------------------------------
NC_000004.12:c1 ------------------------------------------------------------

NC_000013.11:c3 GAAACAACACAGGGGTACTGGTGCTTCTGAGAGTATGTCATTGATAGTCACAAGTAATTT
NC_000001.11:33 ------------------------------------------------------------
NC_000023.11:15 ------------------------------------------------------------
NC_000004.12:c1 ------------------------------------------------------------

NC_000013.11:c3 GCCGGTGCATGCCTTCTTCTTAATGGAAAGGAATTGAAGGAAAAATGTAAAAAGTTGCTA
NC_000001.11:33 ------------------------------------------------------------
NC_000023.11:15 ------------------------------------------------------------
NC_000004.12:c1 ------------------------------------------------------------

NC_000013.11:c3 ACTTCTAGCCAATTAGACAGTAATTCTGAGGAAGGGACAGCTACTCTAAGGCTATCGGAA
NC_000001.11:33 ------------------------------------------------------------
NC_000023.11:15 ------------------------------------------------------------
NC_000004.12:c1 ------------------------------------------------------------

NC_000013.11:c3 GCATGCCTGGGCTGGTGTAAGATGTCCCACCTAGGAGAGAAAATGATTTGAAAAAGATAG
NC_000001.11:33 ------------------------------------------------------------
NC_000023.11:15 ------------------------------------------------------------
NC_000004.12:c1 ------------------------------------------------------------

NC_000013.11:c3 TTTCCTTTGGCTTGGAATGCTGAAAGAAAAAGGAGCCCAGGGAAAGGAGGCTTGTTAACC
NC_000001.11:33 ------------------------------------------------------------
NC_000023.11:15 ------------------------------------------------------------
NC_000004.12:c1 ------------------------------------------------------------

NC_000013.11:c3 CTACACACCAGGGGTCAGGCTATGAGTCAAATGTGACCTGAGGTCTGTTCCTGTAGTCCG
NC_000001.11:33 ------------------------------------------------------------
NC_000023.11:15 ------------------------------------------------------------
NC_000004.12:c1 ------------------------------------------------------------

NC_000013.11:c3 TTGGGATGAGGGTGATTTTTACATTTTTAAGGAGTTATAAAAAAAAAATGTAACAGAAAC
```

```
NC_000001.11:33 ------------------------------------------------------------
NC_000023.11:15 ------------------------------------------------------------
NC_000004.12:c1 ------------------------------------------------------------


NC_000013.11:c3 CTTGTTTGGCCCACAAAGCCTAAAATGTTTACTATCTGACCTTTTGCAGAAAAAGTGTGC
NC_000001.11:33 ------------------------------------------------------------
NC_000023.11:15 ------------------------------------------------------------
NC_000004.12:c1 ------------------------------------------------------------


NC_000013.11:c3 TGTTCCTTGCATATAAGTATAGAGGATTATAGAGGGTCTGTTTGTATCTTCAACTGACAG
NC_000001.11:33 ------------------------------------------------------------
NC_000023.11:15 ------------------------------------------------------------
NC_000004.12:c1 ------------------------------------------------------------


NC_000013.11:c3 GCTTGTGGTATACTGTAAACAGTTGAGTATGAGAAATCTTCCCAAACATTTGTAGCATTT
NC_000001.11:33 ------------------------------------------------------------
NC_000023.11:15 ------------------------------------------------------------
NC_000004.12:c1 ------------------------------------------------------------


NC_000013.11:c3 CATAGGATTATCCTATTTTAGACCAGTGTTAAGAGCTCATTTGTATTAGTTTGCTAGGAC
NC_000001.11:33 ------------------------------------------------------------
NC_000023.11:15 ------------------------------------------------------------
NC_000004.12:c1 ------------------------------------------------------------


NC_000013.11:c3 TGCTGTAACAAAGTACCACAAATTGGGTAGCTTGAACAACAAAAATTTATTTTCTTACGG
NC_000001.11:33 ------------------------------------------------------------
NC_000023.11:15 ------------------------------------------------------------
NC_000004.12:c1 ------------------------------------------------------------


NC_000013.11:c3 TTCTGAAGACTAGAAGTCCAAAATGATGTATTGACATGGTGGGTTTCTTCTGAGGACTGT
NC_000001.11:33 ------------------------------------------------------------
NC_000023.11:15 ------------------------------------------------------------
NC_000004.12:c1 ------------------------------------------------------------


NC_000013.11:c3 GAGAACCTGTCCCATGACTCTTCCTAACTTCTGGTGATTTGCTGAAAATCTTTGGTGTCC
NC_000001.11:33 ------------------------------------------------------------
NC_000023.11:15 ----------------------------------------------------------GC
NC_000004.12:c1 -------------------------------------------------CTCCAATACCC


NC_000013.11:c3 CTTGGCTTGTAGATGTACCGCCCCGGTCTCTGCCTTCATGTTCATTTGATATTCTCCTTG
NC_000001.11:33 CTCAGCTAGTAAATG---------------------------------------------
NC_000023.11:15 CACGGTTTCTGGAAGCA-------------------------------------------
NC_000004.12:c1 TCGGGTTCGTGGACCTG-------------------------------------------
                .  .*.*  *..*

NC_000013.11:c3 TTTGTGTGTATCTGTATCCAAATTTCCTTTTTTTTATAAGGACACTGGTCGTATTGGATT
NC_000001.11:33 ------------------------------------------------------------
NC_000023.11:15 ------------------------------------------------------------
NC_000004.12:c1 ------------------------------------------------------------


NC_000013.11:c3 AGGTCACTCCATGACCTCTTCTTTATCACTTGCAAAGACCCTATTTCCAAATAAAGTCAC
NC_000001.11:33 ------------------------------------------------------------
NC_000023.11:15 ------------------------------------------------------------
NC_000004.12:c1 ------------------------------------------------------------
```

```
NC_000013.11:c3 ATTCACAGGTATTGTAGTTTAGGACTTCAGCATCTTTTGTAAGGGGCATAATCTAACCCA
NC_000001.11:33 ------------------------------------------------------------
NC_000023.11:15 ------------------------------------------------------------
NC_000004.12:c1 ------------------------------------------------------------

NC_000013.11:c3 TAATATCTTCTAAGCCTTTGTTTTACAGAGAAGAACAGAGAAACTATGAAAGTATAAAGA
NC_000001.11:33 ------------------------------------------------------------
NC_000023.11:15 ------------------------------------------------------------
NC_000004.12:c1 ------------------------------------------------------------

NC_000013.11:c3 AGTCATTCAAATCATACAGCTGTCTAGGAGCAGAACCAGTATTCAAATGTAGGTCCACTG
NC_000001.11:33 ------------------------------------------------------------
NC_000023.11:15 ------------------------------------------------------------
NC_000004.12:c1 ------------------------------------------------------------

NC_000013.11:c3 ACTAACCCAGTATTTGTTCACCTACATACTATATAATATGCAATTGCAGTGTAATGAGGT
NC_000001.11:33 ------------------------------------------------------------
NC_000023.11:15 ------------------------------------------------------------
NC_000004.12:c1 ------------------------------------------------------------

NC_000013.11:c3 TCAGTCAAGTGTAATACTTGTTTGTACTAGTTACACTATTCCAGTCATCTTTCCTATGCC
NC_000001.11:33 ------------------------------------------------------------
NC_000023.11:15 ------------------------------------------------------------
NC_000004.12:c1 ------------------------------------------------------------

NC_000013.11:c3 TTCCGTTAATCATGGAGCAATAATAAATTATAGATGGATCAGAATTAATTCATTTCTATT
NC_000001.11:33 ------------------------------------------------------------
NC_000023.11:15 ------------------------------------------------------------
NC_000004.12:c1 ------------------------------------------------------------

NC_000013.11:c3 ACAGAAATGAGCACATCATGCACTATTGTTAGTGTATTCCGTTCTGTATCCTTTGTGTAT
NC_000001.11:33 ------------------------------------------------------------
NC_000023.11:15 ------------------------------------------------------------
NC_000004.12:c1 ------------------------------------------------------------

NC_000013.11:c3 TCAGTTCTGAAAGAATTTACTCAAGGCCTGCTGTTTGCTAAGGAGTATCTAAGCATACTT
NC_000001.11:33 ------------------------------------------------------------
NC_000023.11:15 ------------------------------------------------------------
NC_000004.12:c1 ------------------------------------------------------------

NC_000013.11:c3 GCATAGTAAAGTTGTAGAGAATGTGCCTTAGTCTGGGTTTCTACATTGCCTATTTCAACA
NC_000001.11:33 ------------------------------------------------------------
NC_000023.11:15 ------------------------------------------------------------
NC_000004.12:c1 ------------------------------------------------------------

NC_000013.11:c3 GGTTCTAGTCACAACTCTGCTGCGAGTTCTAAACTCAAGTCTTGCTGGTTTTGTTCTTTG
NC_000001.11:33 ------------------------------------------------------------
NC_000023.11:15 ------------------------------------------------------------
NC_000004.12:c1 ------------------------------------------------------------

NC_000013.11:c3 ATAAATCACTTTCTTAAGCCTTAATTCTCGTGAGTTGTTCCCATGAGATTTTCAGAAACC
NC_000001.11:33 ------------------------------------------------------------
NC_000023.11:15 ------------------------------------------------------------
```

```
NC_000004.12:c1 ------------------------------------------------------------


NC_000013.11:c3 CCAGGTAACTTTAGGATTCCATGATTCTGTGACTGTGGTGAGACATTTTTTGCATTTGGA
NC_000001.11:33 ------------------------------------------------------------
NC_000023.11:15 ---------------------------------------------------GCATTTGGA
NC_000004.12:c1 ------------------------------------------------------------


NC_000013.11:c3 GCATCTGATGGCTAAGGAAACCACATGTAACTTCATGCTAAGGCGCGTATGGTAAACCAC
NC_000001.11:33 ------------------------------------------------------------
NC_000023.11:15 AAATT-------------------------------------------------------
NC_000004.12:c1 ------------------------------------------------------------


NC_000013.11:c3 TATAAAGACAGACTTATCTGGCCAACATGGAGTGATGTTTGTGAATGCTAATTGTAGGGC
NC_000001.11:33 ------------------------------------------------------------
NC_000023.11:15 ------------------------------------------------------------
NC_000004.12:c1 ------------------------------------------------------------


NC_000013.11:c3 CCTAGTATGTGATTATAAAAATAATACCAAACAATTACTTGTGACTTTGTAAGTCTTTCT
NC_000001.11:33 ------------------------------------------------------------
NC_000023.11:15 ------------------------------------------------------------
NC_000004.12:c1 ------------------------------------------------------------


NC_000013.11:c3 TTATATATATTCTGGTGTGTAATTGATTATAAAGGGAGAGAATATTGTCAGAGTGCCGTA
NC_000001.11:33 ------------------------------------------------------------
NC_000023.11:15 -----CATGTTCTG----------------------------------------------
NC_000004.12:c1 ------------------------------------------------------------


NC_000013.11:c3 TTTTGTATTACAATTACTGGCCAGATTATTCAGTGAAGAACTAAATTAAAAAGGTTACAC
NC_000001.11:33 ------------------------------------------------------------
NC_000023.11:15 ------------------------------------------------------------
NC_000004.12:c1 ------------------------------------------------------------


NC_000013.11:c3 CCCAGAGATCACCTAATATTTCTTACCATTGGCATACCAAGGAATTGAGTTTAATTGCAC
NC_000001.11:33 ------------------------------------------------------------
NC_000023.11:15 ------------------------------------------------------------
NC_000004.12:c1 ------------------------------------------------------------


NC_000013.11:c3 CTTCTTCGCACTCCCCTGGTGTTTTCTCTTCTGTGTAGCCAGCTTCCAAGATGGCCCCCA
NC_000001.11:33 ------------------------------------------------------------
NC_000023.11:15 ------------------------------------------------------------
NC_000004.12:c1 ------------------------------------------------------------


NC_000013.11:c3 AGGATTCTTGCCTCTTGTTACTCATGCCCTTATGCAGTCCCCTCCCAGACTAAATAGGAT
NC_000001.11:33 ------------------------------------------------------------
NC_000023.11:15 ------------------------------------------------------------
NC_000004.12:c1 ------------------------------------------------------------


NC_000013.11:c3 TAACTTGTACAGCCAGTAGAATATTGTGTAAATGACAGTATGTCACTTTTGAGGCTAGGT
NC_000001.11:33 ------------------------------------------------------------
NC_000023.11:15 ------------------------------------------------------------
NC_000004.12:c1 ------------------------------------------------------------


NC_000013.11:c3 CATGAAAGACATTGTGGCTTCCACTTTCCTGTCTTGGAATACTCACTATGGGGAAGGTCA
```

```
NC_000001.11:33 ------------------------------------------------------------
NC_000023.11:15 ------------------------------------------------------------
NC_000004.12:c1 ------------------------------------------------------------

NC_000013.11:c3 TGAGGACATTCCAGCAGCCCTGTGGAGAGGTCCATGTGATAAGAAACTAAGGTCTCCTCC
NC_000001.11:33 ------------------------------------------------------------
NC_000023.11:15 ------------------------------------------------------------
NC_000004.12:c1 ------------------------------------------------------------

NC_000013.11:c3 CAGCACCCAGCAGTAACTTGCCTAGTATGTGAGTGAGCCACCTTGCAATTGGATCGTGCA
NC_000001.11:33 ------------------------------------------------------------
NC_000023.11:15 ------------------------------------------------------------
NC_000004.12:c1 ------------------------------------------------------------

NC_000013.11:c3 GCCCTCAAAGCCCTCAGAGCCCTCAAAGGATGGCCGTCCCAGCCGACATCTTGTCTACAG
NC_000001.11:33 ------------------------------------------------------------
NC_000023.11:15 ------------------------------------------------------------
NC_000004.12:c1 ------------------------------------------------------------

NC_000013.11:c3 CTGAGCCAGATCCACCCAGTTAAGCTGCTCCTGGATTTCTGACCACAGAAACTTTATGAA
NC_000001.11:33 ------------------------------------------------------------
NC_000023.11:15 ------------------------------------------------------------
NC_000004.12:c1 ------------------------------------------------------------

NC_000013.11:c3 ATAATAAATGTTTATTGTTTCAAGCTACTAAAGTTAAGGAAATATGCTACGCAGCAATAG
NC_000001.11:33 ------------------------------------------------------------
NC_000023.11:15 ------------------------------------------------------------
NC_000004.12:c1 ------------------------------------------------------------

NC_000013.11:c3 ATAAAAATACAACCTTTGTTGATTCTGCCTTTTTCTCAGATCATTGTATATATCATCTGC
NC_000001.11:33 ------------------------------------------------------------
NC_000023.11:15 ------------------------------------------------------------
NC_000004.12:c1 ------------------------------------------------------------

NC_000013.11:c3 CACTAGATTTTTACTGAGCAGAGCTTGTGTCTGAACTCATTTTTGTAGGCCTTACAGAAG
NC_000001.11:33 -----------------GCAAAGCTAGAATTTGAACTTAT--------------------
NC_000023.11:15 -----------------GTGGGGCTTATTTCTGAATATAG--------------------
NC_000004.12:c1 ------------------------------------------------------------

NC_000013.11:c3 TCGTTACAGCCTTCAATTACTTCTTAGATTGAATGTGGTTGACTTGGATCGTGGCTAGAA
NC_000001.11:33 ------------------------------------------------------------
NC_000023.11:15 ------------------------------------------------------------
NC_000004.12:c1 ------------------------------------------------------------

NC_000013.11:c3 CATCTGGGTTGCTCCTATTGCTTCTGTTTGGAAAGGTGCAAATTACCTCTACCATTTTAT
NC_000001.11:33 ------------------------------------------------------------
NC_000023.11:15 ---------------------------------------------------------GAC
NC_000004.12:c1 ------------------------------------------------------------

NC_000013.11:c3 TTAGAAACAAATGCAGTATTTTTAATGAGTTTCATTTAAAAAGAATTTAGATTGTTTGAA
NC_000001.11:33 ------------------------ATGTGTCTGGCTTAAAAGG-----------------
NC_000023.11:15 TCATAAATAGCTG----------GATCTCTTGGCCTTTCAAAGAA---------------
NC_000004.12:c1 ------------------------------------------------------------
```

```
NC_000013.11:c3 ATATGTAAAAGAAATAAAAATACAATTTTTTATCTGAGACTACATTTCAACAAAGATCTA
NC_000001.11:33 ------------------------------------------------------------
NC_000023.11:15 ------------------------------------------------------------
NC_000004.12:c1 ------------------------------------------------------------

NC_000013.11:c3 AACAAATGGGAGAAGACTCACTAGAACCAGGAAAAACTGTTGAAGGGGGTGGTAATCTTT
NC_000001.11:33 ------------------------------------------------------------
NC_000023.11:15 ------------------------------------------------------------
NC_000004.12:c1 ------------------------------------------------------------

NC_000013.11:c3 GAAGAGTTTACTAGATGAAGAATGACCTTTTTTTGCTTGCTTTTATTTTTCATTTGAAAA
NC_000001.11:33 ------------------------------------------------------------
NC_000023.11:15 ------------------------------------------------------------
NC_000004.12:c1 ------------------------------------------------------------

NC_000013.11:c3 TAACTTCAAATGTAAGTTAAAAAAACAACACAAAGAACGTCTGTATGCCTTTTACCTAGA
NC_000001.11:33 ------------------------------------------------------------
NC_000023.11:15 ------------------------------------------------------------
NC_000004.12:c1 ------------------------------------------------------------

NC_000013.11:c3 TTCACCTGTCATTAAGATTTTGTCTCATTTGGATGCAAAGCCAAACTACAATGAGACACC
NC_000001.11:33 ------------------------------------------------------------
NC_000023.11:15 --------------------------------AAGAAAAACCAAACT-------------
NC_000004.12:c1 ------------------------------------------------------------

NC_000013.11:c3 ATCTCTTACCAGTTCAAAATGGCTACTATTAAAAATCAGAAAAGAACGTATGTTGGCGAG
NC_000001.11:33 ------------------------------------------------------------
NC_000023.11:15 ------------------------------------------------------------
NC_000004.12:c1 ------------------------------------------------------------

NC_000013.11:c3 GCCGTGGAGAAAAGGGAACACTTACACGCCGTTGGTGGGAATGCAAATTAGTTCTACCCC
NC_000001.11:33 ------------------------------------------------------------
NC_000023.11:15 ------------------------------------------------------------
NC_000004.12:c1 ------------------------------------------------------------

NC_000013.11:c3 TGTGGAGAGCAACTAAAAATAGAAATACTTATCTGAGCTAGCAATCCCATTACTGGGTAT
NC_000001.11:33 ------------------------------------------------------------
NC_000023.11:15 ------------------------------------------------------------
NC_000004.12:c1 ------------------------------------------------------------

NC_000013.11:c3 ATACCCACAGGAAAATAAATCATTCTGCCAGAAAGACACCTGCGCTCAGATGTTCATTGC
NC_000001.11:33 ------------------------------------------------------------
NC_000023.11:15 ------------------------------------------------------------
NC_000004.12:c1 ------------------------------------------------------------

NC_000013.11:c3 AGCACCGTTCACAATAGCAAACACATGAAATCAATGTAAGTGCCCATTAATTGATGAGTG
NC_000001.11:33 ------------------------------------------------------------
NC_000023.11:15 ------------------------------------------------------------
NC_000004.12:c1 ------------------------------------------------------------

NC_000013.11:c3 GATAAAGAAAATGTGTTACCTATACAACATGGAATACTACACAGCCATTAAAAAGAATGA
NC_000001.11:33 ------------------------------------------------------------
NC_000023.11:15 ------------------------------------------------------------
```

```
NC_000004.12:c1 ------------------------------------------------------------

NC_000013.11:c3 AATCAAATCCTTTGCAGTGACATGAATGTAACTGGAGGCAATTATCCTAACGGAACTAAC
NC_000001.11:33 ------------------------------------------------------------
NC_000023.11:15 ------------------------------------------------------------
NC_000004.12:c1 ------------------------------------------------------------

NC_000013.11:c3 GCAGAACAAGAAAACTAAATACTGCATGCTCTCACTTGTAAGTGGGAGATAAGTCTTGGG
NC_000001.11:33 ------------------------------------------------------------
NC_000023.11:15 ------------------------------------------------------------
NC_000004.12:c1 ------------------------------------------------------------

NC_000013.11:c3 TACACATGGACATAAAGATGGGAACAGTAGACACTGGGAACTCCAAAAGCAAGGAGGGAG
NC_000001.11:33 ------------------------------------------------------------
NC_000023.11:15 ------------------------------------------------------------
NC_000004.12:c1 ------------------------------------------------------------

NC_000013.11:c3 AGCGGGAGGGAGGGGGCAAGGGCTGAAAAACTGCGTGCTGGGTACTGTGTTCACTGTTGT
NC_000001.11:33 ------------------------------------------------------------
NC_000023.11:15 ------------------------------------------------------------
NC_000004.12:c1 ------------------------------------------------------------

NC_000013.11:c3 GGCAACAGGAACATTAGAGGTCCAAACCTCAGCTTCACACAATATACCCAAGTAATAACT
NC_000001.11:33 ------------------------------------------------------------
NC_000023.11:15 ------------------------------------------------------------
NC_000004.12:c1 ------------------------------------------------------------

NC_000013.11:c3 GCACGTGTATCCCCGGAATCTAAAATTTTAAAAAGCTGAAACTTACAAAATTAAATTTAA
NC_000001.11:33 ------------------------------------------------------------
NC_000023.11:15 ------------------------------------------------------------
NC_000004.12:c1 ------------------------------------------------------------

NC_000013.11:c3 AAATAAAGATTTCATCTCATTTGCTTTATCACTTATGCACATGTGTGCCTTGCTCTCTTC
NC_000001.11:33 ------------------------------------------------------------
NC_000023.11:15 ------------------------------------------------------------
NC_000004.12:c1 ------------------------------------------------------------

NC_000013.11:c3 CGCTAATACACGTGTGTGTAGATACGTATGTAAATGAATATACACACATTTTTTTCTAAG
NC_000001.11:33 ------------------------------------------------------------
NC_000023.11:15 ------------------------------------------------------------
NC_000004.12:c1 ------------------------------------------------------------

NC_000013.11:c3 CCATTTAAGGGTAACTTACATACATCATATCTGTGTGCCTAAATTCTTAGGTGAATAATT
NC_000001.11:33 ------------------------------------------------------------
NC_000023.11:15 ------------------------------------------------------------
NC_000004.12:c1 ------------------------------------------------------------

NC_000013.11:c3 CCTAAGAAATACAGTTAGCAACTTCAGCAAGTTTAACATTGATACTCTACTATTGTAATC
NC_000001.11:33 ------------------------------------------------------------
NC_000023.11:15 ------------------------------------------------------------
NC_000004.12:c1 ------------------------------------------------------------

NC_000013.11:c3 CAGTTTTGTCAGTCAATTCAATACTGTCCTATATGCCATTTTTTCCTTTCTTGCATTTAA
```

```
NC_000001.11:33 ------------------------------------------------------------
NC_000023.11:15 -------------------------------CATGACATCTCTTCCTTT-----------
NC_000004.12:c1 --------------------------------------------CCTTT-----------

NC_000013.11:c3 CTTTAGTCTCCTTCAATCTGTAACATTTCCATAGCCGTCTTTGTCTTTTATGGCATTGAT
NC_000001.11:33 ------------------------------------------------------------
NC_000023.11:15 ------------------------------------------------------------
NC_000004.12:c1 ------------------------------------------------------------

NC_000013.11:c3 ATTTTTGGAAGAATAAAGACCTCTCCCCACTCCTTTTTTAGTGGAATGTTCCACATTTTG
NC_000001.11:33 ------------------------------------------------------------
NC_000023.11:15 ------------------------------------------------------------
NC_000004.12:c1 ------------------------------------------------------------

NC_000013.11:c3 GATCTGTCTGATTTTTTTTATGATTAGACTCGGGTTGTAGATATCTTTTTAAATGTTCTA
NC_000001.11:33 ------------------------------------------------------------
NC_000023.11:15 ------------------------------------------------------------
NC_000004.12:c1 ------------------------------------------------------------

NC_000013.11:c3 AAATGTGGAAACAGTAGTCCATTCAAAGGATCAATGTAAAACAAAATTTAGGCCACAGAA
NC_000001.11:33 ------------------------------------------------------------
NC_000023.11:15 ------------------------------------------------------------
NC_000004.12:c1 ------------------------------------------------------------

NC_000013.11:c3 ACTTTGAAATAATTATTAGAAGCCAAATTGGAAAACCAAAATGTGTTGTAGAATCAACTT
NC_000001.11:33 ------------------------------------------------------------
NC_000023.11:15 ------------------------------------------------------------
NC_000004.12:c1 ------------------------------------------------------------

NC_000013.11:c3 CCTAGAAAATGTTTAGAGTAAGATAGTTTTTAGTCTTTCAAGTTTCTATAGATGGGTGGT
NC_000001.11:33 ------------------------------------------------------------
NC_000023.11:15 ------------------------------------------------------------
NC_000004.12:c1 ------------------------------------------------------------

NC_000013.11:c3 TCGACAACTGGGTTTTCAAAAATCAAGAAATGTCTTGGCATTGAGGTGAACTTTTGTGGC
NC_000001.11:33 ------------------------------------------------------------
NC_000023.11:15 ------------------------------------------------------------
NC_000004.12:c1 ------------------------------------------------------------

NC_000013.11:c3 TTTCTTTAGTTTCATCATGAACTCGGGCTCCCCAAAAGCCAGTGGTATCTTTCTGTAGAA
NC_000001.11:33 ------------------------------------------------------------
NC_000023.11:15 ------------------------------------------------------------
NC_000004.12:c1 ------------------------------------------------------------

NC_000013.11:c3 ACAGGATTTTTCTTTCAACTTCTGCTAAATTTATTCAACAAACATTACCAAATTCTTGTT
NC_000001.11:33 ------------------------------------------------------------
NC_000023.11:15 ------------------------------------------------------------
NC_000004.12:c1 ------------------------------------------------------------

NC_000013.11:c3 ATCCTGACATGGAGGTGGTACCTGGGAATACAAAGAAGGGCATAAAAGAGACTTAATATG
NC_000001.11:33 ------------------------------------------------------------
NC_000023.11:15 ------------------------------------------------------------
NC_000004.12:c1 ------------------------------------------------------------
```

```
NC_000013.11:c3 CCATATTGTTTATTAAAAGATAATCAGAGGCTGACATGCCACAGAGGACAGGAGCAGCTT
NC_000001.11:33 ------------------------------------------------------------
NC_000023.11:15 ------------------------------------------------------------
NC_000004.12:c1 ------------------------------------------------------------

NC_000013.11:c3 CTACGGGGGGGTGGTCTGTAACTGCACTGGAGGGGGAATACCTAAATGCTGCTTTCCGCC
NC_000001.11:33 ------------------------------------------------------------
NC_000023.11:15 ------------------------------------------------------------
NC_000004.12:c1 ------------------------------------------------------------

NC_000013.11:c3 CAAAGTGGGAAATCCCTGCTGCTCCTGTAAGAGTCACATCTATCTGCAAAGAAAAAACTC
NC_000001.11:33 ------------------------------------------------------------
NC_000023.11:15 ------------------------------------------------------------
NC_000004.12:c1 ------------------------------------------------------------

NC_000013.11:c3 TGGCTGGTGCTTGGCATTTTTACCTATTCTCTTGCATTAAAAATGACTAAGCCATACTGA
NC_000001.11:33 ------------------------------------------------------------
NC_000023.11:15 ------------------------------------------------------------
NC_000004.12:c1 ------------------------------------------------------------

NC_000013.11:c3 ATAGTTTCTCACTTGTAAGTGGGAGATAAGCCTTAGGTACATATGGACATAAAGATGGGA
NC_000001.11:33 ------------------------------------------------------------
NC_000023.11:15 ------------------------------------------------------------
NC_000004.12:c1 ------------------------------------------------------------

NC_000013.11:c3 ACAGTAGTAAAGGCAATCCCTTTCAGAGACTGGGGGATAAGAGATTATTTCTTTCAATGC
NC_000001.11:33 ------------------------------------------------------------
NC_000023.11:15 -----------------------------------------GCATTATTTTCTCCAAC--
NC_000004.12:c1 ------------------------------------------------------------

NC_000013.11:c3 TTGAATTCTTGAATTGTTGTTTTGCACTATATGAAGTGTCCCAGGGATTCGCTTTTTTTT
NC_000001.11:33 ------------------------------------------------------------
NC_000023.11:15 ------------------------------------------------------------
NC_000004.12:c1 ------------------------------------------------------------

NC_000013.11:c3 TTTTTTTAAAAAAAAGCACTAAATTTTAAATTATCTGTTTTTTCATAATTTTCAACAGTA
NC_000001.11:33 ------------------------------------------------------------
NC_000023.11:15 ----------------TGTCAATCTTTAAACTGCTCTTTTTCTC----------------
NC_000004.12:c1 ------------------------------------------------------------

NC_000013.11:c3 TCACAGGTAAATAGTAAAATTATTTTAAAATGGAAAATGTTTTTTAAGTTTGAAGACAAA
NC_000001.11:33 ------------------------------------------------------------
NC_000023.11:15 ------------------------------------------------------------
NC_000004.12:c1 ------------------------------------------------------------

NC_000013.11:c3 GCAACTTTCCATCTTCTGATGCTATGGTCCTGGTTGTGGCTTTGTTCCTGACTTTCTTTA
NC_000001.11:33 ------------------------------------------------------------
NC_000023.11:15 ------------------------------------------------------------
NC_000004.12:c1 ------------------------------------------------------------

NC_000013.11:c3 GATTTAAGAAAGATGCTTTGAGATCTTTAGCAAATTCTTAAACAATTCATTTTATCTGAA
NC_000001.11:33 ------------------------------------------------------------
NC_000023.11:15 ------------------------------------------------------------
```

```
NC_000004.12:c1 ------------------------------------------------------------


NC_000013.11:c3 GACTAACATATTCCCAGCCACAACTTCACTTGTCAGGTTTCTGCAGGGAATAGTTTATTA
NC_000001.11:33 ------------------------------------------------------------
NC_000023.11:15 ------------------------------------------------------------
NC_000004.12:c1 ------------------------------------------------------------


NC_000013.11:c3 AACTTGGGGTGCCCAAAATACTGTAACTTAAACCTGGAAATAAAATACCAATATGCTGAA
NC_000001.11:33 ------------------------------------------------------------
NC_000023.11:15 ------------------------------------------------------------
NC_000004.12:c1 ------------------------------------------------------------


NC_000013.11:c3 CCTGGGATAATTTCTAAGAGTACTACTAAAGATACTTCTATGTTTTTCCAAACATAACAT
NC_000001.11:33 -------------------------------------CCATGTTCTTTCCA---------
NC_000023.11:15 ---------------------------------ACCCTTTCATTCTTTCCA---------
NC_000004.12:c1 -------------------------------------CCCCATTCCCTCAG---------
                                                        .. ..**....* .

NC_000013.11:c3 AGGGGAATGGGTATTGGTTGTAGTATGTAGATGTTCTTTAACTTAAATTATATTACCTGG
NC_000001.11:33 ------------------------------------------------------------
NC_000023.11:15 ---GGCAAGGG-------------------------------------------------
NC_000004.12:c1 ------------------------------------------------------------


NC_000013.11:c3 GAAGACTGGTAATTTTCATGTCATTTTCAATATAAGAATAAAGAGAATATAATAATTACC
NC_000001.11:33 -------------CTTCATGTTACTGCCACCA----AGTAAAGAGGAT------------
NC_000023.11:15 -------------CCCTCAGATACTTTCTTTCCA--AATAGGGCTGATA-----------
NC_000004.12:c1 ------------------------------------------------------------


NC_000013.11:c3 CATTCTAAATTATGTGTTGTACATTTTCTTAATACAGTAACTAAATATGCCAAAGAAAAG
NC_000001.11:33 ------------------------------------------------------------
NC_000023.11:15 ------------------------------------------------------------
NC_000004.12:c1 ------------------------------------------------------------


NC_000013.11:c3 GAGCTGTGATTTCATATTATCAGAAATATGACTTTTAAAAGCACCTAGATAACAGAAATT
NC_000001.11:33 ------------------------------------------------------------
NC_000023.11:15 ------------------------------------------------------------
NC_000004.12:c1 ------------------------------------------------------------


NC_000013.11:c3 CAAGAAGATCCATGAATGTAATATAGGGGAAAATGTTTTTTTTTTAATTTTGTGGCTAGA
NC_000001.11:33 ------------------------------------------------------------
NC_000023.11:15 ------------------------------------------------------------
NC_000004.12:c1 ------------------------------------------------------------


NC_000013.11:c3 TATTATTCTATATTGACAAGTATTGACCAAAGAGAAATATTTTATTGATTAAAAATATAT
NC_000001.11:33 ------------------------------------------------------------
NC_000023.11:15 ------------------------------------------------------------
NC_000004.12:c1 ------------------------------------------------------------


NC_000013.11:c3 TTTAATCAAAATAATTCTCTGATAGGACATTCTAAATAAGCTAATTTTGACAATTTTCCT
NC_000001.11:33 ------------------------------------------------------------
NC_000023.11:15 ------------------------------------------------------------
NC_000004.12:c1 ------------------------------------------------------------


NC_000013.11:c3 TCTGTTAAAATTGAGAGGAAAGAACACAGTTGCTTTCTCCCATGCAGTTGAAATGCATTT
```

```
NC_000001.11:33 ------------------------------------------------------------
NC_000023.11:15 ------------------------------------------------------------
NC_000004.12:c1 ------------------------------------------------------------

NC_000013.11:c3 AACTATAGTATATAATATAACTGGTCTTTCAGATTACCTTAGTTATTGCGCTACCCACAG
NC_000001.11:33 ------------------------------------------------------------
NC_000023.11:15 ------------------------------------------------------------
NC_000004.12:c1 ------------------------------------------------------------

NC_000013.11:c3 TAGGACAATATTGTAAAGCATAAGAGAACCTCTGAATCTGATTATAAATGAAATAGAGAT
NC_000001.11:33 ------------------------------------------------------------
NC_000023.11:15 ------------------------------------------------------------
NC_000004.12:c1 ------------------------------------------------------------

NC_000013.11:c3 TACTTAGAGGTTATTTCTTGTCTATATTGTTAATATAGAATAAGAGTTGACTAGGTAGTC
NC_000001.11:33 ------------------------------------------------------------
NC_000023.11:15 ------------------------------------------------------------
NC_000004.12:c1 ------------------------------------------------------------

NC_000013.11:c3 TTACATTTAAAAATCCCTGTTGTGGCCAGGCATGCGTGGTAGGCCAAGGTGGGAGGATCG
NC_000001.11:33 ------------------------------------------------------------
NC_000023.11:15 ------------------------------------------------------------
NC_000004.12:c1 ------------------------------------------------------------

NC_000013.11:c3 CTTGAGCCCAGGAGTTAAAGACCAGCCTGGGCAACATGGCAAAACCCCATCTCTACAAAA
NC_000001.11:33 ------------------------------------------------------------
NC_000023.11:15 ------------------------------------------------------------
NC_000004.12:c1 ------------------------------------------------------------

NC_000013.11:c3 AATTAGCCAGATGTAGTGGCATGCGCCTGTGGTCCCAACCACTCAGGAGGCTGAGGTGGG
NC_000001.11:33 ------------------------------------------------------------
NC_000023.11:15 ------------------------------------------------------------
NC_000004.12:c1 ------------------------------------------------------------

NC_000013.11:c3 AGAATCGCTGAGCCCGAGAGGCAGAGGTTGCAGTGAGCCAAGATTGTGCCACTGCACTCC
NC_000001.11:33 ------------------------------------------------------------
NC_000023.11:15 ------------------------------------------------------------
NC_000004.12:c1 ------------------------------------------------------------

NC_000013.11:c3 AGCCTGGCTGACAGAGCAAGACCCTGTTCAAAAAGAAAAAAAAAAAAAAAATCCCTGGTG
NC_000001.11:33 ------------------------------------------------------------
NC_000023.11:15 ------------------------------------------------------------
NC_000004.12:c1 ------------------------------------------------------------

NC_000013.11:c3 CTCTTTACAGAGCTTAGAGAGCAGAATGTAGAGTGCTTAAAATTCCTTAGCACAATATAT
NC_000001.11:33 ------------------------------------------------------------
NC_000023.11:15 ------------------------------------------------------------
NC_000004.12:c1 ------------------------------------------------------------

NC_000013.11:c3 TATATAAAATCGCCAGGTGTTTCTTTTTTATATCAAAACTTACTGGATATCATACATTTT
NC_000001.11:33 ------------------------------------------------------------
NC_000023.11:15 ------------------------------------------------------------
NC_000004.12:c1 ------------------------------------------------------------
```

```
NC_000013.11:c3 ATATGATTAAACCTTAAACAGGCTTCTCTGTAAAAACACTGTGCCTCGGCCAGGCGCGAT
NC_000001.11:33 ------------------------------------------------------------
NC_000023.11:15 ------------------------------------------------------------
NC_000004.12:c1 ------------------------------------------------------------

NC_000013.11:c3 GGTTCACACCTGTAATCCCAGCACTTTGGGAGGCCGAGGCGGGTGGATCATTTAAGGTCA
NC_000001.11:33 ------------------------------------------------------------
NC_000023.11:15 ------------------------------------------------------------
NC_000004.12:c1 ------------------------------------------------------------

NC_000013.11:c3 AGAGTTCAAGACTAGCCTGGCCAACATAGTGAAACCCCGTCTCTACTAAAAATACAAAAG
NC_000001.11:33 ------------------------------------------------------------
NC_000023.11:15 ------------------------------------------------------------
NC_000004.12:c1 ------------------------------------------------------------

NC_000013.11:c3 ATTAGCTGGGTGTGATGGTGCACACCTGTAATCCCAGCTACTTGGGAGGCTGAGACAGGA
NC_000001.11:33 ------------------------------------------------------------
NC_000023.11:15 ------------------------------------------------------------
NC_000004.12:c1 ------------------------------------------------------------

NC_000013.11:c3 GAATTGCTTGAACCCTGGAGGTGGAGGTTGCAGTGAGCCAAGATCATGCCATTGCACTCC
NC_000001.11:33 ------------------------------------------------------------
NC_000023.11:15 ------------------------------------------------------------
NC_000004.12:c1 ------------------------------------------------------------

NC_000013.11:c3 AGCCTGGGTGAGAGTGAGGCTTTGTCTCAAAAAAAAAAAAAAAAAAAAAAAGGCCCTGCC
NC_000001.11:33 ------------------------------------------------------------
NC_000023.11:15 ------------------------------------------------------------
NC_000004.12:c1 ------------------------------------------------------------

NC_000013.11:c3 TCCTTTTCTAACCTGTTATCCTGCCTTAGTCCACCTTTGTCTTAGTCTATTCAGGCTGCT
NC_000001.11:33 ------------------------------------------------------------
NC_000023.11:15 ------------------------------------------------------------
NC_000004.12:c1 ------------------------------------------------------------

NC_000013.11:c3 ATAACAAAATACTTTACACTGGATAATTTATAAACAGCAGATATTTATTGCTCACAGTTC
NC_000001.11:33 --------------------------------------------------CTCACAGTCT
NC_000023.11:15 --------------------------------------------------CTTGCA---T
NC_000004.12:c1 ------------------------------------------------------------

NC_000013.11:c3 TGTAGGCTGAGAAGCCCAAAATCAAGATGTGGCAGATTCAGTGTCTTGGGAGTGCTCACT
NC_000001.11:33 CCGAGGCGGA------------------------------------------AGCTCCCT
NC_000023.11:15 TTGAAGTGGAG-----------------------------------------TATTTCTT
NC_000004.12:c1 ----------------------------------------------------AGCCTCTA
                                                                     .... .

NC_000013.11:c3 CTCTGCTTCCAACATGGTGCCTTCGTGGTGTGTCCTCACATGGTGGAAGGGTCAAAGAGG
NC_000001.11:33 CTCAGCTTGT--------------------------------------------------
NC_000023.11:15 CCTTGCGTGGGAC-----------------------------------------------
NC_000004.12:c1 CTCGGTCT----------------------------------------------------
                *.. *. *

NC_000013.11:c3 CTACCTCAGGTGCTCATCCTATTCATGAGGGCTCCACCCTCAAAACCAAATTACCTCCTA
NC_000001.11:33 ------------------------------------------------------------
NC_000023.11:15 ------------------------------------------------------------
```

```
NC_000004.12:c1 ------------------------------------------------------------


NC_000013.11:c3 AAGCCCCAAATTCTTAATACTGTTGCATTGAGGATTAAGATTTCAACATACGAATTTGAG
NC_000001.11:33 ------------------------------------------------------------
NC_000023.11:15 ------------------------------------------------------------
NC_000004.12:c1 ------------------------------------------------------------


NC_000013.11:c3 GGGACACAAGTTCAGATCATTGGAACCTTGAATTTCATTCTCTAACAGCATTGAACGGCT
NC_000001.11:33 ------------------------------------------------------------
NC_000023.11:15 ------------------------------------------------------------
NC_000004.12:c1 ------------------------------------------------------------


NC_000013.11:c3 TGTCATTTCTTTGCTCACATCTGACTTCTACATCTTACAGTTGCATGTGCAGTATGGTAG
NC_000001.11:33 ------------------------------------------------------------
NC_000023.11:15 ------------------------------------------------------------
NC_000004.12:c1 ------------------------------------------------------------


NC_000013.11:c3 CCACTACCCGCACGTGGCTATTTAAATTTAAGTTTAGGCCGGGCGTGGTGACTCACACCT
NC_000001.11:33 ------------------------------------------------------------
NC_000023.11:15 ------------------------------------------------------------
NC_000004.12:c1 ------------------------------------------------------------


NC_000013.11:c3 GTATTCTCAGCACTTTGCGGGGAGGCCAAGGCAGGTGGATCACTTGAGCCCGGGAGTTTG
NC_000001.11:33 ------------------------------------------------------------
NC_000023.11:15 ------------------------------------------------------------
NC_000004.12:c1 ------------------------------------------------------------


NC_000013.11:c3 AGACTAACTTGGGCAACATGGTGAAACGCCGTCTCTACCAAAAATACAAAAATTAGCCAG
NC_000001.11:33 ------------------------------------------------------------
NC_000023.11:15 ------------------------------------------------------------
NC_000004.12:c1 ------------------------------------------------------------


NC_000013.11:c3 TCTCATAACCCAGTCTCTAAATAAATAATAGATTAAAATTTAAAAATAAAATTTAAAGTG
NC_000001.11:33 ------------------------------------------------------------
NC_000023.11:15 ------------------------------------------------------------
NC_000004.12:c1 ------------------------------------------------------------


NC_000013.11:c3 CTGTATTAAAAATAAATTTGTTTGAATTCATTAAATTAAATTTAAAATTCAGCCTCTCGG
NC_000001.11:33 ------------------------------------------------------------
NC_000023.11:15 ------------------------------------------------------------
NC_000004.12:c1 ------------------------------------------------------------


NC_000013.11:c3 TTGTCGTAGCTATATTCTAAGTACTCAGTGGCCACATGTGCTAGCAGCTACACCTTATTG
NC_000001.11:33 ------------------------------------------------------------
NC_000023.11:15 -TGTC-------------------------------------------------------
NC_000004.12:c1 ------------------------------------------------------------


NC_000013.11:c3 GACAGTGCAGATATAGAAGATTTCCATCATAGAAAGTGCTGCTGGACAGTGCTACCTTAC
NC_000001.11:33 -----------------------------------------CTGGACAGTG---------
NC_000023.11:15 -----------------------------------------CTATGCAATGTA-------
NC_000004.12:c1 -----------------------------------------CGGCGCAGTGGC-------
                                                         * . .**.**

NC_000013.11:c3 AGCCCTTTGTTTTAAGTAATCTCTATTTCATTTATAATCAGATTCAGAGACCAGCCTGAG
```

```
NC_000001.11:33 ------------------------------------------------------------
NC_000023.11:15 ------------------------------------------------------------
NC_000004.12:c1 ------------------------------------------------------------

NC_000013.11:c3 TAACCAACGTAAGTAATCTCTGATTACTTAGGGTGGTCTCCTCTCACTGCCCACCTCTCT
NC_000001.11:33 ------------------------------------------------------------
NC_000023.11:15 ------------------------------------------------------------
NC_000004.12:c1 ------------------------------------------------------------

NC_000013.11:c3 CTCTCCTTTCTTATCTATTTAAAGCATTCTTCTTCAAGATTTTTATCAGGTGCAAGAAAC
NC_000001.11:33 ------------------------------------------------------------
NC_000023.11:15 ------------------------------------------------------------
NC_000004.12:c1 ------------------------------------------------------------

NC_000013.11:c3 ATTTTCAGCCAGTCCTGTTCATTCCCAATTTCAGGGTTCTAGCCCTCGAGTCTTCCTTTT
NC_000001.11:33 ------------------------------------------------------------
NC_000023.11:15 ------------------------------------------------------------
NC_000004.12:c1 ------------------------------------------------------------

NC_000013.11:c3 TTTTTTTTTGAGACAGAGTCTCACTCTGTTGCCCAGGCTAGAGTGCAGTGGCACAATCTT
NC_000001.11:33 ------------------------------------------------------------
NC_000023.11:15 ------------------------------------------------------------
NC_000004.12:c1 ------------------------------------------------------------

NC_000013.11:c3 GGCTCATGGCAACCTCTGCCTCCCAGGTTCAAGTGACTCTAATGCCTCAGCCTCCTGAGT
NC_000001.11:33 ------------------------------------------------------------
NC_000023.11:15 ------------------------------------------------------------
NC_000004.12:c1 ------------------------------------------------------------

NC_000013.11:c3 AGCTGGGATTACAGGTGTGTACCACCATTACCAGCTAATTTTTTGTATTTTTAGTAGAGA
NC_000001.11:33 ------------------------------------------------------------
NC_000023.11:15 ------------------------------------------------------------
NC_000004.12:c1 ------------------------------------------------------------

NC_000013.11:c3 TGGGGTTTCGCTGTGTTGGCCAGGTTGGTGTCAAACTCCTGGGCTCAAGTGATCTTCCTG
NC_000001.11:33 ------------------------------------------------------------
NC_000023.11:15 ------------------------------------------------------------
NC_000004.12:c1 ------------------------------------------------------------

NC_000013.11:c3 CCTCAGCCTCCCAGAGTGCTGGGATTACAGGCATGAGCCACCATGCACAGCCTCTGTCAT
NC_000001.11:33 ------------------------------------------------------------
NC_000023.11:15 ------------------------------------------------------------
NC_000004.12:c1 ------------------------------------------------------------

NC_000013.11:c3 TTTGGCATTATGCCTTTATTATTGACACTTTACTGTGTGAAAATTTAGGTTTCCATATAT
NC_000001.11:33 ------------------------------------------------------------
NC_000023.11:15 --------------------------------------GGATATTTAG------------
NC_000004.12:c1 ------------------------------------------------------------

NC_000013.11:c3 GTCTTCTCTTCTGACTGTACACTTTTGAAATTTCACCTCCTAAATATTTCTTCAACCCAT
NC_000001.11:33 ----------------------------------------------TTTTTCAAATCCAT
NC_000023.11:15 ----------------------------------------------TATCCCCGGCCGTT
NC_000004.12:c1 ------------------------------------------------------------
```

```
NC_000013.11:c3 CTACTTCACTCTAAATATAGTAGAAATGGTACAGGCTTAGGAGTCAAGCAGGTCTCATTT
NC_000001.11:33 ---------------------------------------------------------TCT
NC_000023.11:15 ---CCTTCCTCT---------------------------------------------TAT
NC_000004.12:c1 ---------------------------------------------------------TCT
                                                                         * *

NC_000013.11:c3 TGAATTCTAGCCCCTCCACTTACAATCTTTGTGACCCTAGACAGCTTTAATCTTGTTGAG
NC_000001.11:33 TGGAGTGTGACCCATC--------------------------------------------
NC_000023.11:15 TGAATTCCAGCAACACC-------------------------------------------
NC_000004.12:c1 CGGGGTCTGACCCGGCGAGCGGCA------------------------------------
                .*.. * ...*    *

NC_000013.11:c3 CCTTAGTTTCCTAATGCATAAAATAAGAATGATAGGCCGGGCGCGGTGGTTCACGCCTGT
NC_000001.11:33 ------------------------------------------------------------
NC_000023.11:15 ------------------------------------------------------------
NC_000004.12:c1 ------------------------------------------------------------

NC_000013.11:c3 AATCCCAGCCCTTTGGGAGGCTGAGGCGGGTGGATCACCTGAGGTTGGGAGTTTGAGACC
NC_000001.11:33 ----------------------------AGTGGGTCA----------------TGAGGTC
NC_000023.11:15 ----------------------------TGCCAGTCATCC-------------AGGCACC
NC_000004.12:c1 -----------TTTGGGG----------TGCGGGCCGGCG-------------AGGGCTG
                             *. ...*.                 *.  .

NC_000013.11:c3 AGCCTGACCAACATGGAGAAACCCCATCTCTACTAAAAATACAAAATTAGCTGAGCATGA
NC_000001.11:33 AGTCTG------------------------------------------------------
NC_000023.11:15 GGCCCCACGAATTT----------------------------------------------
NC_000004.12:c1 GGTCTG------------------------------------------------------
                .*.*.

NC_000013.11:c3 TGGCACATGCCTGTAATCCCAGCTACTCGGGAGGCTGAGGCTGGAGAATCGCTTAAACCT
NC_000001.11:33 ------------------------------------------------------------
NC_000023.11:15 -----------------------------------------CAGAACATCCCTTACA---
NC_000004.12:c1 ------------------------------------------------------------

NC_000013.11:c3 GGGAGACAGAGGTTGTGGTGAGCCAAGATCGTGCCATTGCCCTCCAGCCTGGGCAACAAG
NC_000001.11:33 -------------------------------------------------TGGGTGGCA--
NC_000023.11:15 -------------------------------------------------GAAGTGACA--
NC_000004.12:c1 -------------------------------------------------TGGAGGGCC--
                                                  ... ..*

NC_000013.11:c3 AGCAAGACTCCGTCTCAAAAAAAAAAAAAAAGTGGTAAACTAGGCATTCAGCTTTACAAT
NC_000001.11:33 ------------------------------------------------------------
NC_000023.11:15 ------------------------------------------------------------
NC_000004.12:c1 ------------------------------------------------------------

NC_000013.11:c3 TAGAGGCCATATATATCATCAGAAAACAATAGTAAATGTTATTTATTTTTACCGTTTTTT
NC_000001.11:33 ------------------------------------------------------------
NC_000023.11:15 ------------------------------------------------------------
NC_000004.12:c1 ------------------------------------------------------------

NC_000013.11:c3 TTTAAACAGAGGCTTGCTCTGTCACCCAGGCTGGAGTGCAGTGGCGCGATGTCGGCTCAC
NC_000001.11:33 ------------------------------------------------------------
NC_000023.11:15 ------------------------------------------------------------
NC_000004.12:c1 ------------------------------------------------------------

NC_000013.11:c3 TGCAACCTCCGCCTCCCAGATTCAAGCGAGTCTCCTGCCTCAGCCTCCCAAATAGCTGGG
NC_000001.11:33 ------------------------------------------------------------
NC_000023.11:15 ------CTGCGGCTCCTATA----------------------------------------
```

```
NC_000004.12:c1 ------------------------------------------------------------

NC_000013.11:c3 ACTACAGGCACGAGCCATCATTCACGGCTAATTTCTGTATTTTTAGTAGAGACGGGGTTT
NC_000001.11:33 ------------------------------------------------------------
NC_000023.11:15 ------------------------------------------------------------
NC_000004.12:c1 ------------------------------------------------------------

NC_000013.11:c3 CACCATGTTGGCCAGGCTGGTCTCAAACTCTTGACCTCAAGTGATCTACCCACCTTGGCC
NC_000001.11:33 ------------------------------------------------------------
NC_000023.11:15 ------------------------------------------------------------
NC_000004.12:c1 ------------------------------------------------------------

NC_000013.11:c3 TCCCAAAGTGCTGGGATTACAGGCATGAGCCGCTGCACCCGGCCTAATTGTGTTTTATCT
NC_000001.11:33 ------------------------------------------------------------
NC_000023.11:15 ------------------------------------------------------------
NC_000004.12:c1 ------------------------------------------------------------

NC_000013.11:c3 TCTATTTCTAATGCTTTCATTGTACTCTTTTGACTCAACTTTAAGATCTTTTGTGTCCAA
NC_000001.11:33 ------------------------------------------------------------
NC_000023.11:15 ------------------------------------------------------------
NC_000004.12:c1 ------------------------------------------------------------

NC_000013.11:c3 GCACTATCTTAACATCAAACCTTTTAACCATGCAGTTTCCCATTATTCTGTTTTTTCCCC
NC_000001.11:33 ------------------------------------------------------------
NC_000023.11:15 ------------------------------------------------------------
NC_000004.12:c1 ------------------------------------------------------------

NC_000013.11:c3 CGCTGCAGAGGAAGATTTCTTTACTGTCCCATTAACATTTCACGCTCAGTCCTGTCTCCG
NC_000001.11:33 ------------------------------------------------------------
NC_000023.11:15 ------------------------------------------------------------
NC_000004.12:c1 ------------------------------------------------------------

NC_000013.11:c3 AGTTTTTACATATATTGTATCCCCGATCAGGAACTCCTTTCCCCTGTTCTACATGATCCA
NC_000001.11:33 ------------------------------------------------------------
NC_000023.11:15 ------------------------------------------------------------
NC_000004.12:c1 ------------------------------------------------------------

NC_000013.11:c3 CCTGCCTATCCTTTGGGATCCAGCCCAGATCTCATCTTCATGAAGTTTTTCCTGATTATT
NC_000001.11:33 ------------------------------------------------------------
NC_000023.11:15 ------------------------------------------------------------
NC_000004.12:c1 ------------------------------------------------------------

NC_000013.11:c3 TTAATTTTCAGAATCTAACTATTCTGTACCTCATAATTTATAAATTATGTTATCTTTGCT
NC_000001.11:33 ------------------------------------------------------------
NC_000023.11:15 ------------------------------------------------------------
NC_000004.12:c1 ------------------------------------------------------------

NC_000013.11:c3 TTAGTGCGTATGTATAATTATACATTGTCTTTCTGTGTTACTATGTAATATTTTTAACAT
NC_000001.11:33 ------------------------------------------------------------
NC_000023.11:15 ------------------------------------------------------------
NC_000004.12:c1 ------------------------------------------------------------

NC_000013.11:c3 TATGGTTTCTTGTTTTCTATTTGTGTTAATGTTATTTTCTAAGAATAGATATTATGCTCT
```

```
NC_000001.11:33 ------------------------------------------------------------
NC_000023.11:15 ------------------------------------------------------------
NC_000004.12:c1 ------------------------------------------------------------

NC_000013.11:c3 TTATTTCCTCTTTGGCAGTGCTGAGCACATGATAAATCTTCAATAAATGTTTGTTTCTTA
NC_000001.11:33 -------------------------------------------------------TTTTA
NC_000023.11:15 -------------------------------------------------------CTTTG
NC_000004.12:c1 ------------------------------------------------------------

NC_000013.11:c3 AATAATATGATAAATAGACTGGCAGACATGCATGACAGCATATGATGGTAATTTTTGTAG
NC_000001.11:33 AATAACAACATA------------------------------------------------
NC_000023.11:15 AAAAACATTGCC------------------------------------------------
NC_000004.12:c1 ------------------------------------------------------------

NC_000013.11:c3 GAATTTGTTTGGCTCATAAATTTGTTCACTGCTGATTGATTCATTTTGCATAGTTTGAAG
NC_000001.11:33 ------------------------------------------------------------
NC_000023.11:15 ------------------------------------------------------------
NC_000004.12:c1 ------------------------------------------------------------

NC_000013.11:c3 AAGAGCCTTCCATTCTCACACTTGCTTTTTTCCAGAGATTAGTCATTATGTATTGAGCTT
NC_000001.11:33 ------------------------------------------------------------
NC_000023.11:15 ------------------------------------------------------------
NC_000004.12:c1 ------------------------------------------------------------

NC_000013.11:c3 CTAGAAGGAGTATTGTAATGGACACAGTAGCTCCCTCATTACCAGTTTGTTCCTCAGAAA
NC_000001.11:33 ------------------------------------------------------------
NC_000023.11:15 ------------------------------------------------------------
NC_000004.12:c1 ------------------------------------------------------------

NC_000013.11:c3 TATCTCTCTGTTACCCTTTTTCCAGACTCTTCAAGATAAAAGGAAATCCTATCATGTTGT
NC_000001.11:33 ------------------------------------------------------------
NC_000023.11:15 ------------------------------------------------------------
NC_000004.12:c1 ------------------------------------------------------------

NC_000013.11:c3 TGTTGATAATGATGGGGTGATACAAGAATTTGTTAATTATAATAGCCCTATGAGAATAAG
NC_000001.11:33 ------------------------------------------------------------
NC_000023.11:15 ------------------------------------------------------------
NC_000004.12:c1 ------------------------------------------------------------

NC_000013.11:c3 TAGTTCATTTGCTTGGCTATTCTGTAGTGTGTGAGCATCACATCTTAGTATTTAGCATTG
NC_000001.11:33 ------------------------------------------------------------
NC_000023.11:15 ------------------------------------------------------------
NC_000004.12:c1 ------------------------------------------------------------

NC_000013.11:c3 TAATGAACTATATTAGGAGATATAAAAGACATACCTGTACCTCAAAGAACATGCTGGTAG
NC_000001.11:33 ------------------------------------------------------------
NC_000023.11:15 ------------------------------------------------------------
NC_000004.12:c1 ------------------------------------------------------------

NC_000013.11:c3 ATACAAATCCAAGTTATTTAATAAATCACTAAAAATCCTGTGAATCTTGGTTAAGGACCC
NC_000001.11:33 ------------------------------------------------------------
NC_000023.11:15 ----------------------------------------TGAATCC-------------
NC_000004.12:c1 ------------------------------------------------------------
```

```
NC_000013.11:c3 CACTAGTCTCATTCATACACACACTTGAGAAATTCAGAAAGCTAAATATTTCTGCTGGGT
NC_000001.11:33 ------------------------------------------------------------
NC_000023.11:15 ------------------------------------------------------------
NC_000004.12:c1 ------------------------------------------------------------

NC_000013.11:c3 GTGGTGGCAGGGACCTGTAGTCCCAGCTACTTGGCAAGCTGAGGCAGGAGAATTGCTTGA
NC_000001.11:33 ------------------------------------------------------------
NC_000023.11:15 ------------------------------------------------------------
NC_000004.12:c1 ------------------------------------------------------------

NC_000013.11:c3 GGCCAGGATGCTGTAGCGTGCTGTGGTCATGCCTGCAAATAGCCACTGCACTCCAGCCTG
NC_000001.11:33 ------------------------------------------------------------
NC_000023.11:15 ------------------------------------------------------------
NC_000004.12:c1 ------------------------------------------------------------

NC_000013.11:c3 GGCAACATAGTGAGACTCCATTTCTTTAAAAAGAAAGAAAGATGACTACCTCTAATTTAA
NC_000001.11:33 ---------------------------------AAGTAAAGGTGACTA------------
NC_000023.11:15 ---------------------------------CCAAAGAAGTGACTA------------
NC_000004.12:c1 ------------------------------------------------------------

NC_000013.11:c3 AATTCCAACTTTGATGGTAAACTTTTAGCCATGGCACAGCTGAAAATTTTATTACTATAT
NC_000001.11:33 ------------------------------------------------------------
NC_000023.11:15 ------------------------------------------------------------
NC_000004.12:c1 ------------------------------------------------------------

NC_000013.11:c3 AAGATGGGAAAATCCCTTGCCACATGCTGTATTTATAAAGTTGTGAGATTATGATTTCTT
NC_000001.11:33 ------------------------------------------------------------
NC_000023.11:15 ------------------------------------------------------------
NC_000004.12:c1 ------------------------------------------------------------

NC_000013.11:c3 TGACACCAGTACTGATTTAGCCTATTGTTTTTACAAAGAAATAGTCCTGAGGGAGCACAA
NC_000001.11:33 ------------------------------------------------------------
NC_000023.11:15 ------------------------------------------------------------
NC_000004.12:c1 ------------------------------------------------------------

NC_000013.11:c3 GAGGAAGCCCTAGTTTTAATCAAGTAGAGAAGTTATTTATTGTTGGTCCCTGTTTAAAAA
NC_000001.11:33 ------------------------------------------------------------
NC_000023.11:15 ------------------------------------------------------------
NC_000004.12:c1 ------------------------------------------------------------

NC_000013.11:c3 ATAATAATCCTACAAAGTAAAGTTTTTAAAGAAAATAAATATTACATTTTAATTATAATA
NC_000001.11:33 ------------------------------------------------------------
NC_000023.11:15 ------------------------------------------------------------
NC_000004.12:c1 ------------------------------------------------------------

NC_000013.11:c3 ACTAGTGAGAAAAGAATTCTTCATAAATTTATAGCAACAAATACTTGGGGGAACAAACAT
NC_000001.11:33 ------------------------------------------------------------
NC_000023.11:15 ------------------------------------------------------------
NC_000004.12:c1 ------------------------------------------------------------

NC_000013.11:c3 TTATTCAATATATGAATATAAGAATGATAATATTAGTTATTGAGGGAGGTAGGGACAGGT
NC_000001.11:33 ------------------------------------------------------------
NC_000023.11:15 ------------------------------------------------------------
```

```
NC_000004.12:c1 ------------------------------------------------------------

NC_000013.11:c3 TTGGGAGGGAAAGTCACGAGGTTTATTTTGAACACATTAAGTTTGAGATGCCTATTAAAA
NC_000001.11:33 ------------------------------------------------------------
NC_000023.11:15 ------------------------------------------------------------
NC_000004.12:c1 ------------------------------------------------------------

NC_000013.11:c3 GTCCAAGTGGATATGTCAAGTAGATCAATTAAGAAATCATAGGAAAAAGATTGGGATATC
NC_000001.11:33 ------------------------------------------------------------
NC_000023.11:15 ------------------------------------------------------------
NC_000004.12:c1 ------------------------------------------------------------

NC_000013.11:c3 TTTTCCCAACCTTTGGGAGGCCAAGGTGGGCAGATCACCTGAGGTCAGGAGTTTAAGACC
NC_000001.11:33 ------------------------------------------------------------
NC_000023.11:15 --------------------------------------------------GCTCAAGGCC
NC_000004.12:c1 ------------------------------------------------------------

NC_000013.11:c3 AGCCTGGACACATGGTGAAACCCCATCTCTACTAAAATAGAAAAATTAGCTGGGTATGGT
NC_000001.11:33 ------------------------------------------------------------
NC_000023.11:15 ATACC-------------------------------------------------------
NC_000004.12:c1 ------------------------------------------------------------

NC_000013.11:c3 GGCACACACCTGTAATCCCAGTACTCGGGAGACTGAGGCAGGAAAATTGCTTGAGCCCAG
NC_000001.11:33 ------------------------------------------------------------
NC_000023.11:15 ------------------------------------------------------------
NC_000004.12:c1 ------------------------------------------------------------

NC_000013.11:c3 GAGGTGGAACTTGCAGTGAGCCGAGATTGCACCACTGCACTCCAGCCTGGGCAACAGAGC
NC_000001.11:33 ------------------------------------------------------------
NC_000023.11:15 ------------------------------------------------------------
NC_000004.12:c1 ------------------------------------------------------------

NC_000013.11:c3 GAAACTCCATCTCAAAAAAAAAAAAAAAAAAAAAAAAAACAAAAAAAAACCAGTGATTTCA
NC_000001.11:33 ------------------------------------------------------------
NC_000023.11:15 ------------------------------------------------------------
NC_000004.12:c1 ------------------------------------------------------------

NC_000013.11:c3 GTGTTTCAACAGATAGTATCTAAAGCAATGGGACTGATTGACATCGCCCAGGAGCAGTAC
NC_000001.11:33 ------CAACAG------------------------------------------------
NC_000023.11:15 ------------------------------------------------------------
NC_000004.12:c1 ------------------------------------------------------------

NC_000013.11:c3 AGTAAGGGGAGAAAAAAACTAGGTTCAGTCTTTGTTAATGTTCTTAACATTTAGAGGTCA
NC_000001.11:33 ------------------------------------------------------------
NC_000023.11:15 ------------------------------------------------------------
NC_000004.12:c1 ------------------------------------------------------------

NC_000013.11:c3 GATAAGGAGAAGCTGATAGAAGAACTGGCTAAAAATGTAGGAGGAAAATAACAGGGTTTC
NC_000001.11:33 ---------------------------------------AGTAGAAAATAAGAGTATGTC
NC_000023.11:15 ---------------------------------------ACCAGCCAATGGCA-------
NC_000004.12:c1 ---------------------------------------GGCGGGCAGTCGGAGG-----
                                                       .  .*  *.* . *

NC_000013.11:c3 TTAAAAGTTGAGAGTGTTTCAAGGAGGGAATTATCAGCTATGTTAAAGATTGAGTAATTA
```

```
NC_000001.11:33 ACACAAGTAGAAA-----------------------------------------------
NC_000023.11:15 ----AAGTGGAGA-----------------------------------------------
NC_000004.12:c1 ----AGGCGGAAACTG--------------------------------------------
                    *.*. **.*

NC_000013.11:c3 GCCAGGCATAGCTGTGCATGCCTGTAGTCCCAGCTGAGGTAGAAGGATCTCTTGAACTCA
NC_000001.11:33 ------------------------------------------------------------
NC_000023.11:15 ------------------------------------------------------------
NC_000004.12:c1 ------------------------------------------------------------


NC_000013.11:c3 GGGAGGTTAAGGCTGCAGTGAGCTATGATGGCATCACTGCATTCCAGCCTGGGTGACAGA
NC_000001.11:33 ------------------------------------------------------------
NC_000023.11:15 ------------------------------------------------------------
NC_000004.12:c1 ------------------------------------------------------------


NC_000013.11:c3 GCAAGACCCAGTCTCTTAAAAAATAACAATAAAAAAAATTAGAAAACAGGAGGTGTCTGC
NC_000001.11:33 ------------------------------------------------------------
NC_000023.11:15 ------------------------------------------------------------
NC_000004.12:c1 ------------------------------------------------------------


NC_000013.11:c3 TGGATTTGGCAGCATGAACGTCATTGATGGCCTTCATAAAAGCAGTTTTAGTGGAGAGTA
NC_000001.11:33 ------------------------------------------------------------
NC_000023.11:15 ------------------------------------------------------------
NC_000004.12:c1 ------------------------------------------------------------


NC_000013.11:c3 CTGAGAACCGATGCCAGATTGGAGTGGGCTAAAGATAAGTGGATCAAGAGGAAACAAGCA
NC_000001.11:33 ------------------------------------------------------------
NC_000023.11:15 ------------------------------------------------------------
NC_000004.12:c1 ------------------------------------------------------------


NC_000013.11:c3 ACTCCTGAGAAGTTTTGTGGTCAAGAGGAAAGAAATGGGCCAAGGGTTGGAGACCACAGT
NC_000001.11:33 ------------------------------------------------------------
NC_000023.11:15 ------------------------------------------------------------
NC_000004.12:c1 ------------------------------------------------------------


NC_000013.11:c3 GACAGGACAGTTTTTTCCCCTTAGTCTGGGATTGTAGATTGTATAGAGCCTGTTTACATA
NC_000001.11:33 ------------------------------------------------------------
NC_000023.11:15 ------------------------------------------------------------
NC_000004.12:c1 ------------------------------------------------------------


NC_000013.11:c3 CGTGTATTCATGGGAGTGAGCTAGTAGTTAGTCATAAACCTGCAGATATCAGAGCTTTGT
NC_000001.11:33 ------------------------------------------------------------
NC_000023.11:15 ------------------------------------------------------------
NC_000004.12:c1 ------------------------------------------------------------


NC_000013.11:c3 GATGTTAAAAAAGATATTTATACTTTTTTAAGTCCCTGCAGAATCAACTATATACTTTTT
NC_000001.11:33 ------------------------------------------------------------
NC_000023.11:15 ------------------------------------------------------------
NC_000004.12:c1 ------------------------------------------------------------


NC_000013.11:c3 ACTTTTTTTTTTTGAGATGGAGTCTCTGTTACCCAGGCTGGAGTTCGATCTTGGCTGACT
NC_000001.11:33 ------------------------------------------------------------
NC_000023.11:15 ------------------------------------------------------------
NC_000004.12:c1 ------------------------------------------------------------
```

```
NC_000013.11:c3 GCAACCTCCACCTCCCAGGTTCAAGCGATTCTCCCCCCTCAGCCTCCCGAGTAGCTGGGA
NC_000001.11:33 ------------------------------------------------------------
NC_000023.11:15 ------------------------------------------------------------
NC_000004.12:c1 ------------------------------------------------------------

NC_000013.11:c3 TTACAGGCATGCGCCACCACTCCCGGCTTATTTTGTATTTTGCATTTTTCACTGTGTTAG
NC_000001.11:33 ------------------------------------------------------------
NC_000023.11:15 ------------------------------------------------------------
NC_000004.12:c1 ------------------------------------------------------------

NC_000013.11:c3 CCAGGCTGTTCTTGAATTCCTGACCTTAAGTGATCTGCCCACCTTGGCCTCCCAAAGTGC
NC_000001.11:33 ------------------------------------------------------------
NC_000023.11:15 ------------------------------------------------------------
NC_000004.12:c1 ------------------------------------------------------------

NC_000013.11:c3 TGGGATTACAAGCATGAACCACCATGCCCACCCCTGTACTTTTCTTTATAATGGATTTAA
NC_000001.11:33 ------------------------------------------------------------
NC_000023.11:15 ------------------------------------------------------------
NC_000004.12:c1 ------------------------------------------------------------

NC_000013.11:c3 CATTTCAGCAATGCATTGGGTTTTTTTGTTTGTTTGTTTTGTTTTCTGTTTTTTGTGTTT
NC_000001.11:33 ------------------------------------------------------------
NC_000023.11:15 ------------------------------------------------------------
NC_000004.12:c1 ------------------------------------------------------------

NC_000013.11:c3 TTTTGAAACTGGGTCTCTGTCACTCAGGGCTGGAGTGCAGTGGCGCGGGCTACCCAGGGC
NC_000001.11:33 ------------------------------------------------------------
NC_000023.11:15 ------------------------------------------------------------
NC_000004.12:c1 ------------------------------------------------------------

NC_000013.11:c3 TCACTGCAGTCTCTACCTTCCAGGCTCAGGTGACCCTCCCACCTTAGCCTCCTGAGTAGC
NC_000001.11:33 ------------------------------------------------------------
NC_000023.11:15 ------------------------------------------------------------
NC_000004.12:c1 ------------------------------------------------------------

NC_000013.11:c3 TGGGACTACAGGTGCATGCCACCATGCCTGGCTAATTTTTTTTTTTTTTTAGATGAGGTA
NC_000001.11:33 ------------------------------------------------------------
NC_000023.11:15 ------------------------------------------------------------
NC_000004.12:c1 ------------------------------------------------------------

NC_000013.11:c3 TCATTCTGTAGCCCAGGCTGGAGTGCAGTGACACAATCACGGCTCACTGCAACCTCAATC
NC_000001.11:33 ------------------------------------------------------------
NC_000023.11:15 ------------------------------------------------------------
NC_000004.12:c1 ------------------------------------------------------------

NC_000013.11:c3 TCCTGGGGTCAAGCAGTCCTCCCACCTCTCAGCCTCCTGAGTAGCTGGGACTATAGATGT
NC_000001.11:33 ------------------------------------------------------------
NC_000023.11:15 ------------------------------------------------------------
NC_000004.12:c1 ------------------------------------------------------------

NC_000013.11:c3 CTGCTATTATACCCAGCTAATTTTTGTGTTTTTTATAGAGATGGGGTTTTGCCATGTTGG
NC_000001.11:33 ------------------------------------------------------------
NC_000023.11:15 ------------------------------------------------------------
```

```
NC_000004.12:c1 ------------------------------------------------------------

NC_000013.11:c3 CCAGGCTGGTCTCGAATTCCTGGTATCAAGTGATCCTCCCTCCTCGGCCTTCCACTATGC
NC_000001.11:33 ------------------------------------------------------------
NC_000023.11:15 ------------------------------------------------------------
NC_000004.12:c1 ------------------------------------------------------------

NC_000013.11:c3 TAGGATTACAGGCGTGAACCACTGCACCCAGCCTGGATTGCTTTTTATTAATGAAGAAGA
NC_000001.11:33 ------------------------------------------------------------
NC_000023.11:15 ------------------------------------------------------------
NC_000004.12:c1 ------------------------------------------------------------

NC_000013.11:c3 GTTCAGTGCATTCTGTCTAGTGATAAATACATATTGCTAGATAAAATTGTGTTAATTGTA
NC_000001.11:33 ------------------------------------------------------------
NC_000023.11:15 ------------------------------------------------------------
NC_000004.12:c1 ------------------------------------------------------------

NC_000013.11:c3 AGCATTTTTAGAAGCTATCTGGGTGCTAAAACAATGTAAGAATTCTGGTTGACCAAAATA
NC_000001.11:33 ------------------------------------------------------------
NC_000023.11:15 ------------------------------------------------------------
NC_000004.12:c1 ------------------------------------------------------------

NC_000013.11:c3 GTAAATATTTTATTTAAATAATTAGAAAATACTGCAAAAATCAGTAATTAATTACATTTC
NC_000001.11:33 ------------------------------------------------------------
NC_000023.11:15 ------------------------------------------------------------
NC_000004.12:c1 ------------------------------------------------------------

NC_000013.11:c3 AAAGTGTTCATTTTGCTGAGAAAATGAAATTATATAAGTAAAATGTGTCATAGCTAGTCA
NC_000001.11:33 ------------------------------------------------------------
NC_000023.11:15 ------------------------------------------------------------
NC_000004.12:c1 ------------------------------------------------------------

NC_000013.11:c3 ATAAACAAATTTTTATTAAGTACTTGGTATGTTCCAGGAACTGTCTTAGGTGCTGATGAT
NC_000001.11:33 ------------------------------------------------------------
NC_000023.11:15 ------------------------------------------------------------
NC_000004.12:c1 ------------------------------------------------------------

NC_000013.11:c3 ACAGGAATGAAACAGTCTCTGCCCTCAAGGAATGCTGACATTTTAGGAGGTTGGGGAAAG
NC_000001.11:33 ------------------------------------------------------------
NC_000023.11:15 ------------------------------------------------------------
NC_000004.12:c1 ------------------------------------------------------------

NC_000013.11:c3 GCAACAAGCACATACTAAGGAAATCTTTATGTGCTGAAATGTACTATGGAGAAAATAAAT
NC_000001.11:33 ------------------------------------------------------------
NC_000023.11:15 ------------------------------------------------------------
NC_000004.12:c1 ------------------------------------------------------------

NC_000013.11:c3 TGGAATAATGTAGCAGAGAATGACTGGGATAAATACTTTCGCTAAGTTGTCAAGAAAAGC
NC_000001.11:33 ------------------------------------------------------------
NC_000023.11:15 ------------------------------------------------------------
NC_000004.12:c1 ------------------------------------------------------------

NC_000013.11:c3 CTCTTTGAGCTAAGATTATATAATGGGTGGAGGAGATTCAAGTCAGAGAAAAGAAAAAGT
```

```
NC_000001.11:33 ------------------------------------------------------------
NC_000023.11:15 -------------------------------------TCAAGTCAAA-------------
NC_000004.12:c1 ------------------------------------------------------------

NC_000013.11:c3 GCACATGTTCTGAGGTAGGATTGTGTTTGGCTGGTAAAAGGAAGATAAAAAGGTCTTGTG
NC_000001.11:33 ------------------------------------------------------------
NC_000023.11:15 ------------------------------------------------------------
NC_000004.12:c1 ------------------------------------------------------------

NC_000013.11:c3 GCTGAAAGATGGAGTAATCTGGCAATAGAATGGAAGGAGCTGTTCAGAGACTTAGTTGGC
NC_000001.11:33 ------------------------------------------------------------
NC_000023.11:15 ------------------------------------------------------------
NC_000004.12:c1 ------------------------------------------------------------

NC_000013.11:c3 ATGAGATTATTTGAGCCTTGTTGATTAGGGTAAAGAAGTTGGATTTGGGTTTATTTATTT
NC_000001.11:33 ------------------------------------------------------------
NC_000023.11:15 ------------------------------------------------------------
NC_000004.12:c1 ------------------------------------------------------------

NC_000013.11:c3 ATTATTTTATTTTATTTTGTTTCCTTCCTTCCTTTCTTCCTCTCTCTCTCTCTCTCTTCT
NC_000001.11:33 ------------------------------------------------------------
NC_000023.11:15 ------------------------------------------------------------
NC_000004.12:c1 ------------------------------------------------------------

NC_000013.11:c3 TTTCCTTTCCTTCTTTCTTTTTGTTCATTCATTCATTCTTTTCTTTTCTCTTCTTTAGAG
NC_000001.11:33 ------------------------------------------------------------
NC_000023.11:15 --------------TCCCATTCATTCATTCATTCACTCATTCATTCCCGAAGCTTTA---
NC_000004.12:c1 ------------------------------------------------------------

NC_000013.11:c3 ACAAGGTCTCACTCTCTCCAGACCAGAGCACCGTGGTGCTGTCATAGCTCACTGGAGCCT
NC_000001.11:33 ------------------------------------------------------------
NC_000023.11:15 ------------------------------------------------------------
NC_000004.12:c1 ------------------------------------------------------------

NC_000013.11:c3 CAAATTCCTAGGTTCAAGTGATCCTCCTCCCTCAGCCTCTCAAGTAGTTAAGACAGGACT
NC_000001.11:33 ------------------------------------------------------------
NC_000023.11:15 ------------------------------------------------------------
NC_000004.12:c1 ------------------------------------------------------------

NC_000013.11:c3 AAGGATTTCTTCTTTCTTTCTTTCTTTCTTTCTTTCTTTTTTTTTTTTTTTTTGAGACAG
NC_000001.11:33 ------------------------------------------------------------
NC_000023.11:15 ------------------------------------------------------------
NC_000004.12:c1 ------------------------------------------------------------

NC_000013.11:c3 AGTCTCGCTCGCTCTGTCACCAGGCAATCTTGGCTCACTGCAACCTTCGCCTCCCGGGTT
NC_000001.11:33 ------------------------------------------------------------
NC_000023.11:15 ------------------------------------------------------------
NC_000004.12:c1 ------------------------------------------------------------

NC_000013.11:c3 CAAGCAATTCTCCTGCCTCAGCCTCCCAAGTACCTGAGACTACAGGTGCATGCCACCATG
NC_000001.11:33 ------------------------------------------------------------
NC_000023.11:15 ------------------------------------------------------------
NC_000004.12:c1 ------------------------------------------------------------
```

```
NC_000013.11:c3 CCCAGCTAATTTTTGTATTTTTAGTAGAGATGGGGTTTCACCATGTTGGCCAGGATGGTC
NC_000001.11:33 ------------------------------------------------------------
NC_000023.11:15 ------------------------------------------------------------
NC_000004.12:c1 ------------------------------------------------------------

NC_000013.11:c3 TCGATCTCTTGACCTCATGATCCAGGAGGCCTGCCTTGGCCTCCCAAAGTGATGGGATTA
NC_000001.11:33 ------------------------------------------------------------
NC_000023.11:15 ------------------------------------------------------------
NC_000004.12:c1 ------------------------------------------------------------

NC_000013.11:c3 CAGGTGTGAGCCACCTTGCCCTGCTTCTTTTTTTTTTTTTTTTTTTTTTTGGAGACCAGG
NC_000001.11:33 ---------------------------TTTTTTATCTCTATATCTTCTCTG---------
NC_000023.11:15 ---------------------------ATTCTTTGCTCTAGGGTGTGTCAGGAGAGCA--
NC_000004.12:c1 ------------------------------------------------------------

NC_000013.11:c3 TCTCACTTCATCCAGGCTGAAGTGTAGTGGCATAATCACAGCTCACTGCTGCCTTGACCT
NC_000001.11:33 ------------------------------------------------------------
NC_000023.11:15 ------------------------------------------------------------
NC_000004.12:c1 ------------------------------------------------------------

NC_000013.11:c3 CCAGGGCTCAAGTGATCCTCCCAGCTCAGCCGCCCGAGTAGCTGGGACTATAGGTGCACC
NC_000001.11:33 ------------------------------------------------------------
NC_000023.11:15 ------------------------------------------------------------
NC_000004.12:c1 ------------------------------------------------------------

NC_000013.11:c3 ACCATGCCCAGCTAATTCTTGTATTTTTTGTAGAGATGTAGTTTCACCATGTTGTCTAAG
NC_000001.11:33 ------------------------------------------------------------
NC_000023.11:15 ------------------------------------------------------------
NC_000004.12:c1 ------------------------------------------------------------

NC_000013.11:c3 CTGATGTTGAGCTCCTGGCCTCAAGTGATCCTCCCATCTTGGTCTCCCAAAGTGCTGAGA
NC_000001.11:33 ------------------------------------------------------------
NC_000023.11:15 ------------------------------------------------------------
NC_000004.12:c1 ------------------------------------------------------------

NC_000013.11:c3 TTACAGGCATGAACCATCACACCTGGCCAGGTTGTTTGCTTAATGTGAATTCATTGAAGG
NC_000001.11:33 ------------------------------------------------------------
NC_000023.11:15 ------------------------------------------------------------
NC_000004.12:c1 ------------------------------------------------------------

NC_000013.11:c3 GTTTAATCAAGGAAGGGGCATTATTAGATTTATGCTTAGAAAGATCAGTCAGGGTTAGGC
NC_000001.11:33 ------------------------------------------------------------
NC_000023.11:15 ------------------------------------------------------------
NC_000004.12:c1 ------------------------------------------------------------

NC_000013.11:c3 ACAGTGGCTCATACTTGAAATCCTATCACTTTGGGAGGTTGAGGTGGGAGGATCCCTTGA
NC_000001.11:33 ------------------------------------------------------------
NC_000023.11:15 --------------------------------GAGGGAATGAGG---------GCCTTGA
NC_000004.12:c1 -----------------------------------------------------CCCCTGA

NC_000013.11:c3 AGCCAGGAGTTCAAGACCAGCCTGGGCAACATGGCAAGACCCCGTCTCTACAAAAAATTG
NC_000001.11:33 ------------------------------------------------------------
NC_000023.11:15 ACCGAGACAGTGAAGA--------------------------------------------
```

```
NC_000004.12:c1 ------------------------------------------------------------


NC_000013.11:c3 AAAAAAAAAATAGATCAGTATGGATGCTGTGTGGAATGGAAGCAAGAAGACTCATTCAGA
NC_000001.11:33 ------------------------------------------------------------
NC_000023.11:15 ------------------------------------------------------------
NC_000004.12:c1 ------------------------------------------------------------


NC_000013.11:c3 GTCATCTACAGTAGTACAAGTGGGAGATTATGAGATTTCAGACTAAAGTTGTAGCAGCAG
NC_000001.11:33 ------------------------------------------------------------
NC_000023.11:15 ------------------------------------------------------------
NC_000004.12:c1 ------------------------------------------------------------


NC_000013.11:c3 AGATAGTAGGATATGTGTTAGATTTAGGTTATATTTGGGAGCCAATGCTGACAGGATGTA
NC_000001.11:33 ------------------------------------------------------------
NC_000023.11:15 GGGTAAGAAGAAAAGTGTTATTCTTTGCCCACA---------------------------
NC_000004.12:c1 ------------------------------------------------------------


NC_000013.11:c3 CTGATGGGTTGGATGTGGGGTGCTGATGGATTGCATATGGGATGTAAGGGAAATGGAATC
NC_000001.11:33 ------------------------------------------------------------
NC_000023.11:15 ------------------------------------------------------------
NC_000004.12:c1 ------------------------------------------------------------


NC_000013.11:c3 GTCAAATTCAGCAGAAACATTTGTTAAATACCTGTCGTTACGTGGGGTTATATTAGATAA
NC_000001.11:33 ------------------------------------------------------------
NC_000023.11:15 ------------------------------------------------------------
NC_000004.12:c1 ------------------------------------------------------------


NC_000013.11:c3 CAAATATACCAATTGATTTAAACAGCTATGGCCCTTCTTCTTGGACTGTAATTTTTCATC
NC_000001.11:33 ------------------------------------------------------------
NC_000023.11:15 ------------------------------------------AAACTACAATTTCTCACA
NC_000004.12:c1 ------------------------------------------------------------


NC_000013.11:c3 ACTTCTTCCCATTTTGGTGGATGGTTTTTGTTTTTTTTTTTTAGATGGAGTCTTGCTCTG
NC_000001.11:33 ------------------------------------------------------------
NC_000023.11:15 AATTC-------------------------------------------------------
NC_000004.12:c1 ------------------------------------------------------------


NC_000013.11:c3 TCACCCAGGCTGGAGTGCAGTGGCACGATCTCAGCTCACTGCAACCTCCACCTCCCGGGT
NC_000001.11:33 ------------------------------------------------------------
NC_000023.11:15 ------------------------------------------------------------
NC_000004.12:c1 ------------------------------------------------------------


NC_000013.11:c3 TCATGCCATTCTCCGGCCTCAGCCTCCCGAGTAGCTGGGACCACAGGCACCCGCCACCAC
NC_000001.11:33 ------------------------------------------------------------
NC_000023.11:15 ------------------------------------------------------------
NC_000004.12:c1 ------------------------------------------------------------


NC_000013.11:c3 GCCCGGTTAATTTTTTGTATTTTTAGTGGAGACGGGGTTTCACCGTGTCAGCCAGGATGG
NC_000001.11:33 ------------------------------------------------------------
NC_000023.11:15 ------------------------------------------------------------
NC_000004.12:c1 ------------------------------------------------------------


NC_000013.11:c3 TCTCAATCTCCTGACCTCGTGATCTGCCCGCCTCGGACTCCCAAAGTGCTGGGATTACAG
```

```
NC_000001.11:33 ------------------------------------------------------------
NC_000023.11:15 ------------------------------------------------------------
NC_000004.12:c1 ------------------------------------------------------------


NC_000013.11:c3 GCATGAGCCACCACGCCCGGCTGGTGGATGTTTTTTAAACCACCTAAATGATATCATAAT
NC_000001.11:33 ------------------------------------------------------------
NC_000023.11:15 ------------------------------------------------------------
NC_000004.12:c1 ------------------------------------------------------------


NC_000013.11:c3 TTTCATTCTAGCTTTTAAAATAATGTATTTTTAAATAATGTGTTTTTAAAGATTTTGATT
NC_000001.11:33 ------------------------------------------------------------
NC_000023.11:15 ------------------------------------------------------------
NC_000004.12:c1 ------------------------------------------------------------


NC_000013.11:c3 GCAAAATGAAAGAAACAAGGATAAGTTGTGCTAATTCCAATAAAGTATGGAGGCCATGGT
NC_000001.11:33 ------------------------------------------------------------
NC_000023.11:15 ------------------------------------------------------------
NC_000004.12:c1 ------------------------------------------------------------


NC_000013.11:c3 TTTAGCAACATTCCTGGATCTCCTCTTGCTTAAGTGTCCAGCTCCTGGCACACGGAAACA
NC_000001.11:33 ------------------------------------------------------------
NC_000023.11:15 ------------------TCTGCTCTTGCCAAA---------------------------
NC_000004.12:c1 ------------------------------------------------------------


NC_000013.11:c3 TCCCACATTAAATCTTCCTTAGGTCTGAATCACCCCATATCTTTGACTGATGTCTTGTTT
NC_000001.11:33 -------------------------------------------------------TGCTT
NC_000023.11:15 ----------------------------------------------------TTTTGTTT
NC_000004.12:c1 ------------------------------------------------------------


NC_000013.11:c3 TGCTACCTTCCTGATGCTGAGTTCACTTTTAGTGCCCATTTGTTCACCCTCACCTCTATT
NC_000001.11:33 TGCTACCTTCAT----CTGAATTCACT---------------------------------
NC_000023.11:15 TCCTCTTTTAGC-CCGTCGGTTCCGCA---------------------------------
NC_000004.12:c1 ----------------CCGGGCCCGGT---------------------------------
                                ..*. ..*.

NC_000013.11:c3 TTCAGATTATATTCTGTTTGCCTTCTCTACCATGATACAGTGATTCTATCAGATAATCAT
NC_000001.11:33 ------------------------------------------------------------
NC_000023.11:15 ------------------------------------------------------------
NC_000004.12:c1 ------------------------------------------------------------


NC_000013.11:c3 AAAACACTTCCTTCAGTAAGTCCTGGGACACTTTTACCCCCATATCTCCCTACCTTCCCA
NC_000001.11:33 ------------------------------------------------------------
NC_000023.11:15 ------------------------------------------------------------
NC_000004.12:c1 ------------------------------------------------------------


NC_000013.11:c3 GAAAGTCTTGAGACTTACTGAAGAAAGAAATCTTTGCCAAGGAGAACCAGGTATAGAAGC
NC_000001.11:33 ------------------------------------------------------------
NC_000023.11:15 ------------------------------------------------------------
NC_000004.12:c1 ------------------------------------------------------------


NC_000013.11:c3 ATGGTTACTAAAGATGAAATGCATAGCTGAGCACTTGTTCACAAGGGGAAATGGACTCAA
NC_000001.11:33 ------------------------------------------------------------
NC_000023.11:15 ------------------------------------------------------------
NC_000004.12:c1 ------------------------------------------------------------
```

```
NC_000013.11:c3 AACTTTTCCTGCAGAAAATTCTGACTTTGCCTGCAGGTGTCTCCCTCCTCGTTAAGATGG
NC_000001.11:33 ------------------------------------------------------------
NC_000023.11:15 ------------------------------------------------------------
NC_000004.12:c1 ------------------------------------------------------------

NC_000013.11:c3 CCTTCTATCAAAGCAAGAGTCCTGATTGGGAACCCCAATTTCCATTATATTAGTAGATGA
NC_000001.11:33 ------------------------------------------------------------
NC_000023.11:15 ------------------------------------------------------------
NC_000004.12:c1 ------------------------------------------------------------

NC_000013.11:c3 TCAGCTTTACCACCTGCTGGTCTAACTTCTAATACTTGTGGTTCTGTTCTTAATCAACAA
NC_000001.11:33 ------------------------------------------------------------
NC_000023.11:15 ------------------------------------------------------------
NC_000004.12:c1 ------------------------------------------------------------

NC_000013.11:c3 TATAGAATACTTGTACACTCCAAGGATCAGCAAACTTTCTGTAAAAGGTCAGATGATAAG
NC_000001.11:33 ------------------------------------------------------------
NC_000023.11:15 ------------------------------------------------------------
NC_000004.12:c1 ------------------------------------------------------------

NC_000013.11:c3 TATTTTAGTCTTTGTTAGCCATGTGACCCCTGCCCCAACCACCCAGCTCTGCTGCTGTAG
NC_000001.11:33 ------------------------------------------------------------
NC_000023.11:15 ------------------------------------------------------------
NC_000004.12:c1 ------------------------------------------------------------

NC_000013.11:c3 CGCAAAAGCAGCCATTGACAGTGTGAAAATGAGTGGAAATGACCGTATTCTAATAAAACT
NC_000001.11:33 ------------------------------------------------------------
NC_000023.11:15 ------------------------------------------------------------
NC_000004.12:c1 ------------------------------------------------------------

NC_000013.11:c3 TTGCAAAAATGAGCAGCAGGCAGGATTCAGCCTGTGGGCCATTATTTGCTATCCCCTTCT
NC_000001.11:33 ------------------------------------------------------------
NC_000023.11:15 ------------------------------------------------------------
NC_000004.12:c1 ------------------------------------------------------------

NC_000013.11:c3 CTAGCCTTTGTTAGACAAACTGTTAGTCTGTTCAGCTATCATCAAAAGCTGGGCTGAGAA
NC_000001.11:33 ------------------------------------------------------------
NC_000023.11:15 ------------------------------------------------------------
NC_000004.12:c1 ------------------------------------------------------------

NC_000013.11:c3 AAGTGCTATTTATGGAATATGATAAGAAGGGCCCACTGTATGCAGTGGATCTAGAGCAGA
NC_000001.11:33 ------------------------------------------------------------
NC_000023.11:15 ------------------------------------------------------------
NC_000004.12:c1 ------------------------------------------------------------

NC_000013.11:c3 TTATAGATCTACTCTAAGTAATCTAAGTTATAAGAATCACTTTCTGGATCTGATTCCCTC
NC_000001.11:33 ------------------------------------------------------------
NC_000023.11:15 ------------------------------------------------------------
NC_000004.12:c1 ------------------------------------------------------------

NC_000013.11:c3 ATTTGTTTGTTAAAATTCTTTTTTTTCTTTTTGTATTAGGCAGGTAGACCTACTGCTGTA
NC_000001.11:33 ------------------------------------------------------------
NC_000023.11:15 ------------------------------------------------------------
```

```
NC_000004.12:c1 ------------------------------------------------------------


NC_000013.11:c3 AGTCTGCTTTGTCTACAGTAGAAATGCCAAATAGGCTGGCATGGTGACTCATGCCTGTAA
NC_000001.11:33 ------------------------------------------------------------
NC_000023.11:15 ------------------------------------------------------------
NC_000004.12:c1 ------------------------------------------------------------


NC_000013.11:c3 TCCCACCTCTTTGGCAAGAGGATTCCTTGAGCCCAGGACTTCAAGGTTATAGTGAGCTAT
NC_000001.11:33 ------------------------------------------------------------
NC_000023.11:15 ------------------------------------------------------------
NC_000004.12:c1 ------------------------------------------------------------


NC_000013.11:c3 GATGCACTCCAGCCTAGGTGACAGAGTGAGACCCTGTCCCAGAAAGAAAATGCCCAGTAG
NC_000001.11:33 ------------------------------------------------------------
NC_000023.11:15 -------------------------------------------------ATTTCGGGCGC
NC_000004.12:c1 ------------------------------------------------------------


NC_000013.11:c3 GAAGAATGGCCTCCTATAGCTCCTGATTTCAAGGAAAGGGTGTCTCAATTCTAACGACCA
NC_000001.11:33 ------------------------------------------------------------
NC_000023.11:15 AAATAAAGACCTTC----------------------------------------------
NC_000004.12:c1 ------------------------------------------------------------


NC_000013.11:c3 ATGAGGATTAGCTACATGGAAAAATACAAGCAGTGGATAAAGACAAGCTATTGAGCAAAA
NC_000001.11:33 ------------------------------------------------------------
NC_000023.11:15 ------------------------------------------------------------
NC_000004.12:c1 ------------------------------------------------------------


NC_000013.11:c3 CATGATAGAAATTCTCAGAAAGGCTTATCTTTGCTCTTTCTTGCCTCTTTATGTATCATT
NC_000001.11:33 ------------------------------------------------------------
NC_000023.11:15 ------------------------------------------------------------
NC_000004.12:c1 ------------------------------------------------------------


NC_000013.11:c3 CTAGTCTCCAATTTTATTCAACTAATAGTTATTAAATGTCTGTTACGTGCAAGGCATGGT
NC_000001.11:33 ------------------------------------------------------------
NC_000023.11:15 ------------------------------------------------------------
NC_000004.12:c1 ------------------------------------------------------------


NC_000013.11:c3 GCTATGCATTGTCTCCTGCGAATATCCTTCTATTAGAAAAAAATGCCTGATATAAATGAT
NC_000001.11:33 ----------------------TATCCTTGTATGGAGATAA-------------------
NC_000023.11:15 ----------------------TGGCATTTAGTGGAAAGAG-------------------
NC_000004.12:c1 ------------------------------------------------------------


NC_000013.11:c3 TGGTATCTGTTTTCCTGCCATATATAGTCCATTCCCTGGCATGTAGGAGATGCTCAATAA
NC_000001.11:33 ------------------------------------------------------------
NC_000023.11:15 ------------CCTAGTTGAGCACGATCCGC---------------AGAAGCGGAAT--
NC_000004.12:c1 ------------------------------------------------------------


NC_000013.11:c3 ATATTTGTGGCTAAATTAATATCTCACATTATAAATCAATAAATTTCTTTTTTTTGGTAA
NC_000001.11:33 ----------------------------------------------------------AA
NC_000023.11:15 ---------------------------------------------------------CAG
NC_000004.12:c1 ------------------------------------------------------------


NC_000013.11:c3 ATTGCTCTCCTGTGAATTCCATTATCATGCATCAGTGGATGAATTTTTAAATAAAACAAG
```

```
NC_000001.11:33 ATAGCCCTCCCATAGGCTT-----------------------------------------
NC_000023.11:15 GTGGCTTTCGGATAGGC-------------------------------------------
NC_000004.12:c1 ------------------------------------------------------------

NC_000013.11:c3 CCATAGCTGATGTCTAACTGTGAGCCCAAGGTGATTCCAGATAGCCTGGGATGTCTAATG
NC_000001.11:33 ------------------------------------------------------------
NC_000023.11:15 ------------------------------------------------------------
NC_000004.12:c1 ------------------------------------------------------------

NC_000013.11:c3 ATTTTAACAAGATTACCAAATATCTTTTTCTTTCTCGTAGTGCCACATCCAGCACTTTAA
NC_000001.11:33 ------------------------------------------------------------
NC_000023.11:15 ------------------------------------------------------------
NC_000004.12:c1 ------------------------------------------------------------

NC_000013.11:c3 GTTAAATAAATGCAATATAGGGATTTAGGAGAAAAGAAGTGAGGGCTAGAAACAGGCAAC
NC_000001.11:33 ------------------------------------------------------------
NC_000023.11:15 ------------------------------------------------------------
NC_000004.12:c1 ------------------------------------------------------------

NC_000013.11:c3 TATTTGCTGGCTAACAAAGCAGAACTTATATTTCTTTTTGAACACCTACTAAGTTCTTGT
NC_000001.11:33 ------------------------------------------------------------
NC_000023.11:15 ------------------------------------------------------------
NC_000004.12:c1 ------------------------------------------------------------

NC_000013.11:c3 TTCTGATGTTGCACTAGAACTTTTTAAATGGAAATACTGTGAAATTGTAGTTATTTATAT
NC_000001.11:33 ------------------------------------------------------------
NC_000023.11:15 ------------------------------------------------------------
NC_000004.12:c1 ------------------------------------------------------------

NC_000013.11:c3 TTACAGATGCAAAACTGAATTGAAGAGAGATTAAGTAATTTATTTGAGGGAAGCCCTGAT
NC_000001.11:33 ------------------------------------------------------------
NC_000023.11:15 ------------------------------------------------------------
NC_000004.12:c1 ------------------------------------------------------------

NC_000013.11:c3 TGTCTCACTGTCTCTTTGTATCAGAAATAAAGTTGTGGTTTAGGAGACTGATAGTTAGCC
NC_000001.11:33 ------------------------------------------------------------
NC_000023.11:15 ------------------------------------------------------------
NC_000004.12:c1 ------------------------------------------------------------

NC_000013.11:c3 TGGTGTCCTATTTCTTTCGTCTTAAGTAAGATTGTATATTCACTTGTATTATTTGTCATT
NC_000001.11:33 ------------------------------------------------------------
NC_000023.11:15 ------------------------------------------------------------
NC_000004.12:c1 ------------------------------------------------------------

NC_000013.11:c3 TTCTCCCAATTGAGCGACTTCATTATTGTACCAGATGAACATGACTTTGTAAATAAGTCT
NC_000001.11:33 ------------------------------------------------------------
NC_000023.11:15 ------------------------------------------------------------
NC_000004.12:c1 ------------------------------------------------------------

NC_000013.11:c3 TCATTCATTTTAAGCAGTAGTCATTTTTTATTTCACTGTAATCTATCTTTCTGAGATGCC
NC_000001.11:33 ------------------------------------------------------------
NC_000023.11:15 ------------------------------------------------------------
NC_000004.12:c1 ------------------------------------------------------------
```

```
NC_000013.11:c3 AGCAATGACCTTTTAACCCCAGTTAGTATCAACTATCAATGTAAGCACATTTGACTGAAG
NC_000001.11:33 ------------------------------------------------------------
NC_000023.11:15 ------------------------------------------------------------
NC_000004.12:c1 ------------------------------------------------------------

NC_000013.11:c3 AAACTAATTTTTAACTCTCTTATGTGCCAGGTACCATGTTGTTATCATCATCATTGCTAA
NC_000001.11:33 ------------------------------------------------------------
NC_000023.11:15 ------------------------------------------------------------
NC_000004.12:c1 ------------------------------------------------------------

NC_000013.11:c3 GATTTATTGAATAAATACTAAGTGCCAGGCATTTACGTACGTTCTCTTATGTATTCCTCA
NC_000001.11:33 ------------------------------------------------------------
NC_000023.11:15 ------------------------------------------------------------
NC_000004.12:c1 ------------------------------------------------------------

NC_000013.11:c3 CAATACTCTGTGAGGAAGATCCCATTACTGTCCTCAGGGTGACTTTAGTGAGGAAACTAA
NC_000001.11:33 ------------------------------------------------------------
NC_000023.11:15 ------------------------------------------------------------
NC_000004.12:c1 ------------------------------------------------------------

NC_000013.11:c3 AACTTGTGGGTAAGGTTAAATGTTTTGTAAAGTATCAGAGCAAGCTATGATATGAATTCA
NC_000001.11:33 ------------------------------------------------------------
NC_000023.11:15 ------------------------------------------------------------
NC_000004.12:c1 ------------------------------------------------------------

NC_000013.11:c3 GTCTATCTAACACATTTACTTTTCACATCTCATTTAATCTCCCCAAAATGCTTCATGGTA
NC_000001.11:33 ------------------------------------------------------------
NC_000023.11:15 ------------------------------------------------------------
NC_000004.12:c1 ------------------------------------------------------------

NC_000013.11:c3 GACTTATATTATAGCTGAGGTTATTACGAAAATAATAAATTGTACTAAACTAAGAAATGA
NC_000001.11:33 ------------------------------------------------------------
NC_000023.11:15 ------------------------------------------------------------
NC_000004.12:c1 ------------------------------------------------------------

NC_000013.11:c3 CCATATGCAGTGTTTTCCAAGCCTCTTTTAAAACCACGTTCTACAATGGAACATGCGTTT
NC_000001.11:33 ------------------------------------------------------------
NC_000023.11:15 ------------------------------------------------------------
NC_000004.12:c1 ------------------------------------------------------------

NC_000013.11:c3 TACATTCCAGTCCAATATACAGGTACACATGTGTGTGACAGAAACAAAAATTGTGCCCTG
NC_000001.11:33 ------------------------------------------------------------
NC_000023.11:15 ------------------------------------------------------------
NC_000004.12:c1 ------------------------------------------------------------

NC_000013.11:c3 TGATTTTTTTACTATCCTCTTTTTCATAATGGATCATGGCCTATAGCGTGAAAAGTGATG
NC_000001.11:33 ------------------------------------------------------------
NC_000023.11:15 ------------------------------------------------------------
NC_000004.12:c1 ------------------------------------------------------------

NC_000013.11:c3 ATATAATGTGATTCTGTCTTCAGTATATATTTTTAGTATGACAGTATGATATACAGTAAG
NC_000001.11:33 ------------------------------------------------------------
NC_000023.11:15 ------------------------------------------------------------
```

```
NC_000004.12:c1 ------------------------------------------------------------

NC_000013.11:c3 CCAGAGTTTCTTCTCACTTTTTCTAGCCATTCCGTTCATTAACTTATTTAAAAATTACAA
NC_000001.11:33 ------------------------------------------------------------
NC_000023.11:15 ------------------------------------------------------------
NC_000004.12:c1 ------------------------------------------------------------

NC_000013.11:c3 ACATATCAAGTGTTTATTATGTCTCAGGTACAGTTCTAGTTCCCAGGACATACAAAGATG
NC_000001.11:33 ------------------------------------------------------------
NC_000023.11:15 ------------------------------------------------------------
NC_000004.12:c1 ------------------------------------------------------------

NC_000013.11:c3 AGAATGATTCCGTCCTTGCCCTTGGATTCAAATGGGCAAGACAGACAAAATTATAATAAC
NC_000001.11:33 ------------------------------------------------------------
NC_000023.11:15 ------------------------------------------------------------
NC_000004.12:c1 ------------------------------------------------------------

NC_000013.11:c3 TTGTGGTAAGTCCTGTGCTATCTGTGTGAACAATGTGCTGTGGAAACAGGAGCAAGCAGC
NC_000001.11:33 ------------------------------------------------------------
NC_000023.11:15 ------------------------------------------------------------
NC_000004.12:c1 ------------------------------------------------------------

NC_000013.11:c3 TGATTGCCCCAGTGGATATCTAAGAAAGGCTTCATTAAGGAGTGACTTATGAAATAGGTC
NC_000001.11:33 ---------------------------------------------------------GTT
NC_000023.11:15 ----------------------------------------------------AGCGAGAT
NC_000004.12:c1 ------------------------------------------------------------

NC_000013.11:c3 ATATGGAATGAATGAGAATATCCCAGGCATAAGAGAGAAGGGCATTCCTGACAGAATGAA
NC_000001.11:33 ATGAGGACTGAATGGAA-------------------------------------------
NC_000023.11:15 CCGAGGACTAAAT-----------------------------------------------
NC_000004.12:c1 ------------------------------------------------------------

NC_000013.11:c3 CTGCATATGCAAAGTCAGGGAGGTTTGAAAGCGTTGAACTTGTTCCAGCAACAGTAAGAA
NC_000001.11:33 ------------------------------------------------------------
NC_000023.11:15 ------------------------------------------------------------
NC_000004.12:c1 ------------------------------------------------------------

NC_000013.11:c3 AGAAGTTTAGTGTAGCAAGAGTATAGGTATATATAGAGAAGTGAGGTGGAGCCCATTGAA
NC_000001.11:33 ------------------------------------------------------------
NC_000023.11:15 ------------------------------------------------------------
NC_000004.12:c1 ------------------------------------------------------------

NC_000013.11:c3 GACCTATGCACCTTGCTAAGGAGGTTAGACTTTCTTCTTATATGGTGAGAAGCCCAGAGA
NC_000001.11:33 ------------------------------------------------------------
NC_000023.11:15 ------------------------------------------------------------
NC_000004.12:c1 ------------------------------------------------------------

NC_000013.11:c3 GGTTCCGAATCTAGGATATGTAAGATAGGATATATGTGCTTTAGGATGACCATTCTGTCG
NC_000001.11:33 ------------------------------------------------------------
NC_000023.11:15 ------------------------------------------------------------
NC_000004.12:c1 ------------------------------------------------------------

NC_000013.11:c3 GCTGTTTAAAGGATGGTGTGAAGAAAAGAGAGATTAGAGTTAGGAAATCCTTTGCATTGC
```

```
NC_000001.11:33 ------------------------------------------------------------
NC_000023.11:15 ------------------------------------------------------------
NC_000004.12:c1 ------------------------------------------------------------

NC_000013.11:c3 TTCAAATGGGAAATAAGGACTTCAACTAAGACAGGAGTGATGGGTATGGAAAAGATGGTC
NC_000001.11:33 ------------------------------------------------------------
NC_000023.11:15 ------------------------------------------------------------
NC_000004.12:c1 ------------------------------------------------------------

NC_000013.11:c3 TAGATTCCAGAGCTCAATTCCATTCAGTTTGTGGAACATTTACTGACATTTACCATATAG
NC_000001.11:33 ------------------------------------------------------------
NC_000023.11:15 ------------------------------------------------------------
NC_000004.12:c1 ------------------------------------------------------------

NC_000013.11:c3 CAGGCTGCTAAACACTGTGATACAAAAAAAAAAAAAAAAAAAAAAAAAAAGAACAAGAAC
NC_000001.11:33 ------------------------------------------------------------
NC_000023.11:15 ------------------------------------------------------------
NC_000004.12:c1 ------------------------------------------------------------

NC_000013.11:c3 TGGTCTCTGATCTTGAGTTTTCTGTCTAGTGGGGAACAAAGACACACCAACAGGTCACTT
NC_000001.11:33 ------------------------------------------------------------
NC_000023.11:15 ------------------------------------------------------------
NC_000004.12:c1 ------------------------------------------------------------

NC_000013.11:c3 CAGTATGATAATGGTTAAGTGTCATAATAAATAAAGGCTTGCAAAAGGTATGAAAGGAGC
NC_000001.11:33 ------------------------------------------------------------
NC_000023.11:15 ------------------------------------------------------------
NC_000004.12:c1 ------------------------------------------------------------

NC_000013.11:c3 AAAGATGGGTTCATAACCTACACTAAGGTTCATAAAGCCTTCCTGAGAGGGAAACTTCCA
NC_000001.11:33 -----------------------TGAGGTACATAAAGTGCT--TAGAAGAGAGACT----
NC_000023.11:15 ------------------------------CCCGAGGTCTTGGCGAGCAAGCAGCT----
NC_000004.12:c1 ------------------------------------------------------------

NC_000013.11:c3 AGCAGAATCAAGGAGAATGAATGGGAGCTGGCCAAGGGAAGCAAAGGGCAAGGACGTTGC
NC_000001.11:33 ------------------------------------------------------------
NC_000023.11:15 ------------------------------------------------------------
NC_000004.12:c1 ------------------------------------------------------------

NC_000013.11:c3 AGGCAGAGGGCTGGGGAGAGTCTGCCTGAGGGTCAGGGAACCACCAGGAACTGCACGGAG
NC_000001.11:33 ------------------------------------------------------------
NC_000023.11:15 ------------------------------------------------------------
NC_000004.12:c1 ------------------------------------------------------------

NC_000013.11:c3 TCTCTTGCTGCTTACCTCCATGTGGATGAGGAGTGACACGAAGGAGCCTGAGGTTGGAGT
NC_000001.11:33 ------------------------------------------------------------
NC_000023.11:15 ------------------------------------------------------------
NC_000004.12:c1 ------------------------------------------------------------

NC_000013.11:c3 GGAAAGTTCACCTGTGTTCCTGTAGTGGAACTTGGGGATTCATATGGAGCCTCTAAGAAA
NC_000001.11:33 ------------------------------------GGCTCATATTAAGCATTATATAAA
NC_000023.11:15 ------------------------------------GCCCTCCGCCAGGCATCGAGGAGC
NC_000004.12:c1 ------------------------------------------------------------
```

```
NC_000013.11:c3 TGCTAAGGAAGCTTGGACTTTATCCTGTTGATGGTGGGGAGCCCACTGAAGTGTTTTAAA
NC_000001.11:33 TA----------------------------------------------------------
NC_000023.11:15 GA----------------------------------------------------------
NC_000004.12:c1 ------------------------------------------------------------

NC_000013.11:c3 ACATGAGGATGATGTGGGCAGCCTTGTGGCTTAGGTGGTTTATTCTGATAACGGATGAGA
NC_000001.11:33 ------------------------------------------------------------
NC_000023.11:15 ------------------------------------------------------------
NC_000004.12:c1 ------------------------------------------------------------

NC_000013.11:c3 TAATAGTAGCTCACTTTTCGTTGAATGCTTATAATGTGCCAAGCTGTGTTGTAAGCATTA
NC_000001.11:33 ------------------------------------------------------------
NC_000023.11:15 --------------------------------------------------GCTGCCGCCG
NC_000004.12:c1 ------------------------------------------------------------

NC_000013.11:c3 TGCATGTATCGCCTTGTTGAAACAACCCTGCTATTATTAGCCTCATTTCACAGGGAGGTT
NC_000001.11:33 ------------------------------------------------------------
NC_000023.11:15 CGTTTCTGTCGCGGCGCCGCGGC-------------------------------------
NC_000004.12:c1 ------------------------------------------------------------

NC_000013.11:c3 ACTGACACACAGAGAGATTAAGTAACTTGCTCAGATTTACACAGGCAGTGGCAGAATGAG
NC_000001.11:33 ------------------------------------------------------------
NC_000023.11:15 ------------------------------------------------------------
NC_000004.12:c1 ------------------------------------------------------------

NC_000013.11:c3 GACTTAATCCGCAGTGATCTGACTCTAACCCACAGTGATACTGCTAGGTAGGTAGATGTG
NC_000001.11:33 -------------------------------------------TTAAGTATGTATGTATG
NC_000023.11:15 -------------------------------------------CGAACCGGGTAGAGGTG
NC_000004.12:c1 ------------------------------------------------------------

NC_000013.11:c3 AGAGAGTGGCTGGACTCTAGGGAAGAGAGGATGATGAGCTACTCTGCGGTAACAGAGAGA
NC_000001.11:33 T-----------------------------------------------------------
NC_000023.11:15 G-----------------------------------------------------------
NC_000004.12:c1 ------------------------------------------------------------

NC_000013.11:c3 AGGTCACTGATGAGTCAGCTTGGGCTGCCACAGCAAAATACCACAGACTGGGCGGTGCAA
NC_000001.11:33 ------------------------------------------------------------
NC_000023.11:15 ------------------------------------------------------------
NC_000004.12:c1 ------------------------------------------------------------

NC_000013.11:c3 GCAACAGAAATTTTCTCACAGCTCTGGAGGCTGGAAGTCCTCCAGGTGCTATCAGGATTG
NC_000001.11:33 ------------------------------------------------------------
NC_000023.11:15 ------------------------------------------------------------
NC_000004.12:c1 ------------------------------------------------------------

NC_000013.11:c3 GGTTTTGGTGAGGACGCTCTTCTGGGCTATATATGGCCTTTTTTTTTTTTTTTTTTAAAG
NC_000001.11:33 ------------------------------------------------------------
NC_000023.11:15 ------------------------------------------------------------
NC_000004.12:c1 ------------------------------------------------------------

NC_000013.11:c3 AGACTGGGTCTCATGCTGTCACCCAGGCTGGAGTGCAGAGGTGTGATCATAGCTTGCTGC
NC_000001.11:33 ------------------------------------------------------------
NC_000023.11:15 ------------------------------------------------------------
```

```
NC_000004.12:c1 ------------------------------------------------------------

NC_000013.11:c3 AGCCTCAAATTCCTGGGCTCAAGCCATCCTCCTGCCTCAGCCTCCTGAGGAGCTGGGACC
NC_000001.11:33 ------------------------------------------------------------
NC_000023.11:15 -----------------------------TCTACCATCGGCCCTTCGTGG---TGGGCCC
NC_000004.12:c1 ------------------------------------------------------------

NC_000013.11:c3 ACAGGTGTGTACCACTGTGTCTGACTATTTTATTTTCTTGTAGAGATGGAGTCTCACTCT
NC_000001.11:33 ------------------------------------------------------------
NC_000023.11:15 AGGGGTGT----------------------------------------------------
NC_000004.12:c1 ------------------------------------------------------------

NC_000013.11:c3 GTTGCCCAGGCTGGTCTCAGACTCCTCTCCTTGAGTGATCTTCCCACCTCAGCCTCCCAA
NC_000001.11:33 ------------------------------------------------------------
NC_000023.11:15 ------------------------------------------------------------
NC_000004.12:c1 ------------------------------------------------------------

NC_000013.11:c3 AATACTGGGATTACAGGTGTGAGCCCCTGTGCCCAGCTCTAGTCTCTTCTTATAAGGACA
NC_000001.11:33 ------------------------------------------------------------
NC_000023.11:15 ------------------------------------------------------------
NC_000004.12:c1 ------------------------------------------------------------

NC_000013.11:c3 CTGATCCTATCAGATCAGGGCTCCACCTTTGACCTCACTGAACCTTAATTACTCCTTAGA
NC_000001.11:33 ------------------------------------------------------------
NC_000023.11:15 ------------------------------------------------------------
NC_000004.12:c1 ------------------------------------------------------------

NC_000013.11:c3 GGTCACGTCTCTAAATACAGTCACATCAGGGATTAGGACATATGAATTTGGGGGAACACA
NC_000001.11:33 ------------------------------------------------------------
NC_000023.11:15 ------------------------------------------------------------
NC_000004.12:c1 ------------------------------------------------------------

NC_000013.11:c3 AATATTCAGTCCATGACAGCGACAATTCTGGAGAGTATTTGAAGATAAAATCAGCACAAG
NC_000001.11:33 ------------------------------------------------------------
NC_000023.11:15 ------------------------------------------------------------
NC_000004.12:c1 ------------------------------------------------------------

NC_000013.11:c3 TTGGTAATCGATTTGATGTGGGGAGTGAGGTGGAAGGAGGAGTTTAGGATGACTCTCAGG
NC_000001.11:33 ------------------------------------------------------------
NC_000023.11:15 ------------------------------------------------------------
NC_000004.12:c1 ------------------------------------------------------------

NC_000013.11:c3 TTCTCACAAAGATGCAGAGGAGGGGCAAGCCTGTGGGGTGAATGAACAGCAGTTCGGTTG
NC_000001.11:33 ------------------------------------------------------------
NC_000023.11:15 ------------------------------------------------------------
NC_000004.12:c1 ------------------------------------------------------------

NC_000013.11:c3 AGGTTACTGTAGTTCAGCACTTGGAAAGCTTCCAGGAGATAGTTGCGATGCTGATGTGGA
NC_000001.11:33 ------------------------------------------------------------
NC_000023.11:15 ------------------------------------------------------------
NC_000004.12:c1 ------------------------------------------------------------

NC_000013.11:c3 GAGAGGCAGCGACGTGTGTGGATGGCAGTGGAAGCCACAGGCTGGGTCACATCACCTAGG
```

```
NC_000001.11:33 ------------------------------------------------------------
NC_000023.11:15 ------------------------------------------------------------
NC_000004.12:c1 ------------------------------------------------------------

NC_000013.11:c3 GAAAACATGTAGAGTGAGAAGACATGTAGGACTGCCAAGATGGAAAAATATTTACGAGGC
NC_000001.11:33 ------------------------------------------------------------
NC_000023.11:15 ------------------------------------------------------------
NC_000004.12:c1 ------------------------------------------------------------

NC_000013.11:c3 AAGCAGAAGAAAGCAAAGGGAGACTTAAGAAATAACATACCTTTATCCCTGAACCCAAAG
NC_000001.11:33 ------------------------------------------------------------
NC_000023.11:15 ------------------------------------------------------------
NC_000004.12:c1 ------------------------------------------------------------

NC_000013.11:c3 GAAGAGAGAATTCCAGGCAACAGGGAGTGTTTGACACATTCTGCGGCCTCTTTGTATCAA
NC_000001.11:33 ------------------------------------------------------------
NC_000023.11:15 ------------------------------------------------------------
NC_000004.12:c1 ------------------------------------------------------------

NC_000013.11:c3 ATTTGTATTCTTAGGTGAGAAAAGGGAACAGGCTTAGAATCATAGCTTTTTATTGCCCAG
NC_000001.11:33 ------------------------------------------------------------
NC_000023.11:15 ------------------------------------------------------------
NC_000004.12:c1 ------------------------------------------------------------

NC_000013.11:c3 TAGGACCAAAAGAGATGATTTTATAATTCTTTATTCTACAAACAAGGAAGCTGAAGCCCT
NC_000001.11:33 ------------------------------------------------------------
NC_000023.11:15 ---------------------------------TTCCCCGGGCAGGGGAGCT--------
NC_000004.12:c1 ------------------------------------------------------------

NC_000013.11:c3 GCAAAGCCTTGGGATTTGTCTCTTTCATGGTACAGGCAGGAGTAGAATTTAGGTCTCCCA
NC_000001.11:33 ------------------------------------------------------------
NC_000023.11:15 ------------------------------------------------------------
NC_000004.12:c1 ------------------------------------------------------------

NC_000013.11:c3 AGCCATTTGACTTCAATCCAGCGCTTTTTACAATACATCAAATAAAATCCACCATTGTGG
NC_000001.11:33 ------------------------------------------------------------
NC_000023.11:15 ------------------------------------------------------------
NC_000004.12:c1 ------------------------------------------------------------

NC_000013.11:c3 GGTTTGAAAATTGGGTCTATGTTATAAAAAAAATTACACTAAATGGAGAAAGAAGCATTC
NC_000001.11:33 ------------------------------------------------------------
NC_000023.11:15 ------------------------------------------------------------
NC_000004.12:c1 ------------------------------------------------------------

NC_000013.11:c3 AAATTTTATTTGTATATGCATTTGGGGAGAGGAAGACATAGTATAGTATAGAAAATGTCA
NC_000001.11:33 ------------------------------------------------------------
NC_000023.11:15 ------------------------------------------------------------
NC_000004.12:c1 ------------------------------------------------------------

NC_000013.11:c3 AAGACTTTGTCTCAGAGGAGAGAATACCAATGTTGGCCTTGATGGTGATGATGAGATTGA
NC_000001.11:33 ------------------------------------------------------------
NC_000023.11:15 ------------------------------------------------------------
NC_000004.12:c1 ------------------------------------------------------------
```

```
NC_000013.11:c3 CTAGTGGAAGAGGGCCTAGAGACAGGGATTCTGTCCTTACCATATTCACAGTGGAAATGG
NC_000001.11:33 ------------------------------------------------------------
NC_000023.11:15 ------------------------------------------------------------
NC_000004.12:c1 ------------------------------------------------------------

NC_000013.11:c3 CATGTTACCAGCGGCTCCTGTTATGTCCTGAGCACACTTAAAACGAATTTTTTTTAGAGA
NC_000001.11:33 ------------------------------------------------------------
NC_000023.11:15 ------------------------------------------------------------
NC_000004.12:c1 ------------------------------------------------------------

NC_000013.11:c3 CAGGGTCTCACTCTGTTGCCCAGGCTGAGTGCAGTGGCTTACTGCAGCCTCAAACTCGTG
NC_000001.11:33 ------------------------------------------------------------
NC_000023.11:15 ------------------------------------------------------------
NC_000004.12:c1 ------------------------------------------------------------

NC_000013.11:c3 AGCTCAAGGGATCTTCCTGCCTCAGCCCCTGGAATAGCTGGACTACAGGCAGGTGTCACC
NC_000001.11:33 ------------------------------------------------------------
NC_000023.11:15 ----------------------------------------GACTGCAGGCCAGGGGCGCC
NC_000004.12:c1 ------------------------------------------------------------

NC_000013.11:c3 ATGCACCTAAGCACACATTTTTAAATGTGACTCTGTCTTATGTTTTTTTCCTATCATTGA
NC_000001.11:33 ------------------------------------------------------------
NC_000023.11:15 A-----------------------------------------------------------
NC_000004.12:c1 ------------------------------------------------------------

NC_000013.11:c3 CTAACCCCACATTAGATACATAGCAGTACTGTATTATTAGAAAATTAATTTTAAGTCAGT
NC_000001.11:33 ------------------------------------------------------------
NC_000023.11:15 ------------------------------------------------------------
NC_000004.12:c1 ------------------------------------------------------------

NC_000013.11:c3 ACCTTATTACAGGCTTGAAACTGACAAACTTCATTACTTGGGGAGAGGAGTGGGTTCTTT
NC_000001.11:33 ------------------------------------------------------------
NC_000023.11:15 ------------------------------------------------------------
NC_000004.12:c1 ------------------------------------------------------------

NC_000013.11:c3 TACAGGATTTCAAAATTGCCTTCAGCCACGGAGACTCCCTTAAGTGAAAATTTCTGTCAG
NC_000001.11:33 ------------------------------------------------------------
NC_000023.11:15 ------------------------------------------------------------
NC_000004.12:c1 ------------------------------------------------------------

NC_000013.11:c3 TAACAGACAGTTTATCTTAGAATTCAGTTAATGGAGTATGTATTGAGTATCTACTATAAA
NC_000001.11:33 ---------------------------------------------------------AAA
NC_000023.11:15 ------------------------------------------------------------
NC_000004.12:c1 ------------------------------------------------------------

NC_000013.11:c3 TGAACCACGATGTTGAGCACTTCAGGAGGTATTAATGAATTGAACAGAGTCCCTATGTAA
NC_000001.11:33 CGAGTCATGATGT-----------------------------------------------
NC_000023.11:15 -AGGCCGCGGTGC-----------------------------------------------
NC_000004.12:c1 ------------------------------------------------------------

NC_000013.11:c3 GACATGTATGGCCTGTTGCTAGAGTGAAGGAACAGCGTCATATGAACCTTTAGACTCACA
NC_000001.11:33 ------------------------------------------------------------
NC_000023.11:15 -----------------GGTGGGAGCAGAGGGCAGCG-----------------------
```

```
NC_000004.12:c1 ------------------------------------------------------------

NC_000013.11:c3 AATATTTTAATGGGGTCAGCGTAGTCAATAGGGCCAGTACATCCTCATCAGTACTTACTG
NC_000001.11:33 ------------------------------------------------------------
NC_000023.11:15 -------------------------CAGCGGGGCTGG-----------------------
NC_000004.12:c1 ------------------------------------------------------------

NC_000013.11:c3 AAAATATGAGCTGACTTCGAGCTGTATTTCTCACTTTCAAATTTTAAAAAACACAAACTG
NC_000001.11:33 ------------------------------------------------------------
NC_000023.11:15 ------------------------------------------------------------
NC_000004.12:c1 ------------------------------------------------------------

NC_000013.11:c3 AAGCAAGTTTAACCACCATCATGGTTTGTATGTTAATTGCTTTCTTGTTTTTACTAACTA
NC_000001.11:33 ------------------------------------------------------------
NC_000023.11:15 ------------------------------------------------------------
NC_000004.12:c1 ------------------------------------------------------------

NC_000013.11:c3 CATAAGCATGAACTTAATATAAAAGGCTACTATTTTTTCATTTTATAACTTTATGGCATG
NC_000001.11:33 ------------------------------------------------------------
NC_000023.11:15 ------------------------------------------------------------
NC_000004.12:c1 ------------------------------------------------------------

NC_000013.11:c3 AAAATGTATACGCTGACACTTAAAATAATAAACATATGCACACCATGCAGATACTTTTAC
NC_000001.11:33 AAAATGT-----------------------------------------------------
NC_000023.11:15 ------------------------------------------------------------
NC_000004.12:c1 ------------------------------------------------------------

NC_000013.11:c3 TCGGAGTTTATATTTTTGGTAGATTTTAATATATCAATTAAGATAAAATTATACAGAATA
NC_000001.11:33 ------------------------------------------------------------
NC_000023.11:15 ------------------------------------------------------------
NC_000004.12:c1 ------------------------------------------------------------

NC_000013.11:c3 ACTGATTCCCCAACAGACACATCTTTAGTACGAGGCCATTAATAGAATTTTTATGTAGTA
NC_000001.11:33 ------------------------------------------------------------
NC_000023.11:15 ------------------------------------------------------------
NC_000004.12:c1 ------------------------------------------------------------

NC_000013.11:c3 TATGCATATTACAAAGAGTACATTGTATGGAAAATAAACCAAGCTAGATTCCTGGATTCC
NC_000001.11:33 ------------------------------------------------------------
NC_000023.11:15 ------------------------------------------------------------
NC_000004.12:c1 ------------------------------------------------------------

NC_000013.11:c3 TAGCATGGTTTATTTAATGCAAGTATTAAATTAAAAAGTCTGGTTAGCATGCAGTTAGCA
NC_000001.11:33 ------------------------------------------------------------
NC_000023.11:15 ------------------------------------------------------------
NC_000004.12:c1 ------------------------------------------------------------

NC_000013.11:c3 TCATAAAAAGAATTTCCACATATGACTATAGTAGTATACATTTCTGCAGTAAGGACAGCC
NC_000001.11:33 ------------------------------------------------------------
NC_000023.11:15 ------------------------------------------------------------
NC_000004.12:c1 ------------------------------------------------------------

NC_000013.11:c3 CTCTACAAAATTATGAAGTATGTAGGTATCTTGGCACAACCCATAACATTTTTTATTCCA
```

```
NC_000001.11:33 -----------------------------------------------ATTTCTTATTGCA
NC_000023.11:15 -----------------------------------------------ACTTCATCTTCCC
NC_000004.12:c1 ------------------------------------------------------------


NC_000013.11:c3 AATATGAGAATTCTTACCACTGAAGGCATTTCATGAAAAGTTGGATCCATAATTCTCCAC
NC_000001.11:33 GA----------------------------------------------------------
NC_000023.11:15 AC----------------------------------------------------------
NC_000004.12:c1 ------------------------------------------------------------


NC_000013.11:c3 TAGTCAAGACTTTCAGTAGAATGAGCACCTTATTACATTCATCTTTATTTTCCTATTTCC
NC_000001.11:33 ------------------------------------------------------------
NC_000023.11:15 ------------------------------------------------------------
NC_000004.12:c1 ------------------------------------------------------------


NC_000013.11:c3 TAGCAGGGTCATGGAAATTAGCATATTCTCAATGAAAGTTGATAAATGAATGCATTCTGC
NC_000001.11:33 ------------------------------------------------------------
NC_000023.11:15 ------------------------------------------------------------
NC_000004.12:c1 ------------------------------------------------------------


NC_000013.11:c3 TAAAAGTAAACATTGAGAAGAAATTTTTACCGTCGATTATGTTGCCATAGTGCTATTATC
NC_000001.11:33 ------------------------------------------------------------
NC_000023.11:15 ------------------------------------------------------------
NC_000004.12:c1 ------------------------------------------------------------


NC_000013.11:c3 ACAAGAGAATTCATTTAGCAACCATTTATTGAACATCATCTATGGTAGACAGTGTGTTAG
NC_000001.11:33 ------------------------------------------------------------
NC_000023.11:15 ------------------------------------------------------------
NC_000004.12:c1 ------------------------------------------------------------


NC_000013.11:c3 CACTAGGAATACAGAACTTAATAAGATGAGCCTTCCAGCAGTGGGATTGGGCAACAGTGG
NC_000001.11:33 ------------------------------------------------------------
NC_000023.11:15 ------------------------------------------------------------
NC_000004.12:c1 ------------------------------------------------------------


NC_000013.11:c3 ATGCAAGAGGCAAAAGAACTTCAAACAGTTGTGATAAAGAGGAAAGATAGTAATGCTATC
NC_000001.11:33 ------------------------------------------------------------
NC_000023.11:15 ------------------------------------------------------------
NC_000004.12:c1 ------------------------------------------------------------


NC_000013.11:c3 AGAGAAATATCTACAAACAAAATAGTGTTATGCTATTAGAAAAATACAGAGTGCTGTGGG
NC_000001.11:33 ----------CTGCAATCAAGATG------------------------------------
NC_000023.11:15 --------GCCAGAGGCCGAGGCC------------------------------------
NC_000004.12:c1 ------------------------------------------------------------


NC_000013.11:c3 AGCCCAGAAGAGAAATTAATTCCAGTTGGACGTAGGAGTGTTTGTCAGGATTTCATATGG
NC_000001.11:33 ------------------------------------------------------------
NC_000023.11:15 ------------------------------------------------------------
NC_000004.12:c1 ------------------------------------------------------------


NC_000013.11:c3 GAAGTGGCTTTTGAGTTAAACTTTGAATATCTCACTGGCATCTTGGGCATCTCAAATCCA
NC_000001.11:33 ------------------------------------------------------------
NC_000023.11:15 ------------------------------------------------------------
NC_000004.12:c1 ------------------------------------------------------------
```

```
NC_000013.11:c3 CTGGGTCTATACTTGTGTTCATCATTTATTCATCATTTTCCCCTTCCACCCATTCATCCC
NC_000001.11:33 ------------------------------------------------------------
NC_000023.11:15 ------------------------------------------------------------
NC_000004.12:c1 ------------------------------------------------------------

NC_000013.11:c3 CAGCTTGCATGTCTGATAGTCTTGAACATGATTATTGATATTGCCGTTCACTGCTGGGTC
NC_000001.11:33 ------------------------------------------------------------
NC_000023.11:15 ------------------------------------------------------------
NC_000004.12:c1 ------------------------------------------------------------

NC_000013.11:c3 CTTCCTTTTAGAAAATGCTAGATTAGGCCGGGTGCGGTGGCTCACGCCTGTAATCCCAGC
NC_000001.11:33 -------TTTGAAAATACTGAA------------------CTCACATCTGTAG-------
NC_000023.11:15 -------TCCGGGAATCGCAGG------------------GCCACCTCTTCAA-------
NC_000004.12:c1 ------------------------------------------------------------

NC_000013.11:c3 ACTTTGGGAGGCTGAGTTGGGTAGATCACTTGAGACCAGGAGTTCCAGATCAGCCACGGC
NC_000001.11:33 ------------------------------------------------------------
NC_000023.11:15 ------------------------------------------------------------
NC_000004.12:c1 ------------------------------------------------------------

NC_000013.11:c3 AACCTGGTGAAACCTCGTCTCTGCAAAAAATACCAAAAAAATTAGCTGGACGTGTTGGCG
NC_000001.11:33 ------------------------------------------------------------
NC_000023.11:15 ------------------------------------------------------------
NC_000004.12:c1 ------------------------------------------------------------

NC_000013.11:c3 CATGCCTGTAGTCCCTGCTACTTGGGAGGCCGAGGTGGGAGGATCACCTAAGCCCATAAA
NC_000001.11:33 ------------------------------------------------------------
NC_000023.11:15 ------------------------------------------------------------
NC_000004.12:c1 ------------------------------------------------------------

NC_000013.11:c3 GTTGAGGCTGCAGTGAGCCATGATCGTGCCATTGCACTCCCACCTGGGTGGCACAGTGAG
NC_000001.11:33 ------------------------------------------------------------
NC_000023.11:15 ------------------------------------------------------------
NC_000004.12:c1 ------------------------------------------------------------

NC_000013.11:c3 ACCCTTTCTCCAAAAAAAAAAAAAAAAAGAAAATGCTAGATTCATTTGACTCCTTTTCTT
NC_000001.11:33 ------------------------------------------------------------
NC_000023.11:15 ------------------------------------------------------------
NC_000004.12:c1 ------------------------------------------------------------

NC_000013.11:c3 TCCTCTCCAGTATTTATTTGGTCACTTTAGTTATGTGAAGTTTTGAATAATTTCCAATTT
NC_000001.11:33 ------------------------------------------------------------
NC_000023.11:15 ------------------------------------------------------------
NC_000004.12:c1 ------------------------------------------------------------

NC_000013.11:c3 TTTACAAATTATATGGTTTGTATGTAATGAAAAGCTTATCAAAAATGTTTCAGTTTATCA
NC_000001.11:33 ------------------------------------------------------------
NC_000023.11:15 ------------------------------------------------------------
NC_000004.12:c1 ------------------------------------------------------------

NC_000013.11:c3 GTCTACTAATCAAATATTTTTAAACTTTTAAAATTATACACAGAAATGTGGTCCTATTTT
NC_000001.11:33 ------------------------------------------------------------
NC_000023.11:15 ------------------------------------------------------------
```

```
NC_000004.12:c1 ------------------------------------------------------------

NC_000013.11:c3 GAAACAATTCTCTCATTTTTTTCACATTAAAAAGTTCAATTCTGATTTCTAAAAATCAGA
NC_000001.11:33 ------------------------------------------------------------
NC_000023.11:15 ------------------------------------------------------------
NC_000004.12:c1 ------------------------------------------------------------

NC_000013.11:c3 AAATCATAAAGAATTTATTATGGAGAAGCTATCCTGGTGTACAATTATGACAGTATTTTT
NC_000001.11:33 ------------------------------------------------------------
NC_000023.11:15 ------------------------------------------------------------
NC_000004.12:c1 ------------------------------------------------------------

NC_000013.11:c3 ATTTTAAATGATACTGTCCTTCTAAGAATGTGTTTCTGTCAATTTGTCCTTATCTCTACA
NC_000001.11:33 ------------------------------------------------------------
NC_000023.11:15 ------------------------------------------------------------
NC_000004.12:c1 ------------------------------------------------------------

NC_000013.11:c3 GAACCCTGAAATTGTAGCACTACTGTAGTGTTTCTAGAGAGTAATACGTTTCTGCTGCCA
NC_000001.11:33 ------------------------------------------------------------
NC_000023.11:15 ------------------------------------------------------------
NC_000004.12:c1 ------------------------------------------------------------

NC_000013.11:c3 TTTGGAGGAGCCTGCATCACAGAGTAATGAGTTGAGTCTGACTTACAAAGATGCCTCCTT
NC_000001.11:33 ------------------------------------------------------------
NC_000023.11:15 ------------------------------------------------------------
NC_000004.12:c1 ------------------------------------------------------------

NC_000013.11:c3 TGCAATTCAGACCCCCCAGAAGAATTTGCAGTCAAATTCCTATAATTAATGAGTTGTGTC
NC_000001.11:33 ------------------------------------------------------------
NC_000023.11:15 ------------------------------------------------------------
NC_000004.12:c1 ------------------------------------------------------------

NC_000013.11:c3 TTCTAGATTCCTTTAAGTACTACATCTAATGGAAGTTACAATTATAACTTAATTATTTTA
NC_000001.11:33 ------------------------------------------------------------
NC_000023.11:15 ------------------------------------------------------------
NC_000004.12:c1 ------------------------------------------------------------

NC_000013.11:c3 AATAGTAGAGATTCTGATGGTGTGCTTACACCACTGAAACAAAAGATATTCTGAAAACTG
NC_000001.11:33 ------------------------------------------------------------
NC_000023.11:15 ------------------------------------------------------------
NC_000004.12:c1 ------------------------------------------------------------

NC_000013.11:c3 AAAGTGAAACTGTCAGCCCTTGGCATCTGTAGTTAACAAACTACTCAAGAGTTTTGTTGC
NC_000001.11:33 ------------------------------------------------------------
NC_000023.11:15 --------------AGCCCTTGGCGATCCCAGT---------------------------
NC_000004.12:c1 ------------------------------------------------------------

NC_000013.11:c3 TAGATATCTAGATAATTGTGAGAATAAGCCAAGAGTGTGGGAGGCTTGCTTTGCTCTTCA
NC_000001.11:33 ------------------------------------------------------------
NC_000023.11:15 ------------------------------------------------------------
NC_000004.12:c1 ------------------------------------------------------------

NC_000013.11:c3 GATGAAATGGCTTAAGTTTTGCCAAAATCCCTGAAGGGTAATCACGGTCTCTTTTAATCC
```

```
NC_000001.11:33 ------------------------------------------------------------
NC_000023.11:15 ------------------------------------------------------------
NC_000004.12:c1 ------------------------------------------------------------


NC_000013.11:c3 CAAGTTTCAGATAATACCCACAAATAACTTATTCTTTAGAGTGGTCTTTGGAATTAGAGA
NC_000001.11:33 ------------------------------------------------------------
NC_000023.11:15 ------------------------------------------------------------
NC_000004.12:c1 ------------------------------------------------------------


NC_000013.11:c3 AAGCAGAAATGTATTAAAATGTATTTCATTCACTTTGTTCCTGATCCTTACACCAGGGTC
NC_000001.11:33 ------------------------------------------------------------
NC_000023.11:15 ---------------------------------TCTGTTCCC------------------
NC_000004.12:c1 ------------------------------------------------------------


NC_000013.11:c3 AACGGAGTCACTAAGAATTAGCAAAGTTCATTGGTAAGAGATTTACCCTTCTGATATCTT
NC_000001.11:33 ---------ACGAGGAGTTGGCAGATTT--------------------------------
NC_000023.11:15 ---------CGAGGGGGCTGGCGAGGTG--------------------------------
NC_000004.12:c1 ---------TCTGGGAGTTTTCAATGTC--------------------------------
                            ..*...*  *..  *

NC_000013.11:c3 GCTCTGGTGAAATACTAGAAAAATGTCTTAAGAAGTAAAAGGCAGATGTGAGAGTTAAAG
NC_000001.11:33 ------------------------------------------------------------
NC_000023.11:15 ------------------------------------------------------------
NC_000004.12:c1 ------------------------------------------------------------


NC_000013.11:c3 GAAATTTCAGTTAATTAGCAGAGTTGCATTAAAATAATCTTACCATGGATCATGAAGTCT
NC_000001.11:33 ------------------------------------------------------------
NC_000023.11:15 ------------------------------------------------------------
NC_000004.12:c1 ------------------------------------------------------------


NC_000013.11:c3 TGAATTCTAAATTTGCCTTTGCTGGTATAATCCTAATTAGATATTTTACTTAATTTTTGA
NC_000001.11:33 ------------------------------------------------------------
NC_000023.11:15 ------------------------------------------------------------
NC_000004.12:c1 ------------------------------------------------------------


NC_000013.11:c3 AATTTCATAATAAATAACCCAGCTTCTATCAACAAAATGAAGACTTTGCATTTAGAATTG
NC_000001.11:33 ------------------------------------------------------------
NC_000023.11:15 ------------------------------------------------------------
NC_000004.12:c1 ------------------------------------------------------------


NC_000013.11:c3 CCTTCAAAATGCAATCAATTCCAATGTCATTACAAAATGTAACTGAACGGTCTCTGAGAG
NC_000001.11:33 ------------------------------------------------------------
NC_000023.11:15 ------------------------------------------------------------
NC_000004.12:c1 ------------------------------------------------------------


NC_000013.11:c3 CTAAGTTGAACACTTTATCTCTGAGATGTCGTTGCATTCTATTGCAGTTATTTTCTGACA
NC_000001.11:33 ------------------------------------------------------------
NC_000023.11:15 ------------------------------------------------------------
NC_000004.12:c1 ------------------------------------------------------------


NC_000013.11:c3 TGTGTGGGGGAAGAAAGGTATTTCCTACTTTGAGCATTTATCAACACACTTACCCTGATA
NC_000001.11:33 ------------------------------------------------------------
NC_000023.11:15 ------------------------------------------------------------
NC_000004.12:c1 ------------------------------------------------------------
```

```
NC_000013.11:c3 CCCATGAACAGTCCCACCTCTGCCTGGCATTGTTTTATACTTGCTGTTGTCACATTCATT
NC_000001.11:33 ------------------------------------------------------------
NC_000023.11:15 ------------------------------------------------------------
NC_000004.12:c1 ------------------------------------------------------------

NC_000013.11:c3 CTTCAAGTTTTAACTCAAAGTCAGTTCTTTGAAGTCATGCTTACTCTCCCATCATGAACT
NC_000001.11:33 ------------------------------------------------------------
NC_000023.11:15 ------------------------------------------------------------
NC_000004.12:c1 ------------------------------------------------------------

NC_000013.11:c3 TGCAAAATTAGGCCTGAAGCTATTTTATTTGCTCCCAAGTAATATCTGCTATTCAGGTAT
NC_000001.11:33 ------------------------------------------------------------
NC_000023.11:15 ------------------------------------------------------------
NC_000004.12:c1 ------------------------------------------------------------

NC_000013.11:c3 ATATCAACACAGAAATTGCCTCAGGTGTAGTCTCACAATGTAGACAACTGAGTTTATCAT
NC_000001.11:33 ------------------------------------------------------------
NC_000023.11:15 ------------------------------------------------------------
NC_000004.12:c1 ------------------------------------------------------------

NC_000013.11:c3 ACACCAAATTTACTAAAAGCAAATATAAACATACTAATCCATTAAATTATTTTAATATAT
NC_000001.11:33 ------------------------------------------------------------
NC_000023.11:15 ------------------------------------------------------------
NC_000004.12:c1 ------------------------------------------------------------

NC_000013.11:c3 ATACGAAACTCATTATAAGTGTAGGAACGTAAATATTATTATTATACTATAAAAATATAA
NC_000001.11:33 ------------------------------------------------------------
NC_000023.11:15 ------------------------------------------------------------
NC_000004.12:c1 ------------------------------------------------------------

NC_000013.11:c3 AGTAATACTTCTAAAATACATATACTCTTCATTTGTTAATCTTCCTTAATCCTGAACTAT
NC_000001.11:33 ------------------------------------------------------------
NC_000023.11:15 ------------------------------------------------------------
NC_000004.12:c1 ------------------------------------------------------------

NC_000013.11:c3 TTTCATTTTCTGCTTAGGTAATCTTAGTTTTCTGTAGATTCAGATGGATTTAAATGTTTT
NC_000001.11:33 ------------------------------------------------------------
NC_000023.11:15 ------------------------------------------------------------
NC_000004.12:c1 ------------------------------------------------------------

NC_000013.11:c3 TTGTAAACCTGCTTGTGGCTTCAAAACCACTAGGCACTATGCCAGCTATTAAAAGTAGAT
NC_000001.11:33 ------------------------------------------------------------
NC_000023.11:15 ------------------------------------------------------------
NC_000004.12:c1 ------------------------------------------------------------

NC_000013.11:c3 TTTTATTCTACAGCTTAATTGTCCATAATAAAACTTGGCCATTTAGGCTGGGTACGGTGG
NC_000001.11:33 ------------------------------------------------------------
NC_000023.11:15 ------------------------------------------------------------
NC_000004.12:c1 ------------------------------------------------------------

NC_000013.11:c3 CTCACGCCTGTAATCCCAGCACTTTGGGAGGCTAAGGCGGGCAGATCACAAGGTCAGGAG
NC_000001.11:33 ------------------------------------------------------------
NC_000023.11:15 ------------------------------------------------------------
```

```
NC_000004.12:c1 ------------------------------------------------------------

NC_000013.11:c3 TTCAAGACCAGCCTGGCCAGCATGGTGAAACCCCGTCTCTACTAAAAATAAAAAAAATTA
NC_000001.11:33 ------------------------------------------------------------
NC_000023.11:15 ------------------------------------------------------------
NC_000004.12:c1 ------------------------------------------------------------

NC_000013.11:c3 GCCGGGCATGGTGGCACATGCCTGTAGTCCCAGCTACTTGGGAGGCTGAGGCAAGAGAAT
NC_000001.11:33 ------------------------------------------------------------
NC_000023.11:15 ------------------------------------------------------------
NC_000004.12:c1 ------------------------------------------------------------

NC_000013.11:c3 CACCTGAACCCGGGAGGCGGAGGTTGCAGTGAGCCAAGATTGCGCCACTGCACTCCAGCC
NC_000001.11:33 ------------------------------------------------------------
NC_000023.11:15 ---------------------------------------------------------GGC
NC_000004.12:c1 ------------------------------------------------------------

NC_000013.11:c3 TGGGCGAGAGAGCGAGACTCCATCTCGGAAAAAAAAAAAAAAAAAACCATTTTTTTGCTA
NC_000001.11:33 ------------------------------------------------------------
NC_000023.11:15 GGGGCGGGCAGGCGAGACT-----------------------------------------
NC_000004.12:c1 ------------------------------------------------------------

NC_000013.11:c3 ATCCTGGAAGAGGACTTTCTCACACTCATATTACTTTTTTAAGTTGTCAAACTTTCTACA
NC_000001.11:33 ------------------------------------------------------------
NC_000023.11:15 ----------AGGTCTTTCCC---------------------------------------
NC_000004.12:c1 ------------------------------------------------------------

NC_000013.11:c3 TTGTTTCTTGTCTTACCAAAATCAAAATAAAACAATACAGAATAAAATGACTATATATAT
NC_000001.11:33 ------------------------------------------------------------
NC_000023.11:15 ------------------------------------------------------------
NC_000004.12:c1 ------------------------------------------------------------

NC_000013.11:c3 CACAAATTTTCTTGTGATTGTGCATTCCTAATCAGAATAAGATTTTTCTTCCACCTTCTA
NC_000001.11:33 ------------------------------------------------------------
NC_000023.11:15 ------------------------------------------------------------
NC_000004.12:c1 ------------------------------------------------------------

NC_000013.11:c3 GCTACTCTTATTCTTTTAAATTTGAATTTATGAAAATATATGTAATGAATGCACACATTT
NC_000001.11:33 ------------------------------------------------------------
NC_000023.11:15 ------------------------------------------------------------
NC_000004.12:c1 ------------------------------------------------------------

NC_000013.11:c3 ATTCATGAGCCTGACTTTTGAACATGACTATTTTTAAAAGGCCTATGATACTATTTTTTG
NC_000001.11:33 ------------------------------------------------------------
NC_000023.11:15 ------------------------------------------------------------
NC_000004.12:c1 ------------------------------------------------------------

NC_000013.11:c3 AGAATTGATTTATGTGAAAAATCTTACAAGAATATGAAAGTGAAAGCACATTAGGTAATA
NC_000001.11:33 ------------------------------------------------------------
NC_000023.11:15 ------------------------------------------------------------
NC_000004.12:c1 ------------------------------------------------------------

NC_000013.11:c3 TATTTAGAGAATTGCCTTTATTTAAGAAGAAGGAATTGCATGAAATTAGAACTCCCCCAG
```

```
NC_000001.11:33 ------------------------------------------------------------
NC_000023.11:15 ------------------------------------------------------------
NC_000004.12:c1 ------------------------------------------------------------


NC_000013.11:c3 AAAATACAGAATATAGAACCATCATCTCTATGGAGTGTGGCCCTTATCTATTTAATGGTA
NC_000001.11:33 ------------------------------------------------------------
NC_000023.11:15 ------------------------------------------------------------
NC_000004.12:c1 ------------------------------------------------------------


NC_000013.11:c3 TCTTGGTAGAGTGCTTAACACAGGGGCACAAAAAGATGGCCATTTAATAATAGCTTTTAA
NC_000001.11:33 ------------------------------------------------------------
NC_000023.11:15 ------------------------------------------------------------
NC_000004.12:c1 ------------------------------------------------------------


NC_000013.11:c3 TAACCAGGAGATGAACTTCCTTTGATATGAAGAAGTAACCAACATTACGCTTTTATTCTC
NC_000001.11:33 ------------------------------------------------------------
NC_000023.11:15 ------------------------------------------------------------
NC_000004.12:c1 ------------------------------------------------------------


NC_000013.11:c3 ATTGTTCCTAAGGGTGTAGGAAGCAGAGCATTTAATACAGTGCTTAAACAAGAAAGCTGC
NC_000001.11:33 ------------------------------------------------------------
NC_000023.11:15 ------------------------------------------------------------
NC_000004.12:c1 ------------------------------------------------------------


NC_000013.11:c3 ACACAAAATCAGAAGTGTACCATGTAGAAAGATGATTTACAAAAGCTCAATTACATGAAG
NC_000001.11:33 ------------------------------------------------------------
NC_000023.11:15 ------------------------------------------------------------
NC_000004.12:c1 ------------------------------------------------------------


NC_000013.11:c3 GATATGTAAGTAGACCCCTATGTGTCAGCGTGTGGATGCAAATTGATAAACACTTACTGA
NC_000001.11:33 ------------------------------------------------------------
NC_000023.11:15 ------------------------------------------------------------
NC_000004.12:c1 ------------------------------------------------------------


NC_000013.11:c3 AATTGCTTGTCAGAAAGCTAAGCGATCATAAAGATGCAGAAGTTCAAAGTGAAAAAGTCA
NC_000001.11:33 ------------------------------------------------------------
NC_000023.11:15 ------------------------------------------------------------
NC_000004.12:c1 ------------------------------------------------------------


NC_000013.11:c3 TCTTAGACTAAGTAAAATGAGAATCCCGTTATGCATTAAGGCAGTTTTGCAATACCTAAA
NC_000001.11:33 ------------------------------------------------------------
NC_000023.11:15 ------------------------------------------------------------
NC_000004.12:c1 ------------------------------------------------------------


NC_000013.11:c3 AGTTTTCACTAGCATTTGGAAAGTCATAAGTGAATTTTGTAGAGAATGAGAACTTGAATC
NC_000001.11:33 ------------------------------------------------------------
NC_000023.11:15 ------------------------------------------------------------
NC_000004.12:c1 ------------------------------------------------------------


NC_000013.11:c3 ATTATTACTTTCTTTCTTCTTTATTTTTAATAGAGAAAAGGTCTCGCTATGTTGACCAGG
NC_000001.11:33 ------------------------------------------------------------
NC_000023.11:15 ------------------------------------------------------------
NC_000004.12:c1 ------------------------------------------------------------
```

```
NC_000013.11:c3 CTGGGCTTAAACTCCTGGCCTCAAGTGATCCACCCATCTTGGCCTTCCAAAGTGCTGGGA
NC_000001.11:33 ------------------------------------------------------------
NC_000023.11:15 ------------------------------------------------------------
NC_000004.12:c1 ------------------------------------------------------------

NC_000013.11:c3 GTAACAGGCATGAGCCATTAGTGTACTTGTTTAAGCAGAGCGAGGCTTCCAGGCAATGCC
NC_000001.11:33 ------------------------------------------------------------
NC_000023.11:15 ------------------------------------------------------------
NC_000004.12:c1 ------------------------------------------------------------

NC_000013.11:c3 TCAGACTTCTTTTTCCCTCCTACTCTCTCGTGAGTCATCAAAACCAGTGTGCTGTCCTTC
NC_000001.11:33 ------------------------------------------------------------
NC_000023.11:15 ------------------------------------------------------------
NC_000004.12:c1 ------------------------------------------------------------

NC_000013.11:c3 ACTTACAAAGAAATCGCTGTTGACCCATCAAGGGGGAATGCACTGGGTTTGTAATAATCT
NC_000001.11:33 ------------------------------------------------------------
NC_000023.11:15 ------------------------------------------------------------
NC_000004.12:c1 ------------------------------------------------------------

NC_000013.11:c3 GTTCTGCTTTCTCTCGTCTTCCTCCTACCAAAGCCCATGCATTTACAAACTTCTAGGCAG
NC_000001.11:33 ------------------------------------------------------------
NC_000023.11:15 ------------------------------------------------------------
NC_000004.12:c1 ------------------------------------------------------------

NC_000013.11:c3 GCTAGGGAACTGTTGCTGCTGGCTGTAGATCAGGATTGGCATCACCAGACCTAGAGATAA
NC_000001.11:33 ------------------------------------------------------------
NC_000023.11:15 ------------------------------------------------------------
NC_000004.12:c1 ------------------------------------------------------------

NC_000013.11:c3 AATGCACGACCTCTAGCCTCATGAACACAAGCACTGATTCAGGTTATAAAGTATTTACAT
NC_000001.11:33 -----ACGACCCCCAGC-------------------------------------------
NC_000023.11:15 --TGTTTGGCCTCCGTA-------------------------------------------
NC_000004.12:c1 ------------------------------------------------------------

NC_000013.11:c3 TTGATTTTGGTGTCTTAAAGCCTTCTTATTTACATTTTTTATCTAATCTTCCCACCTCTA
NC_000001.11:33 ------------------------------------------------------------
NC_000023.11:15 ------------------------------------------------------------
NC_000004.12:c1 ------------------------------------------------------------

NC_000013.11:c3 CAGTTTTCTGCTGCCTAGTAACTTATACTTCTGCTCTTTATCCATGAGAGCGCACTCTGG
NC_000001.11:33 ------------------------------------------------------------
NC_000023.11:15 ------------------------------------------------------------
NC_000004.12:c1 ------------------------------------------------------------

NC_000013.11:c3 GAAAGGTGATATAGGAAAGAAGTTGATGTGATTAAGAGCAGGGGCTTTGGAAATCAGACT
NC_000001.11:33 ------------------------------------------------------------
NC_000023.11:15 ------------------------------------------------------------
NC_000004.12:c1 ------------------------------------------------------------

NC_000013.11:c3 GAGTAGCTCGAGGCCTGGTTCTGCTACTTAGTAAATATGTCATCTCAGACAAGTGACGCA
NC_000001.11:33 ------------------------------------------------------------
NC_000023.11:15 ------------------------------------------------------------
```

```
NC_000004.12:c1 ------------------------------------------------------------

NC_000013.11:c3 CTTATGAACATTGTCTTCTCAGTGTTCTCATCTATAAAATGGGGATAATGCCTACCTCCC
NC_000001.11:33 ------------------------------------------------------------
NC_000023.11:15 ------------------------------------------------------------
NC_000004.12:c1 ------------------------------------------------------------

NC_000013.11:c3 AGAATCCTGTAGAGGTTAAACAAGGTAATGTATTTAAGGTGCCTAGTACACTGCCTAGCA
NC_000001.11:33 ------------------------------------------------------------
NC_000023.11:15 ------------------------------------------------------------
NC_000004.12:c1 ------------------------------------------------------------

NC_000013.11:c3 TATACAAATGCATACTAGACAGCCAAAATGAAAAAGGAACTAGGCTTGTTTACACTAAGA
NC_000001.11:33 ------------------------------------------------------------
NC_000023.11:15 ------------------------------------------------------------
NC_000004.12:c1 ------------------------------------------------------------

NC_000013.11:c3 CTGGTGCATTTAACACAGGGATAATGTCTTAGCCCACAAAAATTCACTTTTTTGACAGGA
NC_000001.11:33 ------------------------------------------------------------
NC_000023.11:15 ------------------------------------------------------------
NC_000004.12:c1 ------------------------------------------------------------

NC_000013.11:c3 TATAAAGACATAATCTCTAGTTATGGAATGAGAAACAGTACACAGTTGGTGGAAAGAGGC
NC_000001.11:33 ------------------------------------------------------------
NC_000023.11:15 ------------------------------------------------------------
NC_000004.12:c1 ------------------------------------------------------------

NC_000013.11:c3 CAAGTTTGAAATGGGCCTGTCCGAGCACTTTTATAATCATATAATTCATAGGTACATGCC
NC_000001.11:33 ------------------------------------------------------------
NC_000023.11:15 ------------------------------------------------------------
NC_000004.12:c1 ------------------------------------------------------------

NC_000013.11:c3 TGTGTTGCTTAATGCAGAACTACAGACCTTTAACAACTTCTTTTTGCTACGTAAGACTTG
NC_000001.11:33 ------------------------------------------------------------
NC_000023.11:15 ------------------------------------------------------------
NC_000004.12:c1 ------------------------------------------------------------

NC_000013.11:c3 GCTGTGTCCTAGGATGAAGGAGTCTAGCTCTGTCACCCAGGCTGGAGTGCAGTGGCATTC
NC_000001.11:33 ------------------------------------------------------------
NC_000023.11:15 ------------------------------------------------------------
NC_000004.12:c1 ------------------------------------------------------------

NC_000013.11:c3 ACTACAACCTCCACTTCCTGGGTTCAAGCGATTCTTCTGCCTCAGCCTCCTGAGTAACTG
NC_000001.11:33 ------------------------------------------------------------
NC_000023.11:15 ------------------------------------------------------------
NC_000004.12:c1 ------------------------------------------------------------

NC_000013.11:c3 GGATTACAGATGCACACCACCATGCCCAACTAATTTTTATTTTTAATAGAGATGGGGTTT
NC_000001.11:33 ------------------------------------------------------------
NC_000023.11:15 ------------------------------------------------------------
NC_000004.12:c1 ------------------------------------------------------------

NC_000013.11:c3 CACCATGTTGGCCTGGTTGGTCTCAAACTCCTGACCTCAAGTGATCTCCCCGCCTTAGCC
```

```
NC_000001.11:33 ------------------------------------------------------------
NC_000023.11:15 ------------------------------------------------------------
NC_000004.12:c1 ------------------------------------------------------------

NC_000013.11:c3 TCCCAAAGTGCTGGGGTTATAGGTCTGAGCCACCACGCCTGGCCCTATTTTTATTTTTTT
NC_000001.11:33 ------------------------------------------------------------
NC_000023.11:15 ------------------------------------------------------------
NC_000004.12:c1 ------------------------------------------------------------

NC_000013.11:c3 ATACTGTGTATTAGAGTCCTAATACTGCCATTGCCATAACAAATGACACACAAACTTGGT
NC_000001.11:33 ------------------------------------------------------------
NC_000023.11:15 ------------------------------------------------------------
NC_000004.12:c1 ------------------------------------------------------------

NC_000013.11:c3 GGCTTAAAACAACAGAAATTGATTCTTCACAGTCCTGGAGGCCAGAAGTTCTGGAGGCTG
NC_000001.11:33 ------------------------------------------------------------
NC_000023.11:15 ------------------------------------------------------------
NC_000004.12:c1 ------------------------------------------------------------

NC_000013.11:c3 TGAGGGAGAAATCATCCCATGCATCTCTCCTGGCTTCTGGTTGTAGACAGTCCTTGGCAT
NC_000001.11:33 ------------------------------------------------------------
NC_000023.11:15 ------------------------------------------------------------
NC_000004.12:c1 ------------------------------------------------------------

NC_000013.11:c3 TCCTTGACTTGTAACTGCATCTATGCTTCCTGCTTCACAAGGCCCTCCCCTCTGTGTCTG
NC_000001.11:33 ------------------------------------------------------------
NC_000023.11:15 ------------------------------------------------------------
NC_000004.12:c1 ------------------------------------------------------------

NC_000013.11:c3 TCTCACAGTTCCCTCTACCTTTCTTTTGTAAGGACAAATGTCATTGGATTTAGGGTCCAT
NC_000001.11:33 ------------------------------------------------------------
NC_000023.11:15 ------------------------------------------------------------
NC_000004.12:c1 ------------------------------------------------------------

NC_000013.11:c3 CCTAAATCAGGATGATCTTATCTCAAGATTCTTTATTACATTTACAAAGACCTTTTTTTC
NC_000001.11:33 ------------------------------------------------------------
NC_000023.11:15 ------------------------------------------------------------
NC_000004.12:c1 ------------------------------------------------------------

NC_000013.11:c3 TTTTCTCCTGAGACAGGGAGTCTGACTCTGTCACCCAGGCTGGAGTGGGGTGACATGATC
NC_000001.11:33 ------------------------------------------------------------
NC_000023.11:15 ------------------------------------------------------------
NC_000004.12:c1 ------------------------------------------------------------

NC_000013.11:c3 ATAGCTCACTGCAGCCTTGAACTCCTTGGGCTTGAGCAGTCCTTCTGCCTCAGCCTCTCA
NC_000001.11:33 ------------------------------------------------------------
NC_000023.11:15 ------------------------------------------------------------
NC_000004.12:c1 ------------------------------------------------------------

NC_000013.11:c3 AGTAGCTGGAACTTCAGGTGCATGCCACCACACCTAGCTAATTTTTTATTGCTTATAGAG
NC_000001.11:33 ------------------------------------------------------------
NC_000023.11:15 ------------------------------------------------------------
NC_000004.12:c1 ------------------------------------------------------------
```

```
NC_000013.11:c3 ACGGGGGTCTCAATATGTTGCCTGGGCTGTTCTTGAACTCCTGGGCTCCAACGATTCTCC
NC_000001.11:33 ------------------------------------------------------------
NC_000023.11:15 ------------------------------------------------------------
NC_000004.12:c1 ------------------------------------------------------------

NC_000013.11:c3 CACCTTGGCTTCTCAAAGTTCTGGGACTACACATGCACACCACTGCACCTGGCCCCATTT
NC_000001.11:33 ------------------------------------------------------------
NC_000023.11:15 ------------------------------------------------------------
NC_000004.12:c1 ------------------------------------------------------------

NC_000013.11:c3 TTCCAAATAAGGTTACATCCACAGTTTCCAGGGTTAACACATGGACATATCTTTTTGGGG
NC_000001.11:33 ------------------------------------------------------------
NC_000023.11:15 ------------------------------------------------------------
NC_000004.12:c1 ------------------------------------------------------------

NC_000013.11:c3 GACCACCATTTGGCCCACTACACACTAGGTAAGAGAGAAGAACCAGAATCTGGAAAAATG
NC_000001.11:33 ------------------------------------------------------------
NC_000023.11:15 ------------------------------------------------------------
NC_000004.12:c1 ------------------------------------------------------------

NC_000013.11:c3 GGAGGATGAGCTGGTATGGGAGACTATACATCCTGGAAGATTCCACCCAGGGGGAACACT
NC_000001.11:33 ------------------------------------------------------------
NC_000023.11:15 ------------------------------------------------------------
NC_000004.12:c1 ------------------------------------------------------------

NC_000013.11:c3 AGAGCTTCACTGCCCGTATGTTGTCATGGGACCATTGAAATTAATTTTCTAGGTACAATT
NC_000001.11:33 ------------------------------------------------------------
NC_000023.11:15 ------------------------------------------------------------
NC_000004.12:c1 ------------------------------------------------------------

NC_000013.11:c3 AATCAACTGCCCAATGGACGAGTCTTCATCTTTAGCTGCAGGAGCACAGCAGTTGGGATT
NC_000001.11:33 ------------------------------------------------------------
NC_000023.11:15 ------------------------------------------------------------
NC_000004.12:c1 ------------------------------------------------------------

NC_000013.11:c3 TGCCTAATTGTCTTCTTCAAGTGTGTCAATGCTGTTTAGTTCCCAGGACTTGCTAACTAC
NC_000001.11:33 ------------------------------------------------------------
NC_000023.11:15 ------------------------------------------------------------
NC_000004.12:c1 ------------------------------------------------------------

NC_000013.11:c3 ATTTGGGTAAAGAGCAAGCAAGAGAGCTAGAAGTTCCTTCATGTGGTGTGGGCCTGGGAG
NC_000001.11:33 ------------------------------------------------------------
NC_000023.11:15 ------------------------------------------------------------
NC_000004.12:c1 ------------------------------------------------------------

NC_000013.11:c3 CCGTGATGGAGTGTTCTAACAAGAAAGAGCTGTAGAAGATTTCAGCAGCTATCAGTATGT
NC_000001.11:33 ------------------------------------------------------------
NC_000023.11:15 ------------------------------------------------------------
NC_000004.12:c1 ------------------------------------------------------------

NC_000013.11:c3 TAGCCCCTCAGTTGCCTCACCACCAGCACCCTCTTTTCAGCAGGCATTTACTGGGCCTCA
NC_000001.11:33 ------------------------------------------------------------
NC_000023.11:15 ------------------------------------------------------------
```

```
NC_000004.12:c1 ------------------------------------------------------------

NC_000013.11:c3 AGTGTGTGTGATTAGAAATTTGGTTAACAGGCTGGGCGCAGTGGTTCATGCCTGCAATCC
NC_000001.11:33 ------------------------------------------------------------
NC_000023.11:15 ------------------------------------------------------------
NC_000004.12:c1 ------------------------------------------------------------

NC_000013.11:c3 CAGCACTTTGGGAGGCCGAGGCGGGCGGATCACGAGGTCAGGAGTTTAAGACTAGCCCGG
NC_000001.11:33 ------------------------------------------------------------
NC_000023.11:15 --------------GCCGGCGCCGGCGGGTCGTTCTGT----------------------
NC_000004.12:c1 ---------------------------GGTCACGAGGT----------------------

NC_000013.11:c3 CCAACATGGTGAAACCCCATCTCTACTAAAAGTACAAAAAAATTAGCTGGGTGTGGGGGT
NC_000001.11:33 ------------------------------------------------------------
NC_000023.11:15 ------------------------------------------------------------
NC_000004.12:c1 ------------------------------------------------------------

NC_000013.11:c3 GCACACCTGTAATCCCAGCTACTCGGGAGACTGAGGCAGGAGAATTGCTTGAACCAGTAC
NC_000001.11:33 ------------------------------------------------------------
NC_000023.11:15 ------------------------------------------------------------
NC_000004.12:c1 ------------------------------------------------------------

NC_000013.11:c3 CCAGGAGGCGGAGGTTGCAGTGAACCAAGATCATGCCACTGCACTCCAGCCTGGTGACAG
NC_000001.11:33 ------------------------------------------------------------
NC_000023.11:15 ------------------------------------------------------------
NC_000004.12:c1 ------------------------------------------------------------

NC_000013.11:c3 AGTGAGATTCATTCTCAAAAAAAAAAAAAAAAAAATTTGGTTAACAAGTGAAATCAGTGG
NC_000001.11:33 ------------------------------------------------------------
NC_000023.11:15 ------------------------------------------------------------
NC_000004.12:c1 ------------------------------------------------------------

NC_000013.11:c3 TTAACAAGTTTTAAAATAAGGTTATATTCTGGACATTTTTGGTTTTTCTTTGAATGGAGC
NC_000001.11:33 ------------------------------------------------------------
NC_000023.11:15 -------------------------------------------------TTGAATGAA--
NC_000004.12:c1 ------------------------------------------------------------

NC_000013.11:c3 ACTGGAAATTGACTACAGGGGCTGAAAAAGGAAAGACTAAAGAAATCATCTAATTTTATT
NC_000001.11:33 ------------------------------------------------------------
NC_000023.11:15 ------------------------------------------------------------
NC_000004.12:c1 ------------------------------------------------------------

NC_000013.11:c3 CACCACAGTAGTTTGACTTGTGACCACAGAAAGAACAGTGCTAATACCAGGGCAATTTGC
NC_000001.11:33 ------------------------------------------------------------
NC_000023.11:15 ------------------------------------------------------------
NC_000004.12:c1 ------------------------------------------------------------

NC_000013.11:c3 CTGATTCTTGAAACTAGTAACTGTGCCTAAAATAGTTTGGAATGGTAGTTAGCTCGAGAA
NC_000001.11:33 ------------------------------------------------------------
NC_000023.11:15 --------TGATCCCAGCAGCCGCGCCCAA------------------------------
NC_000004.12:c1 ------------------------------------------------------------

NC_000013.11:c3 AATCCAGAGTGGTAGAGAACTGTGACTTTAGGATAAATTTTTCTACCAACTTGCATAGCA
```

```
NC_000001.11:33 ------------------------------------------------------------
NC_000023.11:15 ------------------------------------------------------------
NC_000004.12:c1 ------------------------------------------------------------


NC_000013.11:c3 AGCTCCCCACCAAAGCAGTGAGTATTGCACAAATTTTTTTAAATCAAAACTCGTGTTCAT
NC_000001.11:33 ------------------------------------------------------------
NC_000023.11:15 ------------------------------------------------------------
NC_000004.12:c1 ------------------------------------------------------------


NC_000013.11:c3 CAAATCTCTAAACCAAGATATCCTCCACATTAGTTACTACATGATTCCTTTTCGCATTTG
NC_000001.11:33 ------------------------------------------------------------
NC_000023.11:15 ------------------------------------------------------------
NC_000004.12:c1 ------------------------------------------------------------


NC_000013.11:c3 TGGTTCTTATCACAGTTTCTATTTAGGTGATGTTTAATTAGCCAATTATTTCAAATATTT
NC_000001.11:33 ------------------------------------------------------------
NC_000023.11:15 -----------------------TGGGCG-------------------------------
NC_000004.12:c1 ------------------------------------------------------------


NC_000013.11:c3 CATCACTGCTTACCTTTTAAGGTGCTTTAATTGATTTTTATAAAGACCAAAATTTTGAGG
NC_000001.11:33 --TTGCAGCCTATTTTTTAAA---------------------------------------
NC_000023.11:15 --TCCACGCCTGCTTAATATG---------------------------------------
NC_000004.12:c1 -------GCTTAGCGGTTAA----------------------------------------
                       **.*. .   **

NC_000013.11:c3 AAGCAAATGACATGATCTAAATGCTGGCTTGGGTGGGTACAATCTCTTTGTTATATAGAA
NC_000001.11:33 ------------------------------------------------------------
NC_000023.11:15 ------------------------------------------------------------
NC_000004.12:c1 ------------------------------------------------------------


NC_000013.11:c3 TAACTTCTTATTGTCATGGTAAGAAGAGATGAGAATTAAATGCATGGTTTCAAGGAGAAA
NC_000001.11:33 ------------------------------------------------------------
NC_000023.11:15 ------------------------------------------------------------
NC_000004.12:c1 ------------------------------------------------------------


NC_000013.11:c3 AATTTGTGGGTATAAATTGGATCGAGATATCATTGCTTCAGAGCAAGGGTCAGCAAACCC
NC_000001.11:33 ------------------------------------------------------------
NC_000023.11:15 ------------------------------------------------------------
NC_000004.12:c1 ------------------------------------------------------------


NC_000013.11:c3 AGCCCACTGCCTGTTTCTATATGGCCTACAGGCTAAAAATGGTTGTTACATCTTTAAAAG
NC_000001.11:33 ------------------------------------------------------------
NC_000023.11:15 ------------------------------------------------------------
NC_000004.12:c1 ------------------------------------------------------------


NC_000013.11:c3 GTTGGGAAAAAATCAAAAGAATATTTCATGACACATGAAAAGTTTATGAAATTCAAATTT
NC_000001.11:33 ------------------------------------------------------------
NC_000023.11:15 --------------------------------------------CATGAGGCCCGCAGCC
NC_000004.12:c1 ------------------------------------------------------------


NC_000013.11:c3 CAGGGTCCACAAATAAAGTTTTTTTGGAAC------ACACCTATTCATGTATGTATTGTG
NC_000001.11:33 -----------AATAAGGTTTTATTGGAACATAGCCACACTCATTGGTTTAGGTATTTTC
NC_000023.11:15 AATGGCCAGGCGAGGAGGCTGTTTTA----------------------------------
NC_000004.12:c1 ------------------------------------------------------------
```

```
NC_000013.11:c3 GAGGGTTGCTTTTGCCCTACAGTGGCAGAGTTT-ATAGCTGGAACAGAAACCATATGTGG
NC_000001.11:33 TATGGTTACTTTCGAGGTATAGTGGCAGAGCTGGGTACCTGCAACAGAAACC--CTGTGG
NC_000023.11:15 --------------------AACGGCAGAGCCCGCTGGCC--------AATCAGGCGGCT
NC_000004.12:c1 ------------------------------------------------------GTGAGG
                                                               .*

NC_000013.11:c3 CCCAGAAACTCTAAAACATTTACTCTCTGGCCCCTTACAGAAAAAGTTTGCCGACCACTG
NC_000001.11:33 TTCACCAAGCCTATCATATTTACTACCTGGCCCTCTA---------------GACCAATG
NC_000023.11:15 CTCGTGGAGGC-----------------------------------------AGCTAGCG
NC_000004.12:c1 CTCGCGACGT--------------------------------------------------
                ..*.  .

NC_000013.11:c3 CTGTAGAGCATGTGTTTGATAAAATTGCTGATTTTTGAAACTTAGTACTGTATAATGAGA
NC_000001.11:33 C-----------------------------------------------------------
NC_000023.11:15 C-----------------------------------------------------------
NC_000004.12:c1 ------------------------------------------------------------


NC_000013.11:c3 ACTTGAGCGTGTCCCCTTAAGTTCTAGTTTCACCTGAGGAAAGTAGGGACTCACTGTATC
NC_000001.11:33 ------------------------------------------------------------
NC_000023.11:15 ------------------------------------------------------------
NC_000004.12:c1 ------------------------------------------------------------


NC_000013.11:c3 AAATATTAATGGCAAAACCACAATTACTTTGCACCAAACTTAGTAGCAGCCAAGCTTCCC
NC_000001.11:33 ------------------------------------------------------------
NC_000023.11:15 ------------------------------------------------------------
NC_000004.12:c1 ------------------------------------------------------------


NC_000013.11:c3 TTCTGCGCACCAAGGGTGGAGTACAAGCCCTTGTCAGCTGGGCGATGCTCCATGGTACTC
NC_000001.11:33 ------------------------------------------------------------
NC_000023.11:15 -----------GAGGCTGGGGAGC------------GCTGAGCCGCGCGTCGTG------
NC_000004.12:c1 ------------------------------------------------------------


NC_000013.11:c3 AGAAAATGGCCCATAACCAAACGGGCCAGGCAGGTAAACCACAGTCTGAATAGGACCCTG
NC_000001.11:33 -------------------------------------------------------TCCTG
NC_000023.11:15 -------------------------------------------------------CCCTG
NC_000004.12:c1 ------------------------------------------------------------


NC_000013.11:c3 CCACCAGCACCACTGACAGACATCCAGTGCCACCAGTGCTGGAAATTATTTCTAAGTTGT
NC_000001.11:33 C-------------------------------------CTGGAGGTCATCTC--------
NC_000023.11:15 C-----------------------------------------------------------
NC_000004.12:c1 ------------------------------------------------------------


NC_000013.11:c3 CCTTGCATCTTTGCAACATAATCTCAAGGGTTGTGGTCCTGGAAAGAATTTTCTGGTTGA
NC_000001.11:33 ------------------------------------------------------------
NC_000023.11:15 ------------------------------------------------------------
NC_000004.12:c1 ------------------------------------------------------------


NC_000013.11:c3 CCACAGTGCCCAAACCTGCTTGTTACAGATAGTAGGGAAGTGAGGTAGACACTTAACTCA
NC_000001.11:33 ------------------------------------------------------------
NC_000023.11:15 ------------------------------------------------------------
NC_000004.12:c1 ------------------------------------------------------------


NC_000013.11:c3 CTCTTAATTTGCTGCTTTTCAGCTCATCCCTCCCATTATGATGCACAAAGAGATCTCACT
NC_000001.11:33 ------------------------------------------------------------
NC_000023.11:15 ----------GCTGC---------------------------------------------
```

```
NC_000004.12:c1 ------------------------------------------------------------

NC_000013.11:c3 TAAATGAGAAGCACTCCTAGGCAGCCAGAGAGTAAATATCAGACATCTTTTTTGCTTCTT
NC_000001.11:33 ------------------------------------------------------------
NC_000023.11:15 ------------------------------------------------------------
NC_000004.12:c1 ------------------------------------------------------------

NC_000013.11:c3 TAAGTGTGGTCCATGGACCAGCAGCATCAGTGCCCTCTGGAAGCATATCAGACATGCAGA
NC_000001.11:33 ------------------------------------------------------------
NC_000023.11:15 ------------------------------------------------------------
NC_000004.12:c1 ------------------------------------------------------------

NC_000013.11:c3 CCCAGACCTAACCTAATCAGAATTTGCATTTTAACAAGATCTCCAGGTGATTTGCTTATG
NC_000001.11:33 ------------------------------------------------------------
NC_000023.11:15 ------------------------------------------------------------
NC_000004.12:c1 ------------------------------------------------------------

NC_000013.11:c3 CGTTAAAAATTGACAAGCAACACAAATGAAGTTCTAATTTGGCAGGATTTCAGGCAGTAT
NC_000001.11:33 ------------------------------------------------------------
NC_000023.11:15 ------------------------------------------------------------
NC_000004.12:c1 ------------------------------------------------------------

NC_000013.11:c3 TATGAAGTGACAGGAAATATTTAGGCAGTTTGGGCACATATATTTTTGGCCCTGGGAGGT
NC_000001.11:33 ------------------------------------------------------------
NC_000023.11:15 ------------------------------------------------------------
NC_000004.12:c1 ------------------------------------------------------------

NC_000013.11:c3 TACCTATAAGCATGACAGATCTGCCTGACAATCTATGCCCCTGAGTAATGCCTGCTTTTT
NC_000001.11:33 ------------------------------------------------------------
NC_000023.11:15 ------------------------------------------------------------
NC_000004.12:c1 ------------------------------------------------------------

NC_000013.11:c3 ACAATTTCTTTCTTTCTTAAGCCCTCAGATTTAGAACCTCCAAAATTATAGCATTCCCCT
NC_000001.11:33 ------------------------------------------------------------
NC_000023.11:15 ------------------------------------------------------------
NC_000004.12:c1 ------------------------------------------------------------

NC_000013.11:c3 TGACAGCTCACTAGGAGTCCTGATGGTAAAAGTGGGCGGTGAGACAGTGACCATCTAGAT
NC_000001.11:33 ------------------------------------------------------------
NC_000023.11:15 ------------------------------------------------------------
NC_000004.12:c1 ------------------------------------------------------------

NC_000013.11:c3 GTGTGGGTTGTTTGTAAATAACAGCTTGCCTGTGTAGGTCACAGAGCCAAAAATTATTTT
NC_000001.11:33 ------------------------------------------------------------
NC_000023.11:15 ------------------------------------------------------------
NC_000004.12:c1 ------------------------------------------------------------

NC_000013.11:c3 AAAAGGACAAAGGTGAGTTATTGACCTCTAATATAAATTCAAAAATGGAAGCTTGATATG
NC_000001.11:33 ------------------------------------------------------------
NC_000023.11:15 ------------------------------------------------------------
NC_000004.12:c1 ------------------------------------------------------------

NC_000013.11:c3 AACCTGGTCACACTTGGATTCAAATCTTAGCTCTGTCTCTTATTTTGTGATTTTTATATA
```

```
NC_000001.11:33 ------------------------------------------------------------
NC_000023.11:15 ------------------------------------------------------------
NC_000004.12:c1 ------------------------------------------------------------


NC_000013.11:c3 ACTTACATAACCTTCCTGAGCCCGAATGTTCTCATCTCTAAAACGGGGTTAATAATTTAA
NC_000001.11:33 ---------------------------------------------------ATAATCTAG
NC_000023.11:15 ---------------------------------------------------CCAGACTAG
NC_000004.12:c1 ---------------------------------------------------GGGAGCTGG
                                                                     .. .*..

NC_000013.11:c3 GAGATAATCTATGTAAAGGACGTACCATAGTGCCCAGCCAGGACTATGTACTTCATTAAT
NC_000001.11:33 GGAATATT----------------------------------------------------
NC_000023.11:15 CGAACAAT----------------------------------------------------
NC_000004.12:c1 GGGACATT----------------------------------------------------
                 ..*.* *

NC_000013.11:c3 TGTAGCTATCATGTGTTTTAATTTGCTGCATTAGGAGACCATGGTTCTTTATGCTTTAGT
NC_000001.11:33 ------------------------------------------------------------
NC_000023.11:15 ------------------------------------------------------------
NC_000004.12:c1 ------------------------------------------------------------


NC_000013.11:c3 TCACTAAAGTGTTACACATTATCAACTCATTTTGCAGTGGGATTGTTAGAACCACTAATG
NC_000001.11:33 ------------------------------------------------------------
NC_000023.11:15 ------------------------------------------------------------
NC_000004.12:c1 ------------------------------------------------------------


NC_000013.11:c3 CTGAATGGTCTATGTTGAGTTTTAGAATTACCGATTCTTAGACATTCAACTTCTCTTTCT
NC_000001.11:33 ------------------------------------------------------------
NC_000023.11:15 ------------------------------------------------------------
NC_000004.12:c1 ------------------------------------------------------------


NC_000013.11:c3 GCCTCCAGTTTCTGTACTATGAGGCATCATAGAGTACAGTTGTTTATATCATGAGGTTAA
NC_000001.11:33 ------------------------------------------------------------
NC_000023.11:15 ------------------------------------------------------------
NC_000004.12:c1 ------------------------------------------------------------


NC_000013.11:c3 TTTGAAAAATTGCAATCATGAATGTTGGAGAGATAAAAGTACATATTTGAAAAACTGCTG
NC_000001.11:33 ------------------------------------------------------------
NC_000023.11:15 ------------------------------------------------------------
NC_000004.12:c1 ------------------------------------------------------------


NC_000013.11:c3 AATCCACTAAAGATATTCTTTAAATATGACTTCTGGAATATTGCCACTAGTGGCCACAAT
NC_000001.11:33 ------------------------------------------------------------
NC_000023.11:15 ------------------------------------------------------------
NC_000004.12:c1 ------------------------------------------------------------


NC_000013.11:c3 AGAAAAATCTCAGCAGAACCTTTCATAAGACAGAAGTGATTACTTTTAGGTATTATAAAC
NC_000001.11:33 ------------------------------------------------------------
NC_000023.11:15 ------------------------------------------------------------
NC_000004.12:c1 ------------------------------------------------------------


NC_000013.11:c3 CTTCTAAAATTTACTTCCAACAGTCTTTAGTGGTAAAGGTAAACAGATCTCTCCAAGCAC
NC_000001.11:33 ------------------------------------------------------------
NC_000023.11:15 ------------------------------------------------------------
NC_000004.12:c1 ------------------------------------------------------------
```

```
NC_000013.11:c3 AGGGCAGCGGTTTGTTTGTTTGTTTGTTTGTTTTTTGAGACTGAGTCTCTCTCTCTCTGT
NC_000001.11:33 ------------------------------------------------------------
NC_000023.11:15 ------------------------------------------------------------
NC_000004.12:c1 ------------------------------------------------------------

NC_000013.11:c3 TGCCCAGGCTGGAGTGCAGTGGTGTATTCTCAGCTCATTGCAACCTCTGCCTCCTGGGTT
NC_000001.11:33 ------------------------------------------------------------
NC_000023.11:15 ------------------------------------------------------------
NC_000004.12:c1 ------------------------------------------------------------

NC_000013.11:c3 CAAATGATTCTTGTGCCTCAGCCTCCCGAGTCAGCCTCCCGAGTAGCTGGGACTGCAGGC
NC_000001.11:33 ------------------------------------------------------------
NC_000023.11:15 ------------------------------------------------------------
NC_000004.12:c1 ------------------------------------------------------------

NC_000013.11:c3 ATAAGCCACCAAAACCCAGCTAAATTTTTTTTTTTTTTTTTTTTTTTGCATTTTTAGTAG
NC_000001.11:33 ------------------------------------------------------------
NC_000023.11:15 ------------------------------------------------------------
NC_000004.12:c1 ------------------------------------------------------------

NC_000013.11:c3 AGCCGGGTTTTTGCCATGTTGCCCAGCCTGGTCTTGAACTCCTGGGCTCAAGCAATCCGC
NC_000001.11:33 ------------------------------------------------------------
NC_000023.11:15 ------------------------------------------------------------
NC_000004.12:c1 ------------------------------------------------------------

NC_000013.11:c3 TCACCGCGGCCTCCCAGAGTGCTGGGATTACAGGCATGAGCCACTGCACGTGGCTCATGG
NC_000001.11:33 ------------------------------------------------------------
NC_000023.11:15 ------------------------------------------------------------
NC_000004.12:c1 ------------------------------------------------------------

NC_000013.11:c3 TTAGTTTCTTAATCATGTCCTATAACTGATCATTTGTCCACTTTTCATTTGAGAATATAT
NC_000001.11:33 ------------------------------------------------------------
NC_000023.11:15 ------------------------------------------------------------
NC_000004.12:c1 ------------------------------------------------------------

NC_000013.11:c3 CACCTTTTAATATTGATTCCAAAAAAATATGAATTGAAATTATTTTCACAGCTAAAATGT
NC_000001.11:33 ------------------------------------------------------------
NC_000023.11:15 ------------------------------------------------------------
NC_000004.12:c1 ------------------------------------------------------------

NC_000013.11:c3 TTTGGCCACCATGCCCACGAAGTTATTAAAATAAATTATTTATTGAAAAGCAAGATAGGT
NC_000001.11:33 ------------------------------------------------------------
NC_000023.11:15 ------------------------------------------------------------
NC_000004.12:c1 ------------------------------------------------------------

NC_000013.11:c3 TTCCCATTTACCATTTTAATTTTTATTGTCTTGTAAATTAAAGAACACAGTTATAAAGTA
NC_000001.11:33 ------------------------------------------------------------
NC_000023.11:15 ------------------------------------------------------------
NC_000004.12:c1 ------------------------------------------------------------

NC_000013.11:c3 GAGACCCAGCTAATAAAGCTTAAACAAGTTGTTAATAAATGAACTGGGTGCATAAAATCC
NC_000001.11:33 ------------------------------------------------------------
NC_000023.11:15 ------------------------------------------------------------
```

```
NC_000004.12:c1 ------------------------------------------------------------


NC_000013.11:c3 TTAAACCACATCTTCATTAGCAATGAGGCATCTTTATCCTCAATTGATTAAACGTTTCAC
NC_000001.11:33 ------------------------------------------------------------
NC_000023.11:15 ------------------------------------------------------------
NC_000004.12:c1 ------------------------------------------------------------


NC_000013.11:c3 TCACATAACAGTTTTGCCAAAGACACCTCTAACATGAAGGTCCTAGGTTACTTCTAACTA
NC_000001.11:33 --------------------GGACACCTCT------------------------------
NC_000023.11:15 ------------------ACAGGTACGTCTCGCA--------------------------
NC_000004.12:c1 ------------------------------------------------------------


NC_000013.11:c3 GAATCTAAATATAGAACTGCAGAAAAATAATTGTGCTAGCCTTTGAAGTGCATGGCTGTA
NC_000001.11:33 ------------------------------------------------------------
NC_000023.11:15 ------------------------------------------------------------
NC_000004.12:c1 ------------------------------------------------------------


NC_000013.11:c3 ATGTACCAAAAGAAAGAAATGGGCTTTTAATTTTTATTTGATGTATTTTTTTGAGACAGA
NC_000001.11:33 ------------------------------------------------------------
NC_000023.11:15 ------------------------------------------------------------
NC_000004.12:c1 ------------------------------------------------------------


NC_000013.11:c3 GTCTCTCTCAGTGTGTTGCGCAGGCTGGAGTGCAGTGGCACAGTCACAGCACGCTGCAGC
NC_000001.11:33 ------------------------------------------------------------
NC_000023.11:15 ------------------------------------------------------------
NC_000004.12:c1 ------------------------------------------------------------


NC_000013.11:c3 CTCAACCTGCTTGGGCTCAAGTGATCCTCCCACCTCAGCTTCCTGAGGAGCTGGGACTAC
NC_000001.11:33 ------------------------------------------------------------
NC_000023.11:15 ------------------------------------------------------------
NC_000004.12:c1 ------------------------------------------------------------


NC_000013.11:c3 AGGTGTCTGCCACCACACCCAGCTAATTTTAAAATTTTTTAGAGAGATGGGATCTTGCTA
NC_000001.11:33 ------------------------------------------------------------
NC_000023.11:15 ------------------------------------------------------------
NC_000004.12:c1 ------------------------------------------------------------


NC_000013.11:c3 TGTTGCCCAGGCTGCTGTCAAACTCCTGGAGTTAAGTGATCCTCCTGCCTCAGCCTCCCA
NC_000001.11:33 ------------------------------------------------------------
NC_000023.11:15 ----------------------------------------CCGCCCGCTCCCGCCGCC--
NC_000004.12:c1 ------------------------------------------------------------


NC_000013.11:c3 AAGTGCCAGGATTACAGGTGTGAGCCACTGCGCCCAGCCACAGCAATGTTTTATTAATAA
NC_000001.11:33 ------------------------------------------------------------
NC_000023.11:15 -----------------------GCCGCCGC-----------------------------
NC_000004.12:c1 ------------------------------------------------------------


NC_000013.11:c3 TAGCCAAGCAATGTTATCACTCCTGGCCAGGCAATATAGGGAACACTCTGTCAGAAATAA
NC_000001.11:33 ------------------------------------------------------------
NC_000023.11:15 ------------------------------------------------------------
NC_000004.12:c1 ------------------------------------------------------------


NC_000013.11:c3 AATGTGTGGGGATTTTACCCCATTGTTTGAACAGAGAACATCTAGATGCTTCTATAAATG
```

```
NC_000001.11:33 ------------------------------------------------------------
NC_000023.11:15 ------------------------------------------------------------
NC_000004.12:c1 ------------------------------------------------------------


NC_000013.11:c3 CAGAAATCGATTTGACCTCGAAGCAGAGCTTCCTTGTTCTAGTAAAACTTGCCTGAGTAA
NC_000001.11:33 ------------------------------------------------------------
NC_000023.11:15 ------------------------------------------------------------
NC_000004.12:c1 ------------------------------------------------------------


NC_000013.11:c3 TTGGCCTTAGAATTACTGGTTGTAATTCCTGCTGTAGACTAGTAAACTCTACTGTTAACA
NC_000001.11:33 ------------------------------------------------------------
NC_000023.11:15 ------------------------------------------------------------
NC_000004.12:c1 ------------------------------------------------------------


NC_000013.11:c3 GTACTTGAGGCTTAAAGCCCTGTGCTCTGGAGGCTGACATTTTTTTACCCCCCAAACCA
NC_000001.11:33 ------------------------------------------------------------
NC_000023.11:15 ------------------------------------------------------------
NC_000004.12:c1 ------------------------------------------------------------


NC_000013.11:c3 TGTAGAACATACATCGCAGACCTGGCTCTTACATGGCATCGCCATTAACACTTGGTCTAC
NC_000001.11:33 ------------------------------------------------------------
NC_000023.11:15 ------------------------------------CGCCGCCGCCGCCGC---------
NC_000004.12:c1 ------------------------------------------------------------


NC_000013.11:c3 TTCCTTCCTTCTTAGTGCTAATCCTTTATGGTTTGCTGATCTTGTGCCTTCCATAAACCA
NC_000001.11:33 ------------------------------------------------------------
NC_000023.11:15 ------------------------------------------------------------
NC_000004.12:c1 ------------------------------------------------------------


NC_000013.11:c3 TCTTAACCCTTTGGGAACACACTAGTTCAGATAATAACAGAGGAAACCAGAATCTTTCCC
NC_000001.11:33 ------------------------------------------------------------
NC_000023.11:15 ------------------------------------------------------------
NC_000004.12:c1 ------------------------------------------------------------


NC_000013.11:c3 CCTGATTGGCTAGTTATCAAGGCCAAGCGTCTGCCATTGTAATAGTAATACCGTACCGTC
NC_000001.11:33 ------------------------------------------------------------
NC_000023.11:15 ------------------------------------------------------------
NC_000004.12:c1 ------------------------------------------------------------


NC_000013.11:c3 TCCAACCAACCCAGCGCCAATTCTATCAGCTTCAGATGTGTTGGCCATGCCCTGACCTCA
NC_000001.11:33 ------------------------------------------------------------
NC_000023.11:15 ------------------------------------------------------------
NC_000004.12:c1 ------------------------------------------------------------


NC_000013.11:c3 GTGTCTCTAAGAATGAAGTCTTTGTACCTCTTAGATTCGTAGCCCTGTCACTGGTGTGGA
NC_000001.11:33 ------------------------------------------------------------
NC_000023.11:15 ------------------------------------------------------------
NC_000004.12:c1 ------------------------------------------------------------


NC_000013.11:c3 ACTGCTTTTCTTTTTCACTATTGGAAATGGATAATTTATACTTCTATTATGTGAAATCGT
NC_000001.11:33 ------------------------------------------------------------
NC_000023.11:15 ------------------------------------------------------------
NC_000004.12:c1 ------------------------------------------------------------
```

```
NC_000013.11:c3 TCTTGAGAAATATTTATTGTCACTATATAAACTTCTGTATAACAAAAATACTGTAATATA
NC_000001.11:33 ------------------------------------------------------------
NC_000023.11:15 ------------------------------------------------------------
NC_000004.12:c1 ------------------------------------------------------------

NC_000013.11:c3 AAAGTAAGTTGATATATAATTTAATTCATATACTTTTCATATAAGAGTACTTTATAGTCA
NC_000001.11:33 ------------------------------------------------------------
NC_000023.11:15 ------------------------------------------------------------
NC_000004.12:c1 ------------------------------------------------------------

NC_000013.11:c3 TTTACATGGAATTTTATATGTCAACAAGTTTATATATAAATTTTATAAACCTGAGTATTT
NC_000001.11:33 ------------------------------------------------------------
NC_000023.11:15 ------------------------------------------------------------
NC_000004.12:c1 ------------------------------------------------------------

NC_000013.11:c3 TATAGTGGTTCTATATAATTTTATATATAAATTTACTTCTATATGAGAGAGTATTTCTTA
NC_000001.11:33 ------------------------------------------------------------
NC_000023.11:15 ------------------------------------------------------------
NC_000004.12:c1 ------------------------------------------------------------

NC_000013.11:c3 CACAGAAATTTATACAGTGAGAATAAATACATCTTTTTTTTTTTTTTTTGAGACTGAGTC
NC_000001.11:33 ------------------------------------------------------------
NC_000023.11:15 ------------------------------------------------------------
NC_000004.12:c1 ------------------------------------------------------------

NC_000013.11:c3 TAGCTCTGTTGCCCAGGCTGGAGTACAGTGGTGCGACCTTGGCTCACTGCAACCTCTGCC
NC_000001.11:33 ------------------------------------------------------------
NC_000023.11:15 --------------------------------------------CGCCGCCGCCGCCGCC
NC_000004.12:c1 ------------------------------------------------------------

NC_000013.11:c3 TCCTGGGTTCAAGCGATTCTCCTGCCTCAGCCTCCCTAGTAGCTGGGATTACAGGCTTTT
NC_000001.11:33 ------------------------------------------------------------
NC_000023.11:15 GCC---------------------------------------------------------
NC_000004.12:c1 ------------------------------------------------------------

NC_000013.11:c3 GTTTTTAGTAGAGGTGGGATTTTGCCATGTTGACCAGGCCAGTCATGAACTCCTGACCTC
NC_000001.11:33 ------------------------------------------------------------
NC_000023.11:15 ------------------------------------------------------------
NC_000004.12:c1 ------------------------------------------------------------

NC_000013.11:c3 AGGTGATCCGCCTGCCTTGGCCTCCCAAAGTGCTTAGATTACAGGCGTGAGCCACTGCGC
NC_000001.11:33 ------------------------------------------------------------
NC_000023.11:15 --------------------------------------------------GCCGCAGCGC
NC_000004.12:c1 ------------------------------------------------------------

NC_000013.11:c3 CCGGCCAAGTATATCACAACTTCTGTATAAGACACTCTTTTCTCTGACTCCATCATTAAC
NC_000001.11:33 ------------------------------------------------------------
NC_000023.11:15 CCGCACAA----------------------------------------------------
NC_000004.12:c1 ------------------------------------------------------------

NC_000013.11:c3 CAGGGAGATGATGCAGTCCTTTACCTGATTATATCTCCACATTCACTCAGAAGTTGGGGG
NC_000001.11:33 ------------------------------------------------------------
NC_000023.11:15 ------------------------------------------------------------
```

```
NC_000004.12:c1 ------------------------------------------------------------

NC_000013.11:c3 ACCAGTTGCTGATGTTTTCACTGTCCTTCTGGCCCCTCCCTCATGTCTGCATTTCCTGTG
NC_000001.11:33 ------------------------------------------------------------
NC_000023.11:15 ------------------------CTTTCCGGCCCGCGC---------------------
NC_000004.12:c1 ------------------------------------------------------------

NC_000013.11:c3 GATGGGATTTTCCTCTCTCATTTCAGTAAATTCCACTATCTTGATGATCCGCTTTGTCTT
NC_000001.11:33 ------------------------------------------------------------
NC_000023.11:15 ------------------------------------------------------------
NC_000004.12:c1 ------------------------------------------------------------

NC_000013.11:c3 CTGTCTTCCAGGTCTGTGAACTCCATCCCAGCAGCATTTAAATCATGCTTATGTATTTCA
NC_000001.11:33 ------------------------------------------------------------
NC_000023.11:15 ------------------------------------------------------------
NC_000004.12:c1 ------------------------------------------------------------

NC_000013.11:c3 GTCTTAAAGAAAACCTCTTGCTTAGCCACAAATTCCTTTCTGGCGACTCCCCTTTATTTC
NC_000001.11:33 ------------------------------------------------------------
NC_000023.11:15 ------------------------------------------------------------
NC_000004.12:c1 ------------------------------------------------------------

NC_000013.11:c3 TAACTATGCCTCTTCAGTGTCTCAGAAGAGTGGTCTGCCCTCTGCTTCCACTCTTACACT
NC_000001.11:33 -------------------TCCCGGGCATGTGGTCTGGTC--------------------
NC_000023.11:15 -------------------CGCCGTGAGCGCGCCCTGCCG--------------------
NC_000004.12:c1 ---------------------------------TCTGTCA--------------------
                                                 .*** .

NC_000013.11:c3 TCTCTCTCCTCAGTCTTCAACCCAGTGTCATCTGTCATCGTTCCTACTACTTCACTGACT
NC_000001.11:33 ------------------------------------------------------------
NC_000023.11:15 ------------------------------------------------------------
NC_000004.12:c1 ------------------------------------------------------------

NC_000013.11:c3 TTTTTTCTCGCAACAGGGTCTTTCTTTGTCACCAATTGGCTGACTACTTGGCAGCAGTGT
NC_000001.11:33 ------------------------------------------------------------
NC_000023.11:15 ------------------------------------------------------------
NC_000004.12:c1 ------------------------------------------------------------

NC_000013.11:c3 CCTTCCTTTAAGAACATCTTCTGCCTCTTCTCCGAGTCCACCATCCTACCTCTCTCATGA
NC_000001.11:33 ------------------------------------------------------------
NC_000023.11:15 ------------------------------------------------------------
NC_000004.12:c1 ------------------------------------------------------------

NC_000013.11:c3 CAGCTGCTCAGGCTCCTTTGCAAGCCTCTCTTCTTGTGTTGTCACCACAGCCCCACTTAA
NC_000001.11:33 ------------------------------------------------------------
NC_000023.11:15 ------------------------------------------------------------
NC_000004.12:c1 ------------------------------------------------------------

NC_000013.11:c3 TGTCTGTGTTCCTGGGGAACTTCTCCTATTCTTTCTTCTTTTCTCATTTGACACACACAA
NC_000001.11:33 ------------------------------------------------------------
NC_000023.11:15 ------------------------------------------------------------
NC_000004.12:c1 ------------------------------------------------------------

NC_000013.11:c3 GCTAGGTGATCTCAGCTACCTCCATGATCTTAATACCATCAGGTAGAGTTGCCTCTTCCT
```

```
NC_000001.11:33 ------------------------------------------------------------
NC_000023.11:15 ------------------------------------------------------------
NC_000004.12:c1 ------------------------------------------------------------


NC_000013.11:c3 CCTGAGCTCCAGACTAGAAAGTCACTGTTGACCATTCCCTCCTTTCTGAAAATACTGCCG
NC_000001.11:33 ------------------------------------------------------------
NC_000023.11:15 ------------------------------------------------------------
NC_000004.12:c1 ------------------------------------------------------------


NC_000013.11:c3 TCTATTGGCTGCCCTAATTCCAGAATCAGGTTTTCCTCCTACTTTTTTTTACCAAATGTG
NC_000001.11:33 ------------------------------------------------------------
NC_000023.11:15 ------------------------------------------------------------
NC_000004.12:c1 ------------------------------------------------------------


NC_000013.11:c3 TCTCAGTCTTCTGAAATCTGTCACTCTCCTGCTTAGAGCCCTTCTGTTGTTGGAATTCAG
NC_000001.11:33 ------------------------------------------------------------
NC_000023.11:15 ------------------------------------------------------------
NC_000004.12:c1 ------------------------------------------------------------


NC_000013.11:c3 TATGAATTATAAATCCAGGGGGTACCTGGCTCCCACCGTGGTGCTTCCCTCCTTGCACTA
NC_000001.11:33 ------------------------------------------------------------
NC_000023.11:15 ------------------------------------------------------------
NC_000004.12:c1 ------------------------------------------------------------


NC_000013.11:c3 GCTCCAGCTATCCTCATCTGACTCTTCTTTCATTTCTACAGACTTCGCTCTGTTATCTTA
NC_000001.11:33 ------------------------------------------------------------
NC_000023.11:15 ------------------------------------------------------------
NC_000004.12:c1 ------------------------------------------------------------


NC_000013.11:c3 TGCTGTTGGAGAAAAAAAAAAAAACATATATAACCTGTAGCCAGTTCTGATTTATGAACT
NC_000001.11:33 ------------------------------------------------------------
NC_000023.11:15 ------------------------------------------------------------
NC_000004.12:c1 ------------------------------------------------------------


NC_000013.11:c3 CCAGCCTCTACTGGTCTAAGAAGTTATTTCAAATGTCCATCGCTCTTCAACCCATCAGTA
NC_000001.11:33 ------------------------------------------------------------
NC_000023.11:15 ------------------------------------------------------------
NC_000004.12:c1 ------------------------------------------------------------


NC_000013.11:c3 TTGTCACCTTTCTCATGTCAGTTGACTTTGCCATCTCCTTCATGGAAAAAGAAAAAAGTG
NC_000001.11:33 ------------------------------------------------------------
NC_000023.11:15 ------------------------------------------------------------
NC_000004.12:c1 ------------------------------------------------------------


NC_000013.11:c3 AGCCTATCATGCTAGAATTTTCTGAGCCTCTTCTTTAACACTTAGAAATTTATCTCCATG
NC_000001.11:33 ------------------------------------------------------------
NC_000023.11:15 ------------------------------------------------------------
NC_000004.12:c1 ------------------------------------------------------------


NC_000013.11:c3 GGAACCTATCCTTTCTTCTTTATTCTCTGTCCTAGGATAGGAAATATGCTCCTACCTCTT
NC_000001.11:33 ------------------------------------------------------------
NC_000023.11:15 ------------------------------------------------------------
NC_000004.12:c1 ------------------------------------------------------------
```

```
NC_000013.11:c3 TAAGCCCAACCCCAAGCCTTCCCAAATCTTGAGGCGTGCGTTTTAACTTCTCTATTGATA
NC_000001.11:33 ------------------------------------------------------------
NC_000023.11:15 ------------------------------------------------------------
NC_000004.12:c1 ------------------------------------------------------------

NC_000013.11:c3 TCGGCCTTTGTCTGAAGTGGTTTCATGCAACGTTACACCTTTATACTCCTTGGGAGGAAA
NC_000001.11:33 ------------------------------------------------------------
NC_000023.11:15 ------------------------------------------------------------
NC_000004.12:c1 ------------------------------------------------------------

NC_000013.11:c3 AACAGCCCCTCGACTCCACATTTCCTTCTAGCTACTGACCTAATTCTCCTAACCTTCAGA
NC_000001.11:33 ------------------------------------------------------------
NC_000023.11:15 ------------------------------------------------------------
NC_000004.12:c1 ------------------------------------------------------------

NC_000013.11:c3 TTTGAGCTTCTTGAAAGAGTAGCTTATAATGGGGTATCCCTACTTCCTTATGCCTGCTTG
NC_000001.11:33 ------------------------------------------------------------
NC_000023.11:15 ------------------------------------------------------------
NC_000004.12:c1 ------------------------------------------------------------

NC_000013.11:c3 ATGACTCAACCCACTGCAGTTAGCTTCTGCTCCCTGATGGAATTGTTCTCGCTAAGGCTA
NC_000001.11:33 ------------------------------------------------------------
NC_000023.11:15 ------------------------------------------------------------
NC_000004.12:c1 ------------------------------------------------------------

NC_000013.11:c3 CCGATAAGCTTCATATTTGCCAAAACCAAAGGCTGATTTTTAACCTTTATCCTACTTTGT
NC_000001.11:33 ------------------------------------------------------------
NC_000023.11:15 ------------------------------------------------------------
NC_000004.12:c1 ------------------------------------------------------------

NC_000013.11:c3 GCCTTCGTGGCATTAGACACTGCTGACCACCCCACCATGGAACTTTTTTCCTGCTTTGAC
NC_000001.11:33 ------------------------------------------------------------
NC_000023.11:15 ------------------------------------------------------------
NC_000004.12:c1 ------------------------------------------------------------

NC_000013.11:c3 TTACATGACCCTACTTTTTCCTGGTGCTTCTGTCTTTGTAGCTTTTTCCCTTGCAGCCTC
NC_000001.11:33 ------------------------ATGCTACTGTCCTTGCAGGCATGCTTGCTCTAGGCTG
NC_000023.11:15 --------------------CCGCCTCCCCCTGCCTCTACTCCCCATTCCCTTCCCGCCCC
NC_000004.12:c1 ------------------------------------------------------------

NC_000013.11:c3 TTTGGGGGTGTCCTCTTTTCTTATTCATTCATTCAACAAATGTTTGTGAAGCTGGATCCT
NC_000001.11:33 T-----------------------------------------------------------
NC_000023.11:15 C-----------------------------------------------------------
NC_000004.12:c1 ------------------------------------------------------------

NC_000013.11:c3 GGAGCTGCAGTGGTAGGTGGGATATAATCTCTACCCTCATTGCTACAGGATACAAGAGAA
NC_000001.11:33 ------------------------------------------------------------
NC_000023.11:15 ------------------------------------------------------------
NC_000004.12:c1 ------------------------------------------------------------

NC_000013.11:c3 ACAATCAAATAATTACACAAATCACTGTAAATAGTTACACACACATTCTGGTAAATGCTG
NC_000001.11:33 ------------------------------------------------------------
NC_000023.11:15 ------------------------------------------------------------
```

```
NC_000004.12:c1 ------------------------------------------------------------


NC_000013.11:c3 GGAGGGGGTGCCTTGGGGGTCTCAATGGTGAGAAACACGTGACATCTGAGTCATGTGAAG
NC_000001.11:33 --------------------------------------GTGAC-----------------
NC_000023.11:15 ------------------------------------------------------------
NC_000004.12:c1 ------------------------------------------------------------


NC_000013.11:c3 GCATCATCCATCTCACTGTACTACCTTTCCTGCTTTTTGGGAATATGAGATTTTGGTGAA
NC_000001.11:33 ------------------------------------------------------------
NC_000023.11:15 ------------------------------------------------------------
NC_000004.12:c1 ------------------------------------------------------------


NC_000013.11:c3 TGAATGCATATTCATCTTAGTCATACTCTTAATTGTTTAATAATTGTCAGTCACATACCC
NC_000001.11:33 ------------------------------------------------------------
NC_000023.11:15 ------------------------------------------------------------
NC_000004.12:c1 ------------------------------------------------------------


NC_000013.11:c3 TTTCACAATCTGCTGTTTCCAAGTGTAGATATTTTTATCCTTTTAATATAAATTCTTCTC
NC_000001.11:33 ------------------------------------------------------------
NC_000023.11:15 ------------------------------------------------------------
NC_000004.12:c1 ------------------------------------------------------------


NC_000013.11:c3 CTCAGTCCCTTGTTAGTAGTTATGGAGAGACTTGGGCTGAAATACATTTTTGTAAATCTT
NC_000001.11:33 ------------------------------------------------------------
NC_000023.11:15 ------------------------------------------------------------
NC_000004.12:c1 ------------------------------------------------------------


NC_000013.11:c3 GAGTTCAGTTATGCCTTAGTTGAGCAGCAGCAGCTAGAACTTCCCAGAATTCCAGGTGTC
NC_000001.11:33 ------------------------------------------------------------
NC_000023.11:15 ------------------------------------------------------------
NC_000004.12:c1 ------------------------------------------------------------


NC_000013.11:c3 CATCTATTTGCAGCTGAATTCCATCCGCTTTCTTAAAAACTGATTTCCCTATGTGGCTAT
NC_000001.11:33 ------------------------------------------------------------
NC_000023.11:15 ------------------------------------------------------------
NC_000004.12:c1 ------------------------------------------------------------


NC_000013.11:c3 CTAAATTTAATCTCATGTGTTAGAGCAGGCAGCCTACTACACGCACATCTCTGAACACTA
NC_000001.11:33 ------------------------------------------------------------
NC_000023.11:15 ------------------------------------------------------------
NC_000004.12:c1 ------------------------------------------------------------


NC_000013.11:c3 CTTTGCAATATGCCATATTGCTGAAGAGGTGTGGACAGAGTACACAGTTGGTTTGAGTCT
NC_000001.11:33 ------------------------------------------------------------
NC_000023.11:15 ------------------------------------------------------------
NC_000004.12:c1 ------------------------------------------------------------


NC_000013.11:c3 GGCTTCCATCACTTCATTCTTTTGACAGGTTATTTAAATTCACTGCCCAAGGAAGCAAAA
NC_000001.11:33 ------------------------------------------------------------
NC_000023.11:15 ------------------------------------------------------------
NC_000004.12:c1 ------------------------------------------------------------


NC_000013.11:c3 CTAGTAAACCAGCAATGAAACTCGGGCCATCTTACCGGGATTGGGAGTTCACCCTGTAAA
```

```
NC_000001.11:33 ------------------------------------------------------------
NC_000023.11:15 ------------------------------------------------------------
NC_000004.12:c1 ------------------------------------------------------------


NC_000013.11:c3 ATAAGCCATGGTCTTGTAAATGACCATTGTCAATGGTGAGGAAAGGTTGTCCCATAGTGT
NC_000001.11:33 ------------------------------------------------------------
NC_000023.11:15 ------------------------------------------------------------
NC_000004.12:c1 ------------------------------------------------------------


NC_000013.11:c3 GCTTCCCTAGCCACTCTAGAAGCACAGGAAGCACTGGCCTTGGAGTGAGGCCAGTGCCTA
NC_000001.11:33 ------------------------------------------------------------
NC_000023.11:15 ------------------------------------------------------------
NC_000004.12:c1 ------------------------------------------------------------


NC_000013.11:c3 ACGTACCTTGGTAAGGAATGATCTAAGGCAGTGCTTCTCAACAGGGGCAATTTTTCCTCC
NC_000001.11:33 ------------------------------------------------------------
NC_000023.11:15 ------------------------------------------------------------
NC_000004.12:c1 ------------------------------------------------------------


NC_000013.11:c3 ACCCGCACCCCCCACCCCAGGTACATTTGGCAATGTAAGAAACAGACTCACCTATTTCTA
NC_000001.11:33 ------------------------------------------------------------
NC_000023.11:15 ------------------------------------------------------------
NC_000004.12:c1 ------------------------------------------------------------


NC_000013.11:c3 GAGAAGTCCTCAAAAGATATTTTTAAAAATTAAAAGTAACTATAGATTCAGAGCTTTCTG
NC_000001.11:33 ------------------------------------------------------------
NC_000023.11:15 ------------------------------------------------------------
NC_000004.12:c1 ------------------------------------------------------------


NC_000013.11:c3 GAAAAGTGTACAGATCATTTATTCATTCAACACATTCTGAACACCTACTGTGAGTCACGA
NC_000001.11:33 ------------------------------------------------------------
NC_000023.11:15 ------------------------------------------------------------
NC_000004.12:c1 ------------------------------------------------------------


NC_000013.11:c3 GCCAGGGTTAACCTTCAACAAGTTTGGTCCTTGCCCTCAAGAAGATTATAATCCATCTGC
NC_000001.11:33 ------------------------------------------------------------
NC_000023.11:15 ------------------------------------------------------------
NC_000004.12:c1 ------------------------------------------------------------


NC_000013.11:c3 ATTGGTGGTCCTCAAATTTTACCGTGCATCAGAATCCCTTGGAGGCTTGTGAAAAACTCA
NC_000001.11:33 ------------------------------------------------------------
NC_000023.11:15 ------------------------------------------------------------
NC_000004.12:c1 ------------------------------------------------------------


NC_000013.11:c3 TTTCTCGGCCCTACTCCCAGAGTTTCTGATTCAGTGGGTCTGGGGTGGGGCCCAAGAATT
NC_000001.11:33 ------------------------------------------------------------
NC_000023.11:15 ------------------------------------------------------------
NC_000004.12:c1 ------------------------------------------------------------


NC_000013.11:c3 TGCATTTCTAACAAGTTCAGGATCACACTTGAAAACTATTGCTCTAGGGGAATAAGGAAA
NC_000001.11:33 ------------------------------------------------------------
NC_000023.11:15 ------------------------------------------------------------
NC_000004.12:c1 ------------------------------------------------------------
```

```
NC_000013.11:c3 GAAAGCTCCTGGGGTTTTGAGGATCAACATTTGCAGTATCTGGGGGACACTTGAATGGCT
NC_000001.11:33 ------------------------------------------------------------
NC_000023.11:15 ------------------------------------------------------------
NC_000004.12:c1 ------------------------------------------------------------

NC_000013.11:c3 TGATTCTATCTGTCAGAATGTCTTTTTGGAAAAAGGAAGGTTTCATAGCCCATGGCTGTT
NC_000001.11:33 ------------------------------------------------------------
NC_000023.11:15 ------------------------------------------------------------
NC_000004.12:c1 ------------------------------------------------------------

NC_000013.11:c3 AGAGACAGACTTGGTGTTTCACTAACAACTAGGATTATTGCTAAAAGCCCGGACATTTTA
NC_000001.11:33 ------------------------------------------------------------
NC_000023.11:15 ------------------------------------------------------------
NC_000004.12:c1 ------------------------------------------------------------

NC_000013.11:c3 ATGCCTATTTCTCTAAAGCCAGCAAGGTTGAACATTTTTTCATCTTAAAAAAAAAATCTT
NC_000001.11:33 ------------------------------------------------------------
NC_000023.11:15 ------------------------------------------------------------
NC_000004.12:c1 ------------------------------------------------------------

NC_000013.11:c3 TTTTTACATGACTTATGTTTTCATTTCAGTTATTTTTTTAAAAAATTTTCTGCTGAGGTC
NC_000001.11:33 ------------------------------------------------------------
NC_000023.11:15 ------------------------------------------------------------
NC_000004.12:c1 ------------------------------------------------------------

NC_000013.11:c3 TTGGTGAGCAAATGGTTTTAAACTCCACACTTTTGGCTAAGAATGAGATGTAAGATATAG
NC_000001.11:33 ------------------------------------------------------------
NC_000023.11:15 ------------------------------------------------------------
NC_000004.12:c1 ------------------------------------------------------------

NC_000013.11:c3 ACACTAGTTCTTAAAATTCAGCACTGTTCACTTTTGCCAAGTGAATATAAACTGAATTGC
NC_000001.11:33 ------------------------------------------------------------
NC_000023.11:15 ------------------------------------------------------------
NC_000004.12:c1 ------------------------------------------------------------

NC_000013.11:c3 ATCAAATTACAGAGAACAGTGTATCTAGAATATTGTTTTATTTTATCATTTTTACCTTTC
NC_000001.11:33 ------------------------------------------------------------
NC_000023.11:15 ------------------------------------------------------------
NC_000004.12:c1 ------------------------------------------------------------

NC_000013.11:c3 CATTGAAAGATGAAATCAGGCGATCAAAGTAGCAAGGGTTAAGTTTCTAGACTACCACAA
NC_000001.11:33 ------------------------------------------------------------
NC_000023.11:15 ------------------------------------------------------------
NC_000004.12:c1 ------------------------------------------------------------

NC_000013.11:c3 ATTAGAATTGACAAGAAAAGCTTTCTGATCCATATTTGCCTGGGAAGGCTCCAAGGGAAA
NC_000001.11:33 ------------------------------------------------------------
NC_000023.11:15 ------------------------------------------------------------
NC_000004.12:c1 ------------------------------------------------------------

NC_000013.11:c3 CACAAGAAGCAGCAACTCAAATCCTGTTCTCTTCATTATCCTTTTTTTTTTAATAGCTCT
NC_000001.11:33 ------------------------------------------------------------
NC_000023.11:15 ------------------------------------------------------------
```

```
NC_000004.12:c1 ------------------------------------------------------------

NC_000013.11:c3 TGCATAGGCTCGGTTTTTGGAAGTTGTATGCTGCTAATTTAGGTATATATGTCATTACAA
NC_000001.11:33 ------------------------------------------------------------
NC_000023.11:15 ------------------------------------------------------------
NC_000004.12:c1 ------------------------------------------------------------

NC_000013.11:c3 TTTTAGCATGGATCCTTCTCAGGCATACACAAGAGATGCCAGACATGTGAAAAAAGGAGG
NC_000001.11:33 ------------------------------------------------------------
NC_000023.11:15 ------------------------------------------------------------
NC_000004.12:c1 ------------------------------------------------------------

NC_000013.11:c3 GAGCCAAGCACAGGAACCTCAGGGACACTGGCATAACTGTTCTAGAGTCAGAAACCACAT
NC_000001.11:33 ------------------------------------------------------------
NC_000023.11:15 ------------------------------------------------------------
NC_000004.12:c1 ------------------------------------------------------------

NC_000013.11:c3 GAACACATACCGAATTGATAGACATGAGGTTCAAGTCTATTCTGTGCCCTGTCTCGCTCT
NC_000001.11:33 ------------------------------------------------------------
NC_000023.11:15 ------------------------------------------------------------
NC_000004.12:c1 ------------------------------------------------------------

NC_000013.11:c3 CTTCTTAACATCCGCTTCTCTTTCTGGAACTGTATTTCATATAACTCTTTAGGTGAGAAC
NC_000001.11:33 ------------------------------------------------------------
NC_000023.11:15 ----------TCCGCCTTCCCTCC------------------------------------
NC_000004.12:c1 ------------------------------------------------------------

NC_000013.11:c3 TAGAGCTATTCAGTCAAAGACTAAAAAGAGGCAAGGTTGGACCAAGTTTAAGAATCAGGT
NC_000001.11:33 ------------------------------------------------------------
NC_000023.11:15 ------------------------------------------------------------
NC_000004.12:c1 ------------------------------------------------------------

NC_000013.11:c3 AACAATTAGCTTTGTGATGGTTAATGAAATCATTTGTCTCGGCATAGACTCTTCCCCTGG
NC_000001.11:33 ------------------------------------------------------------
NC_000023.11:15 ------------------------------------------------------------
NC_000004.12:c1 ------------------------------------------------------------

NC_000013.11:c3 AAGTGTCTTGCAAAAGCTGGTTGTTGGTGAAGTAGGATAGAGAGTTAAGGGAATGGGTTA
NC_000001.11:33 ------------------------------------------------------------
NC_000023.11:15 ------------------------------------------------------------
NC_000004.12:c1 ------------------------------------------------------------

NC_000013.11:c3 GATGACTTCCAAAGCCCTTTTATCTACAAGGTGAATCTGTAAGTGTAAGAGTCTTAGGTC
NC_000001.11:33 ------------------------------------------------------------
NC_000023.11:15 ------------------------------------------------------------
NC_000004.12:c1 ------------------------------------------------------------

NC_000013.11:c3 AACATTTATTGGAGTGCAGATATGGACAGAGCTCTGTGCTAGGCACTGGGCCGCAGAGAC
NC_000001.11:33 ------------------------------------TGCCAGGCACTTGG----------
NC_000023.11:15 ------------------------------------TGCTAGGCGGCCGG----------
NC_000004.12:c1 ------------------------------------GGAAAGGCGTTTTG----------
                                                     *  ****. .  *

NC_000013.11:c3 ATGAAACATGGTTTTGACCCTTGGAGGCTTGCTCCCTAATCAAGGGGACCAGGTAGACAG
```

```
NC_000001.11:33 ------------------------------------------------------------
NC_000023.11:15 ------------------------------------------------------------
NC_000004.12:c1 ------------------------------------------------------------

NC_000013.11:c3 ACATTTAAATAAAATAAAACAAAAACGCAAAGGAACATAGACTAGGCATTCTTTTTAGCT
NC_000001.11:33 ------------------------------------------------------------
NC_000023.11:15 ------------------------------------------------------------
NC_000004.12:c1 ------------------------------------------------------------

NC_000013.11:c3 CTGGAACTCGCGTTGAGGAGGATCACATTGATTACTTTTAGGGTTAACTCCTAGAACTGA
NC_000001.11:33 ------------------------------------------------------------
NC_000023.11:15 ------------------------------------------------------------
NC_000004.12:c1 ------------------------------------------------------------

NC_000013.11:c3 GGAGCACCTTATTTCAGTTAGAACTTCCCATATAAAAATAAAGATGAAAACATGGAAAAA
NC_000001.11:33 ------------------------------------------------------------
NC_000023.11:15 -----------------------------------------------------GAAGGAA
NC_000004.12:c1 ------------------------------------------------------------

NC_000013.11:c3 GATACCCTTAAAAAATTGGAATTCAGTGCAAATTTTATTAGACTACAAAAACATATCATA
NC_000001.11:33 ------------------------------------------------------------
NC_000023.11:15 G-----------------------------------------------------------
NC_000004.12:c1 ------------------------------------------------------------

NC_000013.11:c3 CAATTATTATTTTAGATATATTTATGTAAAGTCATAAAAGCTATGATTTTTGAAAAACAG
NC_000001.11:33 ------------------------------------------------------------
NC_000023.11:15 ------------------------------------------------------------
NC_000004.12:c1 ------------------------------------------------------------

NC_000013.11:c3 CATAAGAAATAATTTTTTAATTTGTTATATAAGTAAATGAACTTTAGCATCTTAAGGATT
NC_000001.11:33 ------------------------------------------------------------
NC_000023.11:15 ------------------------------------------------------------
NC_000004.12:c1 ------------------------------------------------------------

NC_000013.11:c3 CAATGATACAGTTTTTAGATTTATTTTTTAAATTGACAAATACCAATTGTACATATTCTT
NC_000001.11:33 ------------------------------------------------------------
NC_000023.11:15 ------------------------------------------------------------
NC_000004.12:c1 ------------------------------------------------------------

NC_000013.11:c3 GAGAGCACATAATTATATTTCCATACATATAATTTATAGTTATCAGATCAGGGTAATTCG
NC_000001.11:33 ------------------------------------------------------------
NC_000023.11:15 ------------------------------------------------------------
NC_000004.12:c1 ------------------------------------------------------------

NC_000013.11:c3 CACATCCACCATCTCATATGTTTATCATTTCTTTATGTTGAGAATGTGCAATATCCTTCC
NC_000001.11:33 ------------------------------------------------------------
NC_000023.11:15 ------------------------------------------------------------
NC_000004.12:c1 ------------------------------------------------------------

NC_000013.11:c3 AGCTATTTGAAACTATATATTGTATTATTATTAACTATAGTCATTCTACAGTGCTGTCGA
NC_000001.11:33 ------------------------------------------------------------
NC_000023.11:15 ------------------------------------------------------------
NC_000004.12:c1 ------------------------------------------------------------
```

```
NC_000013.11:c3 ACACTAGAACTTATTTCTCTTATCTCGCTATAATTTTGTATCTTTTAACAAATCTCTCTG
NC_000001.11:33 ------------------------------------------------------------
NC_000023.11:15 ------------------------------------------------------------
NC_000004.12:c1 ------------------------------------------------------------

NC_000013.11:c3 TCACTCTCGTCCCAGCCTCTAGTATCCTCTGTCCTACTTTTTACTTCTATGAGATCAACT
NC_000001.11:33 ------------------------------------------------------------
NC_000023.11:15 ------------------------------------------------------------
NC_000004.12:c1 ------------------------------------------------------------

NC_000013.11:c3 TTTTTTAGCTTTCGCATATGAGTGAGAACATGCACTGTTTAACTTTCTGTTCCTGGCTTT
NC_000001.11:33 ------------------------------------------------------------
NC_000023.11:15 ------------------------------------------------------------
NC_000004.12:c1 ------------------------------------------------------------

NC_000013.11:c3 TTTCACTTAAAATAATGTCCTCCAGTTTCATCTATATTCCATCAACGATGGGATTTCACT
NC_000001.11:33 ------------------------------------------------------------
NC_000023.11:15 ------------------------------------------------------------
NC_000004.12:c1 ------------------------------------------------------------

NC_000013.11:c3 CTTTTTTATGGCTGAATAATACTCCATAGTGTGTATATATCACATTTTCTTCATTCATTC
NC_000001.11:33 ------------------------------------------------------------
NC_000023.11:15 ------------------------------------------------------------
NC_000004.12:c1 ------------------------------------------------------------

NC_000013.11:c3 ATCTGTTGTTGGACACCTAGGTTGACTCCATATCTTGGCTATTGAGAATAGTGCTGCAGT
NC_000001.11:33 ------------------------------------------------------------
NC_000023.11:15 ------------------------------------------------------------
NC_000004.12:c1 ------------------------------------------------------------

NC_000013.11:c3 AGACATGGGATGCAGATATCTCTTCGTTATACTATCAATGACTTGTTTTGTTTTCTTTTT
NC_000001.11:33 ------------------------------------------------------------
NC_000023.11:15 ------------------------------------------------------------
NC_000004.12:c1 ------------------------------------------------------------

NC_000013.11:c3 TGAAACAGAGTTTCTCTCTTGTTGCCCAGGCTAGAGTGCAGTGGCAGGATCTCGGCTCAC
NC_000001.11:33 ------------------------------------------------------------
NC_000023.11:15 ------------------------------------------------------------
NC_000004.12:c1 ------------------------------------------------------------

NC_000013.11:c3 TGCAACCTCCACCTCTCGGGCTCAAGCAATTCTCCTGCCTCAGCTCTCGAGTAGCTGGGA
NC_000001.11:33 ------------------------------------------------------------
NC_000023.11:15 -----------------------AAGCAATTCAGGTGTTTCAACTT--------------
NC_000004.12:c1 ------------------------------------------------------------

NC_000013.11:c3 TTACAGTTGCTCACCACCACACCTGGCTAATTTTTGTGTTTTTAGTAGAGACAAGGTTTC
NC_000001.11:33 ------------------------------------------------------------
NC_000023.11:15 --------------------------------------------------------TTCC
NC_000004.12:c1 ------------------------------------------------------------

NC_000013.11:c3 ACTGTGTTGGTCAGGCTGGTCTCAAACTCCCAGTCTCAGGTGATCTGCCCATCTTGGCCT
NC_000001.11:33 ------------------------------------------------------------
NC_000023.11:15 AACGCGTT-----------CCCCGAGCTCCCC----------------------------
```

```
NC_000004.12:c1 ------------------------------------------------------------


NC_000013.11:c3 CCCAAAGTGTTGGGATTACAGGCGTGAGCCACCACGCCCAGCCTCAATGACTTGTTTCAA
NC_000001.11:33 ------------------------------------------------------------
NC_000023.11:15 ------------------------------------------------------------
NC_000004.12:c1 ------------------------------------------------------------


NC_000013.11:c3 TGAATTCTCTCTTTGAAGACATACTGGTATCTCCTGCTTATTGACAATTGTGGCAGCAGC
NC_000001.11:33 ----------------------------------------------------------AT
NC_000023.11:15 ----------------------------------------------------------GC
NC_000004.12:c1 ------------------------------------------------------------


NC_000013.11:c3 TTCCAAAAGCTGATTTTTCTGACATCTTTGAAACTTTTGGACACTTCTCCCATTGGGAAA
NC_000001.11:33 CTCCAGAAGCTCATTACTCT----------------------------------------
NC_000023.11:15 TTTCGGGGGTCGGCCCCCTC----------------------------------------
NC_000004.12:c1 ------------------------------------------------------------


NC_000013.11:c3 ATAGAATTTTTCTTTTGAATAATTCTCATTTCCCACTAAGCATATAAGCAACCTGGAGTA
NC_000001.11:33 ------------------------------------------------------------
NC_000023.11:15 ------------------------------------------------------------
NC_000004.12:c1 ------------------------------------------------------------


NC_000013.11:c3 TCTTGTAAGACCCATACAGATTAATATTTCAAGCATAATAATCCTTATACATTCCTTTTT
NC_000001.11:33 ------------------------------------------------------------
NC_000023.11:15 ------------------------------------------------------------
NC_000004.12:c1 ------------------------------------------------------------


NC_000013.11:c3 GACGATGGAGACTATGGTTACGAGTAGCAGTTTTTAAATAAGGACTACTTTTGAGGTCCC
NC_000001.11:33 ------------------------------------------------------------
NC_000023.11:15 ------------------------------------------------------------
NC_000004.12:c1 ------------------------------------------------------------


NC_000013.11:c3 AAAGCTTTGAAAACCTGAGGTATAAACAAAACAACATATTCCAATGAGATATTGATATCT
NC_000001.11:33 ------------------------------------------------------------
NC_000023.11:15 ------------------------------------------------------------
NC_000004.12:c1 ------------------------------------------------------------


NC_000013.11:c3 AAACTAGTTTTCTTAAATCTGTCATTCAAAGGTTGAGAATTTCTTCCTGAAATTTAGACA
NC_000001.11:33 ------------------------------------------------------------
NC_000023.11:15 ------------------------------------------------------------
NC_000004.12:c1 ------------------------------------------------------------


NC_000013.11:c3 ATATATTATGGAAATGCGGTTTGTCTTTTTGTAAATTTAGGATGATTCTTTTTAAAAATA
NC_000001.11:33 ------------------------------------------------------------
NC_000023.11:15 ------------------------------------------------------------
NC_000004.12:c1 ------------------------------------------------------------


NC_000013.11:c3 ATTATAGGCATTGTTTATTCTGTATATTAGTGTAATAGTCAATGCAGTAGCTAGAATTGT
NC_000001.11:33 ------------------------------------------------------------
NC_000023.11:15 ------------------------------------------------------------
NC_000004.12:c1 ------------------------------------------------------------


NC_000013.11:c3 GGTTGCTAATAGTCACAAAATCTAGTAAAATTGTGAGTACATGAACCCATCCCAGGAAAC
```

```
NC_000001.11:33 ------------------------------------------------------------
NC_000023.11:15 ------------------------------------------------------------
NC_000004.12:c1 ------------------------------------------------------------


NC_000013.11:c3 TGATGGGACAAGTAGTAAATTTTTTTGACTCTTTAACAAAAATATTTAATGCTGCCAAAA
NC_000001.11:33 ------------------------------------------------------------
NC_000023.11:15 ------------------------------------------------------------
NC_000004.12:c1 ------------------------------------------------------------


NC_000013.11:c3 AGTATAAAAATACAGTAGGAATGGCAGTACAACACAAATTAATCTCTCCTAATTTATTTC
NC_000001.11:33 ------------------------------------------------------------
NC_000023.11:15 ------------------------------------------------------------
NC_000004.12:c1 ------------------------------------------------------------


NC_000013.11:c3 TTTTACATATTTCTACGTTTCATACACCCATTAAAAACACTTAACAGGCCAATGCGGTGG
NC_000001.11:33 ------------------------------------------------------------
NC_000023.11:15 ------------------------------------------------------------
NC_000004.12:c1 ------------------------------------------------------------


NC_000013.11:c3 CTCACGCCTGTAATCCCAGCATTTTGGGAGGCCGAGGCGGGCGGATCACGAGGTCAGGAG
NC_000001.11:33 ------------------------------------------------------------
NC_000023.11:15 ------------------------------------------------------------
NC_000004.12:c1 ------------------------------------------------------------


NC_000013.11:c3 ATCGAGACCATCCTGGCGAACACGGTGAAACCCCATCTCTACTAAAAATACAAAAAATTA
NC_000001.11:33 -----------------------------------TCTCTATTA----------------
NC_000023.11:15 ------------------------------------------------------------
NC_000004.12:c1 ------------------------------------------------------------


NC_000013.11:c3 GCCAGGCTTGGTGGCGGGCGCCTGTATTCCCAGCTACTCCGGAGGCTGAGGCAGGAGAAT
NC_000001.11:33 ------------AACTGATGCCTACAT---------------------------------
NC_000023.11:15 ------------GGCGGACGCCCGC-----------------------------------
NC_000004.12:c1 --------------AGGGCGCTTGC-----------------------------------
                                *..**....

NC_000013.11:c3 GGCGTGAACCCAGGAGGCGGAGCTTGCAGTGAGCCGAGATCGCGCCACTGTACTCCAGCC
NC_000001.11:33 ------------------------------------------------------------
NC_000023.11:15 ------------------------------------------------------------
NC_000004.12:c1 ------------------------------------------------------------


NC_000013.11:c3 TGGGCAACAGAGCAGAGACTCCGTCTCAAAAAAAAAAAAAAAAAAAAAAAAACACAACGA
NC_000001.11:33 ------------------------------------------------------------
NC_000023.11:15 ------------------------------------------------------------
NC_000004.12:c1 ------------------------------------------------------------


NC_000013.11:c3 CACATCCAATGTAAGGTTCTGCAAAGTCTTCTGCTGGTGGGTGCTCCTCATACCCTGTAT
NC_000001.11:33 ------------------------------------------------------------
NC_000023.11:15 ------------------------------------------------------------
NC_000004.12:c1 ------------------------------------------------------------


NC_000013.11:c3 GTAAAGTTTACTTTGTAAACAAACAACTGTGAGGCAATCTAGAAGGTTAGCAAGCCTCAC
NC_000001.11:33 ------------------------------------------------------------
NC_000023.11:15 ------------------------------------------------------------
NC_000004.12:c1 ------------------------------------------------------------
```

```
NC_000013.11:c3 TTTAGTTTCTGGAGTGGGCTTCAGGTCTTGTTTTGCACATCAGTGGTTCAAAATTTATAG
NC_000001.11:33 ------------------------------------------------------------
NC_000023.11:15 ------------------------------------------------------------
NC_000004.12:c1 ------------------------------------------------------------

NC_000013.11:c3 CTCCAGAATATTCTCAAGTCATGAATATTAGGTGTCTATCTTGGCCTTTTTCTTTTTTGT
NC_000001.11:33 ------------------------------------------------------------
NC_000023.11:15 ------------------------------------------------------------
NC_000004.12:c1 ------------------------------------------------------------

NC_000013.11:c3 TCGTTCTCATTTAGCCTCTGTTTCATCTGCTGCACCTTCTGAGCTGTGGTGGCCTTTCTG
NC_000001.11:33 ------------------------------------------------------------
NC_000023.11:15 -------------------------------------------CCGTGGCGGCCCC----
NC_000004.12:c1 ------------------------------------------------------------

NC_000013.11:c3 TCCTCTAGAATATGTTTCCGTAGAGCATTCTCACTGTTCTCTTCCATTGTATCTTGCATT
NC_000001.11:33 ------------------------------------------------------------
NC_000023.11:15 ------------------------------------------------------------
NC_000004.12:c1 ------------------------------------------------------------

NC_000013.11:c3 TACTGTACATCGGTCACCTGTCAGTTTTTCTTCTTCTAGACCAATTCTGTTCCCATTGTA
NC_000001.11:33 ------------------------------------------------------------
NC_000023.11:15 ------------------------------------------------------------
NC_000004.12:c1 ------------------------------------------------------------

NC_000013.11:c3 TTTTTCAGTCATTATTTTGTTCGGTGAATGATCAAAAGCAGGAGCTAAGTTTTCCTTAGA
NC_000001.11:33 ----------------------GGTGAAAGGCTAAAACCATGGTA---------------
NC_000023.11:15 --------------------------AGGGGCCGCAGCCGGGAAC---------------
NC_000004.12:c1 ------------------------------------------------------------

NC_000013.11:c3 AAATTCTTTCCACCGTTTCTGTATACTAGAATCTACCTTTATACATTTTGCTCCTTCTAT
NC_000001.11:33 ------------------------------------------------------------
NC_000023.11:15 ------------------------------------------------------------
NC_000004.12:c1 ------------------------------------------------------------

NC_000013.11:c3 TATAGCCATGTATGAGAATCTCAGTTGATCTGGGGTCTGAATAAGACCCATTCAGTATTT
NC_000001.11:33 ------------------------------------------------------------
NC_000023.11:15 ------------------------------------------------------------
NC_000004.12:c1 ------------------------------------------------------------

NC_000013.11:c3 TCTCATGTTCAATAACACTTGTTTAATGTTAATATAATCTCCTTTTCCCATCAAAACAAG
NC_000001.11:33 ------------------------------------------------------------
NC_000023.11:15 ------------------------------------------------------------
NC_000004.12:c1 ------------------------------------------------------------

NC_000013.11:c3 ACAAGCGTCTACCAGAGAGAAGGTGCCCGTGCACCCAGTGCCTTCGCTACAATGGATCAC
NC_000001.11:33 ------------------------------------------------------------
NC_000023.11:15 ------------------------------------------------------------
NC_000004.12:c1 ------------------------------------------------------------

NC_000013.11:c3 TGCAGGCCCATGGTCAGGGTTCAAGGAGCCAGATTCTCTCACTTTCAACAAGAAATTGAG
NC_000001.11:33 -------CTTTGGTCAGGGTCCCCGCA---------------------------------
NC_000023.11:15 -------------CCAGGGCCCCCGAGGCCAGATGTTT----------------------
```

```
NC_000004.12:c1 ------------------------------------------------------------

NC_000013.11:c3 AAATGAAGCTGGTGATTCAGGGACTCCAAAATCTGGCCAGGTCGTATAATGCAAGTGAGA
NC_000001.11:33 ------------------------------------------------------------
NC_000023.11:15 ------------------------------------------------------------
NC_000004.12:c1 ------------------------------------------------------------

NC_000013.11:c3 TATTGTTCTGGTTTCACCACTATTGATATTTTCTAATCGTAGTAGATGTACTGTGTAATA
NC_000001.11:33 ------------------------------------------------------------
NC_000023.11:15 ------------------------------------------------------------
NC_000004.12:c1 ------------------------------------------------------------

NC_000013.11:c3 TGACTTCACATCTTCTGAAAGAGCTTCACATTGAATCCCGTTTCTTTAAACAGCATCTTT
NC_000001.11:33 ------------------------------------------------------------
NC_000023.11:15 ------------------------------------------------------------
NC_000004.12:c1 ------------------------------------------------------------

NC_000013.11:c3 TGGTCATCTGTTGGCCAGTACTGCGCACATTTAACCGATTCTTTCTCCACAATGCGGTTC
NC_000001.11:33 ------------------------------------------------------------
NC_000023.11:15 ------------------------------------------------------------
NC_000004.12:c1 ------------------------------------------------------------

NC_000013.11:c3 AGCACAACAACTGCTTTGGTCTTCTGCTGCCAAACCGTAAGCCAGAAATGGCAGCACATG
NC_000001.11:33 ------------------------------------------------------------
NC_000023.11:15 ------------------------------------------------------------
NC_000004.12:c1 ------------------------------------------------------------

NC_000013.11:c3 TTAGGAAGTGGACCCGGTGTTAAGATGTAACTCCTTCGTGCCTCTTCTATGTCAACTAAG
NC_000001.11:33 ------------------------------------------------------------
NC_000023.11:15 ------------------------------------------------------------
NC_000004.12:c1 ------------------------------------------------------------

NC_000013.11:c3 CTGGCATTAATATAATCATTCTCAGTATTCTGCAGTTTAACACGACTGTGATCATATGGG
NC_000001.11:33 ------------------------------------------------------------
NC_000023.11:15 ------------------------------------------------------------
NC_000004.12:c1 ------------------------------------------------------------

NC_000013.11:c3 CTTACATCTCTGTATCTATTTCGATTTCTGTTTTCTGGAAACTTGGCCACTCTATGAAGA
NC_000001.11:33 ------------------------------------------------------------
NC_000023.11:15 ------------------------------------------------------------
NC_000004.12:c1 ------------------------------------------------------------

NC_000013.11:c3 TAGTCATGGGACTCATTTCGAATTTCCAAGTACGGCAGCTGCCAGCGATGCTGAGCATCC
NC_000001.11:33 ------------------------------------------------------------
NC_000023.11:15 --------------------------------GGGCAGCTGC------------------
NC_000004.12:c1 ------------------------------------------------------------

NC_000013.11:c3 AACTCCTTGAACTCCCGCTCAATGATGGTGGGCATGGCTGGGACAAATAGTGAATCTTAA
NC_000001.11:33 ------------------------------------------------------------
NC_000023.11:15 ------------------------------------------------------------
NC_000004.12:c1 ------------------------------------------------------------

NC_000013.11:c3 GAGAAATGGAAGAGTGAGGTTGGCTCCTAAGCTGGACAGACACTCAATGTGGTTTATTAT
```

```
NC_000001.11:33 ------------------------------------------------------------
NC_000023.11:15 ------------------------------------------------------------
NC_000004.12:c1 ------------------------------------------------------------


NC_000013.11:c3 AGTCAATCATTACTCAAGGAAGTTGTATATATTCTATTCATATATATATTTGGTAAATTT
NC_000001.11:33 ------------------------------------------------------------
NC_000023.11:15 ------------------------------------------------------------
NC_000004.12:c1 ------------------------------------------------------------


NC_000013.11:c3 CTAATTACCATACCCCTATGAAGTCATTACCATACTCCTATAAAGTATTATCTTCATTAT
NC_000001.11:33 ------------------------------------------------------------
NC_000023.11:15 ------------------------------------------------------------
NC_000004.12:c1 ------------------------------------------------------------


NC_000013.11:c3 AAAGTGAAGAGAGTGAGGCTGAGAGAAGTTAAATTATGTGCCCAAGGTCATGGCTAGTGA
NC_000001.11:33 ------------------------------------------------------------
NC_000023.11:15 ---------------------------------------------GGCTGCGGCGGCTGG
NC_000004.12:c1 ------------------------------------------------------------


NC_000013.11:c3 ATGAGCAAACTTGGTTTCAAAGTCAGGTCTGTCTGGCTCCCAAGCCACCAGTCTTTTACG
NC_000001.11:33 ------------------------------------------------------------
NC_000023.11:15 GCGAGCAGAGG-------------------------------------------------
NC_000004.12:c1 ------------------------------------------------------------


NC_000013.11:c3 TTACCACATTGTCTTAGTCTGGGCTCTCCCGAAAGCAAAGCCTGTACAAACAACTTGGAG
NC_000001.11:33 ------------------------------------------------------------
NC_000023.11:15 ------------------------------------------------------------
NC_000004.12:c1 ------------------------------------------------------------


NC_000013.11:c3 GTAGGTAGGGAGTTTATTTGGGAATGTGACACCAAGGAATAGACTGTGATAAGACAGAAG
NC_000001.11:33 ------------------------------------------------------------
NC_000023.11:15 ------------------------------------------------------------
NC_000004.12:c1 ------------------------------------------------------------


NC_000013.11:c3 AATGACCCCCCGGAAGGAGAGAAAGCCAACCCAAGGGTCCCTTTCAAATTGGCAACCATT
NC_000001.11:33 ------------------------------------------------------------
NC_000023.11:15 ------------------------------------------------------------
NC_000004.12:c1 ------------------------------------------------------------


NC_000013.11:c3 ATGAATATCTGCTACTTGATCCTGTGGGAGCTTCTGAGGAACCTTTTAACATGCATCTCA
NC_000001.11:33 ------------------------------------------------------------
NC_000023.11:15 ------------------------------------------------------------
NC_000004.12:c1 ------------------------------------------------------------


NC_000013.11:c3 GAATTGGCCACCCTGGGCTAAAAATGAGACAAGCATTTTTTCTATGAGCTCCTGTCCCTC
NC_000001.11:33 ------------------------------------------------------------
NC_000023.11:15 ------------------------------------------------------------
NC_000004.12:c1 ------------------------------------------------------------


NC_000013.11:c3 AGTGGTCAGGGCAAGCTTACAGGCCTTAACTGTCCTGCATTTCCAGATTGTCCATATCTG
NC_000001.11:33 ------------------------------------------------------------
NC_000023.11:15 ------------------------------------------------------------
NC_000004.12:c1 ------------------------------------------------------------
```

```
NC_000013.11:c3 AGTGCCAAGTGGTTCCCATGGGACTCCTGTGTCACAGCTCCAATGTAGCCCTGGGGCCAG
NC_000001.11:33 ------------------------------------------------------------
NC_000023.11:15 ------------------------------------------------------------
NC_000004.12:c1 ------------------------------------------------------------


NC_000013.11:c3 AAACTAGGGGCAGAGGCCAAAAGCAGGTGCTGTGAGGTTGCATCCGCACAAGGTAGAAGA
NC_000001.11:33 ------------------------------------------------------------
NC_000023.11:15 ------------------------------------------------------------
NC_000004.12:c1 ------------------------------------------------------------


NC_000013.11:c3 AGCCCACAGAGACCTGGGTACCATAGCAGTGTTTGAAATAAGAGGTGAAATTGAGAACAC
NC_000001.11:33 ------------------------------------------------------------
NC_000023.11:15 ------------------------------------------------------------
NC_000004.12:c1 ------------------------------------------------------------


NC_000013.11:c3 GTGATATATTGCTCAGAATGTTTAGAGACACATGACCAGTTACAATGAGAGAGTGGAATG
NC_000001.11:33 -----------------------GGAAACAGATGGCC-----------------------
NC_000023.11:15 -----------------------GGCGGCTGCGGGCT-----------------------
NC_000004.12:c1 -----------------------GGAGTCACCGGACT-----------------------
                                       .* . *    *.*.

NC_000013.11:c3 AGAATCTTCAATGTCTCATCATGCATTCATTCATTCATTTATCCATTCAGTCACCAACGA
NC_000001.11:33 ------------------------------------------------------------
NC_000023.11:15 ------------------------------------------------------------
NC_000004.12:c1 ------------------------------------------------------------


NC_000013.11:c3 ATGCTGGGTAGTATATGAAGTGATAGAAGAATTAAAATGGGTCTAGCTGAGTCTCAGATA
NC_000001.11:33 ------------------------------------------------------------
NC_000023.11:15 ------------------------------------------------------------
NC_000004.12:c1 ------------------------------------------------------------


NC_000013.11:c3 CGACAGAAAAGAAAATAGACAATATGATAGTGTGTGCTAAGTGCTTCAATAGAAGTATAC
NC_000001.11:33 ------------------------------------------------------------
NC_000023.11:15 ------------------------------------------------------------
NC_000004.12:c1 ------------------------------------------------------------


NC_000013.11:c3 AAAAAGGTAGTAAAAGTATCGAAGAGAGATACCCAGTGTATTCTGTGTGTGGACAGAGAG
NC_000001.11:33 ------------------------------------------------------------
NC_000023.11:15 ------------------------------------------------------------
NC_000004.12:c1 ------------------------------------------------------------


NC_000013.11:c3 GGGGTGTGGGGAGAAGGGAGGTCATTTTCCTAGAGCTTAGTCTTGAAAGATAAATAGAAG
NC_000001.11:33 ------------------------------------------------------------
NC_000023.11:15 ------------------------------------------------------------
NC_000004.12:c1 ------------------------------------------------------------


NC_000013.11:c3 TCAGCCAACTGAGGAGAAATATGAGCATCAACTCTATATTCAAATTAAATTTTAGTATTT
NC_000001.11:33 -------------------------------------ACTCAAATTAA------------
NC_000023.11:15 ------------------------------------------------------------
NC_000004.12:c1 ------------------------------------------------------------


NC_000013.11:c3 CAATATTAAATTTCCTAAGTATGATCCTTGTGGTTATGTAAGAGAGTGTCCTATTTTTAG
NC_000001.11:33 ------------------------------------------------------------
NC_000023.11:15 ------------------------------------------------------------
```

```
NC_000004.12:c1 ------------------------------------------------------------

NC_000013.11:c3 GAGATACTTGCTGAAAAATTTAAGAGTAAAGTGTCATAATGTCTGCAACTTACTCTCAAA
NC_000001.11:33 ------------------------------------------------------------
NC_000023.11:15 ------------------------------------------------------------
NC_000004.12:c1 ------------------------------------------------------------

NC_000013.11:c3 TGATTCAGCAAAGTAAATATTTGGGGATGGGGAAAAGCAAGTGAACACAAATGTAGCAAA
NC_000001.11:33 ------------------------------------------------------------
NC_000023.11:15 ------------------------------------------------------------
NC_000004.12:c1 ------------------------------------------------------------

NC_000013.11:c3 ATGTTAATAACTGGTGAATATAACTAAAGGATATGTGTGTGTTTATTGCACTAATCTTAC
NC_000001.11:33 -----AATGATTGGTGA-------------------------------------------
NC_000023.11:15 --GTGGGCGCTGGGCGG-------------------------------------------
NC_000004.12:c1 ------------------------------------------------------------

NC_000013.11:c3 AACTTTTTTAAATTTTCTGAAATAAAAAAAGGAGAGGAGAAAATTAATTGAGGGCCATGG
NC_000001.11:33 ----------------------------------------------------------GG
NC_000023.11:15 ---------------------------------------------------CGGCAGCGG
NC_000004.12:c1 ---------------------------------------------------CGGTGAAGG
                                                                          **

NC_000013.11:c3 GCAGGGGTTGGGGAGGAGCAGTCTGGGATGGGCTTGTGAGAGGGACACCAATACCTGAGG
NC_000001.11:33 GCAGAGTGTGGGAA----------------------------------------------
NC_000023.11:15 GAAGGGGGCCGGGA----------------------------------------------
NC_000004.12:c1 GCGGGCGGCGGCGT----------------------------------------------
                * .*.   . * .

NC_000013.11:c3 ACGCTTGGGGCATTTTTAAAATTGCAGCGATTGGCCAGGTGCAGTGGCTCACACCTGGAA
NC_000001.11:33 ------------------------------------------------------------
NC_000023.11:15 ------------------------------------------------------------
NC_000004.12:c1 ------------------------------------------------------------

NC_000013.11:c3 TCCCAGCACTTTGGAGGCCAAGGCAGGAGGATCGTTTGAGACCAGTCTGGGCAACAGAGC
NC_000001.11:33 ------------------------------------------------------------
NC_000023.11:15 ------------------------------------------------------------
NC_000004.12:c1 ------------------------------------------------------------

NC_000013.11:c3 AAGACCCCGTCTCTACAGAAAATACAATATTAAATAGCCAGGCATGGTGGCACATGGCTG
NC_000001.11:33 ------------------------------------------------------------
NC_000023.11:15 ------------------------------------------------------------
NC_000004.12:c1 ------------------------------------------------------------

NC_000013.11:c3 TGGTCCCAGCTACTCAGGAGGCTGACGTGGGAGGATTGCTTAAGCCCAGGAGTTTGAGGC
NC_000001.11:33 ------------------------------------------------------------
NC_000023.11:15 ------------------------------------------------------------
NC_000004.12:c1 ------------------------------------------------------------

NC_000013.11:c3 TGCAATGAACTATGATCGCACCACTGCACTCCAGCCTGGGCAACAGAGCAAGACCCTGTC
NC_000001.11:33 ------------------------------------------------------------
NC_000023.11:15 ------------------------------------------------------------
NC_000004.12:c1 ------------------------------------------------------------

NC_000013.11:c3 TCTAAGAAAAGTAAATAAATTGCAGTGGTTAGTGGGACACCATGGGACATAAATGCTCAT
```

```
NC_000001.11:33 ------------------------------------------------------------
NC_000023.11:15 ------------------------------------------------------------
NC_000004.12:c1 ------------------------------------------------------------


NC_000013.11:c3 GATAGAGAAGCAACAAAAATGAGACAAGAAAAGGGAGTGATCTGTGCTAGAGTGTTGGGC
NC_000001.11:33 ------------------------------------------------------------
NC_000023.11:15 ------------------------------------------------------------
NC_000004.12:c1 ------------------------------------------------------------


NC_000013.11:c3 TGCATCCTAGAGGCCTGGGTGACATTGAATAATTTCATGCATCTTTTGTGTTTAAATGGT
NC_000001.11:33 ------------------------------------------------------------
NC_000023.11:15 ------------------------------------------------------------
NC_000004.12:c1 ------------------------------------------------------------


NC_000013.11:c3 CACTTTAGCAGCAGTTTAATGAGAATGGATTAGAAAAGGCCAAAACTGTGGCAAGAAGTT
NC_000001.11:33 ------------------------------------------------------------
NC_000023.11:15 ------------------------------------------------------------
NC_000004.12:c1 ------------------------------------------------------------


NC_000013.11:c3 TAGTTTTTATGTTATGGTATTTAAGGTTACAGGTTCTATAGGCCTACCGGAAAATGGTGC
NC_000001.11:33 ------------------------------------------------------------
NC_000023.11:15 ------------------------------------------------------------
NC_000004.12:c1 ------------------------------------------------------------


NC_000013.11:c3 CAAGATAAAGTGAAGACAGCTTTGAAACTGCAGAGGGCAGAAGAGTCAACAGGACTTGGC
NC_000001.11:33 ------------------------------------------------------------
NC_000023.11:15 ------------------------------------------------------------
NC_000004.12:c1 ------------------------------------------------------------


NC_000013.11:c3 AATCCCCTATACTCAGTGAGTTCCTAAGATGGACTTGGTTCAGTTCAGTGATGAGCCTTT
NC_000001.11:33 ------------------------------------------------------------
NC_000023.11:15 ------------------------------------------------------------
NC_000004.12:c1 ------------------------------------------------------------


NC_000013.11:c3 TAAACCCCAAAGAGAAGGAAGACTTGCGGAGGAAAAAAATTAGTTTTAGAATGTATTTAA
NC_000001.11:33 ------------------------------------------------------------
NC_000023.11:15 ------------------------------------------------------------
NC_000004.12:c1 ------------------------------------------------------------


NC_000013.11:c3 CCATGAAATAATGAATTAAGGTGCTTAGTTTATACATCAGGGGTTTATTTATCTTTTATC
NC_000001.11:33 ------------------------------------------------------------
NC_000023.11:15 ------------------------------------------------------------
NC_000004.12:c1 ------------------------------------------------------------


NC_000013.11:c3 ACCATAACTAGTACTGCTTGCATTATAGTAGGTATTCGATAAAATTTTTTAAACTGTGTT
NC_000001.11:33 ------------------------------------------------------------
NC_000023.11:15 ------------------------------------------------------------
NC_000004.12:c1 ------------------------------------------------------------


NC_000013.11:c3 TAGAAATCATGATAGTAGATGGTAAAATGCTTTCTTCAGTTGCTGATACTCGGGGGAAAT
NC_000001.11:33 ------------------------------------------------------------
NC_000023.11:15 ------------------------------------------------------------
NC_000004.12:c1 ------------------------------------------------------------
```

```
NC_000013.11:c3 ATGTTGTATATTTTAATGTTTTCTCCCTGAGGGCTAAGCATTTGGTGAACATCTGTCTCC
NC_000001.11:33 ------------------------------------------------------------
NC_000023.11:15 ------------------------------------------------------------
NC_000004.12:c1 ------------------------------------------------------------

NC_000013.11:c3 TAACACCTGTTGGGCTGGTTTACCTGGCAATCTAAAGTGAACATGTGCCAGGGTAGTGCC
NC_000001.11:33 ------------------------------------------------------------
NC_000023.11:15 ------------------------------------------------------------
NC_000004.12:c1 ------------------------------------------------------------

NC_000013.11:c3 ACTTAAAGTACCTTCACTTTTTAGAATCTATCAGATGTGTTTAGAATCAGTTTTGTCTTC
NC_000001.11:33 ------------------------------------------------------------
NC_000023.11:15 ------------------------------------------------------------
NC_000004.12:c1 ------------------------------------------------------------

NC_000013.11:c3 CACCAGCTAGTTTTCTCAGTTTAGAAAGTACATGTATTTCTACTTCTTAGTAAATGTAAT
NC_000001.11:33 ------------------------------------------------------------
NC_000023.11:15 ------------------------------------------------------------
NC_000004.12:c1 ------------------------------------------------------------

NC_000013.11:c3 ATATGACACATGAATTTAAAGAACGTACAAAGTAGGAAACATTTATATTTCATACAACTT
NC_000001.11:33 ------------------------------------------------------------
NC_000023.11:15 ------------------------------------------------------------
NC_000004.12:c1 ------------------------------------------------------------

NC_000013.11:c3 GAATTTTCAAATGCAAAAATTTCCTCTTGGGAGTGAGTCTTATGTTTTGGAGCAGGAATG
NC_000001.11:33 ------------------------------------------------------------
NC_000023.11:15 ------------------------------------------------------------
NC_000004.12:c1 ------------------------------------------------------------

NC_000013.11:c3 ACAGAACAGATATGGTACCAAAGCCAAGCTCACCTAGATATTGCAGACACACAGGCAAAA
NC_000001.11:33 ------------------------------------------------------------
NC_000023.11:15 ------------------------------------------------------------
NC_000004.12:c1 ------------------------------------------------------------

NC_000013.11:c3 GAGGAGGTGGCCCGAGTGTGTCATCCGAGGGTCCCTCGGCATGGGAGCAGTTCCTCTGGT
NC_000001.11:33 ------------------------------------------------------------
NC_000023.11:15 ------------------------------GTTCCCCGGCCCGCGACGGACTCCGCTGGG
NC_000004.12:c1 ------------------------------------------------------------

NC_000013.11:c3 GGGTAGTGAACTGAGCAAAGAGGCAAATGAATGATAGGTAGTTACACAGAGAGAATCCCT
NC_000001.11:33 ------------------------------------------------------------
NC_000023.11:15 GAACGG------------------------------------------------------
NC_000004.12:c1 ------------------------------------------------------------

NC_000013.11:c3 ACATTAAGGAAGTACGACAAACACGAGGACTCGATTTAGATTCTATGTCTAAGAGCCTTC
NC_000001.11:33 ------------------------------------------------------------
NC_000023.11:15 ------------------------------------------------------------
NC_000004.12:c1 ------------------------------------------------------------

NC_000013.11:c3 CTTTGACATCCCTCCCCACCATGTCAGGGAGGATATGTACTCTCATTCCATTCAGTCGTT
NC_000001.11:33 ------------------------------------------------------------
NC_000023.11:15 ------------------------------------------------------------
```

```
NC_000004.12:c1 ------------------------------------------------------------

NC_000013.11:c3 AGCACTGCTCACACTGGTAATAGCTTCTCGTTGTCTGCTTTCCTCTTTGGACTTTAGATA
NC_000001.11:33 ------------------------------------------------------------
NC_000023.11:15 ------------------------------------------------------------
NC_000004.12:c1 ------------------------------------------------------------

NC_000013.11:c3 GCCCCTTGAAAGCCACCCACCTTTGTGTTCCCAGTGTTTAGATTGGTCCCTAGCACAATG
NC_000001.11:33 ------------------------------------------------------------
NC_000023.11:15 ------------------------------------------------------------
NC_000004.12:c1 ------------------------------------------------------------

NC_000013.11:c3 TCAGTGTTCTGTAACTTTTACATACATGAATGAACAAATGGACAAATGAACATACATGAC
NC_000001.11:33 ------------------------------------------------------------
NC_000023.11:15 ------------------------------------------------------------
NC_000004.12:c1 ------------------------------------------------------------

NC_000013.11:c3 TTCCTAAAAGGATGTCATAGCTCCTGATAGTCATGACAGCACTAGTATTAACCTTTGTTT
NC_000001.11:33 ------------------------------------------------------------
NC_000023.11:15 ------------------------------------------------------------
NC_000004.12:c1 ------------------------------------------------------------

NC_000013.11:c3 TGTAATGAGTTTCAATTTAATGTTAAGGAAAATATAGGATTTTAAAAACTAGTACACACA
NC_000001.11:33 ------------------------------------------------------------
NC_000023.11:15 ------------------------------------------------------------
NC_000004.12:c1 ------------------------------------------------------------

NC_000013.11:c3 CATACACATATTCCAGAGTCTTTCTAGTGAGTTTGAATATTAATTTATAAAAGAATCTAA
NC_000001.11:33 ------------------------------------------------------------
NC_000023.11:15 ------------------------------------------------------------
NC_000004.12:c1 ------------------------------------------------------------

NC_000013.11:c3 CATCACATGCTTTAAAAAATAGTGTTTGTGGCCAGGTGTGGTGGCTCATGCCTGTAACCC
NC_000001.11:33 ------------------------------------------------------------
NC_000023.11:15 ------------------------------------------------------------
NC_000004.12:c1 ------------------------------------------------------------

NC_000013.11:c3 CAGCACTTTGGGAGGCTGAGGCAGGCGGATTACTTGGGGTCAGGAGTTCCAGACCAGCCT
NC_000001.11:33 ------------------------------------------------------------
NC_000023.11:15 ------------------------------------------------------------
NC_000004.12:c1 ------------------------------------------------------------

NC_000013.11:c3 GGCCAACATGGCAAAACCCCGTCTCTACTAAAAATACAAAAATTAGCCAGGCGTGATGGC
NC_000001.11:33 ------------------------------------------------------------
NC_000023.11:15 -------------------------------------------------------GCGGC
NC_000004.12:c1 ------------------------------------------------------------

NC_000013.11:c3 GGGCGCCTGCAATCCCAGGTACTCGGGAAGCTGAGGCAGGAGAATTGCTTGAACCCGAGA
NC_000001.11:33 ------------------------------------------------------------
NC_000023.11:15 CGGTGCCCGC--------------------------------------------------
NC_000004.12:c1 ------------------------------------------------------------

NC_000013.11:c3 GGCGGAGGTTGCAGTGAGTCGAGATCATGCCACTGCATTCCAGCCTGGCGTGCAGGCTGA
```

```
NC_000001.11:33 ------------------------------------------------------------
NC_000023.11:15 --CGGGGGCTGC------------------------------------------------
NC_000004.12:c1 ------------------------------------------------------------


NC_000013.11:c3 GATGGAGCAAGACTCCATCTCAAAAAAAAAAAAACAAACAATTAGTGTTTGTGCCAGGCA
NC_000001.11:33 ------------------------------------------------------------
NC_000023.11:15 ------------------------------------------------------------
NC_000004.12:c1 ------------------------------------------------------------


NC_000013.11:c3 CAGTGGCTCATGCCCGTAATCCCAGTACTTTGGGAGGCCGAAGCAGGAGGGTCTGTTGAG
NC_000001.11:33 ------------------------------------------------------------
NC_000023.11:15 --GCGGCTGGTGCCCG--------------------------------------------
NC_000004.12:c1 ------------------------------------------------------------


NC_000013.11:c3 GCCAGGAGTTCAAGACTAGCCCGGGCAACATAGTGAAGCCCTGTCCCTACACACACACAC
NC_000001.11:33 ------------------------------------------------------------
NC_000023.11:15 ------------------------------------------------------------
NC_000004.12:c1 ------------------------------------------------------------


NC_000013.11:c3 ACAAAAAGTACAAAAATTACTCAGACATGGTGGCCTGCACCTGTAGTCCCAGCTACTCAG
NC_000001.11:33 ------------------------------------------------------------
NC_000023.11:15 ---------------------------------------------------------CCG
NC_000004.12:c1 ------------------------------------------------------------


NC_000013.11:c3 GAGGCTGATGCAGGAGGACCACTTGAGCCCAAGAGCTGCAGTGAGCTGTGATTGTGTACT
NC_000001.11:33 ------------------------------------------------------------
NC_000023.11:15 GGGGCCGCCGCACGGGCAAACTTCGCTCCCGGG---------------------------
NC_000004.12:c1 ------------------------------------------------------------


NC_000013.11:c3 GCTCTCAGCCTGGGCAACAGCGTGAGAATCTGTTTATTACTCTGAACACTATCACTATTA
NC_000001.11:33 ------------------------------------------------------------
NC_000023.11:15 ---CTTGGCCCGG----CGCCGCGCGGGCCCGCAGGCTACGCCG----------------
NC_000004.12:c1 ---CTCGG----------------------------------------------------


NC_000013.11:c3 CTGTAAGAGTGATAGAGTTTATTACTAAGTTGTTGTTTTAGAATCTGTCAAATACAGAGA
NC_000001.11:33 ------------------------------------------------------------
NC_000023.11:15 ------------------------------------------------------------
NC_000004.12:c1 ------------------------------------------------------------


NC_000013.11:c3 TGCTGCCCCAATCTGGTCAGACATTCGTATTTATTTATTTATTCAGACAGCATCTCACTC
NC_000001.11:33 ------------------------------------------------------------
NC_000023.11:15 ------------------------------------------------------------
NC_000004.12:c1 ------------------------------------------------------------


NC_000013.11:c3 TGTCACCTAGGCTGGAGTTCAGTGGCTCAATTACGGCTCACTGCACCCTTGACCTCCTGG
NC_000001.11:33 ------------------------------------------------------------
NC_000023.11:15 --------------------------------------------------------CTGG
NC_000004.12:c1 ------------------------------------------------------------


NC_000013.11:c3 ACCCAAGCGAGCACCTGAGTAGCTGGGACCACAGGTGTATACCAGCATGACCAGCTATTT
NC_000001.11:33 -----------------AGTAGCAGAGACCTCAGA-------------------------
NC_000023.11:15 CTCTGCGTTAACATGGCCGTCGCGGAGCGCCCGGT-------------------------
NC_000004.12:c1 ------------------------------------------------------------
```

```
NC_000013.11:c3 TTCTTATTTTTTGTAGAGATGGGGTCTCACTCTGTTGCCCAGGCTGGTGTTGAACTCCTG
NC_000001.11:33 ------------------------------------------------------------
NC_000023.11:15 ------------------------------------------------------------
NC_000004.12:c1 ------------------------------------------------------------

NC_000013.11:c3 GGCACAAGAGTTCCTCCTACTTGGCCTCCCAAAGTGTTGAGATTACAGGTGTGAGCCATT
NC_000001.11:33 ------------------------------------------------------------
NC_000023.11:15 ------------------------------------------------------------
NC_000004.12:c1 ------------------------------------------------------------

NC_000013.11:c3 GCACCCAGCGTCTCTGATTCTTTTGATTCCATTGAAACTAAAGTTTCAGTACATCAAGAA
NC_000001.11:33 ------------------------------------------------------------
NC_000023.11:15 ------------------------------------------------------------
NC_000004.12:c1 ------------------------------------------------------------

NC_000013.11:c3 AGAAAGCAGCCCAAAGGAACTCACACCGTCCCTTTATTCACTATCAGGGACAATGTTAAT
NC_000001.11:33 ------------------------------------------------------------
NC_000023.11:15 ------------------------------------------------------------
NC_000004.12:c1 ------------------------------------------------------------

NC_000013.11:c3 AGGGACCGCTTCCAGCTGTCTGCACAATGTCCTTTACTGTCCAAGACCAGCACTTCCAAG
NC_000001.11:33 ------------------------------------------------------------
NC_000023.11:15 ------------------------------------------------------------
NC_000004.12:c1 ------------------------------------------------------------

NC_000013.11:c3 ATAGCGTGGTTCATTTGGTCTTAGGAACAGATACTGAGGAGTTGGTCCATAACCCACAGC
NC_000001.11:33 ------------------------------------------------------------
NC_000023.11:15 ------------------------------------------------------------
NC_000004.12:c1 ------------------------------------------------------------

NC_000013.11:c3 AAAGCTCTGCAGGCACCTGATACCATGGAGAGGTGTTCTGACATTCTTATACGTCCCCCA
NC_000001.11:33 ------------------------------------------------------------
NC_000023.11:15 ------------------------------------------------------------
NC_000004.12:c1 ------------------------------------------------------------

NC_000013.11:c3 AGGCAGAAACTGTAGGTTCTGAGTGACATCTCATCATAATTAGTGGAAGCATTAAGAGGC
NC_000001.11:33 ------------------------------------------------------------
NC_000023.11:15 ------------------------------------------------------------
NC_000004.12:c1 ------------------------------------------------------------

NC_000013.11:c3 AAGCAAGCATAATTCTGGGCAGAGAAGGGCTCCCATCATAAGAATTTTGCAAACCGCATA
NC_000001.11:33 ------------------------------------------------------------
NC_000023.11:15 ------------------------------------------------------------
NC_000004.12:c1 ------------------------------------------------------------

NC_000013.11:c3 CTCTTTTATTTTGTAACAATGCTTGTAATTTATCTTTTGGAAAGAGGCACACAGATCTTG
NC_000001.11:33 ------------------------------------------------------------
NC_000023.11:15 ------------------------------------------------------------
NC_000004.12:c1 ------------------------------------------------------------

NC_000013.11:c3 ATGTAACTTGTGTAATAGACACTGTTCGCTAATGAAATATCCTTTTCAAATGAAAGGAAA
NC_000001.11:33 ------------------------------------------------------------
NC_000023.11:15 ------------------------------------------------------------
```

```
NC_000004.12:c1 ------------------------------------------------------------


NC_000013.11:c3 ATATTAGTTTCCTAGTCAGGTGCTGTATTAGTCCATTCTCGTGCTGTTAATAAAGATATA
NC_000001.11:33 ------------------------------------------------------------
NC_000023.11:15 ------------------------------------------------------------
NC_000004.12:c1 ------------------------------------------------------------


NC_000013.11:c3 CCCAAGACTGGGTAATTTATAAAGGAAAGAAGTTTAATTGACTCACAGTTCAGCATGGCT
NC_000001.11:33 ------------------------------------------------------------
NC_000023.11:15 ------------------------------------------------------------
NC_000004.12:c1 ------------------------------------------------------------


NC_000013.11:c3 GGGGAGGCCTCAGGAAACTTACAATCGTGGTGGAAGGGGAACCAAAAAGGTCTTTCTTTA
NC_000001.11:33 ------------------------------------------------------------
NC_000023.11:15 ------------------------------------------------------------
NC_000004.12:c1 ------------------------------------------------------------


NC_000013.11:c3 CATGGCGACAGGAAGGAGAAGTGCCGAGCAAAAGCAGGAAAGCCCCTTATAAAACCGTCA
NC_000001.11:33 ------------------------------------------------------------
NC_000023.11:15 ------------------------------------------------------------
NC_000004.12:c1 ------------------------------------------------------------


NC_000013.11:c3 GATCTCATGAGAATTCACTCAGTATCACAAGAACAGCATGAGGGTAACCACCCCCATGAT
NC_000001.11:33 ------------------------------------------------------------
NC_000023.11:15 ------------------------------------------------------------
NC_000004.12:c1 ------------------------------------------------------------


NC_000013.11:c3 TCAATTACCTCCCACTGGTCCCTCCCACAACACGTGGGGATTATGGGAACTACAATTCAA
NC_000001.11:33 ------------------------------------------------------------
NC_000023.11:15 ------------------------------------------------------------
NC_000004.12:c1 ------------------------------------------------------------


NC_000013.11:c3 GATGAGATTTGGGTGAGGACACAGCCAAACTCTATCAGGTGCTTATGATCATTTTAAAAC
NC_000001.11:33 ------------------------------------------------------------
NC_000023.11:15 ------------------------------------------------------------
NC_000004.12:c1 ------------------------------------------------------------


NC_000013.11:c3 CTGAAACAGTGGTATTCCTATTGCAAATTTTCTTAGAAAAACACCATTAGATAAAATGCA
NC_000001.11:33 ------------------------------------------------------------
NC_000023.11:15 ------------------------------------------------------------
NC_000004.12:c1 ------------------------------------------------------------


NC_000013.11:c3 ACAAGCAATAAAATAGCATTTTATGTTGTAGAATGATAACTTTTATTTTCTCCTAAACAT
NC_000001.11:33 ------------------------------------------------------------
NC_000023.11:15 ------------------------------------------------------------
NC_000004.12:c1 ------------------------------------------------------------


NC_000013.11:c3 ATTGACTAACCCATATCTTTTTTTGAGACAGAGTCTCACTCACTCTGTTGCTCAGGCTGG
NC_000001.11:33 ------------------------------------------------------------
NC_000023.11:15 ------------------------------------------------------------
NC_000004.12:c1 ------------------------------------------------------------


NC_000013.11:c3 ACTCCAGTGGCACAATCTTGGCTCACTGCAGCCTCTGCCTCCCGGGTTCAAGCAATCCTC
```

```
NC_000001.11:33 -------------------GGCTCA-----------------------------------
NC_000023.11:15 -------------------GGCCCG-----------------------------------
NC_000004.12:c1 -------------------GCCCCG-----------------------------------
                                   * *.*.

NC_000013.11:c3 CCTGCTTCAGCCTCCCAAGCAGCTGGGATCACAAGTGTGTGCCACCACGCCTGGCTGATT
NC_000001.11:33 ------------------------------------------------------------
NC_000023.11:15 ------------------------------------------------------------
NC_000004.12:c1 ------------------------------------------------------------


NC_000013.11:c3 TTTGTATTTTTAGTAGAGACGGGGTTTCACCACATTGGCCAGGCTGGTCTCAAACTCCTG
NC_000001.11:33 -------------TAAAAGTAGAGTTTTACCACCCTAGGCAGG-----------------
NC_000023.11:15 -------------GGGGGGCGCGGGCCGGCCGCCCCCCTCTCG-----------------
NC_000004.12:c1 ------------------------------------------------------------


NC_000013.11:c3 ACTTCAAGTGATCCACCAGCCTTGGCCTCCCAAAGTGAGCCACTGTGCCTAGCCATATCT
NC_000001.11:33 ------------------------------------------------------------
NC_000023.11:15 -------------------------CCTCCC-----------------------------
NC_000004.12:c1 ------------------------------------------------------------


NC_000013.11:c3 TTGTATTAAAAAAAAAAAAAATCTAAAGTCCTGTTTGTAAGGACTCTTTTCCCATGGTGT
NC_000001.11:33 ------------------------------------------------------------
NC_000023.11:15 ------------------------------------------------------------
NC_000004.12:c1 ------------------------------------------------------------


NC_000013.11:c3 TAGACAGACAACATAATGCTCTATAGCAAACCACCCCAAACTCAGTGGTTTGTATCAACA
NC_000001.11:33 ------------------------------------------------------------
NC_000023.11:15 ------------------------------------------------------------
NC_000004.12:c1 ------------------------------------------------------------


NC_000013.11:c3 CGTATCTGTTCTCATATTCACAGGTTAAGGTTGGCTGAGGTTTAGCATCTATGGAATGGG
NC_000001.11:33 ------------------------------------------------------------
NC_000023.11:15 ------------------------------------------------------------
NC_000004.12:c1 ------------------------------------------------------------


NC_000013.11:c3 CTCAGCTCTTGACTCGGCTTAAGGCTGTGGGTAGAGGAAACCAATCTTGAGTCAGTTGAA
NC_000001.11:33 ------------------------------------------------------------
NC_000023.11:15 ------------------------------------------------------------
NC_000004.12:c1 ------------------------------------------------------------


NC_000013.11:c3 AATAAGCTTGCCCTGTGTTTTGTTCATGAGGTTTTGCCAACATACATAATTATTAAACTT
NC_000001.11:33 ------------------------------------------------------------
NC_000023.11:15 -----GCCCGCCCGGCG-------------------------------------------
NC_000004.12:c1 ------------------------------------------------------------


NC_000013.11:c3 TCACTGAAAATGTTTAGCAACAATATTTTTTATGAAATGGGAGGGTAAAGTGAGAAAACT
NC_000001.11:33 ------------------------------------------------------------
NC_000023.11:15 ------------------------------------------------------------
NC_000004.12:c1 ------------------------------------------------------------


NC_000013.11:c3 TAAAACTGGAGCAGTGCCTTACCTGTAGTAAGTTCTCAAATAATATTTGGGAAAAGAATA
NC_000001.11:33 ------------------------------------------------------------
NC_000023.11:15 ------------------------------------------------------------
NC_000004.12:c1 ------------------------------------------------------------
```

```
NC_000013.11:c3 AAACAATGGAATATAAAGAAATCAGAGATGATAAAATTTTCTTAAAACTGTAACTAAGCA
NC_000001.11:33 -------AGGAGATAAAGAAA---------------------------------------
NC_000023.11:15 ------------------------------------------------------------
NC_000004.12:c1 ------------------------------------------------------------

NC_000013.11:c3 AACCTTGGACAAAACCTGACAAAGCTAAGCAGCAGAACACTTTTTCCCCATTGCCTTTGT
NC_000001.11:33 ------------------------------------------------------------
NC_000023.11:15 --------------------------------------------CCGCCGCCGCCGCTAA
NC_000004.12:c1 ------------------------------------------------------------

NC_000013.11:c3 CATGAGTTATGTGAATAACATCTCAGGAGGACTTCAGGCTTGGCATGGGCCACACACATT
NC_000001.11:33 ------------------------------------------------------------
NC_000023.11:15 CATGG-----------------------------CTGGCGCGGCGCTGGCCTCC------
NC_000004.12:c1 ------------------------------------------------------------

NC_000013.11:c3 ATCTCTGCTCCTTGCTGAAAACCCATCAGTAATGCTGAAGGAGTAGGATAGGGATAAAGA
NC_000001.11:33 -------------------------------------------------GGGGGCAATGC
NC_000023.11:15 ----------------------------------------------GGCGAGGGCAG---
NC_000004.12:c1 ------------------------------------------------------------

NC_000013.11:c3 ATGTTTTGAAAGATGGGAGACACATGGACCAGTGGTAATTAACAGCATGAAACCTACACC
NC_000001.11:33 CTGTACTGGAAGGAGAGAGTCATTTAGA--------------------------------
NC_000023.11:15 --------------GGGAGGCGGGCGGAGCGGTCGG------------------------
NC_000004.12:c1 ------------------------------------------------------------

NC_000013.11:c3 TACAAATGTGTGTGTTGTAGGGGGTTAGTTAAGAGTCAAGTTTCAAGAAAGGGGAGCCTT
NC_000001.11:33 ------------------------------------------------------------
NC_000023.11:15 ------------------------------------------------------------
NC_000004.12:c1 ------------------------------------------------------------

NC_000013.11:c3 CAGGTACCCCTAAAGGTGGATTCACGGTAGGATTGAAAATTCAATAATTAATGAAATATT
NC_000001.11:33 ------------------------------------------------------------
NC_000023.11:15 ------------------------------------------------------------
NC_000004.12:c1 ------------------------------------------------------------

NC_000013.11:c3 GTATAAAGAATAATTAAACTGGACTGGGCATGGTGGCTTATGCCTGTAATCCCAGCACTT
NC_000001.11:33 ------------------------------------------------------------
NC_000023.11:15 ------------------------------------------------------------
NC_000004.12:c1 ------------------------------------------------------------

NC_000013.11:c3 TGAAAGGCTGAGGTAGGAGGATCAGTTGAGGCCAGGAGTTCAAGACAAGTCTGGACAACA
NC_000001.11:33 ------------------------------------------------------------
NC_000023.11:15 ------------------------------------------------------------
NC_000004.12:c1 ------------------------------------------------------------

NC_000013.11:c3 TAGCAAGACCCCATCTCTAAGAAGAAATACAAAAATTAGCTGGGCATGGTGGCACCCGCC
NC_000001.11:33 ------------------------------------------------------------
NC_000023.11:15 ------------------------------------------------------------
NC_000004.12:c1 ------------------------------------------------------------

NC_000013.11:c3 TGTAGTTCCAGTTACTCAGAAGGCTGAACTGGGAGCACCACTTGAGCCCAGTAGGTTAAG
NC_000001.11:33 ------------------------------GAGGACACCTTATGAGGACCA---------
NC_000023.11:15 ------------------------------CGGGGCGCCCGGCGGGCTCCG---------
```

```
NC_000004.12:c1 ------------------------------GGAGCCACTTGCTGGG--------------
                                               ... *.*.   .*.*

NC_000013.11:c3 GCAGCAGTGAGCTTTAATTGTTCCACTGAACTATAGCTTGGACAACAGAGCAAGACCCAG
NC_000001.11:33 ------------------------------------------------------------
NC_000023.11:15 ------------------------------------------------------------
NC_000004.12:c1 ------------------------------------------------------------


NC_000013.11:c3 TCTCTAAAAAAAGAGGAAAATCACACTTCCAGATTCCCTCCTCAACAGTGTAGTTAGGAA
NC_000001.11:33 ------------------------------------------------------------
NC_000023.11:15 ------------------------------------------------------------
NC_000004.12:c1 ------------------------------------------------------------


NC_000013.11:c3 ACCACTTCTCCCCAAGTATAAAACGTGAGGTTGGCCGGGCTCAGTGGCTCACGCCTGTAA
NC_000001.11:33 ------------------------------------------------------------
NC_000023.11:15 ------------------------------------------------------------
NC_000004.12:c1 ------------------------------------------------------------


NC_000013.11:c3 TCCCAGCATTTTGGGAGGCCAAGGCAGGCAGATCACGAGGTCAGGAGACCAACACCATCC
NC_000001.11:33 ------------------------------------------------------------
NC_000023.11:15 ------------------------------------------------------------
NC_000004.12:c1 ------------------------------------------------------------


NC_000013.11:c3 TGGCCAACATGGTGAAACCCCGTCTCTACTAAAATACAAAAAATTAGCCAGGCGTGGCGA
NC_000001.11:33 ------------------------------------------------------------
NC_000023.11:15 ------------------------------------------------------------
NC_000004.12:c1 ------------------------------------------------------------


NC_000013.11:c3 TGCACACCTGTAGTCCCAGCTACTCGGGAGGCTGAGGCAGGGGAATTGCTTGAACCCGGG
NC_000001.11:33 ------------------------------------------------------------
NC_000023.11:15 ------------------------------------------------------------
NC_000004.12:c1 ------------------------------------------------------------


NC_000013.11:c3 AGGCAGAGATTACAGTGGGCTGAGATCACGCTACTGAACTCCAGCCTGGCAACAGAGCAA
NC_000001.11:33 ------------------------------------------------------------
NC_000023.11:15 ------------------------------------------------------------
NC_000004.12:c1 ------------------------------------------------------------


NC_000013.11:c3 GACTCCGTCTCAAAACAAACAAACAAAACGTGAGGTTTAGTCTCCAGCAGTGTGCCATAG
NC_000001.11:33 ------------------------------------------------------------
NC_000023.11:15 ------------------------------------------------------------
NC_000004.12:c1 ------------------------------------------------------------


NC_000013.11:c3 AACTTTCCGTGATGATGGAAGTGCTTTATATCTGCATTGTCCAACAACTAGTGGCTCAAT
NC_000001.11:33 ------------------------------------------------------------
NC_000023.11:15 ------------------------------------------------------------
NC_000004.12:c1 ------------------------------------------------------------


NC_000013.11:c3 AGCCACCTGAAATGTAGCCAGTGTGGCTGCACGTGGTGGCTCATACCTGTCATCCCAACA
NC_000001.11:33 ------------------------------------------------------------
NC_000023.11:15 ------------------------------------------------------------
NC_000004.12:c1 ------------------------------------------------------------


NC_000013.11:c3 CTTTGGGAGGCCGAGGTGGGTGGTTCGCTTGAGCCCAGGAGTTCAAGACCAGCTTGGGCA
```

```
NC_000001.11:33 ----------------------GTGACCTTCAGTCCAGGGACACAGAACCAG--------
NC_000023.11:15 --------------------------GCCTCGGCCCCGGCGCCCGCGGCCCG--------
NC_000004.12:c1 ----------------------------CTGAGCCGCGGCCGCTCGGGCCGG--------
                                            .* .*.*  **    .  ..** *

NC_000013.11:c3 ACATGTTGAAAACCCGTTTCTATAAAAAACACAAAAATTTGCTGGGCATGGTGGCACACA
NC_000001.11:33 ------------------------------------------------------------
NC_000023.11:15 ------------------------------------------------------------
NC_000004.12:c1 ------------------------------------------------------------

NC_000013.11:c3 CTTGCAGTCCTAGCTACTCAGGAAGTTGAAGCTGAGGCAGGAGGATCTCTTGAGCCCAGG
NC_000001.11:33 ------------------------------------------------------------
NC_000023.11:15 ----------------------------------GGGCGGGCGG----------------
NC_000004.12:c1 ----------------------------------GAGGAGGAGGA---------------

NC_000013.11:c3 AGGTTGAGGCTACAGTGATCCATGATTGCACCACTGCACTCCAGCCTGGGATACAGAGTA
NC_000001.11:33 ------------------------------------------------------------
NC_000023.11:15 ------------------------------------------------------------
NC_000004.12:c1 ------------------------------------------------------------

NC_000013.11:c3 AGACCTTGTCTCAAGAAAAACCCACAAACAAACAAAAATACATGAAATATGGCCAGTGTG
NC_000001.11:33 ------------------------------------------------------------
NC_000023.11:15 ------------------------------------------------------------
NC_000004.12:c1 ------------------------------------------------------------

NC_000013.11:c3 ACTGAGGAAATGAAGTTTAGTTTTAATTTTGATTTAAATAGCCACATATGGCTAGTGGCT
NC_000001.11:33 ------------------------------------------------------------
NC_000023.11:15 ------------------------------------------------------------
NC_000004.12:c1 ------------------------------------------------------------

NC_000013.11:c3 GACATATTGGACAGCGAAGCTCTAGACTCAGGAATACTGGGCACTGCTGAGAATGGGATG
NC_000001.11:33 ------------------------------------------------------------
NC_000023.11:15 ------------------------------------------------------------
NC_000004.12:c1 ------------------------------------------------------------

NC_000013.11:c3 GATTCTTTTGCAGAAAAACAGGAAGTGAGTAGAATTCTTCAAGTGACTGCTGAGATCTGT
NC_000001.11:33 ------------------------------------CTCCAAGTGAC-------------
NC_000023.11:15 ------------------------------------CTCGGAGGGCC-------------
NC_000004.12:c1 ------------------------------------------------------------

NC_000013.11:c3 CAACCTCTTCTATTACATGGGGCCCAGGTTGGAGGATTCCTTTCTGACAAAACTATGCTG
NC_000001.11:33 ------------------------------------------------------------
NC_000023.11:15 ------------------------------------------------------------
NC_000004.12:c1 ------------------------------------------------------------

NC_000013.11:c3 CCAGATTTATATGAATAACAGAATAATATTAATATTATTAATATATTAATAATAATAATA
NC_000001.11:33 ------------------------------------------------------------
NC_000023.11:15 ------------------------------------------------------------
NC_000004.12:c1 ------------------------------------------------------------

NC_000013.11:c3 TTCAGTTATTCTCAATAGAGCAGCCGGCACTCTAATTCATCACCCAGTGACAACACCCTC
NC_000001.11:33 ------------------------------------------------------------
NC_000023.11:15 ------------------------------------------------------------
NC_000004.12:c1 ------------------------------------------------------------
```

```
NC_000013.11:c3 ACACAGAGTTAGCATTTGTTATTGTCGTTTTGTTTTGAGATAGGGTCTCACTGTATTGCC
NC_000001.11:33 ------------------------------------------------------------
NC_000023.11:15 ------------------------------------------------------------
NC_000004.12:c1 ------------------------------------------------------------

NC_000013.11:c3 CAGGCTGACCTTGAACTCCTGGCTCAAGCAATCCTCCATCCTCAGCCTCACATCCATCAG
NC_000001.11:33 ------------------------------------------------------------
NC_000023.11:15 ------------------------------------------------------------
NC_000004.12:c1 ------------------------------------------------------------

NC_000013.11:c3 CATTTTAAATGCCTCGCTCTCAAATCCGACTAGGTAGCCAAGGATCACCATGCATGTGGG
NC_000001.11:33 ------------------------------------------------------------
NC_000023.11:15 ------------------------------------------------------------
NC_000004.12:c1 ------------------------------------------------------------

NC_000013.11:c3 GAAAGCTGCCCAGCTGAAATGGAAATCAATGCAAACAGAAGAAAAAGAACTGAAACAAGA
NC_000001.11:33 ------------------------------------------------------------
NC_000023.11:15 ------------------------------------------------------------
NC_000004.12:c1 ------------------------------------------------------------

NC_000013.11:c3 CATGACAGGGAACATAAGAAAAAAAAATAGTTTTTTTAAGAGAGAAGATATTGCAACCTAA
NC_000001.11:33 ------------------------------------------------------------
NC_000023.11:15 ------------------------------------------------------------
NC_000004.12:c1 ------------------------------------------------------------

NC_000013.11:c3 AACAAGATCAGAGGCTATATAAAAGATTGGAGGCCAGGAGTGCAGAGGAACACGTTTCTA
NC_000001.11:33 ------------------------------------------------------------
NC_000023.11:15 -----------------------------------GGGGGCGC-----------------
NC_000004.12:c1 ------------------------------------------------------------

NC_000013.11:c3 CATCGAGGCGCCTCCTGACTTACCTGAATTCCAACAGAGCACAGTAAGCGCCAGTTCTCT
NC_000001.11:33 ------------------------------------------------------------
NC_000023.11:15 --------------------------------------------------------CGCT
NC_000004.12:c1 ------------------------------------------------------------

NC_000013.11:c3 GCCTGTCCTGGATTGGGCCCTGCAGTTGCAGAGAAAGCTCAGGAATAAAAATGGAGGTAA
NC_000001.11:33 ------CCTGGGGTGG--------------------------------------------
NC_000023.11:15 GCGGGCCCTGGAGCGGCC------------------------------------------
NC_000004.12:c1 ------------------------------------------------------------

NC_000013.11:c3 ATCTTTAAGTGATTACCTATTCTTCTGACCCACCAGCTACAAATCAGGCGTTCCCACCTC
NC_000001.11:33 ------------------------------------------------------------
NC_000023.11:15 ------------------------------------------------------------
NC_000004.12:c1 ------------------------------------------------------------

NC_000013.11:c3 CTTCTCCTCAGGTTCGATTGATTTGACAGAGCAGCTTACAGAGCTCAGGAAAACATTTTA
NC_000001.11:33 ------------------------------------------------------------
NC_000023.11:15 --------------------------------------------------GAGCGTCATG
NC_000004.12:c1 ------------------------------------------------------------

NC_000013.11:c3 CTTATGTTGACCTATTTATTATAGTGGATACAGAGGAACAGATGGGAAAGATGCATAGGG
NC_000001.11:33 -------------------------GGATCCAGGAGAATAAATG----------------
NC_000023.11:15 GCTGCACGGGCGCCTTTGTT-------ATCCCGAGGAGTGCGCC----------------
```

```
NC_000004.12:c1 ------------------------------------------------------------


NC_000013.11:c3 CCAGGTGTGGGGGAAGGGTGCAGAGCTTCCATGCCCTTTTCAGGGGCACCACGTTCCAGG
NC_000001.11:33 ------------------------------------------------------------
NC_000023.11:15 ------------------------------------------------------------
NC_000004.12:c1 ------------------------------------------------------------


NC_000013.11:c3 AACCTCCACGTTTTCAGCTGTCTGGAAGCTCTGCGAACCCAGTCCTTTGAGGGTTTTATG
NC_000001.11:33 ------------------------------------------------------------
NC_000023.11:15 ------------------------------------------------------------
NC_000004.12:c1 ------------------------------------------------------------


NC_000013.11:c3 GAAGCGTCATTACATAGGCATGATGGACTACATCATTGCCTATTGCTGATCAACTCAACC
NC_000001.11:33 ------------------------------------------------------------
NC_000023.11:15 ------------------------------------------------------------
NC_000004.12:c1 ------------------------------------------------------------


NC_000013.11:c3 TTCCGCCCCTCTTCCCTCCCCGGAGGTTGGGGGAGTGAGACTGAAATTCTAACTCTCCAA
NC_000001.11:33 ------------------------------------------------------------
NC_000023.11:15 --------------CCGCGCCGGGGGGCGGGGAGG-------------------------
NC_000004.12:c1 ------------------------------------------------------------


NC_000013.11:c3 TCTTGCCTTGTTCTTTCTGGTGCCAGCCTCCATCCTGAAGCTACTTAGGGGTCCCCAGCC
NC_000001.11:33 CCTTGACTTA-----------ACTTTCCTCCATCTCTGATCTCCTGAGGG----------
NC_000023.11:15 ---------------------TCCGGCCCGGGCCCCCGAGCCCCCGGGCGG---------
NC_000004.12:c1 ------------------------------------------------------------


NC_000013.11:c3 ATTAGTCATCTCATTAGCAAAGCAAGATACCCTTATTGGTCTGGCCGTTCCTAGGGCTTG
NC_000001.11:33 ------------------------------CCCCATTGACC-------------------
NC_000023.11:15 -----------------------------CCCGGGCGGGGCGGGGC--------------
NC_000004.12:c1 ------------------------------------------------------------


NC_000013.11:c3 AGGAACTGTGGCTAGGAAACAGAGATGAAGGCCAGTGGATCACTGGAAGGCTCCCACTAT
NC_000001.11:33 ----------------AAAGTGAGATGGAAACCAGAGGG---------------------
NC_000023.11:15 ----------------GGGGCGGGGTGGGGGCCGACGGG---------------------
NC_000004.12:c1 ----------------GGAGGAGGGTGTGCGCCGGGGCG---------------------
                                ...  ..*.** . .**.. * .

NC_000013.11:c3 CTCACTTTTGGGGCAGAAGTGGACATTCTGAGGTGAGGAGTGGTTGGTTCAGAACCTCTG
NC_000001.11:33 ------------------------------------------------------------
NC_000023.11:15 ---------CGGGCGGCGGCGGC------GGGGCCCGGGCCGCTTTGTTC----------
NC_000004.12:c1 ---------CGGGCGGGGGCGCG-------------------------------------


NC_000013.11:c3 ACTTGCATGTTCAATCCCAGGCAGAGAGACATCGCTAGGACCTGCCAGGGTGGGTAACCT
NC_000001.11:33 ------------------------------------------------------------
NC_000023.11:15 ------------------------------------------------------------
NC_000004.12:c1 ------------------------------------------------------------


NC_000013.11:c3 GCCCACATGGCAGTCACCTGCAGGTGTGGGTGCAGACACATCTAGCTGAGAGTTGAGTGC
NC_000001.11:33 ------------------------------------------------------------
NC_000023.11:15 ------------------------------------------------------------
NC_000004.12:c1 ------------------------------------------------------------


NC_000013.11:c3 AACCATTCCAGATGCCCATCCCCAGAAAGAAAATCTCAGACCAGTAAAGCTCTGAATGGA
```

```
NC_000001.11:33 ------------------------------------------------------------
NC_000023.11:15 ------------------------------------------------------------
NC_000004.12:c1 ------------------------------------------------------------

NC_000013.11:c3 GGTTTGATCTCAATACCAAGGACAATATAAGCAGAGTTGCTTTAAGGATGCTATTGCTGT
NC_000001.11:33 ------------------------------------------------------------
NC_000023.11:15 ----------------------------------------------GCCGCTGTCGCCGC
NC_000004.12:c1 ------------------------------------------------------------

NC_000013.11:c3 TGTCCATGGGAAAGACCCTTTATGCCTGAATTATTTTGATTCCAGATTGAAGGAAAAACT
NC_000001.11:33 ------------------------------------------------------------
NC_000023.11:15 CGCCCGCG----------------------------------------------------
NC_000004.12:c1 ------------------------------------------------------------

NC_000013.11:c3 GCTAATTTCAGTTCGTTTTATAGAGGCTGGAGTTTTGGGCATTCCTGTTTGACTGTAAAC
NC_000001.11:33 ------------------------------------------------------------
NC_000023.11:15 ------------------------------------------------------------
NC_000004.12:c1 ------------------------------------------------------------

NC_000013.11:c3 TCTCAATAAGGTAGGGAAATAAATCAGAAATCATGGGTGTACATTTTTAGAGACCTTGAC
NC_000001.11:33 ------------------------------------------------------------
NC_000023.11:15 ------------------------------------------------------------
NC_000004.12:c1 ------------------------------------------------------------

NC_000013.11:c3 AGACTTATTTTGCAGTTGGGGCTTCAGGGCCCAGAATCTTTTAGCATCCCTGCCCTAGTT
NC_000001.11:33 ------------------------------------------------------------
NC_000023.11:15 ------------------------------------------------------------
NC_000004.12:c1 ------------------------------------------------------------

NC_000013.11:c3 GAAATCAGACACACCCTCTCCCTGGTATCCAAACAAAGCTAGTTCAGGTGCTGCCCTGAA
NC_000001.11:33 ------------------------------------------------------------
NC_000023.11:15 ------------------------------------------------------------
NC_000004.12:c1 ------------------------------------------------------------

NC_000013.11:c3 GGGATTTGGTTGATGTGAAGTTCCAAGTCCTTAAATTAATCAACAGGAGTACTCTCCTGG
NC_000001.11:33 ------------------------------------------------------------
NC_000023.11:15 --------------------------------------------------------CCGG
NC_000004.12:c1 ------------------------------------------------------------

NC_000013.11:c3 ATGGGCCTGATTTGATCAGGTGGGTGACATGTAAGTTCTCCTCCTTTACAGGGGAAGACA
NC_000001.11:33 ------------------------------------------------------------
NC_000023.11:15 CCGCGCGCGGATCAGCCA-------------------------TTTTAGCGAGTGGGACT
NC_000004.12:c1 ------------------------------------------------------------

NC_000013.11:c3 CCAGGACTCGGAGAGTCCCACTTCCCACCTCAGACGCTCTCAAGATGGTGACCTCTCTGG
NC_000001.11:33 ------------------------------------------------------------
NC_000023.11:15 CCGAGGCGCGGCGGACGCCGCCACC-----------------------------------
NC_000004.12:c1 ------------------------------------------------------------

NC_000013.11:c3 GTTTTAACACCTGGAACAGGCCGGGTGCAGTGGCTCACACCTGTCCATCCCAGCACTTTG
NC_000001.11:33 ------------------------------------------------------------
NC_000023.11:15 -----------------------AGTGCCGCGGCTGCCGCCGGCCCGGCC----------
NC_000004.12:c1 ------------------------------------------------------------
```

```
NC_000013.11:c3 GGAGGCTGAGATGGGCAGATCAACTGAGGTTGGGAGTTTGAGACCAGCCTGGCCAACATG
NC_000001.11:33 ------------------------------------------------------------
NC_000023.11:15 ------------------------------------------------------------
NC_000004.12:c1 ------------------------------------------------------------

NC_000013.11:c3 GTGAAACCCTATCTCTACTAAAAATACAAAAATTAGCCAGGCGTGGTGGTGCACACCTGT
NC_000001.11:33 ------------------------------------------------------------
NC_000023.11:15 --------------------------------------------------GCACACC---
NC_000004.12:c1 ------------------------------------------------------------

NC_000013.11:c3 AATCCCAGCTACTCTGGAGGCTGAGGCATGAAAATCGCTTGAACCTGGGAGGCGGAGGTT
NC_000001.11:33 ------------------------------------------------------------
NC_000023.11:15 ------------------------------------------------------------
NC_000004.12:c1 ------------------------------------------------------------

NC_000013.11:c3 GCAGTGAGCTGAGATCGCGCCACTGCACTCTAGCCTGGGCAACAGAGTGAGCCTCTGTCT
NC_000001.11:33 ------------------------------------------------------------
NC_000023.11:15 ------------------------------------------------------------
NC_000004.12:c1 ------------------------------------------------------------

NC_000013.11:c3 TAAAAAAAAAAAAAAAAAAAAAAAAAAACCTGGAACAGGCAGAGCCTGGAGGATGTGTGT
NC_000001.11:33 ------------------------------------------------------------
NC_000023.11:15 ------------------------------------------------------------
NC_000004.12:c1 ------------------------------------------------------------

NC_000013.11:c3 GGACTTCCCATCTGTGCCAAATGTGCTTTGGAGAAAACCCACATAGCTGAATGATTAAAG
NC_000001.11:33 ------------------------------------------------------------
NC_000023.11:15 ------------------------------------------------------------
NC_000004.12:c1 ------------------------------------------------------------

NC_000013.11:c3 TCAAGCACGGGAAGGGATCAGCTATGTAGCAGTGCCCTGTGCACCTTGGCGGAAAGGAAT
NC_000001.11:33 ------------------------------------------------------------
NC_000023.11:15 ----------------------------------CCCCGCGCACC---------------
NC_000004.12:c1 ------------------------------------------------------------

NC_000013.11:c3 TTTTCTGTCCATTGACATTGTAGGTGTTCAGTTTGGAATCTTTCAGTTTTGGGAGTCGCC
NC_000001.11:33 ------------------------------------------------------------
NC_000023.11:15 ------------------------------------------------------------
NC_000004.12:c1 ------------------------------------------------------------

NC_000013.11:c3 ACCCATCAACTTGTCAGTCCCAAGGGCACTCATGAAGCAGTCGCTCATTGACTCTGCACT
NC_000001.11:33 ------------------------------------------------------------
NC_000023.11:15 ------------------------------------------------------------
NC_000004.12:c1 ------------------------------------------------------------

NC_000013.11:c3 GGGGCCGTCCTGCATCACAGTGAGAAATGGGAGACTTCCACAGAGAAAGTGAAGGTCCAA
NC_000001.11:33 ------------------------------------------------------------
NC_000023.11:15 ------------------------------------------------------------
NC_000004.12:c1 ------------------------------------------------------------

NC_000013.11:c3 GGTCCAACGTTGTGTGTCCAGAAGAAGGCCAGACTGTGCCCCTGTGGAGTCCTCTGATGA
NC_000001.11:33 ------------------------------------------TATGGGATCCTCTGATGT
NC_000023.11:15 -------------------------------------GCCACCGCCGCCGCCCCCGGCCC
```

```
NC_000004.12:c1 ------------------------------------------GGCGGCCGCCCTTGGGAG
                                                           .. *   **...*.

NC_000013.11:c3 GGCGGATGAGGAATTTTCATTCATTGGGAAATCCAAGGAACAGGAAGCAGAGCCTGAGGA
NC_000001.11:33 GGCTCAT-----------------------------------------------------
NC_000023.11:15 CGCGCGT-----------------------------------------------------
NC_000004.12:c1 GACGCCC-----------------------------------------------------
                 .*   .

NC_000013.11:c3 ACAGGAGGAGGGCTCAGCCAGCCAGCCAGGCTGTGGCATTTTCAGGACCAGGTGAGTGAC
NC_000001.11:33 ------------------------------------------------------------
NC_000023.11:15 ------------------------------------------------------------
NC_000004.12:c1 ------------------------------------------------------------


NC_000013.11:c3 GAGAGGAAGAGAGATTGGCCGGCATTGGAAAACAGTGGAGCCTGGAGTGATAGAAGCAGG
NC_000001.11:33 ------------------------------------------------------------
NC_000023.11:15 ------------------------------------------------------------
NC_000004.12:c1 ------------------------------------------------------------


NC_000013.11:c3 CAGCTTCAGAAGAGAAGCAGGTGCCTAGCGCCCGGAATGAGCGGATGTCAGTGAAGAAGA
NC_000001.11:33 ------------------------------------------------------------
NC_000023.11:15 ----------------------------CCCCCGG-------------------------
NC_000004.12:c1 ------------------------------------------------------------


NC_000013.11:c3 AGAGGAAGAAACCGAGGATGAGGAGATGGGGTGGCAGTGCAGCACCCTGTGTCAGGGAGT
NC_000001.11:33 ---------------------------------ACAGACCAGCTTCCCAGGCCAGAGA--
NC_000023.11:15 ---------------------------------GGATGACAGCGGCGGATTTCAGGGG--
NC_000004.12:c1 ---------------------------------------------------TCGGGAG--
                                                                   .*.*...

NC_000013.11:c3 ACAGGAGAGGAGACAGGAGTGTGGGAGGCACAGGGGTGGCAGGGGAGGCGGTGAGGAGGG
NC_000001.11:33 ------------------------------------------------------------
NC_000023.11:15 ------------------------------------------------------------
NC_000004.12:c1 ------------------------------------------------------------


NC_000013.11:c3 GCGCCCTGGGGAGCTCAGAGCAGATCTGAGTATGGAGAGTCCACAGACAGTGAGGATGAG
NC_000001.11:33 ------------------------------------------------------------
NC_000023.11:15 ------------------------------------------------------------
NC_000004.12:c1 ------------------------------------------------------------


NC_000013.11:c3 ACGGAGCCTCGCCTGATGCAGGCCCTCACTGGGAAGGAAGAAGGGCTGAGTGACAGTTCG
NC_000001.11:33 ------------------------------------------------------------
NC_000023.11:15 ----------------CACTTTCTTTCATCAGG---------------------------
NC_000004.12:c1 ------------------------------------------------------------


NC_000013.11:c3 AGAACCTGAGGCTGAAGCATTGAACCAGAAGGAGCTGGAGCAGGAGGCAAAAGGCACCCT
NC_000001.11:33 --------AGGTTGAAG------------AGAGGTTGGAGAAGGCTGGAAAGGGGATCTG
NC_000023.11:15 --------AGGCTTTTG------------ACAAAATGGAAGGTGAATTACGGGTGTCCCG
NC_000004.12:c1 ------------------------------------------------GAGGGGGGCGCG
                                                                . ..*   . .

NC_000013.11:c3 GAGGAGGCATGGTGCCCCCTCCAGACTGTAGGAGACCATGGAAGAGCCTGCGGAGAGCAG
NC_000001.11:33 GA----------------------------------------------------------
NC_000023.11:15 GT----------------------------------------------------------
NC_000004.12:c1 GG----------------------------------------------------------
                *

NC_000013.11:c3 GCGGGGCCGCACTACTCTGGATGCGCTCAACACCAAGAGGAGAGTCAGGAGGAGCAGGAG
```

```
NC_000001.11:33 ------------------------------------------------------------
NC_000023.11:15 ------------------------------------------------------------
NC_000004.12:c1 ------------------------------------------------------------


NC_000013.11:c3 GCATGGAAAGTTCAGAAGCTAAAACAAACAAGGGAGCAGAGACGAGGAAGAAGAGCTTGA
NC_000001.11:33 ------------------------------------------------------------
NC_000023.11:15 ------------------------------------------------------------
NC_000004.12:c1 ------------------------------------------------------------


NC_000013.11:c3 GAAGGAGGAAGCAGAAATTGAGCACCTGCGGAAGCTGGCTGAGGAAGAGCAGCAAGCTGA
NC_000001.11:33 ------------------------------------------------------------
NC_000023.11:15 ------------------------------------------------------------
NC_000004.12:c1 ------------------------------------------------------------


NC_000013.11:c3 GCTTCAGGCATATGGCAAGGTCATTACCAGCAAAGCCATTCAGGGCAAATATGAGAAATA
NC_000001.11:33 -----------------------------------------AGGGCAACTCTTAGA----
NC_000023.11:15 ------------------------------------------------------------
NC_000004.12:c1 ----------------------------------------CAGGGC--------------


NC_000013.11:c3 TACAAAAGAGATTTCAGTGCCCCCACCCTGGAGGATCATTTCAACAAAGCCATGCTTCCT
NC_000001.11:33 ------------------------------------------------------------
NC_000023.11:15 ------------------------------------------------------------
NC_000004.12:c1 ------------------------------------------------------------


NC_000013.11:c3 AAAGTCATGCAAGAACTTTGGGCACTCTGGTTATACCAAATACACCCATGGTGGGCATTG
NC_000001.11:33 ------------------------------------------------------------
NC_000023.11:15 ------------------------------------------------------------
NC_000004.12:c1 ------------------------------------------------------------


NC_000013.11:c3 AGATACCACTTCCTTCTACCCAACACGGGGCCAAGAGAGTACCCAGAACACAAAGTTCTT
NC_000001.11:33 ---------------------------------------TACCCAGCACAC---------
NC_000023.11:15 ---------------------------------------GACCCGGCAC-----------
NC_000004.12:c1 ------------------------------------------------------------


NC_000013.11:c3 CAAACAAAAGGTAGCTGAAGCACAAGATGTAGCTGAGCAACCATTTACCAAGAAGCAGAA
NC_000001.11:33 -----------------------------------AGCATTGATTTA---GGAAGCCAGA
NC_000023.11:15 --------------------------------------------------GGGGGCCGAG
NC_000004.12:c1 --------------------------------------------------GGGAGCGGAT
                                                                  .*..** ..

NC_000013.11:c3 GACTACTTAGGGGTCAACTCCTTGTTCTCCCAAATGTGGAACTCAAGGGGAAATCTCAGC
NC_000001.11:33 ------------------------------------------------------------
NC_000023.11:15 ------------------------------------------------------------
NC_000004.12:c1 ------------------------------------------------------------


NC_000013.11:c3 ATCTGGTCCTTGATTTGCTTTTCCTGTTATTTACATGGCCCCTGAATCCAGCCTTTGGAC
NC_000001.11:33 ---------TTTATTTACCTTTCCT-----------------------------------
NC_000023.11:15 ---------GCGGGCTGCCTGCTGC-----------------------------------
NC_000004.12:c1 ------------------------------------------------------------


NC_000013.11:c3 CTACTGCCATGCCTGTGATACTGCGTAGCCCAGTTTTTGAAGGTGCTTTGTGAGATTTAG
NC_000001.11:33 ------------------------------------------------------------
NC_000023.11:15 ------------------------------------------------------------
NC_000004.12:c1 ------------------------------------------------------------
```

```
NC_000013.11:c3 ACTTGTGCTGAAAAATCCCCCAGAAAAGCACTATCCAGGTAGGATTAGAGGCTTCCCACT
NC_000001.11:33 -----------AAGCTCCCCTAAACAAGGAC-----------------------------
NC_000023.11:15 -----CGGCCGGGACTCCCCCGCTCCCGGTC-----------------------------
NC_000004.12:c1 ------------------------------------------------------------

NC_000013.11:c3 TACTTCCAGGAAATCTTAGGTTTTATCAATGCTAAGACTTCCCATATTTACATGAGATTG
NC_000001.11:33 ------------------------------------------------------------
NC_000023.11:15 ------------------------------------------------------------
NC_000004.12:c1 ------------------------------------------------------------

NC_000013.11:c3 TTCTGATTTTTAATTTTAGAGATGGAGTCTCGCTACGTTGCCCATGTTGGATTTGAACTC
NC_000001.11:33 ------------------------------------------------------------
NC_000023.11:15 ------------------------------------------------------------
NC_000004.12:c1 ------------------------------------------------------------

NC_000013.11:c3 CTGGGCTCAAGTGATCCTCCTGCCTCAGCCTCCCAAGTAACTGGGATTACAGGCATGTGC
NC_000001.11:33 --------------TTCCCCTGCTTCATCCTC----------------------------
NC_000023.11:15 --------------CCGCCCCGCCCGGGCCGCC---------------------------
NC_000004.12:c1 ------------------------------------------------------------

NC_000013.11:c3 CACCACACACTAGCTCTGAGTTTCGTTTTCCAGCCCCTTAGCTTGGGTCAGAAACATGGG
NC_000001.11:33 ------------------------------------------------------------
NC_000023.11:15 ------------------------------------------------------------
NC_000004.12:c1 ------------------------------------------------------------

NC_000013.11:c3 CATGTAAGTGGACAATAAATTTAACCCTTAGGGTTAGGAGTAAAATGCTAGGAAATCTAA
NC_000001.11:33 ------------------------------------------------------------
NC_000023.11:15 ------------------------------------------------------------
NC_000004.12:c1 ------------------------------------------------------------

NC_000013.11:c3 ATTCATTGAAACTTGTTTTTCATTTTTGGATGACGTCCAGTTTTCTAGATGAACATATTT
NC_000001.11:33 ------------------------------------------------------------
NC_000023.11:15 ------------------------------------------------------------
NC_000004.12:c1 ------------------------------------------------------------

NC_000013.11:c3 TTTGATCCATTTATGTGTTTGTATCAAGAAATAAAAGAAACACACACACACACACACCCA
NC_000001.11:33 ------------------------------------------------------------
NC_000023.11:15 ------------------------------------------------------------
NC_000004.12:c1 ------------------------------------------------------------

NC_000013.11:c3 AAAGAGAAAAAAACATACAAGAAGGAATGCAGTCTCTGTGCCAGGCCATTCCCAGTGTTT
NC_000001.11:33 ------------------------------------------------------------
NC_000023.11:15 ------------------------------------------------------------
NC_000004.12:c1 ------------------------------------------------------------

NC_000013.11:c3 CACTGGTGCCCTGGGGTCCACATGCTTTTTTAAAGAAGACTAGGCCAGGCACAGTGGCTC
NC_000001.11:33 ------------------------------------------------------------
NC_000023.11:15 ------------------------------------------------------------
NC_000004.12:c1 ------------------------------------------------------------

NC_000013.11:c3 ACGCCTGTAATCCCAGCACTTTGGGAGGCTGAGGCAGGCGGATCACCTGAGGTCGGGAGT
NC_000001.11:33 ------------------------------------------------------------
NC_000023.11:15 ------------------------------------------------------------
```

```
NC_000004.12:c1 ------------------------------------------------------------

NC_000013.11:c3 TCGAGACCAGCCTGACCAACATGGAGAAACCCTGTCTCTACTAAAAATACAAAATTAGCC
NC_000001.11:33 ------------------------------------------------------------
NC_000023.11:15 ------------------------------------------------------------
NC_000004.12:c1 ------------------------------------------------------------

NC_000013.11:c3 GGGCGTGGTGGCAGGTGCCTGTAATCCCAGCTACTCAGGAGGCTGAGGCAGGAGAACTGC
NC_000001.11:33 ------------------------------------------------------------
NC_000023.11:15 -------GCTGCGGGTCTCTG---------------------------------------
NC_000004.12:c1 ------------------------------------------------------------

NC_000013.11:c3 TTGAACCCGGGAGGCAGAGGTTGTGATGAGCCAAGATCGCGCCATTGCACTCCAGCTTGG
NC_000001.11:33 ------------------------------------------------------------
NC_000023.11:15 --------------------------------------------------------TTGG
NC_000004.12:c1 --------------------------------------------------------TTGG

NC_000013.11:c3 GCAACAAGAGCGAAAACTCCATCTCAAAAAAAAAAAAAAAAAAGACTAAAGTGTGCCAAA
NC_000001.11:33 ------------------------------------------------------------
NC_000023.11:15 GCA---------------------------------------------------------
NC_000004.12:c1 GCG---------------------------------------------------------

NC_000013.11:c3 TTTGCAGTAAATTGGAGAATGTCAGACCTGCCTCTTAGACGTAGAGTGTGGCCAGCACAT
NC_000001.11:33 ------------------------------------------------------------
NC_000023.11:15 -----------------------------GCCTCCTGGGC--------------------
NC_000004.12:c1 ------------------------------------------------------------

NC_000013.11:c3 CCAGGTTCATGACACAGCATTGTCTTTTGAAAGTAATGTGCCAAAGTCAGATGTCAACCG
NC_000001.11:33 ------------------------------------------------------------
NC_000023.11:15 ------------------------------------------------------------
NC_000004.12:c1 ------------------------------------------------------------

NC_000013.11:c3 AGGGTTCTACACACAGAATATGGAGAGAGATCTGTAACTCTGATGGAACACGTCTAATTA
NC_000001.11:33 ------------------------------------------------------------
NC_000023.11:15 ------------------------------------------------------------
NC_000004.12:c1 ------------------------------------------------------------

NC_000013.11:c3 GCATGCTGGGGAAAGACACATCCCCCAGTGTCGTGCTGCTCAAACTGGCCTGGACCATAC
NC_000001.11:33 ------------------------------------------------------------
NC_000023.11:15 ------------------------------------------------------------
NC_000004.12:c1 ------------------------------------------------------------

NC_000013.11:c3 CCTACTATCAAAGGAATCAGCCTCACATTTGCTCCTGCTAGGTGAAAGGAGAGTGTCAGA
NC_000001.11:33 ------------------------------------------------------------
NC_000023.11:15 ------------------------------------------------------------
NC_000004.12:c1 ------------------------------------------------------------

NC_000013.11:c3 GAAAAGAGGAGCGTCCCTACAGACAGAAGAAGCCTACAGATCTGGATGATCCCCTTGCTG
NC_000001.11:33 ------------------------------------------------------------
NC_000023.11:15 ------------------------------------------------------------
NC_000004.12:c1 ------------------------------------------------------------

NC_000013.11:c3 ATCAAAATGTTGAAGACTCATATTATGGAATCAATGACCCTATAGCTGATAAGCTTCTAA
```

```
NC_000001.11:33 ------------------------------------------------------------
NC_000023.11:15 ------------------------------------------------------------
NC_000004.12:c1 ------------------------------------------------------------

NC_000013.11:c3 AGCAGACTTCAACCATGCCTCATCTGGACCCACCAGAGGACAAGACTCTCACCACATTAG
NC_000001.11:33 ------------------------------------------------------------
NC_000023.11:15 ------------------------------------------------------------
NC_000004.12:c1 ------------------------------------------------------------

NC_000013.11:c3 ACGTGGATGGTCTGGGTGAAACAGATCAGAGAAATCATTTCTACCAGTTAGGAGAGATTG
NC_000001.11:33 ------------------------------------------------------------
NC_000023.11:15 ------------------------------------------------------------
NC_000004.12:c1 ------------------------------------------------------------

NC_000013.11:c3 ACCATCACTCTAATGCAGAAGCAGCAGGGTGCTTTCACCCAGTTTTCCACAAGGCAGGCT
NC_000001.11:33 ------------------------------------------------------------
NC_000023.11:15 ------------------------------------------------------------
NC_000004.12:c1 ------------------------------------------------------------

NC_000013.11:c3 ACAGACATGGCTGCTGAGAAGTCCTTTAATAAGTTGATTGCAATCGCCACAGACTCAACG
NC_000001.11:33 ------------------------------------------------------------
NC_000023.11:15 --------GGCTGCTG--------------------------------------------
NC_000004.12:c1 ------------------------------------------------------------

NC_000013.11:c3 TGAAATCAAGAAGATCTCAAGCAGCCAGAGGAAAAGAAAAAGAAGGACGGAACCACATAC
NC_000001.11:33 ------------------------------------------------------------
NC_000023.11:15 ------------------------------------------------------------
NC_000004.12:c1 ------------------------------------------------------------

NC_000013.11:c3 TCTGGGATCAAGTTAGAGCCTTTTCCAGGACTGCCATGAGCTCTTCCTCCTGTTGCAGCA
NC_000001.11:33 --------CAAGTTGGAACTTT--------------------------------------
NC_000023.11:15 --------CCAAGGGGAGCTCT--------------------------------------
NC_000004.12:c1 --------GGAAGCGGAGCCCC--------------------------------------
                          *.  .**.*...

NC_000013.11:c3 GCTACTTCAGCCTACCCCTAAGTGATCCTCCACCACTGTGGTGAACATTGCCCTGCCACC
NC_000001.11:33 ------------------------------------------------------------
NC_000023.11:15 ------------------------------------------------------------
NC_000004.12:c1 ------------------------------------------------------------

NC_000013.11:c3 CCCACCACTCCCCGTACTATCAGGTTTGGGGCTACACATGTTCCACCTCATTCCTTTCAT
NC_000001.11:33 ------------------------------------------------------------
NC_000023.11:15 ------------------------------------------------------------
NC_000004.12:c1 ------------------------------------------------------------

NC_000013.11:c3 GAAGACTCCAGGACCAATCCTTTTCAGGACCCTCAGAGGATGGGAGCTCATGCTGGAAAA
NC_000001.11:33 ------------------------------------------------------------
NC_000023.11:15 ------------------------------------------------------------
NC_000004.12:c1 ------------------------------------------------------------

NC_000013.11:c3 TACAGCTGCCCCCAGCACATTAGCACTACTCTGGGGCTCTGTGGAAGAAAGGGCACTTAA
NC_000001.11:33 ------------------------------------------------------------
NC_000023.11:15 ------------------------------------------------------------
NC_000004.12:c1 ------------------------------------------------------------
```

```
NC_000013.11:c3 AACTCCCAGTAAATGAATTTTGGAGTAAATATATTTTTCCTTCCTTCGTAGTTTCCATGG
NC_000001.11:33 ------------------------------------------------------------
NC_000023.11:15 ------------------------------------------------------------
NC_000004.12:c1 ------------------------------------------------------------

NC_000013.11:c3 TAGCTGAATATGCTCAGATATGGACAGTCAGTGAAGGACAGCCATGCTTCTCTACACGTG
NC_000001.11:33 ------------------------------------------------------------
NC_000023.11:15 ------------------------------------------------------------
NC_000004.12:c1 ------------------------------------------------------------

NC_000013.11:c3 TTCAAAGGATCAATGGCCTTCAAATAAGCTGCCGAGGCCACATCTGGTTACAAATACAAC
NC_000001.11:33 ------------------------------------------------------------
NC_000023.11:15 ------------------------------------------------------------
NC_000004.12:c1 ------------------------------------------------------------

NC_000013.11:c3 ATGACTAATAAAACCTAATGCCTGTTCCCCTCACCTCTGTGAGAATAAGTGGCTGGGTGA
NC_000001.11:33 ------------------------------------------------------------
NC_000023.11:15 ------------------------------------------------------------
NC_000004.12:c1 ------------------------------------------------------------

NC_000013.11:c3 ATTGGAATGCCTGATGGAGCTGCCATCTCAAAACAAAATGTTCATTTTGTACCTTTTCAG
NC_000001.11:33 ------------------------------------------------------------
NC_000023.11:15 ------------------------------------------------------------
NC_000004.12:c1 ------------------------------------------------------------

NC_000013.11:c3 CGGAGGCGGGCAGGGGGAGAATTGATCTTCAGTAAAAAACTTCTGGGCTGGCTGGGCGCA
NC_000001.11:33 ------------------------------------------------------------
NC_000023.11:15 ------------------------------------------------------------
NC_000004.12:c1 ------------------------------------------------------------

NC_000013.11:c3 GTGGCTTACGCCTGTAATCCCAGCACTTTGGGAGGCTGAGGCGGGCGGATCACTTAAGGT
NC_000001.11:33 ------------------------------------------------------------
NC_000023.11:15 ------------------------------------------------------------
NC_000004.12:c1 ------------------------------------------------------------

NC_000013.11:c3 CAGGAATTGGAGACCATCCAGGCCAACATGGTGAAACCGCATCTCTACTAAAAATACAAA
NC_000001.11:33 ------------------------------------------------------------
NC_000023.11:15 ------------------------------------------------------------
NC_000004.12:c1 ------------------------------------------------------------

NC_000013.11:c3 AATTAGCTGGGCATGGTGGCAGGCACCTGTAATCCCAGCTACTAGGGAGGCTGAGGCAGG
NC_000001.11:33 ------------------------------------------------------------
NC_000023.11:15 --TTAGTTTTACATGATGAC----------------------------------------
NC_000004.12:c1 ------------------------------------------------------------

NC_000013.11:c3 AGAATCTCTTGAACCCGGGAGGTGGAGGTTGCAGTGAGCCGAGATCGTGCCGTTGCACTC
NC_000001.11:33 ------------------------------------------------------------
NC_000023.11:15 ------------------------------------------------------------
NC_000004.12:c1 ------------------------------------------------------------

NC_000013.11:c3 CAGCCTGGGTGACAGAGCGAGACTCCATCTCAAAAAACAAAACAAAACAAAACACCTTTG
NC_000001.11:33 ------------------------------------------------------------
NC_000023.11:15 ---CTTGGGTG-------------------------------------------------
```

```
NC_000004.12:c1 ------------------------------------------------------------


NC_000013.11:c3 GGCCATTTTTATGTCCATGGGGGTGTTTTTCTTTGTATCATCTTCGAAAAAAAGAAAAAA
NC_000001.11:33 ------------------------------------------------------------
NC_000023.11:15 ---------------------------------------------------TGGGAAAAG
NC_000004.12:c1 ------------------------------------------------------------


NC_000013.11:c3 GAAAGAAAGGGGGAGAGAGAGAGAAAGGAAGAAAGAAAAAGAAAGAAGGAAAGAGAGAGA
NC_000001.11:33 ------------------------------------------------------------
NC_000023.11:15 GAAAAGATGGCGGGAAGCTGGGG-------------------------------------
NC_000004.12:c1 ------------------------------------------------------------


NC_000013.11:c3 GAAAGAGAAAGAAAGAAAAAGAAGAAAGAAAGAAAAAGAAAGAAAAAGAAGAAAGAAAGA
NC_000001.11:33 ------------------------------------------------------------
NC_000023.11:15 ------------------------------------------------------------
NC_000004.12:c1 ------------------------------------------------------------


NC_000013.11:c3 AAAAGAAAGAAAGAAAGAAAGGAAAGAAAGAAAGAAAGAAAGGAAAGAAAGAAAGAAAGA
NC_000001.11:33 ------------------------------------------------------------
NC_000023.11:15 ------------------------------------------------------------
NC_000004.12:c1 ------------------------------------------------------------


NC_000013.11:c3 AAGGAAGAAAGAAAAAGAAAGAGAGAGAGAAAGAGAAAGAAAGAAAAAGAAGAAAGAAAA
NC_000001.11:33 ------------------------------------------------------------
NC_000023.11:15 ------------------------------------------------------------
NC_000004.12:c1 ------------------------------------------------------------


NC_000013.11:c3 AGAAAGAAAGAAAGAAAAAGAAGAAAGAAAAAGAAAGAAAGAAAGAAAGGAAAGAAAGAA
NC_000001.11:33 ------------------------------------------------------------
NC_000023.11:15 ------------------------------------------------------------
NC_000004.12:c1 ------------------------------------------------------------


NC_000013.11:c3 AGGAAAGAAAGAAAGGAAAGAAAGAAAGAAAGAAAGAAAGAAAGAAAGAAAGAAAGAAAG
NC_000001.11:33 ------------------------------------------------------------
NC_000023.11:15 ------------------------------------------------------------
NC_000004.12:c1 ------------------------------------------------------------


NC_000013.11:c3 AAAGAAAGAAAGAATTGCTAGTACTAGTTACTGCAGTGGTGTCAGGGTGATTTAACTATT
NC_000001.11:33 ------------------------------------------------------------
NC_000023.11:15 ------------------------------------------------------------
NC_000004.12:c1 ------------------------------------------------------------


NC_000013.11:c3 GAAGTCATACCCTCAGACAAGTAGAAACCAGCATCCAGCAAAGCCCAAATGCTTTCTTTC
NC_000001.11:33 ------------------------------------------------------------
NC_000023.11:15 ------------------------------------------------------------
NC_000004.12:c1 ------------------------------------------------------------


NC_000013.11:c3 CCCTCCTTTTCTTTATTACTAAAGGAATCTGACTGATAGTTTTACTTCTCTATACCACCA
NC_000001.11:33 --------------------------------------------------GACAACACCA
NC_000023.11:15 --------------------------------------------------GGTGGGACAG
NC_000004.12:c1 ------------------------------------------------------------


NC_000013.11:c3 AAAAATTAAAATAAAACAATAAAAATTACTTTGTATTCCTGTCAGTCGGATGGAAACACA
```

```
NC_000001.11:33 AAAGGTTGA---------------------------------------------------
NC_000023.11:15 GGACGATGA---------------------------------------------------
NC_000004.12:c1 ------------------------------------------------------------

NC_000013.11:c3 GATTTCATGGAGCTGTGTATTATTGAAGAAACCCGGTGTCTGGGATGAAATTATTTTCAA
NC_000001.11:33 ------------------------------------------------------------
NC_000023.11:15 ------------------------------------------------------------
NC_000004.12:c1 ------------------------------------------------------------

NC_000013.11:c3 CAACGTCCTGTAAGACGCTTTCTTTAACAAACAGAAATGAAAAGCATGGAGTGTTTGAAT
NC_000001.11:33 ------------------------------------------------------------
NC_000023.11:15 ------------------------------------------------------------
NC_000004.12:c1 ------------------------------------------------------------

NC_000013.11:c3 GGAAGACTTGTGACCTGCAGCTGGAACTATGATCTTCCACATTCACCTTTGTGTGTCTTT
NC_000001.11:33 ------------------------------------------------------------
NC_000023.11:15 ------------------------------------------------------------
NC_000004.12:c1 ------------------------------------------------------------

NC_000013.11:c3 AGGGGCTCATTGGAATTTCCTGCTCTGGGCAGGGGACTGTCTGTTTGGTCTTAGAAAGTT
NC_000001.11:33 ------------------------------------------------------------
NC_000023.11:15 ------------------------------------------------------------
NC_000004.12:c1 ------------------------------------------------------------

NC_000013.11:c3 TGGGTATAACTGTATTATAGATGATAAAGGAAACTTGATGTTGTGTTAAAAAATAAATTT
NC_000001.11:33 ------------------------------------------------------------
NC_000023.11:15 ------------------------------------------------------------
NC_000004.12:c1 ------------------------------------------------------------

NC_000013.11:c3 TAAAAAAGAGAAGAAGGGAGAAATCACGAAAGAATTAATTCCAGCAATTTTCACCAAACT
NC_000001.11:33 ------------------------------------------------------------
NC_000023.11:15 ------------------------------------------------------------
NC_000004.12:c1 ------------------------------------------------------------

NC_000013.11:c3 GAAGGAAGAATTCAGATTGAAAATGAAAAATGTCTTATGCCGTCCAACAGAACTACATCT
NC_000001.11:33 ------------------------------------------------------------
NC_000023.11:15 ------------------------------------------------------------
NC_000004.12:c1 ------------------------------------------------------------

NC_000013.11:c3 GTTCAACAGAGGGCGTGTACAGAAACATTTATGACAGCCCTATCTCTAGTATCCCCAGAA
NC_000001.11:33 ------------------------------------------------------------
NC_000023.11:15 ------------------------------------------------------------
NC_000004.12:c1 ------------------------------------------------------------

NC_000013.11:c3 TGGAAATAGTCACAAGTCCATCCTCATTAACATTGGATACATCAATTTTATATATATATA
NC_000001.11:33 ------------------------------------------------------------
NC_000023.11:15 ------------------------------------------------------------
NC_000004.12:c1 ------------------------------------------------------------

NC_000013.11:c3 TATATATATATATATATATATATATATATATATGAACAAGAATGAATGAATTGCCACCAC
NC_000001.11:33 ------------------------------------------------------------
NC_000023.11:15 -----------------------------------------------------------C
NC_000004.12:c1 ------------------------------------------------------------
```

```
NC_000013.11:c3 ATGCAGCGACGTGGGTGAATCAGACAAAATTAATGGTGAATGAAAGAGACCAGGAGCAAA
NC_000001.11:33 ------------------------------------------------------------
NC_000023.11:15 ATGCCGCAGC--------------------------------------------------
NC_000004.12:c1 ------------------------------------------------------------

NC_000013.11:c3 AGAGTACATTTTTTTTAAAAAATACACATTTTACAATGATTTCATTTCTATAAAAATTCA
NC_000001.11:33 ------------------------------------------------------------
NC_000023.11:15 ------------------------------------------------------------
NC_000004.12:c1 ------------------------------------------------------------

NC_000013.11:c3 AAAACAGGCAATCTGATCTATGGTACTGGAAATTACAATAGCAGTTCTCACGAGGAGCAG
NC_000001.11:33 ------------------------------------------------------------
NC_000023.11:15 ------------------------------------------------------------
NC_000004.12:c1 ------------------------------------------------------------

NC_000013.11:c3 GTAGTGGCTCTTGGTAGCACTGTTTCTTAATCTAGGTATTAGTTACACAAAATGTTTGTT
NC_000001.11:33 ------------------------------------------------------------
NC_000023.11:15 ------------------------------------------------------------
NC_000004.12:c1 ------------------------------------------------------------

NC_000013.11:c3 CATTTTGTAAAATGATTTGTGCTCTTCTGTATGTTATACTTCAGTAAAAACAACGTTTTT
NC_000001.11:33 ------------------------------------------------------------
NC_000023.11:15 ------------------------------------------------------------
NC_000004.12:c1 ------------------------------------------------------------

NC_000013.11:c3 CTAAGTTCCATATCAAAACACACCATAGTGAAATTTATGGGGATAGAGAGGAGATGCAAA
NC_000001.11:33 ------------------------------------------------------------
NC_000023.11:15 ------------------------------------------------------------
NC_000004.12:c1 ------------------------------------------------------------

NC_000013.11:c3 AAGCTTTGGTTGTGGCTGGGTGACGGTGGGGAGATAAAAGGATCAAGACAGAATAACTTA
NC_000001.11:33 ------------------------------------------------------------
NC_000023.11:15 ------------------------------------------------------------
NC_000004.12:c1 ------------------------------------------------------------

NC_000013.11:c3 AGACTTCTTAAAAGTATTACTGGGAACTAAACAAAAAAGGAGAAATGCCTGTAACATTCT
NC_000001.11:33 ------------------------------------------------------------
NC_000023.11:15 ------------------------------------------------------------
NC_000004.12:c1 ------------------------------------------------------------

NC_000013.11:c3 AAGGGGAAATTATGTCCAAACTGTCAATCAACTGAGAAGGTAAAATAAAGACATTTCATA
NC_000001.11:33 ------------------------------------------------------------
NC_000023.11:15 ------------------------------------------------------------
NC_000004.12:c1 ------------------------------------------------------------

NC_000013.11:c3 AAACATAGTCTCAAAAAATTTTCTGCCTGTGCATCCTTTCTCAGGAAGTTTCTAGAAGAC
NC_000001.11:33 ------------------------------------------------------------
NC_000023.11:15 -----------------------------TGCATTTTTATTTGGAAACTTTGGAGAAAGT
NC_000004.12:c1 ------------------------------------------------------------

NC_000013.11:c3 ATCACAAATATACGAACAGGGAAGACAGAGGACCCATGGAACGGAGAAGAAGGGAAGAGA
NC_000001.11:33 ------------------------------------------------------------
NC_000023.11:15 G-----------------------------------------------------------
```

```
NC_000004.12:c1 ------------------------------------------------------------


NC_000013.11:c3 ATTTCCAAAATGATGGTTGGAAGCAGCTGTAGAGCTGCGGGCTTGGAGATGAATCCAGGC
NC_000001.11:33 ------------------------------------------------------------
NC_000023.11:15 ------------------------------------------------------------
NC_000004.12:c1 ------------------------------------------------------------


NC_000013.11:c3 CCTGTGGAGCAGGATCGAGGATGCTGCCAAGAAGTCAGTAGCCCAAGTGGTACGAGAGGA
NC_000001.11:33 ------------------------------------------------------------
NC_000023.11:15 ------------------------------------------------------------
NC_000004.12:c1 ------------------------------------------------------------


NC_000013.11:c3 GTTTTAAAGTTCTGTTGGAGAGTATGGTGACAAATAGCGATAGGAACCTAGAAAGCTAAG
NC_000001.11:33 ------------------------------------------------------------
NC_000023.11:15 ------------------------------------------------------------
NC_000004.12:c1 ------------------------------------------------------------


NC_000013.11:c3 CAAATGGAAACAAAAAAGATAATTACTAGCTACCGGAAAACAAAAAGATGTCAAGAAAGG
NC_000001.11:33 ------------------------------------------------------------
NC_000023.11:15 ------------------------------------------------------------
NC_000004.12:c1 ------------------------------------------------------------


NC_000013.11:c3 AAATGCAATGAGGGTCCACTACAGTGAACAATATTTACACAGTTGTTGAATAATATGAAT
NC_000001.11:33 ------------------------------------------------------------
NC_000023.11:15 ------------------------------------------------------------
NC_000004.12:c1 ------------------------------------------------------------


NC_000013.11:c3 GTTAATTTAATTTTTAGGCCGGGCATGGTGGCTCACGCCTGTAATCCCAGCACTTTGGGA
NC_000001.11:33 ------------------------------------------------------------
NC_000023.11:15 -----------TTCTCGGCTGGAC------------------------------------
NC_000004.12:c1 ------------------------------------------------------------


NC_000013.11:c3 GGCTGAGGTGGGCAGATCACCTGAGGTCAGGAGTTCGAGACCAGCCTGGCCAACATGGTG
NC_000001.11:33 ------------------------------------------------------------
NC_000023.11:15 ------------------------------------------------------------
NC_000004.12:c1 ------------------------------------------------------------


NC_000013.11:c3 AAAGCCTGTCTCCACTAAAAATACAAAAATTAGCCGGGTGTGGTGGCATGTGCCTATAGT
NC_000001.11:33 ------------------------------------------------------------
NC_000023.11:15 ------------------------------------------------------------
NC_000004.12:c1 ------------------------------------------------------------


NC_000013.11:c3 CCCAGCTACTCTCAAGGCTGAAGCAGGAGAATCACTTGAACCCAGGAGACAGAGCTTGCA
NC_000001.11:33 ------------------------------------------------------------
NC_000023.11:15 ------------------------------------------------------------
NC_000004.12:c1 ------------------------------------------------------------


NC_000013.11:c3 GTGAGCCGAGATCGCACCACTGCACTCCAGTTCAAAAACAAAATTAGTTTTTAAAAATAT
NC_000001.11:33 ------------------------------------------------------------
NC_000023.11:15 -------------------CTGCATTCTGGGTTACAGATGAG------------------
NC_000004.12:c1 ------------------------------------------------------------


NC_000013.11:c3 ACATATTGGGAAATTAGGAGAGGAGAAGGGGGAAAGTACATTGCATTGTGAAGCAAAAGT
```

```
NC_000001.11:33 ------------------------------------------------------------
NC_000023.11:15 ------------------------------------------------------------
NC_000004.12:c1 ------------------------------------------------------------


NC_000013.11:c3 TAAAAAAAAAGTCTGAATCCTAATACCCTATGATAGGAAGTCAATATTATGTTTTATATA
NC_000001.11:33 ------------------------------------------------------------
NC_000023.11:15 ------------------------------------------------------------
NC_000004.12:c1 ------------------------------------------------------------


NC_000013.11:c3 CTATGCATATATGTTGTTTTGATAATTTTAAAAACACTATGAGGAGAAAAATAATTGGTT
NC_000001.11:33 ------------------------------------------------------------
NC_000023.11:15 ------------------------------------------------------------
NC_000004.12:c1 ------------------------------------------------------------


NC_000013.11:c3 AATCTGCAACACAGAATTGTTTATTGCAGTAACTTCTATTTAATTTGAGAAAATAAAACA
NC_000001.11:33 ------------------------------------------------------------
NC_000023.11:15 ------------------------------------------------------------
NC_000004.12:c1 ------------------------------------------------------------


NC_000013.11:c3 TTTTTCTATCCCTTCTGAAGGAAACCTTTAAGTACACTTTAATTTTTTATCTAGTTCTGT
NC_000001.11:33 ------------------------------------------------------------
NC_000023.11:15 ------------------------------------------------------------
NC_000004.12:c1 ------------------------------------------------------------


NC_000013.11:c3 AGTGTACACGGTAAGAAAATCAAATTTAAAATATGCTTCCATTTTCTGAATTACGTTATT
NC_000001.11:33 ------------------------------------------------------------
NC_000023.11:15 ------------------------------------------------------------
NC_000004.12:c1 ------------------------------------------------------------


NC_000013.11:c3 GGTAATAATTACAAAAATGGACTTAGATTACTTGAGTGTGTTGCTTAGCTTTTGCCTATA
NC_000001.11:33 -------------------------------------------CATAGCTTTTCCCT---
NC_000023.11:15 -------------------------------------------CACGACTAGCCCTT---
NC_000004.12:c1 -------------------------------------------GCCAGCGCCCGCCC---
                                                             ...*   . *..

NC_000013.11:c3 TTCCTGTCTTAATCGTGAGCAGAAAATAGGTTTATTTTTGTTTCAGTTTTTGTTTTGAGA
NC_000001.11:33 ------------------------------------------------------------
NC_000023.11:15 ------------------------------------------------------------
NC_000004.12:c1 ------------------------------------------------------------


NC_000013.11:c3 CAGAGTTTTGCTCTTGTCACCAGGCTGGAGTGCAGTGGCACAATCTCAACTCCCTGCAAC
NC_000001.11:33 ------------------------------------------------------------
NC_000023.11:15 ------------------------------------------------------------
NC_000004.12:c1 ------------------------------------------------------------


NC_000013.11:c3 CTCTGCCTCCTGGGTTCAAGTGATTCTCCTGCCTCAGCCTCCCAAGTAGCTGGGATAACA
NC_000001.11:33 ------------------------TTTACTGCCTTTTCTTCTTAAAT-------------
NC_000023.11:15 -----------------------TTTATACACCCTCCCCCTTTAGATAG-----------
NC_000004.12:c1 ------------------------------------------------------------


NC_000013.11:c3 GGCATGTGGCACCACGCCTGGCTAATTTTTGTATTTTTAGTAGAGATGTGGTTTCGCCAT
NC_000001.11:33 ------------------------------------------------------------
NC_000023.11:15 ------------------------------------------------------------
NC_000004.12:c1 ------------------------------------------------------------
```

```
NC_000013.11:c3 GTTGGCCAGGCTGGTTTCGAACTCCTGACCTTAGGTGATCTGCCCTCCTCAGCTTCCCAA
NC_000001.11:33 ------------------------------------------------------------
NC_000023.11:15 ------------------------------------------------------------
NC_000004.12:c1 ------------------------------------------------------------

NC_000013.11:c3 AGTGCTGGGATTACAGGCGTGAGTCCCTGTGCCCTGCAGGTTTTTTAAAAGTGCACATTA
NC_000001.11:33 ------------------------------------------------------------
NC_000023.11:15 ------------------------------------------------------------
NC_000004.12:c1 ------------------------------------------------------------

NC_000013.11:c3 ATTCAAATAAGTGGAATGGTAAGATACAATAAGCATGGTATCTTTGCAGTTTTCCTGCTG
NC_000001.11:33 ------------------------------------------------------------
NC_000023.11:15 -------------------------------------------------------TGACG
NC_000004.12:c1 ------------------------------------------------------------

NC_000013.11:c3 AAGCAATTAATCCTAGAAACTTCCAGAATTTCAAATGGTAAAGAGAAGCATGGAGGAAAA
NC_000001.11:33 ------------------------------------------------------------
NC_000023.11:15 AAGTGATGAAT-------------------------------------------------
NC_000004.12:c1 ------------------------------------------------------------

NC_000013.11:c3 GATGTTCACCTTTTTTGTGAGTTACTAGTATCAATCATCTTTCCAGCTTAGAGAAAGTAG
NC_000001.11:33 ------------------------------------------------------------
NC_000023.11:15 -----------------TGAACTAATAGTA------------------------------
NC_000004.12:c1 -------------------------TGGCA------------------------------

NC_000013.11:c3 CAATACATACATATGCAAACACAGTTTTTTCCCCAAAATATTGAATTTCAGAAGTTCTCA
NC_000001.11:33 ------------------------------------------------------------
NC_000023.11:15 ------------------------------------------------CGGGGGTAC---
NC_000004.12:c1 ------------------------------------------------------------

NC_000013.11:c3 ATCATTCTCATGAAAACTATTTTTGTTGTTTCCAAATCTTGGCAAGAAACTTGAGAATGA
NC_000001.11:33 ------------------------------------------------------------
NC_000023.11:15 ------------------------------------------------------------
NC_000004.12:c1 ------------------------------------------------------------

NC_000013.11:c3 ACTAAGCAAACACTGAAGTTCCCTCCTTTTCAAAGTACTGGCAAAGCACAGCTTCGGATG
NC_000001.11:33 ------------------------------------------------------------
NC_000023.11:15 ------------------------------------------------------------
NC_000004.12:c1 ------------------------------------------------------------

NC_000013.11:c3 AACTCCATCAAGTGCTGCTGGCTGGCTTGTCAAGACAGAGGCTACAATTCTGTGATAGGG
NC_000001.11:33 --------------CTGCTAGCT-------------------------------------
NC_000023.11:15 --------------CTGCTCACT-------------------------------------
NC_000004.12:c1 -------------GCTGCGGGCT-------------------------------------
                              ****  .**

NC_000013.11:c3 CAGAAAGAGAAAGGCTGAGTGGGGAAGGTTGTACAGAGGACCCTCCTTATCCAAAGATTC
NC_000001.11:33 ------------------------------------------------------------
NC_000023.11:15 ------------------------------------------------TTCCAGA-----
NC_000004.12:c1 ------------------------------------------------------------

NC_000013.11:c3 ATTATGAGAGTCCCTGCAGAAAAGATTAACAAATAATCTAATACCGACTTGTGAAATTAG
NC_000001.11:33 ------------------------------------------------------------
NC_000023.11:15 ------------------------------------------------------------
```

```
NC_000004.12:c1 ------------------------------------------------------------

NC_000013.11:c3 TCCTCATTCAAGCATCTCTTGCCTCTTAGCATCTGTAAGTCATTGAACAGTCTCTCCAGT
NC_000001.11:33 ------------------------------------------------------------
NC_000023.11:15 ----------------------------------------------------TCTCCCGT
NC_000004.12:c1 ------------------------------------------------------------

NC_000013.11:c3 GCTGTATACATTAAAGCACAGCAAATGGATAGCACAGTGAAAATGCTAATCTAGAAGGTG
NC_000001.11:33 ------------------------------------------------------------
NC_000023.11:15 GTTGTAAA----------------------------------------------------
NC_000004.12:c1 ------------------------------------------------------------

NC_000013.11:c3 GAAAATGTCACGGAGCCTCTGAAAGTGATGTTAGAAAACAGCTTGAACTGGCTGGGCATG
NC_000001.11:33 ------------------------------------------------------------
NC_000023.11:15 ------------------------------------------------------------
NC_000004.12:c1 ------------------------------------------------------------

NC_000013.11:c3 GTGGCTCAAACCTGTAATCCTAGCACTTTGGGAGGCCAGGGCGGGCAGATCACCTGAGGC
NC_000001.11:33 ------------------------------------------------------------
NC_000023.11:15 ------------------------------------------------------------
NC_000004.12:c1 ------------------------------------------------------------

NC_000013.11:c3 CAGGAGTTCAAGACCAGCCTAGCCAACATGGTGAAACCCCATCTCTACTAAAAGTACAAA
NC_000001.11:33 ------------------------------------------------------------
NC_000023.11:15 ------------------------------------------------------------
NC_000004.12:c1 ------------------------------------------------------------

NC_000013.11:c3 AGTTAACTGATGTGGTGGCACATGCCCATAATCCCAGCTGCTCGGGAGGCTGAGGCAGGA
NC_000001.11:33 ------------------------------------------------------------
NC_000023.11:15 ------------------------------------------------------------
NC_000004.12:c1 ------------------------------------------------------------

NC_000013.11:c3 GAATAGTTTGAACCTGGAGGCGGAGGTTGCAATGAGCAAAGATGGTGTCAAAAAAAAAAA
NC_000001.11:33 ------------------------------------------------------------
NC_000023.11:15 ------------------------------------------------------------
NC_000004.12:c1 ------------------------------------------------------------

NC_000013.11:c3 AAAAAAAAAGCAGCTTGAACCATAGGCTAGAGTTAAACTAATTAACAACATAGAAAAAAA
NC_000001.11:33 ------------------------------------------------------------
NC_000023.11:15 ------------------------------------------------------------
NC_000004.12:c1 ------------------------------------------------------------

NC_000013.11:c3 GAGTAAGATAAGGATGATAACATAATAGGTGCTTCCAAAAAGAATGACAAGAATATTTAA
NC_000001.11:33 ------------------------------------------------------------
NC_000023.11:15 ------------------------------------------------------------
NC_000004.12:c1 ------------------------------------------------------------

NC_000013.11:c3 AAATTTAAGGGCAGTCCTTGGAAAGTTGACAAGGCTCTCCTTAAATATGTGTGGGGAAAA
NC_000001.11:33 ------------------------------------------------------------
NC_000023.11:15 ------------------------------------------------------------
NC_000004.12:c1 ------------------------------------------------------------

NC_000013.11:c3 AAAAATGACCATTTTAATGATCAAGCTATACAAGTCCAACGTGAAGGGTATTATATTGTG
```

```
NC_000001.11:33 ------------------------------------------------------------
NC_000023.11:15 ------------------------------------------------------------
NC_000004.12:c1 ------------------------------------------------------------


NC_000013.11:c3 CTCGCATCCATTTTTGTAGAGCAGAGCTTTCGCCCAAAGAAGGGAGCCATGGGAGGCTAA
NC_000001.11:33 ------------------------------------------------------------
NC_000023.11:15 ------------------------------------------------------------
NC_000004.12:c1 ------------------------------------------------------------


NC_000013.11:c3 AGTGAGAGACGAGACTGAGTTTGGCCTGGGCCGTGGAGTGTGACTGAACTGGCTGAGTGA
NC_000001.11:33 ------------------------------------------------------------
NC_000023.11:15 ------------------------------------------------------------
NC_000004.12:c1 ------------------------------------------------------------


NC_000013.11:c3 GCAGGGGAAATGGGACCAGCTCAGGAAGGAACTTGAATCAGAATGAGGAATTTGGACGGT
NC_000001.11:33 ------------------------------------------------------------
NC_000023.11:15 ----------------------------------------------------------GC
NC_000004.12:c1 ------------------------------------------------------------


NC_000013.11:c3 CATCTTTTTCACATGTTCACCAACCTAGGGAGATAAAGATAGTCTTAGGGCCAGGCGCGG
NC_000001.11:33 ------------------------------------------------------------
NC_000023.11:15 CGTTTTTTT---------------------------------------------------
NC_000004.12:c1 ------------------------------------------------------------


NC_000013.11:c3 TGGCTCACGCATATAATCCCAGCACTTTGGGAGGCAGGCAGATCACCTGAGGTTGGGAGT
NC_000001.11:33 ------------------------------------------------------------
NC_000023.11:15 ------------------------------------------------------------
NC_000004.12:c1 ------------------------------------------------------------


NC_000013.11:c3 TCAAGACCAGCCTTACCAACATGGAGAAACCCTGTCTCCACTAAAAATACAAAATTAGCC
NC_000001.11:33 ------------------------------------------------------------
NC_000023.11:15 ------------------------------------------------------------
NC_000004.12:c1 ------------------------------------------------------------


NC_000013.11:c3 GGGTGTGGTGGTGCATGCCTGTAATCCCAGCTACTTGGGAGGCTAAGGCAGGAGAATTGC
NC_000001.11:33 ------------------------------------------------------------
NC_000023.11:15 ------------------------------------------------------------
NC_000004.12:c1 ------------------------------------------------------------


NC_000013.11:c3 TTGAACCTGGGAGGCGGAGGTTGCGGTGAGCCGAGATCACACCATTGCACTCCAGCCCGG
NC_000001.11:33 ------------------------------------------------------------
NC_000023.11:15 ------------------------AGTAAGCCAAAA-CACAT------------------
NC_000004.12:c1 ------------------------------------------------------------


NC_000013.11:c3 GCAACAAGAGCGAAACTCCATCTTAAAAAAAAAAAAAAAGATAGTCTTTACCAGGATTTA
NC_000001.11:33 ------------------------------------------------------------
NC_000023.11:15 -CAACAATAGTGACAAT-------------------------------------------
NC_000004.12:c1 ------------------------------------------------------------


NC_000013.11:c3 CTACAGAATTTTCAAATTGGGGATTTTTTTTTAAATTATGTAAGGGAAAAATTTACAATA
NC_000001.11:33 ------------------------------------------------------------
NC_000023.11:15 ------------------------------------------------------------
NC_000004.12:c1 ------------------------------------------------------------
```

```
NC_000013.11:c3 CATTGCAATTAGCTAATAGAATTATTATTTTATGACACTTCTGTTTTAGTTTTATACATG
NC_000001.11:33 ------------------------------------------------------------
NC_000023.11:15 ------------------------------------------------------------
NC_000004.12:c1 ------------------------------------------------------------

NC_000013.11:c3 TGTATACCTGTGTCACAAGGCAAATATTGGTCTTACTGAGAGTCATGTAATCCAAAATTT
NC_000001.11:33 ------------------------------------------------------------
NC_000023.11:15 ------------------------------------------------------------
NC_000004.12:c1 ------------------------------------------------------------

NC_000013.11:c3 GGGCCGGGCACAGTGGCTCACACCTGTAATCCTAGAACTTCAGGAGGCCGAGGCCGGTGG
NC_000001.11:33 ------------------------------------------------------------
NC_000023.11:15 ------------------------------------------------------------
NC_000004.12:c1 ------------------------------------------------------------

NC_000013.11:c3 ATCACTCGAGGTCAGGAGTTTGAGATCAGCCTGGCTAACATGGTGAAATCCCGTCTCCAC
NC_000001.11:33 ------------------------------------------------------------
NC_000023.11:15 ------------------------------------------------------------
NC_000004.12:c1 ------------------------------------------------------------

NC_000013.11:c3 TAAAAATACAAAAGTTAGCCAGGCCTGGTGGCACACACCTGTAATCCCAGCTACTTGGGA
NC_000001.11:33 ------------------------------------------------------------
NC_000023.11:15 ------------------------------------------------------------
NC_000004.12:c1 ------------------------------------------------------------

NC_000013.11:c3 GGCTAAGGCAGGAGAATCACTTGAACCCAGGAGGTGAAGGTTGCAATGACCCAAGATCAC
NC_000001.11:33 ------------------------------------------------------------
NC_000023.11:15 ------------------------------------------------------------
NC_000004.12:c1 ------------------------------------------------------------

NC_000013.11:c3 ACCTCTGCACTCTGGCCTGGGTGACAGAGTGAGACTCTGTCTCAAAAACAACAAAAAACA
NC_000001.11:33 ------------------------------------------------------------
NC_000023.11:15 ------------------------------------------------------------
NC_000004.12:c1 ------------------------------------------------------------

NC_000013.11:c3 AAGTTTGAAAGACACTGATATAGAGGAAGCGTTATGGTTCTGTTATAGATGAGAACTCCA
NC_000001.11:33 ------------------------------------------------------------
NC_000023.11:15 ------------------------------------------------------------
NC_000004.12:c1 ------------------------------------------------------------

NC_000013.11:c3 GAAGCCCTAACTGAGCCACATTGGAGAACAGCACCAGACTGTGAAGTCTCTGGGCTGTGG
NC_000001.11:33 ------------------------------------------------------------
NC_000023.11:15 ------------------------------------------------------------
NC_000004.12:c1 ------------------------------------------------------------

NC_000013.11:c3 TTTCACAAATGTCCCCAGGCTCCAATCCCAGTGCCTCCCTGCATGGTACATTGGCTCCAG
NC_000001.11:33 ------------------------------------------------------------
NC_000023.11:15 ------------------------------------------------------------
NC_000004.12:c1 ------------------------------------------------------------

NC_000013.11:c3 GGCCCTTTGCTTGGCCCCACTGCCCACTGCTGCTGAATCCTTCTGTCTCCCCTGCTGCAT
NC_000001.11:33 --------------TCCCGATAGCTATTGTGGGTTAATCTT-------------------
NC_000023.11:15 --------------TTCAGATGTCCCCTACGTATAGTTCTC-CTGCTT------------
```

```
NC_000004.12:c1 --------------CCGCGCCGACCC-----------------------------------
                              ..   . .. *.

NC_000013.11:c3 GTGAATCTGGGTCTCATGGTGGAAAAAATACGTGTTTTGGGCTCAGCTGGACCTGGGTGT
NC_000001.11:33 -----CCTGGATCTTATCATGAGAGGGAT-------------------------------
NC_000023.11:15 -TGGTCTTGTGTTCAGCAGTGGTAAGAGT-------------------------------
NC_000004.12:c1 ------------------------------------------------------------


NC_000013.11:c3 GAAGCTTAGTTCAGCCCCTTACTTTACAGGTGAAGTTATGAAGCTATTGTGATACTCTAT
NC_000001.11:33 ------------------------------------------------------------
NC_000023.11:15 ------------------------------------------------------------
NC_000004.12:c1 ------------------------------------------------------------


NC_000013.11:c3 TGCCTCTTTTGTAAAAGTGGGGGAAATAACACCTATTTCATAGGGTTGTAATGAGGATTT
NC_000001.11:33 ------------------------------------------------------------
NC_000023.11:15 ------------------------------------------------------------
NC_000004.12:c1 ------------------------------------------------------------


NC_000013.11:c3 AAAGAATATATGTCACCTCCAATCAAGCTGTATTCTACTTCTAGGAGCTCATCTGTGGAA
NC_000001.11:33 ------------------------------------------------------------
NC_000023.11:15 ----------TGTTGTTTTTAATTGTGTT-------------------------------
NC_000004.12:c1 ------------------------------------------------------------


NC_000013.11:c3 GTACTCATGTGCACAGATTCACATGCATGGATGGTTGATTGCAGTCTTATAAAACCAAAC
NC_000001.11:33 ------------------------------------------------------------
NC_000023.11:15 ---------------------------------------------------------AAC
NC_000004.12:c1 ------------------------------------------------------------


NC_000013.11:c3 ATCTGGAAGGAGATTAAAAAGCTAATAAGGGGCAGGCATGGTGGCTCACCCCTGTAATCC
NC_000001.11:33 -------GGAAGGTTAAGAGGC--------------------------------------
NC_000023.11:15 AGTTAGCAGATGACCAGATAGT--------------------------------------
NC_000004.12:c1 ------------------------------------------------------------


NC_000013.11:c3 CAGCCGAGGCAGGTGGATCACTTGAGGTCAGGAGTTTGAGACCAGCCTAGCTAACATGGC
NC_000001.11:33 ------------------------------------------------------------
NC_000023.11:15 ------------------------------------------------------------
NC_000004.12:c1 ------------------------------------------------------------


NC_000013.11:c3 GAAACCCCATCTCTACTAAAAACATAAAAATTAGCTGGGCATGATGGTGCATGCCTGTAG
NC_000001.11:33 ------------------------------------------------------------
NC_000023.11:15 ------------------------------------------------------------
NC_000004.12:c1 ------------------------------------------------------------


NC_000013.11:c3 TCCCAGCTACTCAGGAGGTTGAGGCAGGAGAATCACTTGAATCTGGCAGGCAGAGGTTGC
NC_000001.11:33 ------------------------------------------------------------
NC_000023.11:15 ------------------------------------------------------------
NC_000004.12:c1 ------------------------------------------------------------


NC_000013.11:c3 AGTGAGCTGAGATTGCGCCACTGCACTCCACCCTGGGCGACAGAGTGAGACTCCGTCTCA
NC_000001.11:33 ------------------------------------------------------------
NC_000023.11:15 ------------------------------------------------------------
NC_000004.12:c1 ------------------------------------------------------------


NC_000013.11:c3 AAAAACATATATAAAAAATAAAGGATTGATTTATTTTTAATTATGACATAGCAATATAAT
```

```
NC_000001.11:33 ------------------------------------------------------------
NC_000023.11:15 ------------------------------------------------------------
NC_000004.12:c1 ------------------------------------------------------------


NC_000013.11:c3 GGAATACTATGGAGCTATTTAAAAGAATGAAGTATACCTAGGTCTACTTATATGGAAAGA
NC_000001.11:33 ------------------------------------------------------------
NC_000023.11:15 ------------------------------------------------------------
NC_000004.12:c1 ------------------------------------------------------------


NC_000013.11:c3 TATCTGCGTCACTGCTTAGTGGAAAAAAAACAAGTTGCAAAATCCTGACCCCATTTTTTT
NC_000001.11:33 -------------------------ACAGATAAGCTAC----------------------
NC_000023.11:15 --------------CTTA-------AGTCATAGATTTCAAGCTTCT--------------
NC_000004.12:c1 ------------------------------------------------------------


NC_000013.11:c3 TTTTTTTTTTTGAGACAGAGTCTTGCTCTGTCGCCCAGGCTGGAGTACAGTGGTGAGATC
NC_000001.11:33 ------------------------------------------------------------
NC_000023.11:15 ------------------------------------------------------------
NC_000004.12:c1 ------------------------------------------------------------


NC_000013.11:c3 TCAGCTCACGGCAACCTCCGCCTCCCGGGTTCAAGCGATTCTCCTGCCTCAGCCTCCTGA
NC_000001.11:33 ------------------------------------------------------------
NC_000023.11:15 ------------------------------------------------------------
NC_000004.12:c1 ------------------------------------------------------------


NC_000013.11:c3 GTAGCTGGGATTACAGGTGCACACCACCATGCCGGCTAATTTTTGTACTTTTAGTAGAGA
NC_000001.11:33 ------------------------------------------------------------
NC_000023.11:15 ------------------------------------------------------------
NC_000004.12:c1 ------------------------------------------------------------


NC_000013.11:c3 CGGGTTTCACCATGTTGATCAGGCTGGTGTCGAACTCCTTACCTTGTGATCTGCTCGCCT
NC_000001.11:33 ------------------------------------------------------------
NC_000023.11:15 ------------------------------------------------------------
NC_000004.12:c1 ------------------------------------------------------------


NC_000013.11:c3 CGGCCTCCCAAAGTGCTGGGATTACAGGTGTGAGCCACTGCGCCTGGCCTGATTTTTTAA
NC_000001.11:33 ------------------------------------------------------------
NC_000023.11:15 ------------------------------------------------------------
NC_000004.12:c1 ------------------------------------------------------------


NC_000013.11:c3 AAATACATAGATTATGGACATGCATAAAGAAATCTATAATACCATAAACGTGGCTATCTT
NC_000001.11:33 ------------------------------------------------------------
NC_000023.11:15 ------------------------------------------------------------
NC_000004.12:c1 ------------------------------------------------------------


NC_000013.11:c3 TGGGAAGTAGAATTATGAAGGACTTTAGATTTCTGTTTTGTAAAATGTTTTATTTATTAT
NC_000001.11:33 ------------------------------------------------------------
NC_000023.11:15 ------------------------------------------------------------
NC_000004.12:c1 ------------------------------------------------------------


NC_000013.11:c3 TTATTTTATTTATTATTTATTTATTTATTTTGAGACAGTCTTGCCCTATTGCCCAGGCTG
NC_000001.11:33 ------------------------------------------------------------
NC_000023.11:15 ------------------------------------------------------------
NC_000004.12:c1 ------------------------------------------------------------
```

```
NC_000013.11:c3 GAGTGCAATGGTGAGATCTCGGCTCACTGCAACCTACACCTCCTGGGTTCAAGCCATTCT
NC_000001.11:33 ------------------------------------------------------------
NC_000023.11:15 ------------------------------------------------------------
NC_000004.12:c1 ------------------------------------------------------------

NC_000013.11:c3 CCTGCTTCAGCCTCCCGAGTAGTTGGGATTACAGGTGCCCACCACCACGCCCAGCTAATT
NC_000001.11:33 ------------------------------------------------------------
NC_000023.11:15 -------------------------------------------ATCACCCCCACCAAAT-
NC_000004.12:c1 ------------------------------------------------------------

NC_000013.11:c3 TTTGTATTTTTAGTAGTAGAGTTTCACCATGTTGGTCAAGCTGGTCTTGAACTCCTGACC
NC_000001.11:33 ------------------------------------------------------------
NC_000023.11:15 ------------------------------------------------------------
NC_000004.12:c1 ------------------------------------------------------------

NC_000013.11:c3 TCAGGTGATCCGCCCACCTCGGCCTCCCAAAGTGCTGGGATTACAGGCGTGAGCCACCGC
NC_000001.11:33 ------------------------------------------------------------
NC_000023.11:15 ------------------------------------------------------------
NC_000004.12:c1 ------------------------------------------------------------

NC_000013.11:c3 ACCAGCTGTAAAATATTTTACAATGAGCATGTGTGACTTTCATATTCAGGAAAAAAATTT
NC_000001.11:33 ------------------------------------------------------------
NC_000023.11:15 ------------------------------------------------------------
NC_000004.12:c1 ------------------------------------------------------------

NC_000013.11:c3 CGCTGAAAACACACACACACACACACACACACACACACACACACACACACACACACGTAT
NC_000001.11:33 ------------------------------------------------------------
NC_000023.11:15 ------------------------------------------------------------
NC_000004.12:c1 ------------------------------------------------------------

NC_000013.11:c3 ATGTTACCTAGCATAGCAGTTCCGAGTAGTTAGTGGTTGTTATGAGAGTTCCACTTCCAG
NC_000001.11:33 ------------------------------------------------------------
NC_000023.11:15 ------------------------------------------------------------
NC_000004.12:c1 ------------------------------------------------------------

NC_000013.11:c3 CGTGGGTGCGCTACATCTTTAAATATTCCTGTAGCAATGAATAGCCACTGTGCCTAGGGT
NC_000001.11:33 ------------------------------------------------------------
NC_000023.11:15 ------------------------------------------------------------
NC_000004.12:c1 ------------------------------------------------------------

NC_000013.11:c3 TTCTGTGTGAATACATACCCTCTTTTTTTATTCTTGATAGATTCATTATTTCAGTTGAGG
NC_000001.11:33 ------------------------------------------------------------
NC_000023.11:15 --------------GTGTCCTC--------------------------------------
NC_000004.12:c1 ------------------------------------------------------------

NC_000013.11:c3 CCTTGTTCTCTTTTCTTTCATTAGCTTTAATCAGCAGGCTCGGGGCACCTGTCACAGGGG
NC_000001.11:33 ------------------------------------------------------------
NC_000023.11:15 ------------------------------------------------------------
NC_000004.12:c1 ------------------------------------------------------------

NC_000013.11:c3 ATGCCATCATTCATTTTAATTTTTAAAATTATAGTAGCAATTTAAGCTCATCCTAAACAT
NC_000001.11:33 ------------------------------------------------------------
NC_000023.11:15 ------------------------------------------------------------
```

```
NC_000004.12:c1 ------------------------------------------------------------


NC_000013.11:c3 GAAAAACAAATCTGCAGTTAGTAAAGTAAAACCTTAAAGTCATCAATTTCACCATGCCTT
NC_000001.11:33 ------------------------------------------------------------
NC_000023.11:15 ------------------------------------------------------------
NC_000004.12:c1 ------------------------------------------------------------


NC_000013.11:c3 TCAGTCCCACTACCCAAAGATGGCAGTGGTTCAGTGTATGGTCTTCACAGCTTCTGTGCA
NC_000001.11:33 ----------------------------------------------------TCTGTGTG
NC_000023.11:15 ----------------------------------------------------TCTCTACC
NC_000004.12:c1 ------------------------------------------------------------


NC_000013.11:c3 TATGCAAATGTGTGTGTGCACTTGTTTTTTATTTGTCTTTTGAGACAGGGTCTCACTCTT
NC_000001.11:33 TGTGTGTGTGTGTGTGTGT-----------------------------------------
NC_000023.11:15 CTTCCCACTTTGTGATTGT-----------------------------------------
NC_000004.12:c1 ------------------------------------------------------------


NC_000013.11:c3 GCCCGGTCTGGAGTGCAGTGGCACAATATCAGCTCACTGCAGCCTCGACCTTCTGGCCTC
NC_000001.11:33 ------------------------------------------------------------
NC_000023.11:15 ------------------------------------------------------------
NC_000004.12:c1 ------------------------------------------------------------


NC_000013.11:c3 AGGTGACCTTCTCACCTCAGCCTTCTGAGTAGCTGGGACTACAAACATGTGCCACCACAC
NC_000001.11:33 ------------------------------------------------------------
NC_000023.11:15 ------------------------------------------------------------
NC_000004.12:c1 ------------------------------------------------------------


NC_000013.11:c3 CCAGCTAATTTTTGTATTTTTTGTAGAGACAGGATTTCGTTATGTTGGCCAGGCTGGTCT
NC_000001.11:33 ------------------------------------------------------------
NC_000023.11:15 ------------------------------------------------------------
NC_000004.12:c1 ------------------------------------------------------------


NC_000013.11:c3 TGAACTCCTGGGCTCAAGCAATCTGCCTGCCTCCACCTCCCAAAGTGCTAGGATTACAGG
NC_000001.11:33 ------------------------------------------------------------
NC_000023.11:15 ------------------------------------------------------------
NC_000004.12:c1 ------------------------------------------------------------


NC_000013.11:c3 CATGAGCCACCATGCCCAGCTTGCATTTGTGTTCTTGTTGCACCAATAAGATCATGAAGC
NC_000001.11:33 ------------------------------------------------------------
NC_000023.11:15 ------------------------------------------------------------
NC_000004.12:c1 ------------------------------------------------------------


NC_000013.11:c3 ATATAAAAAAAGTCTTTCCACAACAGAGATTTATATATACTATGTCTTTTTTAAGAACTG
NC_000001.11:33 ------------------------------------------------------------
NC_000023.11:15 ------------------------------------------------------------
NC_000004.12:c1 ------------------------------------------------------------


NC_000013.11:c3 CATAATATTCTACTTTGTAGATGTAATTTATTTCACCATTCCCTTATTGACAGAATTTTC
NC_000001.11:33 ------------------------------------------------------------
NC_000023.11:15 ------------------------------------------------------------
NC_000004.12:c1 ------------------------------------------------------------


NC_000013.11:c3 ACTATTGTTAGCAGAGCTACAAAAATACTTTTACCTATATATTTGCACACTTCAGTGAGC
```

```
NC_000001.11:33 ------------------------------------------------------------
NC_000023.11:15 ------------------------------------------------------------
NC_000004.12:c1 ------------------------------------------------------------

NC_000013.11:c3 AGATCTGTAAGATAAATTCCTACAGGGGAACTAGTATTGGTCAAAGGGTATCCATGTGGA
NC_000001.11:33 ------------------------------------------------------------
NC_000023.11:15 ------------------------------------------------------------
NC_000004.12:c1 ------------------------------------------------------------

NC_000013.11:c3 TAATTGTTATCATGTTGCACTGACTTACATTCCCAGCAGTACATGTATGAGTTCCTTTTC
NC_000001.11:33 ------------------------------------------------------------
NC_000023.11:15 ------------------------------------------------------------
NC_000004.12:c1 ------------------------------------------------------------

NC_000013.11:c3 CCCTACACACTTTTACCTGCACAAGTTGAGTATTCTTTACACAAAATGCTCAGGACCAGA
NC_000001.11:33 ------------------------------------------------------------
NC_000023.11:15 ------------------------------------------------------------
NC_000004.12:c1 ------------------------------------------------------------

NC_000013.11:c3 CATGTTTCAGACTTCAGATATTTTAGGATTTTGGAATATTTGCATATACATAATGAGATA
NC_000001.11:33 ------------------------------------------------------------
NC_000023.11:15 ------------------------------------------------------------
NC_000004.12:c1 ------------------------------------------------------------

NC_000013.11:c3 TCGTGGGCATGGGACCCAAATCTAAACATGAAATTCATTGATGTTTCATATACACTCTAT
NC_000001.11:33 ------------------------------------------------------------
NC_000023.11:15 ------------------------------------------------------------
NC_000004.12:c1 ------------------------------------------------------------

NC_000013.11:c3 ACAGGTAGCCTGAAGGTAATTTTATGCAACATTCTTAGTAATTTTTGTGACCCATCACAA
NC_000001.11:33 ------------------------------------------------------------
NC_000023.11:15 ------------------------------------------------------------
NC_000004.12:c1 ------------------------------------------------------------

NC_000013.11:c3 GAAGTCAGGTGTTTTAGAAATTTTAGAAAATTTTGGATTTTGGGTTTTCAGATGAGGGAT
NC_000001.11:33 ------------------------------------------------------------
NC_000023.11:15 ------------------------------------------------------------
NC_000004.12:c1 ------------------------------------------------------------

NC_000013.11:c3 GCTCAACTGTATTGTGTATTATATATTATTGTCTTATTATTGCTTTTATTTGTTATTATT
NC_000001.11:33 ------------------------------------------------------------
NC_000023.11:15 ------------------------------------------------------------
NC_000004.12:c1 ------------------------------------------------------------

NC_000013.11:c3 TTTAAATCTTTGGTAATTCAGTATGTGAAATAATGGGTGTCATTGTTGAATTTGAATTTT
NC_000001.11:33 ------------------------------------------------------------
NC_000023.11:15 ------------------------------------------------------------
NC_000004.12:c1 ------------------------------------------------------------

NC_000013.11:c3 TTTCATCAGACTGAAGACATCATTTCTTTATTGCCACCATCCATTTGGAAATGTTATGCA
NC_000001.11:33 ------------------------------------------------------------
NC_000023.11:15 ------------------------------------------------------------
NC_000004.12:c1 ------------------------------------------------------------
```

```
NC_000013.11:c3 CATTCTGCAAATCCAATTGGAAGTCTGGCCTAGAGTTTGGTTAGATGGTGGCAGGGAAAT
NC_000001.11:33 ------------------------------------------------------------
NC_000023.11:15 ------------------------------------------------------------
NC_000004.12:c1 ------------------------------------------------------------

NC_000013.11:c3 AATATGCTTAGAAACGTGGTCCTGGATAGAATGTTGAGGAAAAGCAGAGAGAGAAGATGT
NC_000001.11:33 ------------------------------------------------------------
NC_000023.11:15 ------------------------------------------------------------
NC_000004.12:c1 ------------------------------------------------------------

NC_000013.11:c3 GTGTGGGAGACAGATTGGATAGTGGCATATTTTTTTGCATAGAAATCTGTTAGCTGTATG
NC_000001.11:33 ------------------------------------------------------------
NC_000023.11:15 ------------------------------------------------------------
NC_000004.12:c1 ------------------------------------------------------------

NC_000013.11:c3 GCTGCTGTTTCAGCATCCCTGAATCCGCCTGGCTGAAATGGGCCATCTATCTATTCACCT
NC_000001.11:33 ------------------------------------------------------------
NC_000023.11:15 ------------------------------------------------------------
NC_000004.12:c1 ------------------------------------------------------------

NC_000013.11:c3 TTGTTCACAACGATAGAAATCGTGTCCAATATTCTCTATGTGCTTTTTCTCACTGAAATC
NC_000001.11:33 ------------------------------------------------------------
NC_000023.11:15 ------------------------------------------------------------
NC_000004.12:c1 ------------------------------------------------------------

NC_000013.11:c3 TGGCATCATAATGACTCAGCATCTGGAGGAGCCAATACAAGTGAATCAAGTTTGCGTTCA
NC_000001.11:33 ------------------------------------------------------------
NC_000023.11:15 ------------------------------------------------------------
NC_000004.12:c1 ------------------------------------------------------------

NC_000013.11:c3 TCACCCAGCAGTGCCTCGTATGCCTAAGGGATTTCATGGAACTTCATTTCAAAATCATAG
NC_000001.11:33 ------------------------------------------------------------
NC_000023.11:15 ------------------------------------------------------------
NC_000004.12:c1 ------------------------------------------------------------

NC_000013.11:c3 TTTGAAATGGTCATGTTTCAAACAACTATAAAGAAACTTTAAAATGTGGCAAAATGTGGA
NC_000001.11:33 ------------------------------------------------------------
NC_000023.11:15 ------------------------------------------------------------
NC_000004.12:c1 ------------------------------------------------------------

NC_000013.11:c3 CAGGGCCTTAAATGTAGGAAGAATGTTCAGATAACTTTGCAAACAGCTTTTTGGTGATAG
NC_000001.11:33 ------------------------------------------------------------
NC_000023.11:15 ------------------------------------------------------------
NC_000004.12:c1 ------------------------------------------------------------

NC_000013.11:c3 CTCAGTTACTTCTTGCCTCATTAGTTGTCCTTGGTTCATAGGACATCCGTGACCCAGCAT
NC_000001.11:33 ------------------------------------------------------------
NC_000023.11:15 ------------------------------------------------------------
NC_000004.12:c1 ------------------------------------------------------------

NC_000013.11:c3 AAGTCAATGATAAAGTTTGTTCCAATACACATTATTTCTCCCTTTCAGAATGAGTGGCCT
NC_000001.11:33 ------------------------------------------------------------
NC_000023.11:15 ------------------------------------------------------------
```

```
NC_000004.12:c1 ------------------------------------------------------------

NC_000013.11:c3 TGGCAGATGTGAAACCTCTCTGAGCAGGTAATGTCCCTAAAGGGGGCACGCAGAGAGACT
NC_000001.11:33 ------------------------------------------------------------
NC_000023.11:15 ------------------------------------------------------------
NC_000004.12:c1 ------------------------------------------------------------

NC_000013.11:c3 GTGCAGGGTCACATTGTGATGGAGATGTGGGTAAAGAAACTCACAGACACCATTGCCTTT
NC_000001.11:33 ------------------------------------------------------------
NC_000023.11:15 ------------------------------------------------------------
NC_000004.12:c1 ------------------------------------------------------------

NC_000013.11:c3 TCCATCATTTAGGATGTCTTTACTGCTTTCTTCATTTCACCATCCAGAAATACCGAATTA
NC_000001.11:33 ------------------------------------------------------------
NC_000023.11:15 ------------------------------------------------------------
NC_000004.12:c1 ------------------------------------------------------------

NC_000013.11:c3 TAAAGGATGTGATTCTTTCTGACTACCCAAGACATCCAAAATTCAATCTTTTTTTTTTTT
NC_000001.11:33 ------------------------------------------------------------
NC_000023.11:15 ------------------------------------------------------------
NC_000004.12:c1 ------------------------------------------------------------

NC_000013.11:c3 TTTTTTTTTTTTTTTTTGAGACGCAGTCTCGCCCTGTCGCCCAGGCTGGAGTGCAGTGGC
NC_000001.11:33 ------------------------------------------------------------
NC_000023.11:15 ------------------------------------------------------------
NC_000004.12:c1 ------------------------------------------------------------

NC_000013.11:c3 GCGATCTCGGCTCACTGCAAGCTCCGCCTCCCGGGTTCACACCATTCTCCTGCCTCAGCC
NC_000001.11:33 ------------------------------------------------------------
NC_000023.11:15 ------------------------------------------------------------
NC_000004.12:c1 ------------------------------------------------------------

NC_000013.11:c3 TACCAAGGAGCTGGGACTAGAGGCGCCCGCCACCACGCCTGGCTAATTTTTTTGTGTTTT
NC_000001.11:33 ------------------------------------------------------------
NC_000023.11:15 ------------------------------------------------------------
NC_000004.12:c1 ------------------------------------------------------------

NC_000013.11:c3 TAGTAGAGACGGGGTTTCACCGTGTTAGCCAGGATGGTCTCGATCTCCTGACCTCGTGAT
NC_000001.11:33 ------------------------------------------------------------
NC_000023.11:15 ------------------------------------------------------------
NC_000004.12:c1 ------------------------------------------------------------

NC_000013.11:c3 CAGCCCACCTCGGTCTCCCAAAGTGCTGGGATTACAGGAGTGAGCCACCGCGCCCGGCCC
NC_000001.11:33 ------------------------------------------------------------
NC_000023.11:15 ------------------------------------------------------------
NC_000004.12:c1 ------------------------------------------------------------

NC_000013.11:c3 CAAAATTCAATCTTATGCAAACTTCAGTTGAGGATGTTTCCCCTGATTCTTTCGCAGCTT
NC_000001.11:33 ------------------------------------------------------------
NC_000023.11:15 -------------------------------------------------------AGTTT
NC_000004.12:c1 ------------------------------------------------------------

NC_000013.11:c3 CCACATCCCAGTAGTTCTCCGTTTTTAACTTATCAGCTGTTCTCTATGGATGTCATGCCT
```

```
NC_000001.11:33 ------------------------------------------------------------
NC_000023.11:15 CCAAAACATAGCAGTTCT------------------------------------------
NC_000004.12:c1 ------------------------------------------------------------

NC_000013.11:c3 TGAGATGTTATCTTCCAAACACACAGCATACACAACTAACTCATTGTTTCTCTTTTTAGA
NC_000001.11:33 ------------------------------------------------------------
NC_000023.11:15 ------------------------------------------------------------
NC_000004.12:c1 ------------------------------------------------------------

NC_000013.11:c3 AAATTAAGCAAATATGTATATAACTGCATATGCGGGCGTCTGGTCAACCAGAGGCAACAA
NC_000001.11:33 -----------GTGTGTGTGTGTGTGTGTGTGTGTGTGTCTG------------------
NC_000023.11:15 -----------GTAAACGTAACACTGAGTAC-----------------------------
NC_000004.12:c1 ------------------------------------------------------------

NC_000013.11:c3 ACGTGACCTCACAAGTTTGTGTAAACTCAGTCAAAAGCAAGGGAGCGCAATACGGTAGTC
NC_000001.11:33 ------------------------------------------------------------
NC_000023.11:15 ------------------------------------------------------------
NC_000004.12:c1 ------------------------------------------------------------

NC_000013.11:c3 CTTTTATGGGTTAATATGATCTCACCCTCCCCCCACCACAGTAGTGATCTTTCTGTTCCT
NC_000001.11:33 ------------------------------------------------------------
NC_000023.11:15 ------------------------------------------------------------
NC_000004.12:c1 ------------------------------------------------------------

NC_000013.11:c3 CAAAAGGGAAGGGCCCTGGGCTGGGAACCCCTTCAGTTATCCCTTCTGAATGGTACAAAT
NC_000001.11:33 ------------------------------------------------------------
NC_000023.11:15 ------------------------------------------------------------
NC_000004.12:c1 ------------------------------------------------------------

NC_000013.11:c3 CTCCTTCATCCCATCCCTCTGATGGTCCCAGTTGGTTGTGGTTGGCCATGAAAGAAGGTA
NC_000001.11:33 ------------------------------------------------------------
NC_000023.11:15 ------------------------------------------------------------
NC_000004.12:c1 ------------------------------------------------------------

NC_000013.11:c3 AATGGCTCAAGATCACTGCACCTCATTCCCCACCTGAAAAAAGACATTTCATATTTACAT
NC_000001.11:33 ------------------------------------------------------------
NC_000023.11:15 ------------------------------------------------------------
NC_000004.12:c1 ------------------------------------------------------------

NC_000013.11:c3 TTTGCTGGCGATGGGAGCCCTGAGTCAAAACCTGTACGTAGAAAACAGCCCAGGAGACGG
NC_000001.11:33 ------------------------------------------------------------
NC_000023.11:15 ------------------------------------------------------------
NC_000004.12:c1 ------------------------------------------------------------

NC_000013.11:c3 GTTGCCTGGTCACTGGCGCTGCAGCACACCGAGTTCCCTGCTCTGGGTCAGGTTGTTAAG
NC_000001.11:33 ------------------------------------------------------------
NC_000023.11:15 ------------------------------------------------------------
NC_000004.12:c1 ------------------------------------------------------------

NC_000013.11:c3 ACCGCATGAGGATGCGCTGCTGTAAAAGTGCCTGCTTTTTCCAATTTAGGTTCACCAGAG
NC_000001.11:33 ------------------------------------------------------------
NC_000023.11:15 ------------------------------------------------------------
NC_000004.12:c1 ------------------------------------------------------------
```

```
NC_000013.11:c3 TGCATAAATCCACTGACAGCTCTCTTCAAACAGTTGTTCTCAACTTTAAATAAAATTTTG
NC_000001.11:33 ------------------------------------------------------------
NC_000023.11:15 ------------------------------------------------------------
NC_000004.12:c1 ------------------------------------------------------------

NC_000013.11:c3 AAATAACACCTGGTTTGCCTTTTTAAAATTTCAATTTTAAAAGAGACAGGGCAGCAGGAG
NC_000001.11:33 ------------------------------------------------------------
NC_000023.11:15 ------------------------------------------------------------
NC_000004.12:c1 ------------------------------------------------------------

NC_000013.11:c3 GGTATAGATTAGGGGGCTATGGAAGGCATTCTACTATGGGGGGAAGAGCACACATCTTTC
NC_000001.11:33 ------------------------------------------------------------
NC_000023.11:15 ------------------------------------------------------------
NC_000004.12:c1 ------------------------------------------------------------

NC_000013.11:c3 AGAGTTTGACTTTGAACCTTGACTTCACTAAACATTTGGCTATATGACCTTGGGAAGGTT
NC_000001.11:33 ------------------------------------------------------------
NC_000023.11:15 ------------------------------------------------------------
NC_000004.12:c1 ------------------------------------------------------------

NC_000013.11:c3 ATTTTTAAGCTTTGATTCCCTTCTCTGGGAGCAGGAATAAGACTCCTTTGGAGTCTTGTG
NC_000001.11:33 ------------------------------------------------------------
NC_000023.11:15 ------------------------------------------------------------
NC_000004.12:c1 ------------------------------------------------------------

NC_000013.11:c3 GACATTTATGAATAATAACTTCAGCACAGCATAGGGCTGGGTGTGGGGTCAGGGCTGAGT
NC_000001.11:33 ------------------------------------------------------------
NC_000023.11:15 ------------------------------------------------------------
NC_000004.12:c1 ------------------------------------------------------------

NC_000013.11:c3 AAAGGTGAGCTTGCCCATCTCCTCTCCATCTCTTTTTGTGTCACACTGTATCGGCGCCTT
NC_000001.11:33 ------------------------------------------------------------
NC_000023.11:15 ------------------------------------------------------------
NC_000004.12:c1 ------------------------------------------------------------

NC_000013.11:c3 CTGTTTCTGCAGTTAAGGGCAGTGCTGGATGTCGTGGTGATGCTGACCCTGAGGAAGTGA
NC_000001.11:33 ------------------------------------------------------------
NC_000023.11:15 ------------------------------------------------------------
NC_000004.12:c1 ------------------------------------------------------------

NC_000013.11:c3 CAGCCCACTTTCTCAGGGGAAGTGGTGGCATAGACACAGGCAATCATATGTCATTCATAG
NC_000001.11:33 ------------------------------------------------------------
NC_000023.11:15 ------------------------------------------------------------
NC_000004.12:c1 ------------------------------------------------------------

NC_000013.11:c3 AAGCAAGTCCCCAGCCACTGTGGACCACCGACATCTGAGGAAGGTGGGAAGAGGCTCAGA
NC_000001.11:33 ------------------------------------------------------------
NC_000023.11:15 ------------------------------------------------------------
NC_000004.12:c1 ------------------------------------------------------------

NC_000013.11:c3 GGTAGGAGCTACCACCCCAACTCAGGCGTTACTCACCGAGCTACATGGCAGTCTCAGGGT
NC_000001.11:33 ------------------------------------------------------------
NC_000023.11:15 ------------------------------------------------------------
```

```
NC_000004.12:c1 ------------------------------------------------------------

NC_000013.11:c3 GGGGACAAAGGTCAGGGGGCCCACCATCCCGAGAGGTTCCTTCCTTGGTGAGCTGACAGG
NC_000001.11:33 ------------------------------------------------------------
NC_000023.11:15 ------------------------------------------------------------
NC_000004.12:c1 ------------------------------------------------------------

NC_000013.11:c3 CCAGCTTCTCCCTGAAGTTTCTTACAACTGAAGTTTCAGTTCTGGCAAACAGACTACGGA
NC_000001.11:33 ------------------------------------------------------------
NC_000023.11:15 ------------------------------------------------------------
NC_000004.12:c1 ------------------------------------------------------------

NC_000013.11:c3 CCTAAGTCCTAGGCCCAGCAACTGCCTTCCCAGGCAGGTCCCATTCCATTTTTGAGATCA
NC_000001.11:33 ------------------------------------------------------------
NC_000023.11:15 ------------------------CCTACTTGGGTCGGCTCCATT---------------
NC_000004.12:c1 ------------------------------------------------------------

NC_000013.11:c3 CTGCTCCTTCTCAGCTTTCTCGCCCGCCTCCCTGGAGAGAGAAGGTTCAGCACCGGATGC
NC_000001.11:33 ------------------------------------------------------------
NC_000023.11:15 ------------------------------------------------------------
NC_000004.12:c1 ------------------------------------------------------------

NC_000013.11:c3 ACAGCCGCTGCGGCCCCTGCTCTGTGAGGACTCTTGAGCCTCTAGCGGGGAATGACCCAT
NC_000001.11:33 ------------------------------------------------------------
NC_000023.11:15 ------------------------------------------------------------
NC_000004.12:c1 ------------------------------------------------------------

NC_000013.11:c3 GCTACTTGTTAAAAGGCGGGCTCGGCCCCTCCGGGCTCCTCCTGGCCAAGGAGCCTGCGT
NC_000001.11:33 ------------------------------------------------------------
NC_000023.11:15 ------------------------------------------------------------
NC_000004.12:c1 ------------------------------------------------------------

NC_000013.11:c3 AGCTGCAGGATGTAAAGCCAAAGGCCATCAGAAGGATGTGTGGAAAACACTGCGGTTAGG
NC_000001.11:33 ------------------------------------------------------------
NC_000023.11:15 ------------------------------------------------------------
NC_000004.12:c1 ------------------------------------------------------------

NC_000013.11:c3 AATGAGCAGACATTCCTAATGGAGCACGGACCTCAGGCTGATTGTGATCTGAGACAAAAC
NC_000001.11:33 ------------------------------------------------------------
NC_000023.11:15 -------------------TGGAGCAC---------------------------------
NC_000004.12:c1 ------------------------------------------------------------

NC_000013.11:c3 AGTGCCAAGTGCTGCTAAATCATTAGAACACTTGTGGATAACAGCAAGAGAAATAAAGAC
NC_000001.11:33 ------------------------------------------------------------
NC_000023.11:15 ------------------------------------------------------------
NC_000004.12:c1 ------------------------------------------------------------

NC_000013.11:c3 ACACAGACTCTCTCACAGGAAGGCTCTGTGGTATGGAGAGGAGCTTTGGCGACAGAGGGC
NC_000001.11:33 ------------------------------------------------------------
NC_000023.11:15 ------------------------------------------------------------
NC_000004.12:c1 ------------------------------------------------------------

NC_000013.11:c3 AGAGGCCCCTTTCTGGTTGTCTTGGGACAAGTCATTTAGTGATGGTGGAGCTAGGGCTCG
```

NC_000001.11:33 --
NC_000023.11:15 --
NC_000004.12:c1 --

NC_000013.11:c3 TGGTGGCAGGCTTGCTGTCTCCCCATATCAAGGTCTTGCTGTGTGCCAAGCACTGTCAGG
NC_000001.11:33 --
NC_000023.11:15 --
NC_000004.12:c1 --

NC_000013.11:c3 TTCCTCGTAGACGTCATGTCCCAATAGCCCTATGAGGCAGGCACTGTGATTATAGCAATT
NC_000001.11:33 --
NC_000023.11:15 --
NC_000004.12:c1 --

NC_000013.11:c3 TGATTTTAATTTTTTTTAGGTTATCTGGTGAGGTTGCAGATCTCTCTGGATGAAGAATCT
NC_000001.11:33 --
NC_000023.11:15 -----TTGATCGCTTTTGAG--
NC_000004.12:c1 --

NC_000013.11:c3 CAGACTCAGAGGTGAAATGATATGCAGGCAGAACCAGTCTCACTGTTCTCTGTCTTAATG
NC_000001.11:33 --
NC_000023.11:15 --
NC_000004.12:c1 --

NC_000013.11:c3 TCTCTCAGCCTTCATTTATCATCTGCAAAATGGGAATAAAAAATACAATCTCATAGAGTT
NC_000001.11:33 --
NC_000023.11:15 --
NC_000004.12:c1 --

NC_000013.11:c3 AGAGAAATAATGAATAATTCACAAGTCTGTGGTATATAATATGTGTTTGGGCCAGGCGTA
NC_000001.11:33 --
NC_000023.11:15 -----------AAGTAACTCGCA-------------------------------------
NC_000004.12:c1 --

NC_000013.11:c3 GTGGCTTGCGCCTATAAACCCAGCACTTTGGGAGGCCGAGGCAGGCGCATCACTTGAGGT
NC_000001.11:33 --
NC_000023.11:15 --
NC_000004.12:c1 --

NC_000013.11:c3 CAGGAGTTTGAGACCAGCCTGGCCAACATGGTGAAACCCCATCTCTACTAAAAATAGAAA
NC_000001.11:33 --
NC_000023.11:15 --
NC_000004.12:c1 --

NC_000013.11:c3 AATGAGCCAGACATGGTGGTGCATGCCTGTAATCCCAGCTACTCAGGAGACTGAGGCGTG
NC_000001.11:33 --
NC_000023.11:15 --
NC_000004.12:c1 --

NC_000013.11:c3 AGAATCACTTGAACCTGGAAGGTGGTGGTTGCATTGAGCCAATATCATGCTACTGCCTTC
NC_000001.11:33 --
NC_000023.11:15 --
NC_000004.12:c1 --

```
NC_000013.11:c3 CAGCCTGGGCATCAGAGCAAGACCCTGTCTCAAAAAAAAGAAATAAAAGTTGTTAGATGT
NC_000001.11:33 ------------------------------------------------------------
NC_000023.11:15 ------------------------------------------------------------
NC_000004.12:c1 ------------------------------------------------------------

NC_000013.11:c3 GGGTTATTTAATGTTGCATATTGCCTTCCAATTTTAAAAAAAATTGCCAGGCTGAGCACA
NC_000001.11:33 ------------------------------------------------------------
NC_000023.11:15 ------------------------------------------------------------
NC_000004.12:c1 ------------------------------------------------------------

NC_000013.11:c3 GTGGCTCATGCCTATAATCCCAACACTTTGGGAGGCCAAAGCGGACAGATCGCTTGAGCT
NC_000001.11:33 ------------------------------------------------------------
NC_000023.11:15 ------------------------------------------------------------
NC_000004.12:c1 ------------------------------------------------------------

NC_000013.11:c3 CAGAAGCTCCAGACCAGCCTGGGCAACATGACGAAACCCCCATTATATGGTTAGGCTTTG
NC_000001.11:33 ---------------AGCCTGAGCAG----------------------------------
NC_000023.11:15 ---------------AACCAGCGCGC----------------------------------
NC_000004.12:c1 ------------------------------------------------------------

NC_000013.11:c3 TGTCCCCCCACAATCTCATCTTGAATTGTAATCCCCACCTGTCAAGGGAGAGACCAGATG
NC_000001.11:33 ------------------------------------------------------------
NC_000023.11:15 ------------------------------------------------------------
NC_000004.12:c1 ------------------------------------------------------------

NC_000013.11:c3 GAGGTAATTGAATCACAGGGGCCATTTCCCCATGCTGTTCTTATGGTAGCGAGTGAGTTC
NC_000001.11:33 ------------------------------------------------------------
NC_000023.11:15 ------------------------------------------------------------
NC_000004.12:c1 ------------------------------------------------------------

NC_000013.11:c3 CCGTGAGACCTGATGGTTTTTTTTTTTATGTTAATAATGGAGTTTAATATTTTGTTTCTG
NC_000001.11:33 ------------------------------------------------------------
NC_000023.11:15 ------------------------------------------------------------
NC_000004.12:c1 ------------------------------------------------------------

NC_000013.11:c3 TGCAATCTTATGAAATACAACTTGTTTTATAAGAATTTTCAACAAAGTATTAGTTTCCTT
NC_000001.11:33 ---AGTCTGATGAAT-------------ATGTGGACTTTCATTAAAG-------------
NC_000023.11:15 TTCGTGCTGGTCAAA-------AGTTAAATGACAAGCGACAATGAAGC------------
NC_000004.12:c1 ------------------------------------------------------------

NC_000013.11:c3 TTGCAAAAAGTTATTAGTTTGCCATTTAGTAGCTTTTAAGTATGTTGAATTTTTTTACAT
NC_000001.11:33 ------------------------------------------------------------
NC_000023.11:15 -----------------------------------------------------------T
NC_000004.12:c1 ------------------------------------------------------------

NC_000013.11:c3 AATTAAGAACCTCAATATTCTTGTTATCAAAAACATTTAGTGAGTACCTCCATGTGCAAA
NC_000001.11:33 ------------------------------------------------------------
NC_000023.11:15 GATT--------------------------------------------------------
NC_000004.12:c1 ------------------------------------------------------------

NC_000013.11:c3 ACAACTGGGCTAAAGAGTCATAGGACAGAGCCCTACTGTCAAGGATCTTTGAGTCTGTCT
NC_000001.11:33 ------------------------------------------------------------
NC_000023.11:15 ------------------------------------------------------------
```

```
NC_000004.12:c1 ------------------------------------------------------------


NC_000013.11:c3 TGAGAGATACTTTATTCTTTCATCTCTTCCTTTGCTGTGGTTCACATCTGATGTCACAAA
NC_000001.11:33 ----------TTCTTCCTCATGTCCCATCTTTTG--------------------------
NC_000023.11:15 ----------TCTTTCCTACTCTGCCTTCTGCCACTG-----------------------
NC_000004.12:c1 ----------TCCGGCTTCCCCTCTCCCCCCTCG--------------------------
                          *..  ..*  . * .* .*. ...

NC_000013.11:c3 AACCAGTGGGGGTTGGGAGTACGCCAAAGAACAAGAATACTGAGACATTTTGCTGCAATA
NC_000001.11:33 ------------------------------------------------------------
NC_000023.11:15 ------------------------------------------------------------
NC_000004.12:c1 ------------------------------------------------------------


NC_000013.11:c3 TTTTGTGCATCCCTAAAGGCCATTTCTAAATAACAACAATGGTGATGACAACAAAATTAC
NC_000001.11:33 ------------------------------------------------------------
NC_000023.11:15 ------------------------------------------------------------
NC_000004.12:c1 ------------------------------------------------------------


NC_000013.11:c3 ACCTGTCATTTATCAGGAACATACAATGTCAAAGCACTGTGCTGAATGATCATTTATCTT
NC_000001.11:33 ------------------------------------------------------------
NC_000023.11:15 ------------------------------------------------------------
NC_000004.12:c1 ------------------------------------------------------------


NC_000013.11:c3 TAAATGATCAGATGCTTTCAATGATCATCCAGTTCTTCCACCTACTCTCTGAGGTTTCTA
NC_000001.11:33 ------------------------------------------------------------
NC_000023.11:15 ------------------------------------------------------------
NC_000004.12:c1 ------------------------------------------------------------


NC_000013.11:c3 TTCTTATTTTTGCCATTTTATACATGGAGTCTGAGCTTAGAGAGATTAAGAAACTTCTAT
NC_000001.11:33 ------------------------------------------------------------
NC_000023.11:15 ------------------------------------------------------------
NC_000004.12:c1 ------------------------------------------------------------


NC_000013.11:c3 TATTTTAATAGTTAGCAGAGTTCGGATTTAAACTCAATCCTGTTAGACTTCAAAGCCATA
NC_000001.11:33 ------------------------------------------------------------
NC_000023.11:15 ------------------------------------------------------------
NC_000004.12:c1 ------------------------------------------------------------


NC_000013.11:c3 TTTGTAACCATTTTACTATGCCGATTTGTTCTGTCTGGCCTACAAATAAGAATGTATTTG
NC_000001.11:33 ------------------------------------------------------------
NC_000023.11:15 ------------------------------GTGTCCGGC---------------------
NC_000004.12:c1 ------------------------------------------------------------


NC_000013.11:c3 TTTGTTTAATACTTATTTGTTGGATATCTACTATGTGATAAAAAAACAACGCATATATAA
NC_000001.11:33 ------------------------------------------------------------
NC_000023.11:15 ------------------------------------------------------------
NC_000004.12:c1 ------------------------------------------------------------


NC_000013.11:c3 CCACAGGCAAGGTTCCAATTAAGAAATAAATCTTAGGAGGGGCCTGTGGTGAAATACAAT
NC_000001.11:33 -----------------------------ATCTCAGAAAGGAGCTGTG------------
NC_000023.11:15 -----------------------------ATTGGGGGAAGGGGACTTC------------
NC_000004.12:c1 -----------------------------GCCCCGTCAGGTGGACGCG------------
                                               ...  .  *.* .  . .

NC_000013.11:c3 GTGACCAAATCTTTATATATCTTTTCAAGTGTATAAACTGCAGATGGTTACAGACATAAA
```

```
NC_000001.11:33 ------------------------------------------------------------
NC_000023.11:15 ---------------------------------CTAACAGCAAGTG--------------
NC_000004.12:c1 ------------------------------------------------------------


NC_000013.11:c3 TATTGGTAACTTCATATACCAGTTTCAGTTAGCCAAGTCTGTTGAAGAATTTCACCAAAT
NC_000001.11:33 ------------------------------------------------------------
NC_000023.11:15 ------------------------------------------------------------
NC_000004.12:c1 ------------------------------------------------------------


NC_000013.11:c3 TATTATTATTATTATTATTATTATTTATTTTATTTTATTTTTGAGACAGAGTCTTGCTCT
NC_000001.11:33 ------------------------------------------------------------
NC_000023.11:15 ------------------TAGTTTTAGTTTTGCTCTGCCTTTGGTACG------------
NC_000004.12:c1 ------------------------------------------------------------


NC_000013.11:c3 TGTCACCCAGGCTGGAGTGCAATGGCACGATCTTGGTTCACTGCAACCTCTGCCTCCTGG
NC_000001.11:33 ------------------------------------------------------------
NC_000023.11:15 ------------------------------------------------------------
NC_000004.12:c1 ------------------------------------------------------------


NC_000013.11:c3 GTTCAAATGATTCTCCTGCCTCAGCCTCCCAAGTAGCTGGGATTACAGGCACCTGATGGT
NC_000001.11:33 ------------------------------------------------------------
NC_000023.11:15 ------------------------------------------------------------
NC_000004.12:c1 ------------------------------------------------------------


NC_000013.11:c3 GGTTTTATAAGTGTTTGGTAGTTCCTCCTGCATTCATTCCCTCTCCTGCCGCCTTGTAAA
NC_000001.11:33 ------------------------------------------------------------
NC_000023.11:15 ------------------------------------------------------------
NC_000004.12:c1 ------------------------------------------------------------


NC_000013.11:c3 GAAGGTGCCTTGCTTCCCCTTCACCGTCCACCACGATTGTAAGTTTCCTGAAGTCTTCCT
NC_000001.11:33 --------------TCCCATTCATC-----------------------------------
NC_000023.11:15 --------------TCTTGTACTTC-----------------------------------
NC_000004.12:c1 ------------------------------------------------------------


NC_000013.11:c3 AGCCGTGCTCAACTGTGAGTCGATTAAACCTCTTTCCACCCAGTCTCAGGCAGTTCTTCT
NC_000001.11:33 ----------------------------------------------TAGGCAGCCCTGCT
NC_000023.11:15 ----------------------------------------------TTGTCAATTCCTCC
NC_000004.12:c1 ---------------------GAT----------------------CTGTCAAC------
                                                              . * **..

NC_000013.11:c3 TTTTTTTCTTTTTTTTTTTTTTTTTTTTTAATATACTTTAAGCTCTGGGATACATGTGCAG
NC_000001.11:33 C-----------------------------------------------------------
NC_000023.11:15 TTGCTTT-----------------------------------------------------
NC_000004.12:c1 ------------------------------------------------------------


NC_000013.11:c3 AACGTGCAGGTTTTATTAATAGGTATACATGTGCTATGGTGGTTTGCTGCACCCATCAAC
NC_000001.11:33 ------------------------------------------------------------
NC_000023.11:15 ------------------------------------------------------------
NC_000004.12:c1 ------------------------------------------------------------


NC_000013.11:c3 CCATCATCTACATTAGGTATTTGTCCTAATGCTCTCCCTCCCCTTGCTCCCCACCCCTTG
NC_000001.11:33 ------------------------------------------------------------
NC_000023.11:15 ------------------------------------------------------------
NC_000004.12:c1 ------------------------------------------------------------
```

```
NC_000013.11:c3 ACAGGCCCTGGTGTGTGATGTTCCCCTCCCTGTGTCCATGTGTTCTCATTGTTCAACTCC
NC_000001.11:33 ------------------------------------------------------------
NC_000023.11:15 ------------------------------------------TCCTCATTGT--------
NC_000004.12:c1 ------------------------------------------------------------

NC_000013.11:c3 CCCTTACGAGTGAGAGCATGTGGTGTTTGGTTTTCTGTTCCTGTGTTAGTTTGCTGAGAA
NC_000001.11:33 ------------------------------------------------------------
NC_000023.11:15 ------------------------------------------------------------
NC_000004.12:c1 ------------------------------------------------------------

NC_000013.11:c3 TGATGGTTTCCAGCTTCATCCATGTCCCTGCAAAGGACATGAACTAATCCTTTTTTATGG
NC_000001.11:33 ------------------------------------------------------------
NC_000023.11:15 ------------------------------------------------------------
NC_000004.12:c1 ------------------------------------------------------------

NC_000013.11:c3 CTGCATAGTATTCCATGGTGTATATGTGTCCAGCAGTTCTTTATAGCAGTGTGAAAATGG
NC_000001.11:33 ------------------------------------------------------------
NC_000023.11:15 ------------------------------------------------------------
NC_000004.12:c1 ------------------------------------------------------------

NC_000013.11:c3 ACTAATACACCCCCTCTCTACAAAAGATACAAAAAAAACAGTGGGGGAATGGTGGCACTC
NC_000001.11:33 ------------------------------------------------------------
NC_000023.11:15 ------------------------------------------------------------
NC_000004.12:c1 ------------------------------------------------------------

NC_000013.11:c3 TCCTGTAGTCCCAACTACATAGGAAGCTGAAGCAGGAAGATTGCTTGAGTCTGGGAGATT
NC_000001.11:33 ------------------------------------------------------------
NC_000023.11:15 ------------------------------------------------------------
NC_000004.12:c1 ------------------------------------------------------------

NC_000013.11:c3 GGGGTTGCAGTGAGCCAAGATGGCACCACTGCACTCCAGCCTGGGCAATAGAGTGAGGCC
NC_000001.11:33 ------------------------------------------------------------
NC_000023.11:15 ------------------------------------------------------------
NC_000004.12:c1 ------------------------------------------------------------

NC_000013.11:c3 CCATCTCAAACAAACAAAAAAAAAAGGGCCCAACAAAAACAAATGCCAAATGAAGCACCAG
NC_000001.11:33 ------------------------------------------------------------
NC_000023.11:15 ------------------------------------------------------------
NC_000004.12:c1 ------------------------------------------------------------

NC_000013.11:c3 ATGGTACAAGTAGCAGCTGCTGTAAGCAAGATGAGGGGATAGGCTGAGCATGGCTTACGT
NC_000001.11:33 ------------------------------------------------------------
NC_000023.11:15 ------------------------------------------------------------
NC_000004.12:c1 ------------------------------------------------------------

NC_000013.11:c3 GTGAAAAGGACACTGGTTCTTTCAACCACACCAGCATATGCAGAGAGTGGGCCCTATCCT
NC_000001.11:33 ------------------------------------------------------------
NC_000023.11:15 ------------------------------------------------------------
NC_000004.12:c1 ------------------------------------------------------------

NC_000013.11:c3 AATATCATTTTGTTGTACAAAGAGTGGTAACATCTTTTGGACAAATCCTGAAAAAAAAAA
NC_000001.11:33 ------------------------------------------------------------
NC_000023.11:15 ------------------------------------------------------------
```

```
NC_000004.12:c1 ------------------------------------------------------------

NC_000013.11:c3 CTTGGCTTCAGTAGATAGATATACCACATGCTGATTAACCTATCTCACTCTGTAACTCCA
NC_000001.11:33 ------------------------------------------------------------
NC_000023.11:15 ------------------------------------------------------------
NC_000004.12:c1 ------------------------------------------------------------

NC_000013.11:c3 ATAAGATTTTATGGTCCAGTGATTCGAAGTTGACTTTTCCTAGCAAAGTAGGAAAGCTTT
NC_000001.11:33 ------------------------------------------------------------
NC_000023.11:15 ------------------------------------------------------------
NC_000004.12:c1 ------------------------------------------------------------

NC_000013.11:c3 ATTTAGAGGCCTTTTCCCTTGCTCAGCCAGGGTTGAGCAATGTGCAGAGTTTCTTTGACT
NC_000001.11:33 ------------------------------------------------------------
NC_000023.11:15 ------------------------------------------------------------
NC_000004.12:c1 ------------------------------------------------------------

NC_000013.11:c3 AGGAAGGAGTGGTGCTGTAGCACTTAACCTCCAAGGAGATTATTTCATAAAATATTGATT
NC_000001.11:33 ------------------------------------------------------------
NC_000023.11:15 ------------------------------------------------------------
NC_000004.12:c1 ------------------------------------------------------------

NC_000013.11:c3 TTTATGCACAAAAAGGAGATAGTTCATGAATAGAAAATGTAAGCGTAAGCAAAATTTTTT
NC_000001.11:33 ------------------------------------------------------------
NC_000023.11:15 -----------------------------------------------------ATTTCTT
NC_000004.12:c1 ------------------------------------------------------------

NC_000013.11:c3 CAGTTCTCTCAGTTCATAAACACAAAAAGTAATGAATCCTGTAACCAAGGCTGATGTCTA
NC_000001.11:33 ------------------------------------------------------------
NC_000023.11:15 TGTTTCTCT---------------------------------------------------
NC_000004.12:c1 ------------------------------------------------------------

NC_000013.11:c3 CTGTACCTGCTGGGAGATAGGCAGAGCTAGAAAGTCATTCTTCCCCAATGTTATAACTAC
NC_000001.11:33 ------------------------------------------------------------
NC_000023.11:15 ------------------------------------------------------------
NC_000004.12:c1 ------------------------------------------------------------

NC_000013.11:c3 AAGAACTGTTGTACCAAAAGTAGACCAACCTGGATGGTACAGCCATTTCCTGTAGGCTGT
NC_000001.11:33 ------------------------------------------------------------
NC_000023.11:15 ------------------------------------------------------------
NC_000004.12:c1 ------------------------------------------------------------

NC_000013.11:c3 GAAGGCAAGGCTATAGAATTTGTTGCAAGTTGGGCCAGGCACAGTGGCTCACGCCTGTAA
NC_000001.11:33 ------------------------------------------------------------
NC_000023.11:15 ------------------------------------------------------------
NC_000004.12:c1 ------------------------------------------------------------

NC_000013.11:c3 TCCCAACACTTTGGGAGGCCGAGGTGGGTGGATCACTTGAACCCAGGAATTCTAGACCAC
NC_000001.11:33 ------------------------------------------------------------
NC_000023.11:15 ------------------------------------------------------------
NC_000004.12:c1 ------------------------------------------------------------

NC_000013.11:c3 CCTGGGCAGTATGGCGAAACCCTGTCTACTAAAAATACAAAAATGAGCCAGGCACGGCAG
```

```
NC_000001.11:33 ------------------------------------------------------------
NC_000023.11:15 ------------------------------------------------------------
NC_000004.12:c1 ------------------------------------------------------------

NC_000013.11:c3 TGCATGCTTGTAGTCCCAGCTACTCAGGAGGCAGAGGTGGGAAGAATGCTTGTGCCTGGG
NC_000001.11:33 ------------------------------------------------------------
NC_000023.11:15 ------------------------------------------------------------
NC_000004.12:c1 ------------------------------------------------------------

NC_000013.11:c3 AAGTGGAGGTTGCACCACTGCACTAAAGCCAGGGCAATAGAGCAAGACCTTGTCTCAAAA
NC_000001.11:33 ------------------------------------------------------------
NC_000023.11:15 ------------------------------------------------------------
NC_000004.12:c1 ------------------------------------------------------------

NC_000013.11:c3 AAAAAAAAAGACTTTTAGACTTGTTTGCCTTCATAACCCAAGCTATAACTGAGCCTTTTC
NC_000001.11:33 ------------------------------------------------------------
NC_000023.11:15 ------------------------------------------------------------
NC_000004.12:c1 ------------------------------------------------------------

NC_000013.11:c3 CCAATATCACATCCATCTTACTGAGCTGCTGCAAACCAAAAATATGGTTTCACTAGCTAT
NC_000001.11:33 ------------------------------------------------------------
NC_000023.11:15 ------------------------------------------------------------
NC_000004.12:c1 ------------------------------------------------------------

NC_000013.11:c3 TGCTGTCTTGCAGATGCCAGTAGCCATGCCATATCACCTAAAGTGGTGTTGTCATACTAT
NC_000001.11:33 ------------------------------------------------------------
NC_000023.11:15 ------------------------------------------------------------
NC_000004.12:c1 ------------------------------------------------------------

NC_000013.11:c3 GTCCTGACAGCATTTCTGCACCCAGTCCTCCCCCTCCCCAGTTTACCTCTAATTCGTCAT
NC_000001.11:33 ------------------------------------------------------------
NC_000023.11:15 ------------------------------------------------------------
NC_000004.12:c1 ------------------------------------------------------------

NC_000013.11:c3 CACTCTTGACCTGACTCTATCACAGTTAGGTAGCTATCCTGGGGTTGGAACAGCCCGGAG
NC_000001.11:33 ------------------------------------------------------------
NC_000023.11:15 ---------------TTTATCACAGTCAGG------------------------------
NC_000004.12:c1 ------------------------------------------------------------

NC_000013.11:c3 GGGTAAAAAGGCAATGGTCTCAGAAACTAGAAGCGAGATTATGTCCAGGCACAGTGGCTC
NC_000001.11:33 ------------------------------------------------------------
NC_000023.11:15 ------------------------------------------------------------
NC_000004.12:c1 ------------------------------------------------------------

NC_000013.11:c3 ACACCTATAATCCCAACACTTTAAGAGGCTGAGATGGGAGGATCACTTGAGCCCAAAAGT
NC_000001.11:33 ------------------------------------------------------------
NC_000023.11:15 ------------------------------------------------------------
NC_000004.12:c1 ------------------------------------------------------------

NC_000013.11:c3 TTGAGACCAACATGTCAAGACCCAGTCTCTACAAAAAATAAAGAAGTTAGCTGGGCATGA
NC_000001.11:33 ------------------------------------------------------------
NC_000023.11:15 ------------------------------------------------------------
NC_000004.12:c1 ------------------------------------------------------------
```

```
NC_000013.11:c3 TGGCATCTACCTGCGGTCCCAGCTACTCAAGAGGCTGAGGTGAGAGGATCACTTGAGTCC
NC_000001.11:33 ------------------------------------------------------------
NC_000023.11:15 ------------------------------------------------------------
NC_000004.12:c1 ------------------------------------------------------------


NC_000013.11:c3 AGGAGTTCAAGATTGCAGTGAGCTATGATCATACCACTGCACTTCAACCTGGGTGACAGA
NC_000001.11:33 ------------------------------------------------------------
NC_000023.11:15 ------------------------------------------------------------
NC_000004.12:c1 ------------------------------------------------------------


NC_000013.11:c3 GCAAGACCTTGTCTTGCCCAAAAAAAAAAAAAAAAAGTATCATAATTATGTTTTAACTCA
NC_000001.11:33 ------------------------------------------------------------
NC_000023.11:15 ------------------------------------------------------------
NC_000004.12:c1 ------------------------------------------------------------


NC_000013.11:c3 CATTCTGTCACTAAGCTATCTTGAGCAAGTCAGTGAATTACTCTTAATGTTTGTTTCATT
NC_000001.11:33 ------------------------------------------------------------
NC_000023.11:15 ------------------------------------------------------------
NC_000004.12:c1 ------------------------------------------------------------


NC_000013.11:c3 GACTGTGTATAAGGAATGTTGATAAATGTTGCAAGGATCAAATGAGAAAATGCAGGTGAA
NC_000001.11:33 ------------------------------------------------------------
NC_000023.11:15 ------------------------------------------------------------
NC_000004.12:c1 ------------------------------------------------------------


NC_000013.11:c3 AGTGCTTTAAAAATAAGTGCAATGCCATAATGAGTTATAAGTTCACACCCACTAAGATGG
NC_000001.11:33 ------------------------------------------------------------
NC_000023.11:15 ------------------------------------------------------------
NC_000004.12:c1 ------------------------------------------------------------


NC_000013.11:c3 TTCTAATCACAAAAACAGATAATAACAAGTGTTGGACAAGTATGTAGAGAAATTGAAACC
NC_000001.11:33 ------------------------------------------------------------
NC_000023.11:15 ------------------------------------------------------------
NC_000004.12:c1 ------------------------------------------------------------


NC_000013.11:c3 CTCATATACTGCTGGTAGGATTGTAAAATGGTTCCTCTGCTTTGGAATACAAGGTGGCTG
NC_000001.11:33 ------------------------------------------------------------
NC_000023.11:15 ------------------------------------------------------------
NC_000004.12:c1 ------------------------------------------------------------


NC_000013.11:c3 TTCTTCAAACAGGTAAATATTGACTTATCCTGTGATCCAGCAGTTCTACTTCTAGGGATA
NC_000001.11:33 -----------------------------------------------------------A
NC_000023.11:15 ------------------------------------------------ATGGCTAAAGGTG
NC_000004.12:c1 ------------------------------------------------ATGGGTAAAGGAG
                                                                           .

NC_000013.11:c3 TACCCAAGAGACGTGAAAACACGTATCTACACAAAATCTTGTACATGAATGCTTATAGCA
NC_000001.11:33 TGTCCAAAAAATGTG---------------------------------------------
NC_000023.11:15 ACCCCAAGAAACCAA---------------------------------------------
NC_000004.12:c1 ACCCCAACAAGCCGC---------------------------------------------
                  .**** *...

NC_000013.11:c3 GCATTATTCATAATAGCCAAAAGCAGAAACCACTCATGTGTCCATCAACTGATGAGTGAA
NC_000001.11:33 ------------------------------------------------------------
NC_000023.11:15 ------------------------------------------------------------
```

```
NC_000004.12:c1 ------------------------------------------------------------

NC_000013.11:c3 TAAATAAAATGTGATATATCCATGCAACTGATATTATTCAACAATAAAAGGATATGAAGG
NC_000001.11:33 ------------------------------------------------------------
NC_000023.11:15 ------------------------------------------------------------
NC_000004.12:c1 ------------------------------------------------------------

NC_000013.11:c3 GCTCATATACTACAACATGGATGAACCTTGAAAACATTATGCTAAGGAAAGGAAGCCAGT
NC_000001.11:33 ------------------------------------------------------------
NC_000023.11:15 ------------------------------------------------------------
NC_000004.12:c1 ------------------------------------------------------------

NC_000013.11:c3 CACAAAAGACCACATATTGCAATTCCACTTATACAAAATATCCAGAATAGGCAAATCTAT
NC_000001.11:33 ------------------------------------------------------------
NC_000023.11:15 ------------------------------------------------------------
NC_000004.12:c1 ------------------------------------------------------------

NC_000013.11:c3 AGAGATAAAAAGTAGATTAATGGTGGCCAGGGGCTTGGAGTCAGGGGAGGAATGGATACT
NC_000001.11:33 ------------------------------------------------------------
NC_000023.11:15 ------------------------------------------------------------
NC_000004.12:c1 ------------------------------------------------------------

NC_000013.11:c3 AACTACTAATTGGTCCAGGATTTCTTTCTGGGGTAATGAAATTGTACTAAAATTGATTAT
NC_000001.11:33 ------------------------------------------------------------
NC_000023.11:15 ------------------------------------------------------------
NC_000004.12:c1 ------------------------------------------------------------

NC_000013.11:c3 CAGTACATTCTCAACATACTAAACTCCATTGAATTTTTCATTTTAAGTGGGTAGATTGTA
NC_000001.11:33 ------------------------------------------------------------
NC_000023.11:15 ------------------------------------------------------------
NC_000004.12:c1 ------------------------------------------------------------

NC_000013.11:c3 TGGTATGTGAATTTCATTTCAATAAAACCGTTCTGGGAAAAAATAAGGGCAACACAAAGG
NC_000001.11:33 ------------------------------------------------------------
NC_000023.11:15 ------------------------------------------------------------
NC_000004.12:c1 ------------------------------------------------------------

NC_000013.11:c3 CCATTGATAATAATACTGGGTTTTCAGCAAATGACCAAATGGAACTGAACTCCAGCAAAG
NC_000001.11:33 ------------------------------------------------------------
NC_000023.11:15 ------------------------------------------------------------
NC_000004.12:c1 ------------------------------------------------------------

NC_000013.11:c3 ATCAGTTCTGGGCTTGATTAACCTCTTACCAACATGTCTGAAATTCAGGACACATAATGG
NC_000001.11:33 ------------------------------------------------------------
NC_000023.11:15 ------------------------------------------------------------
NC_000004.12:c1 ------------------------------------------------------------

NC_000013.11:c3 AAGAGGTCTCACAATCTGCTTGCCAGGCCACAGTCGTCATGTCCATGAACATCAAAAGCA
NC_000001.11:33 ------------------------------------------------------------
NC_000023.11:15 ------------------------------------------------------------
NC_000004.12:c1 ------------------------------------------------------------

NC_000013.11:c3 CTAGTGTCCAAACTTAGAAAATGGGTATCAGTTGCCTGGAATAAAATCCCAGAAGGAAGA
```

```
NC_000001.11:33 ------------------------------------------------------------
NC_000023.11:15 ------------------------------------------------------------
NC_000004.12:c1 ------------------------------------------------------------

NC_000013.11:c3 GTGAAGCGCTCCTTTCCTTCCTGGGAACCAAATGTTTATGGGAAAAGAGGTGGGGAGAAT
NC_000001.11:33 ------------------------------------------------------------
NC_000023.11:15 ------------------------------------------------------------
NC_000004.12:c1 ------------------------------------------------------------

NC_000013.11:c3 GATAAATCATCACGTGCTTGCCAATAATCTTCAAGCAGGAATCAAAAATAGGCAAGCAAA
NC_000001.11:33 ------------------------------------------------------------
NC_000023.11:15 ------------------------------------------------------------
NC_000004.12:c1 ------------------------------------------------------------

NC_000013.11:c3 GAGCCCAGCCCCAAGAGCTTTTAGTACTGTTGGATTCTCTTAAGAAATGCTGCATCACCA
NC_000001.11:33 ------------------------------------------------------------
NC_000023.11:15 ------------------------------------------------------------
NC_000004.12:c1 ------------------------------------------------------------

NC_000013.11:c3 GTGCCCTTGGTGGCACAGAGGACGATACTGAGTGAATAAGCAGTGAAGCAGACTCTGAGT
NC_000001.11:33 -------------------------------------------------------TGAGT
NC_000023.11:15 -------------------------------------------------------AGGGC
NC_000004.12:c1 -------------------------------------------------------GGGGC
                                                                        *.*.

NC_000013.11:c3 CAAACAAGTGATTCACAAGGCTGAGATTCTGAATGAGAAGACAATTTGGGAATTTTATTT
NC_000001.11:33 CAACC-------------------------------------------------------
NC_000023.11:15 AAGAT-------------------------------------------------------
NC_000004.12:c1 AAAAT-------------------------------------------------------
                 *. .

NC_000013.11:c3 TATTTTATTTTATTTATTTATTTATTGAGACGGAGTCTCACTCTGTGGCCCAGGCTGAAG
NC_000001.11:33 ------------------------------------------------------------
NC_000023.11:15 ------------------------------------------------------------
NC_000004.12:c1 ------------------------------------------------------------

NC_000013.11:c3 TGCAGTGGCCTGATCACAGCTCACTGCAACCTCCGCCTCCCAGGTTCAAGTGATTCTCGT
NC_000001.11:33 ------------------------------------------------------------
NC_000023.11:15 ------------------------------------------------------------
NC_000004.12:c1 ------------------------------------------------------------

NC_000013.11:c3 GCCTCAGCCACCCAAGTAGCTGGGATTACAGGCATACGCCACCACACCTGACTAATTTTT
NC_000001.11:33 ------------------------------------------------------------
NC_000023.11:15 ------------------------------------------------------------
NC_000004.12:c1 ------------------------------------------------------------

NC_000013.11:c3 GTATTTTTAGTGGAAACAGGGTTTCACCATGTTGGCCAGGCTGGTCCCAAACTCCTGGCC
NC_000001.11:33 ------------------------------------------------------------
NC_000023.11:15 ------------------------------------------------------------
NC_000004.12:c1 ------------------------------------------------------------

NC_000013.11:c3 TCAAGTGAGCCGCCCGCCTCGGCCTCCCAATGTGCTGGGATTACAGGTGTGAGCCACTGC
NC_000001.11:33 ------------------------------------------------------------
NC_000023.11:15 ------------------------------------------------------------
NC_000004.12:c1 ------------------------------------------------------------
```

```
NC_000013.11:c3 GCCCAGCCTATTTTTATTTTTTTTGAGACGGAGTCTCACCCTGTTGCCCAGGCTGGAGTG
NC_000001.11:33 ------------------------------------------------------------
NC_000023.11:15 -----GTCCGCTTATGCCTTCTTTG-----------------------------------
NC_000004.12:c1 -----GTCCTCGTACGCCTTCTTCG-----------------------------------

NC_000013.11:c3 TAGTGGCCCGATCTTGGCTCACTGCAACCTCCACCTCCCAGGTTCAAGTGATTCTCGTGC
NC_000001.11:33 ------------------------------------------------------------
NC_000023.11:15 ------------------------------------------------------------
NC_000004.12:c1 ------------------------------------------------------------

NC_000013.11:c3 CTCAGCCTCCAGAGTAGCTGGGATTACAGGCATGTGCCACGATGCCCAGCTAGTTTTTGT
NC_000001.11:33 ------------------------------------------------------------
NC_000023.11:15 ------------------------TGCAGACATGC-------------------------
NC_000004.12:c1 ------------------------TGCAGACCTGC-------------------------

NC_000013.11:c3 ATTTTTAGTAGAGATGGGGTTTCACCGTGTTGGCCAGGCTGGTCTCGAACTCCTGAGCTC
NC_000001.11:33 ------------------------------------------------------------
NC_000023.11:15 ------------------------------------------------------------
NC_000004.12:c1 ------------------------------------------------------------

NC_000013.11:c3 CAGTGATCCTCCTGCCTTGGCCTCCCAAAGTGCTGGGATTATGGGCATGAGCCACTGTGC
NC_000001.11:33 ------------------------------------------------------------
NC_000023.11:15 ------------------------------------------------------------
NC_000004.12:c1 ------------------------------------------------------------

NC_000013.11:c3 CTGGCCAAGAAGACAATTTAGGGATATTTTAAATCCATTTATTTTGCCTATATTTTCCTT
NC_000001.11:33 ------------------------------------------------------------
NC_000023.11:15 ------------------------------------------------------------
NC_000004.12:c1 ------------------------------------------------------------

NC_000013.11:c3 TTTATGTATGCAAAAGAGTGTTAAATATTTTAAAAATCTAAGTCTAAAAGAGCAATTTCA
NC_000001.11:33 ------------------------------------------------------------
NC_000023.11:15 ------------------------------------------------------------
NC_000004.12:c1 ------------------------------------------------------------

NC_000013.11:c3 AAAAGTATAAAATGGTAAGTGATAAGGAAGCATCATCTCATAGTTTAGTTTAATTAAAGG
NC_000001.11:33 ------------------------------------------------------------
NC_000023.11:15 ------------------------------------------------------------
NC_000004.12:c1 ------------------------------------------------------------

NC_000013.11:c3 AACATTTTTCCTTTCTAAGGACTGCATAATATAATGCTGCATCAATGGGCTTACATTTGA
NC_000001.11:33 ------------------------------------------------------------
NC_000023.11:15 ------------------------------------------------------------
NC_000004.12:c1 ------------------------------------------------------------

NC_000013.11:c3 TCATATTTAGTATTTATTATGAAAGATAGTTCTTGAGGGTGGCAGGAACTGTTTTCTAAG
NC_000001.11:33 ------------------------------------------------------------
NC_000023.11:15 ------------------------------------------------------------
NC_000004.12:c1 ------------------------------------------------------------

NC_000013.11:c3 TTCAATGTGACATCTTTAGCTGCTATGTTGATTTTTTTTTTTTTAATTGATGGAGTCTTG
NC_000001.11:33 ------------------------------------------------------------
NC_000023.11:15 ------------------------------------------------------------
```

NC_000004.12:c1 --

NC_000013.11:c3 CTTTGTCGCCCAGGCTGGAGTGCAGTGGTGCGATCTCAGCTCACTGCAAGCTCCGTCTCC
NC_000001.11:33 --
NC_000023.11:15 --
NC_000004.12:c1 --

NC_000013.11:c3 TGGGTTCACGCCATTCTCCTGCCTCAGCCTCCCAAGTAGCTGGGACTACAGGCGCCCGCC
NC_000001.11:33 --
NC_000023.11:15 --
NC_000004.12:c1 --

NC_000013.11:c3 ACCACGCCTGGCTAATTTTTTTTTTTGTATTTTTAGTAGAGACAGGGTTTCACTGTGTTA
NC_000001.11:33 --
NC_000023.11:15 --
NC_000004.12:c1 --

NC_000013.11:c3 GCCGGGATGGTCTCGATCTCCTGACCTCGTGATCCGCCCACCTTGGCCTCCCAAAGTGCT
NC_000001.11:33 --
NC_000023.11:15 --
NC_000004.12:c1 --

NC_000013.11:c3 GGGATTACAGGCGTGAACCACCGCGCCCGGCCTTTTACTTTTTTTTTTTTTTTGAGACAG
NC_000001.11:33 --
NC_000023.11:15 --
NC_000004.12:c1 --

NC_000013.11:c3 AGTGTCACTCTGTCACTCAGGCTAGAGTGCAGTGGTGTGATCTTGGCTCACTGCAACCTC
NC_000001.11:33 --
NC_000023.11:15 --
NC_000004.12:c1 --

NC_000013.11:c3 CATCTCCTGGGTTCAAGCGATTCTTCTGCCTCAGCCTCCCGAGTAGCTGGGACTACAGAT
NC_000001.11:33 --
NC_000023.11:15 --
NC_000004.12:c1 --

NC_000013.11:c3 GTGTGCCACCACGTCTCCCTAATTTTTGTATTTTTAATAGAGACGGGGTTTCACCATGTT
NC_000001.11:33 --
NC_000023.11:15 --
NC_000004.12:c1 --

NC_000013.11:c3 GGCCAGGCTGGTCTTGAACTGCTAGCCTCAAATGATCCACCCGCCTCGGCCTCCCAAAGT
NC_000001.11:33 --
NC_000023.11:15 --
NC_000004.12:c1 --

NC_000013.11:c3 GCAGGGATTACAGGCATGAGCCACCGCATCCAGCCTGAAAATTATTATAGCATAATTCTC
NC_000001.11:33 --
NC_000023.11:15 --
NC_000004.12:c1 --

NC_000013.11:c3 ACTACTCAAGCATTGAAATTTGTTGACACTTATTCCAGGACTGTCTAGAGAATGTTTATT

```
NC_000001.11:33 ------------------------------------------------------------
NC_000023.11:15 ------------------------------------------------------------
NC_000004.12:c1 ------------------------------------------------------------


NC_000013.11:c3 TGGGCTGGGCACAGTGGCTCACACCCATAATCCCAACCCCTTGGGAGGCCAAGGCAGGAG
NC_000001.11:33 -------------------------------TCTGGCCCCTTGGGAGAAAGAGCCAGGGG
NC_000023.11:15 -------------------------------------------AGAGAAGAACATAAGAA
NC_000004.12:c1 -------------------------------------------CGGGAAGAGCACAAGAA
                                                            *.*.  ..  .*.*..

NC_000013.11:c3 GATCATTTGAGCCCAGGAATTCAAGACCAGCCCCCATCTCTACAAAAATAAAAATAAAAA
NC_000001.11:33 GA----------------------------------------------------------
NC_000023.11:15 GA----------------------------------------------------------
NC_000004.12:c1 GA----------------------------------------------------------
                **

NC_000013.11:c3 TAAAATAAAATAAAAAATTAGCTGGGTGTGGTGGCATGTAGCTTTTTTTTTTTTTTTTTG
NC_000001.11:33 ------------------------------------------------------------
NC_000023.11:15 ------------------------------------------------------------
NC_000004.12:c1 ------------------------------------------------------------


NC_000013.11:c3 TGGTCCCAGCTACTTGGGAGGCTGAGGTGGGAGGCTCGCTTGAGCCTGGGAAGTTGAGGC
NC_000001.11:33 ------------------------------------------------------------
NC_000023.11:15 ------------------------------------------------------------
NC_000004.12:c1 ------------------------------------------------------------


NC_000013.11:c3 TGCAGTGAGCCATGCTTGTGGACACTGTGCTCCATCCTAGGCAAGAGAGTGAGACCCTGA
NC_000001.11:33 ------------------------------------------------------------
NC_000023.11:15 ------------------------------------------------------------
NC_000004.12:c1 ------------------------------------------------------------


NC_000013.11:c3 ATCAAAAAAAAAAAGGTTTCTATGAATGTTAAGAGCTGTGTGCTATCTATTGCTCCCTTA
NC_000001.11:33 ------------------------------------------------------------
NC_000023.11:15 ----AAAACCCAGAGGTCCCTGTCAATTTTGCGGA-------------------------
NC_000004.12:c1 ----AACACCCGGACTCTTCCGTCAATTTCGCGGA-------------------------


NC_000013.11:c3 CTCTGTGTCAGGCACTGTGCTAAATGCTCTTCAAGCATTTCTCATTGGCACCTAACAATA
NC_000001.11:33 ------------------------------------------------------------
NC_000023.11:15 ------------------------------------------------------------
NC_000004.12:c1 ------------------------------------------------------------


NC_000013.11:c3 ATAATATGTAGAAGGTATAATACAATTTCTCCTATTTTACAGAAGAGGAAACTGAGGTTA
NC_000001.11:33 ------------------------------------------AAAAGGCTACCTAGGCTG
NC_000023.11:15 ---------------------------------ATTTTCCAAGAAGTGCTCTGAGAGGTG
NC_000004.12:c1 ---------------------------------ATTCTCCAAGAAGTGTTCGGAGAGATG
                                                          .*.. *      ..* *.

NC_000013.11:c3 AGGAATATTAAATAACTTTCCCAGAGTCCTTCAGCAACTAAATGACAGAGCTAGGACCTG
NC_000001.11:33 AGAAGTG----------TCCCCAGAGT---------------------------------
NC_000023.11:15 GAAGGTATT------TTTCTTTGGTACCCTTCA---------------------------
NC_000004.12:c1 GAAGGTG-----------------------------------------------------
                .....*.

NC_000013.11:c3 GATCTGTCTGACTCCAAAAGATCATGCCCTCGATGTTCTGCCAGGACACCTTTATGGCTG
NC_000001.11:33 ------------------------------------------------------------
NC_000023.11:15 ------------------------------------------------------------
NC_000004.12:c1 ------------------------------------------------------------
```

```
NC_000013.11:c3 ATCTAAATTGGGTCTTGAAAATCACAGACAATTCAACACAAAGGGAAATAGGAAAAGACA
NC_000001.11:33 ------------------------------------------------------------
NC_000023.11:15 ------------------------------------------------------------
NC_000004.12:c1 ------------------------------------------------------------

NC_000013.11:c3 GCTGGATATAGAAGTGTTTTGTTTTGCTTGTTTCAGACAAGGTCTCTGTTGTCCAGGCTG
NC_000001.11:33 ------------------------------------------------------------
NC_000023.11:15 ------------------------------------------------------------
NC_000004.12:c1 ------------------------------------------------------------

NC_000013.11:c3 GAGTACAGTAGCGTGAACACAGCTCACGGCAGCCTTGAATTCCTGGGCTCAAGTAACCTT
NC_000001.11:33 ------------------------------------------------------------
NC_000023.11:15 ------------------------------------------------------------
NC_000004.12:c1 ------------------------------------------------------------

NC_000013.11:c3 CCTACCTTGGCATCTCAAGTAGATGGGACTATGGACATGTGCCACCACACCCAGCTATTT
NC_000001.11:33 ------------------------------------------------------------
NC_000023.11:15 ------------------------------------------------------------
NC_000004.12:c1 ------------------------------------------------------------

NC_000013.11:c3 TTTTATTTTTATTTTTAGTAGAGATGAAGTCTCGCTATCTTGCCCAGGCTGGTCTTAAAT
NC_000001.11:33 ------------------------------------------------------------
NC_000023.11:15 ------------------------------------------------------------
NC_000004.12:c1 ------------------------------------------------------------

NC_000013.11:c3 TCCTAGGCTCAAGCGATCCTCCCACCTCAGCCTCCCATAAGTGTTCGGATTACAGGCGTG
NC_000001.11:33 ------------------------------------------------------------
NC_000023.11:15 ------------------------------------------------------------
NC_000004.12:c1 ------------------------------------------------------------

NC_000013.11:c3 AGCCACCACACCCAGCCAGATATAAGAGTTAGAAGGCAGTTGCAGACTTCTGGTCTTTCT
NC_000001.11:33 ------------------------------------------------------------
NC_000023.11:15 ------------------------------------------------------------
NC_000004.12:c1 ------------------------------------------------------------

NC_000013.11:c3 CTGACTACACTAACAGTCAACTTGGCCAGCTCTGGGTTTTCCATGTTACTCGATGGAAGT
NC_000001.11:33 ------------------------------------------------------------
NC_000023.11:15 ------------------------------------------------------------
NC_000004.12:c1 ------------------------------------------------------------

NC_000013.11:c3 CAAAGCAAACACAAGTGAGAATCTACAGTTATAGTTTCCAGTGTTCTCCTGTAAATTCTG
NC_000001.11:33 ------------------------------------------------------------
NC_000023.11:15 ------------------------------------------------------------
NC_000004.12:c1 ------------------------------------------------------------

NC_000013.11:c3 CTGGTGAGATATAAAATAAAGTTTCTTTCACATTGATTGTCCCAGCATTCTGGGTTTTTT
NC_000001.11:33 ------------------------------------------------------------
NC_000023.11:15 ------------------------------------------------------------
NC_000004.12:c1 ------------------------------------------------------------

NC_000013.11:c3 TGTTTTCTGATTTTTGGTTTTTGTATGCTGAAATACCCGTGGAGAGAACTACAGAGGATT
NC_000001.11:33 ------------------------------------------------------------
NC_000023.11:15 ------------------------------------------------------------
```

```
NC_000004.12:c1 ------------------------------------------------------------

NC_000013.11:c3 TGAGATGCAGAACCAGAACAAGCATAACCTACAGCAAAATCCCAAATCACAGAACTTCAG
NC_000001.11:33 ------------------------------------------------------------
NC_000023.11:15 ------------------------------------------------------------
NC_000004.12:c1 ------------------------------------------------------------

NC_000013.11:c3 AGCAGAAGGCTCTCAGAGGCCAGGCGCAGTGGCTCATGCCTGTAATCCCAATACTTTGAG
NC_000001.11:33 ------------------------------------------------------------
NC_000023.11:15 ------------------------------------------------------------
NC_000004.12:c1 ------------------------------------------------------------

NC_000013.11:c3 AGGCCAAGGCGGGCGGATCATTTGAGGTCAGGAGTTCGAGCCTGGCCAACATGGTGAAAC
NC_000001.11:33 ------------------------------------------------------------
NC_000023.11:15 ------------------------------------------------------------
NC_000004.12:c1 ------------------------------------------------------------

NC_000013.11:c3 CCCATCTCTACTAAAAATACAAAAATTAGCCAGGCATGGTGTAGTCCCAGCTACTCTGGA
NC_000001.11:33 ------------------------------------------------------------
NC_000023.11:15 ------------------------------------------------------------
NC_000004.12:c1 ------------------------------------------------------------

NC_000013.11:c3 GGCTGAGGCAGGAGAATCGCTTGAACCCTGGAGGTGGAGGTTGCAGTGAGCTGAGACTGT
NC_000001.11:33 ------------------------------------------------------------
NC_000023.11:15 ------------------------------------------------------------
NC_000004.12:c1 -----AGGCAGGAGA---------------------------------------------

NC_000013.11:c3 GCCACTGCACTCCAGTCTGGGTGACAGAGCGAGACTCCGTCTCAAAACACAAACAAAAAG
NC_000001.11:33 ---------------------------------AATCCGTTCC-----------------
NC_000023.11:15 ---------------------------------AACTAGTCTT-----------------
NC_000004.12:c1 -------------------------------GGGACGGAGCTC-----------------
                                                 . .  . ...

NC_000013.11:c3 GCTCTGAGAAATTGGTACACATTAAAACCACCTATGCAGCTTTTCCCAGACAGCTGTTGA
NC_000001.11:33 ------------------------------------------------------------
NC_000023.11:15 ------------------------------------------------------------
NC_000004.12:c1 ------------------------------------------------------------

NC_000013.11:c3 ATGAGAATCTCAGAGCAGGTTGGTATAAGAACTGCATTCTCAGTGGTTCTCAAAATAGGC
NC_000001.11:33 ------------------------------------------------------------
NC_000023.11:15 ------------------------------------------------------------
NC_000004.12:c1 ------------------------------------------------------------

NC_000013.11:c3 TTCCTGGACAGCAGCTTCACATCACCTGGGAACTTGTTAGAAACGCATATTTTGGGGGTC
NC_000001.11:33 ------------------------------------------------------------
NC_000023.11:15 ------------------------------------------------------------
NC_000004.12:c1 ------------------------------------------------------------

NC_000013.11:c3 TCACCCCAGACCATTGGATCAGAAATTCTGGTGGCAGGGGCCAGCAACCTGTACTTTACC
NC_000001.11:33 ------------------------------------------------------------
NC_000023.11:15 ------------------------------------------------------------
NC_000004.12:c1 ------------------------------------------------------------

NC_000013.11:c3 AAGCCTTCCAGGAGGTTCTAAAACCTGCTAAAGTTTGAGAACCACTGAACTAGACCAAGA
```

```
NC_000001.11:33 ------------------------------------------------------------
NC_000023.11:15 ------------------------------------------------------------
NC_000004.12:c1 ---------AGGGGGTGC------------------------------------------


NC_000013.11:c3 ATTTGGATAGGATTCTAAGGTCATTCCTTCTTTTTGGAAGTACATCACAGCTCTATTTCA
NC_000001.11:33 ------------------------------------------------------------
NC_000023.11:15 ------------------------------------------------------------
NC_000004.12:c1 ------------------------------------------------------------


NC_000013.11:c3 AATTCAATTTCTTCACATTCGAAATACTTATTTTCATGTTTTATATACTTGGAACTTTTT
NC_000001.11:33 ------------------------------------------------------------
NC_000023.11:15 -AGTTGGGTTTCTTACTTTGGGGTTACTTACCTTCAGATTTT------------------
NC_000004.12:c1 -AGGTGTGGTTTTCGGCTAGGAGGGCCTTAGG----------------------------


NC_000013.11:c3 TTTAGCATTTATGTATTTAAAAGCACAAGAACCCTAGCCTAAAATTCTGAACCTTATTAT
NC_000001.11:33 ------------------------------------------------------------
NC_000023.11:15 ------------------------------------------------------------
NC_000004.12:c1 ------------------------------------------------------------


NC_000013.11:c3 TATTATTTTGAGACTGGGTCTCGTTCTGTCACCCAGGCTGGAGGGCAATGGTGTGATCAC
NC_000001.11:33 ------------------------------------------------------------
NC_000023.11:15 ------------------------------------------------------------
NC_000004.12:c1 ------------------------------------------------------------


NC_000013.11:c3 AGCTCATTGCAGCCTCGATCTCCCAGTCTCAAGTGATCCTCTCACCTTAGCCTTCCAAGT
NC_000001.11:33 ------------------------------------------------------------
NC_000023.11:15 ---------------------------------------------------CTCCCAGAT
NC_000004.12:c1 -------------------------------------------------------CAGGT


NC_000013.11:c3 AGCTGGGACTACAGGAGTATACCACCACTCCTGGCTAATTTTTTATTTTTTGTATAGACA
NC_000001.11:33 ------------------------------------------------------------
NC_000023.11:15 AGCTG-------------------------------------------------------
NC_000004.12:c1 AGTAGG------------------------------------------------------


NC_000013.11:c3 GGGTCTCACTATGGTGCTCAGGCTGGTCTCAAACTCATAGACTCAGGCAATCCTCCTGCC
NC_000001.11:33 ------------------------------------------------------------
NC_000023.11:15 ------------------------------------------------------------
NC_000004.12:c1 ------------------------------CAACTCATGAGCTTCAAC------------


NC_000013.11:c3 TCAGCCTCCCAAAGTGCTGGGATTACAGGCAGGAGGCACTATGCCCAGCCCTGATTCTAT
NC_000001.11:33 ------------------------------------------------------------
NC_000023.11:15 ------------------------------------------------------------
NC_000004.12:c1 ------------------------------------------------------------


NC_000013.11:c3 TTTTCTACCTCCTCCTTAACATCGACTAAAAATATATTTAAAAGTTTGTCAGTTTTAAGT
NC_000001.11:33 ------------------------------------------------------------
NC_000023.11:15 ---CTTGCTTCCTCTTTTACTTTTACTTAGAAT-------CATTTTTGCTTGTCTTAAGA
NC_000004.12:c1 ---TTTGCCACCCCAGTGACTC---------------------ACCTGAATATCTTAAGA


NC_000013.11:c3 GTTTTCTGCATTAAATTATAAAGTATTAGATGACAAAACAACCTCTTATATAAAAAAAAT
NC_000001.11:33 ------------------------------------------------------------
NC_000023.11:15 ATTTTGTGTGT-------------------------------------------------
NC_000004.12:c1 GTTCCAGGCAG-------------------------------------------------
```

```
NC_000013.11:c3 TCACTGTGATAATGCACAAATACATATATGGTATGGCTGAAACTATTCTAGAAAATTCCT
NC_000001.11:33 ------------------------------------------------------------
NC_000023.11:15 ------------------------------------------------------------
NC_000004.12:c1 ------------------------------------------------------------

NC_000013.11:c3 TGGAACATTTACTTTGCTGTATGGGTATTTTGTTAAAATTAATACAGTTGTTAAAAATCA
NC_000001.11:33 ------------------------------------------------------------
NC_000023.11:15 ------------------------------------------------------------
NC_000004.12:c1 ------------------------------------------------------------

NC_000013.11:c3 CTTTTCAAATTTGTTGACTCAAGTAAAAAAAAATTAGTGAAAAGTTACTATAGTCCATAA
NC_000001.11:33 ------------------------------------------------------------
NC_000023.11:15 ------------------------------------------------------------
NC_000004.12:c1 ------------------------------------------------------------

NC_000013.11:c3 GGAAAGGAGGAAGACACTTTACCAGCTCTTGAATATCTTGAATCAATTTAACTTCTGAAA
NC_000001.11:33 ------------------------------------------------------------
NC_000023.11:15 ------------------------------------------------------------
NC_000004.12:c1 ------------------------------------------------------------

NC_000013.11:c3 TATTCCTTGTAGTCCTACTATTTTGATTTAATACTTGGTCAGGAGGGAGGAATTTGTTCA
NC_000001.11:33 ------------------------------------------------------------
NC_000023.11:15 -----------GTGCTTTTATTTT------------------------------------
NC_000004.12:c1 ----------------ACGATTTT------------------------------------

NC_000013.11:c3 GGCCAGCATTTCCCAAATTGCATTTCTGCAAGAGGTCAAAAATTATTTCTCAAAGAAAGA
NC_000001.11:33 ------------------------------------------------------------
NC_000023.11:15 ------------------------------------------------------------
NC_000004.12:c1 ------------------------------------------------------------

NC_000013.11:c3 TTCTGTCGTAAGTATATTTGGGAGATGCTGTATTAAAAAAATTAAATGGGTAGATTCACT
NC_000001.11:33 ------------------------------------------------------------
NC_000023.11:15 -----------------------------------------------------------T
NC_000004.12:c1 ------------------------------------------------------------

NC_000013.11:c3 TCATGACTTCATAGAGTTTTTAATACGGTAATATGGACTGTAAATCTCCGGGGGTGAGGG
NC_000001.11:33 ------------------------------------------------------------
NC_000023.11:15 TTAAGGCCCTGCACAGGTTTCA--------------------------------------
NC_000004.12:c1 -------------AGGTTTTA---------------------------------------

NC_000013.11:c3 GGGAAGGTGGAGTGTTGTATGTAGCATTTGCCAGACTTTTTAAATAAAAATTTTTAGAAA
NC_000001.11:33 ------------------------------------------------------------
NC_000023.11:15 ----------------------GGCCTTTACC----------------------------
NC_000004.12:c1 ------------------------------------------------------------

NC_000013.11:c3 CCAACAAACCATTGTTTCTAAGGACCTCGGAAGTAGGTTTGTTATATAGAATCTTTCTTT
NC_000001.11:33 ------------------------------------------------------------
NC_000023.11:15 ------------------------------------------------------------
NC_000004.12:c1 ------------------------------------------------------------

NC_000013.11:c3 GTAATGGTAATTCATGCAAAGAAATTTTACCTGTAAACTCTTTTGATGATTACCTTAGTT
NC_000001.11:33 ----------------------------------------------------------TT
NC_000023.11:15 --------------------------------------------------TACCCCCTTT
```

```
NC_000004.12:c1 ----------------------------------------------------------CT
                                                                          .*

NC_000013.11:c3 TGTAAAATATTCTACTAGCTCATTTTTGGTGTCCTGAAACTACAATGTAGCCCCTGGGTG
NC_000001.11:33 TGCGAGACCCCCTACTAGCCCA--------------------------------------
NC_000023.11:15 TGCAAGTGGTTCTAGCAACTGCT-------------------------------------
NC_000004.12:c1 TATATAAGACTTCGAAAGCAAA--------------------------------------
                *... .   .....  *.*

NC_000013.11:c3 TATTAAATGTTTCAAGGTGCATCTCTGCTTTAAATAATTCCTTTCATGTTTTCTAAAAAC
NC_000001.11:33 ------------------------------------------------------------
NC_000023.11:15 ------------------------------------------------------------
NC_000004.12:c1 ------------------------------------------------------------


NC_000013.11:c3 ATCCCTTCTAGAATAATGTGGGCTTTTTTTTTTTTTTTTAGCAATTCAATAAATATAAAA
NC_000001.11:33 ------------------------------------------------------------
NC_000023.11:15 ------------------------------------------AGTTTAATCACAAGAAAC
NC_000004.12:c1 ------------------------------------------ATGCTGTTAAGGAAAATG


NC_000013.11:c3 TGAGCATGACAGATATTGTTCAAATTAAAATCTTTGTTAAAATAGTTTTTAATGTTGATA
NC_000001.11:33 ------------------------------------------------------------
NC_000023.11:15 TGAATA------------------------------------------------------
NC_000004.12:c1 TGGTCCTTAAAGATG---------------------------------------------


NC_000013.11:c3 ATAGGCAGAAAAGGCCAGCTGGCATTATGACCTTACTGGCGATAAGATAAAGTTATTTCA
NC_000001.11:33 ------------------------------------------------------------
NC_000023.11:15 ------------------------------------------------------------
NC_000004.12:c1 ------------------------------------------------------------


NC_000013.11:c3 ATTATTAATGTTTTCTCAGTGAGTATTTATTGAATGTAATGTTAAATTACGTTTTGTTCA
NC_000001.11:33 ------------------------------------------------------------
NC_000023.11:15 -----------------GGTATGTGTTCACTGCAT-------------------------
NC_000004.12:c1 -----------------ATCTTGTCGTCTTTGGATGTT----------------------


NC_000013.11:c3 ATAGTGAAAGCACTGAAGAGAAGTACTATTAGTCTTCTATTGTTGCATGACAAAAATGAT
NC_000001.11:33 ------------------------------------------------------------
NC_000023.11:15 ------------------------------------------------------------
NC_000004.12:c1 ------------------------------------------------------------


NC_000013.11:c3 CACAAACAGAGGCTTCAAGCAGCACCCACGTATTAGCACACAGTTGTGTAGGTCAGAAGT
NC_000001.11:33 ------------------------------------------------------------
NC_000023.11:15 ------CGAGGGATTTAA------------------------------------------
NC_000004.12:c1 ------TATAGGTAACAA------------------------------------------


NC_000013.11:c3 TCAGTTTGGCATAGTCAGGTTCTCTGCTCAAGGGAACACAAGCCCAAATGAAGGTGTTGG
NC_000001.11:33 ------------------------------------------------------------
NC_000023.11:15 ------------------------------------------------------------
NC_000004.12:c1 ------------------------------------------------------------


NC_000013.11:c3 CCAAATTGAGCTCACATCCACTTCCAAGCTCATTCAGGTTCTGTTGGCAGAATCCACTTC
NC_000001.11:33 ------------------------------------------------------------
NC_000023.11:15 ------------------------------------------------------------
NC_000004.12:c1 ------------------------------------------------------------


NC_000013.11:c3 CTTGTGGCTCTGTAACTGAGGTCCCACTTTCTTGCTGATCCATAGTAGGGCACTGCTGTC
```

```
NC_000001.11:33 ------------------------------------------------------------
NC_000023.11:15 ------------------------------------------------------------
NC_000004.12:c1 ------------------------------------------------------------

NC_000013.11:c3 TGGTTCTAAAAGCTGCTTTCAGGTCCTTTTCCTGTGACCCCCAACACCTTCAAGCCAGCA
NC_000001.11:33 ------------------------------------------------------------
NC_000023.11:15 ------------------------------------------------------------
NC_000004.12:c1 --------------------------TTTTGCTGTATTTT--------------------

NC_000013.11:c3 AGGGCGAGTCCTAGTCTTCTTGTGCTTTGAATCTCTGATCCTCCATCTGCCTCCAGCGCT
NC_000001.11:33 ------------------------------------------------------------
NC_000023.11:15 ----CGAGTCCTGTTC--------------------------------------------
NC_000004.12:c1 ----TGGCTCTTATTT--------------------------------------------

NC_000013.11:c3 CTGCTTTTAATGATTTAGTATGATGAGGGTCAGGCCCACCTGGGTAATCTCCTGTAGCAT
NC_000001.11:33 ------------------------------------------------------------
NC_000023.11:15 ------------------------------------------------------------
NC_000004.12:c1 ------------------TATG--------------------------------------

NC_000013.11:c3 TAACTTCAGCTGTGCCATCTAACACAACCTAATCATGGGAGTCAACTCTCGTCATACTCA
NC_000001.11:33 ------------------------------------------------------------
NC_000023.11:15 ------------------------------------------------------------
NC_000004.12:c1 ------------------------------------------------------------

NC_000013.11:c3 CTTACTCTGCCTGTGTTCAAAGGAGAAAGGATTCTCTAAAGATGAGGCTTAGGGGAGGTC
NC_000001.11:33 ------------------------------------------------------------
NC_000023.11:15 ----------------------------------TTTGCAGACGATGTCCGGGAAAGA--
NC_000004.12:c1 ----------------------------------TCCACAGACCATGTCTGCAAAGGA--

NC_000013.11:c3 ATCGAGGATCATCTTAGAGTTCCGCCTACCACAAAGTGTAATACACATCCCAGTACTCCC
NC_000001.11:33 ------------------------------------------------------------
NC_000023.11:15 ------------------------------------------------------------
NC_000004.12:c1 ------------------------------------------------------------

NC_000013.11:c3 AAGTTGAACAGTTAAAAGATGATAGTTAGGCATTGTGACACACACCTGTACTCCCAGCTA
NC_000001.11:33 ------------------------------------------------------------
NC_000023.11:15 ------------------------------------------------------------
NC_000004.12:c1 ------------------------------------------------------------

NC_000013.11:c3 CTTGGGAGTCTGAGGCGGGAGGATGGCTTGAGCCCAGGAGTTTGAGGCTGCAGTGAGCTA
NC_000001.11:33 ------------------------------------------------------------
NC_000023.11:15 ------------------------------------------------------------
NC_000004.12:c1 ------------------------------------------------------------

NC_000013.11:c3 TGATGGTGCTGATGAATAGCCACTGTACTGCAGCCTGAGGAACACAGCCAGACCCTCTCT
NC_000001.11:33 ------------------------------------------------------------
NC_000023.11:15 ------------------------------------------------------------
NC_000004.12:c1 ------------------------------------------------------------

NC_000013.11:c3 CAAAAAAACTAAACAAAAAGTGAGAAATAGATACACACATATGACCAGAATGTACAGTTT
NC_000001.11:33 ------------------------------------------------------------
NC_000023.11:15 ------------------------------------------------------------
NC_000004.12:c1 ------------------------------------------------------------
```

```
NC_000013.11:c3 AAAATGAATACTACAATCAGTGCTGCAGTCGTTTATAAAAAGGTGATTGTCAATGGAAAA
NC_000001.11:33 ------------------------------------------------------------
NC_000023.11:15 ------------------------------------------------------------
NC_000004.12:c1 ------------------------------------------------------------


NC_000013.11:c3 AGATTGATTAGAAAGGTCACCTAGAGTTTTTAAAAATTGCCAGGTACGGTGGCACATGCC
NC_000001.11:33 ------------------------------------------------------------
NC_000023.11:15 ------------------------------------------------------------
NC_000004.12:c1 ------------------------------------------------------------


NC_000013.11:c3 TGTAGTCCTAACTACATGGGAGGCTGAGGTGGGAGGATCACTTGAGCCCAGGAGTTTGAG
NC_000001.11:33 ------------------------------------------------------------
NC_000023.11:15 --------------------------------------------------------GAAA
NC_000004.12:c1 --------------------------------------------------------GAAG


NC_000013.11:c3 TCCAAACTCGGTAACATAGTGAGACCCTATCTCTTAAAAAAAAAAAAAAAAAAGATTGAA
NC_000001.11:33 ------------------------------------------------------------
NC_000023.11:15 TCTAAATTTGATGAAATGGCAA--------------------------------------
NC_000004.12:c1 TCGAAGTTTGAAGATATGGCAA--------------------------------------


NC_000013.11:c3 CTAGACCCTGAGGGATAATAATAGCAATGGCTAGCATTGATTGAGCTCTAAACCCCTACT
NC_000001.11:33 ------------------------------------------------------------
NC_000023.11:15 ------------------------------------------------------------
NC_000004.12:c1 ------------------------------------------------------------


NC_000013.11:c3 GCAGCTCAGTACAGCTCAGTACTAGGGGTTTATAACTCAATCAATGCTAGCCATTGCTAT
NC_000001.11:33 ------------------------------------------------------------
NC_000023.11:15 ------------------------------------------------------------
NC_000004.12:c1 ------------------------------------------------------------


NC_000013.11:c3 TATTATCATGAGGTAATGTGCATCCTCATTCAATCATTACAAAAATCCTACGGTTACTCC
NC_000001.11:33 ------------------------------------------------------------
NC_000023.11:15 ------------------------------------------------------------
NC_000004.12:c1 ------------------------------------------------------------


NC_000013.11:c3 CATTTTATAGATAAGAACCAGTTAGATGACTTCCATAACAGTCCAGATGAGAGCTATCAA
NC_000001.11:33 ---------------------------GACTTCCTTGATGG-------------------
NC_000023.11:15 -------------------AGGCAGATAAAGTGCGCTATGATCGGGAAATGAAGGATTAT
NC_000004.12:c1 -------------------AAAGTGACAAAGCTCGCTATGACAGGGAGATGAAAAATTAC
                                           .*  . * . *...

NC_000013.11:c3 GGGCCTTAGATGAGAGGTGGACCATATGAAGAGAGAGTGGATGGATGAAATTTGGGAAGG
NC_000001.11:33 ------------------------------------------------------------
NC_000023.11:15 GGAC--------------------------------------------------------
NC_000004.12:c1 GTTC--------------------------------------------------------


NC_000013.11:c3 CCAGATGCAGTGGTTCACACCTGTAGTCCCAGCACTTTGGGAGCTGAGGCGAGAGGATCA
NC_000001.11:33 -----------------CACCT--------------------------------------
NC_000023.11:15 -----------------CAGCT-------------------AAGGGAGGCAAGAAGA---
NC_000004.12:c1 -----------------CTCCC-------------------AAAGGTGATAAGAAGG---
                                 *  *.

NC_000013.11:c3 CTTGAGGCCAAGAGTTTGAGACCAGCCTAGGCAACATAGAGACACCCCATAGACCCCATT
NC_000001.11:33 ----------------------------------------GATATCTTATAGGCCCC---
NC_000023.11:15 -------------------------------------AGAAGGATCCTAATGCTCCC---
```

```
NC_000004.12:c1 -------------------------------GGAAGAAAAAGGACCCCAATGCTCCT---
                                        .. *.*..*  * .**.

NC_000013.11:c3 TCTACAAAGACAAAAAGAAATCTGGTGACTCGCTAGATGCATAGTATTGCTATGGTTTGA
NC_000001.11:33 ------------------------------------------------------------
NC_000023.11:15 ------------------------------------------------------------
NC_000004.12:c1 ------------------------------------------------------------


NC_000013.11:c3 ATGTTTGTCACCTCCAAAACTCATGTTGAAACTTGATCCCCACTGCAACAGTATTGAGAA
NC_000001.11:33 ------------------------------------------------------------
NC_000023.11:15 ------------------------------------------------------------
NC_000004.12:c1 ------------------------------------------------------------


NC_000013.11:c3 GTGGGGCCTTTGGGAGGTGATTGGATCATGAGAAGCCTTGTGAATGGGTTAATCCATACA
NC_000001.11:33 ------------------------------------------------------------
NC_000023.11:15 ------------------------------------------------------------
NC_000004.12:c1 ------------------------------------------------------------


NC_000013.11:c3 TGGATTAATGGGTTATCATGAGAGTGGATTAGTTATCAAGAGAGTGGGTCAAGAAAACTT
NC_000001.11:33 ------------------------------------------------------------
NC_000023.11:15 ------------------------------------------------------------
NC_000004.12:c1 ------------------------------------------------------------


NC_000013.11:c3 GGCATTGGCCAAATTAAAGTTGAGTTATCTGTATTACATCCAAGTAGAGATGACCAGTAG
NC_000001.11:33 ------------------------------------------------------------
NC_000023.11:15 ------------------------------------------------------------
NC_000004.12:c1 ------------------------------------------------------------


NC_000013.11:c3 AAACTTGGACATACAGGCCTGAGGCTCAGCAGAGAAGTCTTCCCTGAGAGGCAGATTTTT
NC_000001.11:33 ------------------------------------------------------------
NC_000023.11:15 ------------------------------------------------------------
NC_000004.12:c1 ------------------------------------------------------------


NC_000013.11:c3 GGAATCTCAGTGCTCTGGAATGATTTCAGCACTGTTGTATTTAAGTTCCCACTACCTTAT
NC_000001.11:33 ------------------------------------------------------------
NC_000023.11:15 ------------------------------------------------------------
NC_000004.12:c1 ------------------------------------------------------------


NC_000013.11:c3 GCTAGGCCCTAGAAATACAAATATGAAGATACAGATCGTGCCATAGAGAAGATGTGAACT
NC_000001.11:33 -------------------------------CAAATCCTGCCACACA-------------
NC_000023.11:15 -------------------------------AAAAGGCCACCGTAAG-------------
NC_000004.12:c1 -------------------------------AAAAGGCCACCGTAAG-------------
                                *.*   ..**..* .

NC_000013.11:c3 CACACACACCTGTGCAGATGTTTTGGTGTTACAGTGAGCACATGCATAGCATACAATGAG
NC_000001.11:33 ------------------------------------------------------------
NC_000023.11:15 ------------------------------------------------------------
NC_000004.12:c1 ------------------------------------------------------------


NC_000013.11:c3 AGCATTCACATGGGGTGACTTCCTGAAGCAGAGGACTCCAGGCAGATGAGAGTAAAACCA
NC_000001.11:33 ------------------------------------------------------------
NC_000023.11:15 ------------------------------------------------------------
NC_000004.12:c1 ------------------------------------------------------------


NC_000013.11:c3 GGAGGGAAAAATTGAAGAGACCTTCTACAGACCACATGAAAAGTGACTAAAGAGCCAAAA
```

```
NC_000001.11:33 ------------------------------------------------------------
NC_000023.11:15 ------------------------------------------------------------
NC_000004.12:c1 ------------------------------------------------------------

NC_000013.11:c3 GCTGGGGTGGCCAGAGAGAAAATGGAATCCAGGTTAAAATCCTGTTGTTTAGTCATGTGG
NC_000001.11:33 ------------------------------------------------------------
NC_000023.11:15 ------------------------------------------------------------
NC_000004.12:c1 ------------------------------------------------------------

NC_000013.11:c3 GGTTTTTTGGGTTTTTGTTTTTTGTTTTTGTTTTTTTTGAGATGCAGTCTCACTCTGATG
NC_000001.11:33 ------------------------------------------------------------
NC_000023.11:15 ------------------------------------------------------------
NC_000004.12:c1 ------------------------------------------------------------

NC_000013.11:c3 CCCAGGCTGGAGTGCAGTGCAGTGGTGTGATCTCAGCTCACTACAACCTCTGCCTCCCAG
NC_000001.11:33 ------------------------------------------------------------
NC_000023.11:15 ------------------------------------------------------------
NC_000004.12:c1 ------------------------------------------------------------

NC_000013.11:c3 GTTCAAGCAATTCTCCTGCCTCAGCCTCCTGAGTAACTGGGACTACAGGTGTCCACCATC
NC_000001.11:33 ------------------------------------------------------------
NC_000023.11:15 ------------------------------------------------------------
NC_000004.12:c1 ------------------------------------------------------------

NC_000013.11:c3 ACGCCTTGCTAATTTTTGTATTTGTATTAGAGATGGGGTTTCACCACGTTGGCCAGGCTG
NC_000001.11:33 ------------------------------------------------------------
NC_000023.11:15 ------------------------------------------------------------
NC_000004.12:c1 ------------------------------------------------------------

NC_000013.11:c3 GTCTTGAACTCCTGACCTCAAATGATTCGCCTACCTTGGCCTCCCAAAGTGCTGGGATTA
NC_000001.11:33 ------------------------------------------------------------
NC_000023.11:15 ------------------------------------------------------------
NC_000004.12:c1 ------------------------------------------------------------

NC_000013.11:c3 CAGGTGTGAGCCACTGTGTGCGGCCCTAGTCATGTGGTTTTACAATCGTGCACTCAGTAT
NC_000001.11:33 ------------------------------------------------------------
NC_000023.11:15 ------------------------------------------------------------
NC_000004.12:c1 ------------------------------------------------------------

NC_000013.11:c3 TGAAGGATGCAGGAGACTGGCTCAAAAGGTGCATGGACTGTCCACACAGAGGCCCCTTAG
NC_000001.11:33 ------------------------------------------------------------
NC_000023.11:15 ------------------------------------------------------------
NC_000004.12:c1 ------------------------------------------------------------

NC_000013.11:c3 AGCACATTTACCTCGGTCAGTTTACCGTGGTCCTGGAGCTGTTCCTCAGGCTCAGACATG
NC_000001.11:33 ------------------------------------------------------------
NC_000023.11:15 ------------------------------------------------------------
NC_000004.12:c1 ------------------------------------------------------------

NC_000013.11:c3 TGATTTACTTGTTAATTATCATTATTATTACTGAGTGGAAGCGGCTCCATTTCTCAGCAG
NC_000001.11:33 ------------------------------------------------------------
NC_000023.11:15 ------------------------------------------------------------
NC_000004.12:c1 ------------------------------------------------------------
```

```
NC_000013.11:c3 TGGCCTTACTTTAGCCAAGCTCATCAAAACTGATTATCCACGCATATGTATGCAGCTGAA
NC_000001.11:33 ------------------------------------------------------------
NC_000023.11:15 ------------------------------------------------------------
NC_000004.12:c1 ------------------------------------------------------------

NC_000013.11:c3 GGCCAATGCATTTGGTCTGCAGGGGGATCTGCAACTGGCAGCCTGTCTGTCTGATCAGGG
NC_000001.11:33 ------------------------------------------------------------
NC_000023.11:15 ------------------------------------------------------------
NC_000004.12:c1 ------------------------------------------------------------

NC_000013.11:c3 CCCATGCCATGAAAAACAAGCTGCTCCAGGATTGCTGTGGAATCAAACTACTGAAGGGCA
NC_000001.11:33 ------------------------------------------------------------
NC_000023.11:15 ------------------------------------------------------------
NC_000004.12:c1 ------------------------------------------------------------

NC_000013.11:c3 TTGTTAGGTGATTTCAGTGTTCACGCATCAGAGCCTGAACTGTCACGTTCATTCTTCAGG
NC_000001.11:33 ------------------------------------------------------------
NC_000023.11:15 ------------------------------------------------------------
NC_000004.12:c1 ------------------------------------------------------------

NC_000013.11:c3 GATGTGGGTAGACATCCTCATTGACTTAACAGATGGGTGTTGCCTACTTTTCACTCCTTG
NC_000001.11:33 ------------------------------------------------------------
NC_000023.11:15 ------------------------------------------------------------
NC_000004.12:c1 ------------------------------------------------------------

NC_000013.11:c3 GGTTCCCTTTTCTGACCCTTCTAGTCACACTCCTCCCCTAAGCATGTTCAGTGTTTGCCT
NC_000001.11:33 ------------------------------------------------------------
NC_000023.11:15 ------------------------------------------------------------
NC_000004.12:c1 ------------------------------------------------------------

NC_000013.11:c3 CTGCTCCCTAAATTACAATCACCTTCAAATCCTACTTCCCTGATATTACTCCCAGATTTT
NC_000001.11:33 ------------------------------------------------------------
NC_000023.11:15 ------------------------------------------------------------
NC_000004.12:c1 ------------------------------------------------------------

NC_000013.11:c3 GATTGGAGTTTTGGGTTACCTTGACCTCCTCAGTCACTCATTTACAGAGCAGGCATCCTT
NC_000001.11:33 ------------------------------------------------------------
NC_000023.11:15 ------------------------------------------------------------
NC_000004.12:c1 ------------------------------------------------------------

NC_000013.11:c3 TTGATTTACTCTTGCCAATCAATCTTTTTTTTTTTTAAGAGACAAGCTCTCACTATGTTG
NC_000001.11:33 ------------------------------------------------------------
NC_000023.11:15 ------------------------------------------------------------
NC_000004.12:c1 ------------------------------------------------------------

NC_000013.11:c3 CCTAGTCTGGTCTCGAACTCCTGAGCTCAAGTGATCCTCCTGCCTCAGCCTCCCCAAGTG
NC_000001.11:33 ------------------------------------------------------------
NC_000023.11:15 ------------------------------------------------------------
NC_000004.12:c1 ------------------------------------------------------------

NC_000013.11:c3 CTAGGATTATAGGCATGAGTCACCATGCCTGGCCCCAATTATGTCTATGGTGTGTGGATT
NC_000001.11:33 ------------------------------------------------------------
NC_000023.11:15 ---TGACTATAGG-----------------------------------------------
```

```
NC_000004.12:c1 ------------------------------------------------------------

NC_000013.11:c3 TAATCAGGTGCATCTCAGGTAACAACTTTAGCTCTTAAAGGGTAGGCCACAGTCGCCCCC
NC_000001.11:33 ------------------------------------------------------------
NC_000023.11:15 -----------ATTCAAGATAACAA-----------------------------------
NC_000004.12:c1 ------------------------------------------------------------

NC_000013.11:c3 ACAAAGAAGAATCAGAGAGGTTAGACCAGGAGGGTAGGCTAAGGTTGAAAAGGCCTTGAA
NC_000001.11:33 ------------------------------------------------------------
NC_000023.11:15 ------------------------------------------------------------
NC_000004.12:c1 ------------------------------------------------------------

NC_000013.11:c3 GATGTAGCTAAGGAGATTATGACTTGTCCTCTGAATAGTAGGGAACCTCTAGAGGTGTGT
NC_000001.11:33 ---------------ACTACAGCTTGTCCCCTG---------------------------
NC_000023.11:15 ---------------TTAATACCTTTTCTTCTG---------------------------
NC_000004.12:c1 ------------------------------------------------------------

NC_000013.11:c3 GTTTGGGGCAGTGGGACAGCTTGATGAAATCAATGTTTGGGGCCTGCAGGTAAACTGCAC
NC_000001.11:33 ------------------------------------------------------------
NC_000023.11:15 ------------------------------------------------------------
NC_000004.12:c1 ------------------------------------------------------------

NC_000013.11:c3 TAGAGTAAGGAGAAACCAAAGGCCGAGAGCCCAGGTAAGAAGCCAGGGCAAGGGAGATAG
NC_000001.11:33 ------------------------------------------------------------
NC_000023.11:15 ------------------------------------------------------------
NC_000004.12:c1 ------------------------------------------------------------

NC_000013.11:c3 ATGGGACATGCAAGGCCCAGCCTAAGATGGTGCCAAAATCCCAAAAGGAAAAACTGCGTT
NC_000001.11:33 ------------------------------------------------------------
NC_000023.11:15 ------------------------------------------------------------
NC_000004.12:c1 ------------------------------------------------------------

NC_000013.11:c3 CAATAGACATTTCACAGAGAGAAGCAAGAAGACTTGGTGTATTAGTCTGTTTTCCATTGC
NC_000001.11:33 ------------------------------------------------------------
NC_000023.11:15 --------------------------------CTTG------------------------
NC_000004.12:c1 ------------------------------------------------------------

NC_000013.11:c3 CATAAAGGAATGCCTGAGGCTGGTAATTTATAAAGAAAAGAGGTTTATTTGGCTCACGAT
NC_000001.11:33 -------------------------------------GAGGGGCTTCCCTGGC-------
NC_000023.11:15 ----------------------------------GAGGATTTGGTTTTTTGGT-------
NC_000004.12:c1 ------------------------------------------------------------

NC_000013.11:c3 TCTGCAGGCTATATAAAAAGCATGGCACCAGCATCTGCTCAGTCTCAGGAAGCTTTCACT
NC_000001.11:33 ---------------------------------------CAGTCC---------------
NC_000023.11:15 ---------------------------------------CGTCCT---------------
NC_000004.12:c1 ------------------------------------------------------------

NC_000013.11:c3 CATGGAAGAAGATGAAGGGGGAGCAGGCACGTCACATGGCAAGATAGAGCAAGAGAGAGA
NC_000001.11:33 ------------------------------------------------------------
NC_000023.11:15 ------------------------------------------------------------
NC_000004.12:c1 ------------------------------------------------------------

NC_000013.11:c3 TGAGGAGGTGCCAGGCTCCTTTAAACAACCAGCTCTCACTCATTACCTCAGGGAGGGCAC
```

```
NC_000001.11:33 ------------------------------------------------------------
NC_000023.11:15 ------------------------------------------------------------
NC_000004.12:c1 ------------------------------------------------------------


NC_000013.11:c3 CAAACCATTCATGAGGGATCCACCCCCATGGCCCAAACACCTCCCACCAGGCCCTGCCTC
NC_000001.11:33 ------------------------------------------------------------
NC_000023.11:15 ------------------------------------------------------------
NC_000004.12:c1 ------------------------------------------------------------


NC_000013.11:c3 CAGCGTTGGGGATGACATTTCAACATGAAATTTGGAGAGAAGCAATATCCAAACCGTATC
NC_000001.11:33 ------------------------------------------------------------
NC_000023.11:15 ------------------------------------------------------------
NC_000004.12:c1 ------------------------------------------------------------


NC_000013.11:c3 ATTTGGTGACTGCTGGGCTGTAGTGGATAATGAAAAGGAAAACATTGATGCCCTGTTTTT
NC_000001.11:33 ------------------------------------------------------------
NC_000023.11:15 ------------------------------------------------------------
NC_000004.12:c1 ------------------------------------------------------------


NC_000013.11:c3 CTTTAGTTCTATGATTATTACTGGCAGCATTTCAGGTATCTTTAATTACCATTATACTTG
NC_000001.11:33 ------------------------------------------------------------
NC_000023.11:15 -----------------------------------------------------GTATTTC
NC_000004.12:c1 ------------------------------------------------------------


NC_000013.11:c3 GTTTTAGGTGTGTGTTTATAAGCACTGTATATGTGTAAAGCCGTATTACCTCAGTGTGAT
NC_000001.11:33 ------------------------------------------------------------
NC_000023.11:15 ATTTC-------------------------------------------------------
NC_000004.12:c1 --TTT-------------------------------------------------------


NC_000013.11:c3 GAGTAATAGGGTCTGGCTCCTCCACTCTGGTTCACTTAGTACATTTAAAAGAATGAGCAA
NC_000001.11:33 -------------------------------------------------AGAATGCTCAA
NC_000023.11:15 -------------------------------------------------AAAATGAAATA
NC_000004.12:c1 -------------------------------------------------AAAATAACCCA
                                                                 *.***.    *

NC_000013.11:c3 CTTGTAAAAAACTATCTATTGGCTAGACTCTCTTCTCAAACATCTTAGGAAGAGATGTTC
NC_000001.11:33 TTTATGAGAGATCAT---------------------------------------------
NC_000023.11:15 TTTTTTAAAAGATACTTA------------------------------------------
NC_000004.12:c1 ------------------------------------------------------------


NC_000013.11:c3 CTGATATACACAATACATTTTGAAGAATAAGGAATAGAATTTGGAGAGAAACTGAGGATA
NC_000001.11:33 ------------------------------------------------------------
NC_000023.11:15 ------------------------------------------------------------
NC_000004.12:c1 ------------------------------------------------------------


NC_000013.11:c3 CTGGACTGATTGAGGAGGGCTGGAGGAGAATTGATCTCTCAATTAGTAAGCGTTTATTAA
NC_000001.11:33 ------------------------------------------------------------
NC_000023.11:15 ------------------------------------------------------------
NC_000004.12:c1 ------------------------------------------------------------


NC_000013.11:c3 GCACTTACTATATGCCCAAGATTGTGCGAGATGCTGTGGAAGCTACAAAAGCCATGTAAC
NC_000001.11:33 ------------------------------------------------------------
NC_000023.11:15 ------------------------------------------------------------
NC_000004.12:c1 ------------------------------------------------------------
```

```
NC_000013.11:c3 AGCTTCTTTTCATGAGGAACTTCATAGCCTCCTCGCGAGTTAGATCTTACTCTGAGAAAT
NC_000001.11:33 ------------------------------------------------------------
NC_000023.11:15 ------------------------------------------------------------
NC_000004.12:c1 ------------------------------------------------------------

NC_000013.11:c3 AACCAGAAGCAAGCAACATAAAAGGAAATGGAACCGTGCCGCTAAGCCAGAGCATAGAGA
NC_000001.11:33 ------------------------------------------------------------
NC_000023.11:15 ------------------------------------------------------------
NC_000004.12:c1 ------------------------------------------------------------

NC_000013.11:c3 CCAGTGGTATTTTCATACAAACTGAGTGATGAGCCTCTGGAAGCCATGGCAGAGACAACA
NC_000001.11:33 ------------------------------------------------------------
NC_000023.11:15 --------------------------------------------------AAAGATGGCA
NC_000004.12:c1 ------------------------------------------------------------

NC_000013.11:c3 ACAAGGTCAGAAGGATGGAAAAAAAAAATCATAGCTTGAAAGGAATAGGCAAACTGGGGC
NC_000001.11:33 ------------------------------------------------------------
NC_000023.11:15 ATA---------------------------------------------------------
NC_000004.12:c1 ------------------------------------------------------------

NC_000013.11:c3 TGGTAAGTTTAGTAACGGATTCCAGAGGGATTTATCCTTTCTTTTTTTTCCCAGAGGTTT
NC_000001.11:33 ------------------------------------------------------------
NC_000023.11:15 ------------------------------------------------------------
NC_000004.12:c1 ------------------------------------------------------------

NC_000013.11:c3 ATTCTTTGCAAAACACTTTTATGTGTATTAATCTCATTTTAGCTTTGACACAATCCAGCA
NC_000001.11:33 ------------------------------------------------------------
NC_000023.11:15 --CCTCTGCAAACCATTCT-----------------------------------------
NC_000004.12:c1 ------------------------------------------------------------

NC_000013.11:c3 AGGGAGGCAAGGTGGGGATTAAAAATGGGGAAAGGATTTGAGAGAGATTTATGATGTAGA
NC_000001.11:33 ------------------------------------------------------------
NC_000023.11:15 ---------------------------------GTACTTGGGC----TTTACGATGCAGT
NC_000004.12:c1 ------------------------------------------------------------

NC_000013.11:c3 AACAGCAGGATTTTATGACCAATTAATCTTCTGGTTAAGGGTGAGGGAGAGGCAAAGATG
NC_000001.11:33 ------------------------------------------------------------
NC_000023.11:15 GCCAGCGTTGT-----------------CTGTGGTTAAGGCTGA----------------
NC_000004.12:c1 ------------------------------------------------------------

NC_000013.11:c3 CCTACCCCCACTCACTCATTCACTCATAAAATCACTCATACAACAAATACTTGTTGAGGA
NC_000001.11:33 ------------------------------------------------------------
NC_000023.11:15 ------------------------------------------------------------
NC_000004.12:c1 ------------------------------------------------------------

NC_000013.11:c3 TATATTATGTGTCCTGTCTATCCACTGGTACTGGGCTTGCAGATACCTGGCTGAGGCTAT
NC_000001.11:33 ------------------------------------------------------------
NC_000023.11:15 ------------------------------------------------------------
NC_000004.12:c1 ------------------------------------------------------------

NC_000013.11:c3 GAGGTCAGTTACGGTGCCCGGCCTGAGCAGGAGGTGGTGGAGGAAGAGTACGGGATGCAG
NC_000001.11:33 ------------------------------------------------------------
NC_000023.11:15 ------------------------------------------------------------
```

```
NC_000004.12:c1 ------------------------------------------------------------


NC_000013.11:c3 GTGGTTGTTCAGGTTCCAGGGGATTTGGGTTTCCACCCTGTGGATTTGGGAGGGCTATGG
NC_000001.11:33 ------------------------------------------------------------
NC_000023.11:15 ------------------------------------------------------------
NC_000004.12:c1 ------------------------------------------------------------


NC_000013.11:c3 GACACACAGGTGAAATTGACAAGTAGGCAATTTTACAGAAAGGTTAGCTTTCTCTGGACA
NC_000001.11:33 ------------------------------------------------------------
NC_000023.11:15 ------------------------------------------------------------
NC_000004.12:c1 ------------------------------------------------------------


NC_000013.11:c3 GAGGTCTGGGATATGAAATAGACTATTAATTAGCCTATAGGTAAGAGTTAAAACTGAGAG
NC_000001.11:33 ------------------------------------------------------------
NC_000023.11:15 ------------------------------------------------------------
NC_000004.12:c1 ------------------------------------------------------------


NC_000013.11:c3 TGGATTAGGTAACCTCCAGAGATCTTGGTTAAGATACTTAACAATTCAGAGCTTCAGTTT
NC_000001.11:33 ------------------------------------------------------------
NC_000023.11:15 ------------------------------------------------------------
NC_000004.12:c1 ------------------------------------------------------------


NC_000013.11:c3 CTTGCTACGGGAAATGAAGAAGATAGTATCTACACCGCGGGTTTATTTTAGATCCAGTGA
NC_000001.11:33 ------------------------------------------------------------
NC_000023.11:15 ------------------------------------------------------------
NC_000004.12:c1 ------------------------------------------------------------


NC_000013.11:c3 ATATGCATGGACCTTGAATACAGCAGTGCATTATATATGTTGAAAATATTATTGCCAATA
NC_000001.11:33 ------------------------------------------------------------
NC_000023.11:15 ------------------------------------------------------------
NC_000004.12:c1 ------------------------------------------------------------


NC_000013.11:c3 TTTTACCACTATAGTATCTGCATCTTGTCTCGCCCATCAGTTTAGTAAATTCCTCAAAAA
NC_000001.11:33 ------------------------------------------------------------
NC_000023.11:15 ------------AGTGTTTGTG--------------------------------------
NC_000004.12:c1 ------------------------------------------------------------


NC_000013.11:c3 CCAAAGTTTTGCACTCATATTCCTGCATTATCTCATTATCTTCATATCCCCAGGCAAAGG
NC_000001.11:33 ------------------------------------------------------------
NC_000023.11:15 ------------------------------------------------------------
NC_000004.12:c1 ------------------------------------------------------------


NC_000013.11:c3 TTAAGAGTTAAACATGAATAGGTCTTGACATCTGGGCTTTGGAATAGAAATTAAGAATAT
NC_000001.11:33 ------------------------------------------------------------
NC_000023.11:15 ------------------------------------------------------------
NC_000004.12:c1 ------------------------------------------------------------


NC_000013.11:c3 TGGGGCACGGCCAGGCGTGGTGGCTCACATCTGTAATCCCAACACTTTGGGAGGCTGAGG
NC_000001.11:33 --------------------TGGCTCACA-------------------------------
NC_000023.11:15 --------GGCTGTGCTTGCACATTCACA-------------------------------
NC_000004.12:c1 ------------------------------------------------------------


NC_000013.11:c3 CAGGTGAATAATTTGAGGTCAGGAGTTTAAGACCAGCTGGCCAACATTGCGAAACCCCGT
```

```
NC_000001.11:33 ------------------------------------------------------------
NC_000023.11:15 ------------------------------------------------------------
NC_000004.12:c1 ------------------------------------------------------------

NC_000013.11:c3 CTCAACTAAAAATACAAAAATTAGCTGAGTGTGGTGGTACACACCTGTAGTCCCAGCTAC
NC_000001.11:33 ------------------------------------------------------------
NC_000023.11:15 ------------------------------GTGGTTGT------CTGTTGGCCTTTCCAT
NC_000004.12:c1 ------------------------------------------------------------

NC_000013.11:c3 TCAGGAGGCTGAGACACGATAATCATATGAACCCAGGAGGCAGAGGTTGCAGTGAGCCAA
NC_000001.11:33 ------------------------------------------------------------
NC_000023.11:15 T-----------------------------------------------------------
NC_000004.12:c1 ------------------------------------------------------------

NC_000013.11:c3 GATCACACCACCACACTCCAGCCTGGGTGACAGAGTGAGATTCCATCTCAAAAAAAAAAG
NC_000001.11:33 ------------------------------------------------------------
NC_000023.11:15 ------------------------------------------------------------
NC_000004.12:c1 ------------------------------------------------------------

NC_000013.11:c3 AATATTGGTGCTGGGCGCAGTGACTCATGCCTATAATTCCAGCATTTTGAGAGGCCGAGG
NC_000001.11:33 ---------------------------------------------------------GGG
NC_000023.11:15 ---------------------------------------------------------GTG
NC_000004.12:c1 ------------------------------------------------------------

NC_000013.11:c3 AGGAAGGATGCCTAAAACCCAAGAGCTAGAGACCAGCCTGGGCAAAATGGTGAGACCCCC
NC_000001.11:33 AGGAAGG-----------------------------------------------------
NC_000023.11:15 AAGGAGG--------------------------CACTCTAGTTTTGGTTGTCAGGGTCCT
NC_000004.12:c1 ------------------------------------------------------------

NC_000013.11:c3 ATCTCTACAAAAAAGCATGTTTTTAATTAGCAGGGCATGGTGGCGCACCTGTGGTTCCAG
NC_000001.11:33 ------------------------------------------------------------
NC_000023.11:15 ----------TGAAGTATGTTCTTTAT-------------------------------GC
NC_000004.12:c1 ------------------------------------------------------------

NC_000013.11:c3 CTGCTCCAGCAGCTGAGGCAGAAGGATGGCTTGAACCCAGGAAGTCGAGGCTACAGTGTG
NC_000001.11:33 -----------------------GGATGGCTTAG--------------------------
NC_000023.11:15 CTTTCCTAGTAATTGAAAGACTGGGACAGCACCA-------------------------T
NC_000004.12:c1 ------------------------------------------------------------

NC_000013.11:c3 CTGTGATAGCACCACTGCACTCCAGCCTGGATGACATAGCAAGACCCTGTCTCAAAACAA
NC_000001.11:33 ----------------GTAGGCCAGCC----------------ACCTTGCAGCAGAGCTG
NC_000023.11:15 ATATATATGCGCGTATATATACGCATATGTATTATATATGCATACACACACACAGAGCAC
NC_000004.12:c1 ------------------------------------------------------------

NC_000013.11:c3 AACAAAAATATTGGATTAATT-CATGAATATATTTTAGATATGAAAGGTGTATCTGATCA
NC_000001.11:33 GGCAAGAATTCAGGATTGG-----------------------------------------
NC_000023.11:15 CATATATATATGCGCGTATATATACGCATATATTTTATATATGCA---------------
NC_000004.12:c1 ------------------------------------------------------------

NC_000013.11:c3 GGAACTGGGGCAGAAAGATAATTATGCAAGGCAGAACGGTGGTGCTGTTGTTGAGTTTGA
NC_000001.11:33 ------------------------------------------------------------
NC_000023.11:15 ------------------------------------------------------------
NC_000004.12:c1 ------------------------------------------------------------
```

```
NC_000013.11:c3 TTTCCAAAAGCACAGCTAATGAATGATTAATGTGAAAAAGTGTTTGCACATAGTCTGACC
NC_000001.11:33 ------------------------------------------------------------
NC_000023.11:15 --------------------------------------------TACACACA--------
NC_000004.12:c1 ------------------------------------------------------------

NC_000013.11:c3 CTTGAAAAATGAAGGGGAGGGGAAATTTAGAGAAATTTGAGAGAAAAATTCAGGTTATGA
NC_000001.11:33 ------------------------------------------------------------
NC_000023.11:15 ------------------------------------------------------------
NC_000004.12:c1 ------------------------------------------------------------

NC_000013.11:c3 TTAGACTCCCTGATTGTCAAAATGAAAGGCGGTGAGAAAACCAGGTACCTTTAGGAAAAT
NC_000001.11:33 ------------------------------------------------------------
NC_000023.11:15 ------------------------------------------------------------
NC_000004.12:c1 ------------------------------------------------------------

NC_000013.11:c3 ATTCAAAGCAAATTGGTGGAGACTAGGTTTAGGAGCACTGTTTTAACTGAGTACAAGGAT
NC_000001.11:33 ------------------------------------------------------------
NC_000023.11:15 ------------------------------------------------------------
NC_000004.12:c1 ------------------------------------------------------------

NC_000013.11:c3 CAAACAGGTGACCAAGGAATGTTTTCAAAGGCCTACTTTTTTGTCATTTTCTTTTCTTTT
NC_000001.11:33 ------------------------------------------------------------
NC_000023.11:15 -------------------------CACACGCATACATAT--------------------
NC_000004.12:c1 ------------------------------------------------------------

NC_000013.11:c3 CTTTTCTTTTTTTCTTGAGAGGGAGTCTTGCTCTGTCATCAGACTGGAGTGCGGTGGTGT
NC_000001.11:33 ------------------------------------------------------------
NC_000023.11:15 --------------TTGAGATGGAGTTTCGCTCT-TGACCAGGCTGGAGTGCAA-GGTGT
NC_000004.12:c1 ------------------------------------------------------------

NC_000013.11:c3 GATCTTGGCTCACTGCAACCTCTGCCTCCCAGGTTCAAGCAATTCTCCTGCCTCAGCCTC
NC_000001.11:33 ------------------------------------------------------------
NC_000023.11:15 GATCTTGGCTCACTGCAACCTCCACCTCCTGGGTTCAAGTGATTCTCCTGCCTCAGCCTC
NC_000004.12:c1 ------------------------------------------------------------

NC_000013.11:c3 CCGAGTAGCTAGGACTACAGGCATACACCCCCACGCCCAGCTAA---TTTTTGTATTTTT
NC_000001.11:33 ------------------------------------------------------------
NC_000023.11:15 CTGAGTAGCTGGGATTACAGACATGCACCACCATGCCCGGCTAATTTTTTTTATATTTTT
NC_000004.12:c1 ------------------------------------------------------------

NC_000013.11:c3 ATTAGAGATGGGGTTTCACCATGTTGGCCAGGCTGGTCTTGAACTCCTGGCCTCAGCGGA
NC_000001.11:33 ------------------------------------------------------------
NC_000023.11:15 AGTAGAGACGGGGTTTCTCCATGTTGGTCAGGCTGGTCTGAAACTCCCAACCTCAGGTGA
NC_000004.12:c1 ------------------------------------------------------------

NC_000013.11:c3 TCTGCCTGCCTCAGCCTCCCAAAGCGCTGAGATTACAGGCATGAGCCACTCCGCCTGGCA
NC_000001.11:33 ------------------------------------------------------------
NC_000023.11:15 TCCGCCTGCCTCGGCCTCCCAAAGTGCTGGAATTACAGGCGTG-----------------
NC_000004.12:c1 ------------------------------------------------------------

NC_000013.11:c3 TGTCATTTTCAATTCCATAAGACATGGTATAAACACGACTTTCTTAGATTAATCTATTTT
NC_000001.11:33 ------------------------------------------------------------
NC_000023.11:15 ------------------------------------------------------------
```

```
NC_000004.12:c1 ------------------------------------------------------------


NC_000013.11:c3 TTGAATGATGTTACTCTAACTCCATGAATTAGAACCTGCCTGGGGAAATAAGGAACATAT
NC_000001.11:33 ------------------------------------------------------------
NC_000023.11:15 ------------------------------------------------------------
NC_000004.12:c1 ------------------------------------------------------------


NC_000013.11:c3 TTATACTCAGTTTTTAATTTTTCATCCAGTATGATATGGGAGTAACAGTTCAAGGCCATG
NC_000001.11:33 ------------------------------------------------------------
NC_000023.11:15 ------------------------------------------------------------
NC_000004.12:c1 ------------------------------------------------------------


NC_000013.11:c3 GCCAGCCACTCCATTTAATTTTCTGGCTCTCATTAATTATTTTTTCTTTTTTAGAGAGAG
NC_000001.11:33 -----------------------AGACTCTTATTAGTTTCCATCTTTCTTTTA-------
NC_000023.11:15 -----------------------AGCCGCGCACCACATATTTTTCTAACCTTA-------
NC_000004.12:c1 -----------------------AATTGCTCCTTGGATTTTTCCTTCAGTTTA-------
                         . . * . ...  * .. ....   .***

NC_000013.11:c3 AGTCTCACTCTGTGGCCCAGGCTGGAATGTAGTAGTGCCGTCATAGCTCGCTGCAGCCTT
NC_000001.11:33 ------------------------------------------------------------
NC_000023.11:15 ------------------------------------------------------------
NC_000004.12:c1 ------------------------------------------------------------


NC_000013.11:c3 GAATCCCGGGGCTCAAGCGATCCTCCCGATTCAGCCTTCCTTAATAGCTGGGACTACAGG
NC_000001.11:33 ------------------------------------------------------------
NC_000023.11:15 ------------------------------------------------------------
NC_000004.12:c1 ------------------------------------------------------------


NC_000013.11:c3 TGCACACCACCACACCTGGCTAATTTTTTAAAATTTTATGTAGAGATGGAGTCTCACTAT
NC_000001.11:33 ------------------------------------------------------------
NC_000023.11:15 ---------------------------------------------------------TAT
NC_000004.12:c1 ------------------------------------------------------------


NC_000013.11:c3 GTTTTCTAGGCTGGTCATGAACTCCTAGGCTCAAATGTTCTTCCCACTTCAGCCTCCCAA
NC_000001.11:33 ------------------------------------------------------------
NC_000023.11:15 ATTTT-------------------------------------------------------
NC_000004.12:c1 ------------------------------------------------------------


NC_000013.11:c3 GCAGCTGGGATTCCAACTCTGGCTCTTGTAATTGATGGGTGCGTAGGGATGTCTTCTGTG
NC_000001.11:33 ------------------------------------------------------------
NC_000023.11:15 ------------------------------------------------------------
NC_000004.12:c1 ------------------------------------------------------------


NC_000013.11:c3 TAGGTAACCAAATCTCAGAAGTTAACTAGAGGAAGAAGAATGGTATCCCGGAACTGGAGA
NC_000001.11:33 --------------------------CAGAAGATGAACACTGAAGTCTTGGAAC------
NC_000023.11:15 ---------AGAACCTACAACTT---TGTAGCATTGTATATGGTATCATAGATT------
NC_000004.12:c1 ------------------------------------------------------------


NC_000013.11:c3 TTTGCCTACTCTGCTATCAGGAAAACAAGGTGGGGTAGTTTCTTGAAATAGTAATTCTGC
NC_000001.11:33 ------------------------------------------------------------
NC_000023.11:15 ------------------------------------------------------------
NC_000004.12:c1 ------------------------------------------------------------


NC_000013.11:c3 TGTGCTAGTCTTCCTTAGACCCCAGGAATGACTGTGAAGAGTCAGTCTGTCTTTCTGCGG
```

```
NC_000001.11:33 ------------------------------------------------------------
NC_000023.11:15 ------------------------------------------------------------
NC_000004.12:c1 ------------------------------------------------------------

NC_000013.11:c3 TGACAGAAGCTAAAGAAACCAGATACTTTGTTCCCTCATTCAGTCATTCATCAAACATTT
NC_000001.11:33 ------------------------------------------------------------
NC_000023.11:15 ------------------------------------------------------------
NC_000004.12:c1 ------------------------------------------------------------

NC_000013.11:c3 GTGCCAGAGAGTGTGGTGATTGCTGGAGAGGGGTGGATGGAGTAATAGTTCTTGCTCCTA
NC_000001.11:33 ------------------------------------------------------------
NC_000023.11:15 ------------------------------------------------------------
NC_000004.12:c1 ------------------------------------------------------------

NC_000013.11:c3 GGAATCTTCCAGTCCAGTGGAGAAGATAATACTGGGTTCCAGGTGCCACCTCTGAATACG
NC_000001.11:33 ------------------------------------------------------------
NC_000023.11:15 ------------------------------------------------------------
NC_000004.12:c1 ------------------------------------------------------------

NC_000013.11:c3 TGTGTGAGGTGAAATGCCCACCCAGGGTTAGGGCCAGGTTGCCCTTCACACAGGTTGTGA
NC_000001.11:33 ------------------------------------------------------------
NC_000023.11:15 ------------------------------------------------------------
NC_000004.12:c1 ------------------------------------------------------------

NC_000013.11:c3 CGCTGAAGCTGAGTCTTATGGGATGAGCAGGAAGTGGCCAAATTAGATTGGGAGTTGGAA
NC_000001.11:33 ------------------------------------------------------------
NC_000023.11:15 ------------------------------------------------------------
NC_000004.12:c1 ------------------------------------------------------------

NC_000013.11:c3 TTCCAGGCAACAGGGAGAGAGCTTAGTGGAGGGTTAAGGCATTTGAGCTATAGTGGAAGA
NC_000001.11:33 ------------------------------------------------------------
NC_000023.11:15 -----------------------------------------------------------A
NC_000004.12:c1 ------------------------------------------------------------

NC_000013.11:c3 CTGCACTTTTATGAGTGAGTGACAGAAGATGGAGCTGGAGTGGTAGTCAGGCTCAGGAGG
NC_000001.11:33 ------------------------------------------------------------
NC_000023.11:15 CTTCACTTT----------------AGGGAGAAGTCATAGTGGTA---------------
NC_000004.12:c1 ------------------------------------------------------------

NC_000013.11:c3 GCCTTGAATGCCATTCTGATGAATTTGGAATTTTGCCCTGAATATGAACAACAAGGAGGC
NC_000001.11:33 ------------------------------------------------------------
NC_000023.11:15 ------------------------------------------------------------
NC_000004.12:c1 ------------------------------------------------------------

NC_000013.11:c3 AAGCACCCTGATCGCTTTGCTTTGGAGGGATCGCCTCCCTATAGACTGAGTCGCCTTCAG
NC_000001.11:33 ------------------------------------------------------------
NC_000023.11:15 ------------------------------------------------------------
NC_000004.12:c1 ------------------------------------------------------------

NC_000013.11:c3 AGTGAGGATGGTCTTATTCATCTTTGTAGCCCCATTATTTTCTGACACACATTTCCTGAG
NC_000001.11:33 ------------------------------------------------------------
NC_000023.11:15 ------------------------------------------------------------
NC_000004.12:c1 ------------------------------------------------------------
```

```
NC_000013.11:c3 CACCTTCGGAGTGAGCACCATGAGAACTAGGGGTGTGGGAGAAATAAGACAGCCTCTGCC
NC_000001.11:33 ------------------------------------------------------------
NC_000023.11:15 ------------------------------------------------------------
NC_000004.12:c1 ------------------------------------------------------------

NC_000013.11:c3 ACTGGGAGAGGGTGATACCCACACAGCTGTGCAACCTGGGGACAGAAGTGCCAGGTAACC
NC_000001.11:33 ------------------------------------------------------------
NC_000023.11:15 ------------------------------------------------------------
NC_000004.12:c1 ------------------------------------------------------------

NC_000013.11:c3 CCAGAGCCTGCAGGAGCGCACCCCACTCAGCAGGAAGATGTGTGTCACTGGCACTTTCCT
NC_000001.11:33 ------------------------------------------------------------
NC_000023.11:15 ------------------------------------------------------------
NC_000004.12:c1 ------------------------------------------------------------

NC_000013.11:c3 AAAGCAGGTGAGGCTTTGAGAATGCCAAACCAGCCAGGCCAAGAGAGCTGGAGAGAAGGG
NC_000001.11:33 ------------------------------------------------------------
NC_000023.11:15 ------------------------------------------------------------
NC_000004.12:c1 ------------------------------------------------------------

NC_000013.11:c3 AAGGCTGAGAGGGGCAGCTCGAGTGTGGATGGATCAATGGCACCTAGCTAGGTGTGCTGC
NC_000001.11:33 ------------------------------------------------------------
NC_000023.11:15 ------------------------------------------------------------
NC_000004.12:c1 ------------------------------------------------------------

NC_000013.11:c3 AGAGAAAGTGCCCGCGGAGAGGCAGAATGACTGACAGAAGGTGGAGGTGGACACGTTGGC
NC_000001.11:33 ------------------------------------------------------------
NC_000023.11:15 ------------------------------------------------------------
NC_000004.12:c1 ------------------------------------------------------------

NC_000013.11:c3 CAGAAACAACAAGGACCGGTCTTCGTGTGAGATGGAAGGAGAAGTGTGAGGGCAGATGGG
NC_000001.11:33 ------------------------------------------------------------
NC_000023.11:15 ---------------------------------GGAGGGAGA------------------
NC_000004.12:c1 ------------------------------------------------------------

NC_000013.11:c3 ACTACAGGCTGGGGTACAGAGGGGTCAGTGATAGAGAAGGGTTCAATAAATGTTGGCTCA
NC_000001.11:33 ------------------------------------------------------------
NC_000023.11:15 ------------------------------------------------------------
NC_000004.12:c1 ------------------------------------------------------------

NC_000013.11:c3 GTCATCGGTTAAGAGGGTAGCCTCCGTGCTACACTTGGGTGGGTCTGGGAACGCAGCTGA
NC_000001.11:33 ------------------------------------------------------------
NC_000023.11:15 ------------------------------------------------------------
NC_000004.12:c1 ------------------------------------------------------------

NC_000013.11:c3 CCTTTCCTCAGCCAGATTCCTGTTTCCTCTGGCTGTAGCCCTCTCCACAACTGAGGAGAA
NC_000001.11:33 ------------------------------------------------------------
NC_000023.11:15 ------------------------------------------------------------
NC_000004.12:c1 ------------------------------------------------------------

NC_000013.11:c3 TATGCCCTTCAGTCCAGAAGCTAGAAAGAATGTCAGCAGATCTCAAATGCTTCCACCCAA
NC_000001.11:33 ------------------------------------------------------------
NC_000023.11:15 ------------------------------------------------------------
```

```
NC_000004.12:c1 ------------------------------------------------------------


NC_000013.11:c3 GACGCCACCCCTGCCCACTCAGCTCATTCCTCCCTGCAAGGCCCCACTAATACCTGTTCA
NC_000001.11:33 ------------------------------------------------------------
NC_000023.11:15 ------------------------------------------------------------
NC_000004.12:c1 ------------------------------------------------------------


NC_000013.11:c3 GGTCCCCTGATGCTGCCACCTGGGAAGGGGCTGGAAAGGGCTTGAGGCCTGGCTGCACCT
NC_000001.11:33 ------------------CCTGGGAATGAACCTGGAAAAATTCAAAATATGG--------
NC_000023.11:15 -------------TGCTTCTCAGGAATGTG-------GGATTTCAACTTTAACTCAAATT
NC_000004.12:c1 -----------------------------------------TTAAACTCTGT--------
                                                         *. *. . *.

NC_000013.11:c3 CCCCCGATCCGATCCTGATTCAAAAGGTCTAGCTCTCTGCCTTGGTCAGCCAGTTTAGTC
NC_000001.11:33 ------------------------------------------------------------
NC_000023.11:15 -----------------------------------------------------TTTTGTG
NC_000004.12:c1 ------------------------------------------------------------


NC_000013.11:c3 TTTCTGTGGGGACTGTTTTCTTCTCTTCTCAAATCTGTCTCCAGGCCAGGTGCAGTGGCT
NC_000001.11:33 ------------------------------------------------------------
NC_000023.11:15 GTTTCTCATGTAATGTATGTAATTCTTCCAAGGTCTG-----------------------
NC_000004.12:c1 -------------------TGCTTCCTTTCAGATCTG-----------------------


NC_000013.11:c3 CACACCTGTAATCTCAGCACTTTGGGAGGCCGAGAGGCAGGCTGATCACCAGACGCCAGG
NC_000001.11:33 ------------------------------------------------------------
NC_000023.11:15 ------------------------------------------------------------
NC_000004.12:c1 ------------------------------------------------------------


NC_000013.11:c3 GGTTCAAGACCAGCCTGGCGAACATGACAAAACCCTGTCTCTACTAAAAATACAAAAAAA
NC_000001.11:33 ------------------------------------------------------------
NC_000023.11:15 ------------------------------------------------------------
NC_000004.12:c1 ------------------------------------------------------------


NC_000013.11:c3 CAAAAAAACTGTCTACTTGATGTCCTCAGACTGCCCCCTGAGAGGATGGGAGTGAGTGAA
NC_000001.11:33 ------------------------------------------------------------
NC_000023.11:15 ------------------------------------------------------------
NC_000004.12:c1 ------------------------------------------------------------


NC_000013.11:c3 GACAAAGGGGGGATCCAAAAAGCATGCTTGGCTCTAAGGTGATTGTGTGCACTGAGGTAA
NC_000001.11:33 ------------------------------------------------------------
NC_000023.11:15 ------------------------------------------------------------
NC_000004.12:c1 ------------------------------------------------------------


NC_000013.11:c3 GTGCAGATGAAATCATTGCTTCACACTAATCCACTAGATAGGAGTAAGATGGAAACGGGA
NC_000001.11:33 ------------------------------------------------------------
NC_000023.11:15 ------------------------------------------------------------
NC_000004.12:c1 ------------------------------------------------------------


NC_000013.11:c3 TGGGCCCTGTGTTGAGAACAGTTGAGGTTGAATAACGGGTACAGGGGGTTTCGTTATACT
NC_000001.11:33 ------------------------------------------------------------
NC_000023.11:15 ------------------------------------------------------------
NC_000004.12:c1 ------------------------------------------------------------


NC_000013.11:c3 CTACTTTTGCAATTTTCCATAATAAGAAGTTGAAAAATAGGTCGGGCATGGTGGCTCACA
```

```
NC_000001.11:33 ------------------------------------------------------------
NC_000023.11:15 ------------------------------------------------------------
NC_000004.12:c1 ------------------------------------------------------------

NC_000013.11:c3 CCTGTAATCCCAGAACTTTGGGAGGCCAAGGTGGGTGGATCACCTGAGGCCAGGAGTTCG
NC_000001.11:33 ------------------------------------------------------------
NC_000023.11:15 ------------------------------------------------------------
NC_000004.12:c1 ------------------------------------------------------------

NC_000013.11:c3 AGACCAGCCTAACCAATATGGTGAAACCCATCTCTATTAAAAATATAAAAATGGTTGGGC
NC_000001.11:33 ------------------------------------------------------------
NC_000023.11:15 ------------------------------------------------------------
NC_000004.12:c1 ------------------------------------------------------------

NC_000013.11:c3 GCGGTGGCTTACACCTGTAATCCCAGCACTTTGGGAGGTCAAGGTGGGCTGATCACGAGG
NC_000001.11:33 ------------------------------------------------------------
NC_000023.11:15 ------------------------------------------------------------
NC_000004.12:c1 ------------------------------------------------------------

NC_000013.11:c3 TCAGGAGATGGAGACCATCCTGGCTAACACGGTGAAACCCTGTCTCTACTAAAAATACAA
NC_000001.11:33 ------------------------------------------------------------
NC_000023.11:15 ------------------------------------------------------------
NC_000004.12:c1 ------------------------------------------------------------

NC_000013.11:c3 AAAATTAGCCAGGCCTGGTGGCACGCACCTGTAATCCCAGCTACTAGGGAGGCTGAGGCA
NC_000001.11:33 ------------------------------------------------------------
NC_000023.11:15 ------------------------------------------------------------
NC_000004.12:c1 ------------------------------------------------------------

NC_000013.11:c3 GGAGAATCGTTTGAACCCAGGAGGCGGAGGTTGCAGTGAGCTGAGATGGCGCCACTGTAC
NC_000001.11:33 ------------------------------------------------------------
NC_000023.11:15 ------------------------------------------------------------
NC_000004.12:c1 ------------------------------------------------------------

NC_000013.11:c3 TCCAGCCTGGGTGACAAGGGCAAGACTCCGTCTCAAACAATAACAACAACAACAACAACG
NC_000001.11:33 ------------------------------------------------------------
NC_000023.11:15 ------------------------------------------------------------
NC_000004.12:c1 ------------------------------------------------------------

NC_000013.11:c3 ACAACAACAAATATATAAATATATATATATAAATTAGCTGGGCATGGTGGCGGCGCCTGT
NC_000001.11:33 ------------------------------------------------------------
NC_000023.11:15 ------------------------------------------------------------
NC_000004.12:c1 ------------------------------------------------------------

NC_000013.11:c3 AGTCCCAGCTACTTGGGAAGCTGAGATAAGAGAATTGCTTAAACCCGGGAGGCAGAGGTT
NC_000001.11:33 ------------------------------------------------------------
NC_000023.11:15 ------------------------------------------------------------
NC_000004.12:c1 ------------------------------------------------------------

NC_000013.11:c3 GCAGTGAGCCGAGATCACACCACTGCACTCCAGCCTGGGTGACAGAGTGAGACTCTCTCT
NC_000001.11:33 ------------------------------------------------------------
NC_000023.11:15 ------------------------------------------------------------
NC_000004.12:c1 ------------------------------------------------------------
```

```
NC_000013.11:c3 CAAAAAGAAAAAAAAATTGAAAAATAAATCGTTGGTATTTTGAAAATGCTGTGAATTGAC
NC_000001.11:33 ------------------------------------------------------------
NC_000023.11:15 ------------------------------------------------------------
NC_000004.12:c1 ------------------------------------------------------------


NC_000013.11:c3 ACTAATACTTCATTTTTGCCAGTCATGTGATAAACGTAATTATTTACCAGTGTAGGGGGA
NC_000001.11:33 ------------------------------------------------------------
NC_000023.11:15 ------------------------------------------------------------
NC_000004.12:c1 ------------------------------------------------------------


NC_000013.11:c3 AAAAAGCAAAGAACTGTTTTGTAGGAACACTGCTGGTTTGTAGCTCTCCTTCGTTCTCTC
NC_000001.11:33 ------------------------------------------------------------
NC_000023.11:15 ------------------------------------------------------------
NC_000004.12:c1 ------------------------------------------------------------


NC_000013.11:c3 ACCTCCAACTCCAGTCTTTGGCCAGTATTATCTGGTATTACCCCTCCTTGGTTTTAATCA
NC_000001.11:33 ---------TCTAGTCTTAGGC--------------------------------------
NC_000023.11:15 ------------------------------------------------------------
NC_000004.12:c1 ------------------------------------------------------------


NC_000013.11:c3 GAGTTACCCTTCAACTTCATTCCTCCTGTCTGAGCATGCTTGCCTTTATCTGTTTTTCAT
NC_000001.11:33 ------------------------------------------------------------
NC_000023.11:15 ------------------------------------------------------------
NC_000004.12:c1 ------------------------------------------------------------


NC_000013.11:c3 CTGTAAGGATAATCCTAGTGTAAGGAACTTCGATTTCAACCTAACCTAAACCTTCCCCTG
NC_000001.11:33 ------------------------------------------------------------
NC_000023.11:15 ------------------------------------------------------------
NC_000004.12:c1 ------------------------------------------------------------


NC_000013.11:c3 ATCTAAGCTTTTCTTTCCACTGCAGCTATGACTTTGATGTGATGGGGAGTTGCTGGTTGT
NC_000001.11:33 ------------------------------------------------------------
NC_000023.11:15 ------------------------------------------------------------
NC_000004.12:c1 ------------------------------------------------------------


NC_000013.11:c3 CATACTTGTTTTCAAATTACCCCTCTTGGATAAATAATTTTTTTTGTCTAGCTTTATCAT
NC_000001.11:33 -----------------------------------AAGTTTCCTTGTCCGGCT-------
NC_000023.11:15 -----------------------------------GATTCTTCCTGTTCTGTT-------
NC_000004.12:c1 -----------------------------------CCTTCTTCCTGTTTTGCT-------
                                                         *.*...***.. *.*

NC_000013.11:c3 AAAGGTCTCTGATGTTTTGAAAAGAAGTATCTAGATAACCAACCAGAGAACTGTTGGGGG
NC_000001.11:33 ------------------------------------------------------------
NC_000023.11:15 ------------------------------------------------------------
NC_000004.12:c1 ------------------------------------------------------------


NC_000013.11:c3 GAAAAAAAGCAAAACAAAACCAAAAGAAGTATCAGAGCACTGAAAATATGCTTAGCAATA
NC_000001.11:33 ------------------------------------------------------------
NC_000023.11:15 ------------------------------------------------------------
NC_000004.12:c1 ------------------------------------------------------------


NC_000013.11:c3 TTAAGCGTGAAAAACAAATTTCAGATATGAAAGATTATCTTGGATTGAAACCACATTGTG
NC_000001.11:33 ------------------------------------------------------------
NC_000023.11:15 ------------------------------------------------------------
```

```
NC_000004.12:c1 ------------------------------------------------------------

NC_000013.11:c3 ATTGAGAAACTTGAAGACAAAGTAACTTCATGAGTCAAAAGATAAGAAACCAACTAGCAA
NC_000001.11:33 ------------------------------------------------------------
NC_000023.11:15 ------------------------------------------------------------
NC_000004.12:c1 ------------------------------------------------------------

NC_000013.11:c3 GAACAGCAGATGTTTTGGGTGGAGCTTTTGTCACAGGCTGGGGTGGGGTAATTCACATCC
NC_000001.11:33 ------------------------------------------------------------
NC_000023.11:15 ------------------------------------------------------------
NC_000004.12:c1 ------------------------------------------------------------

NC_000013.11:c3 AGCCTTCTAACTGTCCTTCCCTCCAGGCCCAACCCATCTCATTCCACCTTCTTCAGGGCT
NC_000001.11:33 ------------------------------------------------------------
NC_000023.11:15 ------------------------------------------------------------
NC_000004.12:c1 ------------------------------------------------------------

NC_000013.11:c3 CCTCATTAGCTTCAAGATAAAACCTGAATTTCTTAAAGGGAATATAAGATGACTCTACCT
NC_000001.11:33 ------------------------------------------------------------
NC_000023.11:15 ------------------------------------------------------------
NC_000004.12:c1 ------------------------------------------------------------

NC_000013.11:c3 ATCTCCCACCTTGTATCTCAAAGTTTCCTTCTTGGAAGCCTTTCTTGACTCCCCTTAAAA
NC_000001.11:33 ------------------------------------------------------------
NC_000023.11:15 ------------------------------------------------------------
NC_000004.12:c1 ------------------------------------------------------------

NC_000013.11:c3 AAAAAAATGGTGATTTTTTCCATGGGTGCTCCCAAAACACCAAAATACACAATACATTTC
NC_000001.11:33 ------------------------------------------------------------
NC_000023.11:15 ------------------------------------------------------------
NC_000004.12:c1 ------------------------------------------------------------

NC_000013.11:c3 TGTATGTATGTAGGTATGTATGTATGTCTTTATTTTGGAGGCAGGGTCTTGCTATGTTGC
NC_000001.11:33 ------------------------------------------------------------
NC_000023.11:15 -----------------------------------------CAGAATTCCGCC-------
NC_000004.12:c1 -----------------------------------------CTGAACATCGCC-------

NC_000013.11:c3 CCAGGCTGCAGTGCAGTGGCTATTCACAGGTGTGATCATAGCACACTGCAGCCTTAAACT
NC_000001.11:33 ------------------------------------------------------------
NC_000023.11:15 ------------------------------------------------------------
NC_000004.12:c1 ------------------------------------------------------------

NC_000013.11:c3 CCTGGGCTCAAGCAGTCCTCACTCCATGGCCTCCCTCTCCAGTAGCTGGAATTACAGACA
NC_000001.11:33 ---------------------------------------CAGCTGCTGAGACCACATA--
NC_000023.11:15 ---------------------------------------CCAAGATCAAATCCACAAA--
NC_000004.12:c1 ---------------------------------------CAAAGATCAAAAGTGAACA--
                                                       * .  ......  .. * *

NC_000013.11:c3 CTACGGTATCACCCAGCCCTGCAATACCTTTATTATTGTTTTCCCTGCTGCAAGTTCTTT
NC_000001.11:33 -----------TCTGGACCCTCAGTGCC--------------------------------
NC_000023.11:15 ------------------CCCCGGCATCTCTAT---------------------------
NC_000004.12:c1 ------------------CCCTGGCCTATCCAT---------------------------
                                  *. .... .

NC_000013.11:c3 GAGCTCGGACAGTCTTGTTGACCTGTGCAGCACCTAAGCTTAGCACCTAGGAGTTTCTCA
```

```
NC_000001.11:33 ------------------------------------------------------------
NC_000023.11:15 ------------------------------------------------------------
NC_000004.12:c1 ------------------------------------------------------------


NC_000013.11:c3 AAAAAAAAAAAAAAAAAAAAAAGAAGCTGAGTAGGATCGGTGACCCTACAGAATTGTATT
NC_000001.11:33 ------------------------------------------------------------
NC_000023.11:15 ------------------------------------------------------------
NC_000004.12:c1 ------------------------------------------------------------


NC_000013.11:c3 ATAGCAGCCGCAAATGAAGATGGCTAATTAAACAAATCTGTAGGCTGGCAACAGAAATGT
NC_000001.11:33 --------------------------------------------CTGGCAACAGC-----
NC_000023.11:15 --------------TGGAGAC-----------------------GTGGCAAAAAA-----
NC_000004.12:c1 --------------TGGGGAT-----------------------ACTGCAAAGAA-----
                                                             . **** ..

NC_000013.11:c3 TACTTTCCTAACAGAAGAGGAACCTTATCCATAAAAGATACAGTATACAGACTATAATAA
NC_000001.11:33 ------------------------------------------------------------
NC_000023.11:15 ------------------------------------------------------------
NC_000004.12:c1 ------------------------------------------------------------


NC_000013.11:c3 CGATTTATTATTTAATAAAATGATTTATTTTATTATTTATTCATATATTTTTATTGTGTG
NC_000001.11:33 ------------------------------------------------------------
NC_000023.11:15 ------------------------------------------------------------
NC_000004.12:c1 ------------------------------------------------------------


NC_000013.11:c3 CTTACTAAGGAGTCAGCCTCTATGCTAAACACTTTATCTACATTACCTTATTTGCATTTA
NC_000001.11:33 ------------------------------------------------------------
NC_000023.11:15 ------------------------------------------------------------
NC_000004.12:c1 ------------------------------------------------------------


NC_000013.11:c3 TCCTAAACTTTTTTTTTTTATTTTTTGAGACAGAGTCTTGCTCTGTCACCCAGGCTGGAG
NC_000001.11:33 -----------------------------------------TCTG---------------
NC_000023.11:15 ------------------------------------------------------------
NC_000004.12:c1 ------------------------------------------------------------


NC_000013.11:c3 TGCAGTGGCGCGATCTCGGCTCACTGCAAGCTCCACCTCCCGGGTTCACGCCATTCTCCT
NC_000001.11:33 ------------------------------------------------------------
NC_000023.11:15 ------------------------------------------------------------
NC_000004.12:c1 ------------------------------------------------------------


NC_000013.11:c3 GCCTCAGCCTCCTGAGTAGCTGGCACTACAGGCATGTGGCACCACGCCTAGCTAATTTTC
NC_000001.11:33 ------------------------------------------------------------
NC_000023.11:15 ------------------------------------------------------------
NC_000004.12:c1 ------------------------------------------------------------


NC_000013.11:c3 TTTTTTTTTGTATTTTTAGTAGAGACGGGGTTTCACTATGTCAGCCAGGATGGTCTCGAT
NC_000001.11:33 ---------------------------------------TGCAGGCAGGATG--------
NC_000023.11:15 ---------------------------------------GCTGGGTGAGATG--------
NC_000004.12:c1 ---------------------------------------ATTGGGTGAAATG--------
                                                         ..* ....***

NC_000013.11:c3 CTCCTGACCTCATGATCCCTCGCCTCGGCCTCCCAAAGTGCTGGGATTACAGGTGTGAGC
NC_000001.11:33 ------------------------------------------------------------
NC_000023.11:15 ------------------------------------------------------------
NC_000004.12:c1 ------------------------------------------------------------
```

```
NC_000013.11:c3 CACCGCTCCTGGCCTATCCTAAACTTTTATCTGTTTGTTTGTATCCCTCTCCTTCACCTC
NC_000001.11:33 ------------------------------------------------------------
NC_000023.11:15 ------------------------------------------------------------
NC_000004.12:c1 ------------------------------------------------------------

NC_000013.11:c3 CTTTATATCTCATCAACTCTGTTAAGAGTGTGGAGACTAAGGCCGGACGCCATGGCTCAC
NC_000001.11:33 ------------------------------------------------------------
NC_000023.11:15 ------------------------------------------------------------
NC_000004.12:c1 ------------------------------------------------------------

NC_000013.11:c3 GCCTGCAATCCCAGCACTTTGGGAGATGGGTGGATTGCTTGACACCAGGAGTTTGAGACC
NC_000001.11:33 ------------------------------------------------------------
NC_000023.11:15 ------------------------------------------------------------
NC_000004.12:c1 ------------------------------------------------------------

NC_000013.11:c3 AGCCTGGCCAGCATGGTGAAACCCCATCTCTATTAAAAATACAAAAATTAGCCGGGTATG
NC_000001.11:33 ------------------------------------------------------------
NC_000023.11:15 ------------------------------------------------------------
NC_000004.12:c1 ------------------------------------------------------------

NC_000013.11:c3 GTGGCAGGCACCTGTAATCCCAGCTACTTGGGAAGCTGAGGCAGGTGAATCACTTGAATC
NC_000001.11:33 ------------------------------------------------------------
NC_000023.11:15 ------------------------------------------------------------
NC_000004.12:c1 ------------------------------------------------------------

NC_000013.11:c3 CAGGAGGCAGAGGTTGCAGTGAGGTGGAGGTTGCAGTGAGCCGGGATTGTGCCACTGTAT
NC_000001.11:33 ------------------------------------------------------------
NC_000023.11:15 ------------------------------------------------------------
NC_000004.12:c1 ------------------------------------------------------------

NC_000013.11:c3 CCCAGCCTGGGTGACAGAGTGAGACCCTGTCTCAAAATAAAATAAAATAAAATAAAATAA
NC_000001.11:33 ------------------------------------------------------------
NC_000023.11:15 ------------------------------------------------------------
NC_000004.12:c1 ------------------------------------------------------------

NC_000013.11:c3 ATAAAATAAAGTAAAGTAAAATCTGCATACCTAGATAATAATGCAACACTGCAAATACAG
NC_000001.11:33 ------------------------------------------------------------
NC_000023.11:15 ------------------------------------------------------------
NC_000004.12:c1 ------------------------------------------------------------

NC_000013.11:c3 TAAAAAATGTGACTGTACATGTTACGCAAGTGAATTTTATGATGTGCCTATACATCCATG
NC_000001.11:33 ------------------------------------------------------------
NC_000023.11:15 ------------------------------------------------------------
NC_000004.12:c1 ------------------------------------------------------------

NC_000013.11:c3 CAGTATGTACATTATATCTCAATAAAACTGGTTTTAAAATCCTATGTAAGGTTTTCTATG
NC_000001.11:33 ------------------------------------------------------------
NC_000023.11:15 ------------------------------------------------------------
NC_000004.12:c1 ------------------------------------------------------------

NC_000013.11:c3 TAAATAAACTAAAAATGTCTAGAATAACTGTTAATTGCAAGGAAGGAACTGAGATGGGGG
NC_000001.11:33 ------------------------------------------------------------
NC_000023.11:15 -------------------TGGAATAA---------------------------------
```

```
NC_000004.12:c1 -------------------TGGTCTGA---------------------------------

NC_000013.11:c3 ATGGGCCTTAAGGAGAACAAGTTTGATTTTTTTTATCTGTGCACTTCTGTATCTGAATGT
NC_000001.11:33 ------------------------------------------------------------
NC_000023.11:15 ------------------------------------------------------------
NC_000004.12:c1 ------------------------------------------------------------

NC_000013.11:c3 TTTCCAGTGAGCATATGTGTTAACTTGTATATTTTTGGTCTGTTTTATTCACTGCTGTAA
NC_000001.11:33 ------------------------------------------------------------
NC_000023.11:15 ------------------------------------------------------------
NC_000004.12:c1 ------------------------------------------------------------

NC_000013.11:c3 TCCTAGCACCCAGATCAGTGCCTACACGTAGCAGAGGCTCAATAAATATTTGTTAAGTGA
NC_000001.11:33 ------------------------------------------------------------
NC_000023.11:15 ------------------------------------------------------------
NC_000004.12:c1 ------------------------------------------------------------

NC_000013.11:c3 ATAAATAAATGAATTATACTTTAAATGTAAAAAAACCCAAGTGGAGTACATAATAACTTG
NC_000001.11:33 ------------------------------------------------------------
NC_000023.11:15 ------------------------------------------------------------
NC_000004.12:c1 ------------------------------------------------------------

NC_000013.11:c3 ATTGGTTTGTGTATGGCTGACTCAAACTTTCTAATTTTTAAAATTCATTTCTAAAATAGA
NC_000001.11:33 ------------------------------------------------------------
NC_000023.11:15 ------------------------------------------------------------
NC_000004.12:c1 ------------------------------------------------------------

NC_000013.11:c3 TTTATGGAACCAATGCTTTTTAGCAATTTGAGAACTATTCTTGGAATACTCTGAATGGGT
NC_000001.11:33 ------------------------------------------------------------
NC_000023.11:15 ------------------------------------------------------------
NC_000004.12:c1 ------------------------------------------------------------

NC_000013.11:c3 CCTGGCTTAAAAAATCCAAGCACTGAATTTGATTTTTGGCTGTAAAATTTGGCTATGAAA
NC_000001.11:33 ------------------------------------------------------------
NC_000023.11:15 ------------------------------------------------------------
NC_000004.12:c1 ------------------------------------------------------------

NC_000013.11:c3 GGAGTTTCCCCAGTATATCTTTTAATCAATCTGCTATAAAGCATGTACAATCATTTATTT
NC_000001.11:33 ------------------------------------------------------------
NC_000023.11:15 ------------------------------------------------------------
NC_000004.12:c1 ------------------------------------------------------------

NC_000013.11:c3 TTCTTATCTAATATGAGAAAATGAGGTGTTGTAACTGAACATAGCTGTAACTATGTTCAA
NC_000001.11:33 ------------------------------------------------------------
NC_000023.11:15 ------------------------------------------------------------
NC_000004.12:c1 ------------------------------------------------------------

NC_000013.11:c3 TAGTGACTTGATTTGTATTTGGTATTGCCTTTACATTATCTGATTGATGATTTTTTTAAA
NC_000001.11:33 ------------------------------------------------------------
NC_000023.11:15 ------------------------------------------------------------
NC_000004.12:c1 ------------------------------------------------------------

NC_000013.11:c3 CAATAGTGAAAGTAAATTTACCAGTAGTTTGCAATCCAGGTTAAAATCTGTATCTGAACA
```

```
NC_000001.11:33 ------------------------------------------------------------
NC_000023.11:15 ------------------------------------------------------------
NC_000004.12:c1 ------------------------------------------------------------


NC_000013.11:c3 CCTCCTTCCCTGATGTACTTGCACGTATATTAAGGTAAATACTTGTTGACACATATCTAA
NC_000001.11:33 ------------------------------------------------------------
NC_000023.11:15 ------------------------------------------------------------
NC_000004.12:c1 ------------------------------------------------------------


NC_000013.11:c3 AATGCTGAAATAATATTGTCTATTAGTGTTTAAATGTGGTTACTAATTATGTGATGATTA
NC_000001.11:33 ------------------------------------------------------------
NC_000023.11:15 ----------------------------TTTAAATG------------------------
NC_000004.12:c1 ------------------------------------------------------------


NC_000013.11:c3 TTTACAACAATCAACCTTTTATCTTGCTATTGCTAAAATGGAAGTTTTTAATTTCTATTT
NC_000001.11:33 ------------------------------------------------------------
NC_000023.11:15 ------------------------------------------------------------
NC_000004.12:c1 ------------------------------------------------------------


NC_000013.11:c3 AATGCATTTATAAATATATAAAATAATATATTGATTTTAATCTTACCCTTCTTCTAAAAC
NC_000001.11:33 ------------------------------------------------------------
NC_000023.11:15 ------------------------------------------------------------
NC_000004.12:c1 ------------------------------------------------------------


NC_000013.11:c3 AGGGGTTCTTAAGCCTTTTGGAGATCCTTAAGGATCTGATAAAAGCAGTGAGGCCTTGCA
NC_000001.11:33 ------------------------------------------------------------
NC_000023.11:15 ------------------------------------------------------------
NC_000004.12:c1 --------------------------------------------GCAGTCAG--------


NC_000013.11:c3 CTGTAAAAACGTACACATTTAGGTTACAGTGAAGGCCAGCCACTGCACTCCAGCCCAGGC
NC_000001.11:33 --------AAGCACAGGCTTAGGTC-----------------------------------
NC_000023.11:15 --ACAGTGAAAAGCAGCCTTACATCACTAAGGCGG-------------------------
NC_000004.12:c1 --CCAAAGATAAACAACCATATGAACAGAAAGCAG-------------------------
                        * . .**  . ** .

NC_000013.11:c3 AACAGAGCAAGACTTTGTCTCTAAACTAAACTAAACTAAACTAGATGTACACAGTTGAGG
NC_000001.11:33 ------------------------------------------------------------
NC_000023.11:15 ------------------------------CAAAGCTGAAGGAGAAGTA-----------
NC_000004.12:c1 ------------------------------CTAAGCTAAAGGAGAAATA-----------


NC_000013.11:c3 ATGGTTCCAAACCTAAGGTTAATAACTCTTGTTCTAAATCATTCAAATGCATGGAGCTAT
NC_000001.11:33 ------------------------------------------------------------
NC_000023.11:15 ------------------------------------------------------------
NC_000004.12:c1 ------------------------------------------------------------


NC_000013.11:c3 CCATAGATACTTCCACATCCAATTTTACATATATATATAGCTTAAAAGTATGTAATTTGA
NC_000001.11:33 ------------------------------------------------------------
NC_000023.11:15 ------------------------------------------------------------
NC_000004.12:c1 ------------------------------------------------------------


NC_000013.11:c3 CCAAGGTTCCTAGCTTTTTATATTGATGGTGACTTTCACATTTAACCTGTGGCTCTTCAG
NC_000001.11:33 ------------------------------------------------------------
NC_000023.11:15 ------------------------------------------------------------
NC_000004.12:c1 ------------------------------------------------------------
```

```
NC_000013.11:c3 GGTATCCAATTCATTTATTAGAATATTATACTCAGACACAGTGGGTCACATCTGTAATCT
NC_000001.11:33 ------------------------------------------------------------
NC_000023.11:15 ------------------------------------------------------------
NC_000004.12:c1 ------------------------------------------------------------

NC_000013.11:c3 CACATTTTGGGAGGCCAAGGTGGGAGGATCACTTCAGGAGGAGTTCAAGACTAGTCTGGG
NC_000001.11:33 ------------------------------------------------------------
NC_000023.11:15 -------TGAGAAGGTAAGGTGGGG-----------------------------------
NC_000004.12:c1 -------TGAAAAGGTACAGTGTCA-----------------------------------

NC_000013.11:c3 CAACACTGCAATACCCCATGTCTGCAAAAAAATTTTTTAAAAAATTAGCCAGGTGTGGGC
NC_000001.11:33 ------------------------------------------------------------
NC_000023.11:15 ------------------------------------------------------------
NC_000004.12:c1 ------------------------------TCTTTTTTA---------------------

NC_000013.11:c3 TGAGTGCAGTGGCTCACACCTGTAATCCCAGCACTTTGGGAGGCCAAGGCAAGAAAATCA
NC_000001.11:33 ------------------------------------------------------------
NC_000023.11:15 ------------------------------------------------------------
NC_000004.12:c1 ------------------------------------------------------------

NC_000013.11:c3 CTTGAGGCCAGGAGTTCTAGACCAGCCTGGCTAACATGGCGAAACCCCATCCCACCTTCT
NC_000001.11:33 ------------------------------------------------------------
NC_000023.11:15 ------------------------------------------------------------
NC_000004.12:c1 ------------------------------------------------------------

NC_000013.11:c3 CAGGAGGCTGAGGCAGGAGAATCCCTTGAACCCGGGAGGTGGAGCTTGCAGTGAGCCAAG
NC_000001.11:33 ------------------------------------------------------------
NC_000023.11:15 ------------------------------------------------------------
NC_000004.12:c1 ------------------------------------------------------------

NC_000013.11:c3 ATCATGCCATTGCACTCCAGCCTGGGCGACAGAGCAAGAATCCATCTCAAAAAAAAAAAA
NC_000001.11:33 ------------------------------------------------------------
NC_000023.11:15 ------------------------------------------------------------
NC_000004.12:c1 ------------------------------------------------------------

NC_000013.11:c3 AAAGAAATTAAAAAACAGAAGTTAGCTGGGTGTGATGCTGCACACCTGTGATCCCAGCTA
NC_000001.11:33 ------------------------------------------------------------
NC_000023.11:15 ------------------------------------------------------------
NC_000004.12:c1 ------------------------------------------------------------

NC_000013.11:c3 CTCAGGAGGCTGAGGCAGGAGAATCGCTTGAACCCAGGAGGCAGAGGTTGAATTGAGCTG
NC_000001.11:33 --------------------------------------AAGCAAT---------------
NC_000023.11:15 --CTGGAAGCCTGGACTGGTGAAC--------------AGGCAGTGGTT-----------
NC_000004.12:c1 --------------------------------------AAGCCGTGGAT-----------
                                                      *.** .

NC_000013.11:c3 AGATCATGCCACTGCACTCCAGCCTGGGCAACAGAATGAGACTCTGTCTCAAAAAAAAAA
NC_000001.11:33 ------------------------------------------------------------
NC_000023.11:15 ------------------------------------------------------------
NC_000004.12:c1 ------------------------------------------------------------

NC_000013.11:c3 AAAAAAAAAAAAATTAGCCAGGTGAGGGAGCACACACATGTAGTCCCAGCTATTTGGGAG
NC_000001.11:33 ------------------------------------------------------------
NC_000023.11:15 ----------------------------------------------CTGCTATCAGTAGG
```

```
NC_000004.12:c1 ---------------------------------------------------------AAG

NC_000013.11:c3 GCTGAGTGGGGAGGATTGCTTGAGCCCAGGAATTCAGGGCTGAGGTGAGCCATGATTGCA
NC_000001.11:33 ------------------------------------------------------------
NC_000023.11:15 TTGCAGGGCATGTGGCAGCTT-----------TGCTGGGCTGAG----------------
NC_000004.12:c1 ACTAGGTATAGGTAA---------------------------------------------

NC_000013.11:c3 CCACTGCACTCCAGCCTGAGCAACAGAATGGGACCCTGTCTCTCAAAACACACACACAAG
NC_000001.11:33 ------------------------------------CAGCCGTCAAAATCCACA------
NC_000023.11:15 ------------------------------------TACTTAGCATAGCGCTCA------
NC_000004.12:c1 ------------------------------------TAACTGTAGAAA------------
                                                    .. ..   . *.

NC_000013.11:c3 AATATCATACTCAACTTGCATCTCCTGGTGTCTTAGTTCATTTTCTGTTGCTAAGACAGA
NC_000001.11:33 ------------------------------------------------------------
NC_000023.11:15 ------------------------------------------------------------
NC_000004.12:c1 ------------------------------------------------------------

NC_000013.11:c3 ATACCTGAGACTCGGTAATTTACAAATTAAAGAAGTTTATTTGACTTATGGTTCAGGAGG
NC_000001.11:33 ------------------------------------------------------------
NC_000023.11:15 ------------------------------------------------------------
NC_000004.12:c1 ------------------------------------------------------------

NC_000013.11:c3 CCAGGTAGTCCAAGAACATGGCAGCAGCTTCTGGTGCAAGCTTTCGTGCTGCATCTTAAC
NC_000001.11:33 ------------------------------------------------------------
NC_000023.11:15 ------------------------------------------------------------
NC_000004.12:c1 ------------------------------------------------------------

NC_000013.11:c3 ATGGTGGAAAAGCAGAAGGACAAGCAGGACATGCAAAAGAGACTGCAAGAGTGAGCCAAG
NC_000001.11:33 ------------------------------------------------------------
NC_000023.11:15 ------------------------------------------------------------
NC_000004.12:c1 ------------------------------------------------------------

NC_000013.11:c3 CTAGCTTTTATAACAACCTGCTCTGGAGAGGGCTCACTCCTGTGAGAGCCAATTCACTCC
NC_000001.11:33 --------------------------------CTCTCACCTGTGAGTGGCTGTTCAACC-
NC_000023.11:15 ----------------------------AGGGCCCATCCCCCTGCAAGGTAG--------
NC_000004.12:c1 -------------------------------------ACCTGGGAAATTTAG--------
                                                      **.  * .   . .

NC_000013.11:c3 CTCGAGAACTCACTTCACCTGATGGCATTAATTTATACCTGAGGGCTCTACCCTCATGAC
NC_000001.11:33 ------------------------------------------------------------
NC_000023.11:15 ------------------------------------------------------------
NC_000004.12:c1 ------------------------------------------------------------

NC_000013.11:c3 CCAATCATCTCTTAAAGGCCCCATCTCTTAATACTGTTACATTGGCAATTAAGCTTCCAA
NC_000001.11:33 ------------------------------------------------------------
NC_000023.11:15 ------------------------------------------------------------
NC_000004.12:c1 ------------------------------------------------------------

NC_000013.11:c3 GACAGGAACTTTGGGGAGACACATTTCCCCAGGCCCGAGGAACTCAGATGGCTCTAGGAT
NC_000001.11:33 ------------------------------------------------------------
NC_000023.11:15 ------------------------------------------------------------
NC_000004.12:c1 ------------------------------------------------------------

NC_000013.11:c3 GATTGACAGACAGGCTTCAGATTCACTTTAACCAAATCCCAGTGGCTTGGGGCAGGGTTT
```

```
NC_000001.11:33 ------------------------------------------------------------
NC_000023.11:15 ------------------------------------------------------------
NC_000004.12:c1 ------------------------------------------------------------


NC_000013.11:c3 AGTTTTGTTTAATTGAATTCAGCAGTTTTCTTAATTCTTCAAACAAAGTCACATAATTAT
NC_000001.11:33 ------------------------------------------------------------
NC_000023.11:15 ------------------------------------------------------------
NC_000004.12:c1 ------------------------------------------------------------


NC_000013.11:c3 TTAAATGTGATATAACTAATGGATTGACTTTTCAAGGTTCCTTTTTGGTCTTAAGATCCT
NC_000001.11:33 TTGAACATG---------------------------------------------------
NC_000023.11:15 CTACGGGTG---------------------------------------------------
NC_000004.12:c1 TTAATCTTG----TATTAATGG--------------------------------------
                .*.    **

NC_000013.11:c3 AAGAAGTCCAAGACTAGCAAATATGGTTTTTAAAGAAGAGGGAATCTACAATTAATGAAG
NC_000001.11:33 ------------------------------------------------------------
NC_000023.11:15 ------------------------------------------------------------
NC_000004.12:c1 ------------------------------------------------------------


NC_000013.11:c3 GTTTATTTCTCATACTTTGTGCTTCTGCGAGAACAAGAAAAGGGTATTGTGGTTTTTAAA
NC_000001.11:33 --TTACTTTTTATGTT--------------------------------------------
NC_000023.11:15 --CTGGTGTTCATGCT--------------------------------------------
NC_000004.12:c1 --TTGTCAGCTATGTT--------------------------------------------
                  .*. .  ..**..*

NC_000013.11:c3 TGTGGTTTGGGAAATGTTCATGACAATGGAAAGATCAGTGTTGGGTCAGACTGTGAGGCT
NC_000001.11:33 ------------------------------------------------------------
NC_000023.11:15 ------------------------------------------------------------
NC_000004.12:c1 ------------------------------------------------------------


NC_000013.11:c3 GCCTGATACAAAGAGAAACTCAGTTTGAAACAAGAAAAAAGAGAAACGGGAAGGACAAAA
NC_000001.11:33 ----------------------------------------------------------AA
NC_000023.11:15 ------------------------------------------------------------
NC_000004.12:c1 ------------------------------------------------------------


NC_000013.11:c3 TGAAGGAAGGGTGAGAGTTGCAATTTTTGAGAAGTAATGATCGAGAGACTTTCTGGTGAA
NC_000001.11:33 TGAA--------------------------------------------------------
NC_000023.11:15 ------------------------------------------------------------
NC_000004.12:c1 ------------------------------------------------------------


NC_000013.11:c3 GAGCCACCCAATCTGGAGATCAGAGATTCAACAGACCCATTTCAGGCCAAAGCACACCTA
NC_000001.11:33 ------------------------------------------------------------
NC_000023.11:15 ------------------------------------------------------------
NC_000004.12:c1 ------------------------------------------------------------


NC_000013.11:c3 TCATTCAGTTGTAGTTAGTAGGAAAACCCAAAGGGTTTGGGATGATTTCTGAACCCATCT
NC_000001.11:33 ------------------------------------------------------------
NC_000023.11:15 ------------------------------------------------------------
NC_000004.12:c1 ------------------------------------------------------------


NC_000013.11:c3 TCGGGGCGAAGAATTTGCTGATTCCAGGCTGGGCACATTGGCTCACACCTGTAAGTGTGA
NC_000001.11:33 ------------------------------------------------------------
NC_000023.11:15 ------------------------------------------------------------
NC_000004.12:c1 ------------------------------------------------------------
```

```
NC_000013.11:c3 GCCAATCCCAGCAGGTTGGGAGGCTGAGGCGGGTGGATCACCTGAGGTCAGGAGTTTGAG
NC_000001.11:33 ------------------------------------------------------------
NC_000023.11:15 ------------------------------------------------------------
NC_000004.12:c1 ------------------------------------------------------------

NC_000013.11:c3 ACCAGCCTAGCCAACATGGCAAAACCTTGTCTCTACTAAAAATATAAAAATTAGCTGGGC
NC_000001.11:33 ------------------------------------------------------------
NC_000023.11:15 ------------------------------------------------------------
NC_000004.12:c1 ------------------------------------------------------------

NC_000013.11:c3 ATGGTAGTGCACACCTGTAATCCCAGCTACTTAGGAGACTAAGGCAGGAAAATCACTTGA
NC_000001.11:33 ------------------------------------------------------------
NC_000023.11:15 ------------------------------------------------------------
NC_000004.12:c1 ------------------------------------------------------------

NC_000013.11:c3 ACCCAGGAGGCGGAGGTTGGAGTGAGCCGAGATTGCACCAGTGCACTCCAGCCTGGGCAA
NC_000001.11:33 ------------------------------------------------------------
NC_000023.11:15 ------------------------------------------------------------
NC_000004.12:c1 ------------------------------------------------------------

NC_000013.11:c3 CAGAGCAAGACTCCTTCTCAAAAAAAAAAAAAACAAAAAAGAGGCTGGGCGCAGTGGCTC
NC_000001.11:33 ------------------------------------------------------------
NC_000023.11:15 ------------------------------------------------------------
NC_000004.12:c1 ------------------------------------------------------------

NC_000013.11:c3 ACACTTGTAATCCCAGCACTTTAGGAGGCTGAGACAGGTGGATCACTTGAGGTCAGAAGT
NC_000001.11:33 ------------------------------------------------------------
NC_000023.11:15 ------------------------------------------------------------
NC_000004.12:c1 ------------------------------------------------------------

NC_000013.11:c3 TCAAGACCAGTCTGGCCAACATGGTGAAACCCTGTCTCTACTAAAAATACAGAAATTAGC
NC_000001.11:33 ------------------------------------------------------------
NC_000023.11:15 ------------------------------------------------------------
NC_000004.12:c1 ------------------------------------------------------------

NC_000013.11:c3 CGGGTATAGTGGCTCATGCCTGTAGTCCCAGCTACTCGGGAGGCTAAGGCAGGAGAATCT
NC_000001.11:33 ------------------------------------------------------------
NC_000023.11:15 ------------------------------------------------------------
NC_000004.12:c1 ------------------------------------------------------------

NC_000013.11:c3 CTTGAACACAGGAGGTGGAGGTTTCAGTGAGCCCAGGTCATGCCACTGCACTCCAGCGTG
NC_000001.11:33 ------------------------------------------------------------
NC_000023.11:15 ------------------------------------------------------------
NC_000004.12:c1 ------------------------------------------------------------

NC_000013.11:c3 GGCCACAAAGCAAGACTTCGTCTTAATAATAATAATAATAATAATAATAATAATAATAAT
NC_000001.11:33 ------------------------------------------------------------
NC_000023.11:15 ------------------------------------------------------------
NC_000004.12:c1 ------------------------------------------------------------

NC_000013.11:c3 AATTTGCTGATTCCTTTGCCTAGATTTGTGTATTTGGGGCAAGAATCATTGAAATTGTGT
NC_000001.11:33 ---------ACTCAGTTTTCTAGTCCTATAAATTTGGGGC--------------------
NC_000023.11:15 -----------TTTTTTTGACAGGTCTTTGTTTTGGGGACAA------------------
```

```
NC_000004.12:c1 ------------------------------------------------------------

NC_000013.11:c3 CCTACTCTATAGCTAGACGTGGGGTGGTGGGGGGCATAAAGGGATAGAGTGAGTGATGTA
NC_000001.11:33 ------------------------------------------------------------
NC_000023.11:15 ------------------------------------------------------------
NC_000004.12:c1 ------------------------------------------------------------

NC_000013.11:c3 GACCTTGCAAAGGAAGCAAGCCTAGCCATCTCTCCTGATACCCACCACAGCTCTTATCCC
NC_000001.11:33 ------------------------------------------------------------
NC_000023.11:15 ------------------------------------------------------------
NC_000004.12:c1 ------------------------------------------------------------

NC_000013.11:c3 TCTCCAGCCCCTCCCGCTCCTGCTGCATCCCAGTGGGACAAGGAAAAGTCAGTGAGAATA
NC_000001.11:33 ------------------------------------------------------------
NC_000023.11:15 ------------------------------------------------------------
NC_000004.12:c1 ------------------------------------------------------------

NC_000013.11:c3 TAAATTCCAAGAGAATACCATCAAACTTGTCCAACAAAAAGTCTTAATTGACCTTGGGAG
NC_000001.11:33 ------------------------------------------------------------
NC_000023.11:15 ------------------------------------------------------------
NC_000004.12:c1 ------------------------------------------------------------

NC_000013.11:c3 GATATATTTTGAGATGGAGTCTCACTCTGTTCCCCAGGCTGGAGTGCAGTGGTGCAATCT
NC_000001.11:33 ------------------------------------------------------------
NC_000023.11:15 ------------------------------CTTCAGGC----------------------
NC_000004.12:c1 ------------------------------------------------------------

NC_000013.11:c3 CGGCTCACTACAACCTCCACCTCCTGAGTTTAAGTGATTGTCCTGCTTCAGCCTCCCAAG
NC_000001.11:33 ------------------------------------------------------------
NC_000023.11:15 ------------------------------------------------------------
NC_000004.12:c1 ------------------------------------------------------------

NC_000013.11:c3 TAGCTGGCAAGAGAAATGCAAATCAAAACCCCAATGAGATACTATCCCACACTAGTCAGA
NC_000001.11:33 ------------------------------------------------------------
NC_000023.11:15 ------------------------------------------------------------
NC_000004.12:c1 ------------------------------------------------------------

NC_000013.11:c3 ATGGCCATCACTAGAAAGTCAAAAAATAACAGATGCTTGCGAGGTTGTGGAGAGAAGGGG
NC_000001.11:33 ------------------------------------------------------------
NC_000023.11:15 ------------------TTGAAAAAT---------------------------------
NC_000004.12:c1 ------------------TTGAAAAGG---------------------------------

NC_000013.11:c3 ATGCTTACACACTGCTGGTAGGAATGTAAATTAGTCCAGCCACTGTGGAAAGCAGTTTGG
NC_000001.11:33 ------------------------------------------------------------
NC_000023.11:15 ------------------------------------------------------------
NC_000004.12:c1 ------------------------------------------------------------

NC_000013.11:c3 CGATTCCTCAAAGAACTTAAAACAGAACTACCATTCCACCCAGCAATTCCATTACTGGGT
NC_000001.11:33 ------------------------------------------------------------
NC_000023.11:15 ------------------------------------------------------------
NC_000004.12:c1 ------------------------------------------------------------

NC_000013.11:c3 ATATACCCAAAGGACTAGAAATCATTCTACCATAAAGACACACACACATGCGTATGTTCA
```

```
NC_000001.11:33 ------------------------------------------------------------
NC_000023.11:15 ------------------------------------------------------------
NC_000004.12:c1 ------------------------------------------------------------


NC_000013.11:c3 TTGCAGCACTCTTCCCAATAGCATAAGACATGGAATGAACCTAAATGCCCATCAGTGGTA
NC_000001.11:33 ------------------TATCATCAACCATTAAGTGATATTAAA---------------
NC_000023.11:15 ------------------TTTCCTTACTCTTTAAGGAAAAAAAAATGTT-----------
NC_000004.12:c1 ------------------------------------------------------------


NC_000013.11:c3 GACTGGATAAGGAAAATGTGGTACATTTACACCATGGAATACTACCCAGCCATAAAAAAG
NC_000001.11:33 ------------------------------------------------------------
NC_000023.11:15 ------------------------------------------------------------
NC_000004.12:c1 ------------------------------------------------------------


NC_000013.11:c3 AATAAGATCATATCCTTTGCAGCAAGATGGATAGAGCTGGAGGTCATTACCCTGAGGGAA
NC_000001.11:33 ------------------------------------------------------------
NC_000023.11:15 ------------------------------------------------------------
NC_000004.12:c1 ------------------------------------------------------------


NC_000013.11:c3 CTAATGCAGGAACAGAAAGTCAGATAGTGCATGTTTCCATCTATAAGTGGGAGCTAAACA
NC_000001.11:33 ------------------------------------------------------------
NC_000023.11:15 ----------AACAGAGGATTTGATAGT--------------------------------
NC_000004.12:c1 ------------------------------------------------------------


NC_000013.11:c3 GTGAGTACACACGGATACAAAGAGGGGAACAAGAGACACTGGGGCCTACTTGAGGGTGGA
NC_000001.11:33 ------------------------------------------------------------
NC_000023.11:15 ------------------------------------------------------------
NC_000004.12:c1 ------------------------------------------------------------


NC_000013.11:c3 GGCTGAGAGGAGGGTGAGGATCAAAAAACTACCCGTCAGGCACCATGCTTATTAACTGGG
NC_000001.11:33 ------------------------------------------------------------
NC_000023.11:15 ------------------------------------------------------------
NC_000004.12:c1 ------------------------------------------------------------


NC_000013.11:c3 TAACAAAATAATCTGTACACCAAGCCCCCATGACACAAAATTTACCTATATAACAAACCT
NC_000001.11:33 -------------------------CCCAGTGCCACACA---------------------
NC_000023.11:15 -------------------------CCTACTAATGTGAC---------------------
NC_000004.12:c1 -------------------------CCTAATGA---------------------------
                                         **.  *.

NC_000013.11:c3 GCATGTGTATCCCCGAACCTAAAATGAAAGTTAAAAAAAAAAAACAGCCTGGCCAACATG
NC_000001.11:33 ------------------------------------------------------------
NC_000023.11:15 ------------------------------------------------------------
NC_000004.12:c1 ------------------------------------------------------------


NC_000013.11:c3 GTGAAATCCCATCTCTACTAAAAATACAAAAAGAAATTAGCTGGGTGTAGTGGCAGGCAC
NC_000001.11:33 ---------------------------------------------------GGCAAGTAC
NC_000023.11:15 -------------------------------------------------TTGAAGGACAC
NC_000004.12:c1 ------------------------------------------------------------


NC_000013.11:c3 CTGTAATCCCAGCTACTCGGGAGGCTGAGGCAGGAGAATCGCTTGAACCCGGAAGGCGGA
NC_000001.11:33 CTA---------------------------------------------------------
NC_000023.11:15 TTGAACACCCACTAACT-------------------------------------------
NC_000004.12:c1 ------------------------------------------------------------
```

```
NC_000013.11:c3 GGTTGCAGTGAGCCGAGATCGCACCACTGTACTCAAGCCCGGGTGACAGAGAATCAGTGG
NC_000001.11:33 ------------------------------------------------------------
NC_000023.11:15 -------------------------------TTTAAATTCGAATGGCTAAGAACCA----
NC_000004.12:c1 ------------------------------------------------------------


NC_000013.11:c3 TAGATCCAAAAATGGAATGAGTGCGGCATAATCATTCGAAGTGTTCAGTGTGAGTGAAGA
NC_000001.11:33 -------------------------------------GCAGTGTCCA-------------
NC_000023.11:15 -------------------------------------AATTCATATA-------------
NC_000004.12:c1 -------------------------------------AAATTGTACA-------------
                                                     .   ..* .*

NC_000013.11:c3 GAGGGCAGGGTTGGTGCAGTAGTATTGACAAGTACATCTGCATTGAAGGGTCCTGGTTAC
NC_000001.11:33 ------------------------------------------------------------
NC_000023.11:15 ------------------------------------------------------------
NC_000004.12:c1 ------------------------------------------------------------


NC_000013.11:c3 AGTGAAACGTGAAGAGGAAGGAGCCAGCGAAAAGAGATAGGGGATCATTTTGGAAAATGA
NC_000001.11:33 ------------------------------------------------------------
NC_000023.11:15 ------------------------------------------------------------
NC_000004.12:c1 ------------------------------------------------------------


NC_000013.11:c3 GATGACTGAGGATCAAGGTCTCAGTTTATAATTGAAGGGGAGAAAGACTGAAGGCTGGGG
NC_000001.11:33 ------------------------------------------------------------
NC_000023.11:15 ------------------------------------------------------------
NC_000004.12:c1 ------------------------------------------------------------


NC_000013.11:c3 AGGGGATGGCTTTCGTGTGTAGAGGAATATGAGCACTGAATTTCTAAGAAGAGAAGAATA
NC_000001.11:33 ------------------------------------------------------------
NC_000023.11:15 ------------------------------------------------------------
NC_000004.12:c1 ------------------------------------------------------------


NC_000013.11:c3 AAAGAGACACTACTTGTGAGGCTCCGGGCATGACGGGAATATTGGTGGGCGGTGTGTGTG
NC_000001.11:33 -----------------AAGGCCCCCAGAATG----------------------------
NC_000023.11:15 -------------TGGAAACACTTCAGATATA----------------------------
NC_000004.12:c1 ------------------------------------------------------------


NC_000013.11:c3 GTGTGTGTGTTGCAGGGAGCAGGCATTGATCTGATGGAAGGAATGTCTGTGTGGAAGAGT
NC_000001.11:33 ------------------------------------------------------------
NC_000023.11:15 ------------------------------------------------------------
NC_000004.12:c1 ------------------------------------------------------------


NC_000013.11:c3 AAATAGCCACCAGCCTGAAGTTGACCAGTAAAAAATCCCAAAACACATTCAACTGAGTCT
NC_000001.11:33 ------------------------------------------------------------
NC_000023.11:15 ------------------------------------------------------------
NC_000004.12:c1 ------------------------------------------------------------


NC_000013.11:c3 TATCCTCTTAAATGACTCCAAGTTTCAAGAAAATTTGCCTGGACAAGCAATCCTCTTCCT
NC_000001.11:33 ------------------------------------------------------------
NC_000023.11:15 ------------------------------------------------------------
NC_000004.12:c1 ------------------------------------------------------------


NC_000013.11:c3 CACCCATTAAAGATACTGCATTTTACTTTTGAAAAGCCAACAACAAAGATGCAACACCTT
NC_000001.11:33 -----------------------------------------------------ACACTTT
NC_000023.11:15 -------------------------------------------TATGATAGCAGCACTGT
```

```
NC_000004.12:c1 ------------------------------------------------CTTCAACACAAG
                                                                     .***

NC_000013.11:c3 TTTAATAAAGAATCTCCCCCTTTGATGGCTTAGATTTTTAGCTGCCTAGAGAAAGGCAAG
NC_000001.11:33 TTCA--------------------------------------------------------
NC_000023.11:15 CTTA--------------------------------------------------------
NC_000004.12:c1 GTAA--------------------------------------------------------
                 * *

NC_000013.11:c3 CTAGGACGCTGCTTTCTTGGTCCATTCCCCTATACGCTTTTAGGTAAGGTTTTTGTTTGA
NC_000001.11:33 ------------------------------------------------------------
NC_000023.11:15 ------------------------------------------------------------
NC_000004.12:c1 ------------------------------------------------------------


NC_000013.11:c3 TGCACAGTGTCTGAACTGCCATGTACTTTTGCAAAAGTGCATTTCCTGGGTGTGTGTGTT
NC_000001.11:33 ------------------------------------------------------------
NC_000023.11:15 ------------------------------------------------------------
NC_000004.12:c1 ------------------------------------------------------------


NC_000013.11:c3 TTATGGCAATTGCTACCATTTCTTCTAATCACTTGCCACAGGTGTCCCATAGCTATCAGG
NC_000001.11:33 ------------------------------------------------------------
NC_000023.11:15 ------------------------------------------------------------
NC_000004.12:c1 ------------------------------------------------------------


NC_000013.11:c3 GGTAGAACTGAGGGTGCAGCGGGGAGGCCCATGTGCCAGGCTGTGAGTCTAGGCAGGTTG
NC_000001.11:33 ------------------------------------------------------------
NC_000023.11:15 ------------------------------------------------------------
NC_000004.12:c1 ------------------------------------------------------------


NC_000013.11:c3 TTGGAAACGCAGTTGTTTACTAGGCCTTGGTGTTTGGTACATTACTGATATGAAGCCTGG
NC_000001.11:33 ------------------------------------------------------------
NC_000023.11:15 ------------------------------------------------------------
NC_000004.12:c1 ------------------------------------------------------------


NC_000013.11:c3 AGGGAGGACTGGTGTGCAGATGATCTGCATCTGCTATCAGGAGCAAGAAACATTGAATAA
NC_000001.11:33 ------------------------------------------------------------
NC_000023.11:15 ------------------------------------------------------------
NC_000004.12:c1 ------------------------------------------------------------


NC_000013.11:c3 ATAGGCAAAATTGCTAAGGAAAAAAATGCTCTCCTAACTTCAATTCTTTTCCTTTTCCTT
NC_000001.11:33 --------------------------------------------TCTTTTCTCTTCCCTC
NC_000023.11:15 --------------------------------CTTGATTTCAATTCCTGTCCTCTTACCT
NC_000004.12:c1 ---------------------------------------TTGAAACCTTCCTTTTGACTG
                                                             *.* .*...*  *.

NC_000013.11:c3 CCTTTGGGCACCCCCATCTTCAGGTGCCCAATAGTGTCATAAAAATGTATTTCTTAAAAA
NC_000001.11:33 CAATTG------------------------------------------------------
NC_000023.11:15 GAGATG------------------------------------------------------
NC_000004.12:c1 AAACCA------------------------------------------------------
                    ..

NC_000013.11:c3 ACCCTAAGTGTTTGAGTTGACTCCAAGTTAATATATATCAACATTAAAACTAAAAAAGTT
NC_000001.11:33 ------------------------------------------------------------
NC_000023.11:15 ------------------------------------------------------------
NC_000004.12:c1 ------------------------------------------------------------


NC_000013.11:c3 AAAGGCATATTAGGCTGAGTTATAGTAGTAAAACAGCAGAGCAAAGGATACAATGGTCCC
```

```
NC_000001.11:33 ------------------------------------------------------------
NC_000023.11:15 ------------------------------------------------------------
NC_000004.12:c1 ------------------------------------------------------------

NC_000013.11:c3 ACTCTAGATGGTTGTAGAGTCTAGATGATTATATTCTGTTTTAGGCATCCTGTTTTGATA
NC_000001.11:33 ------------------------------------------------------------
NC_000023.11:15 ----------------AGGACTGCATGTTTCTACTTTGTGTTAA----------------
NC_000004.12:c1 -------------------------------------GTGTTTG----------------

NC_000013.11:c3 AGGACACTGACCAACTGAACCACAATCAGAGAAAAGCAGCTAGGGTCTGGGAACCACATT
NC_000001.11:33 ------------------------------------------------------------
NC_000023.11:15 ------------------------------------------------------------
NC_000004.12:c1 ------------------------------------------------------------

NC_000013.11:c3 ACCTGAGAAATGACTCAAGGCCTGGAAAAAGAAGACCGATAGTTATCTTTATATATTAAA
NC_000001.11:33 ------------------------------------------------------------
NC_000023.11:15 ------------------------------------------------------------
NC_000004.12:c1 ------------------------------------------------------------

NC_000013.11:c3 GGGTTGTCTGCCACATAGTAAAGGGAAATATTGGCCGGGTGCAGTGGCTCACACCTGTAA
NC_000001.11:33 ------------------------------------------------------------
NC_000023.11:15 -----AACAGCCGCATACTCATTTGAAATGT-----------------------------
NC_000004.12:c1 ------------------------------------------------------------

NC_000013.11:c3 TCCCAGCACTTTGGGAGGCCAACGCGGGCGGATCACAGGGTCAGGAGATCGAGACCATCC
NC_000001.11:33 ------------------------------------------------------------
NC_000023.11:15 ------------------------------------------------------------
NC_000004.12:c1 ------------------------------------------------------------

NC_000013.11:c3 TGGCTAACACGGTGAAACCCCGTCTCTATTAAAAATATAAAAAATTAGCCAGGCGTGGCG
NC_000001.11:33 ------------------------------------------------------------
NC_000023.11:15 ---------------------GTCCCTGTT------------------------------
NC_000004.12:c1 ------------------------------------------------------------

NC_000013.11:c3 GCACGCGCCTGTAGTCCCAACTATTCGGGAGGCTGAGGCGGGAGAATGGTGTGAACCCGG
NC_000001.11:33 ------------------------------------------------------------
NC_000023.11:15 -------------------------------------------------------CCTCT
NC_000004.12:c1 ------------------------------------------------------------

NC_000013.11:c3 GAGGCAGAGCTTGCAGTGAGCCGAGATGGTGCCACTGCACTCCAGCCTGGGCGACAGAGC
NC_000001.11:33 ------------------------------------------------------------
NC_000023.11:15 AGGATGTTGCTGACTATAAGTCG-------------------------------------
NC_000004.12:c1 ------------------------------------------------------------

NC_000013.11:c3 GAGACTCCGTCTCCAAAAAAAAAAAAAAAAGAAAAGAAAAAGAAAAGGGAAATATTCAGTG
NC_000001.11:33 -------------------------------------AAAGACTAGTGGAATATCTGCAA
NC_000023.11:15 -------------------------------------AAAGGAAAGTTTGATGGTGCAAA
NC_000004.12:c1 -------------------------------------TAGCACTAGTATATTCCTGCAGA
                                      *. .  **   . *  .    .

NC_000013.11:c3 TCGCTCTGGAGGACAGAGCTAGGACCAATGGGAGGGAGTGACCAGGAAATATATATTGGA
NC_000001.11:33 TTACTTTG----------------------------------------------------
NC_000023.11:15 GGGTCCTG----------------------------------------------------
NC_000004.12:c1 CAGACTTG----------------------------------------------------
                  . ..**
```

NC_000013.11:c3 TTAAAATAAGAAAGAAATTTCTACGAATTAGAGTTGTTCAAAAGGAGAGTGGAAAAAAGC
NC_000001.11:33 --
NC_000023.11:15 ---------------------------CTAAAGTTGCCCGGAAAAAG-------------
NC_000004.12:c1 --

NC_000013.11:c3 AAACTGCAGAACAGCATTAGAAGTACAATCCTATTTGACTTTGAAATTGCACACAGAAGT
NC_000001.11:33 --
NC_000023.11:15 --
NC_000004.12:c1 --

NC_000013.11:c3 CCTTGAACAGTGGAGAGACAGGATCATTGCTTTAGAAAAACGATACTGGTGGCAAGCTAG
NC_000001.11:33 --
NC_000023.11:15 --
NC_000004.12:c1 --

NC_000013.11:c3 AAGGTGCGTGAGAAAAGGAGGTAAGGATGGCAGTGAATAGCCCATTGAAATAGTCCAGCC
NC_000001.11:33 --
NC_000023.11:15 -------GTGGAAGAGGAAGATGAAGA---------------------------------
NC_000004.12:c1 --

NC_000013.11:c3 AAAAGATGATGAGGCCTTAAACTGGGGTAGTAGCAGTGGGCATCATGAATTAGGGATGAC
NC_000001.11:33 --
NC_000023.11:15 --
NC_000004.12:c1 --

NC_000013.11:c3 CCAATTGTCCGTCGATGGATAAATGGAGAAACAAAATGTAGTCTCTGCATACCATGGAAT
NC_000001.11:33 --
NC_000023.11:15 --
NC_000004.12:c1 --

NC_000013.11:c3 ATGATTCCATCTTAAAGGTGAAGGAAATGCTCACACAGACTACAACATGGATGAACATTG
NC_000001.11:33 --
NC_000023.11:15 --
NC_000004.12:c1 --

NC_000013.11:c3 AGGACATTGTGTTAAGTAAAATAAACCAATCACAGAAGTACAAATACTGCATGATTTCTC
NC_000001.11:33 --
NC_000023.11:15 --
NC_000004.12:c1 --

NC_000013.11:c3 TTATATGCGGTGGAATATATACTCTTACATACTCTACCACCTAGAGTAGTCAAATTCATA
NC_000001.11:33 --
NC_000023.11:15 --
NC_000004.12:c1 --

NC_000013.11:c3 GAGACAGAAAGTAGAATGTTAGTTGGCACAGGGGTTGGGGGAGGGAAAGGGGAGTGGGAG
NC_000001.11:33 --
NC_000023.11:15 -----------------------------AGAGGAGGAGGAAGAAGAGGAGGAGGAGGAG
NC_000004.12:c1 -------------------TAGTT------------------------------------

NC_000013.11:c3 TTAGTATTTAATGGATACAGAGTTTTAATTGGGGAAGATGAAAAAGTTCTGGAGATGGAT
NC_000001.11:33 --
NC_000023.11:15 ------------------------------GAGGAGGATGAATAAA--------------

```
NC_000004.12:c1 ------------------------------------------------------------


NC_000013.11:c3 GGTACTATTGGTTGCACAATCATGTGAATTTACTTAATGCAATTGAACTGTACACTTAAA
NC_000001.11:33 ------------------------------------------------------------
NC_000023.11:15 -----------------------------------GAAACTGTTTATCTGTCTCCTTGTG
NC_000004.12:c1 ------------------------------------------------------------


NC_000013.11:c3 AGTGGTTAAGGCCGGGAGCAGTAGCTCACGCCTGTAATTCCAGCACTTTGGAAGGCCAAG
NC_000001.11:33 ------------------------------------------------------------
NC_000023.11:15 AAT---------------------------------------------------------
NC_000004.12:c1 ------------------------------------------------------------


NC_000013.11:c3 GCGGGCGGATCACCTGAGGTTGGGAGTTCGAGACCAGCCTGACCAACATGAAGAAACCCT
NC_000001.11:33 ------------------------------------------------------------
NC_000023.11:15 ------------------------------------------------------------
NC_000004.12:c1 ------------------------------------------------------------


NC_000013.11:c3 GTCTCTACTAAAAATACAAAATTAGCCAGGCTTGGTGGCGCACGCCTGTAATCCCAGCTA
NC_000001.11:33 ------ACCAAAAGCCAGAAGCCGGCC---------------------------------
NC_000023.11:15 ------ACTTAGAGTAGGGGAGCGCCG---------------------------------
NC_000004.12:c1 ------ACTTGTAGT---------------------------------------------
                      **. . *..

NC_000013.11:c3 CACCGGAGGCTGAGGCAGGAGAATCGCTTGAACCTAGGAGGCGGAGGTTGCAGTGAGCCG
NC_000001.11:33 ------------------------------------------------------------
NC_000023.11:15 ------------------------------------------------------------
NC_000004.12:c1 ------------------------------------------------------------


NC_000013.11:c3 AGATCACACCATTGCACTCCAGCCTGGGCAACAAGAGCGAAACTCCATCTCAAAAAAAAA
NC_000001.11:33 ------------------------------------------------------------
NC_000023.11:15 ------------------------------------------------------------
NC_000004.12:c1 ------------------------------------------------------------


NC_000013.11:c3 AAGTGGTTAAAATGGTAACTTTTATGTTATGTATATTTTACCACAGTAAAAAACACTTCT
NC_000001.11:33 ------------------------------------------------------------
NC_000023.11:15 ------------------------------------------------------------
NC_000004.12:c1 ------------------------------------------------------------


NC_000013.11:c3 AAATTTAAAACAAACAAGGTTAGAGATAAATTTGAGAAAGAGTTTAAAGATACAAATGAA
NC_000001.11:33 ------------------------------------------------------------
NC_000023.11:15 ------------------------------------------------------------
NC_000004.12:c1 ------------------------------------------------------------


NC_000013.11:c3 AGTAGAGTAAAGGTGATTGCCAAGCTATCTAGCTTAGCTGATTAGGTAGATGATGAGGCC
NC_000001.11:33 ------------------------------------------------------------
NC_000023.11:15 ------------------------------------------------------------
NC_000004.12:c1 ------------------------------------------------------------


NC_000013.11:c3 ATTAGCCAAGAAAGAGAACATTAACCAAGAGGACAAACAGATGATTAGGAGCTCAGTTGG
NC_000001.11:33 ------------------------------------------------------------
NC_000023.11:15 ------------------------------------------------------------
NC_000004.12:c1 ------------------------------------------------------------


NC_000013.11:c3 AGAAATGTGAAGTTTGAGTGGCCTGCCAAACACTCAATGGGAGATTTTTAAAATAACAAC
```

```
NC_000001.11:33 ----------AGTTTGAGTG----------------------------------------
NC_000023.11:15 ------------------------------------------------------------
NC_000004.12:c1 ------------------------------------------------------------


NC_000013.11:c3 TTTACTGAGTTGTATACAATAAATTGCACCTATTTAAAGTGTGCCATTGAAAGAGTTTTT
NC_000001.11:33 ------------------------------------------------------------
NC_000023.11:15 ------------------------------------------------------------
NC_000004.12:c1 ------------------------------------------------------------


NC_000013.11:c3 TTTTTTAATAAAGGCTAGCATTTATTAGATGCTTATTACAGCCATTTTCCCAATTTTTTT
NC_000001.11:33 -----------------------------TAGCTGTTACAGC------------TTTGTG
NC_000023.11:15 -----------------------------TAATTGACACATCTCTT--------------
NC_000004.12:c1 ----------------------TATTGTATAGTCTGTATAGTCTGTT-----ATTTTTTT
                                             *. ..  .*.* .

NC_000013.11:c3 TTTTAATGACATTTCACTACGTTGCCCAATCTGGTCTTGAAATCCTGGGCTCAGCAACCC
NC_000001.11:33 TTCTAA------------------------------------------------------
NC_000023.11:15 ------------------------------------------------------------
NC_000004.12:c1 TTTTAA------------------------------------------------------


NC_000013.11:c3 TCCCCCACCTCAGCCTCCTGAGCAGCTGGGATTACAGGTGCACACAACCACACCTGGCTC
NC_000001.11:33 ------------------------------------------------------------
NC_000023.11:15 ------------------------------------------------------------
NC_000004.12:c1 ------------------------------------------------------------


NC_000013.11:c3 AATTCAAAGAGTTTTTGTTTTGTTCTATGTAAGAGATGAGTGTCTTGCTTTGATGCCCAG
NC_000001.11:33 --------------------------ACCTAAGAAATGGGGACC----------------
NC_000023.11:15 --------------------------ATTTGAGAAGTGTCTGTT----------------
NC_000004.12:c1 ---GCAGAGA----------------GTCAAAGAAATTGTTTTA----------------
                                          ..  .***..*     .

NC_000013.11:c3 GCTGGAGTGCTGTGGTGTGATCATAGCTCAATGCAGCCTCAAACTCCTGGGCTCAAGAGA
NC_000001.11:33 ------------------------------------------------------------
NC_000023.11:15 ------------------------------------------------------------
NC_000004.12:c1 ------------------------------------------------------------


NC_000013.11:c3 TCCTCCCATTCAGCCTCACAAGTAGCTGGGACCACAGGCGTGTGCGCCTCGCCTGGCTAA
NC_000001.11:33 -CCTCACATGAGAG----------------------------------------------
NC_000023.11:15 -GCCCTCATTAGGTTTAATTACAAAATTTGATCACGATCATAT-----------------
NC_000004.12:c1 ------------------------------------------------------------


NC_000013.11:c3 TTTTTTCATTTTTATTTTTGTAGAGATGAGGTCTTGCTATCTTGCCCAGGCTGGTCTCAA
NC_000001.11:33 ------------------------------------------------------------
NC_000023.11:15 ------------------------------------------------------------
NC_000004.12:c1 ------------------------------------------------------------


NC_000013.11:c3 ACTCCTGGCCTCAAGTGATCCTCCCGCTTTGGCCTCCAAAGTGCTAGGATTACAGGCGTG
NC_000001.11:33 ------------------------------------------------------------
NC_000023.11:15 ------------------------------------------------------------
NC_000004.12:c1 ------------------------------------------------------------


NC_000013.11:c3 AGCCACTGCACACAGCCAGTTCAATGCACACATCTGTGAACCCACCACAACCAAGGTTCA
NC_000001.11:33 ------------------------------------------------------------
NC_000023.11:15 ------------------------------------------------------------
NC_000004.12:c1 ------------------------------------------------------------
```

```
NC_000013.11:c3 GAACATTCCTGTCATCCCCCAGATTTTCTCGTTTCCCTTATCCTTCTCCATGTCTGCCCC
NC_000001.11:33 ------------------------------------------------------------
NC_000023.11:15 ------------------------------------------------------------
NC_000004.12:c1 ------------------------------------------------------------


NC_000013.11:c3 GGAAAACAACTGAGCTTTCTGCCACTTACCGATTAGTTTGCATTTTCTATAAATGAAAAC
NC_000001.11:33 ------------------------------------------------------------
NC_000023.11:15 ------------------------------------------------------------
NC_000004.12:c1 ------------------------------------------------------------


NC_000013.11:c3 ATATAATATGTAGTTTTGTGAGGAGGTCTGACTTATTTCACTAAGCATAAAGATTTTGAG
NC_000001.11:33 --------TGGGG-----------------------------------------------
NC_000023.11:15 --------TGTAGTCTCTCAAAGTGCTCTAG-----------------------------
NC_000004.12:c1 --------TGTAG-----------------------------------------------
                        ** .*

NC_000013.11:c3 AGCCATCCATGTTGTGAGCATTAGTAGTTCATTCCTGTTTATTACTGACTCATGTTGCAC
NC_000001.11:33 ------------------------------------------------------------
NC_000023.11:15 ------------------------------------------------------------
NC_000004.12:c1 ------------------------------------------------------------


NC_000013.11:c3 CTTTTGACTATGCTCCAATTTGTTTATGGCATAAACAAATTGGTTTATGCCAGTTGTTCA
NC_000001.11:33 ------------------------------------------------------------
NC_000023.11:15 ----------------AAATTGTCAGTGGTTTACATGAAGTG------------------
NC_000004.12:c1 ----------------------------ATATATATAAAATG------------------


NC_000013.11:c3 ACATTTGGTTTGCTTCTAGTTTTTAACTATTACAAATAAACCTACAATGAACATTGCTGT
NC_000001.11:33 ------------------------------------------------------------
NC_000023.11:15 ------------------------------------------------------------
NC_000004.12:c1 ------------------------------------------------------------


NC_000013.11:c3 ACAAGTCTTTGTGTGAACATATATTTTTATTTTTCCTGGCTGAGTACCCAGAGGCAGAAC
NC_000001.11:33 ------------------------------------------------------------
NC_000023.11:15 ------------------------------------------------------------
NC_000004.12:c1 ------------------------------------------------------------


NC_000013.11:c3 GACGAGGTTGAATGGCAGGTGTATGTTTAACTTTTTTTTTTTTTTTTTTTTTTTTGAGAT
NC_000001.11:33 ------------------------------------------------------------
NC_000023.11:15 ------------------------------------------------------------
NC_000004.12:c1 ------------------------------------------------------------


NC_000013.11:c3 GTAGTCTCACTTCGTCACCTGGCCAGGCTGGAGTGCAGTGGCAAGACCTCGGCTCACTGC
NC_000001.11:33 ------------------------------------------------------------
NC_000023.11:15 ------------------------------------------------------------
NC_000004.12:c1 ------------------------------------------------------------


NC_000013.11:c3 AACCTCCCCCTCCCAGGTTCAAGCAATTCTCCTGCCTCAGGCTCCTGAGTAGCTGGGACT
NC_000001.11:33 --------------------------------------AGGCTTCTGG------------
NC_000023.11:15 ---------------------------------GCCATGGGTGTCTGG------------
NC_000004.12:c1 ---------------------------------------------TGA------------
                                                             **.

NC_000013.11:c3 ACAGGCCCACGCTACCACGCCCAGCTAATTTTTGTATTTTTAGTAGAGACAGGGATTCAC
NC_000001.11:33 ------------------------------------------------------------
NC_000023.11:15 ------------------------------------------------------------
```

```
NC_000004.12:c1 ------------------------------------------------------------


NC_000013.11:c3 CATGTTGGCCTGGCTGGTGTCGAACTCCTGACCTCAGGTGATCCGCCTGCCTCAGCCTCC
NC_000001.11:33 ------------------------------------------------------------
NC_000023.11:15 ------------------------------------------------------------
NC_000004.12:c1 ------------------------------------------------------------


NC_000013.11:c3 CAAAGTGCTAGGATTATAGGCATGAGCCAGCGTGCCCGGCCATGTTTAACTTTTAAAGAA
NC_000001.11:33 ------------------------------------------------------------
NC_000023.11:15 ------------------------------------------------------------
NC_000004.12:c1 ------------------------------------------------------------


NC_000013.11:c3 GCTATCAGTTTTCCAAAGTGCTTGTACCATTTTACATTCCTATCAGCAGTTGTACATCTT
NC_000001.11:33 ------------------------------------------------------------
NC_000023.11:15 ------------------------------------------------------------
NC_000004.12:c1 ------------------------------------------------------------


NC_000013.11:c3 CACCAATACTTGGTATTGTCATCATTTTATCTTGAGCCATTCTCATTGGTGTGTAGTGGC
NC_000001.11:33 ------------------------------------------------------------
NC_000023.11:15 ------------------------------------------------------------
NC_000004.12:c1 ------------------------------------------------------------


NC_000013.11:c3 ATCTCACTGTGGTTTTAATTTGTGTTTTCCAGATAACTAACAATATTGAGCATCTTTTCA
NC_000001.11:33 ------------------------------------------------------------
NC_000023.11:15 ------------------------------------------------AGCACCCT----
NC_000004.12:c1 ------------------------------------------------------------


NC_000013.11:c3 TGTAACTTCTTTTTATAAAGCATTTGTTTGTCTTTTGCCTACTGAAAAAATTGCATTGCT
NC_000001.11:33 ------------------------------------------------------------
NC_000023.11:15 ----------------------------------------------GAAACTGTATCA--
NC_000004.12:c1 ------------------------------------------------------------


NC_000013.11:c3 TTCTTACTGAGTTCTAATAGCCCTTTATACATTCTAGATACCAGTCCATTATATGTATTG
NC_000001.11:33 ----------------------------------------------------ATGTGCTA
NC_000023.11:15 ----------------------------------------------------AAGTTGTA
NC_000004.12:c1 ----------------------------------------------------AGGTACAG
                                                                    * **   .

NC_000013.11:c3 CAAATATTTTTTCATTCTATGCTTTATCTTTTTTCATTTTTTTTTAATGTATGAATAATT
NC_000001.11:33 CAAATTCCATTTCATTTT------------------------------------------
NC_000023.11:15 CATATTTCCAAACATTTT------------------------------------------
NC_000004.12:c1 GAGGGACTATGGCACTGTG-----------------------------------------
                 * .  ..    **.* *

NC_000013.11:c3 TTTTTTTGAGATGAGGCTGACTGTGTTGCCCAGGCTGTTCTCAAACTCCTGAGCTCAAGC
NC_000001.11:33 ------------------------------------------------------------
NC_000023.11:15 ------TAAAATGAA---------------------------------------------
NC_000004.12:c1 ------TGTGATGTA---------------------------------------------


NC_000013.11:c3 AACCCTCCCACCTCGGCCTCCCAAACTGCTGGAATTACAGGCATGAACCACTGTGCCTAG
NC_000001.11:33 -----------------------------------------CATGAACTCCAGTG-----
NC_000023.11:15 -----------------------------------------AAGGCACTCTCGTG-----
NC_000004.12:c1 -----------------------------------------AAAGGGTATTGGTA-----
                                                          * * ..  . **.

NC_000013.11:c3 CCTTTTCATTTACTTAACATATCTTTCTTTCTTTTTTTTTTTTTAGAGGGAGTCTTGCTC
```

```
NC_000001.11:33 ---------TTAATTAATA-----------------------------------------
NC_000023.11:15 ---TTCTCCTCACTCTGTGCACTTTGC---------------------------------
NC_000004.12:c1 ------------------------------------------------------------


NC_000013.11:c3 ATCGCCCAGGCTGGAGTGCAGTGGTGCAATCTTGGCTCACTGCAACCTCCGCCTCCTGGC
NC_000001.11:33 ------------------------------------------------------------
NC_000023.11:15 ------------------------------------------------------------
NC_000004.12:c1 ------------------------------------------------------------


NC_000013.11:c3 TTCAAGCGATTCTCCTGCCTCAGCCTCCCGAGTAGCTGGGACTACAGGTGCATACTACCA
NC_000001.11:33 ------------------------------------------------------------
NC_000023.11:15 ------------------------------------------------------------
NC_000004.12:c1 ------------------------------------------------------------


NC_000013.11:c3 CGCCTGGCTAATTTTTTGTATGTTTAGTAGAAATGGGGTTTCACCTTGTTAGCCAGGATG
NC_000001.11:33 ------------------------------------------------------------
NC_000023.11:15 ------------------------------------------------------------
NC_000004.12:c1 ------------------------------------------------------------


NC_000013.11:c3 GTCTTGATCTCCTGACCTCCTGATCTGCCCGCCTCGGCCTCCCAAAGTGCTGGGATTACA
NC_000001.11:33 ------------------------------------------------------------
NC_000023.11:15 -----------------------------------------------TGTTGGTGTGACA
NC_000004.12:c1 ----------------------------------------GTGAAAGTACTGATACTGCT


NC_000013.11:c3 GGCGTAAGCCACCGCACCTGGCCAATGATATCTTTCTAAGAGCAAATTTTTAAGTGTTGA
NC_000001.11:33 ------------------------------------------------------------
NC_000023.11:15 ------------------------------------------------------------
NC_000004.12:c1 ------------------------------------------------------------


NC_000013.11:c3 CAAGGTTTAGTTTATCAGTTTTCTCTTTTGTGAAAGTTTGCTTTTTTTGTCCTAAGACTT
NC_000001.11:33 ------------------------------------------------------------
NC_000023.11:15 ------------------------------------------------------------
NC_000004.12:c1 ------------------------------------------------------------


NC_000013.11:c3 CTCCTCTGTTTAACTCTTGGAGTTTTCTAGATTGTACCCTTAGGTCTCGCATTCATTTTG
NC_000001.11:33 ------------------------------------------------------------
NC_000023.11:15 ------------------------------------------------------------
NC_000004.12:c1 ------------------------------------------------------------


NC_000013.11:c3 AGTTGATTTCTGTATGTAGCATGATGTAAAAGTCAAAGTTTATTTCTTTTTTTTTCTTTT
NC_000001.11:33 ---------------------------AAATCTCAAGGTTCTCCCCCCTCTTTCGCCTCA
NC_000023.11:15 ---------------------------AGGCATTTAAAGATGTTTCTGGCATTTTCTTTT
NC_000004.12:c1 -----------------------------GTATCGCAACCCTTGTCATTTTACGTCATTA
                                             .  *.  ..  . .*   .  .  * *.

NC_000013.11:c3 TTTTTTTTTGAGATAGGGTCTTGCTTTGTCACCCAAGCTAGACCAGTGGCATCATCATGG
NC_000001.11:33 CATTTCT-----------------------------------------------------
NC_000023.11:15 TATTTGT-----------------------------------------------------
NC_000004.12:c1 ACTTGTT-----------------------------------------------------
                  **  *

NC_000013.11:c3 CTCGCTGCAACCTCTGCCTCCCAGGCTTAGGTGATCCTCCCACCTCAGCCTCCTGAATAG
NC_000001.11:33 ------------------------------------------------------------
NC_000023.11:15 ------------------------------------------------------------
NC_000004.12:c1 ------------------------------------------------------------
```

```
NC_000013.11:c3 CTGGAATTACAGGCGTGCACCACCACACACAGCTAATTTTTATATTTTTTGTAGAAATGG
NC_000001.11:33 ------------------------------------------------------------
NC_000023.11:15 ------------------------------------------------------------
NC_000004.12:c1 ------------------------------------------------------------

NC_000013.11:c3 GGTTTTGCCATGTTGCCCAGGCTGGCTATCCGCCTGCCTCAGCCTCCTGAAGTGTTGGAA
NC_000001.11:33 ------------------------------------------------------------
NC_000023.11:15 ------------------------------------------------------------
NC_000004.12:c1 ------------------------------------------------------------

NC_000013.11:c3 TTACAGGCATGAGCCACCGTGCCCAGCCTCAAGGTTTGTTTCTTTTTCCATATGACTACC
NC_000001.11:33 ------------------------------------------------------------
NC_000023.11:15 ------------------------------------------------------------
NC_000004.12:c1 ------------------------------------------------------------

NC_000013.11:c3 CAGTTGTTTTAGTAAAATTTGTTGAAAAGTCTTTCCTATGGGCCAGGCGCGGTGGCTCAC
NC_000001.11:33 ------------------------------------------------------------
NC_000023.11:15 ------------------------------------------------------------
NC_000004.12:c1 ------------------------------------------------------------

NC_000013.11:c3 GCCTGTAATCTCAGCACTTTGGGAGGCCAAGGCAGGCAGATCATCTAAGGTCAGGAGTTC
NC_000001.11:33 ------------------------------------------------------------
NC_000023.11:15 ------------------------------------------------------------
NC_000004.12:c1 ------------------------------------------------------------

NC_000013.11:c3 AAGACCAGCCTGGCTAACATGGTGAAACCCCGTTTCTACTAAAAATACAAAAAATTAGCC
NC_000001.11:33 ------------------------------------------------------------
NC_000023.11:15 ------------------------------------------------------------
NC_000004.12:c1 ------------------------------------------------------------

NC_000013.11:c3 AGGCGTGGTGCCACACGCCTGTAATCCCAGCTACTCGGGAGGCTGAGGCAGGAGAATCGC
NC_000001.11:33 ------------------------------------------------------------
NC_000023.11:15 ------------------------------------------------------------
NC_000004.12:c1 ------------------------------------------------------------

NC_000013.11:c3 TTGAATCTGGGAGGCAGAGGTTGCAGTGAGCCAAGACTGCGCCTTTGCATTCTGGCTTGG
NC_000001.11:33 ------------------------------------------------------------
NC_000023.11:15 ------------------------------------------------------------
NC_000004.12:c1 ------------------------------------------------------------

NC_000013.11:c3 GCAACAAGAGCGAAACTCCGTCTCAAAAAAAAAAAAAGTATTTTTGTGCTGAATGTCTTA
NC_000001.11:33 ----------------------TCAGAGAAATGAAGAGT---------------------
NC_000023.11:15 ----------------------------------AAGGT---------------------
NC_000004.12:c1 ----------------------------------AAAGC---------------------
                                                  *..*.

NC_000013.11:c3 CCAACTTTGTTGAAAATGAATTGAACATATATGTCTGGGACTATTTCTGGTGTCTATTCA
NC_000001.11:33 ------------------------------------------------------------
NC_000023.11:15 ------------------------------------------------------------
NC_000004.12:c1 ------------------------------------------------------------

NC_000013.11:c3 CTTGCAGGTGAAGATATCTAGTGGAATGCGTGACAGAGGGAATTAGAGCTACTTTTTTGC
NC_000001.11:33 ------------------------------------------------------------
NC_000023.11:15 -------------------GGTGG------------------------------------
```

```
NC_000004.12:c1 -----------------CTAGTGG------------------------------------

NC_000013.11:c3 TGAATACCAGCAGTGTACCTTTGCCTACACACAGAGAAAAGCACGTCTTCAGGAAAGCCT
NC_000001.11:33 ------------------------------------------------------------
NC_000023.11:15 ------------------------------------------------------------
NC_000004.12:c1 ------------------------------------------------------------

NC_000013.11:c3 ACCTACCAACATCAAGTTTGCATATCATTTCCTGATTTTTTTTTTAAGAGACAGGGTCTT
NC_000001.11:33 ------------------------------------------------------------
NC_000023.11:15 ------------------------------------------------------------
NC_000004.12:c1 ------------------------------------------------------------

NC_000013.11:c3 ATTCTGTCGCCCAAGCTGGAATGTAGTGGTGTGATCATAGCTCATTGCAGCCTCAAATTC
NC_000001.11:33 ------------------------------------------------------------
NC_000023.11:15 ------------------------------------------------------------
NC_000004.12:c1 ------------------------------------------------------------

NC_000013.11:c3 TTGGGCTTAAGCGATCCTCCCACCACAGCCTCCCAAATTCCTAAATTCTTTTTTATTCGC
NC_000001.11:33 ------------------------------------------------------------
NC_000023.11:15 ------------------------------------------------------------
NC_000004.12:c1 ------------------------------------------------------------

NC_000013.11:c3 CGAATTCCTAATTTTTTTTTTTTTTTTTCTGTTCAGACAGTCTCACTCTGTTGCCCAGGC
NC_000001.11:33 ------------------------------------------------------------
NC_000023.11:15 ------------------------------------------------------------
NC_000004.12:c1 ------------------------------------------------------------

NC_000013.11:c3 TGGAGTGCAGTGGCAAAATGTCAGCTCACTGCAACCTCTGCCTCCCAGGCTCAAGTGATT
NC_000001.11:33 ------------------------------------------------------------
NC_000023.11:15 ------------------------------------------------------------
NC_000004.12:c1 ------------------------------------------------------------

NC_000013.11:c3 CTTGCGCCTCAGCCTCCCAAGTAGCTGGGATTATAGGCAAGCACTACCACGCCAACTAAC
NC_000001.11:33 ------------------------------------------------------------
NC_000023.11:15 ---------------------TAACTATGGTTATT-------------------------
NC_000004.12:c1 ------------------------------------------------------------

NC_000013.11:c3 TTTTGCATTTCTATTAGAGACAGGGTTTTGCCGTGTTGGCCAGGCTGGTCTCAAACTCCT
NC_000001.11:33 --------------------------------------------------TCCAACTCAT
NC_000023.11:15 ------------------------------------------------------------
NC_000004.12:c1 ------------------------------------------------------------

NC_000013.11:c3 AGCCCCAAGTGATCTGCCCACCTCGGCCTCCCAAAGTGCTGGGATTACAGGCATGAACCA
NC_000001.11:33 ------------------------------------------------------------
NC_000023.11:15 ------------------------------------------------------------
NC_000004.12:c1 ------------------------------------------------------------

NC_000013.11:c3 CCACGCCCAGCCCAAATTCCTAAATTCTTAATTGAAATACTTATCTCATATGACTCTTTG
NC_000001.11:33 --------AGCCCAAGTGCTCAGGTTTTAAATTAGACAACTT------------------
NC_000023.11:15 --------GGCTAGAAATCCTGAGTTTTCAACTGTATATATC------------------
NC_000004.12:c1 --------GATAAGTGCTCTAAAAACTTAGACTGGTTACCTT------------------
                        ...  . .  *. ... ..* .*.*.      *.

NC_000013.11:c3 TAGAGCCAAGACCTACAGTGATAAGGCAAGGGAAAATATTAAAACCTCGGTTCAGACTTG
```

```
NC_000001.11:33 ------------------------------------------------------------
NC_000023.11:15 ---------------------TATAGTTTGTAAAAAGAACAAAAC---------------
NC_000004.12:c1 ------------------------------------------------------------


NC_000013.11:c3 GTGAAGGCATAATGGCAAAAAGTATTTGGGATACCTGATTCTTGACAACACTAAACATAT
NC_000001.11:33 ------------------------------------------------------------
NC_000023.11:15 ----------AACCGAGACAAACCCTTGATGCTCCTTGCTC-------------------
NC_000004.12:c1 ------------------------------------------------------------


NC_000013.11:c3 TTATTATTTCTGTGAAAAAAATTTACAGGTCAAAGAATTGAAAATGCCTGGAAGATAGCT
NC_000001.11:33 ------------------------------------------------------------
NC_000023.11:15 ---------------------------------GGCGTTGAGGCTGTGGGGAAGAT----
NC_000004.12:c1 ------------------------------------------------------------


NC_000013.11:c3 AAAGGATCTGGAGGCTTTTGGGGCAGACAGTCAGGATCTGGACATCAGCAAACTCTCCAG
NC_000001.11:33 ------------------------------------------------------------
NC_000023.11:15 ------------GCCTTTTGGGAGAGGCTGT-----------------------------
NC_000004.12:c1 ------------------------------------------------------------


NC_000013.11:c3 CTTTTTTTTTTTTTTTTTTTTTTGAGACCAGCGCAGTGGCTGATGCCTGTAATCCCAACA
NC_000001.11:33 ------------------------------------------------------------
NC_000023.11:15 ------------------------------------------------------------
NC_000004.12:c1 ------------------------------------------------------------


NC_000013.11:c3 TTCTGGGAGGCCGGGGCAGGCGGATCACTTGGGGTCAGGAGTTCGAGGCCAACACGGTGA
NC_000001.11:33 ------------------------------------------------------------
NC_000023.11:15 ---------------------------------------AGCTCAGGGCGTGCACTGTGA
NC_000004.12:c1 ------------------------------------------------------------


NC_000013.11:c3 AACCCCGTCTCTACTAAAATTACAAAAATTAGCCGGGCATGGTGGTGCATGCCTGTAATC
NC_000001.11:33 ------------------------------------------------------------
NC_000023.11:15 G-----------------------------------------------------------
NC_000004.12:c1 ------------------------------------------------------------


NC_000013.11:c3 CCAGCTACTCAGGAGGCTGAGGCAGGAGAATTGCTGGAACTCAGAAGGCAGAGATTGCAG
NC_000001.11:33 ------------------------------------------------------------
NC_000023.11:15 --------------------------------GCTGGA----------------------
NC_000004.12:c1 ------------------------------------------------------------


NC_000013.11:c3 TGAGCTGAGATCTTGCTACTGCACTCCAGCCTGGGCGACAGAGCGAGACTCCGTCTCAGG
NC_000001.11:33 ------------------------------------------------------------
NC_000023.11:15 ------------------------------------------------------------
NC_000004.12:c1 ------------------------------------------------------------


NC_000013.11:c3 AAAAAAAAAAAAAAAAGAAATTCCATTTGAATTGGTCATTTAAAAGGATAGAAGACTGTG
NC_000001.11:33 ------------------------------------------------------------
NC_000023.11:15 ------------------------------------------------------------
NC_000004.12:c1 ------------------------------------------------------------


NC_000013.11:c3 CTGAAGAACATGCATTCAGAAGTGAGTGACCCAGTGGGCCAAGGAATCTATTCGTATCCT
NC_000001.11:33 ------------------------------------------------------TGTCCT
NC_000023.11:15 ------------------------------------------------------CCTGTT
NC_000004.12:c1 ------------------------------------------------------------
```

```
NC_000013.11:c3 GACTTTGGCACACATATTAACAGTGTTAACCTCTGTTCCTCCTCCTAGTGAGAGCTACAC
NC_000001.11:33 GAC---------------------------------------------------------
NC_000023.11:15 GACTCTG-----------------------------------------------------
NC_000004.12:c1 ------------------------------------------------------------


NC_000013.11:c3 GTGCTATTAGGTCAAATACATAGCTACTGTTTAACTATGCTTATTTAAAATGAGTAAGTT
NC_000001.11:33 ------------------------------------------------------------
NC_000023.11:15 -------CAGG-------------------------------------------------
NC_000004.12:c1 ------------------------------------------------------------


NC_000013.11:c3 AAAAATATGTACCGAACAAATCCTTTTCCACTTACACATGCTGTATTTTTTGTTTTTACA
NC_000001.11:33 ------------------------------------------------------------
NC_000023.11:15 ------------------------------------------------------------
NC_000004.12:c1 ------------------------------------------------------------


NC_000013.11:c3 ACTTTAAATTTCTATCCTCTTCCCACCAGTACAACTGCATCTTCTTGTTAAGAAAATAGT
NC_000001.11:33 ------------------------------------------------------------
NC_000023.11:15 ------------------------------------------------------------
NC_000004.12:c1 ------------------------------------------------------------


NC_000013.11:c3 TAACTTAGGAATTAATTTTATATCCTTCAATAAGAGTTTTTTTGTTTGTTCATGTGTTTG
NC_000001.11:33 ------AGGAACCAAGTC------------------------------------------
NC_000023.11:15 ------GGGCATCCATTTAGCTTC---------AGGTTGTCTTGTTTCTGTATAT-----
NC_000004.12:c1 ---TTTAGACAGTTATTA---------------GGGTTAT--------------------
                      .*. * . * *

NC_000013.11:c3 AGATGGGGGTCTCACTATATTGCCCAGGCTGGTCTCAAATTCCTGGGCTCAAGTGACTGT
NC_000001.11:33 ------------------------------------------------------------
NC_000023.11:15 ------------------------------------------------------------
NC_000004.12:c1 ------------------------------------------------------------


NC_000013.11:c3 CCTGCCTCAACCTACTGAGTAGCTGGGACTACAGGCACGTACTACTGCACCAGGCTTCAA
NC_000001.11:33 ------------------------------------------------------------
NC_000023.11:15 ------------------------------------------------------------
NC_000004.12:c1 ------------------------------------------------------------


NC_000013.11:c3 CAAGAGTTTTTAGAAGTGCCTAGAAATTAAAGTTCTAGGGTTTTGTACTGTTGAGTTAAT
NC_000001.11:33 ------------------------------------------------------------
NC_000023.11:15 ------------------------------------------------------------
NC_000004.12:c1 ------------------------------------------------------------


NC_000013.11:c3 GGTGTTTCTGAAGAAAGAGTGTCAAATCCATCAGTTGCTAAGCCTGCTCCCTTAAAGTCA
NC_000001.11:33 ------------------------------------------------------------
NC_000023.11:15 -----------------AGTGACATAGCATTCTGCTGC----------------------
NC_000004.12:c1 ------------------------------------------------------------


NC_000013.11:c3 CTCAAAAATGTATTTAGACTCCAAGGCAATCAACATAAAGGAAAAATTATTTTTCCCAAC
NC_000001.11:33 ------------------------------------------------------------
NC_000023.11:15 ------------------------------------------------------------
NC_000004.12:c1 ------------------------------------------------------------


NC_000013.11:c3 ATTTGTGTTTCTGAATGCTTGCTGTGCTGAATATTGGCACTGAATGTTCATGTTTAACCT
NC_000001.11:33 ------------------------------------------------------------
NC_000023.11:15 ------------------------------------------------------------
```

```
NC_000004.12:c1 ------------------------------------------------------------

NC_000013.11:c3 TTTGTCTAATTATTTCATTTCAGGTCCTCCTTTTAGGTCATTATTATTATATGTATATAT
NC_000001.11:33 ------------------------------------------------------------
NC_000023.11:15 ------------------------------------------------------------
NC_000004.12:c1 ------------------------------------------------------------

NC_000013.11:c3 GTATGTGTATGCATTTCTGTATGTGTGTATGTATGTTAGGTGTGTGGAGGACAGCATAAG
NC_000001.11:33 ------------------------------------------------------------
NC_000023.11:15 -------------------------------CATCTTAGCTGTG----------------
NC_000004.12:c1 ------------------------------------------------------------

NC_000013.11:c3 CTATCTTTAAGTTTCCCATAATTGAAAAGTTGTATGGGCACAGTGGCTCATACCTGTAAT
NC_000001.11:33 ------------------------------------------------------------
NC_000023.11:15 ------------------------------------------------------------
NC_000004.12:c1 ------------------------------------------------------------

NC_000013.11:c3 CCCAGCACTTTGGGAGGCTGAGGCAGGAGGATCTCTTGAGCCCAGGAGTTCAAAACCAGC
NC_000001.11:33 ------------------------------------------------------------
NC_000023.11:15 ------------------------------------------------------------
NC_000004.12:c1 ------------------------------------------------------------

NC_000013.11:c3 CTGGGCAACATAGTGAGACCCTGTATCTAAAAGTAATAATAATAATAATAATAATAATAA
NC_000001.11:33 ------------------------------------------------------------
NC_000023.11:15 ------------------------------------------------------------
NC_000004.12:c1 ------------------------------------------------------------

NC_000013.11:c3 TAATAATAATAATAATAATAATAAAATTAAAAATTAGCCAGGCTTGTAGCCCCAGTCACT
NC_000001.11:33 ------------------------------------------------------------
NC_000023.11:15 ------------------------------------------------------------
NC_000004.12:c1 ------------------------------------------------------------

NC_000013.11:c3 CAGGAGGCTCAGATGGGAGGATCGCTTGAGCCTGGGAGGGCAAGACTACAGTGAGCTGGG
NC_000001.11:33 ------------------------------------------------------------
NC_000023.11:15 ------------------------------------------------------------
NC_000004.12:c1 ------------------------------------------------------------

NC_000013.11:c3 TTTGGGCCACTGCACTCCAGCCTGGGCAACAGAGAGAGACCCTGTCTCAAACAGAAAAGA
NC_000001.11:33 ------------------------------------------------------------
NC_000023.11:15 ---------------------------GACAAAGGGGGG---------------------
NC_000004.12:c1 ------------------------------------------------------------

NC_000013.11:c3 AAAGAGAAAGTTGATAGCTCCCTGGAATCACATTAAGCCAGTGGATGTCTGTTTACTACT
NC_000001.11:33 ------------------------------------------------------------
NC_000023.11:15 ------------------------------------------------------------
NC_000004.12:c1 ------------------------------------------------------------

NC_000013.11:c3 TTGAATCTTTTGGGTCTAAAATTTTTGTAATTATTGCCATAGCAGCAATTCAGATCCATT
NC_000001.11:33 ------------------------------------------------------------
NC_000023.11:15 ------------------------------------------------------------
NC_000004.12:c1 ------------------------------------------------------------

NC_000013.11:c3 TCTTAGTGATGTTACAACAACTATCATAATATCCAAATTAGAAAATAAAAATGCTGGTAA
```

```
NC_000001.11:33 ----------------ACAGCTGTCATA--------------------------------
NC_000023.11:15 ----------------TCAGCTGGCATG-------------AGAATA-------------
NC_000004.12:c1 ----------------TGGTCAATCATC--------------------------------
                                  . * . ***

NC_000013.11:c3 GAGTTTGTTTTTGTTTTGTTTAGTGATAGTTTAAATTTTTATTGAATTTTTATTATTTGA
NC_000001.11:33 -----------------------------------------------CCTGATTACTT--
NC_000023.11:15 -----------------------------------------------TTTTTTTTTTTTA
NC_000004.12:c1 -----------------------------------------------TTAGATTGTTTAC
                                                               ..   ** .**

NC_000013.11:c3 AGTTTACTGGTCGGCTATAATTAATGGTGTCTGTTTTACACGCTATTTAGTATATGAAGT
NC_000001.11:33 ------------------------------------------------------------
NC_000023.11:15 A-----------------------------------------------------------
NC_000004.12:c1 A-----------------------------------------------------------


NC_000013.11:c3 TTGTATGACCACCTGGTGTATTTGGATGGGTTCAAAGATTACACAACAATTCTGTAATTC
NC_000001.11:33 ------------------------------------------------------------
NC_000023.11:15 ------------------------------------------------------------
NC_000004.12:c1 ------------------------------------------------------------


NC_000013.11:c3 TTTAAAGACTTTGGCAAATGTTTTCTCCCCTCAATTTTAAATCATTGTTTTGTAACGAAA
NC_000001.11:33 ------------------------------------------------------------
NC_000023.11:15 ------------------------------------------------------------
NC_000004.12:c1 ------------------------------------------------------------


NC_000013.11:c3 TTTTAATTAGAAAAAAATTGTTTCTTTTCTAAGTGCAGGCTTCTCCATTGAGCCAATAAA
NC_000001.11:33 ------------------------------------------------------------
NC_000023.11:15 ------------------------------------------------------------
NC_000004.12:c1 ------------------------------------------------------------


NC_000013.11:c3 GAATTTGTTTGAAAATAATTCCCAGCCTCTAGAACTACTAAGAGTATAATTTTTTTCCCT
NC_000001.11:33 ------------------------------------------------------------
NC_000023.11:15 ------------------------------------------------------------
NC_000004.12:c1 ------------------------------------------------------------


NC_000013.11:c3 TTAATTAGTAGCTTAGAATTTTTTTTAAACCAATGCCAAATGCAATGTGAGCTCAAGTTT
NC_000001.11:33 ------------TTAGAGTTACTACTAAAC------------------------------
NC_000023.11:15 ------------GTGCGGTAGTTTTTAAAC------------------------------
NC_000004.12:c1 ------------ACTAAGTGGTTTTTCACA------------------------------
                             .  ..*  .* .* *

NC_000013.11:c3 TTAGTTCAGTTCTAAACTTAGAACCCGTGATTTTCAAGTGGGTTCTAAATTTGGCAAAAT
NC_000001.11:33 ------------------------------------------------------------
NC_000023.11:15 ------------------------------------------------------------
NC_000004.12:c1 ------------------------------------------------------------


NC_000013.11:c3 GTCTTCTCCCTGCCATAGTAGCTGGTGTTTTCCCATTCTTAAGGGTTCATATGTCTTATT
NC_000001.11:33 ------------------------------------------------------------
NC_000023.11:15 ------------------------------------------------------------
NC_000004.12:c1 ------------------------------------------------------------


NC_000013.11:c3 TTCTAAGCAAGCAATACTGTTTTCCTTCACTATTTAATAATTTTTCCATTTCCTCTTTGT
NC_000001.11:33 ------------------------------------------------------------
NC_000023.11:15 ------------------------------------------------------------
NC_000004.12:c1 ------------------------------------------------------------
```

```
NC_000013.11:c3 GGAATACCAAATGTGGTGCAACCTTTTTTAGCCCGTAAAATAAAATAGATACGGCAGCTT
NC_000001.11:33 ------------------------------------------------------------
NC_000023.11:15 ------------------------------------------------------------
NC_000004.12:c1 ------------------------------------------------------------

NC_000013.11:c3 TTTGAAAGAAAAAGGGGAGGAAATGGTAAGGGGGAAAAAAGTCATTTAGCTTGCTTCCCT
NC_000001.11:33 ------------------------------------------------------------
NC_000023.11:15 ------------------------------------------------------------
NC_000004.12:c1 ------------------------------------------------------------

NC_000013.11:c3 CTTACAAGGAAAAGACATACTGTATGTCTTGGATATTGAATTACACAAAGTTTATATATA
NC_000001.11:33 ------------------------------------------------------------
NC_000023.11:15 ------------------------------------------------------------
NC_000004.12:c1 ------------------------------------------------------------

NC_000013.11:c3 GGTTGGTAGAAATCATCAAGTCAGAATTCAGTTCAGGTGCTTATTTTTTCTGCAACCCTC
NC_000001.11:33 ------------------------------------------------------------
NC_000023.11:15 ------------------------------------------------------------
NC_000004.12:c1 ------------------------------------------------------------

NC_000013.11:c3 TGCATAAATGCAAAAGAGTCCCAGAACCTTCCTGAGGAAAGGATACGTGAAACAGCATCC
NC_000001.11:33 ------------------------------------------------------------
NC_000023.11:15 ------------------------------------------------------------
NC_000004.12:c1 ------------------------------------------------------------

NC_000013.11:c3 CATTCGATCACCACATAAAGAAGCCTGTTCAACAAAGCCATCCTTCCTAGTCTGCTCTTT
NC_000001.11:33 ------------------------------------------------------------
NC_000023.11:15 ------------------------------------------------------------
NC_000004.12:c1 ------------------------------------------------------------

NC_000013.11:c3 GCCAAGTATTTCATGGCGCTCCTTGCAGATGAAGGCACCAAGTGTTGAAAGCAGAACTCA
NC_000001.11:33 ------------------------------------------------------------
NC_000023.11:15 ------------------------------------------------------------
NC_000004.12:c1 ------------------------------------------------------------

NC_000013.11:c3 CTAGGTGCAGTGCTCAGGCCTGGCAAGCTAGGGGCACTGGCCTCTGAAGAGTATAGGTGG
NC_000001.11:33 ----------------------------------------CCTTTGTATGGCATGAGCTG
NC_000023.11:15 ----------------------------------TGTTTGTTTTTAAACAA---------
NC_000004.12:c1 ------------------------------------------------------------

NC_000013.11:c3 TTACTGTGTAGATGTTGTAGGTAGTTGGAAAAAGCCACTTCCATCCACAAAGTGTTGGAG
NC_000001.11:33 CCACTGTTCAA-------------------------------------------------
NC_000023.11:15 --ACTATAGAA-------------------------------------------------
NC_000004.12:c1 ------------------------------------------------------------

NC_000013.11:c3 AAAAAGACACCAGGATCTCTCCCCAAAACACAAGACCACATTCACTGCATTCTCACACTC
NC_000001.11:33 ------------------------------------------------------------
NC_000023.11:15 ------------------------------------------------------------
NC_000004.12:c1 ------------------------------------------------------------

NC_000013.11:c3 ACCTGCATTGATCTCTGCTGTTCAGCTCCCTGGCTCAGGTCTCTCCCTATCATGTCCCTT
NC_000001.11:33 ------------------------------------------------------------
NC_000023.11:15 ------------------------------------------------------------
```

```
NC_000004.12:c1 ------------------------------------------------------------


NC_000013.11:c3 GTATCCTTTTTTCTCCTGTTACAATTCTTTCATAAAAATAAAATGACCCAGAGGAAATAA
NC_000001.11:33 ------------------------------------------------------------
NC_000023.11:15 ------------------------------------------------------------
NC_000004.12:c1 ------------------------------------------------------------


NC_000013.11:c3 GACCGGCATGAAGAAAAAGGTGAAATGAGGTTTTTATTCCCCTCCCGCACCCAACATTTG
NC_000001.11:33 ------------------------------------------------------------
NC_000023.11:15 ------------------------------------------------------------
NC_000004.12:c1 ------------------------------------------------------------


NC_000013.11:c3 GAATGCGGTGCAGTCCCTCGGGCAGCACTCCTCTGCAGCAGACTGTATCTGTTGGAGCAG
NC_000001.11:33 ------------------------------------------------------------
NC_000023.11:15 ------------------------------------------------------------
NC_000004.12:c1 ------------------------------------------------------------


NC_000013.11:c3 CTTCCCTTTCTTCCCATGATCATGTTGTTTAGGCATTTGTAAATGTCACCCCAACCGAGA
NC_000001.11:33 ------------------------------------------------------------
NC_000023.11:15 ---------CTCTTCATTGTCA---------------------------------------
NC_000004.12:c1 ------------------------------------------------------------


NC_000013.11:c3 GCAACAAGGCTCAAGCTTGCTCTTTCTCAGAGATAGAGCATTTCCCAGCACTTCCCTGGA
NC_000001.11:33 ------------------------------------------------------------
NC_000023.11:15 ------------------------------------------------------------
NC_000004.12:c1 ------------------------------------------------------------


NC_000013.11:c3 GGTCTTGGCAAATAGGAATCCCGTTCACTCTAGTCTCTCTGGTCCTACCCTCTTCTCTGC
NC_000001.11:33 ------GGCAAACAGAAACACTTCACATTTTA----------------------------
NC_000023.11:15 ------GCAAAGCAAAGAGTCACTGCAT--------------------------------
NC_000004.12:c1 ------GTTGAGTAATGATACCGGATGCTTTATTTTTT----------------------
                      *  .*..*. .*  *    ...

NC_000013.11:c3 TGGGGAGGAAGAACCATCCAGCTCCTTCTAGCAGCAGGCAGCCTCCAGCAAGAGTGGCCT
NC_000001.11:33 ---------------------------------------------------------TCA
NC_000023.11:15 ---------------------------------------------------------CAA
NC_000004.12:c1 ---------------------------------------------------------TGA
                                                                         .

NC_000013.11:c3 TGACAGTCCTGTCCAACAGGTTCCCCTGCTTTATTTTTCTCTTGCTTCCCTGGTGCCAAG
NC_000001.11:33 TGACAGCCCTG-------------------------------------------------
NC_000023.11:15 TGAAAGTTCAA-------------------------------------------------
NC_000004.12:c1 CAATATTTCAG-------------------------------------------------
                ..* * ..* .

NC_000013.11:c3 AGGCGAGGAATACTTAAGATATTTTCCACTCCCAGCCTTCCTGGATACTAAGCATTCAGA
NC_000001.11:33 ------------------------------------------------------------
NC_000023.11:15 ------------------------------------------------------------
NC_000004.12:c1 ------------------------------------------------------------


NC_000013.11:c3 CTACCAGATTGTGGGCTGTGGCAGCAGTAGATTTTCCAGGATGGACTGCCCTCCCGTCAT
NC_000001.11:33 ------------------------------------------------------------
NC_000023.11:15 ------------------------------------------------------------
NC_000004.12:c1 ------------------------------------------------------------


NC_000013.11:c3 CCATACCACTGCCATTGGCAGCTGTCTCCTCTCCCCCAGAGCCGGCAAATGTTGGTAGAA
```

```
NC_000001.11:33 ------------------------------------------------------------
NC_000023.11:15 ------------------------------------------------------------
NC_000004.12:c1 ------------------------------------------------------------


NC_000013.11:c3 TTCCATTGACCTCAGCATTCCTAGAGTGGCTAGAATTGACTTGAACATCCAACCTTTAAG
NC_000001.11:33 ------------------------------------------------------------
NC_000023.11:15 ------------------------------------------------------------
NC_000004.12:c1 ------------------------------------------------------------


NC_000013.11:c3 GGAATGCAGCAAAGTGAACCAGCCAGTGCACTGCCCATGTGGGACCATATGGGTGATTCG
NC_000001.11:33 ------------------------------------------------------------
NC_000023.11:15 ------------------------------------------------------------
NC_000004.12:c1 ------------------------------------------------------------


NC_000013.11:c3 AGCTTGAGGGAGTAAGAAATCGAAATGGGTTCAGATCAGCAGACCAGACTATATGCCAGC
NC_000001.11:33 ------------------------------------------------------------
NC_000023.11:15 ------------------------------------------------------------
NC_000004.12:c1 ------------------------------------------------------------


NC_000013.11:c3 CTCTGACCGTGAAGCAAAGGGCCATTCTGTGAGAGAACATTGTTGCTGTACCATGTCAGC
NC_000001.11:33 ------------------------------------------------------------
NC_000023.11:15 ------------------------------------------------------------
NC_000004.12:c1 ------------------------------------------------------------


NC_000013.11:c3 CTCAAACTTTTAGGGTAGTGCCACACAACATTCCTGGTTTTCCTTAACAATACCTTATTT
NC_000001.11:33 ------------------------------------------------------------
NC_000023.11:15 ------------------------------------------------------------
NC_000004.12:c1 ------------------------------------------------------------


NC_000013.11:c3 ATCTGGCCTTGTCTCCAACATCCCTATCTTGGGCCTCTATCATCAGTCATAGTGTTTTCA
NC_000001.11:33 ------------------------------------------------------------
NC_000023.11:15 ------------------------------------------------------------
NC_000004.12:c1 ------------------------------------------------------------


NC_000013.11:c3 TATTAAATAATCTAATAGAAAAAGCATGAATAGAAATCAAGGGTTCGAGCTTCAGCTCTG
NC_000001.11:33 ------------------------------------------------------------
NC_000023.11:15 ------------------------------------------------------------
NC_000004.12:c1 ------------------------------------------------------------


NC_000013.11:c3 CTGTAAGGTGGTGTATGGCCCCGGGCAAGGCACTGACTTTCTCTAGATCCAATTTCTTTC
NC_000001.11:33 ------------------------------------------------------------
NC_000023.11:15 ------------------------------------------------------------
NC_000004.12:c1 ------------------------------------------------------------


NC_000013.11:c3 ATCTAAGACATAGGGATGCTGGATTTGATTATAACTGTGTTCTAAAATTCCATAAATCTT
NC_000001.11:33 ------------------------------------------------------------
NC_000023.11:15 ------------------------------------------------------------
NC_000004.12:c1 ------------------------------------------------------------


NC_000013.11:c3 CCTTAAACTACTACTTACTAGTCTAATGAACAGCAAAGAGTATTTAAAATACTATTGTAA
NC_000001.11:33 ------------------------------------------------------------
NC_000023.11:15 ------------------------------------------------------------
NC_000004.12:c1 ------------------------------------------------------------
```

```
NC_000013.11:c3 GATACTGTAATATTTTCCCCATAAGCGTCCCTAAGTTACTTTGGTCAAACTTTAAGGAAT
NC_000001.11:33 ------------------------------------------------------------
NC_000023.11:15 ------------------------------------------------------------
NC_000004.12:c1 ------------------------------------------------------------

NC_000013.11:c3 TCCACTATTTCTAGTCTTGTAGGTTCACCCTACCTGTGCTTTTCATGACTTATCTTCCTC
NC_000001.11:33 ----------------------------------------------GATCTATC------
NC_000023.11:15 ------------------------------------------------------------
NC_000004.12:c1 ------------------------------------------------------------

NC_000013.11:c3 ATGCTCCCGCTTTTCCTTTGATGTTAGTGATTGCAGATCTGCTGTCTTTTCTTCTCTTTA
NC_000001.11:33 ------------------------------------------------------------
NC_000023.11:15 ------------------------------------------------------------
NC_000004.12:c1 ------------------------------------------------------------

NC_000013.11:c3 TCTACTTTTATTTATAAGTCTCAGCATGCACTTGATTATTTTTGTTGCCCATCTCTAGAG
NC_000001.11:33 ------------------------------------------------------------
NC_000023.11:15 ------------------------------------------------------------
NC_000004.12:c1 ------------------------------------------------------------

NC_000013.11:c3 AGACCCTCGCTCTAAAAAAAGTCATAGGTACTCAAGGCAGGACACTGGTGTTTTCCGTGT
NC_000001.11:33 ------------------------------------------------------------
NC_000023.11:15 ------------------------------------------------------------
NC_000004.12:c1 ------------------------------------------------------------

NC_000013.11:c3 TTTCCGGTGACACCACACAGTTTGGTGGCTTTGTGATGGCTGCGGCATGCTGAGCTGGTG
NC_000001.11:33 ------------------------------------------------------------
NC_000023.11:15 ------------------------------------------------------------
NC_000004.12:c1 ------------------------------------------------------------

NC_000013.11:c3 CCCTTTGGGGGCTGCCCCCAGTGTCTCCCAAGTTGCCTTCCTATGTTATAAGTGGGAGTT
NC_000001.11:33 ---------------------------------CACCTCCCCATGTT-------------
NC_000023.11:15 ---------------------------------GAACCTCCTGTACT-------------
NC_000004.12:c1 ---------------------------------GATATTGCTGCATA-------------
                                                  .  .. *.....

NC_000013.11:c3 AGGGTCTCTCATCTTAAAAGTACATTTGAAATGTTTTCCTGCTGGGCACAGTGGTTCACA
NC_000001.11:33 ------------------------------------------------------------
NC_000023.11:15 ------------------------------------------------------------
NC_000004.12:c1 ------------------------------------------------------------

NC_000013.11:c3 CCTGTAATCCCAGCACTTTGGGAGGCCAATATGGGTGGATTCCTTGAGGTCAGGAGTTTG
NC_000001.11:33 ------------------------------------------------------------
NC_000023.11:15 ------------------------------------------------------------
NC_000004.12:c1 ------------------------------------------------------------

NC_000013.11:c3 AGACCAGCCTGACCAACATGGTGAAACCCTTCTCTACTAAAAATACAAAAATTAACTGGG
NC_000001.11:33 ------------------------------------------------------------
NC_000023.11:15 ------------------------------------------------------------
NC_000004.12:c1 ------------------------------------------------------------

NC_000013.11:c3 CGTGGTGGCTCATACCTGTAGTCCCAGCTACTTGGGAGGCTGAGGCAGGAGAACCACTTG
NC_000001.11:33 ------------------------------------------------------------
NC_000023.11:15 ------------------------------------------------------------
```

```
NC_000004.12:c1 ---------TCGTGCC--------------------------------------------

NC_000013.11:c3 AACCCGGGAGGTGGGGATTGCAGTAAGCCGAGATTGCACTATGGCACTCCAGCCTGGGCA
NC_000001.11:33 ------------------------------------------------------------
NC_000023.11:15 ------------------------------------------------------------
NC_000004.12:c1 ------------------------------------------------------------

NC_000013.11:c3 ACAGAGTGAGACTCTGTCTCAAAAAAAAAAAAAAAGAAAAAGAAAAAAAGAAATGTTTTC
NC_000001.11:33 ------------------------------------------------------------
NC_000023.11:15 ------------------------------------------------------------
NC_000004.12:c1 ------------------------------------------------------------

NC_000013.11:c3 CATGAAATGCTTCACTCTTTGCTATATTAATACTTATCTCCCATTTTTTGCACATTTACC
NC_000001.11:33 ------------------------------------------------------------
NC_000023.11:15 ------------------------------------------------------------
NC_000004.12:c1 ------------------------------------------------------------

NC_000013.11:c3 CAGCTGTGAGAGGTCCTTTGGCTGTTTATCCCCTTGGCTCTTTGTCTCCTGGAAGAAGAG
NC_000001.11:33 ------------------------------------------------------------
NC_000023.11:15 ------------------------------------------------------------
NC_000004.12:c1 ------------------------------------------------------------

NC_000013.11:c3 TAATCTATAGAATAAAAGATTGCACTGTGTATTTCCACTTCCATGTCATCTTCCCTTCTC
NC_000001.11:33 ------------------------------------------------------------
NC_000023.11:15 ------------------------------------------------------------
NC_000004.12:c1 ------------------------------------------------------------

NC_000013.11:c3 TTCTCTTCTCTTCCCTTCTCTTTTCTTCCCTTCTCTTCTTCTCTCCTCTCCTCTCCTCTC
NC_000001.11:33 ------------------------------------------------------------
NC_000023.11:15 ------------------------------------------------------------
NC_000004.12:c1 ------------------------------------------------------------

NC_000013.11:c3 CTCTTTCCTCTTCCTCCTACTCCTCCTCCTTCTTTTCTCTCTCTCTCTTTCTCTCTTTCT
NC_000001.11:33 ------------------------------------------------------------
NC_000023.11:15 ------------------------------------------------------------
NC_000004.12:c1 ------------------------------------------------------------

NC_000013.11:c3 TTTTTTGAGACAGGGTCTCACAATGTTTCCTAGGCTAGTTTTGAACTCCTGGGCTCAAGC
NC_000001.11:33 ------------------------------------------------------------
NC_000023.11:15 ------------------------------------------------------------
NC_000004.12:c1 ------------------------------------------------------------

NC_000013.11:c3 AGTCCTCCTGCCTTGGCCTCCCAAAGTGCTGGGATTACAGGCGTGAACCACTGCGCCTGG
NC_000001.11:33 ------------------------------------------------CATTATGCTCAG
NC_000023.11:15 -------------------------------------TAAACACGATTCGCAACGTTCTG
NC_000004.12:c1 ------------------------------------------------------------

NC_000013.11:c3 CCCATTTTTTCTTAATGGTGAAAACAAAAAACAAAACAATGTAATAAGCTCTTATACCCA
NC_000001.11:33 CCTGTCTTT---------------------------------------------------
NC_000023.11:15 TTATTTTTTTTGTAT---------------------------------------------
NC_000004.12:c1 ------------------------------------------------------------

NC_000013.11:c3 TCCCAGTCCCTGAGACAGAGTAGCGGTTGTTGGGGGATGGAGGGTGAGGAACGTGGTGAA
```

```
NC_000001.11:33 ---------------------------------GGAAGGGATGATGAGGCA---------
NC_000023.11:15 ---------------------------------GTTTAGAATGCTGAAATG---------
NC_000004.12:c1 ---------------------------------AAGGGCAAAAGTGAAGCA---------
                                                 .     .* . ***.. .

NC_000013.11:c3 GATCCTACTTCAGGAATGGCAAAATAGCATCCTGCTTCACTCAGTTTTAGTAGCAGGTAT
NC_000001.11:33 ------------------------------------------------------------
NC_000023.11:15 ------------------------------------------------------------
NC_000004.12:c1 ------------------------------------------------------------


NC_000013.11:c3 AGAGTAGTTGGTAGCAAAGCTGGTAATAGACTCTTATTCCTTCATTACTTTCCCCCACTT
NC_000001.11:33 ------------------------------------------------------------
NC_000023.11:15 ------------------------------------------------------------
NC_000004.12:c1 ------------------------------------------------------------


NC_000013.11:c3 TATCATTTTAGACATTTAATTATGAATTGAGTGGCTCCTGGAGGTTAGATGCTGGGGAAA
NC_000001.11:33 -----------------------------------TCCTGAAAATCGTATAGTGAGGAAG
NC_000023.11:15 -----------------------------------TTTTTGAAGTTAAAT----------
NC_000004.12:c1 ------------------------------------------------------------


NC_000013.11:c3 ATGGGGAAGAAAGCACACCCTGCCCTGCAGGGGCTTACAGAGGTGAAGGTCACAGACGCC
NC_000001.11:33 GC----------------------------------------------------------
NC_000023.11:15 ------------------------------------------------------------
NC_000004.12:c1 ------------------------------------------------------------


NC_000013.11:c3 TGGGTAGTACAGACCCTGGCCCTGGAGACATTGCCAAGGAGGGGCAGCTCAGGCTGGGAT
NC_000001.11:33 ------------------------------------------------------------
NC_000023.11:15 ------------------------------------------------------------
NC_000004.12:c1 ------------------------------------------------------------


NC_000013.11:c3 GGGGGGTACCAAGGGACTGCTTCCCCCACAGCCAAGCTCTGAATGGGATGGTCAGGGGCA
NC_000001.11:33 ------------------------------------------------------------
NC_000023.11:15 ------------------------------------------------------------
NC_000004.12:c1 ------------------------------------------------------------


NC_000013.11:c3 GCTTCTCAGAGCTGAAAGACAAGCATGAGTTGGACAGGTGTAAGGTTATCCTGCTGGAAA
NC_000001.11:33 -----------------------------------------------------------A
NC_000023.11:15 -----------------------------------------------------------A
NC_000004.12:c1 -----------------------------------------------------------G
                                                                           .

NC_000013.11:c3 GACATCAAGGCCTGAGAGAGCATGACCATCCCAGGACCTCCCAGTGGCTCAGGATGGGAG
NC_000001.11:33 GACCCCATGGCCTG----------------------------------------------
NC_000023.11:15 AACAGTATTACATT----------------------------------------------
NC_000004.12:c1 GAAAGAAGGGCCCTGGC-------------------------------------------
                .*    *  .* .

NC_000013.11:c3 CTTCAGCATGGGCAGGACAGAGAAGCAGGGCCTGACCATGAGTGGCCTAGGATGCTCTGC
NC_000001.11:33 ------------------------------------------------------------
NC_000023.11:15 ------------------------------------------------------------
NC_000004.12:c1 ------------------------------------------------------------


NC_000013.11:c3 TAAGAGCTGAACATTCGGCTGGGTGCAGTAGCTCAAACCTATAATCCCAGCACTTTGAGA
NC_000001.11:33 ------------------------------------------------------------
NC_000023.11:15 ------------------------------------------------------------
NC_000004.12:c1 ------------------------------------------------------------
```

```
NC_000013.11:c3 GGCTGAGGCAGGAGGATTGCTTCAGCCCAAGAGTTTGAGACCAGCCTGGGCAACATGGTG
NC_000001.11:33 ----------------TCACTACAGCCTAGGGATTTTAAGCC------------------
NC_000023.11:15 ---------------------------------TTTAAAACT------------------
NC_000004.12:c1 -------------------------------------AGGCC------------------
                                                     *..*.

NC_000013.11:c3 AGACACCATCTCTACAAAAAATTTAAAAATTAGTTGGGTGTGGTGGTGCGTGTCTGTGGC
NC_000001.11:33 ------------------------------------------------------------
NC_000023.11:15 ------------------------------------------------------------
NC_000004.12:c1 ------------------------------------------------------------


NC_000013.11:c3 CCCAACTACTCAGGAGGCTGAGGAGGGAGGATCGCTTGAGTCCAGGAGGCAGAGGCTGCA
NC_000001.11:33 ------------------------------------------------------------
NC_000023.11:15 ------------------------------------------------------------
NC_000004.12:c1 ------------------------------------------------------------


NC_000013.11:c3 GTGAGCCCAGATGATGTCACTGCACTCCAGCCTGGTGACAAAGGGAGACCCTGACTCAAA
NC_000001.11:33 ------------------------------------------------------------
NC_000023.11:15 ------------------------------------------------------------
NC_000004.12:c1 ------------------------------------------------------------


NC_000013.11:c3 AAAAAAAAAAAAGAAATTTGCACGTTCTCTTGTGGGCAACAGGGAGTCATGCAGGAATTA
NC_000001.11:33 ------------------------------------------------------------
NC_000023.11:15 ------------------------------------------------------------
NC_000004.12:c1 ------------------------------------------------------------


NC_000013.11:c3 TTTAAGCAGAGGAACATCACGATCATAATGTCATAATTTACCTTAGAAACGTCACTCGGA
NC_000001.11:33 ------------------------------------------------------------
NC_000023.11:15 ------------------------------------------------------------
NC_000004.12:c1 ------------------------------------------------------------


NC_000013.11:c3 GTTTCCTAACACTAAGGCCCAGGGTTGGAGTAGAGTGAGCAGGAGAAGGTGGCCTCGGAA
NC_000001.11:33 ------------------------------------------------------------
NC_000023.11:15 ------------------------------------------------------------
NC_000004.12:c1 ------------------------------------------------------------


NC_000013.11:c3 ATGAAGGGAAGTGACAGGGATCAGAGACACACATTAAAGAGGTAGGATCCCTAGGACTGG
NC_000001.11:33 ------------------------------------------------------------
NC_000023.11:15 ------------------------------------------------------------
NC_000004.12:c1 ------------------------------------------------------------


NC_000013.11:c3 AGGGTTTGCTGGTTGCGGGGAGTGAGGGAAAGGAGGAGTTAATGATGATACTCAGGTTTC
NC_000001.11:33 ------------------------------------------------------------
NC_000023.11:15 ------------------------------------------------------------
NC_000004.12:c1 ------------------------------------------------------------


NC_000013.11:c3 TGGGCCGGGCAACTGAGTGAGAGGACCCCCAGAGGAAGTTTTAATGATGAGCACAAAGGT
NC_000001.11:33 ------------------------------------------------------------
NC_000023.11:15 ------------------------------------------------------------
NC_000004.12:c1 ------------------------------------------------------------


NC_000013.11:c3 GATTCTTTATGGCCGTGCCAGGCTTCCAAGCCCTGGTGGATGCAGAATGGAGTGGGGTCT
NC_000001.11:33 ------------------------------------------------------------
NC_000023.11:15 ------------------------------------------------------------
```

```
NC_000004.12:c1 ------------------------------------------------------------


NC_000013.11:c3 TGTGTTTGCTTTGCTCACTCACCTGTGGGTGAGGAGTGCTGGGCTGCTACCCCTTGGCTA
NC_000001.11:33 ------------------------------------------------------------
NC_000023.11:15 ------------------------------------------------------------
NC_000004.12:c1 ------------------------------------------------------------


NC_000013.11:c3 TATTCTCTGACCTGTCATCTTAACATTAGGACAATTGCTCCTCAATAATTGGTAAAGGAC
NC_000001.11:33 ------------------------------------------------------------
NC_000023.11:15 -------------------------------------CTTCTCTATTAT-----------
NC_000004.12:c1 ------------------------------------------------------------


NC_000013.11:c3 CAGCCATGGTGGCTCACACTATAATTCCAGCACTTTAGGAGGCCGAGGCAGGAGGATTGC
NC_000001.11:33 ------------------------------------------------------------
NC_000023.11:15 ------------------------------------------------------------
NC_000004.12:c1 ------------------------------------------------------------


NC_000013.11:c3 TTAAGGCCAGAAGTTCGGGACCAGCCTGGGCAACAGGATATAGCTATACCCTGTCTCTAC
NC_000001.11:33 ------------------------------------------------------------
NC_000023.11:15 -------------------------------AACAGTCAATT------------------
NC_000004.12:c1 -------------------------------AACAGG-----------------------


NC_000013.11:c3 GGAAAAAAAAAAAAAAATAGCTGGTGTTGGTGGTGTGCACTTGTAGTCCTAGATACCCAGG
NC_000001.11:33 ------------------------------------------------------------
NC_000023.11:15 ------------------------------------------------------------
NC_000004.12:c1 ------------------------------------------------------------


NC_000013.11:c3 AGGCTGAGGCATGAGGATGGCTTGAGCCCGGGAGGTCAAGGCTACAGTGAGCTATGATCA
NC_000001.11:33 ------------------------------------------------------------
NC_000023.11:15 ------------------------------------------------------------
NC_000004.12:c1 ------------------------------------------------------------


NC_000013.11:c3 CACCACTGTACTCCAGCCTGGGCAACAGAATAAGACTCTGTCTCTATTTAAACAACAACA
NC_000001.11:33 ----------------------------------------------CCTAAAGTGCAGCA
NC_000023.11:15 ----------------------------------------------TCTGACTCACAGCA
NC_000004.12:c1 ----------------------------------------------CTCAAAGAAGAAGA
                                                              ....*   . *. *

NC_000013.11:c3 AAAAAAAAAATTGGTAAAGACCTCACTTTTTATTTTATTTTTTTTATTTCCCGCAAAGAC
NC_000001.11:33 AAA---------------------------------------------------------
NC_000023.11:15 GTGAACAA----------------------------------------------------
NC_000004.12:c1 ACGAA-------------------------------------------------------
                . .

NC_000013.11:c3 CCTTGAAGTCGCTACCAATGTCATTTGCTACCTTTGGAGTGCATCTTTCTGATACTGTTG
NC_000001.11:33 ------------------------------------------------------------
NC_000023.11:15 ------------------------------------------------------------
NC_000004.12:c1 ------------------------------------------------------------


NC_000013.11:c3 GTTTTAATGACAAAGGCCAGAACGGGAAAGAGACAGACATGGAAGGTGTCGAGTCAGACT
NC_000001.11:33 ------------------------------------------------------------
NC_000023.11:15 ------------------------------------------------------------
NC_000004.12:c1 ------------------------------------------------------------


NC_000013.11:c3 TGGCTTCAGAGCCAGAAAGATGGGTTTGGAACACTAGCTGGGTAAGCTTAGGCAACCTTA
```

```
NC_000001.11:33 ------------------------------------------------------------
NC_000023.11:15 ------------------------------------------------------------
NC_000004.12:c1 ------------------------------------------------------------

NC_000013.11:c3 ATTTCCTCACCTGTAAAATAAGGAAAATAACACCCACCCCAAAGGCTTGATGTGATAATT
NC_000001.11:33 ------------------------------------------------------------
NC_000023.11:15 ------------------------------------------------------------
NC_000004.12:c1 ------------------------------------------------------------

NC_000013.11:c3 ATAATGAGGTAAAGTATTTATGATCCTAGCAGATGGTGAATGCCCAGTAAATATTAGTTT
NC_000001.11:33 ------------------------------------------------------------
NC_000023.11:15 ------------------------------------------------------------
NC_000004.12:c1 ------------------------------------------------------------

NC_000013.11:c3 CTGCCCCCTCCTGGCCCCACCATAGACGCTTGCGGCCTGAGCCCCACTGTCTGCTAACTA
NC_000001.11:33 ------------------------------------------CCCACTGCCT--------
NC_000023.11:15 ---------------------------------------ACCCCCACTCCATTGTATTTG
NC_000004.12:c1 ------------------------------------------------------------

NC_000013.11:c3 ATAAAACATGAGCTTGGTCTTTACCATCTTGATCTCCCTGTTGCTTTTGTGTGTAGGTTT
NC_000001.11:33 ------------------------------------------------------------
NC_000023.11:15 GAGA------------------------CTGGCCTCCCTATAAATGTGGT----AGCTTC
NC_000004.12:c1 ------------------------------------------------------------

NC_000013.11:c3 TTGTTTTATTTAATCTTTCGGACAATGAGAGAAGGGAAGAGAAAAGAGAAAGGGAGAGGA
NC_000001.11:33 ------------------------------------------------------------
NC_000023.11:15 TTTTATTACTCA------------------------------------------------
NC_000004.12:c1 ------------------------------------------------------------

NC_000013.11:c3 GGAGGGACACGGAATGATACAAACTCAATTTAGAACTTTCCTAAGTGAAAATGTTGCCTG
NC_000001.11:33 ------------------------------------------------------------
NC_000023.11:15 ------------------------------------------------------------
NC_000004.12:c1 ------------------------------------------------------------

NC_000013.11:c3 AAAGAGTACCTTCAGGTGTCCTGCCTCAGTTCTTTCTCCAAGAGAAATATTAACCTATGT
NC_000001.11:33 ------------------------------------------------------------
NC_000023.11:15 ------------------------------------------------------------
NC_000004.12:c1 ------------------------------------------------------------

NC_000013.11:c3 GACAATAATATGTGTCTACAGTAGCACATTTAAGACTAAGAATACCTCGAGGCAGGCAGA
NC_000001.11:33 ------------------------------------------------------------
NC_000023.11:15 ---------------------------------------------------GTGGCCAGC
NC_000004.12:c1 ------------------------------------------------------------

NC_000013.11:c3 TCACTTGAGATCAGGAGTTCAAGACCAGCCTGGCCAATATGGTGAAACCCCGTTTCTACT
NC_000001.11:33 ------------------------------------------------------------
NC_000023.11:15 TCACTTAGGGCT------------------------------------------------
NC_000004.12:c1 ------------------------------------------------------------

NC_000013.11:c3 AAAAATACAAAAATTAGCTGGGCGTGGTGGCACACACCTGTAGTCCCAGATACTCAGGAG
NC_000001.11:33 -----------------------------------------------------CCAGGAA
NC_000023.11:15 ---------------------------------------------------------GAG
NC_000004.12:c1 -----------------------------------------------------CCAGAAG
                                                                         .*.
```

```
NC_000013.11:c3 GCTGAGGCAGGAGAATCGCTTGAACCTGGGAGGCGGAGGTTGTGGTGAGCCAAGAGGCAC
NC_000001.11:33 GCCA----AGGAGAATTATTT---------------------------------------
NC_000023.11:15 ATGAAG--GAGAGGGCTACTTGAAGCTA--------------------------------
NC_000004.12:c1 ATGAGG--AGGAGGA---------------------------------------------
                .. .    ..***..

NC_000013.11:c3 CACTGCACTGGTGACAGAGCAAGACTCCATTTCAAAAGAAAAAAAAAAGAAAGACTTAAG
NC_000001.11:33 ------------------------------------------------------------
NC_000023.11:15 ------------------------------------------------------------
NC_000004.12:c1 ------------------------------------------------------------


NC_000013.11:c3 AATACCTACATGGAGCTGGCATATCAGGCTAATGAGTAAGACAGAAGCTTCCTGGCCTTC
NC_000001.11:33 ------------------------------------------------------------
NC_000023.11:15 ------------------------------------------------------------
NC_000004.12:c1 ------------------------------------------------------------


NC_000013.11:c3 CAAGACTGCGTCCTTTTTCCCTTGAGCACCAGCCTTGGATAACTAGGGTCTGACAGAGGC
NC_000001.11:33 ------------------------------------------------------------
NC_000023.11:15 ------------------------------------------------------------
NC_000004.12:c1 ------------------------------------------------------------


NC_000013.11:c3 ACGCTTCAAGAGTGGAGCCTCAGTAGAGGTTTACTGTTTTCATCATGATGGCAGATGTGT
NC_000001.11:33 ------------------------------------------------------------
NC_000023.11:15 ------------------------------------------------------------
NC_000004.12:c1 ------------------------------------------------------------


NC_000013.11:c3 TGTGTCCCATCCACTGATTTTGCCCCACCGGGGGAGCCAGTCTTCATCCTTGAATCTTAT
NC_000001.11:33 ------------------------------------------------------------
NC_000023.11:15 ------------------------------------------------------------
NC_000004.12:c1 ------------------------------------------------------------


NC_000013.11:c3 AGTCCTAAGGTATGATGGGATTGGCATCTCAATTTAGAAAAATGCCAGGCCTCCGCCTTG
NC_000001.11:33 ------------------------------------------------------------
NC_000023.11:15 ------------------------------------------------------------
NC_000004.12:c1 ------------------------------------------------------------


NC_000013.11:c3 GGTCCTGTCCAAATCTTTCAGCTTTTGGAGATACGCATTCTTTCTTATTACTCCTTCTGG
NC_000001.11:33 ------------------------------------------------------------
NC_000023.11:15 ------------------------------------------------------------
NC_000004.12:c1 ------------------------------------------------------------


NC_000013.11:c3 ACATAGATAAGATCTAACCCTTCATTTAGCGTTTAGCGGTTAGTGCTTGCAATCTCCCTC
NC_000001.11:33 ------------------------------------------------------------
NC_000023.11:15 ------------------------------------------------------------
NC_000004.12:c1 ------------------------------------------------------------


NC_000013.11:c3 AGGCCACCTTTCTTAGATGTTGGGTAGCTTCGGAATGTTTTGGCATTGGCTTCCGCTCTC
NC_000001.11:33 ------------------------------------------------------------
NC_000023.11:15 ------------------------------------------------------------
NC_000004.12:c1 ------------------------------------------------------------


NC_000013.11:c3 CAGCCTTCACTCTTCCCCTTCTTGGCATTCTCTTTTTCTGTTGCTGTAGCAGATCTTCTT
NC_000001.11:33 ------------------------------------------------------------
NC_000023.11:15 ------------------------------------------------------------
```

```
NC_000004.12:c1 ------------------------------------------------------------

NC_000013.11:c3 CTAATCTCATCTGTGTGTTTTTTTTTTCTCCATCAAAAAATCCCTCTGAAAAAGATCGAA
NC_000001.11:33 ------------------------------------------------------------
NC_000023.11:15 ----------CTGTGTGATTTTGTT-----------------------------------
NC_000004.12:c1 ------------------------------------------------------------

NC_000013.11:c3 AGTTCAGTTCTAGCCACTGAAGAATGTATAGATACATGAAAGTGTTTTTTTGAGTCTTCT
NC_000001.11:33 ------------------------------------------------------------
NC_000023.11:15 ------------------------------------------------------------
NC_000004.12:c1 ------------------------------------------------------------

NC_000013.11:c3 AATTGTCTTTAACAAACATACAGTACTTGAAAACAACTTGTTATCATATAATACTTAGGT
NC_000001.11:33 ------------------------------------------------------------
NC_000023.11:15 ------------------------------------------------------------
NC_000004.12:c1 ------------------------------------------------------------

NC_000013.11:c3 CAGGACATAGTTTCTACCTGCAAGTTTACATTTATTTCTTAAGGTAAGGTTAAAGAAAAA
NC_000001.11:33 ------------------------------------------------------------
NC_000023.11:15 ------------------------------------------------------------
NC_000004.12:c1 ------------------------------------------------------------

NC_000013.11:c3 AAAACACACACAAGAGGAAAAAGATTCAAGAGACTAATATTACACCTCTTTAAAAGGCAG
NC_000001.11:33 ------------------------------------------------------------
NC_000023.11:15 ------------------------------------------------------------
NC_000004.12:c1 ------------------------------------------------------------

NC_000013.11:c3 AGCAGGAAACCATAGGGAGAGTCTCACAAAAATCCATTCTTCTGCTTGGGATGAGATTAT
NC_000001.11:33 ------------------------------------------------------------
NC_000023.11:15 ------------------------------------------------------------
NC_000004.12:c1 ------------------------------------------------------------

NC_000013.11:c3 TGATTTCCTTAGTTACTACTTTTCCCTGAAGTGTTGAAATAAGTGCCTTTTGATGCTTGG
NC_000001.11:33 ------------------------------------------------------------
NC_000023.11:15 ------------------------------------------------------------
NC_000004.12:c1 ------------------------------------------------------------

NC_000013.11:c3 ACTTGAGAAAAACTTGTTCCCCAAAGCCATTTGGCACGTATAAATCCCAGGAACTAGGTA
NC_000001.11:33 ------------------------------------------------------------
NC_000023.11:15 ------------------------------------------------------------
NC_000004.12:c1 ------------------------------------------------------------

NC_000013.11:c3 CTTTAGGCACTGTGTTTGCCAAGCATGAGAGAGAAGGGCGGGGAGGGGAGGAGACAGAAA
NC_000001.11:33 ------------------------------------------------------------
NC_000023.11:15 ----------TGTGTCTG------------------------------------------
NC_000004.12:c1 ------------------------------------------------------------

NC_000013.11:c3 AAGGAGCGAGGGAAGGAGGGAAAGTGGCTGTACTAGGTGCCAGAGGAACCATAGCACTGC
NC_000001.11:33 ------------------------------------------------------------
NC_000023.11:15 ------------------------------------------------------------
NC_000004.12:c1 ------------------------------------------------------------

NC_000013.11:c3 GTGAGAGCGACCGACATAGAAGGGAGCAGGTCCTCACTGAGGCCTGGCTCTCCTTCTCTG
```

```
NC_000001.11:33 ------------------------------------------------------------
NC_000023.11:15 ------------------------------------------------------------
NC_000004.12:c1 ------------------------------------------------------------


NC_000013.11:c3 AGTTCTACCAGCTTCACCCAGGGCATCTACATCACCCACACTGCTCCTGTGAAACTGGGC
NC_000001.11:33 ------------------------------------------------------------
NC_000023.11:15 ------------------------------------------------------------
NC_000004.12:c1 ------------------------------------------------------------


NC_000013.11:c3 TAATTTCATGCAAATCTAAATAGGTTTCATCCAAATGTAAAACCTACAGCTGAGTGCAGT
NC_000001.11:33 ------------------------------------------------------------
NC_000023.11:15 ----------------------AGTGGCATTCAGATG-----------------------
NC_000004.12:c1 ------------------------------------------------------------


NC_000013.11:c3 GGTCTCCTGTAGTTCCAGCTATTCAGGAGCCTGAGGCAGGAGGATCCCTTGAGCCTGGGC
NC_000001.11:33 ------------------------------------------------------CTGGGG
NC_000023.11:15 --------------------------------------------------AAGTCTGGAG
NC_000004.12:c1 --------------------------------------------------------GGAG
                                                                        **.

NC_000013.11:c3 AACATAGTGAGACCCCATCTCTAAAATTAAAATAAACAAACAAATATATATGTAAGGAAA
NC_000001.11:33 GACATAG-----------------------------------------------------
NC_000023.11:15 GAGTTAG-----------------------------------------------------
NC_000004.12:c1 GAAGAAG-----------------------------------------------------
                .*   **

NC_000013.11:c3 ACCTAGACTGAATTCATTGAATTTAGTGAATACACATTTTGTTGTCAAAGTGAATATTCT
NC_000001.11:33 ------------------------------------------------------------
NC_000023.11:15 ------------------------------------------------------------
NC_000004.12:c1 ------------------------------------------------------------


NC_000013.11:c3 ACACATCTCTAAAATTAAAATAAATGAACAAATATATAAATAAATGAAACGTAGACTGAA
NC_000001.11:33 ------------------------------------------------------------
NC_000023.11:15 ------------------------------------------------------------
NC_000004.12:c1 ------------------------------------------------------------


NC_000013.11:c3 TTCATTGAATTTAACGAATACATATTTTGGTGTCAAAGTAAATATTCCACACAAGGCAAT
NC_000001.11:33 ------------------------------------------------------------
NC_000023.11:15 ------------------------------------------------------------
NC_000004.12:c1 ------------------------------------------------------------


NC_000013.11:c3 ACATTTTGAAGGAGTTTATGAACTTCTATGATTTCTGGTTATATGTAGAAAATCACATGC
NC_000001.11:33 ------------------------------------------------------------
NC_000023.11:15 ------------------------------------------------------------
NC_000004.12:c1 ------------------------------------------------------------


NC_000013.11:c3 TTTAACTTCCGAAAGCATCTTTATTGTCACACAGTAAATATTTAAAAGGAGAGATTGGGT
NC_000001.11:33 ------------------------------------------------------------
NC_000023.11:15 ------------------------------------------------------------
NC_000004.12:c1 ------------------------------------------------------------


NC_000013.11:c3 TTTAGCAGCAGAGGAGGGGGAAGGACATTACAGGCCAGAGAAACAGTGTGACCAGAATCT
NC_000001.11:33 ------------------------------------------------------------
NC_000023.11:15 -----------------GAGAACGACA---------------------------------
NC_000004.12:c1 ------------------------------------------------------------
```

```
NC_000013.11:c3 TGAAGGTGGGAAAGTACAAGGTCCAGTTTGATTAGAAATCAGGAGGGCAGCAGGAGGGAA
NC_000001.11:33 ------------------------------------------------------------
NC_000023.11:15 ------------------------------------------------------------
NC_000004.12:c1 ------------------------------------------------------------

NC_000013.11:c3 AGCAAGAGAGAAACTGCCAGATTAGGGAGGGCCCTGAGGGCCAGAAGAGGATTTTGTAGT
NC_000001.11:33 ------------------------------------------------------------
NC_000023.11:15 ------------------------------------------------------------
NC_000004.12:c1 ------------------------------------------------------------

NC_000013.11:c3 TAATTTGGAAGAAAACATTGTAATTAAATAAAAAGTTTTTAAAGCAGTGGAGCTGTATGC
NC_000001.11:33 ------------------------------------------------------------
NC_000023.11:15 ------------------------------------------------------------
NC_000004.12:c1 ------------------------------------------------------------

NC_000013.11:c3 ATTAGGAAAATTATAATAACATCCAACATCTGTTGATCATTTACAGTGTTCTAAGCGATT
NC_000001.11:33 ------------------------------------------------------------
NC_000023.11:15 ------------------------------------------------------------
NC_000004.12:c1 ------------------------------------------------------------

NC_000013.11:c3 TACACTGTAGGCTGAGCTCTTTAGGTATATGAGCTCATTGAAACTCACAACACTCTTGCA
NC_000001.11:33 ------------------------------------------------------------
NC_000023.11:15 ------------------------------------------------------------
NC_000004.12:c1 ------------------------------------------------------------

NC_000013.11:c3 AAGGTGATATCATCATTTTATGGAAGCAGCTGAGGCTCAGAGAGGTTCATTCACTTTGCC
NC_000001.11:33 ------------------------------------------------------------
NC_000023.11:15 ------------------------------------------------------------
NC_000004.12:c1 ------------------------------------------------------------

NC_000013.11:c3 CAAGGTGACACAGCTAGTAAGCACCAGGACCAGGATTTTAAACTTAGAGGTGTCCGGTTC
NC_000001.11:33 ------------------------------------------------------------
NC_000023.11:15 ------------------------------------------------------------
NC_000004.12:c1 ------------------------------------------------------------

NC_000013.11:c3 CAAAGCCTCCATTCTTAACCACAAAATAATAAATGCCAGAACAAAAGAAGTGAAAAAAAG
NC_000001.11:33 ------------------------------------------------------------
NC_000023.11:15 ------------------------------------------------------------
NC_000004.12:c1 ------------------------------------------------------------

NC_000013.11:c3 AATTCAAAAACTCTTGTGACTTAAATGTTACTGAAGTTGGTGTGAGGGGAGAGGAGAAAG
NC_000001.11:33 ------------------------------------------------------------
NC_000023.11:15 ------------------------------------------------------------
NC_000004.12:c1 ------------------------------------------------------------

NC_000013.11:c3 GGGAGTCAAAAACAACTGCTAGAATTTGAGCCAGTGTGGGAATTCTGCAGGTAGTGAAAT
NC_000001.11:33 ------------------------------------------------------------
NC_000023.11:15 ------------------------------------------------------------
NC_000004.12:c1 ------------------------------------------------------------

NC_000013.11:c3 CGGAAAGAGGTAAGGTTTAGAGGGCAAACTGGAGAGCTTGCAAAACTGGTGAGTGGTGGG
NC_000001.11:33 ------------------------------------------------------------
NC_000023.11:15 ------TAGGCAAGGTTCAG----------------------------------------
```

```
NC_000004.12:c1 ------AAGATGAAGATGAG----------------------------------------

NC_000013.11:c3 AATAGGAGTCTGAAGTCCAGGATACAGCCTGGGGCTGGAATGGAAATCAAGGGTCATTTA
NC_000001.11:33 ------------------------------------------------------------
NC_000023.11:15 ------------------------------------------------------------
NC_000004.12:c1 ------------------------------------------------------------

NC_000013.11:c3 CATAATGACAGTGTTTAAAACGCCTCTAAGAAGAGTTGGTCACTGAGGTCTTAAAGAGAG
NC_000001.11:33 ------------------------------------------------------------
NC_000023.11:15 ------------------------------------------------------------
NC_000004.12:c1 ------------------------------------------------------------

NC_000013.11:c3 CAGAAGAGAAGCAAAGATTCGGACTAAGGTCCAAATTACAGGGAAAGAATGAATAGCTAT
NC_000001.11:33 ------------------------------------------------------------
NC_000023.11:15 ------------------------------------------------------------
NC_000004.12:c1 ------------------------------------------------------------

NC_000013.11:c3 TAGAGAAGACACATCAATAGTGACAAGAGGTAAAAGTTTAGAGCTATGATAAATGTGCAT
NC_000001.11:33 ------------------------------------------------------------
NC_000023.11:15 ------------------------------------------------------------
NC_000004.12:c1 ------------------------------------------------------------

NC_000013.11:c3 ATTTATCTTTACAGGGAATATTTCCCCATAGGGAACAGTTTCTAGAGTAATGCTGAATTT
NC_000001.11:33 ------------------------------------------------------------
NC_000023.11:15 ------------------------------------------------------------
NC_000004.12:c1 ------------------------------------------------------------

NC_000013.11:c3 GTAAAATTATCTGAATCTTTGAGTCCTCTAGTAGCACATTTCTTCTCTTAGAAATTGGGA
NC_000001.11:33 ------------------------------------------------------------
NC_000023.11:15 ------------------------------------------------------------
NC_000004.12:c1 ------------------------------------------------------------

NC_000013.11:c3 TTTGGCCCAGCTATTTGGGAGGCTGAGGCACGAGAATCGCTTGAACCCGGGAGGCAGAGG
NC_000001.11:33 ------------------------------------------------------------
NC_000023.11:15 ------------------------------------------------------------
NC_000004.12:c1 ------------------------------------------------------------

NC_000013.11:c3 TTGCAGTGAGCTGAGATCGCGCCACTGCACTCCAGCCTAGGTGACAGAGGGAGACTCTGT
NC_000001.11:33 ------------------------CTGCCTCATAGCCTA---------------------
NC_000023.11:15 ------------------------CAGCCTTCCAAGGTATAGGAAGGTGGGTGA------
NC_000004.12:c1 ----------------------------------------GAGGAAGAGGATGA------

NC_000013.11:c3 CTCAAAAAAAAAAAAAAAGAAAAGAAAAGAAAAGAAAAAAGAAATTGGGATTTGGGGGCT
NC_000001.11:33 ------------------------------------------------------------
NC_000023.11:15 ------------------------------------------------------------
NC_000004.12:c1 ------------------------------------------------------------

NC_000013.11:c3 GGGCACCGTGTGGCTCATGCCTATAATCCCAGCACTTTGGGAGGCTGAGGTGGGAAGATC
NC_000001.11:33 ------------------------------------------------------------
NC_000023.11:15 ------------------------------------------------------------
NC_000004.12:c1 ------------------------------------------------------------

NC_000013.11:c3 ACTTGAGCCCAAGAGTTTGAGGCTGCAGTGAGCTATGATTGTGCCAATGCACTCCAGCCT
```

```
NC_000001.11:33 --------------------------------CTATGAATGAACC---------------
NC_000023.11:15 --------------------------------TTAGGACTGAGGCTAT------------
NC_000004.12:c1 -----------------------------AGATGAAGAATAAATG---------------
                                                . * ** *. .

NC_000013.11:c3 GGGCAACAGAGCAAGAACCTGTCTCAAAAAAGAAAAGAAAAAGAAAGCAAAGATTATCTT
NC_000001.11:33 ----------------------------------------------------ATCATCTT
NC_000023.11:15 -----------------------------------------------CTAGGTTTAACTT
NC_000004.12:c1 ----------------------------------------------------GCTATCCT
                                                                     ..* *.*

NC_000013.11:c3 CCATCAGAAATAAATATGATTAACATTTATTAGTATATTCTTTCAGTCTTTTCTGGATTC
NC_000001.11:33 TCATGAGAGGCAAACATTG-----------------------------------------
NC_000023.11:15 TTGT--------------------------------------------------------
NC_000004.12:c1 TTAA--------------------------------------------------------
                ...

NC_000013.11:c3 ATACATACTCTTTTTCAAACAGGGATCCTTCTGAAAATAGTTTTTCTCACAAGATATTGT
NC_000001.11:33 ------------------------------------------------------------
NC_000023.11:15 ------------------------------------------------------------
NC_000004.12:c1 ------------------------------------------------------------


NC_000013.11:c3 GTATATTTTTGCAAGGCATTCAGTTTAGTATTCATAAGAATTTGATAGTGTTATAATGAT
NC_000001.11:33 ------------------------------------------------------------
NC_000023.11:15 ------------------------------------------------------------
NC_000004.12:c1 ------------------------------------------------------------


NC_000013.11:c3 AATAATGCATCATAAAGGCAGATTAAAATGCATTTAGCCAAACCTCTCATTGGACATCTA
NC_000001.11:33 ------------------------------------------------------------
NC_000023.11:15 ------------------------------------------------------------
NC_000004.12:c1 ------------------------------------------------------------


NC_000013.11:c3 GGTTGTTTCCACATTATAAATAATGCTGCAATTCATAATTTTTTTAATTAAAAATAATTC
NC_000001.11:33 ------------------------------------------------------------
NC_000023.11:15 ------------------------------------------------------------
NC_000004.12:c1 ------------------------------------------------------------


NC_000013.11:c3 TTTTTAGAGACAGGGTCTCACTCTGTCCCCAGGCTGGAGTGTGTCTATTCACAGGTACAA
NC_000001.11:33 ------------------------------------------------------------
NC_000023.11:15 ------------------------------------------------------------
NC_000004.12:c1 ------------------------------------------------------------


NC_000013.11:c3 TGACAGCTCACTGCAGCCTTGAACTCCCGGGCCCAAGTGATCCTCCTGCCTTGGCCTCCC
NC_000001.11:33 ------------------------------------------------------------
NC_000023.11:15 --------------------------------------------CCCACCTCCACCCCC-
NC_000004.12:c1 ------------------------------------------------------------


NC_000013.11:c3 AAAGTACTGGGAATACAGGAGTGAGCCACCATGCCTGGCCACTTTTTACTTATTTCTGAA
NC_000001.11:33 ------------------------------------------------------------
NC_000023.11:15 ------------------------------------------------------------
NC_000004.12:c1 ------------------------------------------------------------


NC_000013.11:c3 TATTTCCTAGAAGCAAAATCACTGGTCAAATGCCATGAGTGTGTTCAATGCAGAGTTCAT
NC_000001.11:33 ------------------------------------------------------------
NC_000023.11:15 TATTTTGTGGGGCCAAATGCATTGCTAAA-------------------------------
NC_000004.12:c1 ------------------------------------------------------------
```

```
NC_000013.11:c3 TTCTGAACATGACTATCAATTTGCTGGTAGTACTTATGTTAGACTGTAGTGAACCATGCT
NC_000001.11:33 ------------------------------------------------------------
NC_000023.11:15 ------------------------------------------------------------
NC_000004.12:c1 ------------------------------------------------------------

NC_000013.11:c3 ATCTCTTTCCTACTTCCTCATTTCCATTCTTGTAAGTCAAAAGGAAACCTTACAACAAGG
NC_000001.11:33 ------------------------------------------------------------
NC_000023.11:15 ------------------------------------------------------------
NC_000004.12:c1 ------------------------------------------------------------

NC_000013.11:c3 ATCAACAGCAACCTTTCAGAAAAAGCATACACGTTTTTTGTTGTTGATTTTTTTTTTCTT
NC_000001.11:33 ------------------------------------------------------------
NC_000023.11:15 ------------------------------------------------------------
NC_000004.12:c1 ------------------------------------------------------------

NC_000013.11:c3 TAACACCTTCAGGTATGGGTTAGCAGTCACCCTCTGTTTACCCTTACAAACCGCTTTGGG
NC_000001.11:33 ----ACCCTCAAG-----------------------------------------------
NC_000023.11:15 CAGCAATTTCAGA----------------------------------------------G
NC_000004.12:c1 ------------------------------------------------------------

NC_000013.11:c3 CACTCAGTGTTTTTTAAACAAAGAGGCCTGGTAACTTGGATGGTAAGGCTGTCTGTCCCA
NC_000001.11:33 ------------------------------------------------------------
NC_000023.11:15 TGTATGGTGTGTCAAAAATTAAGG------------------------------------
NC_000004.12:c1 ------------------------------------------------------------

NC_000013.11:c3 ACCGGTCCAGCTCCTCACTGGCAGAGGGACTTCTGCATTTCCAGCCTGTGATTTTAGTTA
NC_000001.11:33 ------------------------------------------------------------
NC_000023.11:15 ------------------------------------------------------------
NC_000004.12:c1 ------------------------------------------------------------

NC_000013.11:c3 GAGGCAAATCCAAGGTGGGGAGCCTCACTCAGGTGAGGAAGATATTTGTCAGGTCTGCTA
NC_000001.11:33 ------------------------------------------------------------
NC_000023.11:15 ------------------------------------------------------------
NC_000004.12:c1 ------------------------------------------------------------

NC_000013.11:c3 GGATTTCTCTCCCAGGCTTCAGAAATTTGCAAGCATCTTTGCATTTCCTGGCATTCTCTG
NC_000001.11:33 ------------------------------------------------------------
NC_000023.11:15 ------------------------------------------------------------
NC_000004.12:c1 ------------------------------------------------------------

NC_000013.11:c3 GCCTGGAATGCTGTTTTTTGTCTTGGGCCTCCCTGGCAGAGCAAGGCAGTCCCCTTTTCT
NC_000001.11:33 ------------------------------------------------------------
NC_000023.11:15 ------------------------------------------------------------
NC_000004.12:c1 ------------------------------------------------------------

NC_000013.11:c3 GTCTTCAGAGCCTTTCTCTGGGCTTGGGACAGGCCCACTGTGTTGGCACAAGCTTCATTT
NC_000001.11:33 ------------------------------------------------------------
NC_000023.11:15 ------------------------------------------------------------
NC_000004.12:c1 ------------------------------------------------------------

NC_000013.11:c3 GGTCTGCTGGGGAGCTTGGCTGCAGGCTCTGGGTGCTCGGCATCTCCCCTGGCTGCTCCT
NC_000001.11:33 ------------------------------------------------------------
NC_000023.11:15 ---------------------------------------------------------CCT
```

```
NC_000004.12:c1 ------------------------------------------------------------

NC_000013.11:c3 TTCTGTCCTATTCAATCAGCCTCAGCACCTGCACTTGTCAGTTAGGCCTGATGTTACCCT
NC_000001.11:33 --------TAACCAACCAATACGAGCACCTGTAC--------------------------
NC_000023.11:15 TATTGTTTTTCTCTTTCACCCCTACCCCCCGTGC--------------------------
NC_000004.12:c1 ------------------------------------------------------------

NC_000013.11:c3 GGATGGGAAATGGAACTTCTTTACTGTTGCTCTAATTCCATGTTTCACAAACCGCAGGGC
NC_000001.11:33 ------------------------------------------------------------
NC_000023.11:15 ------------------------------------------------------------
NC_000004.12:c1 ------------------------------------------------------------

NC_000013.11:c3 CTATGGATGGATTGGGAAGTAAATTTAAAATGTGAAGGAAAAAAAAATCGGAGTCCATCG
NC_000001.11:33 ------------------------------------------------------------
NC_000023.11:15 ------------------------------------------------------------
NC_000004.12:c1 ------------------------------------------------------------

NC_000013.11:c3 CAACAATGGTAGGTATTGTTTCATTAAACTTTTTTCAGATTTTATATATGCAAACTGTAA
NC_000001.11:33 ------------------------------------------------------------
NC_000023.11:15 ------------------------------------------------------------
NC_000004.12:c1 ------------------------------------------------------------

NC_000013.11:c3 GGGTCCTATTCAAAGACCTTGAAAGCCAAGAATGTGAACTGTGCATTTCAAACGTATGTC
NC_000001.11:33 ------------------------------------------------------------
NC_000023.11:15 ------------------------------------------------------------
NC_000004.12:c1 ------------------------------------------------------------

NC_000013.11:c3 AGTATTCCTATGGGGCTGTGATGCATATTTAGGTGGGGAAATGTGATACCGTTTTTATTT
NC_000001.11:33 ------------------------------------------------------------
NC_000023.11:15 ------------------------------------------------------------
NC_000004.12:c1 ------------------------------------------------------------

NC_000013.11:c3 TATTAGCTAGGCTAATAACCGCAAGATCATATGGCTTTAGTGGAGAAAACACAAGGCTTT
NC_000001.11:33 ------------------------------------------------------------
NC_000023.11:15 ------------------------------------------------------------
NC_000004.12:c1 ------------------------------------------------------------

NC_000013.11:c3 GGACTCAGAACTGGGTCAATATTGTTGCTAACTAGCTGTGTGACCTTGGACAATTCAACG
NC_000001.11:33 ------------------------------------------------------------
NC_000023.11:15 ------------------------------------------------------------
NC_000004.12:c1 ------------------------------------------------------------

NC_000013.11:c3 AATCTCTCTGAGCCTCCCTTAGTATTGCCTGTAAAAGACAGATAAGCATCCTGGCCTTAT
NC_000001.11:33 ------------------------------------------------------------
NC_000023.11:15 ------------------------------------------------TCCTGGC-----
NC_000004.12:c1 ------------------------------------------------------------

NC_000013.11:c3 AGGATTACTGTTAGGATTAAACAAAATCTTTTTTTTTTTTTTTTGAGACAGAATCTCGCT
NC_000001.11:33 ------------------------------------------------------------
NC_000023.11:15 ------------------------------------------------------------
NC_000004.12:c1 ------------------------------------------------------------

NC_000013.11:c3 CTTTTGCCCAGGCTGGAGCACAGTGGTATGATCTTGGCTCTCTCTAACCTTCGCCTCCTG
```

```
NC_000001.11:33 ------------------------------------------------------------
NC_000023.11:15 ------------------------------------------------------------
NC_000004.12:c1 ------------------------------------------------------------

NC_000013.11:c3 GGTTCAAGTGATTCTCGTGCCTCAACCTCCCTAGTATCTGGGATCACAGGCACACGCCAC
NC_000001.11:33 ------------------------------------------------------------
NC_000023.11:15 ------------------------------------------------------------
NC_000004.12:c1 ------------------------------------------------------------

NC_000013.11:c3 CACACTCAGCTAATACTTTTGTACTTTTAGTAGAGCTGGGGTTTTGCCATGTTGGCCAGG
NC_000001.11:33 ------------------------------------------------------------
NC_000023.11:15 ------------------------------------------------------------
NC_000004.12:c1 ------------------------------------------------------------

NC_000013.11:c3 CTGGTCTTGAACTCCTGGGCTCAAGTGATCCACCCACCTCTGCCTCCCAAAATGCTGGGA
NC_000001.11:33 ------------------------------------------------------------
NC_000023.11:15 ------------------------------------------------------------
NC_000004.12:c1 ------------------------------------------------------------

NC_000013.11:c3 TTACAAGCGTGAGCCATCACACCAGCCATAAACAAAATACTCTATGTATAGTGCTTAGCA
NC_000001.11:33 ------------------------------------------------------------
NC_000023.11:15 -------------------------------ACATATCACATTATTTGTGGTGCCC----
NC_000004.12:c1 ------------------------------------------------------------

NC_000013.11:c3 CATGGGAGACACATGCTAAATAAAGCTTGTTGTCTAGTCTTTTGTCTGGGCCATAAGACA
NC_000001.11:33 ------------------------------------------------------------
NC_000023.11:15 ------------------------------------------------------------
NC_000004.12:c1 ------------------------------------------------------------

NC_000013.11:c3 TCCTCTCTTGGCAGCACAGCCACTCTCTTCCTCATCTCAAGAGTACATGCTGGCTGGGCG
NC_000001.11:33 ------------------------------------------------------------
NC_000023.11:15 ------------------------------------------------------------
NC_000004.12:c1 ------------------------------------------------------------

NC_000013.11:c3 CGATGGCTCAGGCCTGTAATCCCAGCACTTTGGGAGGCTGAGGTGGGCAGAACACTTGAG
NC_000001.11:33 ------------------------------------------------------------
NC_000023.11:15 --------------------------------------------------AACATTTGGG
NC_000004.12:c1 ------------------------------------------------------------

NC_000013.11:c3 GTCAGGAGTTCGAGACCAGTCTGGCCAACATGGTGAAACCCCATCTCTACTGAAAATACA
NC_000001.11:33 ------------------------------------------------------------
NC_000023.11:15 GTCTTGAGCCTGCTGCTGGTCT--------------------------------------
NC_000004.12:c1 ------------------------------------------------------------

NC_000013.11:c3 AAAAATTAGCTGAGTGTGGTGGCTTGCGCCTGTAATCCCACTACTCGGGAGGCTGAGCTT
NC_000001.11:33 ------------------------------------------------------------
NC_000023.11:15 ------------------------------------------------------------
NC_000004.12:c1 ------------------------------------------------------------

NC_000013.11:c3 GAGCCTGAGAGGCTGAGGTCGCAATGAGGCTGCACTCCAGCCTGGGCAAAAGAGTAAGAC
NC_000001.11:33 ------------------------------------------------------------
NC_000023.11:15 ------------------------------------------------------------
NC_000004.12:c1 ------------------------------------------------------------
```

```
NC_000013.11:c3 TCTGTCTCAAAATAAAAAAACTGAGAGAGAGTACATGCTACCTGTGTCTCAAAAAAAAAA
NC_000001.11:33 ------------------------------------------------------------
NC_000023.11:15 ------------------------------------------------------------
NC_000004.12:c1 ------------------------------------------------------------

NC_000013.11:c3 AAAAAAAAAAAGTACATGCCACCTGCGATATCACAACAGTTCTGTTTCTCAGTTTCCCAC
NC_000001.11:33 ------------------------------------------------------------
NC_000023.11:15 ------------------------------------------------------------
NC_000004.12:c1 ------------------------------------------------------------

NC_000013.11:c3 TCTATGGTTTGTATGATGACAATAAGTGTTTCCTATAAAAAGAATCTCTGATCAAGAAAA
NC_000001.11:33 ------------------------------------------------------------
NC_000023.11:15 ------------------------------------------------------------
NC_000004.12:c1 ------------------------------------------------------------

NC_000013.11:c3 TTTCAGAATTACAGTTTACAATCATATGAAATGCTTAGGCCCCAACTCAAATGTCTGCAG
NC_000001.11:33 ----------------------------------------------ACAAATGCCCACAG
NC_000023.11:15 ---------------------------------------------CCTGGATGCCAGTGA
NC_000004.12:c1 ------------------------------------------------------------

NC_000013.11:c3 GGGCCAGGGAGGTGATATGATTGGGTGAAAAGTCAGGCTAACCACTTAGGGAATGGCGGG
NC_000001.11:33 G-----------------------------------------------------------
NC_000023.11:15 G-----------------------------------------------------------
NC_000004.12:c1 ------------------------------------------------------------

NC_000013.11:c3 GACTGTGGTAAACTTGAGAACCCTGACCTCTTTGAGGGAGGCAGTTACTCTTCATTCCAA
NC_000001.11:33 ------------------------------------------------------------
NC_000023.11:15 ------------------------------------------------------------
NC_000004.12:c1 ------------------------------------------------------------

NC_000013.11:c3 CCATCCTCCCCATGTGGAAATGTGAACCCAACCTTATCAGATCTCTCACTTTTCAAAAGA
NC_000001.11:33 ------------------------------------------------------------
NC_000023.11:15 ------------------------------------------------------------
NC_000004.12:c1 ------------------------------------------------------------

NC_000013.11:c3 AGCCAAATATCTGGATTTTTTATTGTGAAGATTTCTACTGTTTTCTGGGTTTTGTTTGTT
NC_000001.11:33 --------------------------------------TGTTGGCTGGGATTGGCTGGCT
NC_000023.11:15 ---------------------------------------GGTATGTGGGATGGGGTGGTG
NC_000004.12:c1 --------------------------------------TGATGCGTGTGGAATGTGTGTG
                                                       * *   ** *    *   *.

NC_000013.11:c3 TGTTTAGAGACAGGGTCTCTCTCTGTTGCCCAGGCTGGATGGAGTGTGATGGCAGGATCA
NC_000001.11:33 TGT---------------------------------------------------------
NC_000023.11:15 GGGTAGGGGACGGTATCCTTTT--------------------------------------
NC_000004.12:c1 TGT---------------------------------------------------------
                 *

NC_000013.11:c3 TAGCTCACTGCAGCCTCAAACTCCCGGGCTCAAACAATCCTCCTATCTCAGCCTTCTGAG
NC_000001.11:33 ------------------------------------------------------------
NC_000023.11:15 ------------------------------------------------------------
NC_000004.12:c1 ------------------------------------------------------------

NC_000013.11:c3 TGAGTAGGTGGAACTACAGGTGAGCGCCACCACCACACCCAGGGAATTTTTTTTTTTTTT
NC_000001.11:33 ------------------------------------------------------------
NC_000023.11:15 ------------------------------------------------------------
```

```
NC_000004.12:c1 ------------------------------------------------------------

NC_000013.11:c3 TTTTAGAGATGGTCTCAAACCGGGCACATTGGCTCACACTTGTAATCCCAGCACTTTGGG
NC_000001.11:33 ------------------------------------------------------------
NC_000023.11:15 ----------------------------TTTGCTCCTACTTG------------------
NC_000004.12:c1 ------------------------------------------------------------

NC_000013.11:c3 AGGCTGAGACAGGCAGATCACTTGAGCCCAGGAGTTCAAGACCAGCCCAGGCAACATGGA
NC_000001.11:33 ------------------------------------------------------------
NC_000023.11:15 ------------------------------------------------------------
NC_000004.12:c1 ------------------------------------------------------------

NC_000013.11:c3 GAGACCCCCATCTCTACAAAAAATACAAAAACATAATTAACCAGGCATGATGGTGTGCAC
NC_000001.11:33 ------------------------------------------------------------
NC_000023.11:15 -------------------GAAACACCAAA------------------------------
NC_000004.12:c1 ------------------------------------------------------------

NC_000013.11:c3 CTGTAGTCCCAGCTACTTGGGGTGGGGTGAAGGGAGGGGAGAGGTGGGAGGAGACTGCAG
NC_000001.11:33 ------------------------------------------------------------
NC_000023.11:15 ------------------------------------------------------------
NC_000004.12:c1 ------------------------------------------------------------

NC_000013.11:c3 TGAGCCGAGGTCACGCCACTGCACTCCAGCCTAGGTGAGACCCTATCTCAAAAAAAAAAA
NC_000001.11:33 ------------------------------------------------------------
NC_000023.11:15 ------------------------------------------------------------
NC_000004.12:c1 ------------------------------------------------------------

NC_000013.11:c3 AAAAGAGAGAGAGAGAGACAGTGAGCTGAAGTCGCATCACTGCACTCCAGCCTAGGCGAG
NC_000001.11:33 ------------------------------------------------------------
NC_000023.11:15 ------------------------------------------------------------
NC_000004.12:c1 ------------------------------------------------------------

NC_000013.11:c3 ACCCTGTCTCAAAAAAAAAAAAAAAAAAAAAGAGAGAGAGAGAGAGAGAGACAGTGAGCC
NC_000001.11:33 ------------------------------------------------------------
NC_000023.11:15 ------------------------------------------------------------
NC_000004.12:c1 ------------------------------------------------------------

NC_000013.11:c3 AAGGTTGCACCACTGCACTCCAGCCTAGGCGACAGAGCGAGACTGTCTCAAAAAAAAAAA
NC_000001.11:33 ------------------------------------------------------------
NC_000023.11:15 ------------------------------------------------------------
NC_000004.12:c1 ------------------------------------------------------------

NC_000013.11:c3 AAAAGAGAGAGAGAGAGAGAGCTGAGGTCGCATCACTGCACTCCAGCCTAAGGGAGAC
NC_000001.11:33 ------------------------------------------------------------
NC_000023.11:15 ---------------------------------------------CACCCCAAGGAAGA-
NC_000004.12:c1 ------------------------------------------------------------

NC_000013.11:c3 CCTGTCTCAAAAAAAAAAAAAAAAAAAAAACAAAAAAAGAAAAGAAAAAAAAAAGAGAAA
NC_000001.11:33 ------------------------------------------------------------
NC_000023.11:15 ------------------------------------------------------------
NC_000004.12:c1 ------------------------------------------------------------

NC_000013.11:c3 GAGAGTGAGCCAAGGTTGCACCATTGCACTCCAGCCTAGGCGAAAGAGCAAGACCCTGTC
```

```
NC_000001.11:33 ------------------------------------------------------------
NC_000023.11:15 ------------------------------------------------------------
NC_000004.12:c1 ------------------------------------------------------------


NC_000013.11:c3 TCAAAAAAAAAAAAACAAAAAAAGAGAGAGAGAGAGTGCCAAGGTTGCACCATTGCACTC
NC_000001.11:33 ------------------------------------------------------------
NC_000023.11:15 --------------------------------------------------TGATAGGCTC
NC_000004.12:c1 ------------------------------------------------------------


NC_000013.11:c3 CAGCTTAGGCGATAGAGCGAAACCATCTCAAAAAAAAAAAAAAAAAAAAAGGAGAGAGAG
NC_000001.11:33 ------------------------------------------------------------
NC_000023.11:15 CATCTTGGGC--------------------------------------------------
NC_000004.12:c1 ------------------------------------------------------------


NC_000013.11:c3 ACCATCTCGCTCTGTCGCCCAGGCTGGAGTGCAGTGGTTCAATCACTGCTTACTGCATCC
NC_000001.11:33 ------------------------------------------------------------
NC_000023.11:15 ---------------CACCTGAGCTATAGGGCAGG-------------------------
NC_000004.12:c1 ------------------------------------------------------------


NC_000013.11:c3 TTGAACTCCTCTTCTGCCTCGGCCTCCCAAAGTGCTAAGATTACAAGTGTGAGCCACCAT
NC_000001.11:33 ------------------------------------------------------------
NC_000023.11:15 ------------------------------------------------------------
NC_000004.12:c1 ------------------------------------------------------------


NC_000013.11:c3 ACCTGCCCAATGTCTACCTTTTTAAAACACCGCTTTGACCGAATGAAACGTCTGTGGGTC
NC_000001.11:33 ------------------------------------------------------------
NC_000023.11:15 ------------------------------------------------------------
NC_000004.12:c1 ------------------------------------------------------------


NC_000013.11:c3 AGATAAGGCCAGAGAGGGGCCAGTTTACGCTGTCAGTCCTAACCCTTCATGGCTGGGCAT
NC_000001.11:33 ------------------------------------------------------------
NC_000023.11:15 ------------------------------------------------------------
NC_000004.12:c1 ------------------------------------------------------------


NC_000013.11:c3 ACTCATTACGGTCACCCCCGGCTCTCCAGAAAAGCCTCCCACGGTATCCCTATAAAAGCG
NC_000001.11:33 ------------------------------------------------------------
NC_000023.11:15 ------------------------------------------------------------
NC_000004.12:c1 ------------------------------------------------------------


NC_000013.11:c3 CTTGTTTAAAATTACTATAATATTTTGCAGCAAAAATATACTTTAAGCACAGAATATGTA
NC_000001.11:33 ------------------------------------------------------------
NC_000023.11:15 ------------------------------------------------------------
NC_000004.12:c1 ------------------------------------------------------------


NC_000013.11:c3 ACTCCAATAATAGATGAAAATTCTACCTAATGAAGAGGACCATCTTTTTCACAAGGTGTT
NC_000001.11:33 ------------------------------------------------------------
NC_000023.11:15 --------------------------CTAATGGAATCAACCATTTCT-------------
NC_000004.12:c1 ------------------------------------------------------------


NC_000013.11:c3 AAACACCATGAACAGCAGGAAAGAAATAGGCAAAGTTGGCGAGGGAGTGTTCCATTGTTC
NC_000001.11:33 ------------------------------------------------------------
NC_000023.11:15 ------------------------------------------------------------
NC_000004.12:c1 ------------------------------------------------------------
```

```
NC_000013.11:c3 CTGTCTTTAGTTAACCAAAACCACTCGTAAGACATTTATTGCTTTTTAAAAAATAGCTTT
NC_000001.11:33 ------------------------------------------------------------
NC_000023.11:15 -----------------------------GAGCACTAAATGTATCATGAAAAGTT-----
NC_000004.12:c1 ------------------------------------------------------------

NC_000013.11:c3 ATCAAAATATGATTCATACACCATGTAATTTACCCACTTACAGTGTACAATTCAATGGTT
NC_000001.11:33 ------------------------------------------------------------
NC_000023.11:15 ------------------------------------------------------------
NC_000004.12:c1 ------------------------------------------------------------

NC_000013.11:c3 TTGGGCATATTTACCAGGTCATGCTGCCATTACTATAATCTAATCTGCAACATTTTTGTA
NC_000001.11:33 ------------------------------------------------------------
NC_000023.11:15 ------------------------------------------------------------
NC_000004.12:c1 ------------------------------------------------------------

NC_000013.11:c3 CCCCTAAAAGAAACTCCATACCCATTAGCAGGCACTCTCTTTTTCCCCAAACCCTGGCAG
NC_000001.11:33 ------------------------------------------------------------
NC_000023.11:15 ------------------------------------------------------------
NC_000004.12:c1 ------------------------------------------------------------

NC_000013.11:c3 CCACTAATCTACTTCCCTGTCTATAATATTTCCTGTTCTGGATATTTTATATAAATGGAA
NC_000001.11:33 ------------------------------------------------------------
NC_000023.11:15 ------------------------------------------------------------
NC_000004.12:c1 ------------------------------------------------------------

NC_000013.11:c3 TCATACAATATGTGGCCTTCTTATGTCTGGGCTCTTTCACTTAGCATAATGTCAAATATA
NC_000001.11:33 ------------------------------------------------------------
NC_000023.11:15 ------------------------------------------------------------
NC_000004.12:c1 ------------------------------------------------------------

NC_000013.11:c3 TATGTATGTGTATGGATATACCACATTCGATTTATCCACTCATCAGTTGATGGGCATTTG
NC_000001.11:33 ------------------------------------------------------------
NC_000023.11:15 ------------------------------------------------------------
NC_000004.12:c1 ------------------------------------------------------------

NC_000013.11:c3 GGTGTTTTCCACTTTTTGACTGTTACAAATGCTGCTGCTATGAAGATTCATGTACAAGTG
NC_000001.11:33 ------------------------------------------------------------
NC_000023.11:15 ------------------------------------------------------------
NC_000004.12:c1 ------------------------------------------------------------

NC_000013.11:c3 TGTGTGGACATGTTTTTGTTTCTTTTGGGTATATACCTAGGAGTGGAATTGCTGGGTCAA
NC_000001.11:33 ------------------------------------------------------------
NC_000023.11:15 ------------------------------------------------------------
NC_000004.12:c1 ------------------------------------------------------------

NC_000013.11:c3 CTGCTATCTATCTGTGTTTAACCTTTTGAGGAACTGTCAGCTTGTTTTCCAAAACACCTG
NC_000001.11:33 ------------------------------------------------------------
NC_000023.11:15 ------------------------------------------------------------
NC_000004.12:c1 ------------------------------------------------------------

NC_000013.11:c3 CCCGTTTACATTTCCACAAGTGTGCATAAGGGTTCCAGTTTCTTTCTCCACATGCTCACC
NC_000001.11:33 ------------------------------------------------------------
NC_000023.11:15 ------------------------------------------------------------
```

```
NC_000004.12:c1 ------------------------------------------------------------


NC_000013.11:c3 CATGCTTGCTACTGTCTATCTCCTTGATTATAGCCATCTGCGTGGGTGTGTGATAAGCAT
NC_000001.11:33 ------------------------------------------------------------
NC_000023.11:15 ------------------------------------------------------------
NC_000004.12:c1 ------------------------------------------------------------


NC_000013.11:c3 TTATTCTGAAGGTGTAAGGCAGTTTTAATAGAGCAGCTGAATTTGATGTTTGATGTGATT
NC_000001.11:33 ------------------------------------------------------------
NC_000023.11:15 ------------------------------------------------------------
NC_000004.12:c1 ------------------------------------------------------------


NC_000013.11:c3 ATTGAGATCAAGACCTAATATAGGGATCTTTTCTGAACTTAGATCACCTTTCTGTAAGAA
NC_000001.11:33 ------------------------------------------------------------
NC_000023.11:15 ------------------------------------------------------------
NC_000004.12:c1 ------------------------------------------------------------


NC_000013.11:c3 CTTTTACTATTGCGTTTTCCACTCGTCATGTGTACCATTCTTTGACTAACCAGAGGACAG
NC_000001.11:33 ------------------------------------------------------------
NC_000023.11:15 ------------------------------------------------------------
NC_000004.12:c1 ------------------------------------------------------------


NC_000013.11:c3 GCCTCTTAAGGAAAGGT---CTGATATTTTCCCCTTACTAACCAGTAAAATCATCCAATT
NC_000001.11:33 ----------GGATGAT---GTCACAGTTTCCCTCCTCTGAC------------------
NC_000023.11:15 ----------GAATGGCCTGCTCATAAGTTTAGCTCATTCACTGGAA-------------
NC_000004.12:c1 -------------------GCTCAGGCAATTATTTTGCTAAG------------------
                                     * * .   *.  ... .* *

NC_000013.11:c3 CTAGAGAACTTATTGATATAAATTAGCTTTGGTACAATTTTCTGTGCCATAGCATTGATG
NC_000001.11:33 ------------------------------------------------------------
NC_000023.11:15 ---------------ATGTAGATTG-----------------------------------
NC_000004.12:c1 ------------------------------------------------------------


NC_000013.11:c3 GCTATCATCAATGCTGCCCACGGAGAAGCTGCTGATTTCCCCCCGGAAAACTCGGGCTGA
NC_000001.11:33 ----TCAACAATGCCATC------------------------------------------
NC_000023.11:15 ---ATGTTCAATGTT---------------------------------------------
NC_000004.12:c1 ---------AATGTG---------------------------------------------
                         ****.

NC_000013.11:c3 GGCAGGAGGTACTTAAGCAGCTGGGGAAGAGGTTCTCCTTCTCCATACATGAACACATAT
NC_000001.11:33 ------------------------------------------------------------
NC_000023.11:15 ------------------------------------------------------------
NC_000004.12:c1 ------------------------------------------------------------


NC_000013.11:c3 AGCACCATCTCCCTCTCTTTCGCCTCTGGTCTTTCTTCTTGCCTCTCCATTTAGTCATGA
NC_000001.11:33 ------------------------------------------------------------
NC_000023.11:15 ------------------------------------------------------------
NC_000004.12:c1 ------------------------------------------------------------


NC_000013.11:c3 AACATCCTTTCAAGTTAGACTAAGGTAGTGACAGCCCTAAGGAAATGTTGAGGAAACTGA
NC_000001.11:33 -------------------------------------------AGCACTGAAAAGGCTGA
NC_000023.11:15 -------------------------------------------AAACTGGAAGGAGCTTG
NC_000004.12:c1 -------------------------------------------AATTCAAGTGCAGCTCA
                                                           *.  . .. . ..** .

NC_000013.11:c3 GCATAGACCTCGGTGACTATGAGAGGTCCGTGCATAGTGAATTGAGGGGATTGGATGTCA
```

```
NC_000001.11:33 GCCCTGACTACAG-----------------------------------------------
NC_000023.11:15 GTTTGTGTGTCAGTGGTTAT-----ATTAGTGGGTAGTGTAACAT---------------
NC_000004.12:c1 ATACTAGCTTCAGT---------------ATAAAAACTGTACAGA---------------
                .. .  ..  *.*

NC_000013.11:c3 GTATCCTTGCTTCTAGTCTGGAGTTCACAAACCTGTGCTCTCTTCTTTTCCTAAATGCCT
NC_000001.11:33 ---------------------------------------CTCTT----------------
NC_000023.11:15 ---------------------------------------TTTATCCAGGTTGGGGTG---
NC_000004.12:c1 ---------------------------------------TTTTTGT--------------
                                                       .*. *

NC_000013.11:c3 CATTCCTTTATTTTCACTGCCCTAAAAAAAAAAAAAAAAGTTTTCAGTATGTGTGATCCT
NC_000001.11:33 ------------------------------------------------------------
NC_000023.11:15 ------------------------------AGGGGAGATGGCCACAGTA-----------
NC_000004.12:c1 ------------------------------------------------------------


NC_000013.11:c3 TAGTATGGTTGTATGGTTATATGGTTGACCTCATCAGACATTTTGGGTTAGCGTTTCCTC
NC_000001.11:33 ------------------------------------------------------------
NC_000023.11:15 ------------------------------------------------------------
NC_000004.12:c1 ------------------------------------------------------------


NC_000013.11:c3 TCCTTTTCCGAAACTGGCCTGTTTTGCAAAGCAGACAAGAGAATTCATATAGCTTGACTT
NC_000001.11:33 ------------------------------GCAGACAGAAAAATTCG-------------
NC_000023.11:15 ------------------------------GCAAGTGGTGACACTAA-------------
NC_000004.12:c1 ------------------------------ATAGCTGATAAGATTCT-------------
                                              ..*. ... .. *.*

NC_000013.11:c3 TAGGACTCATTGCATTGGGAGAGATCAATATTTTCAACCAATGAAAAACTTACAGCTCCC
NC_000001.11:33 ------------------------------------------------------------
NC_000023.11:15 ------------------------------------------------------------
NC_000004.12:c1 ------------------------------------------------------------


NC_000013.11:c3 AGTCTGCATTTTTGTTGTTGTTGTTGTTTTCTGTATTGTTTTGTTTTGTTTTGAAATGGA
NC_000001.11:33 ------------------------------------------------------------
NC_000023.11:15 ------------------------------------------------------------
NC_000004.12:c1 ------------------------------------------------------------


NC_000013.11:c3 GTCTTTCTCTGTAGCCCAGGCTGGAGTGCAGTGGCACCATCTCAGCTCACTGCAACCTCT
NC_000001.11:33 ------------------------------------------------------------
NC_000023.11:15 ------------------------------------------------------------
NC_000004.12:c1 ------------------------------------------------------------


NC_000013.11:c3 GTCTCCCAGGTTCAAGCGATTCTCATGCCTCAGCCTCCGGAGTAGCTGGGAGTACAGGTG
NC_000001.11:33 ------------------------------------------------------------
NC_000023.11:15 ------------------------------------------------------------
NC_000004.12:c1 ------------------------------------------------------------


NC_000013.11:c3 TGCGCCACCATGCCTGGCTAAGTTTTGTATTTTTAGTAGAGATGCGGTTTCAACATGTTG
NC_000001.11:33 ------------------------------------------------------------
NC_000023.11:15 ------------------------------------------------------------
NC_000004.12:c1 ------------------------------------------------------------


NC_000013.11:c3 GCCAGGCTGGTCTCAAACCCCTGACCTTAAGTTATCCGCCTGCCTCGGCCTCCCAAAGTG
NC_000001.11:33 ------------------------------------------------------------
NC_000023.11:15 ------------------------------------------------------------
NC_000004.12:c1 ------------------------------------------------------------
```

```
NC_000013.11:c3 CTGGGATTACCAGTGTGAGCCACTGTGCCTGGCCTTGCATGTCGTATTTTTTTTACGTCA
NC_000001.11:33 ------------------------------------------------------------
NC_000023.11:15 ------------------------------------------------------------
NC_000004.12:c1 ------------------------------------------------------------

NC_000013.11:c3 GAGTTTTTTATTTGTATCTTTGTTTTGAGACAGAGTCTCACTCTGTGGCACTCTGGTGTA
NC_000001.11:33 ------------------------------------------------------------
NC_000023.11:15 ------------------------------------------------------------
NC_000004.12:c1 ------------------------------------------------------------

NC_000013.11:c3 GTCTCCATTCACTGCAACTTCCGCCCCCTGGGTTCAAGTGATTCTCGTGCTTCAGCCTCC
NC_000001.11:33 ------------------------------------------------------------
NC_000023.11:15 ------------------------------------------------------------
NC_000004.12:c1 ------------------------------------------------------------

NC_000013.11:c3 CAGGTAGCTGGGACTACAGGCATGCGCCATCACACCCGGACTCATTTTGGTCTTTTTAGT
NC_000001.11:33 ------------------------------------------------------------
NC_000023.11:15 ------------------------------------------------------------
NC_000004.12:c1 ------------------------------------------------------------

NC_000013.11:c3 AGAGACAGGGTTTTGCCGTGTTGGCCAGGCTGGTCTTGAACTCCTGGCCTCAAGTGATCC
NC_000001.11:33 ------------------------------------------------------------
NC_000023.11:15 ------------------------------------------------------------
NC_000004.12:c1 ------------------------------------------------------------

NC_000013.11:c3 ACCCACTTCTGCCTCCCAAAGTAGTGGGATTACAGGCATAAGCCACCACACCTGGTCACC
NC_000001.11:33 ------------------------------------------------------------
NC_000023.11:15 ------------------------------------------------------------
NC_000004.12:c1 ------------------------------------------------------------

NC_000013.11:c3 TTTTAAGTTTTAAAAGCCCATTGGTGGCCAGCCGCGGTGGCTCACGCCTGTAATCCCAGC
NC_000001.11:33 ------------------------------------------------------------
NC_000023.11:15 ------------------------------------------------------------
NC_000004.12:c1 ------------------------------------------------------------

NC_000013.11:c3 ACTCTGGGAGGCCTTAGGCGGGCGGATCACCTGAGGTCAGGAGTTCAAGACCAGCCTGGC
NC_000001.11:33 ------------------------------------------------------------
NC_000023.11:15 ------------------------------------------------------------
NC_000004.12:c1 ------------------------------------------------------------

NC_000013.11:c3 CATGGTGAAACCCCATCTCTACTAAAATTACAAAAAATTAGCCAGGCGTGGTGGTGCGTG
NC_000001.11:33 ------------------------------------------------------------
NC_000023.11:15 ------------------------------------------------------------
NC_000004.12:c1 ------------------------------------------------------------

NC_000013.11:c3 CCTGTAATCCCAGCTACTCTGGAGGCTGAGGCAGGAGAATCGCTTGAACCCGGGAGGTGG
NC_000001.11:33 ------------------------------------------------------------
NC_000023.11:15 ------------------------------------------------------------
NC_000004.12:c1 ------------------------------------------------------------

NC_000013.11:c3 ACGTTGCAGTGAGCCGAGATGGTGCCACTGCACTCCAGCCTGGGCAACAAGAGTGAAACT
NC_000001.11:33 ------------------------------------------------------------
NC_000023.11:15 ------------------------------------------------------------
```

```
NC_000004.12:c1 ------------------------------------------------------------


NC_000013.11:c3 CCATCTCAAAAAAAAAAAAAAAAAAAAAAAAAAAAAAGGCCGGGCACGGTGGCTCATGC
NC_000001.11:33 ------------------------------------------------------------
NC_000023.11:15 ------------------------------------------------------------
NC_000004.12:c1 ------------------------------------------------------------


NC_000013.11:c3 CTGTAATCCCAGCACTTTGGGAGGCGGAGGCGGGTGGATCACCTGATGTTAGGAGTTCGA
NC_000001.11:33 ------------------------------------------------------------
NC_000023.11:15 ------------------------------------------------------------
NC_000004.12:c1 ------------------------------------------------------------


NC_000013.11:c3 GACCAGCCTGGCTAACATGGTGAAATCCCGTCTCTACTAAAAATACAAAAAAAAAAAATT
NC_000001.11:33 ------------------------------------------------------------
NC_000023.11:15 ------------------------------------------------------------
NC_000004.12:c1 ------------------------------------------------------------


NC_000013.11:c3 AGCCAGGTGTGGTGGCCCATGCCTATAATCTCTGCTACTTGGGAGGCTGAGGCAGGAGAA
NC_000001.11:33 ------------------------------------------------------------
NC_000023.11:15 -------------------------------ATACCATTTTGAAGGCTGAT---------
NC_000004.12:c1 ------------------------------------------------------------


NC_000013.11:c3 TCACTTGAACCTGGGAGACAGAGGTTGCAGTGAGCCGAGATCGCGCCACTGCACTCCAGC
NC_000001.11:33 ------------------------------------------------------------
NC_000023.11:15 ------------------------------------------------------------
NC_000004.12:c1 ------------------------------------------------------------


NC_000013.11:c3 ATAGGCAACAAGAGTGAAACCCCGTCTCAAAAACAAAAAACAAAAAACAAAAAAACAAAA
NC_000001.11:33 ------------------------------------------------------------
NC_000023.11:15 ------------------------------------------------------------
NC_000004.12:c1 ------------------------------------------------------------


NC_000013.11:c3 AAACCAACCATTGGTTTGGTTGAACATGGGAGACAAACATTAAATTAAAGATAAAATAAA
NC_000001.11:33 ------------------------------------------------------------
NC_000023.11:15 ------------------------------------------------------------
NC_000004.12:c1 ------------------------------------------------------------


NC_000013.11:c3 ATAGGCCAGGCGCGCTGGCTCACACCTGTAATTCCAGCACTTTGGGAGGCCAAGGCAGGT
NC_000001.11:33 -------------------------CTGCAAGTACAGCACTTT-----------------
NC_000023.11:15 -------------------------GTGTATATACATCATTAC-----------------
NC_000004.12:c1 -------------------------CTGTAGAGAAAATACTTT-----------------
                                          **.*     * .*.* .

NC_000013.11:c3 GGATCACGAGGTCAGGAGTTCAAGACCGGCCTGTCCAATATAATGAAACCCTGTCTCTAC
NC_000001.11:33 ------------------------------------------------------------
NC_000023.11:15 ------------------------------------------------------------
NC_000004.12:c1 ------------------------------------------------------------


NC_000013.11:c3 TAAAAATACAAAAATTAGCCATGGCGCGTGCCTGTAGTCCCAGCTACTCAGGAGTCTGAG
NC_000001.11:33 ------------------------------------------------------------
NC_000023.11:15 ----------------------------TGTCCGTAG-----------------------
NC_000004.12:c1 ------------------------------------------------------------


NC_000013.11:c3 GCAGAAGAACTGCTTGAACCTGGGAGGTGGAGGTCGCAGTGAGCTGAGACCACACCACTG
```

```
NC_000001.11:33 ------------------------------------------------------------
NC_000023.11:15 ------------------------------------------------------------
NC_000004.12:c1 ------------------------------------------------------------


NC_000013.11:c3 CACTCCAACCTGGGCAATAGAGCAAGCCTCTGTCTCAAAAAATTAATTAATTAATTAAAT
NC_000001.11:33 ------------------------------------------------------------
NC_000023.11:15 ------------------------------------------------------------
NC_000004.12:c1 ------------------------------------------------------------


NC_000013.11:c3 ATAAATAAATAAATAAATAAATAAGTCTGGGCATGGTGGCTCACACCTGTAATCCCAGCA
NC_000001.11:33 ------------------------------------------------------------
NC_000023.11:15 ------------------------------------------------------------
NC_000004.12:c1 ------------------------------------------------------------


NC_000013.11:c3 CTTTGGAAGGCTGAGGCGGGTGGATCACCTAAGGTCAGGGGTTCAAGACCAGCCTGGCCA
NC_000001.11:33 ------------------------------------------------------------
NC_000023.11:15 ------------------------------------------------------------
NC_000004.12:c1 ------------------------------------------------------------


NC_000013.11:c3 ACATGGTGAAACCCCGTCTCTACTAAAAATACAAAAATTAGTTGGGCATGGTGGCACACG
NC_000001.11:33 ------------------------------------------------------------
NC_000023.11:15 -------------------CAATGAAGGATACAGTACTGTGTTGTG--------------
NC_000004.12:c1 ----------------------TAAAAAATGCAG------GTTGTA--------------


NC_000013.11:c3 CCTGTGATCCTAGCTACTTGGGAGGCTGAGGCAGGAGAATCACTTGAACCCAGGAGGCAG
NC_000001.11:33 ------------------------------------------------------------
NC_000023.11:15 ------------------------------------------------------------
NC_000004.12:c1 ------------------------------------------------------------


NC_000013.11:c3 AGGTTGCAGTGAGCCAAGATCGCGCCACCGCCCTCCAACCTGGACGACTGAGTGAGACTC
NC_000001.11:33 ------------------------------------------------------------
NC_000023.11:15 ------------------------------------------------------------
NC_000004.12:c1 ------------------------------------------------------------


NC_000013.11:c3 CGTCTCAAAAAAAAAATAAAATAAAACAATAAAAAGCCAATTGGTAACAATCTTTGGGCA
NC_000001.11:33 ------------------------------------------------------------
NC_000023.11:15 ------------------------------------------------------------
NC_000004.12:c1 ------------------------------------------------------------


NC_000013.11:c3 GACAATCTTGCAGTTTTCTGTGATAAAGTCTAATTTGGCTTGCCATAGTTCACTGTATCC
NC_000001.11:33 ------------------------------------------------------------
NC_000023.11:15 ------------------------------------------------------------
NC_000004.12:c1 ------------------------------------------------------------


NC_000013.11:c3 ACCAAGGAATAATCTTGGCCTAATTGGAAACTGGTAAATGCTTAGACTATATGTGTTTCT
NC_000001.11:33 ------------------------------------------------------------
NC_000023.11:15 --------------------------------GGTGAGTGTT------------------
NC_000004.12:c1 ------------------------------------------------------------


NC_000013.11:c3 CTACATTTGAGATCCCCTCTCACCCTCCAACTGTGCCTCTCCCTTTTTAAGTTACGTCAA
NC_000001.11:33 ------------------------------------------------------------
NC_000023.11:15 ------------------------------------------------------------
NC_000004.12:c1 ------------------------------------------------------------
```

```
NC_000013.11:c3 TGAGATGTAATTGGCACCTATCCTCAATCTTAGTATCCTAACATTCGCTTCTTATTCTAT
NC_000001.11:33 ------------------------------------------------------------
NC_000023.11:15 ------------------------------------------------------------
NC_000004.12:c1 ------------------------------------------------------------

NC_000013.11:c3 TTTCCTGCCTTTTGATTTAGAAAGACCTAAATGCTTTTAGATCTAAAGATAGAAGAGGAA
NC_000001.11:33 ------------------------------------------------------------
NC_000023.11:15 ------------------------------------------------------------
NC_000004.12:c1 ------------------------------------------------------------

NC_000013.11:c3 GGTTAAAACAATAATTTGCAATACGTACTCCTTCAGCTATTAATTCATTTATGCAACGGG
NC_000001.11:33 ------------------------------------------------------------
NC_000023.11:15 -----------------------------------GCTATTG------------------
NC_000004.12:c1 -----------------------------------GCTTTTT------------------

NC_000013.11:c3 CTTTATTTAATGCCTGCTGTATGCCAAGTACTCTGCTAGGTGCTTGGGATTCAAAGATAG
NC_000001.11:33 ------------------------------------------------------------
NC_000023.11:15 ------------------------------------------------------------
NC_000004.12:c1 ------------------------------------------------------------

NC_000013.11:c3 GGGCTGGGCTCAGTTTCTCATGCCTGTAATCCCAACATTTTGGGAGGCCAAGGAGTTTGA
NC_000001.11:33 ------------------------------------------------------------
NC_000023.11:15 ------------------------------------------------------------
NC_000004.12:c1 ------------------------------------------------------------

NC_000013.11:c3 GACCAGCCTGAGCAACATGGAGAGACCCTGCCTCTACAAAAAAAAAAATAGCCAGCACCA
NC_000001.11:33 ------------------------------------------------------------
NC_000023.11:15 ------------------------------------------------------------
NC_000004.12:c1 ------------------------------------------------------------

NC_000013.11:c3 GGCACGGTGGTTCATGCCTGTCATCCCAGCACTTTGGGAGGCCAAGGTAGGAGGATCACT
NC_000001.11:33 ------------------------------------------------------------
NC_000023.11:15 ------------------------CCCAGCATT---------------------AATATT
NC_000004.12:c1 ------------------------------------------------------------

NC_000013.11:c3 TGAGCCCATGAGTTTGAGACCAGCCTGGGCAACATGGAGAGACCTTGTCTCTACAAAAAA
NC_000001.11:33 ------------------------------------------------------------
NC_000023.11:15 TGGGTGTGTATGTTTGAG------------------------------------------
NC_000004.12:c1 ------------------------------------------------------------

NC_000013.11:c3 TAAAAATAAAAATAAATTAGCTGGGGCTGGGCACAGTGGCTCATGCCTGTAATCCCAGCA
NC_000001.11:33 ------------------------------------------------------------
NC_000023.11:15 ------------------------------------------------------------
NC_000004.12:c1 ------------------------------------------------------------

NC_000013.11:c3 CTTTGGGAGGCCAAGGCGGGCGGATCACAAGGTCAAGAGATGGAGACCATCCTGGCCAAC
NC_000001.11:33 ------------------------------------------------------------
NC_000023.11:15 ------------------------------------------------------------
NC_000004.12:c1 ------------------------------------------------------------

NC_000013.11:c3 ATGGTGAAACCCCGTCTCCACTAAAAATACAAAAATAAGCTGGGCGTGGTGGCACGCGCC
NC_000001.11:33 ------------------------------------------------------------
NC_000023.11:15 ------------------------------------------------------------
```

```
NC_000004.12:c1 ------------------------------------------------------------

NC_000013.11:c3 TGTAGTCACAGCTGTTCGGGAGGCTGAGGCAGGGGAATCACTTAAACCAAGGACGCAGAG
NC_000001.11:33 ------------------------------------------------------------
NC_000023.11:15 ------------------------------------------------------------
NC_000004.12:c1 ------------------------------------------------------------

NC_000013.11:c3 GTTGCAGTGATCCAAGATTGTGCCACTGCACTCCAGCCTGGTGACAGAGGGAGACTCCGT
NC_000001.11:33 ------------------------------------------------------------
NC_000023.11:15 ------------------------------------------------------------
NC_000004.12:c1 ------------------------------------------------------------

NC_000013.11:c3 CTCAAATAAATAAATAAATAGGCCGGGCGTGGTGGCACTCACCTGTAGTCCTGGCTTCCC
NC_000001.11:33 ------------------------------------------------------------
NC_000023.11:15 ------------------------------------------------------------
NC_000004.12:c1 ------------------------------------------------------------

NC_000013.11:c3 AGGAAGCTGAGGTGGGAGGATGGCTTCAGTCCCAGAAAGTCAAGGCTGCAGTGAGCCGTG
NC_000001.11:33 ------------------------------------------------------------
NC_000023.11:15 ------------------------------------------------------------
NC_000004.12:c1 ------------------------------------------------------------

NC_000013.11:c3 ATCATGCCACTGCACTGCACTCCAGCCTGGGCAATACAGCCATAACCTGTCTTAGAAAAA
NC_000001.11:33 ------------------------------------------------------------
NC_000023.11:15 ------------------------------------------------------------
NC_000004.12:c1 ------------------------------------------------------------

NC_000013.11:c3 AAAAAACAAAAAATACAGAGTTCTTGCCTTTAAGGTGTACAGTGCAGTGGGACAAGCAGA
NC_000001.11:33 ------------------------------------------------------------
NC_000023.11:15 -------------------------------------------GCTATGAAACACGCAGG
NC_000004.12:c1 ---------------------------------------------GATGGGCTACTCATA

NC_000013.11:c3 TACAACTGGAGAACCACAATTGTGATTGCAGTAATTAATATACACAATATTACAGGATTA
NC_000001.11:33 ------------------------------------------------------------
NC_000023.11:15 ------------------------------------------------------------
NC_000004.12:c1 ------------------------------------------------------------

NC_000013.11:c3 CAGAAAAGAGGTACGTCCCCTATCCTAGAGGGGATGGAGGAAGGGCATAAAGTACATTAA
NC_000001.11:33 ------------------------------------------------------------
NC_000023.11:15 ------------------------------------------------------------
NC_000004.12:c1 ------------------------------------------------------------

NC_000013.11:c3 TGACGATCTGTAATTCAGTTGTTTAAATTGAGCATGGGAGAGGGTGTTCAGGCCTCTTCG
NC_000001.11:33 ------------------------------------------------------------
NC_000023.11:15 ------------------------------------------------------------
NC_000004.12:c1 ------------------------------------------------------------

NC_000013.11:c3 ACTGGATGACTTTGTAATTTGGGATAAAACACCCAATAACTTGGCCGGGTACCAGGGCTC
NC_000001.11:33 ------------------------------------------------------------
NC_000023.11:15 ------------------------------------------------------------
NC_000004.12:c1 ------------------------------------------------------------

NC_000013.11:c3 ATGCCTGTAATCTTAGCACTTTGGGAGGCTGAGGCGGGCAGATCACTTGAGGTCAGGAGT
```

```
NC_000001.11:33 ------------------------------------------------------------
NC_000023.11:15 ------------------------------------------------------------
NC_000004.12:c1 ------------------------------------------------------------

NC_000013.11:c3 TCAAGACCAGCCTGACCAACATGGTGAAACCGTCTCTATCAAAAATTCAAAATTACCCAG
NC_000001.11:33 ------------------------------------------------------------
NC_000023.11:15 ------------------------------------------------------------
NC_000004.12:c1 ------------------------------------------------------------

NC_000013.11:c3 GCATGGTGGTGCATGCCTGTAGTCCCAGCTACTCGGGAGGCTGAGGCACGAGAATTGCTT
NC_000001.11:33 ------------------------------------------------------------
NC_000023.11:15 ------------------------------------------------------------
NC_000004.12:c1 ------------------------------------------------------------

NC_000013.11:c3 GAACCCGGGAGGTGGAGGTTGCAGTGAGCTGAGATCGTGCCATTCCACTCCAGCCTGGGC
NC_000001.11:33 ------------------------------------------------------------
NC_000023.11:15 ------------------------------------------------------------
NC_000004.12:c1 ------------------------------------------------------------

NC_000013.11:c3 AACAGAGCAAGACCCTGTCTCATAAAAACAAAAACAACAACAACAAAAACCCAATAACTG
NC_000001.11:33 ------------------------------------------------------------
NC_000023.11:15 ------------------------------------------------------------
NC_000004.12:c1 ------------------------------------------------------------

NC_000013.11:c3 TAAAGTAAAATATGTCAACTTGTGACTAAGGATTCATGTGGAAGAAGAAAAGCGTCTAAT
NC_000001.11:33 ------------------------------------------------------------
NC_000023.11:15 --------------------------------------------------AGTGTTT---
NC_000004.12:c1 ------------------------------------------------------------

NC_000013.11:c3 ATCATAAAGGAACATCAAGATTGTGGGGTTAGAGCTAGAAGATGTTGGTTTTATTTTAAA
NC_000001.11:33 ------------------------------------------------------------
NC_000023.11:15 --------------------TTGTGCTATTAATTTTAAGAGAAAGCAGCTTTTTCTTAAA
NC_000004.12:c1 ---------------------------------------------CAGTTAGATTTTACA

NC_000013.11:c3 ATTTCTGTCTTTTTTATGTTCTTCCCTGCCTATATCACAGAAAATCAAATATAAGAAATG
NC_000001.11:33 ------------------------------------------------------------
NC_000023.11:15 ATTCACT-----------------------------------------GTTGAGAAACTT
NC_000004.12:c1 GCTTCTG-----------------------------------------------------

NC_000013.11:c3 AATTGCCTGCAAGTGGTGGTTTTTAAACTCTTCTCTCTGATATAGTCTCTGTTAAACTTG
NC_000001.11:33 ------------------------------------------------------------
NC_000023.11:15 GCATGTCTGGAGGCGGT-------------------------------------------
NC_000004.12:c1 ------------------------------------------------------------

NC_000013.11:c3 GGAATAAATAAAGGTACCATCACCAGACTAGGGGAAGATTAGCATTGTTTGAGGTTAGGA
NC_000001.11:33 ------------------------------------------------------------
NC_000023.11:15 ------------------------------------------------------------
NC_000004.12:c1 ------------------------------------------------------------

NC_000013.11:c3 TTTAAAAAAAACAAAACAAGGCCGGGTGCAGTGGCTCATGCCTGTAATCCCAGCACTTTG
NC_000001.11:33 ------------------------------------------------------------
NC_000023.11:15 ------------------------------------------------------------
NC_000004.12:c1 ------------------------------------------------------------
```

```
NC_000013.11:c3 GGAGGCCAAGGTGGGCGGATCACCTGAGGTCAAGAGTTCAAGACCAGCCTGACCAACATG
NC_000001.11:33 ------------------------------------------------------------
NC_000023.11:15 ------------------------------------------------------------
NC_000004.12:c1 ------------------------------------------------------------

NC_000013.11:c3 GTGAAACCCCGTCTCTACTAAAAATAAAAAATTAGCCGGGCATGGTGGCGCATGCCTGTA
NC_000001.11:33 ------------------------------------------------------------
NC_000023.11:15 ------------------------------------------------------------
NC_000004.12:c1 ------------------------------------------------------------

NC_000013.11:c3 TTCCCAGCTACTCGGGAGGCTGAGGCAGAAGAATCGATTGAACCAGGAGGTGTAGGTTGC
NC_000001.11:33 ------------------------------------------------------------
NC_000023.11:15 ------------------------------------------------------------
NC_000004.12:c1 ------------------------------------------------------------

NC_000013.11:c3 AGTGAGCCGAGATTGCACCGCTGCACTCCAGCCTGGGCAGTAAGAGCAAAACTCTGTCTC
NC_000001.11:33 ------------------------------------------------------------
NC_000023.11:15 ------------------------------------------------------------
NC_000004.12:c1 ------------------------------------------------------------

NC_000013.11:c3 AAACAACAACAATAACAACAACAACAAAAAATTCACTAGATTTGCAATCACTGCCTTCAT
NC_000001.11:33 ------------------------------------------------------------
NC_000023.11:15 ------------------------------------------------------------
NC_000004.12:c1 ------------------------------------------------------------

NC_000013.11:c3 ATAAGATTAAAATATGAAGAAAAACTCAAGGTTAACTGCACTTGAGGTTTTCTAAATTAT
NC_000001.11:33 ------------------------------------------------------------
NC_000023.11:15 ------------------------------------------------------------
NC_000004.12:c1 ------------------------------------------------------------

NC_000013.11:c3 GGGATTGAAGGGGTCTATATAATTTTAAACATTAAAAAAAGTTTAATCTTTACAACCCTG
NC_000001.11:33 ------------------------------------------------------------
NC_000023.11:15 ------------------------------------------------------------
NC_000004.12:c1 ------------------------------------------------------------

NC_000013.11:c3 TGGTCCCCTCCATTTTATAGCTGAGAGAATTGAAGCATGGAGGTTTAAAAACATGCCCAA
NC_000001.11:33 ------------------------------------------------------------
NC_000023.11:15 --GTCCTCTCC-------------------------------------------------
NC_000004.12:c1 ------------------------------------------------------------

NC_000013.11:c3 GATCATACAGCTAGTAAGTAGCAGAGCTGGGATTTGCACCCAAGTCTATTCAATTCCAGT
NC_000001.11:33 ------------------------------------------------------------
NC_000023.11:15 ------------------------------------------------------------
NC_000004.12:c1 ------------------------------------------------------------

NC_000013.11:c3 GCCTATTACTCACCATATCATCCTGTCAAAATAGAAACCTTGTGTGGGCCCATTGCTCTC
NC_000001.11:33 ------------------------------------------------------------
NC_000023.11:15 ------------------------------------------------------------
NC_000004.12:c1 ------------------------------------------------------------

NC_000013.11:c3 AAGATGTCCTGTGGCCATAAATGCACCCTGGCAGGAGCAAGCTGGTATAGACAACCAGTA
NC_000001.11:33 ------------------------------------------------------------
NC_000023.11:15 ------------------------------------------------------------
```

```
NC_000004.12:c1 ------------------------------------------------------------


NC_000013.11:c3 AAGTGTATGGCCGTGCCAGTACAAAGCCAACACTGCTCCTGGAAAATATTGATAGTGCCA
NC_000001.11:33 ---------------------------CTAGATTGCTCCTGGA-----------------
NC_000023.11:15 -------------------------GCCCTGTCGGGTCCTGGA-----------------
NC_000004.12:c1 -------------------------ATGTTGAATGTTCCTAAA-----------------
                                                  * ****..*

NC_000013.11:c3 TATTCAACTAATCGTCAAATTTTAATATCATCGATGCCACTGAAAGTATCTTAATCATTC
NC_000001.11:33 ------------------------------------------------------------
NC_000023.11:15 ------------------------------------------------------------
NC_000004.12:c1 ------------------------------------------------------------


NC_000013.11:c3 TCATGAATATTTTGTAATATGAGACATTGAGAAGATAAGACAGTATTGAAATAAATATCT
NC_000001.11:33 ------------------------------------------------------------
NC_000023.11:15 ------------------------------------------------------------
NC_000004.12:c1 ------------------------------------------------------------


NC_000013.11:c3 AGATTTAAAAAATATAAAATTTGAATGATTAGTAGAGGGAAGCAGAGGATAGTAAATATG
NC_000001.11:33 -----------------------------------------------------------G
NC_000023.11:15 ----------------------------------------------------------TG
NC_000004.12:c1 ------------------------------------------------------------


NC_000013.11:c3 TGTGAAAATAATAACATAAAAGTTAACTTTAGATATATTATCTTTAAACACTGCATGTGC
NC_000001.11:33 TGTGGGAACAACAG----------------------------------------------
NC_000023.11:15 AGTACGAGTTATGG----------------------------------------------
NC_000004.12:c1 ------------------------------------------------------------


NC_000013.11:c3 AGTATACCAATTTATATCCTCTTTAATATTATAGTCTTTTTCATGTGCATGTGTGATATA
NC_000001.11:33 ------------------------------------TCTCTCCTGTCCACGT--------
NC_000023.11:15 -----------------TCACGGTCACAGCCTGATCTCTTATGTGTTCATAG--------
NC_000004.12:c1 -------------------TATTTAATGGTTTTTTTAATTTCTTGTGTATGG--------
                                                      *. . *** .*..

NC_000013.11:c3 TTGTCCATTCACCAAGACTATAATCCCTGAGTCCGATAGTGTATTTTCTTTTAAAAAGAA
NC_000001.11:33 ------------------------------------------------------------
NC_000023.11:15 ----CCATTCGCT-----------------------------------------------
NC_000004.12:c1 ------------------------------------------------------------


NC_000013.11:c3 ATCAACATTAAAACAGGAAAGATTATCTAAGCTTACTCTCAAGTAGTTTAATACATTTGA
NC_000001.11:33 ------------------------------------------------------------
NC_000023.11:15 -----------------------------------CTCCCA-------------------
NC_000004.12:c1 ------------------------------------------------------------


NC_000013.11:c3 GGAAATAAATTAGGTTATGGATGCTTATAAGCGATATAAAAAATCAAATTAACACGATGT
NC_000001.11:33 ------------------------------------------------------------
NC_000023.11:15 ------------------------------------------------------------
NC_000004.12:c1 ------------------------------------------------------------


NC_000013.11:c3 AGGTGATTTATTCCAAATTTGATCTGTTACATTTGGAGGAATTGGTTGTTGGATTTAGAA
NC_000001.11:33 ------------------------------------------------------------
NC_000023.11:15 ------------------------------------------------------------
NC_000004.12:c1 ------------------------------------------------------------


NC_000013.11:c3 GTGTACAAAATACCATTAGAGAGTGAAAAAAAAGGGAAGTCTGTTATAACTGAATAGGGG
```

```
NC_000001.11:33 ------------------------------------------------------------
NC_000023.11:15 ------------------------------------------------------------
NC_000004.12:c1 ------------------------------------------------------------

NC_000013.11:c3 CGTGGTCTGCTCAGGCTAAAAGGAATAGATAGTAGTTTCATAAAGGACTGATATAACTGT
NC_000001.11:33 ------------------------------------------------------------
NC_000023.11:15 ------------------------------------------------------------
NC_000004.12:c1 ------------------------------------------------------------

NC_000013.11:c3 AGAGTCTTCAGAACTGGCCACTACAGCCAGCACATTTTCTCTACAGACGTAAGTATGTCT
NC_000001.11:33 ------------------------------------------------------------
NC_000023.11:15 -------TCAGAACTGTTT-----------------------------------------
NC_000004.12:c1 ------------------------------------------------------------

NC_000013.11:c3 ATTAATACCAGCCAGCTTGGTGAGTTAGAGAAACCACAGCCTGTGGATTTGGGCACATGC
NC_000001.11:33 ------------------------------------------------------------
NC_000023.11:15 ------------------------------------------------------------
NC_000004.12:c1 ------------------------------------------------------------

NC_000013.11:c3 ACTCTCTGCCTGCATCTAATCCTTCGGGTAACCATATACCTACAACTCCTGTAATCCTAG
NC_000001.11:33 ------------------------------------------------------------
NC_000023.11:15 ------------------------------------------------------------
NC_000004.12:c1 ------------------------------------------------------------

NC_000013.11:c3 AGCAGTTACATGGATGCACTAAGTGGTGTGATGGGAGGGAAAGATAGCATCACAAGGATT
NC_000001.11:33 ------------------------------------------------------------
NC_000023.11:15 ------------------------------------------------------------
NC_000004.12:c1 ------------------------------------------------------------

NC_000013.11:c3 CGTTTGATTGTAACAGTAATCCACACCAAGTCAAATAAAATAGCCAAACAGTTTAGAATG
NC_000001.11:33 ------------------------------------------------------------
NC_000023.11:15 --------------------------------------------------GTCCTGAATG
NC_000004.12:c1 ------------------------------------------------------------

NC_000013.11:c3 TGGTCTCTAATGAATTTGCTAATTCTACCAGGGCCTCACAAACATTTCCTGCTAGAGTAC
NC_000001.11:33 ------------------------------------------------------------
NC_000023.11:15 TG----------------------------------------------------------
NC_000004.12:c1 ------------------------------------------------------------

NC_000013.11:c3 CCCATATCAAACCAAGTGGCTGTTCTTTAATTACATGGAATAATTATTTGATTTCCAGGA
NC_000001.11:33 ------------------------------------------------------------
NC_000023.11:15 ------------------------------------------------------------
NC_000004.12:c1 ------------------------------------------------------------

NC_000013.11:c3 AGTTAAGACAGTGCTTTGTATTTAAATAAAAAGTTCTAGACATGCTGTGAAACTCGATCA
NC_000001.11:33 -------------------------------------------------------TACTG
NC_000023.11:15 ----------------------------------------------------TTCCTCTA
NC_000004.12:c1 ------------------------------------------------------------

NC_000013.11:c3 AACCAAGAAAAAGGGTGAAACTGAATGCCTGCTCACTGACTCAAGATTTCAGCTGCTCAG
NC_000001.11:33 AATCCAGAAAAAAGGTGAA-----------------------------------------
NC_000023.11:15 GTTCTAGAAAA------------------TGACCACTAATTTAAA---------------
NC_000004.12:c1 ------------------------------------------------------------
```

```
NC_000013.11:c3 CGAGCCTGACGTGACGGCTTATGAGCCCAGAAAAGGGGTATTTTAAACCCGGCTGGCTGG
NC_000001.11:33 -----------------CTTACGAACATGGGAAAAG--------AAATCCAGCT------
NC_000023.11:15 --------------------------------------------AAACTCGGTTGT----
NC_000004.12:c1 --------------------------------------------TAGCACAGCA------
                                                             *.. *.*.

NC_000013.11:c3 ATCATGTCACTTTCTTATAGAAGCCCTGCCTTTCCAGAGACCTAAAGTGTAACGCAATCA
NC_000001.11:33 ------------------------------------------------------------
NC_000023.11:15 ------------------------------------------------------------
NC_000004.12:c1 ------------------------------------------------------------


NC_000013.11:c3 GAGACCTTTGAGGGGCTCGAGGCTCGGCGGCTTCCGCCCCTCAGCACCAGCAGAGACCCC
NC_000001.11:33 ------------------------------------------------------------
NC_000023.11:15 ------------------------------------------------------------
NC_000004.12:c1 ------------------------------------------------------------


NC_000013.11:c3 AGTTTTCAGGGGACATGATCCCATAGTGTCGCCCTCACTTTTGAAGGGCCATTAAAAGCC
NC_000001.11:33 ------------------------------------------------------AAAGCC
NC_000023.11:15 ------------------------------------------------------GAGGTT
NC_000004.12:c1 ------------------------------------------------------------


NC_000013.11:c3 TGGGGCCTCTTATCTCAACGGCTTTGGGCGTGAATGTGGGGCAAGAAGGGGGGGGGAGAC
NC_000001.11:33 T-----------------------------------------------------------
NC_000023.11:15 TG----------------------------------------------------------
NC_000004.12:c1 ------------------------------------------------------------


NC_000013.11:c3 CTGTGGGTGTCTCTTTTGCCTGAGGAGTTGGAGACACTTGTGGAAAAGTCAGGCCCTTTT
NC_000001.11:33 ------------------------------------------------------------
NC_000023.11:15 ------------------------------------------------------------
NC_000004.12:c1 ------------------------------------------------------------


NC_000013.11:c3 CGCTCCGGCGGCCGCTCCGGTGTGGGGCTGGCTTGGGTTAGACACATGCACACATACACC
NC_000001.11:33 ------------------------------------------------------------
NC_000023.11:15 ------------------------------------------------------------
NC_000004.12:c1 ------------------------------------------------------------


NC_000013.11:c3 ATAGAGCTCTGCTTTCCCGTAGCAGCTGCTGCCTCTGCCTCTGCCTCTCCCGCCTCAGCC
NC_000001.11:33 ------------------------------------------------------------
NC_000023.11:15 ------------------------------------------------------------
NC_000004.12:c1 ------------------------------------------------------------


NC_000013.11:c3 TCTTTGCCCGGCATACACACACATTCAGATTTGCGCGCTGTTTCAATCCTTGATGACGTG
NC_000001.11:33 ------------------------------------------------------------
NC_000023.11:15 ------------------------------------------------------------
NC_000004.12:c1 ------------------------------------------------------------


NC_000013.11:c3 TCCCCGGAGACAGCCAATAGCAAACGGGCTCTGGTCAGGACAATGGGAGGTATCGGGCCA
NC_000001.11:33 ------------------------------------------------------------
NC_000023.11:15 ------------------------------------------------------------
NC_000004.12:c1 ------------------------------------------------------------


NC_000013.11:c3 ATGAGCGAGCCCCGTGAGTTGGCGGTAGCCAATAGGAGCCGCGCTGGCTGGAGAGTAATG
NC_000001.11:33 ------------------------------------------------------------
NC_000023.11:15 ------------------------------------------------------------
```

NC_000004.12:c1 --

NC_000013.11:c3 TTACAGAGCGGAGAGAGTGAGGAGGCTGCGTCTGGCTCCCGCTCTCACAGCCATTGCAGT
NC_000001.11:33 --
NC_000023.11:15 --
NC_000004.12:c1 --

NC_000013.11:c3 ACATTGAGCTCCATAGAGACAGCACCGGGGCAAGTGAGAGCCGGACGGGCACTGGGCGAC
NC_000001.11:33 --
NC_000023.11:15 --
NC_000004.12:c1 --

NC_000013.11:c3 TCTGTGCCTCGCTGAGGGTGAGTCTGGGGCAGCGCCGCGGCGGGGAGAGCGCCTCCGGCA
NC_000001.11:33 --
NC_000023.11:15 --
NC_000004.12:c1 --

NC_000013.11:c3 GCTCCCCAGCCCGCGCGGCGGCCGGATCCCCGCGGCCGGGAGCCGGCGGGTCAGGATCCA
NC_000001.11:33 --
NC_000023.11:15 --
NC_000004.12:c1 --

NC_000013.11:c3 CACAAAGGCAAATGAGGGGGGACCGTGGGGGGAACTGCGCACGGAGCGAGCCTCTGCCCG
NC_000001.11:33 ----AAGGCAAATG--
NC_000023.11:15 CCCAGAGGCACTTG--
NC_000004.12:c1 --

NC_000013.11:c3 GGCGCCGGGAACGCTGCCCCGCGCCGGTGCCCCGGCCCTCAGGCAGCCTGAGGCGCCGGG
NC_000001.11:33 --
NC_000023.11:15 --
NC_000004.12:c1 --

NC_000013.11:c3 AGCCCCGCGCCCCGCGAGTTTCCACCCCCGGCGGCGTCCGCGCTGACTGGCGCAAAAAAA
NC_000001.11:33 --
NC_000023.11:15 ----------TTCCAGAATTTCC-------------------------------------
NC_000004.12:c1 --

NC_000013.11:c3 AAAATTTTTTTTTTAATTAAAAAAATTTTGAACGTGTTTTGGGCCCTCGGGCCGGGCGTT
NC_000001.11:33 --
NC_000023.11:15 --
NC_000004.12:c1 --

NC_000013.11:c3 CGGGCGGGCGGCGTGCGCGGAGCGCGGCCGGGGCGGCGGGGCCGGCGCGGCTCGGCGGCG
NC_000001.11:33 --
NC_000023.11:15 --
NC_000004.12:c1 --

NC_000013.11:c3 GCGGCGGGAGGGCAGCGGCGGCGCTTCCCCGGGCTGCATTGGCCGCCGCCGCAGCGAGCC
NC_000001.11:33 --
NC_000023.11:15 --
NC_000004.12:c1 --

NC_000013.11:c3 GGGCGCTGGCGGGGAGCGCGGCCAGCCGGGCGGGCGGCGGGGCGGGCGGGGCGCCGCGGC

```
NC_000001.11:33 ------------------------------------------------------------
NC_000023.11:15 ------------------------------------------------------------
NC_000004.12:c1 ------------------------------------------------------------


NC_000013.11:c3 GGGCGAGGGCGGCGCGGGGGCCTGGGGGCGGCAGTGCGGGCCCGGCCGGCCTCGGCCCGG
NC_000001.11:33 ------------------------------------------------------------
NC_000023.11:15 ------------------------------------------------------------
NC_000004.12:c1 ------------------------------------------------------------


NC_000013.11:c3 TCGCGGCGGCGGCGGCGGCCGGGCGGCGGGGGGAGCGGCGCCGCTGCGCTCGCTGGAACA
NC_000001.11:33 ------------------------------------------------------------
NC_000023.11:15 ------------------------------------------------------------
NC_000004.12:c1 ------------------------------------------------------------


NC_000013.11:c3 TGGCTGACTCGGGCCCGGCGCTGCTGGCTGGAGAGAAAACAAGGCGGGCGGGCGGGGGAG
NC_000001.11:33 ------------------------------------------------------------
NC_000023.11:15 ------------------------------------------------------------
NC_000004.12:c1 ------------------------------------------------------------


NC_000013.11:c3 CTGGGCGCAGCAGTTCCGAGGCAACTTTTTTTTTCCTCTCTCTTTTCACAGCCCCGCGTT
NC_000001.11:33 --------------------------------------TCTCTTCTTACGTTCAC-----
NC_000023.11:15 --------------------------------------CCTCCTGCTTCAGCCAT-----
NC_000004.12:c1 ------------------------------------------------------------


NC_000013.11:c3 CTCCGTGCGGGGGCGGCGGGGCGCGCGGCCCGCGCGGAGGGAGACGGGGAGTGGCGGGCG
NC_000001.11:33 ------------------------------------------------------------
NC_000023.11:15 ------------------------------------------------------------
NC_000004.12:c1 ------------------------------------------------------------


NC_000013.11:c3 GGTGGGCCCTCGGGCGGCCCCCACCGCGGCGGGGGGAGGGGAGCGGCGCGGAGGGGAGGG
NC_000001.11:33 ------------------------------------------------------------
NC_000023.11:15 ------------------------------------------------------------
NC_000004.12:c1 ------------------------------------------------------------


NC_000013.11:c3 CCCGGCCGCGCGCCCCCCGCCCCTCCCCCGGCCGTAATGGCCGAGTGTGTGCGCCAGAGC
NC_000001.11:33 ------------------------------------------------------------
NC_000023.11:15 ------------------------------------------------------------
NC_000004.12:c1 ------------------------------------------------------------


NC_000013.11:c3 GCGGCTCGCACCCCGCCCGCCGCCGCCGCCGCGCCCGCACCCTCGCACTCACACACTCTC
NC_000001.11:33 ------------------------------------------------------------
NC_000023.11:15 ------------------------------------------------------------
NC_000004.12:c1 ------------------------------------------------------------


NC_000013.11:c3 TCATACACACACACACACACACACACACACACAAAGGGAAGGAGCCATATTCTCGCTCGCGC
NC_000001.11:33 ------------------------------------------------------------
NC_000023.11:15 ------------------------------------------------------------
NC_000004.12:c1 ------------------------------------------------------------


NC_000013.11:c3 TCGCCCTCGCGGCGGCGGCGGCGCAGGCGGAGAAGACGCGCAGCGGCCATTCCGTGCGCG
NC_000001.11:33 ------------------------------------------------------------
NC_000023.11:15 ------------------------------------------------------------
NC_000004.12:c1 ------------------------------------------------------------
```

```
NC_000013.11:c3 CCGGCCCCGGCGGCCGCGGGCGGAGCCAGCCCCCATTTCGAGCGGGGCTTCTCCCTGCGC
NC_000001.11:33 ------------------------------------------------------------
NC_000023.11:15 ------------------------------------------------------------
NC_000004.12:c1 ------------------------------------------------------------

NC_000013.11:c3 CGAGCCTGACAAAATGGGGGCGGCGGCGGCGCGGGCCTGCAGGGCCTGCCGGGCGCACGT
NC_000001.11:33 ------------------------------------------------------------
NC_000023.11:15 ------------------------------------------------------------
NC_000004.12:c1 ------------------------------------------------------------

NC_000013.11:c3 GGCGGCCTCGGGCCTGGGAGCCGGGCCGCGTCCTCTCTCCTCGGCCGCGCGGCCACCGGC
NC_000001.11:33 ------------------------------------------------------------
NC_000023.11:15 ------------------------------------------------------------
NC_000004.12:c1 ------------------------------------------------------------

NC_000013.11:c3 GAAGTTCTAGGGGCGGGGGGCTCGCCCCGCGCAGGAGTCACCCCAACTTTCACGGCTCCA
NC_000001.11:33 ------------------------------------------------------------
NC_000023.11:15 ------------------------------------------------------------
NC_000004.12:c1 ------------------------------------------------------------

NC_000013.11:c3 AAAAATACTTCCCGAGTTGGGGGAGGGGGCCACCGAGCCACGAGCAGGAGTGGCTTTTGT
NC_000001.11:33 ------------------------------------------------------------
NC_000023.11:15 ----------------------------------------------------GTCCTTGT
NC_000004.12:c1 ------------------------------------------------------------

NC_000013.11:c3 CCCTCATCCTTGTTTACTCGGAGAAACTTCAGACCGGACGTGTTTAGTCAGAACAGAAAT
NC_000001.11:33 ------------------------------------------------------------
NC_000023.11:15 CACTTGGCAT--------------------------------------------------
NC_000004.12:c1 ------------------------------------------------------------

NC_000013.11:c3 ACATCTCAGGGCCAAACCGATAGGAAACGAGGCTGCCTCGCGGTGGCACCGCCACCCCCC
NC_000001.11:33 ------------------------------------------------------------
NC_000023.11:15 ------------------------------------------------------------
NC_000004.12:c1 ------------------------------------------------------------

NC_000013.11:c3 AACCGGGTTCCGAGCACCGGAGCTGGCTGCTGCTCCCTCTTTGGAGCAAAGTTTTATGCA
NC_000001.11:33 ------------------------------------------------------------
NC_000023.11:15 ------------------------------------------------------------
NC_000004.12:c1 ------------------------------------------------------------

NC_000013.11:c3 AAGAGGGTGTTTTTTGAAACTTTCGGTGCACGGTGATTTTTTTTTTTTAAGGTCCCATAA
NC_000001.11:33 ------------------------------------------------------------
NC_000023.11:15 ------------------------------------------------------------
NC_000004.12:c1 ------------------------------------------------------------

NC_000013.11:c3 TTAGGAAGAGTCGACTCGCTTAGGCCCTTGTTTATTCCCTATCTAGTGCAAAGCCACGAA
NC_000001.11:33 ------------------------------------------------------------
NC_000023.11:15 ------------------------------------------------------------
NC_000004.12:c1 ------------------------------------------------------------

NC_000013.11:c3 TTGGCAGCATGTTTTCTGACCTTTGGTTTGGTTGGTTTAAAATGGTGTTCTAGATTTTAA
NC_000001.11:33 ------------------------------------------------------------
NC_000023.11:15 -----------------------------------TCTAAGCTAAAGCTTTAGCTTCCCA
```

```
NC_000004.12:c1 ------------------------------------------------------------


NC_000013.11:c3 AATCGTTTAAGTGACCAGTTAGATACTCATTCAGAGCAGACTCGGGCGGATAGATAGGGA
NC_000001.11:33 ------------------------------------------------------------
NC_000023.11:15 ATTCGT------------------------------------------------------
NC_000004.12:c1 ------------------------------------------------------------


NC_000013.11:c3 ATACTGTATGGGTATATCTTTGTGTCTAGACTTTTTGAGATCGCCCTGAAGGACTGTTTT
NC_000001.11:33 ------------------------------------------------------------
NC_000023.11:15 ------------------------------------------------------------
NC_000004.12:c1 ------------------------------------------------------------


NC_000013.11:c3 TGTTTTGTTTTGTTTGCTTGGCATAGCCCCTTCAAGGAATTTAATCTCTCGGCCATATTC
NC_000001.11:33 ------------------------------------------------------------
NC_000023.11:15 ------------------------------------------------------------
NC_000004.12:c1 ------------------------------------------------------------


NC_000013.11:c3 TTGTCTGATTTTACGGAGGTTGATGTCGCTACTGTGTTAAATAACCAGTACTTTGGTTTT
NC_000001.11:33 ------------------------------------------------------------
NC_000023.11:15 ------------------------------------------------------------
NC_000004.12:c1 ------------------------------------------------------------


NC_000013.11:c3 CATTCCCTTACTAAGTACTTTAAGGTCTTATATGTCATAATTTTATTGCTAACATCAAAT
NC_000001.11:33 ------------------------------------------------------------
NC_000023.11:15 ------------------------------------------------------------
NC_000004.12:c1 ------------------------------------------------------------


NC_000013.11:c3 ATTTATTTTATTTTTTAGAAAAATAACTAAACATGGGCAAAGGAGATCCTAAGAAGCCGA
NC_000001.11:33 ------------------------------------------------------------
NC_000023.11:15 ------------------------------------------------------------
NC_000004.12:c1 ------------------------------------------------------------


NC_000013.11:c3 GAGGCAAAATGTCATCATATGCATTTTTTGTGCAAACTTGTCGGGAGGAGCATAAGAAGA
NC_000001.11:33 --------------------------TTTTTGCTGAATTACAGAAACAAATTCAAGGAGC
NC_000023.11:15 ----------------------------------GATGTGCTAGGCCAAGATTCGGGAGC
NC_000004.12:c1 ----------------------------------AACTTGTAGGAATTAGTATCAATAGT
                                                  .*  *.. ...   *.  . .. **

NC_000013.11:c3 AGCACCCAGATGCTTCAGTCAACTTCTCAGAGTTTTCTAAGAAGTGCTCAGAGAGGTGGA
NC_000001.11:33 AGCAGCCAAATACCTATGTTGGCTTTAAAGAGTTCTCTAGAAAGTGTTCGGAAAAATGGA
NC_000023.11:15 TGTTGCCAGCCTCGTC-----------------------------------AAATATGGA
NC_000004.12:c1 AAATTTTGGGTTTTTT--------------------------------------------
                 .   .... . . *

NC_000013.11:c3 AGGTAAGAGGGCTTAAAACATGCTAACAAGGTAATTAAAAGACAGTTTCCAATTGAGGAT
NC_000001.11:33 GA----------------------------------------------------------
NC_000023.11:15 AGAGAAACAACCT-----------------GCGGTCAAAAGGGAGTGATTTGTTAA----
NC_000004.12:c1 ------------------------------------------------------------


NC_000013.11:c3 GCAAAAAAAAAGCCTAGTTGGCATTCTCGTAGTGGGACGCTATTACATAGCAAAAGACATT
NC_000001.11:33 -------------------------------------TCCATCTCAAAGC----------
NC_000023.11:15 ---------------------------GTGGTGCGCGTCTATCTCATAAC----------
NC_000004.12:c1 ------------------------------------------------------------


NC_000013.11:c3 GGTTTTGAGGATAATTTACTTAAATGTTACAACTTAAACTTACAAATAATTATTTTGTAG
```

```
NC_000001.11:33 ------------------------------------------------------------
NC_000023.11:15 --------------------TAGATGTACCAACC--------------------------
NC_000004.12:c1 --------------------AGGATGTTGCATTT--------------------------

NC_000013.11:c3 ACCATGTCTGCTAAAGAGAAAGGAAAATTTGAAGATATGGCAAAAGCGGACAAGGCCCGT
NC_000001.11:33 -------------ATGAAAAGGCCAAATATGAAGCCCTGGCCAAACTCGACAAAGCCCGA
NC_000023.11:15 -------------AGGGAAGGGCCAAGGATGGA---------------------------
NC_000004.12:c1 ----CGTTTTTTTAAAAAAAATTTTGTAATAAAATTATGT--------------------
                             * ...*..    .   *..*

NC_000013.11:c3 TATGAAAGAGAAATGAAAACCTATATCCCTCCCAAAGGGGAGACAAAAAAGAAGTTCAAG
NC_000001.11:33 TACCAGGAAGAAATGATGAATTATGT---TGGCAAGAGGAAGAAACGGAGAAAG---CGG
NC_000023.11:15 ----------AAGGGGTAACTTTTGT-GCTTCCAAAGTAGCTAAGCAGAAG------TGG
NC_000004.12:c1 ----------ATATTATTTCTATTGT-------------------------------CTT
                          * .  .    .  *.*

NC_000013.11:c3 GATCCCAATGCACCCAAGAGGCCTCCGTGAGTATCTTGCCTGTTTTTACTTCCCAGACAC
NC_000001.11:33 GATCCCCAGGAACCCAGACGGCCTCCA---------------------------------
NC_000023.11:15 GGGAGCAGTTTAGCCAGATGATCTTTG---------------------------------
NC_000004.12:c1 TGTCTTAATATGCTAAGTTAATTTTCA---------------------------------
                 .   . .   . . *.  ....*...

NC_000013.11:c3 GTTTTACAGTAGAATCTGAGAGAAATTTAGCAAGCTACTTTGTCAGTTTAGAGTGTAAAT
NC_000001.11:33 ------------------------------------------------------------
NC_000023.11:15 -------------ATTAGGCAAACATTGAG------------------------------
NC_000004.12:c1 ------------------------------------------------------------

NC_000013.11:c3 GTACAATCAAAGTTTCTTAGCTAATACTTGTTCATATTGGTTATATTTAAATAGTATAAA
NC_000001.11:33 ------------------------------------------------------------
NC_000023.11:15 ------------------------------------------------------------
NC_000004.12:c1 ------------------------------------------------------------

NC_000013.11:c3 ATTCCTGTTGGGTGGGAGTGTTCCCAGAGCATTTGAATTAGACATTTGGTCTCCTTTGCC
NC_000001.11:33 ------------------------------------------------------------
NC_000023.11:15 ------------------------------------------------------------
NC_000004.12:c1 ------------------------------------------------------------

NC_000013.11:c3 CAGTGTATCTCCTTTTGATCTTTTTATTTCTTGAAAAATACTATCCCTTTGAAATAGTGT
NC_000001.11:33 ------------------------------------------------------------
NC_000023.11:15 -----------------------------TTTTAAAGAGGCTGTCAAGTTGAGG------
NC_000004.12:c1 -----------------------------CTTTAAAAAAGCCAT----TTGAAG------

NC_000013.11:c3 AATTGTAGAATGTTCATCTAGGGTTCTAGCTAGTATAAATTAAATAGTTGTAAATTAAGC
NC_000001.11:33 ------------------------------------------------------------
NC_000023.11:15 -------------CCACTTGGTCCATTAGCTGG---------------------------
NC_000004.12:c1 ----------------------------ACCAG---------------------------

NC_000013.11:c3 TTTGGTTGTGAAGGATATTTAGTATATTATAGTATTTGCACCCTGTCCAATGCATCACAG
NC_000001.11:33 ------------------------------------------------------------
NC_000023.11:15 ------------------------------------------------------------
NC_000004.12:c1 ------------------------------------------------------------

NC_000013.11:c3 AAATTCACAGGCAGCTTTAAATAGCAATGCAGTGTACACTTGATAGTATTTGTTTTTGTG
NC_000001.11:33 ------------------------------------------------------------
NC_000023.11:15 ---------GGCAGC---------------------------------------------
NC_000004.12:c1 ------------AGC---------------------------------------------
```

```
NC_000013.11:c3 ATCTGTTAACTTAAAATCCTAAAATTAATTTTTTTTTTAGTTCGGCCTTCTTCCTCTTCT
NC_000001.11:33 -----------------------------------------TCATCCTTCCTACTCTTCT
NC_000023.11:15 --------------------AAGATCACTAC----------TCAACGTTTTCACAC-TGT
NC_000004.12:c1 --------------------TATGTTGATTTTTT-------TCGGTATTTCTG-------
                                                         **. . **...

NC_000013.11:c3 GCTCTGAGTATCGCCCAAAAATCAAAGGAGAACATCCTGGCCTGTC-----CATTGGTGA
NC_000001.11:33 GCCAAGACCACTATGCTCAGCTGAAGAGGGAGAACCCGAACTGGTC-----GGTGGTGCA
NC_000023.11:15 GGCAAGATTGCTCTTC-----TAGTGGAATAATGCCCTAGTTTCTCT--GAGATGATGTA
NC_000004.12:c1 -----------------------------------CCTAGTAGTTCTTAGACACAGTTGA
                                                   ** ...   **      .. .   *

NC_000013.11:c3 TGTTGCGAAGAAACTGGGAGAGATGT----GGAATAACACTGCTGCAGATGACAAGCAGC
NC_000001.11:33 GGTGGCCAAGGCCACAGGGAAGATGT----GGTCAACAGCGACAGACCTGGAGAAGCACC
NC_000023.11:15 AGTGGC-------------ATGATGT----TACCTAAGGC--------------------
NC_000004.12:c1 CCTAGT-------------AAAATGTTTGAGAATTAAAAC------------CAAACATG
                  * *.             . .****     .   *  .*

NC_000013.11:c3 CTTATGAAAAGAAGGCTGCGAAGCTGAAGGAAAAATATGAAAAGGTAAGAAGTGTGGGTT
NC_000001.11:33 CTTATGAGCAAAGAGTGGC-----------------------------------------
NC_000023.11:15 ------------------------------------------------------------
NC_000004.12:c1 CTCATA------------------------------------------------------


NC_000013.11:c3 TGCTTGGTAAAATGATGACAAGTACGCCAGATGTATGATTGTACTTAGTTTGAGGTGTAA
NC_000001.11:33 ------------------------------------------------------------
NC_000023.11:15 --------------------------------------TTAGGCTTAGCTTGA-------
NC_000004.12:c1 ------------------------------------------------------------


NC_000013.11:c3 TAAGTTTTTAGGGTAACAGCTACATTAAGTATGGTGTTGATATAAGTCCTCATCCTTCAA
NC_000001.11:33 ----TCTCCTGA------------------------------------------------
NC_000023.11:15 ----TTTCTGGG------------------------------------------------
NC_000004.12:c1 ----TTTGCAAA------------------------------------------------
                    *.* . ..

NC_000013.11:c3 AGAATGCAGAGGACCAAATAAATTAGGGTTTTTTTGACTAAAATGTAATCAGACTCAGAC
NC_000001.11:33 ------------------------------------------------------------
NC_000023.11:15 ------------------------------------------------------------
NC_000004.12:c1 ------------------------------------------------------------


NC_000013.11:c3 AAAGGCTGTGTACATTTATGTTGGTTTTGTTATTCCCCAGTATCTTGAAGTTCATGAAAA
NC_000001.11:33 ------------------------------------------------------------
NC_000023.11:15 -----------------------------------CCCACTGTCT---------------
NC_000004.12:c1 ------------------------------------------------------------


NC_000013.11:c3 TGTTGGTAGTCACTTCAAGTCAAAAATGAGCATTTTCAAATGGCTTGGCATACAGTACAA
NC_000001.11:33 ------------------------------------------------------------
NC_000023.11:15 ------------------------------------------------------------
NC_000004.12:c1 ------------------------------------------------------------


NC_000013.11:c3 AAACAGGCTAGACAAAGTAATATAGGCTATATTTTTCTTAGTCATATCCTGAAACATTTA
NC_000001.11:33 ------------------------------------------------------------
NC_000023.11:15 -------------------------------------------GTGTTCTTAAG------
NC_000004.12:c1 -------------------------------------------ATGTTCTTTAA------


NC_000013.11:c3 TGTTCTTTTCCTTTAGATACTCAAAAAACCACAGCATCACTAAGTTAAATTACAAGTCTG
NC_000001.11:33 ------------------------------------------------------------
NC_000023.11:15 ------------------------------------------------------------
```

```
NC_000004.12:c1 ------------------------------------------------------------


NC_000013.11:c3 CTGCTCTGTCCAGTAAATTAATAAGATTAAGGAAATCTATAACTCTTATAGTTCAGTAAA
NC_000001.11:33 ------------------------------------------------------------
NC_000023.11:15 ------------------------------------------------------------
NC_000004.12:c1 ------------------------------------------------------------


NC_000013.11:c3 TTGAAATATTAAATACTTAATTTTCAGCTTTAGTCATTCTGAAAAGTGTTTATTTCTAGA
NC_000001.11:33 -----GAGCTAAGTACTT------------------------------------------
NC_000023.11:15 -----ATGCCAACCTGTTGCTT--------------------------------------
NC_000004.12:c1 -----AAGTTACATGTTGAACT--------------------------------------
                     . ...*  .  *

NC_000013.11:c3 TGTTTCTTAACCTAATTGCATGTTTATTGACAAATTACCTTTTTTTTTTAAGACCACATT
NC_000001.11:33 ------------------------------------------------------------
NC_000023.11:15 ---------------------------------------TTTTTTTTTT-----------
NC_000004.12:c1 ------------------------------------------------------------


NC_000013.11:c3 TCCTACTAAGGATTAAGGTCTGACAGTGTAAACCTGTAGAGTGCTTTTTTGCATTCAGAA
NC_000001.11:33 ------------------------------------------------------------
NC_000023.11:15 --------------------------------------------TTTCCCCCATTTAAAA
NC_000004.12:c1 ------------------------------------CAGTGAACTTT-------------


NC_000013.11:c3 GGTGGCAGTGTCTACCCTTTAATCAAAGTCTCTACATTCTGGTTTTAATAGAGTTAGGAT
NC_000001.11:33 ------------------------------------------------------------
NC_000023.11:15 G--GATAGTACCTACTCCCT----------------------------------------
NC_000004.12:c1 ------------------------------------------------------------


NC_000013.11:c3 GTGGTACATAATTGCACCTCAATGAGGCATAACTTTGCAAATATTAGACTATGCCATTTC
NC_000001.11:33 ------------------------------------------------------------
NC_000023.11:15 ------------------------------------------------------------
NC_000004.12:c1 ------------------------------------------------------------


NC_000013.11:c3 ATGAGTTATAGATTGTTATAATGATCTTGTATTTTTATGTTCATTTATTGAAGTTCTAGT
NC_000001.11:33 ------------------------------------------------------------
NC_000023.11:15 ------------------------------------------------------------
NC_000004.12:c1 ------------------------------------------------------------


NC_000013.11:c3 TATTTCTGGAGTTGCTGTGGATCTACAGATACGTGATATTTTGGTATAACTAGAATCTTG
NC_000001.11:33 -------------------------------------------------CGAGGAACTTG
NC_000023.11:15 -------------------------------------------------CTAACCACCTC
NC_000004.12:c1 -------------------------------------------------ATAAGAATTTA
                                                                   *.   ..*

NC_000013.11:c3 ATTTCTTTCATAAAGTTCTGCCATGTTCTATTTCTTTCCTTAATGTTTTTTTCTTCCCTA
NC_000001.11:33 AACTCTACCGTAAA----------------------------------------------
NC_000023.11:15 ACCCCATTCTTGAA--------------------------------------------TG
NC_000004.12:c1 TGCAGTTTTACAGA-----------------------------------------ACGTT
                  .    .. ...*

NC_000013.11:c3 CTGTTTTATCCTCCCTTTGCTTTGGAAGGATATTGCTGCATATCGAGCTAAAGGAAAGCC
NC_000001.11:33 ------------------------------------------------------------
NC_000023.11:15 ACATTTTATCCTTC----------------------------------------------
NC_000004.12:c1 AAGTTTTGTACTTG----------------------------------------------


NC_000013.11:c3 TGATGCAGCAAAAAAGGGAGTTGTCAAGGCTGAAAAAAGCAAGAAAAAGAAGGAAGAGGA
```

```
NC_000001.11:33 ------------------------------------------------------------
NC_000023.11:15 -------------------------------GGAAAGAACA-------------------
NC_000004.12:c1 ------------------------------------------------------------

NC_000013.11:c3 GGAAGATGAGGAAGATGAAGAGGATGAGGAGGAGGAGGAAGATGAAGAAGATGAAGATGA
NC_000001.11:33 ------------------------------------------------------------
NC_000023.11:15 ------------------------------------------------------------
NC_000004.12:c1 ------------------------------------------------------------

NC_000013.11:c3 AGAAGAAGATGATGATGATGAATAAGTTGGTTCTAGCGCAGTTTTTTTTTTCTTGTCTAT
NC_000001.11:33 ------------------------------------------------------------
NC_000023.11:15 ------------------------------------------------------------
NC_000004.12:c1 ------------------------------------------------------------

NC_000013.11:c3 AAAGCATTTAACCCCCCTGTACACAACTCACTCCTTTTAAAGAAAAAAATTGAAATGTAA
NC_000001.11:33 ------------------------------------------------------------
NC_000023.11:15 ------------------------------------------------------------
NC_000004.12:c1 ------------------------------------------------------------

NC_000013.11:c3 GGCTGTGTAAGATTTGTTTTTAAACTGTACAGTGTCTTTTTTTGTATAGTTAACACACTA
NC_000001.11:33 ------------------------------------------------------------
NC_000023.11:15 ------------------------------------------------------------
NC_000004.12:c1 ------------------------------------------------------------

NC_000013.11:c3 CCGAATGTGTCTTTAGATAGCCCTGTCCTGGTGGTATTTTCAATAGCCACTAACCTTGCC
NC_000001.11:33 ------------------------------------------------------------
NC_000023.11:15 ------------------------------------------------------------
NC_000004.12:c1 ------------------------------------------------------------

NC_000013.11:c3 TGGTACAGTATGGGGGTTGTAAATTGGCATGGAAATTTAAAGCAGGTTCTTGTTGGTGCA
NC_000001.11:33 ------------------------------------------------------------
NC_000023.11:15 ------------------------------------------------------------
NC_000004.12:c1 ------------------------------------------------------------

NC_000013.11:c3 CAGCACAAATTAGTTATATATGGGGATGGTAGTTTTTTCATCTTCAGTTGTCTCTGATGC
NC_000001.11:33 ------------------------------------------------------------
NC_000023.11:15 ------------------------------------------------------------
NC_000004.12:c1 ------------------------------------------------------------

NC_000013.11:c3 AGCTTATACGAAATAATTGTTGTTCTGTTAACTGAATACCACTCTGTAATTGCAAAAAAA
NC_000001.11:33 ------------------------------------------------------------
NC_000023.11:15 ------------------------------------------------------------
NC_000004.12:c1 ------------------------------------------------------------

NC_000013.11:c3 AAAAAAAAGTTGCAGCTGTTTTGTTGACATTCTGAATGCTTCTAAGTAAATACAATTTTT
NC_000001.11:33 ------------------------------------------------------------
NC_000023.11:15 ------------------------------------------------------------
NC_000004.12:c1 ------------------------------------------------------------

NC_000013.11:c3 TTTATTAGTATTGTTGTCCTTTTCATAGGTCTGAAATTTTTCTTCTTGAGGGGAAGCTAG
NC_000001.11:33 ------------------------------------------------------------
NC_000023.11:15 ------------------------------------------------------------
NC_000004.12:c1 ------------------------------------------------------------
```

```
NC_000013.11:c3 TCTTTTGCTTTTGCCCATTTTGAATCACATGAATTATTACAGTGTTTATCCTTTCATATA
NC_000001.11:33 ------------------------------------------------------------
NC_000023.11:15 ------------------------------------------------------------
NC_000004.12:c1 ------------------------------------------------------------


NC_000013.11:c3 GTTAGCTAATAAAAAGCTTTTGTCTACACACCCTGCATATCATAATGGGGGTAAAGTTAA
NC_000001.11:33 ------------------------------------------------------------
NC_000023.11:15 ------------------------------------------------------------
NC_000004.12:c1 ------------------------------------------------------------


NC_000013.11:c3 GTTGAGATAGTTTTCATCCATAACTGAACATCCAAAATCTTGATCAGTTAAGAAATTTCA
NC_000001.11:33 ------------------------------------------------------------
NC_000023.11:15 ------------------------------------------------------------
NC_000004.12:c1 ------------------------------------------------------------


NC_000013.11:c3 CATAGCCCACTTACATTTACAAACTGAAGAGTAATCAATCTACTCAAAGCATGGGATTAT
NC_000001.11:33 ------------------------------------------------------------
NC_000023.11:15 ------------------------------------------------------------
NC_000004.12:c1 ------------------------------------------------------------


NC_000013.11:c3 TAGAATCAAACATTTTGAAAGTCTGTCCTTGAAGGACTAATAGAAAAGTATGTTCTAACC
NC_000001.11:33 ------------------------------------------------------------
NC_000023.11:15 ------------------------------------------------------------
NC_000004.12:c1 ------------------------------------------------------------


NC_000013.11:c3 TTTACATGAGGACTCTATTCTTTAACTCCCATTACCATGTAATGGCAGTTATATTTTGCA
NC_000001.11:33 ----------------------------------CAATGTAATGCCAG------------
NC_000023.11:15 ----------------------------AGGCTGTGATGTAGTGACTATTGTCT------
NC_000004.12:c1 ----------------------------ACGTTTCTGTTTATTAGCTA------------
                                                  . .* ** *. * .

NC_000013.11:c3 GTTCCCACATTAAAGAAGACCTGAGAATGTATCCCCAAAAGCGTGAGCTTAAAATACAAG
NC_000001.11:33 ------------------------------------------------------------
NC_000023.11:15 ------------------------------------------------------------
NC_000004.12:c1 ------------------------------------------------------------


NC_000013.11:c3 ACTGCCATATTAAATTTTTTGTTGACATTAGTCTCAGTGAAGACTATGAAAATGCTGGCT
NC_000001.11:33 ------------------------------------------------------------
NC_000023.11:15 ------------------------------------------------------------
NC_000004.12:c1 ------------AATT--------------------------------------------


NC_000013.11:c3 ATAGATGTCTTTTCCCATTTATCTAAATATGGACTGCTCAGGAAACGAGACTTTCCATTA
NC_000001.11:33 ------------------------------------------------------------
NC_000023.11:15 ------------------------------------------------------------
NC_000004.12:c1 ------------------------------------------------------------


NC_000013.11:c3 CAAGTATTTTTAATTAATTGGGCCAGCTTTTCAAACAAAGATGCCACATTCAAAATAGGG
NC_000001.11:33 ------------------------------------------------------------
NC_000023.11:15 ------------------------------------------------------------
NC_000004.12:c1 ------------------------------------------------------------


NC_000013.11:c3 TATATTTTCCTATATTACGGTTTGCCCCTTTATAAATCCAAGTAGATAGGAAGAAAGAAG
NC_000001.11:33 ------------------------------------------------------------
NC_000023.11:15 ------------------------------------------------------------
```

```
NC_000004.12:c1 ------------------------------------------------------------


NC_000013.11:c3 ACAAACTTTGCATCTCAGTATGAATTATTCAATTTATTTGAATGATTTTTCTTTACAAAA
NC_000001.11:33 ------------------------------------------------------------
NC_000023.11:15 ------------------------------------------------------------
NC_000004.12:c1 ------------------------------------------------------------


NC_000013.11:c3 CAAACTCATTCATTAGTCATGTTTATCTGCTTAGGAGTTTAGGGAACAATTTGGCAATTT
NC_000001.11:33 ------------------------------------------------------------
NC_000023.11:15 ------------------------------------------------------------
NC_000004.12:c1 ------------------------------------------------------------


NC_000013.11:c3 TGTGGTTTTCGAGATTATCGTTTTCTTAAAGTGCCAGTATTTTAAAATAGCGTTCTTGTA
NC_000001.11:33 ------------------------------------------------------------
NC_000023.11:15 ------------------------------------------------------------
NC_000004.12:c1 ------------------------------------------------------------


NC_000013.11:c3 ATTTTACACGCTTTTGTGATGGAGTGCTGTTTTGTTATATAATTTAGACTTGGATTCTTT
NC_000001.11:33 ------------------------------------------------------------
NC_000023.11:15 -----------------------------------------------------GTGTCTC
NC_000004.12:c1 -----------------------------------------------------GTTCCTC


NC_000013.11:c3 CCATTTGCATTTGTTTATGTAATTTCAGGAGGAATACTGAACATCTGAGTCCTGGATGAT
NC_000001.11:33 ---------------------------GAAGAAGTACCGAATGTCAG-------------
NC_000023.11:15 CTGTGTGTGTCTGTTCTTGTCA---------------CAAATGTATT-------------
NC_000004.12:c1 AGGTGTGTGTATATATATATACA--------------TATATATATA-------------
                                                     .. *..*

NC_000013.11:c3 ACTAATAAACTAATAATTGCAGAGGTTTTAAATACTAGTTAAATGGCTTTCACTTAAGAA
NC_000001.11:33 ------------------------------------------------------------
NC_000023.11:15 ------------------------------------------------------------
NC_000004.12:c1 ------------------------------------------------------------


NC_000013.11:c3 CTTAAGATTTTGTTACATATTTTTAAATCTTGTTTCTAATAATACCTCTTAGCAGTACCT
NC_000001.11:33 ------------------------------------------------------------
NC_000023.11:15 ------------------------------------------------------------
NC_000004.12:c1 ------------------------------------------------------------


NC_000013.11:c3 TTTAAATAAGTATAAGGGATGGCAAAGTTTTTCCCTTTAAAAATACTCACTTTATGCTTA
NC_000001.11:33 ------------------------------------------------------------
NC_000023.11:15 ------------------------------------------------------------
NC_000004.12:c1 ------------------------------------------------------------


NC_000013.11:c3 TAAATAGGTTAATGGGCTGATAAAAGGTTTTGTCAAACATTGCAAGTATTCGGTGCTATA
NC_000001.11:33 ------------------------------------------CTAGAAACCGGTGC----
NC_000023.11:15 ------------------------------------------------------------
NC_000004.12:c1 ------------------------------------------------------------


NC_000013.11:c3 TATAAAGGAGGAAAAACTAGTTTTACTTTCAGAATGATTTAAACAAGATTTTTAAAAACA
NC_000001.11:33 ---AGAGGGAAAAGAGTCAG----------------------------------------
NC_000023.11:15 ------------------------------------------------------------
NC_000004.12:c1 ------------------------------------------------------------


NC_000013.11:c3 AGATACATGCAAGCGAACAGCAGGGTTAGTGATAGGCTGCAATTGTGTCGAACATCAGAT
```

```
NC_000001.11:33 -------------------GCAGAGCTGATG-----------------------------
NC_000023.11:15 --------------------TGGGGACGTTG-----------------------------
NC_000004.12:c1 ------------------------------------------------------------


NC_000013.11:c3 TTTTTGTTAAGAGGAGCAAATGACTCAATCTGATTTAGATGGAAGTTTCTACTGTATAGA
NC_000001.11:33 ------------------------------------------------------------
NC_000023.11:15 ------------------------------------------------------------
NC_000004.12:c1 ------------------------------------------------------------


NC_000013.11:c3 AATCACCATTAATCACCAACATTAATAATTCTGATCCATTTAAAATGAATTCTGGCTCAA
NC_000001.11:33 --------------------------------GATCCAGTTTGAA---------------
NC_000023.11:15 --------------------------------GATGCATTC-------------------
NC_000004.12:c1 --------------------------------TATATATAT-------------------
                                                 ** .*  .

NC_000013.11:c3 GGAGAATTTGTAACTTTAGTAGGTACGTCATGACAACTACCATTTTTTTAAGATGTTGAG
NC_000001.11:33 ------------------------------------------------------------
NC_000023.11:15 ------------------------------------------------------------
NC_000004.12:c1 ------------------------------------------------------------


NC_000013.11:c3 AATGGGAACAGTTTTTTTAGGGTTTATTCTTGACCACAGATCTTAAGAAAATGGACAAAA
NC_000001.11:33 ------------------------------------------------------------
NC_000023.11:15 ------------------------------------------------------------
NC_000004.12:c1 ------------------------------------------------------------


NC_000013.11:c3 CCCCTCTTCAATCTGAAGATTAGTATGGTTTGGTGTTCTAACAGTATCCCCTAGAAGTTG
NC_000001.11:33 ------------------------------------------------------------
NC_000023.11:15 ------------------------------------------------------------
NC_000004.12:c1 ------------------------------------------------------------


NC_000013.11:c3 GATGTCTAAAACTCAAGTAAATGGAAGTGGGAGGCAATTTAGATAAGTGTAAAGCCTTGT
NC_000001.11:33 ------------------------------------------------------------
NC_000023.11:15 ------------------------------------------------------------
NC_000004.12:c1 ------------------------------------------------------------


NC_000013.11:c3 AACTGAAGATGATTTTTTTTAGAAAGTGTATAGAAACTATTTTAATGCCAAGATAGTTAC
NC_000001.11:33 ------------------------------------------------------------
NC_000023.11:15 ------------------------------------------------------------
NC_000004.12:c1 ------------------------------------------------------------


NC_000013.11:c3 AGTGCTGTGGGGTTTAAAGACTTTGTTGACATCAAGAAAAGACTAAATCTATAATTAATT
NC_000001.11:33 ------------------------------------------------------------
NC_000023.11:15 ------------------------------------------------------------
NC_000004.12:c1 ------------------------------------------------------------


NC_000013.11:c3 GGGCCAACTTTTAAAATGAAGATGCTTTTTAAAACTAATGAACTAAGATGTATAAATCTT
NC_000001.11:33 ------------------------------------------------------------
NC_000023.11:15 ------------------------------------------------------------
NC_000004.12:c1 ------------------------------------------------------------


NC_000013.11:c3 AGTTTTTTTGTATTTTAAAGATAGGCATATGGCATATTGATTAACGAGTCAAATTTCCTA
NC_000001.11:33 ------------------------------------------------------------
NC_000023.11:15 ------------------------------------------------------------
NC_000004.12:c1 ------------------------------------------------------------
```

```
NC_000013.11:c3 ACTTTGCTGTGCAAAGGTTGAGAGCTATTGCTGATTAGTTACCACAGTTCTGATGATCGT
NC_000001.11:33 ------------------------------------------------------------
NC_000023.11:15 ------------------------------------------------------------
NC_000004.12:c1 ------------------------------------------------------------

NC_000013.11:c3 CCCATCACAGTGTTGTTAATGTTTGCTGTATTTATTAATTTTCTTAAAGTGAAATCTGAA
NC_000001.11:33 ------------------------------------------------------------
NC_000023.11:15 -------------------------------------ATTTTC-----------------
NC_000004.12:c1 ------------------------------------------------------------

NC_000013.11:c3 AAATGAAATTTGTGTGTCCTGTGTACCCGAGGGGTAATGATTAAATGATAAAGATAAGAA
NC_000001.11:33 ------------------------------------------------------------
NC_000023.11:15 ------------------------------------------------------------
NC_000004.12:c1 ------------------------------------------------------------

NC_000013.11:c3 AAGCGCCCATGTAACACAAACTGCCATTCAACAGGTATTTCCCTTACTACCTAAGGAATT
NC_000001.11:33 ------------AAAACAAAATGCCATTCA------------------------------
NC_000023.11:15 ---------TGTAATAAAGTTTCTTAATCA------------------------------
NC_000004.12:c1 ------------------------------------------------------------

NC_000013.11:c3 GTAACCATTGCTCAGACATTGTAGGATTTAACTATGTTGAAAACTACAGGAGAGGCCGGG
NC_000001.11:33 ------------------------------------------------------------
NC_000023.11:15 ------------------------------------------------------------
NC_000004.12:c1 ------------------------------------------------------------

NC_000013.11:c3 CGCAGTGGCTCACGCCTGTAATCCCAGCACTTTGGGAGGCCAAGGCGGGCAGATCACGAG
NC_000001.11:33 ------------------------------------------------------------
NC_000023.11:15 ------------------------------------------------------------
NC_000004.12:c1 ------------------------------------------------------------

NC_000013.11:c3 GTCAGGAGATTGAGACCATCCTGGCTAACGTGGTGAAACCCCGCCTCTACTAAAAATACA
NC_000001.11:33 ------------------------------------------------------------
NC_000023.11:15 ------------------------------------------------------------
NC_000004.12:c1 ------------------------------------------------------------

NC_000013.11:c3 AAAAATTAGCCAAGCGTGGTGCTGGGCGCCTGTAGTCCCAGTAACTCAGGAGGCTGAGGC
NC_000001.11:33 ------------------------------------------------------------
NC_000023.11:15 ------------------------------------------------------------
NC_000004.12:c1 ------------------------------------------------------------

NC_000013.11:c3 AGGAGAATGGCGTGAACCCGGGAGGCGGAGGTTGCAGTGAGCCGAGATTGTGCCACTGCA
NC_000001.11:33 ------------------------------------------------------------
NC_000023.11:15 ------------------------------------------------------------
NC_000004.12:c1 ------------------------------------------------------------

NC_000013.11:c3 CTCCAGCCTGGGTGACAGAGCAAGACTCCATCTCAAAAAAAAAAAAAAAACACAGGAGAG
NC_000001.11:33 ------------------------------------------------------------
NC_000023.11:15 ------------------------------------------------------------
NC_000004.12:c1 ------------------------------------------------------------

NC_000013.11:c3 ACAACTGGTTTTTGAATGAAATACATGGGTACTGCCTTGCTTGACATCACATAGTCCTTG
NC_000001.11:33 ------------------------------------------------------------
NC_000023.11:15 ------------------------------------------------------------
```

```
NC_000004.12:c1 ------------------------------------------------------------


NC_000013.11:c3 ATGAAAGTTCACATTTAGGTCTGCTTGGTACAATACGCCTCCTAAAAAGGTCCTTGATGA
NC_000001.11:33 ------------------------------------------------------------
NC_000023.11:15 -----------------------------------CTCTTCCCAAAAA------------
NC_000004.12:c1 ------------------------------------------------------------


NC_000013.11:c3 AAGTTCACATTTAGGTCTGCTTGGTACAACACGCCTCCTGAAAGGGTCTGATAGCTTTCA
NC_000001.11:33 ------------------------------------------------------------
NC_000023.11:15 ------------------------------------------------------------
NC_000004.12:c1 ------------------------------------------------------------


NC_000013.11:c3 GTAGCAGTAAGACACTTGCATGTGATGGTAAGGTATCTGCAAATTTGCACACACCGTACA
NC_000001.11:33 ----------------------------------------------------ACCGTA--
NC_000023.11:15 ------------------------------------------------------------
NC_000004.12:c1 ------------------------------------------------------------


NC_000013.11:c3 CAGCTTAAGTCTTAGAATTAACTTGCTAAAATGTGAGCCTTTGGTAATTAGGCTGTTTTA
NC_000001.11:33 ------------------------------------------------------------
NC_000023.11:15 ------------------------------------------------------------
NC_000004.12:c1 ------------------------------------------------------------


NC_000013.11:c3 TTAGGGAGTGTGATAATATTTGAATTTCTTTTCATATTTGTGCTTTGTGTCATTTTCAAA
NC_000001.11:33 ------------------------------------------------------------
NC_000023.11:15 ------------------------------------------------------------
NC_000004.12:c1 ------------------------------------------------------------


NC_000013.11:c3 TGACCCTTGAAATGTATTTTAAAAGTAGATAAAAGCCAGAAAGTGATTTGATTGTCTATC
NC_000001.11:33 ------------------------------------------------------------
NC_000023.11:15 ------------------------------------------------------------
NC_000004.12:c1 ------------------------------------------------------------


NC_000013.11:c3 CAGCAAAAGATGGTGGTTCATTTTCTTGTCCTCTTAATACCAAGGCAGTGCTAATAACAC
NC_000001.11:33 ------------------------------------------------------------
NC_000023.11:15 ------------------------------------------------------------
NC_000004.12:c1 ------------------------------------------------------------


NC_000013.11:c3 TTGCCACAACTTGGGAAATTCCATGGGTCTATGCCACATTGCTCCCAGAGTAATGAGGCA
NC_000001.11:33 ------------------------------------------------------------
NC_000023.11:15 ------------------------------------------------------------
NC_000004.12:c1 ------------------------------------------------------------


NC_000013.11:c3 AAATAGTGCTCTGTTATAGAATTGCTTGTTTCACGATACATCATGACAGATAACCATACA
NC_000001.11:33 ------------------------------------------------------------
NC_000023.11:15 ------------------------------------------------------------
NC_000004.12:c1 ------------------------------------------------------------


NC_000013.11:c3 ACATGGAATGACACAAACATAATATGCCACACTCCAGAATATGTAATGCTCGTCTTCCAG
NC_000001.11:33 ------------------------------------------------------------
NC_000023.11:15 ------------------------------------------------------------
NC_000004.12:c1 ------------------------------------------------------------


NC_000013.11:c3 GGGGGTTCAGTCTAAGGTAATCTCTACCAGGAAGAAATGCTAGATGACTTTAGACATGTG
```

```
NC_000001.11:33 ------------------------------------------------------------
NC_000023.11:15 ------------------------------------------------------------
NC_000004.12:c1 ------------------------------------------------------------

NC_000013.11:c3 CATTGGTTTGGACCTTCTAATTAGTGGAATTTTTACTTATTTTGACATGAGAGATTACAT
NC_000001.11:33 ------------------------------------------------------------
NC_000023.11:15 ------------------------------------------------------------
NC_000004.12:c1 ------------------------------------------------------------

NC_000013.11:c3 AGAATCTCTATGTTGCCCAGGTTGGTCTCCAAATCTGCTCAAACAATCCTCCCGCCTCAG
NC_000001.11:33 ------------------------------------------------------------
NC_000023.11:15 ------------------------------------------------------------
NC_000004.12:c1 ------------------------------------------------------------

NC_000013.11:c3 TTTCTCAAGTAGCTGGGATTACAGGGACACACCACTGTGCTTAGCTTAAATAGTTGAATT
NC_000001.11:33 ------------------------------------------------------------
NC_000023.11:15 ------------------------------------------------------------
NC_000004.12:c1 ------------------------------------------------------------

NC_000013.11:c3 TATTGGGCATCCACTGAAAAGAAGGAAGTAGGAATAAAATCTGCAATAGTATGACTAAAC
NC_000001.11:33 ------------------------------------------------------------
NC_000023.11:15 ------------------------------------------------------------
NC_000004.12:c1 ------------------------------------------------------------

NC_000013.11:c3 CTGTAAGTGAGCATGAGCAGTGGTGGGCAAGGGTATGCAGACTCTGGAATCCAGAAATAG
NC_000001.11:33 ------------------------------------------------------------
NC_000023.11:15 ------------------------------------------------------------
NC_000004.12:c1 ------------------------------------------------------------

NC_000013.11:c3 AAAGTCAGTGGAAAGCCAGGCACAGTGGCTCACACTTATAATCCCAGCACTTTGGGAGGC
NC_000001.11:33 ------------------------------------------------------------
NC_000023.11:15 ------------------------------------------------------------
NC_000004.12:c1 ------------------------------------------------------------

NC_000013.11:c3 CGAGGTGGGAGGATCACTTGAGGCCAGGAGTTTGACACTAGCCTGGGCAACATAGTGAGG
NC_000001.11:33 ------------------------------------------------------------
NC_000023.11:15 ------------------------------------------------------------
NC_000004.12:c1 ------------------------------------------------------------

NC_000013.11:c3 CCTTGTCTACTAGAAATAAATAATTTTGCAGATATACTAAATACCATTTTAAAAATAAGT
NC_000001.11:33 ------------------------------------------------------------
NC_000023.11:15 ------------------------------------------------------------
NC_000004.12:c1 ------------------------------------------------------------

NC_000013.11:c3 GGAAAAGGGATTAGAGAGATCCTTGTAAATTATGTACTCATATTCATTGTTCTCTTTAGT
NC_000001.11:33 ------------------------------------------------------------
NC_000023.11:15 ------------------------------------------------------------
NC_000004.12:c1 ------------------------------------------------------------

NC_000013.11:c3 CACATAATTCAGTCATGTACGTGACATTCCACAGTATGGTGCTCCATAATACATATTTTT
NC_000001.11:33 ------------------------------------------------------------
NC_000023.11:15 ------------------------------------------------------------
NC_000004.12:c1 ------------------------------------------------------------
```

```
NC_000013.11:c3 TCTTTTTTCGATCTCACTTGTATATGATCCATAATATTTTTCATCATTCCCTTACTGATG
NC_000001.11:33 ------------------------------------------------------------
NC_000023.11:15 ------------------------------------------------------------
NC_000004.12:c1 ------------------------------------------------------------

NC_000013.11:c3 GACATTTAGATTGTTTCCTATTTTTTGCTGTTTAACAGTACTGCGCTGAACATCTGTACA
NC_000001.11:33 ------------------------------------------------------------
NC_000023.11:15 ------------------------------------------------------------
NC_000004.12:c1 ------------------------------------------------------------

NC_000013.11:c3 TTTCTCTCAGGCATGTGTGGGTCTTCAGATACCTCAAAAGTAGAACTGCTGAGTCAAAAG
NC_000001.11:33 ------------------------------------------------------------
NC_000023.11:15 ------------------------------------------------------------
NC_000004.12:c1 ------------------------------------------------------------

NC_000013.11:c3 ACATTTGCATTTAACATTTTGGAAGATGTTGATAAATTGCACCCCACCACCCCCCCCCCC
NC_000001.11:33 ------------------------------------------------------------
NC_000023.11:15 ------------------------------------------------------------
NC_000004.12:c1 ------------------------------------------------------------

NC_000013.11:c3 CGCCCACAAAAGCTGTTATTTATGCTGTGAAACATTTTAACAATGATTTTGCAATTAA
NC_000001.11:33 ----------------------------------------------------------
NC_000023.11:15 ----------------------------------------------------------
NC_000004.12:c1 ----------------------------------------------------------
```
